# Beyond Friedel–Crafts: Spontaneous and Fluoride-Catalyzed Acylation of 2‑(Trialkylsilyl)pyridines

**DOI:** 10.1021/acs.orglett.5c03140

**Published:** 2025-09-20

**Authors:** Jan Dudziński, Damian Antoniak, Kacper Błaziak, Michał Barbasiewicz

**Affiliations:** † 49605 University of Warsaw, Faculty of Chemistry, Pasteura 1, 02-093 Warsaw, Poland; ‡ University of Warsaw, Biological and Chemical Research Centre, Żwirki i Wigury 101, 02-089 Warsaw, Poland

## Abstract

2-(Trialkylsilyl)­pyridines react spontaneously with acyl chlorides to give 2-pyridyl ketones in high yields. The process consists of four elementary steps of *N*-acylation, desilylation, *C*-acylation, and *N*-deacylation and results in a selective monosubstitution at the carbonyl group. The key step in this mechanism is the intrinsic generation of a stabilized ylide (Hammick intermediate), which acts as a nucleophile. For the functionalization of complex molecules, an alternative fluoride-catalyzed protocol utilizing more stable acyl fluorides has been developed.

## Graphical abstract

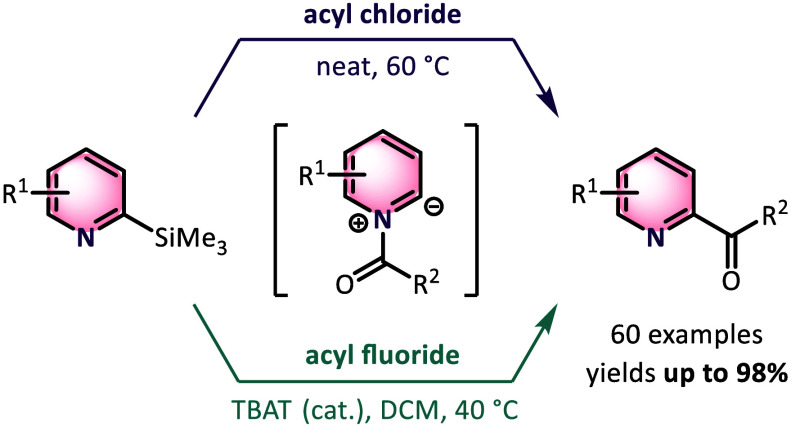

Functionalization of the pyridine ring plays a critical role in the synthesis of pharmaceuticals, pesticides, ligands, functional materials, etc., and remains an emerging field of synthetic methodology. Although electrophilic substitution reactions usually fail for electron-deficient heteroarenes,^1^ alternative nucleophilic,^2^ radical, photochemical, and electrochemical transformations,^3^ transition metal-catalyzed protocols,^4^ temporary dearomatizations,^5^ installation of P-^6^ and S-based auxiliaries,^7^ activation by N-functionalization,^8^ and a very potent class of the metalation-trapping sequences were developed for the task.^9^ In the latter case, *thermodynamic* deprotonations usually affect the distant C4 and C3 positions of the pyridine ring,^10^ whereas hydrogens adjacent to the heteroatom display a diminished acidity due to the destabilizing effect of its lone electron pair.^11^ Furthermore, C2-nucleophilic reagents often display undesired properties in the transition-metal-catalyzed cross-coupling reactions, recognized in the literature as the “2-pyridyl problem”.^12^

Effective equivalents of unstable organometallic species are arylsilanes (ArSiR_3_), capable of reacting via direct electrophilic *ipso*-substitution^13^ (e.g., Friedel–Crafts acyl-desilylation)^14^ or generating highly reactive aryl anions by removal of the silyl group with a Lewis base. Since the ease of heterolytic C–Si bond cleavage correlates with the stabilization of released carbanion, dedicated classes of precursors for unsupported phenyl anions (e.g., siloxanes^15^ and *N*-silylated diazenes),^16^ and for analogues with adjacent electron withdrawing groups (EWGs)^17^ or η^6^-complexed aromatic rings^18^ were reported in the literature (Scheme 1, top). Interestingly, anions of some heteroaromatic systems may also display a unique stabilization with ylide-carbene resonance. Deprotonation of five-membered ring azolium salts forms *N*-heterocyclic carbenes (NHCs), which remain stable after isolation^19^ and find numerous applications as ligands and organocatalysts.^20^ Enhanced stabilization of heteroaryl ylides also enables acylation of imidazoles and thiazoles, which runs via C2-deprotonation of *N*-acylated intermediates with a weak amine base (Regel-Büchel acylation; Scheme 1, middle).^21^ Surprisingly, much less recognized are ylides generated from six-membered-ring pyridinium salts. In 1937, Hammick reported^22a,22b^ decarboxylation of 2-pyridinecarboxylic (2-picolinic) acid and postulated the formation of a zwitterionic intermediate^22c,23^ that facilitates the process. Later, BF_3_-complexation of the nitrogen atom was demonstrated to redirect deprotonation of the pyridine ring to the nearest C2 position.^9a,24^ Finally, pyridinylidene-metal complexes^25^ and a stable, crystalline (naphtho)­pyridinylidene^26^ were synthesized and characterized with X-ray studies. Importantly, estimated Brønsted basicity of pyridin-2-ylidenes is ca. 8 p*K*_a_ units higher than that of analogous imidazol-2-ylidenes,^27^ and thus their generation requires use of strong bases or alternative decarboxylation and desilylation methods.^22a,22b,26,28^ In this context, nucleophilic ylide-type character of the species was demonstrated on reactions of 2-(trimethylsilyl)­pyridines with aldehydes, which yielded secondary (2-pyridyl)­carbinols.^29^ Interestingly, analogous acylation reactions have remained virtually unexplored,^30^ and only a conceptually related synthesis of 2-picolinic esters involved the formation of the intermediate zwitterionic adduct with CO_2_ (Scheme 1, bottom).^31^ In the current report, we demonstrate application of the potent species for the synthesis of 2-pyridyl ketones.

**Scheme 1:**
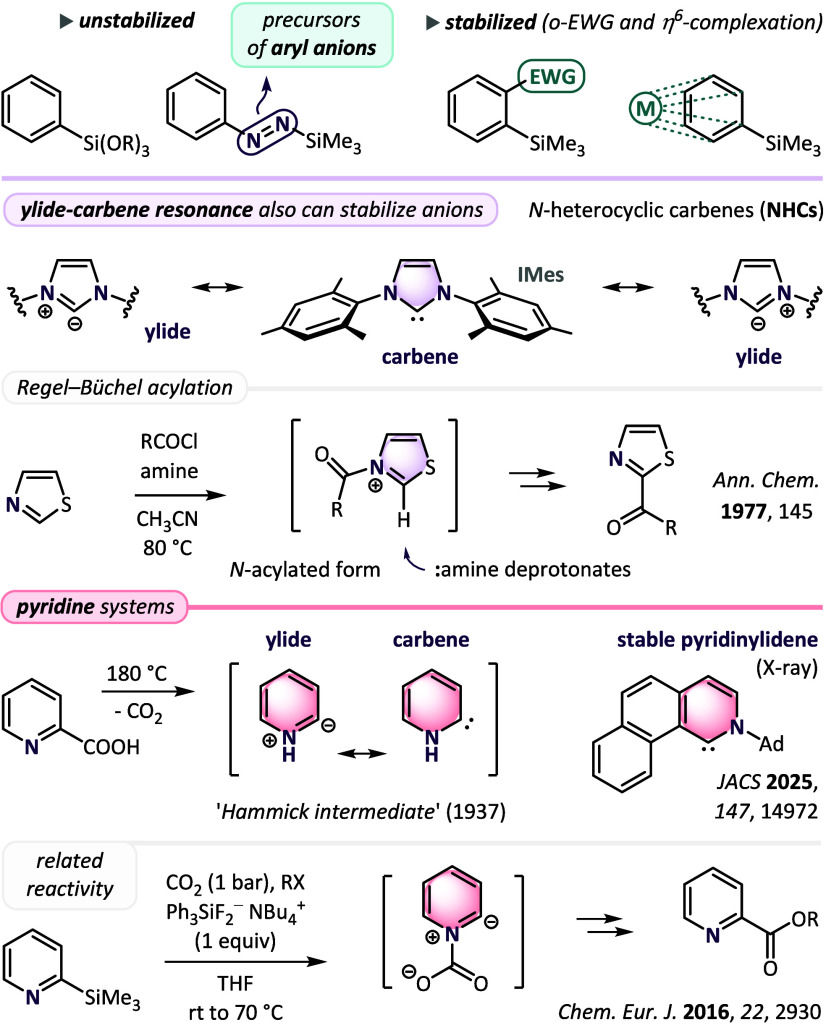
Selected Literature Data

Our project began with the exploration of a model reaction between commercially available 2-(trimethylsilyl)­pyridine (2-TMS-Py) and benzoyl chloride (PhCOCl). We observed that the uncatalyzed process runs best when equimolar mixture of the substrates is gently heated at 60 °C for 1 d, giving 2-benzoylpyridine^30a^ (**1a**), isolated in 88–91% yields (in two runs; Scheme 2, top). The same reaction tested in acetonitrile solution (*c* = ∼1 M) led to a slightly lower yield (85%), while in THF and toluene solutions the substrate conversions were not complete. The presence of substituents on the benzoyl ring was well-tolerated, and in the investigated series with 2-TMS-Py, 2-bromophenyl-2′-pyridyl ketone (**1f**) was isolated with the highest yield of 96%. Importantly, enolizable alkanoyl chlorides^32^ and tertiary dehydroabietic and 1-adamantanecarbonyl chlorides also afforded ketones **1m**–**r** (68–87%), and only the most sterically demanding 2,4,6-triisopropylbenzoyl chloride failed to react. Next, we tested substitution of the azine ring and the reactivity of bicyclic quinoline and isoquinoline substrates with PhCOCl (Scheme 2, middle). In all cases, products **2a**–**l** were formed in good to excellent yields, and only reactions with less basic Cl- and CF_3_-substituted pyridines required use of more vigorous conditions (100–120 °C).^29a^ Encouraged by these observations, we also tested preparation of ketones bearing a less common substitution pattern (**3a**–**m**). Donor- and acceptor-substituted 2-pyridyl ketones **3a**–**d**, quinoline and isoquinoline products **3e**–**h**, and Mosher’s acid (**3i**) and pyrazole (**3j**) derivatives were formed in 52–98% yields. Also, it is worth mentioning that the presence of two trimethylsilyl (TMS) groups at the 2- and 5-positions of the pyridine ring led exclusively to the reaction with the former, giving the expected product **3k** (73%). Moreover, the reaction conditions also appeared to be compatible with the fluorosulfonyl group, opening the possibility for preparing substrates for the SuFEx (sulfur fluoride exchange) click reaction.^33^ Accordingly, fluorosulfonyl-substituted ketone **3m** was synthesized at a gram-scale (62%, 1.74 g) and subjected to two orthogonal reactions with benzylamine. Under LiHMDS-promoted conditions^34^ sulfonamide **4a** was formed in excellent yield (96%), while iodine-promoted oxidative cyclization^35^ of **3m** afforded *m*-C_6_H_4_SO_2_F-substituted imidazo­[1,5-*a*]­pyridine (**4b**) in 69% yield.

**Scheme 2:**
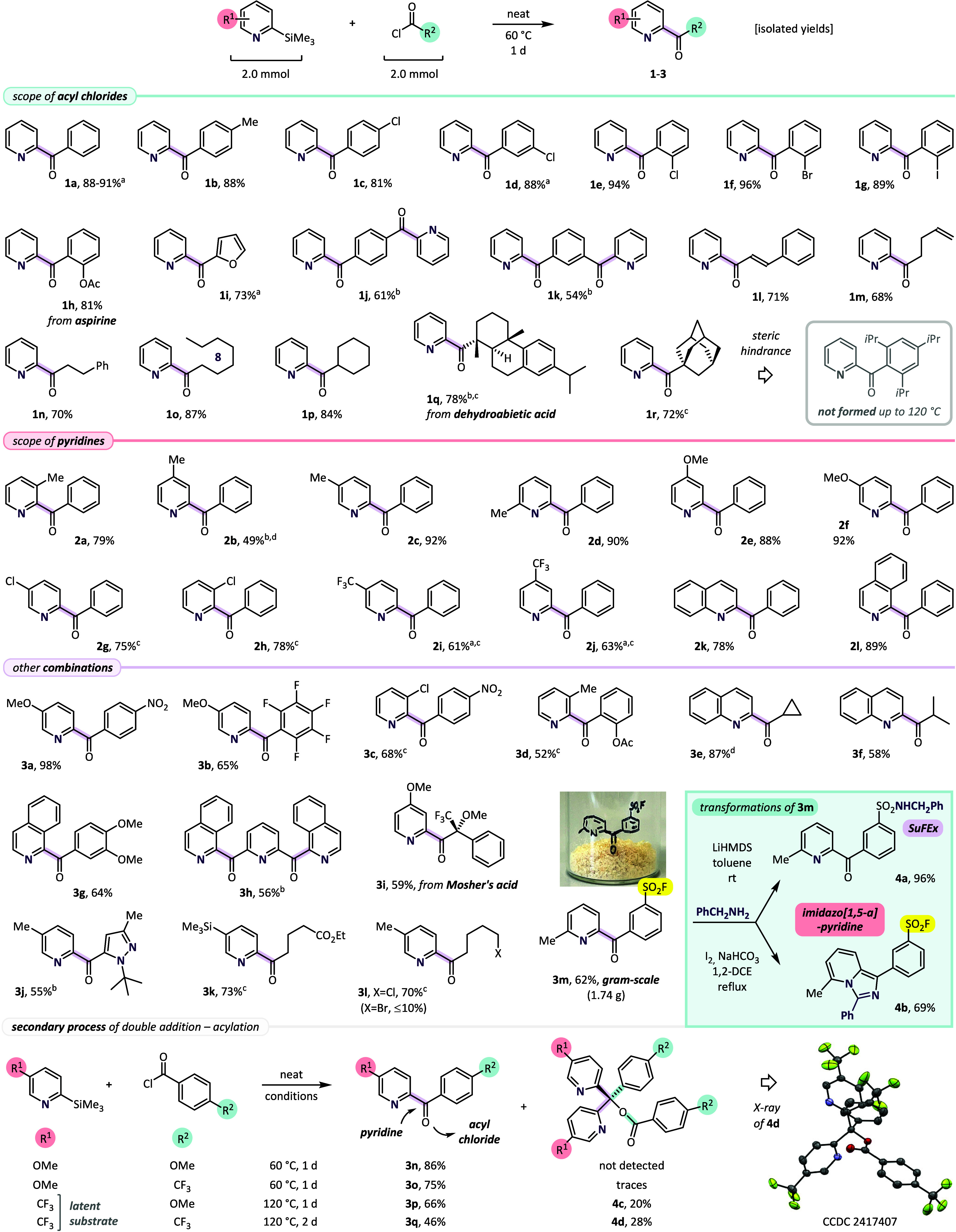
Spontaneous Acylation of 2-(Trimethylsilyl)­pyridines with Acyl Chlorides^a^

a A double addition-acylation product was isolated.
b A different reagent ratio was applied.
c A different temperature was applied.
d A different reaction time was applied. The ORTEP projection of **4d** was drawn at 50% of probability level with H atoms omitted for clarity.
e See the SI for details.

Importantly, in most of the examples from the scope and limitation studies, double pyridine addition byproducts (triarylcarbinol derivatives) were formed only in trace amounts. However, under more forcible conditions (120 °C, 1–2 d) required for latent 5-CF_3_-2-TMS-Py, ketones **3p** and **3q** (66% and 46%) were accompanied by significant amount of byproducts **4c** and **4d** (20% and 28%, respectively). The minor components displayed doubled molecular masses and contained four aromatic rings, as confirmed by X-ray studies of **4d** (Scheme 2, bottom). This behavior suggests attenuated reactivity of the nucleophilic species,^36^ which react selectively with acyl chlorides in the presence of accumulating ketone products, and only for EWG-substituted pyridines at higher temperatures can the subsequent addition followed by O-acylation of the resulting triarylcarbinol take place. The selectivity strongly contrasts from that of unstabilized phenyl anions, generated from *N*-phenyl-*N*′-(trimethylsilyl)­diazenes, which exclusively follow the double addition pathway.^16^

The successful synthetic results inspired us to perform mechanistic studies of the spontaneous acylation reaction. First, we tested use of a series of acylating agents, such as benzoyl fluoride (PhCOF),^37^ benzoyl cyanide, and methyl benzoate. All of these substrates showed low or no conversion with 2-TMS-Py, and only benzoyl bromide (PhCOBr) reacted as expected, giving **1a** in a diminished yield of 78% (Scheme 3, top). Also, changes applied to the silyl group at the pyridine ring were discouraging. Increasing the steric hindrance at the silicon atom considerably slowed the reaction with PhCOCl, and only 2-(triethylsilyl)­pyridine at elevated temperature (80 °C) formed ketone **1a** (86%), while *tert*-butyldimethylsilyl and triisopropylsilyl derivatives remained poorly reactive up to 120 °C. The model acylation reaction of 2-TMS-Py and PhCOCl was studied also in a CD_3_CN solution. Multiple NMR spectra revealed only gradual formation of **1a**, without the observation of peaks attributed to the intermediate species. In contrast, mixing 2-TMS-Py with more electrophilic PhCOBr neat at rt resulted in an immediate precipitation of solid *N*-benzoyl 2-(trimethylsilyl)­pyridinium bromide (structure assigned by NMR). Interestingly, the common acylation intermediate^38^ dissolved in CD_3_CN and heated to 60 °C also directly transformed into **1a** (Scheme 3, middle; see the SI for details). Finally, we tested reactions of isomeric 3- and 4-TMS-Py with PhCOCl under neat conditions, but in both cases the process stopped at the *N*-acylated products, and pyridyl ketones were not detected in the mixtures up to 120 °C (NMR). The result confirmed an exceptional selectivity toward the formation of 2-pyridyl ketones that supports the ylide mechanism.^29a,39^ Accordingly, significantly weaker stabilization of the corresponding *N*-acylated 3- and 4-pyridyl carbanions^27^ (betaines) hinders the desilylation step, and as a result the acylation reaction does not occur for these isomers. To integrate the findings from these mechanistic studies, reaction pathway was modeled using DFT *ab initio* calculations (Scheme 3, bottom). Initial addition of 2-TMS-Py to PhCOCl required overcoming a barrier of Δ*G* ^#^ = +21.0 kcal/mol (**TS1**) and gave *N-*acylated intermediate (**Int1**) located +2.7 kcal/mol above the level of substrates. Then, chloride anion-assisted breaking of the C–Si bond via **TS2** (Δ*G* ^#^ = +21.9 kcal/mol) formed an ylide (**Int2**) in a moderately endergonic process. Remaining steps of the *C*-acylation with second molecule of PhCOCl (**TS3**, Δ*G* ^#^ = +11.7 kcal/mol) and final removal of the benzoyl group from the nitrogen atom (**TS4**, Δ*G* ^#^ = +13.4 kcal/mol) displayed lower barriers, in agreement with lack of the intermediates observed in the NMR studies. An alternative pathway of intramolecular rearrangement of **Int2** (via **TS5**, Δ*G* ^#^ = +17.8 kcal/mol, shown in gray)^40^ and other mechanisms tested by the calculations (*e.g.*, formation of unsupported 2-pyridyl anion, direct *ipso*-substitution, etc.) appeared unlikely, due to much higher energy barriers (see the SI for details).

**Scheme 3:**
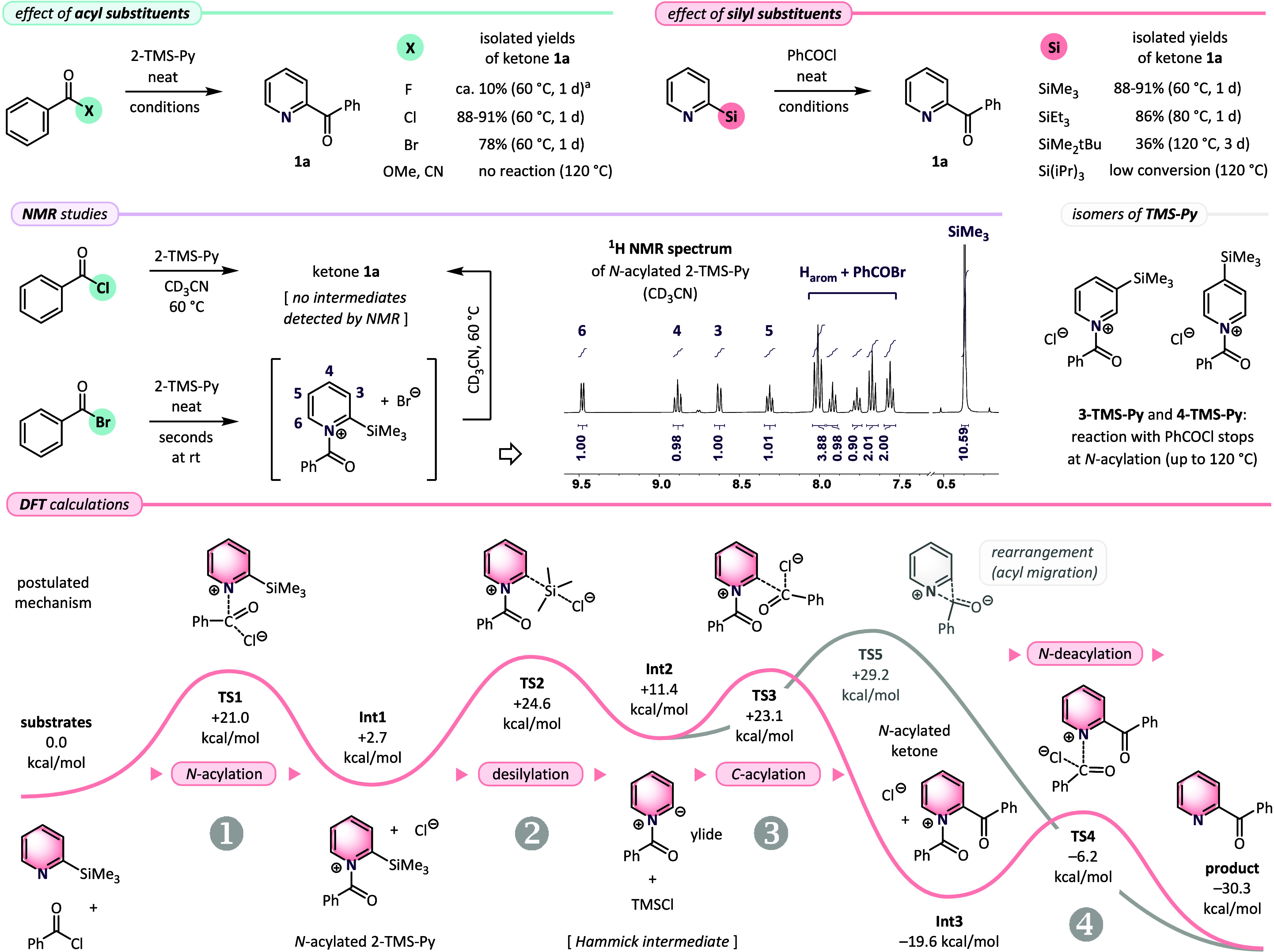
Mechanistic Studies of the Spontaneous Acylation Reaction^b^

a Conversion of PhCOF at 120 °C did not exceed 60%.
b See the SI for details. The Gibbs free energy profiles were computed at the M06-2X/6–31++g­(d,p) level of theory with SMD (pyridine) solvation model (energies were referred to the level of substrates). In NMR studies (spectrum reproduction), PhCOBr was used in a 2-fold excess.

In the last part of the project, we attempted application of the methodology for functionalization of biologically active compounds. Unfortunately, preliminary attempts at the preparation and transformation of complex acid chlorides revealed the formation of byproducts and decomposition events. Therefore, we considered use of more stable acyl fluorides^41^ in combination with fluoride anions, which form strong Si–F bonds.^42^ When 2-TMS-Py, PhCOF, and a catalytic amount of the fluoride activator (Ph_3_SiF_2_NBu_4_,^31^ 30 mol %) were combined in a DCM solution at 40 °C, ketone **1a** was isolated in an encouraging yield of 77%^43^ (Scheme 4, top). Using this protocol, 2-pyridyl ketones substituted with a chlorine atom (**1e**, 89%) and a diazo group (**1s**, 63%), derivatives of lithocholic (**1t**, 76%), (−)-camphanic (**1u**, 92%) and piperic (**1w**, 70%) acids, as well as adapalene (**1y**, 70%), probenecid (**1x**, 61%), and febuxostat (**1z**, 80%) were isolated in good yields. Although α-amino 2′-pyridyl ketones are known to be configurationally unstable,^44^ we also attempted the reaction with *N*-Cbz-*N*-methyl-leucine fluoride, which afforded ketone **1v**, isolated in 70% yield (analogous NH-Cbz substrate failed to give the corresponding product). Also, the coupling of dansyl-type 1-(triethylsilyl)­isoquinoline with arachidonic acid fluoride gave fluorescent^45^ product **3r** (69%). Functional group compatibility of the fluoride-catalyzed protocol was also demonstrated on reactions with 2- and 6-(triethylsilyl)­nicotine (Scheme 4, bottom). When model PhCOF afforded ketone **2m** in 80% yield, an analogous uncatalyzed reaction with PhCOCl tested under neat conditions led only to a complex mixture.^46^ Finally, hybrid products of the nicotine with hydrocinnamoyl fluoride (**3s**, 83%), piperic acid fluoride (**3t**, 58%), and febuxostat fluoride (**3u**, 80%, gram-scale, 1.11 g) were successfully prepared.

**Scheme 4:**
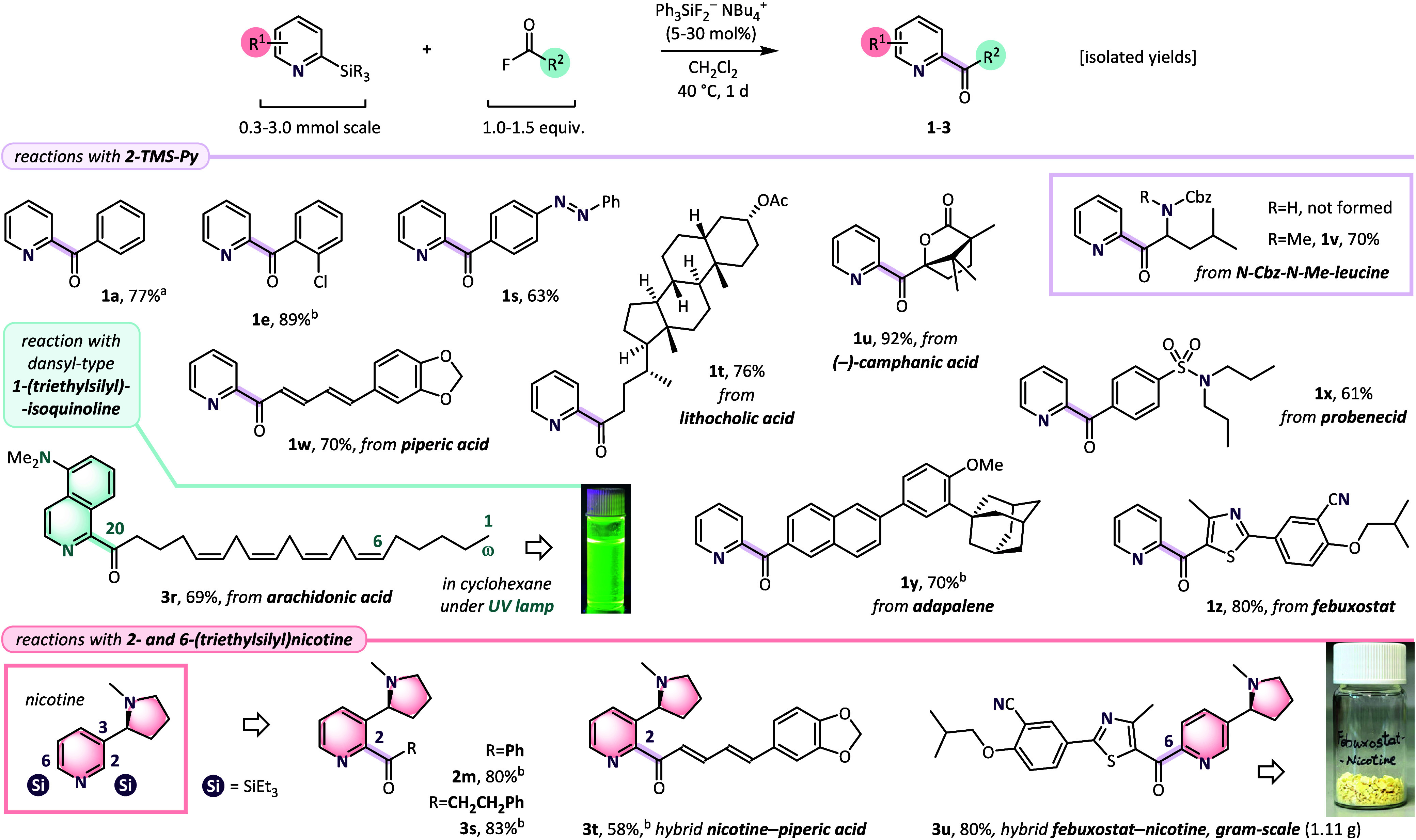
Fluoride-Catalyzed Acylation of 2-(Trialkylsilyl)­pyridines with Acyl Fluorides^a^

a A double addition-acylation product was isolated (22%).
b Different reaction conditions were applied.
c See the SI for details. Cbz = benzyloxycarbonyl group.

In conclusion, we have developed a spontaneous synthesis of 2-pyridyl ketones using acyl chlorides and easily available 2-(trialkylsilyl)­pyridines.^47^ Although this process resembles electrophilic *ipso*-substitution (the Friedel–Crafts reaction), its mechanism involves only nucleophilic species thus bypassing the typical reactivity limitations of pyridine systems. Elementary steps of *N*-acylation, desilylation, *C*-acylation, and *N*-deacylation run in succession, ensuring high selectivity for the formation of ketone products. Importantly, the initial *N*-acylation step is essential for breaking of the C–Si bond by stabilizing the released carbanion as an ylide (Hammick intermediate). We also demonstrated that the synthesis of 2-pyridyl ketones proceeds efficiently with acyl fluorides in a process catalyzed with fluoride anions. The mild conditions display good functional group tolerance that enables transformations of complex molecules.

## Supplementary Material



## Data Availability

The data underlying this study are available in the published article and its Supporting Information.

## Notes

Experimental procedures, characterization data, calculations data, and NMR spectra reproductions of the synthesized compounds (PDF)

## ∇

R&D Centre, Celon Pharma SA, Marymoncka 15, 05-152 Kazuń Nowy, Poland
