# Supplementary material for: Beyond Friedel–Crafts: Spontaneous and Fluoride-Catalyzed Acylation of 2‑(Trialkylsilyl)pyridines
Source: Org Lett. 2025 Sep 20;27(39):10954–61. doi: 10.1021/acs.orglett.5c03140 (PMC12501938; doi:10.1021/acs.orglett.5c03140)

Supporting Information

Beyond Friedel-Crafts: Spontaneous and Fluoride-Catalyzed Acylation of 2-(Trialkylsilyl)pyridines

Jan Dudziński,[1] Damian Antoniak,[1,†] Kacper Błaziak,[1,2] Michał Barbasiewicz[1,*]

[1] *University of Warsaw, Faculty of Chemistry, Pasteura 1, 02-093 Warsaw, Poland;*

[2] *University of Warsaw, Biological and Chemical Research Centre, Żwirki i Wigury 101, 02-089 Warsaw, Poland*

[†] Present Address: *Celon Pharma SA, R&D Centre, Marymoncka 15, 05-152 Kazuń Nowy, Poland*

* – barbasiewicz@chem.uw.edu.pl, www.aromaticity.pl

Table of Contents

1. General Information ... 3
2. Synthesis of substrates ... 4
2.1. Synthesis of silylated pyridines ... 4
2.2. Synthesis of acyl chlorides ... 17
2.3. Synthesis of acyl fluorides ... 19
3. Experimental procedures for the synthesis of 2-pyridyl ketones ... 24
3.1. General Procedure A: Synthesis of ketones 1a-r ... 24
3.2. General procedure B: Synthesis of ketones 2a-l ... 33
3.3. General procedure C: Synthesis of ketones 3a-q ... 39
3.4. General procedure D: Fluoride-catalyzed synthesis of ketones ... 48
3.5. Orthogonal transformations of 3m: Synthesis of 4a,b ... 56
4. Mechanistic studies ... 58
4.1. Effect of acyl and silyl substituents ... 58
4.2. NMR studies of the reaction course ... 59
4.3. Reactivity of isomeric TMS-pyridines ... 66
4.4. DFT calculations data ... 68
5. Single-crystal XRD analysis data for 4d (CCDC 2417407) ... 92
6. NMR spectra reproductions ... 94

1. General Information

Commercially available solvents and materials were used without further purification. Melting points were uncorrected. Analytical gas-liquid chromatography (GLC) was performed with a PerkinElmer Clarus 580 chromatograph equipped with a flame ionization detector, and a GL Sciences InertCap 5MS/Sil column with helium as a carrier gas (column 0.25 mm×30 m, carrier flow 1.5 mL/min, method parameters 50 °C, +10 °C/min to 300 °C, then 15 min at 300 °C). Thin-layer chromatography (TLC) was performed on Supelco silica gel on TLC Al foil with fluorescent indicator 254 nm and was visualized under UV lamp. Column chromatography was performed on silica gel (SiliaFlash® P60, 230-400 mesh particle size, 40-63 µm). ^{1}H, ^{19}F, and ^{13}C NMR spectra were recorded with an Agilent 400 MHz NMR spectrometer. Chemical shifts (δ) are given in parts per million (ppm) with solvent resonance as the internal standard (for $CDCl_3$: 7.26 and 77.16 ppm, for CD_3CN: 1.94 and 118.26 ppm) or with $CFCl_3$ in $CDCl_3$ (0.0 ppm for ^{19}F NMR). Standard abbreviations are used to denote spin multiplicity.

2. Synthesis of substrates

2.1. Synthesis of silylated pyridines

2-(Trimethylsilyl)pyridine is commercially available and it was used as received. Other silylated substrates were synthesized as described:

Synthesis of 2-(trimethylsilyl)azines listed below:

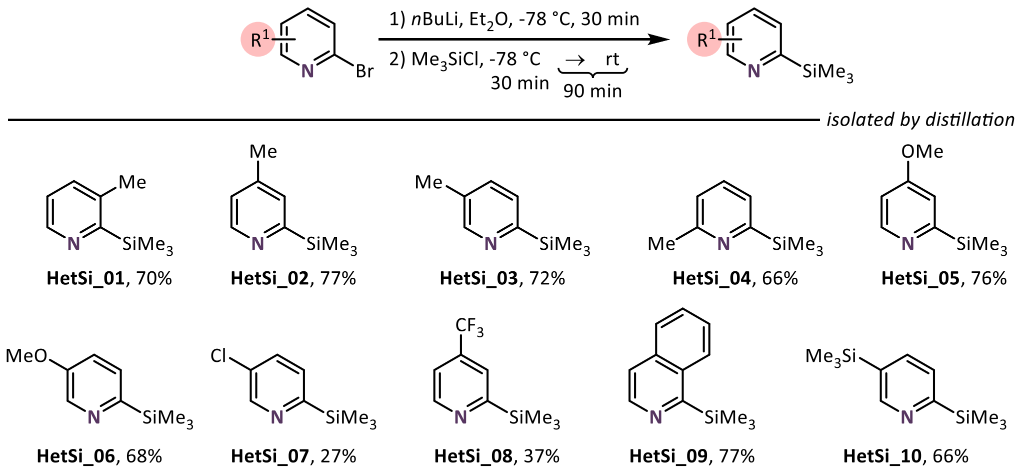

A 100 mL Schlenk flask was charged with the corresponding 2-bromopyridine (15-50 mmol scale) and flushed with argon. Anhydrous Et_2O (40 mL, regardless of scale) was added. Resulting mixture was placed in an acetone bath (–78 °C). Then, *n*-butyllithium solution (1.1 equiv., 2.5 M in hexanes) was added dropwise over 5-10 min. After 30 min, Me_3SiCl (1.2 equiv.) was added dropwise (1-2 min). After next 30 min the Schlenk flask was taken out of the bath and allowed to warm to room temperature. After 90 min of stirring at room temperature, the mixture was quenched with an aqueous solution of $NaHCO_3$ (20 mL, 5%). Then, the mixture was transferred into separatory funnel, where next portion of an aqueous solution of $NaHCO_3$ (80 mL, 5%) was added, and the mixture was extracted with ethyl acetate (3×100 mL). Combined organic layers were washed with water (100 mL), brine (100 mL), and dried over anhydrous $MgSO_4$. Then, the mixture was filtered, evaporated, and product was isolated by distillation under reduced pressure.

Note: Solubility of bromopyridines in Et_2O at –78 °C is poor and most of them formed suspensions, which clarified during the reaction.

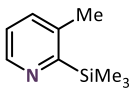

HetSi_01

Yield: 70% (3.299 g, 19.96 mmol, 28.33 mmol scale), colorless oil,
bp: 88–89 °C (p=20 mbar)

^{1}H NMR (400 MHz, $CDCl_3$): δ = 8.57 (ddd, *J*=4.8, 1.6, 0.7 Hz, 1H), 7.37–7.29 (m, 1H), 7.08 (dd, *J*=7.8, 4.7 Hz, 1H), 2.41 (s, 3H), 0.37 (s, 9H).

^{13}C NMR (100 MHz, $CDCl_3$): δ = 166.4, 147.0, 139.2, 135.8, 122.6, 20.7, –0.4.

NMR spectra were consistent with those reported in the literature: *Chem. Eur. J.* **2016**, *22*, 2930.

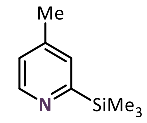

HetSi_02

Yield: 77% (3.657 g, 22.12 mmol, 28.85 mmol scale), colorless oil,
bp: 81–83 °C (p=20 mbar).

^{1}H NMR (400 MHz, $CDCl_3$): δ = 8.59 (dd, *J*=5.0, 0.9 Hz, 1H), 7.31–7.26 (m, 1H), 6.96 (ddd, *J*=5.0, 1.9, 0.8 Hz, 1H), 2.27 (s, 3H), 0.28 (s, 9H).

^{13}C NMR (100 MHz, $CDCl_3$): δ = 167.7, 150.0, 144.7, 129.9, 123.7, 21.1, –1.7.

NMR spectra were consistent with those reported in the literature: *Chem. Eur. J.* **2016**, *22*, 2930.

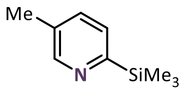

HetSi_03

Yield: 72% (2.909 g, 17.60 mmol, 24.50 mmol scale), colorless oil,
bp: 79–80 °C (p=15 mbar)

^{1}H NMR (400 MHz, $CDCl_3$): δ = 8.65–8.59 (m, 1H), 7.42–7.37 (m, 2H), 2.31 (s, 3H), 0.30 (s, 9H).

^{13}C NMR (100 MHz, $CDCl_3$): δ = 164.5, 150.9, 134.5, 132.1, 128.3, 18.6, –1.7.

NMR spectra were consistent with those reported in the literature: *Chem. Eur. J.* **2016**, *22*, 2930.

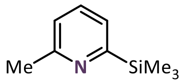

HetSi_04

Yield: 66% (3.269 g, 19.78 mmol, 29.98 mmol scale), colorless oil,
bp: 65–66 °C (p=15 mbar)

^{1}H NMR (400 MHz, $CDCl_3$): δ = 7.48–7.43 (m, 1H), 7.31–7.28 (m, 1H), 7.05–7.02 (m, 1H), 2.58 (s, 3H), 0.31 (s, 9H).

^{13}C NMR (100 MHz, $CDCl_3$): δ = 167.7, 158.4, 134.0, 125.8, 122.4, 25.1, –1.6.

NMR spectra were consistent with those reported in the literature: *Chem. Eur. J.* **2016**, *22*, 2930.

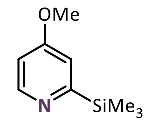

HetSi_05

Yield: 76% (3.474 g, 19.16 mmol, 25.23 mmol scale), colorless oil,
bp: 90–92 °C (p=10 mbar)

^{1}H NMR (400 MHz, $CDCl_3$): δ = 8.55 (dd, *J*=5.8, 0.6 Hz, 1H), 7.00 (dd, *J*=2.8, 0.6 Hz, 1H), 6.66 (dd, *J*=5.7, 2.8 Hz, 1H), 3.77 (s, 3H), 0.27 (s, 9H).

^{13}C NMR (100 MHz, $CDCl_3$): δ = 169.6, 164.2, 151.5, 115.5, 108.2, 54.8, –1.8.

^{1}H NMR spectrum was partially consistent with that reported in the literature: *Chem. Ber.* **1992**, *125*, 1131.

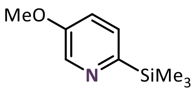

HetSi_06

Yield: 68% (3.193 g, 17.61 mmol, 26.09 mmol scale), colorless oil,
bp: 87–88 °C (p=7 mbar)

^{1}H NMR (400 MHz, CDCl$_3$): δ = 8.52 (dd, *J*=2.9, 0.7 Hz, 1H), 7.43 (dd, *J*=8.4, 0.7 Hz, 1H), 7.10 (ddd, *J*=8.3, 3.0, 0.6 Hz, 1H), 3.85 (s, 3H), 0.30 (s, 9H).

^{13}C NMR (100 MHz, CDCl$_3$): δ = 158.8, 155.1, 138.9, 129.1, 118.1, 55.3, –1.5.

^{1}H NMR spectrum was consistent with that reported in the literature: *Chem. Ber.* **1992**, *125*, 1131.

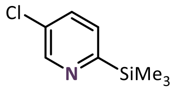

HetSi_07

Yield: 27% (2.654 g, 14.29 mmol, 52.62 mmol scale) colorless oil,
bp: 72–74 °C (p=10 mbar)

^{1}H NMR (400 MHz, CDCl$_3$): δ = 8.70 (dd, *J*=2.4, 0.9 Hz, 1H), 7.52 (dd, *J*=8.1, 2.4 Hz, 1H), 7.40 (dd, *J*=8.1, 0.8 Hz, 1H), 0.28 (s, 9H).

^{13}C NMR (100 MHz, CDCl$_3$): δ = 166.3, 149.2, 133.7, 131.8, 129.3, –1.8.

^{1}H NMR spectrum was partially consistent with that reported in the literature: *Chem. Ber.* **1992**, *125*, 1131.

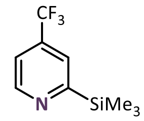

HetSi_08

Yield: 37% (1.807 g, 8.24 mmol, 22.06 mmol scale), colorless oil,
bp: 70-90 °C (temperature of the oil bath; p=35 mbar)

^{1}H NMR (400 MHz, CDCl$_3$): δ = 8.93 (d, *J*=5.0 Hz, 1H), 7.68–7.63 (m, 1H), 7.37 (ddd, *J*=5.2, 1.9, 0.8 Hz, 1H), 0.32 (s, 9H).

^{13}C NMR (100 MHz, CDCl$_3$): δ = 170.7, 150.6, 136.5 (q, *J*=33.6 Hz), 123.7 (q, *J*=3.4 Hz), 123.3 (q, *J*=273 Hz), 118.2 (q, *J*=3.6 Hz), –2.0.

^{19}F NMR (376 MHz, CDCl$_3$): δ = –65.4.

^{1}H NMR spectrum was consistent and ^{13}C NMR spectrum was partially consistent with those reported in the literature: *Chem. Eur. J.* **2016**, *22*, 2930.

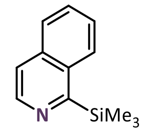

HetSi_09

Yield: 77% (3.553 g, 17.65 mmol, 22.84 mmol scale), colorless oil,
bp: 87–88 °C (p=5.3×10^{-2} mbar)

^{1}H NMR (400 MHz, CDCl$_3$): δ = 8.69 (d, *J*=5.7 Hz, 1H), 8.26–8.23 (m, 1H), 7.84 –7.80 (m, 1H), 7.65 (ddd, *J*=8.2, 6.8, 1.3 Hz, 1H), 7.61–7.56 (m, 2H), 0.53 (s, 9H).

^{13}C NMR (100 MHz, $CDCl_3$): δ = 171.6, 142.9, 134.5, 133.0, 129.4, 127.85, 127.78, 126.6, 120.2, 0.2.

MS (EI) m/z: (%) 201 (61, [M^+]), 200 (100), 186 (62), 159 (13), 130 (9), 72 (8).

HRMS (EI) m/z: calcd. for $C_{12}H_{15}NSi$ [M^+] 201.0974; found 201.0971.

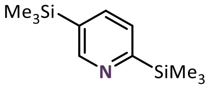

HetSi_10

Synthesized using **2.5 equiv.** of ***n*BuLi** and **3.0 equiv**. of **Me_3SiCl**, the product was isolated by **crystallization** from *n*-heptane

Yield: 66% (16.61 mmol, 25.00 mmol scale), pale brown solid,
mp: 83.5–85 °C (Lit. 82–84 °C: *Helv. Chim. Acta* **1972**, *55*, 289)

^{1}H NMR (400 MHz, $CDCl_3$): δ = 8.85 (s, 1H), 7.67 (dd, *J*=7.4, 1.8 Hz, 1H), 7.46 (dd, *J*=7.4, 1.1 Hz, 1H), 0.30 (s, 9H), 0.27 (s, 9H).

^{13}C NMR (100 MHz, $CDCl_3$): δ = 168.4, 154.1, 139.1, 133.8, 128.2, –1.3, –1.8.

^{1}H NMR spectrum was consistent with that reported in the literature: *Helv. Chim. Acta* **1972**, *55*, 289.

Synthesis of 5-(trifluoromethyl)-2-(trimethylsilyl)pyridine

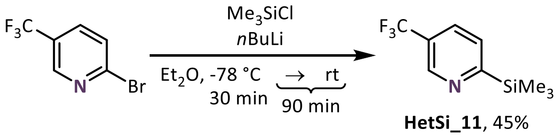

HetSi_11, 45%

A 100 mL Schlenk flask was charged with 2-bromo-5-trifluoromethylpyridine (4.973 g, 22.01 mmol) and flushed with argon. Anhydrous Et_2O (40 mL) was added. Resulting mixture was placed in an acetone bath (–78 °C), where Me_3SiCl (11.2 mL, 88.3 mmol, 4.0 equiv.) was added. Then, *n*-butyllithium solution (9.7 mL, 2.5 M in hexanes, 24.3 mmol, 1.1 equiv.) was added dropwise over ca. 5 min. After 30 min, the Schlenk flask was taken out of the bath and allowed to warm to room temperature. After next 90 min of stirring at room temperature, the mixture was poured onto a vigorously stirred aqueous solution of $NaHCO_3$ (100 mL, 5%). Then, the mixture was transferred into separatory funnel and extracted with ethyl acetate (3×100 mL). Combined organic layers were washed with water (100 mL), brine (100 mL), and dried over anhydrous $MgSO_4$. The mixture was filtered, evaporated, and the product was separated by distillation under reduced pressure (p=30 mbar; T=75-76 °C), to afford 5-(trifluoromethyl)-2-(trimethylsilyl)pyridine (2.159 g, 9.85 mmol, 45%), as a colorless oil.

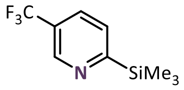

HetSi_11

Yield: 45% (2.159 g, 9.85 mmol, 22.01 mmol scale), colorless oil,
bp: 75–76 °C (p=30 mbar)

^{1}H NMR (400 MHz, $CDCl_3$): δ = 9.00 (s, 1H), 7.78 (dd, *J*=7.9, 2.3 Hz, 1H), 7.61 (d, *J*=7.9 Hz, 1H), 0.33 (s, 9H).

^{13}C NMR (100 MHz, $CDCl_3$): δ = 173.7, 146.5 (q, *J*=4.0 Hz), 130.8 (q, *J*=3.6 Hz), 128.1, 125.4 (q, *J*=32.7 Hz), 123.9 (q, *J*=272 Hz), –1.9.

^{19}F NMR (376 MHz, $CDCl_3$): δ = –63.2.

^{1}H NMR spectrum was consistent and ^{13}C NMR spectrum was partially consistent with those reported in the literature: *Chem. Eur. J.* **2016**, *22*, 2930.

Synthesis of 3-chloro-2-(trimethylsilyl)pyridine

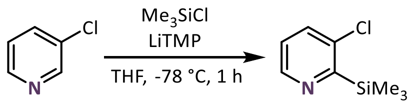

HetSi_12, 43%

Generation of LiTMP: A 100 mL Schlenk flask was flushed with argon and charged with 2,2,6,6-tetramethylpiperidine (6.7 mL, 39.4 mmol, 1.3 equiv.). Anhydrous THF (20 mL) was added. Resulting solution was placed in an ice-water bath and then, n-butyllithium solution (14.4 mL, 2.5 M in hexanes, 36.0 mmol, 1.2 equiv.) was added dropwise. After 30 min of stirring in the ice-water bath, the solution was transferred into reaction flask using syringe.

A 100 mL Schlenk flask was charged with 3-chloropyridine (3.411 g, 30.04 mmol) and flushed with argon. Anhydrous THF (20 mL) was added. Resulting mixture was placed in an acetone bath (–78 °C), where Me_3SiCl (9.2 mL, 72.5 mmol, 2.4 equiv.) was added. Then, a previously prepared LiTMP solution was added dropwise over ca. 5 min. After 1 h, the Schlenk flask was taken out of the bath and the mixture was poured onto vigorously stirred H_2O (100 mL). Then, the mixture was transferred into separatory funnel and extracted with ethyl acetate (3×100 mL). Combined organic layers were washed with water (100 mL), brine (100 mL), and dried over anhydrous $MgSO_4$. The mixture was filtered, evaporated, and the product was separated by distillation under reduced pressure (p=25 mbar; T = 99–101 °C), to afford 3-chloro-2-(trimethylsilyl)pyridine (2.414 g, 13.00 mmol, 43%), as pale yellow oil.

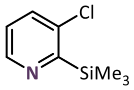

HetSi_12

Yield: 43% (2.414 g, 13.00 mmol, 30.04 mmol scale), pale yellow oil,
bp: 99–101 °C (p=25 mbar)

^{1}H NMR (400 MHz, $CDCl_3$): δ = 8.66–8.59 (m, 1H), 7.56–7.51 (m, 1H), 7.13 (ddd, *J*=8.1, 4.6, 0.6 Hz, 1H), 0.41 (s, 9H).

^{13}C NMR (100 MHz, $CDCl_3$): δ = 165.6, 147.6, 139.5, 135.0, 123.7, –1.2.

^{1}H NMR spectrum was partially consistent with that reported in the literature: *J. Organomet. Chem.* **1981**, *216*, 139.

Synthesis of 2-(trimethylsilyl)quinoline

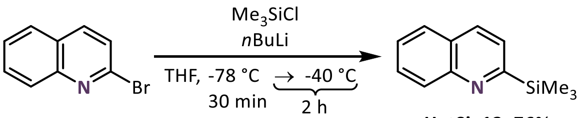

HetSi_13, 76%

A 100 mL Schlenk flask was charged with 2-bromoquinoline (4.161 g, 20.00 mmol) and flushed with argon. Anhydrous THF (40 mL) was added. Resulting mixture was placed in an acetone bath (–78 °C), where Me_3SiCl (5.1 mL, 40.2 mmol, 2.0 equiv.) was added. Then, *n*-butyllithium solution (12.0 mL, 2.5 M in hexanes, 30.0 mmol, 1.5 equiv.) was added dropwise over ca. 5 min. After 30 min, the Schlenk flask allowed to warm to –40 °C (reached in ca. 1 h). After 1 h of additional stirring at –40 °C, the mixture was poured onto a vigorously stirred aqueous solution of $NaHCO_3$ (100 mL, 5%). Then, the mixture was transferred into separatory funnel and extracted with ethyl acetate (3×100 mL). Combined organic layers were washed with water (100 mL), brine (100 mL), and dried over anhydrous $MgSO_4$. The mixture was filtered, evaporated, and the product was separated by distillation under reduced pressure (p=3.2×10^{-2} mbar; T=80-81 °C), to afford 2-(trimethylsilyl)quinoline (3.071 g, 15.25 mmol, 76%), as a colorless oil.

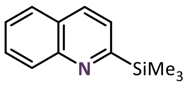

HetSi_13

Yield: 76% (3.071 g, 15.25 mmol, 20.00 mmol scale), colorless oil,
bp: 80-81 °C (p=3.2×10^{-2} mbar)

^{1}H NMR (400 MHz, $CDCl_3$): δ = 8.26 (dd, *J*=8.5, 1.0 Hz, 1H), 8.04 (dd, *J*=8.2, 0.9 Hz, 1H), 7.78 (dd, *J*=8.1, 1.5 Hz, 1H), 7.72 (ddd, *J*=8.4, 6.9, 1.5 Hz, 1H), 7.63 (d, *J*=8.2 Hz, 1H), 7.52 (ddd, *J*=8.1, 6.9, 1.2 Hz, 1H), 0.49 (s, 9H).

^{13}C NMR (100 MHz, $CDCl_3$): δ = 170.6, 149.0, 133.3, 130.1, 129.0, 127.8, 127.4, 126.4, 125.1, –1.6.

^{1}H NMR spectrum was consistent with that reported in the literature: *J. Org. Chem.* **2020**, *85*, 14420.

Synthesis of 2-(triethylsilyl)nicotine

2-(Triethylsilyl)nicotine was synthesized following the procedure reported in the literature (*Org. Lett.* **2005**, *7*, 5457):

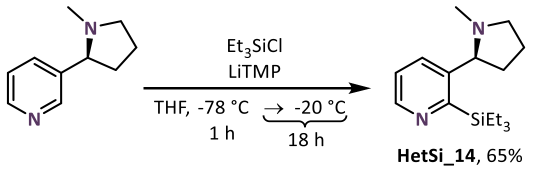

HetSi_14, 65%

A 250 mL Schlenk flask was flushed with argon and charged with 2,2,6,6-tetramethylpiperidine (5.6 mL, 32.9 mmol, 1.3 equiv.). Anhydrous THF (30 mL) was added. Resulting mixture was placed in an ice-water bath, where *n*-butyllithium solution (12.0 mL, 2.5 M in hexanes, 30.0 mmol, 1.2 equiv.) was added dropwise over ca. 5 min. The mixture was stirred at 0 °C for additional 15 min and then it was placed in an acetone bath (–78 °C), where Et_3SiCl (10.1 mL, 60.2 mmol, 2.4 equiv.) was added. Then, previously

cooled to 0 °C *L*-nicotine (4.091 g, 25.22 mmol) solution in anhydrous THF (20 mL) was added over ca. 30 s. After 1 h of stirring at –78 °C the mixture was placed in the freezer (ca. –20 °C). After 18 h, the mixture was taken out of the freezer and it was poured onto a vigorously stirred aqueous solution of $NaHCO_3$ (100 mL, 5%). Then, the mixture was transferred into separatory funnel and extracted with ethyl acetate (3×100 mL). Combined organic layers were washed with water (100 mL), brine (100 mL), and dried over anhydrous $MgSO_4$. Then, the mixture was filtered, evaporated, and the product was separated by column chromatography (eluent: 1st column: cyclohexane/ethyl acetate 10:1 to 1:1 (+ 1% NEt_3, v/v), 2nd column: DCM, then DCM/ethyl acetate 6:1 to 1:1 (+ 1% NEt_3, v/v)) to afford 2-(triethylsilyl)Nicotine (4.553 g, 16.47 mmol, 65%), as pale yellow oil.

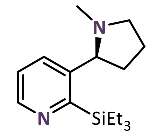

HetSi_14

Yield: 65% (4.553 g, 16.47 mmol, 25.22 mmol scale), a pale yellow oil

Eluent: 1st column: cyclohexane/ethyl acetate 10:1 to 1:1 (+ 1% NEt_3, v/v), 2nd column: DCM, then DCM/ethyl acetate 6:1 to 1:1 (+ 1% NEt_3, v/v)

^{1}H NMR (400 MHz, $CDCl_3$): δ = 8.59 (dd, *J*=4.6, 1.7 Hz, 1H), 7.82 (dd, *J*=8.1, 1.8 Hz, 1H), 7.14 (dd, *J*=8.1, 4.6 Hz, 1H), 3.38–3.29 (m, 1H), 3.24–3.15 (m, 1H), 2.34–2.23 (m, 1H), 2.19–2.12 (m, 1H), 2.10 (s, 3H), 1.98–1.84 (m, 1H), 1.83–1.69 (m, 1H), 1.62–1.48 (m, 1H), 0.97–0.85 (m, 15H).

^{13}C NMR (100 MHz, $CDCl_3$): δ = 163.9, 148.4, 146.5, 132.9, 123.1, 67.6, 56.9, 40.2, 36.3, 22.9, 7.6, 4.8.

MS (EI) m/z: (%) 276 (64, [M^+]), 247 (100), 231 (59), 219 (35), 84 (41), 60 (18).

HRMS (EI) m/z: calc. for $C_{16}H_{28}N_2Si$ 276.2022 [M^+]; found 276.2033.

Note: During the reaction also minor 6-isomer is formed. For its preparation, see the following section.

Synthesis of 6-(triethylsilyl)nicotine

6-(Triethylsilyl)nicotine was synthesized following the procedure reported in the literature (*Org. Lett.* **2005**, *7*, 5457):

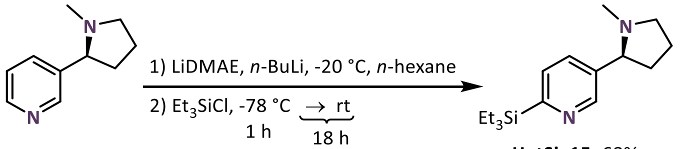

HetSi_15, 68%

A 250 mL Schlenk flask was flushed with argon and charged with *N*,*N*-dimethylaminoethanol (6.0 mL, 59.6 mmol, 2.9 equiv.). Anhydrous *n*-hexane (40 mL) was added. Resulting mixture was placed in an ice-water bath, where *n*-butyllithium solution (43.2 mL, 2.5 M in hexanes, 108 mmol, 5.3 equiv.) was added dropwise over ca. 10 min. The mixture was stirred at 0 °C for additional 20 min and then it was placed in an acetone bath (–20 °C), where *L*-nicotine (3.303 g, 20.36 mmol) was added dropwise over ca. 2 min. After 1 h, the flask was cooled to –78 °C and Et_3SiCl (13.5 mL, 80.4 mmol, 4.0 equiv.) was added dropwise over ca. 2 min. After 1 h of stirring at –78 °C the cooling bath was allowed to warm to room temperature.

After 18 h, the mixture was taken out of the bath and it was poured onto a vigorously stirred aqueous solution of $NaHCO_3$ (100 mL, 5%). Then, the mixture was transferred into separatory funnel and it was extracted with ethyl acetate (3×100 mL). Combined organic layers were washed with water (100 mL), brine (100 mL), and dried over anhydrous $MgSO_4$. The mixture was filtered, evaporated, and the product was separated by column chromatography (eluent: cyclohexane/ethyl acetate 10:1 to 2:1 (+ 1% NEt_3, v/v)) to afford 6-(triethylsilyl)Nicotine (3.823 g, 13.83 mmol, 68%), as a yellowish oil.

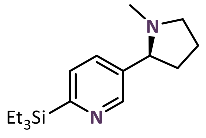

HetSi_15

Yield: 68% (3.823 g, 13.83 mmol, 20.36 mmol scale), a yellowish oil

Eluent: cyclohexane/ethyl acetate 10:1 to 2:1 (+ 1% NEt_3, v/v)

^{1}H NMR (400 MHz, $CDCl_3$): δ = 8.58 (dd, *J*=2.3, 0.9 Hz, 1H), 7.47 (dd, *J*=7.7, 2.2 Hz, 1H), 7.31 (dd, *J*=7.8, 1.0 Hz, 1H), 3.15–3.06 (m, 1H), 2.93 (t, *J*=8.2 Hz, 1H), 2.22–2.11 (m, 1H), 2.11–2.01 (m, 4H), 1.89–1.75 (m, 1H), 1.73–1.55 (m, 2H), 0.91–0.82 (m, 9H), 0.78–0.69 (m, 6H).

^{13}C NMR (100 MHz, $CDCl_3$): δ = 164.7, 150.2, 137.3, 132.2, 129.7, 68.9, 56.9, 40.3, 35.0, 22.6, 7.3, 3.0.

HRMS (ESI) m/z calcd. for $C_{16}H_{28}N_2Si$ 277.2100 [M+H$^+$]; found 277.2103

Note: During the reaction also minor 2-isomer is formed. For its preparation, see the previous section.

Synthesis of 1-(triethylsilyl)-5-(dimethylamino)isoquinoline

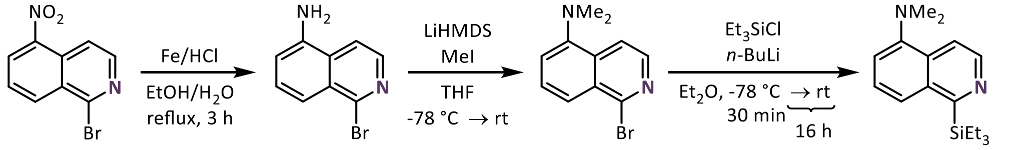

HetSi_16
45% over 3 steps

A 250 mL round-bottom flask was charged with 1-bromo-5-nitroisoquinoline (2.534 g, 10.01 mmol). Then EtOH (50 mL, 95%), water (10 mL), iron powder (2.791 g, 49.98 mmol, 5.0 equiv.) and HCl (1.9 mL, 35-38%, ca. 22 mmol, ca. 2.2 equiv.) were added and the mixture was refluxed (mantle T=100 °C) for 3 h. Then, the mixture was cooled to room temperature, filtered and concentrated. A saturated aqueous solution of $NaHCO_3$ (100 mL) was added and the mixture was extracted with DCM (3×100 mL). Combined organic layers were washed with water (100 mL), brine (100 mL) and dried over anhydrous Na_2SO_4. The mixture was filtered, evaporated and dried under vacuum, to afford crude 1-bromo-5-amino-isoquinoline (1.989 g, 8.92 mmol).

The crude 1-bromo-5-amino-isoquinoline was placed in a 250 mL round-bottom flask and flushed with argon. Anhydrous THF (40 mL) was added and the resulting suspension was placed in an acetone bath (–78 °C). A LiHMDS solution (18.8 mL, 1.0 M in THF, 18.8 mmol, 2.1 equiv.) was added dropwise over ca. 5 min. After next 30 min, MeI (1.25 mL, 20.08 mmol, 2.25 equiv.) was added and the flask was taken out of the bath. After next 2 h at room temperature, the mixture was poured onto an aqueous solution of $NaHCO_3$ (100 mL, 5%) and extracted with ethyl acetate (3×100 mL). Combined organic layers were

washed with water (100 mL), brine (100 mL) and dried over anhydrous Na_2SO_4. The mixture was filtered, evaporated and dried under vacuum, to afford crude 1-bromo-5-(dimethylamino)isoquinoline (2.236 g, 8.90 mmol).

The crude 1-bromo-5-(dimethylamino)isoquinoline was placed in a 250 mL round-bottom flask and flushed with argon. Anhydrous Et_2O (20 mL) and Et_3SiCl (1.8 mL, 10.7 mmol, 1.2 equiv.) were added, and the flask was placed in an acetone bath (–78 °C). Then, *n*-butyllithium solution (3.9 mL, 2.5 M in hexanes) was added dropwise over ca. 5 min. After 30 min, the flask was taken out of the bath and after 16 h of stirring at room temperature, the mixture was poured onto an aqueous solution of $NaHCO_3$ (100 mL, 5%) and it was extracted with ethyl acetate (3×100 mL). Combined organic layers were washed with water (100 mL), brine (100 mL) and dried over anhydrous $MgSO_4$. The mixture was filtered, concentrated and separated with column chromatography (eluent: cyclohexane, then cyclohexane : ethyl acetate 20:1 to 18:1 (+1 % Et_3N v/v)) to afford 1-(triethylsilyl)-5-(dimethylamino)isoquinoline (1.298 g, 4.53 mmol, 45% over 3 steps), as a pale brown oil.

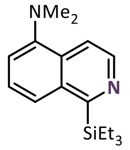

HetSi_16

Yield: 45% (1.298 g, 4.53 mmol, 10.01 mmol scale), a pale brown oil

Eluent: cyclohexane, then cyclohexane/ethyl acetate 20:1 to 18:1 (+ 1% NEt_3, v/v)

^{1}H NMR (400 MHz, $CDCl_3$): δ = 8.70 (d, *J*=5.9 Hz, 1H), 7.92 (d, *J*=5.9 Hz, 1H), 7.87 (d, *J*=8.4 Hz, 1H), 7.47 (dd, *J*=8.4, 7.5 Hz, 1H), 7.19 (d, *J*=7.5 Hz, 1H), 2.89 (s, 6H), 1.14–1.03 (m, 6H), 1.04–0.97 (m, 9H).

^{13}C NMR (100 MHz, $CDCl_3$): δ = 170.1, 150.7, 142.5, 135.1, 130.2, 126.5, 122.0, 116.8, 116.3, 45.1, 7.8, 4.9.

MS (EI) m/z: (%) 286 (100, [M^+]), 271 (25), 257 (82), 230 (45), 201 (46), 100 (31).

HRMS (EI) m/z: calcd. for $C_{17}H_{26}N_2Si$ 286.1865 [M^+]; found 286.1867.

Synthesis of 2-(triethylsilyl)pyridine

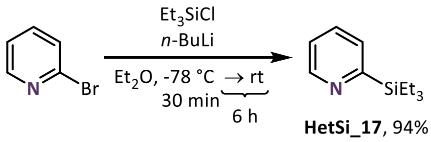

HetSi_17, 94%

A 100 mL Schlenk flask was charged with 2-bromopyridine (1.581 g, 10.00 mmol) and flushed with argon. Anhydrous Et_2O (20 mL) was added. Resulting solution was placed in an acetone bath (–78 °C), where Et_3SiCl (1.85 mL, 11.02 mmol, 1.1 equiv.) was added, and *n*-butyllithium solution (4.4 mL, 2.5 M in hexanes, 11 mmol, 1.1 equiv.) was added dropwise over ca. 2 min. After 30 min, the Schlenk flask was taken out of the bath and it was allowed to warm to room temperature. After 6 h of stirring at room temperature, the mixture was quenched with an aqueous solution of $NaHCO_3$ (20 mL, 5%). Then, the mixture was transferred into separatory funnel, another portion of an aqueous solution of $NaHCO_3$ (80

mL, 5%) was added, and it was extracted with ethyl acetate (3×100 mL). Combined organic layers were washed with water (100 mL), brine (100 mL), and dried over anhydrous $MgSO_4$. The mixture was filtered, evaporated, and the product was separated by column chromatography, to afford 2-(triethylsilyl)pyridine (1.812 g, 9.37 mmol, 94%), as a yellow oil.

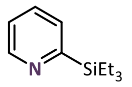

HetSi_17

Yield: 94% (1.812 g, 9.37 mmol, 10.00 mmol scale), a yellow oil

Eluent: cyclohexane, then cyclohexane/ethyl acetate 20:1 to 10:1 (+ 1% NEt_3, v/v)

^{1}H NMR (400 MHz, $CDCl_3$): δ = 8.74–8.69 (m, 1H), 7.52–7.45 (m, 1H), 7.42–7.37 (m, 1H), 7.12–7.06 (m, 1H), 0.96–0.88 (m, 9H), 0.85–0.77 (m, 6H).

^{13}C NMR (100 MHz, $CDCl_3$): δ = 166.3, 150.1, 133.7, 129.9, 122.6, 7.3, 2.9.

NMR spectra were consistent with those reported in the literature: *J. Am. Chem. Soc.* **2019**, *141*, 127.

Synthesis of 2-(*tert*-butyldimethylsilyl)pyridine

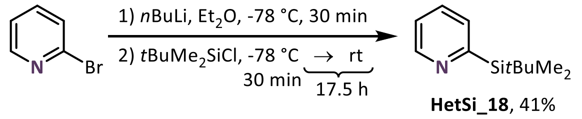

HetSi_18, 41%

A 100 mL Schlenk flask was charged with 2-bromopyridine (4.7485 g, 30.05 mmol) and flushed with argon. Anhydrous Et_2O (40 mL) was added. Resulting solution was placed in an acetone bath (–78 °C). *n*-Butyllithium solution (13.5 mL, 2.5 M in hexanes, 33.8 mmol, 1.1 equiv.) was added dropwise over ca. 4 min. After 30 min, a solution of tBuMe$_2$SiCl (5.431 g, 36.04 mmol, 1.2 equiv.) in anhydrous Et_2O (10 mL) was added. After next 30 min, the Schlenk flask was taken out of the bath and it was allowed to warm to room temperature. After 17.5 h of stirring at room temperature, the mixture was quenched with an aqueous solution of $NaHCO_3$ (20 mL, 5%). Then, the mixture was transferred into separatory funnel, another portion of an aqueous solution of $NaHCO_3$ (80 mL, 5%) was added, and the mixture was extracted with ethyl acetate (3×100 mL). Combined organic layers were washed with water (100 mL), brine (100 mL), and dried over anhydrous $MgSO_4$. The mixture was filtered, evaporated, and the product was separated by column chromatography, to afford 2-(*tert*-butyldimethylsilyl)pyridine (2.404 g, 12.43 mmol, 41%) as an orange oil.

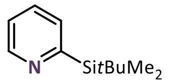

HetSi_18

Yield: 41% (2.404 g, 12.43 mmol, 30.05 mmol scale), an orange oil

Eluent: cyclohexane/ethyl acetate 10:1 to 6:1 (+ 1% NEt_3, v/v)

^{1}H NMR (400 MHz, $CDCl_3$): δ = 8.77 (d, *J*=4.9 Hz, 1H), 7.57–7.51 (m, 1H), 7.47 (d, *J*=7.4 Hz, 1H), 7.16 (ddd, *J*=7.3, 4.8, 1.2 Hz, 1H), 0.90 (s, 9H), 0.31 (s, 6H).

^{13}C NMR (100 MHz, $CDCl_3$): δ = 166.9, 150.0, 133.6, 130.0, 122.6, 26.7, 17.0, –6.2.

^{1}H NMR spectrum was consistent with that reported in the literature: *Synthesis* **2011**, (16), 2590.

Synthesis of 2-(triisopropylsilyl)pyridine

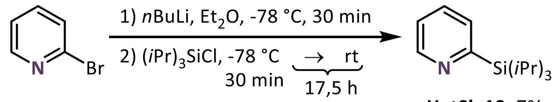

HetSi_19, 7%

A 100 mL Schlenk flask was charged with 2-bromopyridine (4.785 g, 30.29 mmol) and flushed with argon. Anhydrous Et_2O (40 mL) was added. Resulting solution was placed in an acetone bath (-78 °C) and *n*-butyllithium solution (13.5 mL, 2.5 M in hexanes, 33.8 mmol, 1.1 equiv.) was added dropwise over ca. 5 min. After 30 min, *i*Pr_3SiCl (7.75 mL, 36.2 mmol, 1.2 equiv.) was added dropwise (ca. 1 min). After next 30 min, the Schlenk flask was taken out of the bath and it was allowed to warm to room temperature. After 17.5 h of stirring at room temperature, the mixture was quenched with an aqueous solution of $NaHCO_3$ (20 mL, 5%). The mixture was transferred into separatory funnel, where another portion of an aqueous solution of $NaHCO_3$ (80 mL, 5%) was added, and it was extracted with ethyl acetate (3×100 mL). Combined organic layers were washed with water (100 mL), brine (100 mL), and dried over anhydrous $MgSO_4$. The mixture was filtered, evaporated, and the product was separated by column chromatography, to afford 2-(triisopropylsilyl)pyridine (0.503 g, 2.13 mmol, 7%), as a yellowish oil.

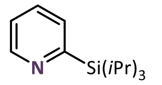

HetSi_19

Yield: 7% (0.503 g, 2.13 mmol, 30.29 mmol scale), a yellowish oil

Eluent: cyclohexane, then cyclohexane/ethyl acetate 20:1 to 5:1 (+1% NEt_3, v/v)

^{1}H NMR (400 MHz, $CDCl_3$): δ = 8.81–8.74 (m, 1H), 7.57–7.48 (m, 1H), 7.48–7.41 (m, 1H), 7.13 (ddd, *J*=7.5, 4.8, 1.5 Hz, 1H), 1.47 (hept, *J*=7.5 Hz, 3H), 1.09 (d, *J*=7.7 Hz, 18H).

^{13}C NMR (100 MHz, $CDCl_3$): δ = 165.1, 150.1, 133.3, 130.9, 122.4, 18.6, 11.0.

NMR spectra were consistent with those reported in the literature: *J. Am. Chem. Soc.* **2007**, *129*, 5332.

Synthesis of 3-(trimethylsilyl)pyridine

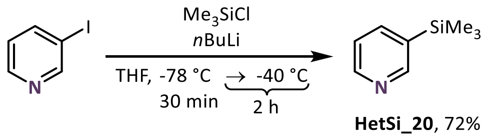

HetSi_20, 72%

A 100 mL Schlenk flask was charged with 3-iodopyridine (4.475 g, 21.83 mmol) and flushed with argon. Anhydrous THF (40 mL) was added. Resulting mixture was placed in an acetone bath (–78 °C), where Me_3SiCl (5.54 mL, 43.7 mmol, 2.0 equiv.) was added. Then, *n*-butyllithium solution (13.1 mL, 2.5 M in hexanes, 32.8 mmol, 1.5 equiv.) was added dropwise over ca. 5 min. After 30 min, the Schlenk flask allowed to warm to –40 °C (reached after ca. 1 h). After 1 h of additional stirring at –40 °C, the mixture was poured onto a vigorously stirred aqueous solution of $NaHCO_3$ (100 mL, 5%). Then, the mixture was transferred into separatory funnel and it was extracted with ethyl acetate (3×100 mL). Combined organic layers were washed with water (100 mL), brine (100 mL), and dried over anhydrous $MgSO_4$. The mixture was filtered, evaporated, and the product was separated by column chromatography, to afford 3-(trimethylsilyl)pyridine (2.364 g, 15.63 mmol, 72%), as a yellowish oil.

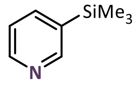

HetSi_20

Yield: 72% (2.364 g, 15.63 mmol, 21.83 mmol scale), a yellowish oil

Eluent: cyclohexane, then cyclohexane/ethyl acetate 20:1 to 2:1

^{1}H NMR (400 MHz, $CDCl_3$): δ = 8.63 (dd, *J*=1.8, 1.0 Hz, 1H), 8.51 (dd, *J*=4.9, 1.9 Hz, 1H), 7.75–7.71 (m, 1H), 7.19 (ddd, *J*=7.5, 4.9, 1.1 Hz, 1H), 0.24 (s, 9H).

^{13}C NMR (100 MHz, $CDCl_3$): δ = 153.7, 149.7, 141.4, 135.4, 123.4, –1.2.

NMR spectra were consistent with these reported in the literature: *Chem. Eur. J.* **2016**, *22*, 2930.

Synthesis of 4-(trimethylsilyl)pyridine

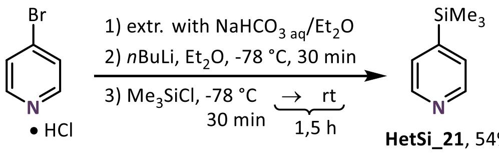

HetSi_21, 54%

A 400 mL beaker was charged with 4-bromopyridine hydrochloride (9.760 g, 50.19 mmol), and the compound was dissolved in H_2O (25 mL). Then, Et_2O (50 mL) was added and to the resulting vigorously stirred solution, and a saturated aqueous solution of $NaHCO_3$ (100 mL) was added in small portions. After 5 min of stirring, the mixture was transferred into separatory funnel, the phases were separated and aqueous phase was extracted with Et_2O (50 mL). Then, a saturated aqueous solution of $NaHCO_3$ (50 mL) was added to the aqueous phase, which was extracted with another portion of Et_2O (50 mL). Combined organic layers were then washed with brine (50 mL) and dried over anhydrous $MgSO_4$. The mixture was filtered into 250 mL round-bottom flask and it was concentrated on the rotary evaporator, where majority of Et_2O was evaporated. Then the flask was flushed with argon and anhydrous Et_2O (40

mL) was added. Resulting mixture was placed in an acetone bath (–78 °C). Then, *n*-butyllithium solution (22.0 mL, 2.5 M in hexanes, 55.0 mmol, 1.1. equiv.) was added dropwise over ca. 10 min. Then, 30 min after the end of *n*-BuLi addition, Me_3SiCl (7.75 mL, 61.1 mmol, 1.2 equiv.) was added dropwise (1-2 min). After 30 min the Schlenk flask was taken out of the acetone bath and it was allowed to warm to room temperature. After 1.5 h of stirring at room temperature, the mixture was poured onto a vigorously stirred aqueous solution of $NaHCO_3$ (100 mL, 5%). Then, the mixture was transferred into separatory funnel and it was extracted with ethyl acetate (3×100 mL). Combined organic layers were washed with water (100 mL), brine (100 mL), and dried over anhydrous $MgSO_4$. The mixture was filtered, evaporated, and the product was separated by column chromatography, to afford 4-(trimethylsilyl)pyridine (4.110 g, 27.17 mmol, 54%), as a pale yellow oil.

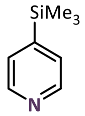

HetSi_21

Yield: 54% (4.110 g, 27.17 mmol, 50.19 mmol scale), a pale yellow oil

Eluent: cyclohexane/ethyl acetate 10:1 to 4:1 (+ 0,5% NEt_3, v/v)

^{1}H NMR (400 MHz, $CDCl_3$): δ = 8.53–8.49 (m, 2H), 7.34–7.31 (m, 2H), 0.23 (s, 9H).

^{13}C NMR (100 MHz, $CDCl_3$): δ = 150.2, 148.7, 128.1, –1.8.

NMR spectra were consistent with these reported in the literature: *Org. Lett.* **2001**, *3*, 1197.

2.2. Synthesis of acyl chlorides

Majority of acyl chlorides were commercially available and were used as received. *O*-Acetylsalicyloyl chloride, dehydroabietic acid chloride and 3-(fluorosulfonyl)benzoyl chloride were synthesized from the corresponding carboxylic acids following the procedures described below.

Synthesis of *O*-acetylsalicyloyl chloride

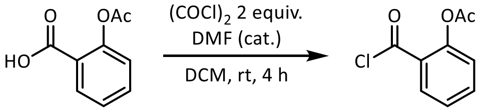

COCl_01, >99%

A 250 mL round-bottom flask was charged with *O*-acetylsalicylic acid (9.009 g, 50.01 mmol) and flushed with argon. Anhydrous DCM (90 mL) and anhydrous DMF (three drops) were added. To the resulting solution, oxalyl chloride (8.6 mL, 100 mmol, 2.0 equiv.) was added dropwise. After 4 h, the mixture was concentrated on the rotary evaporator and the residue was dried under vacuum to afford *O*-acetylsalicyloyl chloride (9.902 g, 49.86 mmol, >99%), as a yellowish solid.

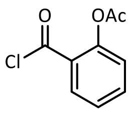

COCl_01

Yield: >99% (9.902 g, 49.86 mmol, 50.01 mmol scale), a yellowish solid,
mp: 48.5–51.5 °C (Lit. 48–50 °C: *Pharm. Chem. J.* **2023**, *57*, 243.)

^{1}H NMR (400 MHz, $CDCl_3$): δ = 8.24 (ddd, *J*=8.0, 1.7, 0.4 Hz, 1H), 7.71–7.65 (m, 1H), 7.44–7.38 (m, 1H), 7.16 (ddd, *J*=8.1, 1.2, 0.4 Hz, 1H), 2.35 (s, 3H).

^{13}C NMR (100 MHz, $CDCl_3$): δ = 169.3, 164.8, 150.5, 136.2, 134.5, 126.62, 126.56, 124.4, 21.0.

NMR spectra were consistent with those reported in the literature: *J. Am. Chem. Soc.* **2015**, *137*, 8324.

Synthesis of dehydroabietic acid chloride

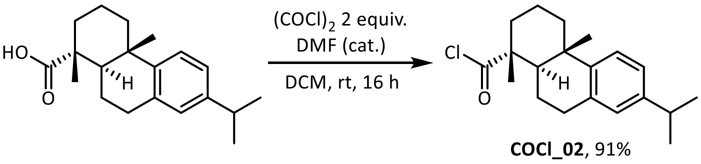

COCl_02, 91%

A 50 mL round-bottom flask was charged with dehydroabietic acid (1.577 g, 5.25 mmol) and flushed with argon. Anhydrous DCM (15 mL) and anhydrous DMF (one drop) were added. To the resulting solution, oxalyl chloride (0.90 mL, 10.5 mmol, 2.0 equiv.) was added dropwise. After 16 h, the mixture was concentrated on the rotary evaporator and the residue was dried under vacuum to afford dehydroabietic acid chloride (1.528 g, 4.79 mmol, 91%), as a pale brown oil.

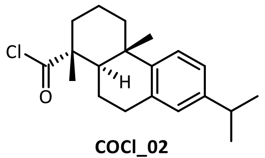

COCl_02

Yield: 91% (1.528 g, 4.79 mmol, 5.25 mmol scale), a pale brown oil

^{1}H NMR (400 MHz, $CDCl_3$): δ = 7.18 (d, *J*=8.1 Hz, 1H), 7.03 (d, *J*=8.2 Hz, 1H), 6.91 (s, 1H), 3.05–2.78 (m, 3H), 2.41–2.29 (m, 2H), 1.98–1.71 (m, 5H), 1.57–1.46 (m, 2H), 1.39 (s, 3H), 1.24 (s, 3H), 1.23 (s, 6H).

^{13}C NMR (100 MHz, $CDCl_3$): δ = 181.7, 146.3, 146.2, 134.6, 127.1, 124.31, 124.26, 58.1, 45.0, 37.7, 37.1, 35.9, 33.6, 30.0, 25.2, 24.1, 21.9, 18.5, 17.9.

NMR spectra were consistent with those reported in the literature: *Chem. Nat. Compd.* **2022**, *58*, 874.

Dehydroabietic acid was prepared from abietic acid (technical grade) according to the procedure described in the literature: *Org. Biomol. Chem.* **2019**, *17*, 4736.

Synthesis of 3-(fluorosulfonyl)benzoyl chloride

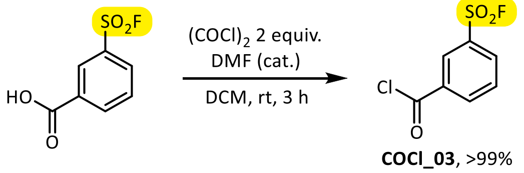

COCl_03, >99%

A 50 mL round-bottom flask was charged with 3-(fluorosulfonyl)benzoic acid (2.450 g, 12.00 mmol) and flushed with argon. Anhydrous DCM (30 mL) and anhydrous DMF (one drop) were added. To the resulting suspension, oxalyl chloride (2.1 mL, 24.5 mmol, 2.0 equiv.) was added dropwise (suspension dissolved). After 3 h, the mixture was concentrated on the rotary evaporator and the residue was dried under vacuum to afford 3-(fluorosulfonyl)benzoyl chloride (2.670 g, 12.00 mmol, >99%), as a pale brown oil.

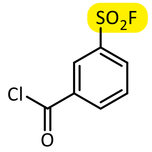

COCl_03

Yield: >99% (2.670 g, 12.00 mmol, 12.00 mmol scale), a pale brown oil

^{1}H NMR (400 MHz, $CDCl_3$): δ = 8.72–8.69 (m, 1H), 8.52 (d, *J*=8.0 Hz, 1H), 8.35–8.29 (m, 1H), 7.92–7.84 (m, 1H).

^{13}C NMR (100 MHz, $CDCl_3$): δ = 166.6, 137.5, 135.0, 134.6 (d, *J*=26.7 Hz), 134.3, 131.0, 130.9.

^{19}F NMR (376 MHz, $CDCl_3$): δ = 65.78, 65.73 (resonance of ^{34}S molecule, ca. 5%).

2.3. Synthesis of acyl fluorides

Benzoyl fluoride and 2-chlorobenzoyl fluoride were synthesized during our previous work, reported in: *Synthesis* **2022**, *54*, 1446.

Acyl fluorides listed below were synthesized according to the procedure reported in the literature: *Org. Lett.* **2019**, *21*, 1659.

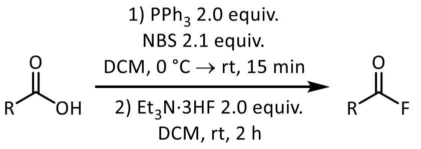

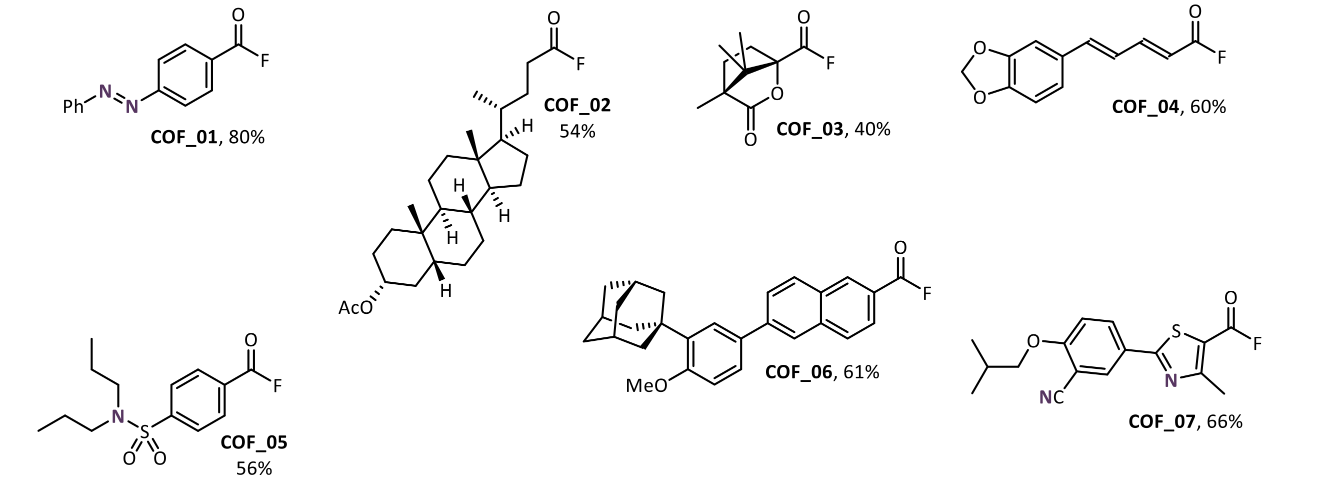

A 500 mL round-bottom flask was charged with carboxylic acid and flushed with argon. Anhydrous DCM was added and the flask was placed in an ice-water bath. Then, PPh_3 (2.0 equiv.) and NBS (2.1 equiv.) were added as solids, with vigorous stirring. After 2 min the bath was removed and after next 15 min at room temperature $Et_3N{\cdot}3HF$ (2.0 equiv.) was added. After 2 h of stirring at room temperature, hexane was added and after next 10 min the resulting mixture was filtered through a pad of silica, with subsequent washing out with solvents indicated below. After evaporation of solvents and drying, analytically pure products were obtained.

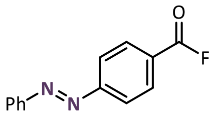

COF_01

Synthesized at 5.49 mmol scale, using DCM (25 mL), hexane (120 mL), silica pad (ø=6 cm, L=3 cm) was washed with DCM/hexane (200 mL, 1:1)

Yield: 80% (1.003 g, 4.39 mmol, 5.49 mmol scale), an orange solid,
mp: 96–100 °C

^{1}H NMR (400 MHz, $CDCl_3$): δ = 8.20 (d, *J*=8.6 Hz, 2H), 8.01 (d, *J*=8.6 Hz, 2H), 8.00–7.94 (m, 2H), 7.60–7.51 (m, 3H).

^{13}C NMR (100 MHz, $CDCl_3$): δ = 157.0 (d, *J*=344 Hz), 156.5, 152.5, 132.7 (d, *J*=3.8 Hz), 132.4, 129.4, 126.3 (d, *J*=61.4 Hz), 123.5, 123.3.

^{19}F NMR (376 MHz, $CDCl_3$): δ = 18.7.

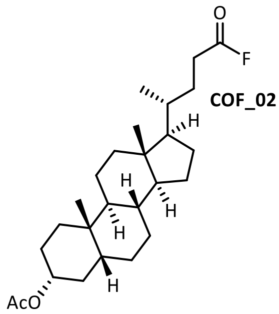

COF_02

Synthesized at 3.00 mmol scale, using DCM (15 mL), hexane (120 mL), silica pad (ø=6 cm, L=3 cm) was washed with DCM/hexane (300 mL, 1:1)

Yield: 64% (0.805 g, 1.91 mmol, 3.00 mmol scale), a white solid,
mp: 158–160.5 °C (Lit. 158–159 °C: *J. Org. Chem.* **1962**, *27*, 3164.)

^{1}H NMR (400 MHz, $CDCl_3$): δ = 4.66 (ddd, *J*=16.1, 10.2, 4.7 Hz, 1H), 2.56–2.44 (m, 1H), 2.43–2.30 (m, 1H), 1.97 (s, 3H), 1.95–1.89 (m, 1H), 1.87–1.71 (m, 5H), 1.68–1.59 (m, 1H), 1.58–1.45 (m, 2H), 1.44–1.28 (m, 8H), 1.28–0.93 (m, 10H), 0.92–0.85 (m, 6H), 0.61 (s, 3H).

^{13}C NMR (100 MHz, $CDCl_3$): δ = 170.5, 164.0 (d, *J*=360 Hz), 74.3, 56.5, 55.9, 42.8, 41.9, 40.4, 40.1, 35.8, 35.1, 35.0, 34.6, 32.2, 30.0, 29.2 (d, *J*=50.0 Hz), 28.2, 27.0, 26.6, 26.3, 24.2, 23.3, 21.4, 20.8, 18.1, 12.0.

^{19}F NMR (376 MHz, $CDCl_3$): δ = 45.3.

O-Acetylation of the lithocholic acid was performed according to the procedure reported in the literature: *Eur. J. Med. Chem.* **2014**, *86*, 279.

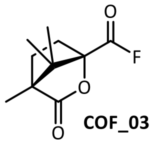

COF_03

Synthesized at 6.00 mmol scale, using DCM (25 mL), hexane (200 mL), silica pad (ø=6 cm, L=3 cm) was washed with DCM/hexane (250 mL, 1:1)

Yield: 40% (0.475 g, 2.37 mmol, 6.00 mmol scale), a white malleable solid

^{1}H NMR (400 MHz, $CDCl_3$): δ = 2.43 (ddd, *J* = 13.4, 10.8, 4.2 Hz, 1H), 2.14 (ddd, *J* = 13.7, 9.4, 4.6 Hz, 1H), 1.99 (ddd, *J* = 13.3, 10.8, 4.6 Hz, 1H), 1.73 (ddd, *J* = 13.5, 9.4, 4.2 Hz, 1H), 1.12 (s, 3H), 1.09 (s, 1H), 1.02 (s, 3H).

^{13}C NMR (100 MHz, $CDCl_3$): δ = 176.8, 157.9 (d, *J*=364 Hz), 89.1 (d, *J*=60.7 Hz), 55.1 (d, *J*=1.9 Hz), 54.8 (d, *J*=2.7 Hz), 30.6, 28.8, 16.63, 16.60 (d, *J*=1.9 Hz), 9.6.

^{19}F NMR (376 MHz, $CDCl_3$): δ = 35.5.

NMR spectra were consistent with those reported in the literature: *Org. Lett.* **2023**, *25*, 9025.

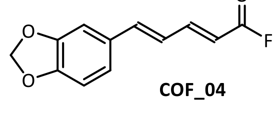

COF_04

Synthesized at 7.99 mmol scale, using DCM (30 mL), hexane (200 mL), silica pad (ø=6 cm, L=3 cm) was washed with DCM/hexane (250 mL, 1:1)

Yield: 60% (1.055 g, 4.79 mmol, 7.99 mmol scale), yellowish solid, **mp:** 100–102 °C

^{1}H NMR (400 MHz, $CDCl_3$): δ = 7.54 (dd, *J*=15.1, 11.1 Hz, 1H), 7.01 (d, *J*=1.7 Hz, 1H), 6.98–6.90 (m, 2H), 6.81 (d, *J*=8.1 Hz, 1H), 6.74 (dd, *J*=15.4, 11.1 Hz, 1H), 6.01 (s, 2H), 5.84 (dd, *J*=15.2, 8.1 Hz, 1H).

^{13}C NMR (100 MHz, CDCl$_3$): δ = 157.4 (d, *J*=336 Hz), 151.5 (d, *J*=6.1 Hz), 149.6, 148.6, 144.1, 129.9, 124.2, 123.5, 113.6 (d, *J*=66.8 Hz), 108.8, 106.2, 101.7.

^{19}F NMR (376 MHz, CDCl$_3$): δ = 23.5 (d, *J*=8.1 Hz).

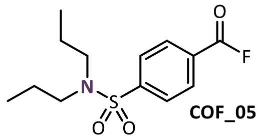

COF_05

Synthesized at 7.60 mmol scale, using DCM (40 mL), hexane (200 mL), silica pad (ø=6 cm, L=2 cm) was washed with DCM/hexane (400 mL, 1:1), after evaporation the mixture was filtered again through a silica pad (ø=4 cm, L=1.5 cm), washing with cyclohexane/ethyl acetate (210 mL, 20:1) to remove residual contamination with Ph_3PO.

Yield: 56% (1.219 g, 4.24 mmol, 7.60 mmol scale), a white solid, **mp:** 71–73.5 °C (Lit. 65–71 °C: *Org. Lett.* **2019**, *21*, 1659.)

^{1}H NMR (400 MHz, CDCl$_3$): δ = 8.21–8.13 (m, 2H), 7.99–7.90 (m, 2H), 3.16–3.08 (m, 4H), 1.62–1.48 (m, 4H), 0.87 (t, *J*=7.4 Hz, 6H).

^{13}C NMR (100 MHz, CDCl$_3$): δ = 156.1 (d, *J*=346 Hz), 146.7, 132.11 (d, *J*=3.4 Hz), 128.1 (d, *J*=62.6 Hz), 127.6, 50.0, 22.0, 11.2.

^{19}F NMR (376 MHz, CDCl$_3$): δ = 19.7.

NMR spectra were consistent with those reported in the literature: *Org. Lett.* **2019**, *21*, 1659.

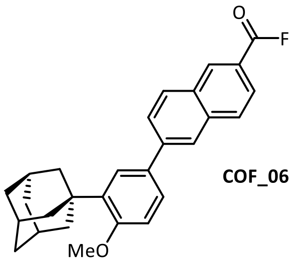

COF_06

Synthesized at 3.10 mmol scale, using DCM (40 mL), hexane (200 mL), silica pad (ø=6 cm, L=2 cm) was washed with DCM/hexane (300 mL, 1:1)

Yield: 61% (0.780 g, 1.88 mmol, 3.10 mmol scale), a white solid,
mp: 230–232 °C (Lit. 232–233 °C: *Chem. Eur. J.* **2020**, *26*, 16261.)

^{1}H NMR (400 MHz, CDCl$_3$): δ = 8.64 (s, 1H), 8.08–7.94 (m, 4H), 7.86 (dd, *J*=8.5, 1.9 Hz, 1H), 7.62 (d, *J*=2.4 Hz, 1H), 7.56 (dd, *J*=8.4, 2.3 Hz, 1H), 7.01 (d, *J*=8.4 Hz, 1H), 3.92 (s, 3H), 2.20 (bs, 6H), 2.13 (bs, 3H), 1.82 (bs, 6H).

^{13}C NMR (100 MHz, CDCl$_3$): δ = 159.3, 157.9 (d, *J*=343 Hz), 143.0, 139.3, 137.1, 133.9 (d, *J*=3.1 Hz), 132.2, 131.1, 130.1, 129.2, 127.3, 126.14, 126.09, 126.0, 124.9, 121.5 (d, *J*=60.7 Hz), 112.3, 55.3, 40.7, 37.4, 37.2, 29.2.

^{19}F NMR (376 MHz, CDCl$_3$): δ = 17.1.

NMR spectra were consistent with those reported in the literature: *Chem. Eur. J.* **2020**, *26*, 16261.

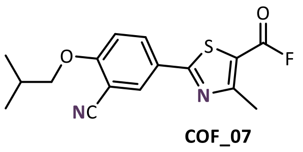

Synthesized at 10.01 mmol scale, using DCM (40 mL), hexane (200 mL), silica pad (ø=6 cm, L=3 cm) was washed with DCM/hexane (300 mL, 1:1)

Yield: 66% (2.119 g, 6.65 mmol, 10.01 mmol scale), a white solid, **mp:** 154–155 °C (Lit. 153–155 °C: *Org. Lett.* **2023**, *25*, 9025.)

^{1}H NMR (400 MHz, $CDCl_3$): δ = 8.22 (d, *J*=2.3 Hz, 1H), 8.11 (dd, *J*=8.9, 2.3 Hz, 1H), 7.04 (d, *J*=8.9 Hz, 1H), 3.92 (d, *J*=6.5 Hz, 2H), 2.79 (s, 3H), 2.21 (hept, *J*=6.7 Hz, 1H), 1.09 (d, *J*=6.7 Hz, 6H).

^{13}C NMR (100 MHz, $CDCl_3$): δ = 170.7, 166.6 (d, *J*=6.9 Hz), 163.1, 151.9 (d, *J*=330 Hz), 133.0, 132.4, 125.1, 115.7 (d, *J*=70.2 Hz), 115.1, 112.8, 103.1, 75.8, 28.2, 19.0, 17.8 (d, *J*=2.7 Hz).

^{19}F NMR (376 MHz, $CDCl_3$): δ = 37.1.

NMR spectra were consistent with those reported in the literature: *Org. Lett.* **2023**, *25*, 9025.

Acyl fluorides listed below were synthesized according to the procedure reported in the literature: *Indian J. Chem., Sect. B: Org. Chem. Incl. Med. Chem.* **2000**, *39,* 384.

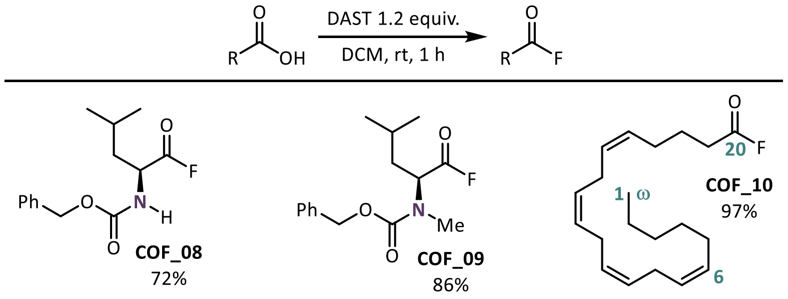

A 100 mL round-bottom flask was charged with carboxylic acid and flushed with argon. Anhydrous DCM was added and then diethylaminosulfur trifluoride, DAST (1.2 equiv.) was added dropwise with vigorous stirring. After 1 h, the mixture was quenched with ice-cold water (50 mL). Layers were separated and the organic layer was dried over anhydrous Na_2SO_4. Then, the mixture was filtered through a Schott funnel with a pad of silica (ø=4 cm, L=1 cm), which was washed with DCM (100 mL). After evaporation of solvent and drying, analytically pure products were obtained.

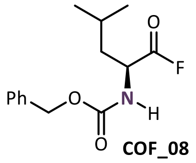

Synthesized at 1.00 mmol scale, using DCM (10 mL)

Yield: 72% (0.194 g, 0.72 mmol, 1.00 mmol scale), a colorless oil

^{1}H NMR (400 MHz, $CDCl_3$): δ = 7.42–7.29 (m, 5H), 5.14 (s, 2H), 5.08 (d, *J*=8.4 Hz, 1H), 4.59–4.47 (m, 1H), 1.82–1.66 (m, 2H), 1.67–1.56 (m, 1H), 0.97 (d, *J*=5.9 Hz, 6H).

^{13}C NMR (100 MHz, CDCl$_3$): δ = 163.3 (d, *J*=372 Hz), 156.0, 135.9, 128.7, 128.4, 128.2, 67.5, 51.4 (d, *J*=60.3 Hz), 39.9, 24.7, 22.7, 21.5.

^{19}F NMR (376 MHz, CDCl$_3$): δ = 29.3.

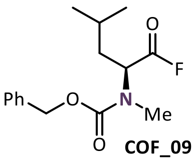

COF_09

Synthesized at 5.01 mmol scale, using DCM (30 mL)

Yield: 86% (1.216 g, 4.32 mmol, 5.01 mmol scale), a colorless oil

^{1}H NMR (400 MHz, CDCl$_3$): δ = 7.42–7.29 (m, 5H), 5.18 (s, 2H), 5.13–5.03 and 4.92–4.83 (m, 1H), 3.00–2.90 (m, 3H), 1.91–1.49 (m, 3H), 1.03–0.92 (m, 5H), 0.87 (d, *J*=6.3 Hz, 1H).

^{13}C NMR (100 MHz, CDCl$_3$): δ (two rotamers) = 162.3 (d, *J*=375 Hz) and 162.0 (d, *J*=375 Hz), 156.8 and 155.7, 136.3 and 136.0, 128.6, 128.4, 128.3 and 128.2, 127.9, 68.1 and 67.9, 56.1 and 55.6, 37.3 and 36.9, 30.9 and 30.5, 24.7 and 24.5, 23.09 and 23.07, 21.2 and 21.0.

^{19}F NMR (376 MHz, CDCl$_3$): δ = 31.6.

N-methylation of Cbz-Leucine was performed according to the literature procedure: *J. Am. Chem. Soc.* **1997**, *119*, 2111.

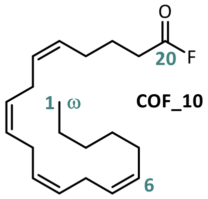

COF_10

Synthesized at 5.00 mmol scale, using DCM (30 mL)

Yield: 97% (1.493 g, 4.87 mmol, 5.00 mmol scale), a yellowish oil

^{1}H NMR (400 MHz, CDCl$_3$): δ = 5.51–5.28 (m, 8H), 2.88–2.75 (m, 6H), 2.51 (t, *J*=7.4 Hz, 2H), 2.17 (q, *J*=7.4 Hz, 2H), 2.06 (q, *J*=7.0 Hz, 2H), 1.76 (p, *J*=7.3 Hz, 2H), 1.41–1.25 (m, 6H), 0.89 (t, *J*=6.8 Hz, 3H).

^{13}C NMR (100 MHz, CDCl$_3$): δ = 163.5 (d, *J*=360 Hz), 130.6, 129.8, 128.7, 128.5, 128.1, 127.9, 127.8, 127.6, 31.6, 31.5 (d, *J*=50.4 Hz), 29.4, 27.3, 26.1, 25.73 (d, *J*=1.1 Hz), 25.70, 23.8 (d, *J*=2.3 Hz), 22.7, 14.2.

^{19}F NMR (376 MHz, CDCl$_3$): δ = 45.2.

3. Experimental procedures for the synthesis of 2-pyridyl ketones

3.1. General Procedure A: Synthesis of ketones 1a-r

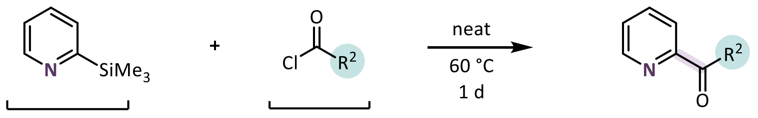

A 20 mL Schlenk flask was charged with the corresponding acyl chloride (2.0 mmol, 1.0 equiv.) and flushed with argon. Then, 2-(trimethylsilyl)pyridine (0.34 mL, 2.0 mmol, 1.0 equiv.) was added. The flask was sealed, placed in an oil bath (60 °C), and the mixture was stirred for 24 h. Then, the Schlenk flask was taken out of the bath, the mixture was allowed to cool to room temperature and it was directly separated with column chromatography (L=ca. 15-25 cm, ø=3 cm, 100-200 mL of silica gel), and eluted with eluents indicated hereinafter (typically cyclohexane/ethyl acetate).

1a, synthesized according to the General Procedure A

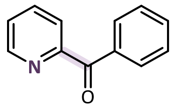

Yield: 91% (0.338 g, 1.84 mmol, 2.02 mmol scale), a pale yellow oil

Eluent: cyclohexane/ethyl acetate 10:1 to 1:1 (+ 0.5% NEt_3(v/v))

^{1}H NMR (400 MHz, $CDCl_3$): δ = 8.73 (ddd, *J*=4.8, 1.7, 0.9 Hz, 1H), 8.08–8.03 (m, 3H), 7.94–7.88 (m, 1H), 7.62–7.57 (m, 1H), 7.52–7.45 (m, 3H).

^{13}C NMR (100 MHz, $CDCl_3$): δ = 194.0, 155.2, 148.7, 137.1, 136.4, 133.0, 131.1, 128.3, 126.3, 124.7.

NMR spectra were consistent with those reported in the literature: *Angew. Chem. Int. Ed.* **2012**, *51*, 2745.

In this reaction a double addition-acylation byproduct **4e** was isolated as a more polar fraction:

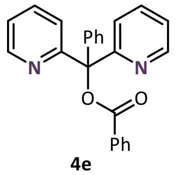

4e

Yield: 8% (0.029 g, 0.08 mmol, 2.02 mmol scale), a white solid, **mp:** 170 °C (dec).

Eluent: cyclohexane/ethyl acetate 10:1 to 1:1 (+ 0.5% NEt_3(v/v))

^{1}H NMR (400 MHz, $CDCl_3$): δ = 8.60–8.54 (m, 2H), 8.26–8.18 (m, 2H), 7.86 (d, *J*=8.1 Hz, 2H), 7.73–7.66 (m, 2H), 7.63–7.53 (m, 3H), 7.52–7.45 (m, 2H), 7.38–7.29 (m, 2H), 7.30–7.23 (m, 1H), 7.16 (dd, *J*=7.5, 4.8 Hz, 2H).

^{13}C NMR (100 MHz, $CDCl_3$): δ = 164.8, 160.9, 148.8, 142.5, 136.1, 133.2, 131.0, 130.1, 128.5, 128.3, 128.0, 127.6, 123.1, 122.2, 89.4.

MS (EI) m/z: (%) 366 (1, [M^+]), 261 (100), 245 (99), 167 (27), 105 (25), 77 (26).

HRMS (EI) m/z: calcd. for $C_{24}H_{18}N_2O_2$ 366.1368 [M^+]; found 366.1365.

The same reaction repeated in solution (2.0 mmol scale, 2 mL of solvent, 60 °C, 1 d) gave the following results:

MeCN – 100% conversion, 85% isolated yield (+5% of byproduct),

THF – ca. 80% conversion (GC, NMR),

toluene – ca. 70% conversion (GC, NMR).

1b, synthesized according to the General Procedure A

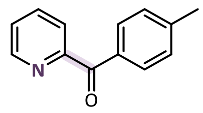

Yield: 88% (0.348 g, 1.76 mmol, 2.01 mmol scale), a colorless oil

Eluent: cyclohexane, then cyclohexane/ethyl acetate 10:1 to 6:1 (+ 0.5% NEt_3(v/v))

^{1}H NMR (400 MHz, $CDCl_3$): δ = 8.72 (d, *J*=4.8 Hz, 1H), 8.01 (d, *J*=7.9 Hz, 1H), 7.97 (d, *J*=8.2 Hz, 2H), 7.93–7.85 (m, 1H), 7.47 (ddd, *J*=7.6, 4.8, 1.3 Hz, 1H), 7.29 (d, *J*=8.2 Hz, 2H), 2.43 (s, 3H).

^{13}C NMR (100 MHz, $CDCl_3$): δ = 193.5, 155.4, 148.5, 143.7, 137.0, 133.6, 131.1, 128.9, 126.0, 124.5, 21.7.

NMR spectra were consistent with those reported in the literature: *Angew. Chem. Int. Ed.* **2012**, *51*, 2745.

1c, synthesized according to the General Procedure A

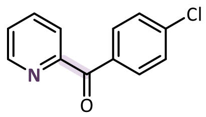

Yield: 81% (0.354 g, 1.63 mmol, 2.00 mmol scale), a white solid,
mp = 63.5–65 °C (Lit. 63–64 °C: *Angew. Chem. Int. Ed.* **2012**, *51*, 2745.)

Eluent: cyclohexane, then cyclohexane/ethyl acetate 15:1 to 10:1 (+ 0.5% NEt_3(v/v))

^{1}H NMR (400 MHz, $CDCl_3$): δ = 8.72 (ddd, *J*=4.8, 1.8, 0.9 Hz, 1H), 8.09–8.04 (m, 3H), 7.94–7.89 (m, 1H), 7.51 (ddd, *J*=7.6, 4.8, 1.3 Hz, 1H), 7.50–7.39 (m, 2H).

^{13}C NMR (100 MHz, $CDCl_3$): δ = 192.5, 154.8, 148.6, 139.5, 137.3, 134.7, 132.6, 128.6, 126.5, 124.8.

NMR spectra were consistent with those reported in the literature: *Angew. Chem. Int. Ed.* **2012**, *51*, 2745.

1d, synthesized according to the General Procedure A

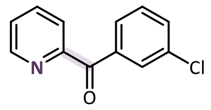

Yield: 88% (0.386 g, 1.77 mmol, 2.02 mmol scale), a white solid,
mp = 78–79 °C (Lit. 79 °C: *Russ. Chem. Bull.* **2002**, *51*, 540.)

Eluent: cyclohexane, then cyclohexane/ethyl acetate 10:1 to 3:1 (+ 0.5% NEt_3(v/v))

^{1}H NMR (400 MHz, $CDCl_3$): δ = 8.72 (ddd, *J*=4.8, 1.7, 0.9 Hz, 1H), 8.09–8.04 (m, 2H), 7.97 (ddd, *J*=7.8, 1.6, 1.1 Hz, 1H), 7.95–7.87 (m, 1H), 7.55 (ddd, *J*=8.0, 2.2, 1.1 Hz, 1H), 7.50 (ddd, *J*=7.6, 4.8, 1.3 Hz, 1H), 7.45–7.39 (m, 1H).

^{13}C NMR (100 MHz, $CDCl_3$): δ = 192.4, 154.6, 148.7, 138.0, 137.3, 134.4, 132.9, 131.1, 129.6, 129.3, 126.7, 124.8.

NMR spectra were consistent with those reported in the literature: *Org. Lett.* **2019**, *21*, 5321.

In this reaction a double addition-acylation byproduct **4f** was isolated as a more polar fraction:

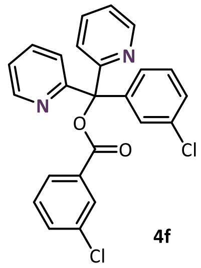

4f

Yield: 10% (0.043 g, 0.10 mmol, 2.02 mmol scale), a white solid,
mp = 110.5–112.5 °C

Eluent: cyclohexane, then cyclohexane/ethyl acetate 10:1 to 3:1 (+ 0.5% NEt_3(v/v))

^{1}H NMR (400 MHz, $CDCl_3$): δ = 8.57 (ddd, *J*=4.8, 1.9, 1.0 Hz, 2H), 8.14 (ddd, *J*=2.0, 1.5, 0.4 Hz, 1H), 8.06 (ddd, *J*=7.8, 1.6, 1.1 Hz, 1H), 7.81–7.77 (m, 2H), 7.76–7.67 (m, 2H), 7.58 (ddd, *J*=8.0, 2.2, 1.1 Hz, 1H), 7.53–7.50 (m, 1H), 7.47–7.41 (m, 1H), 7.41–7.36 (m, 1H), 7.28–7.21 (m, 2H), 7.19 (ddd, *J*=7.4, 4.8, 1.2 Hz, 2H).

^{13}C NMR (100 MHz, $CDCl_3$): δ = 163.6, 159.9, 149.0, 144.4, 136.4, 134.8, 134.0, 133.4, 132.5, 130.1, 130.0, 129.2, 128.7, 128.2, 128.0, 126.7, 123.0, 122.5, 89.2.

MS (EI) m/z: (%) 434 (1, [M^+]), 295 (67), 279 (100), 244 (37), 216 (10), 201 (11), 139 (24), 111 (29).

HRMS (ESI) m/z: calcd. for $C_{24}H_{16}[^{35}Cl]_2N_2O_2$ [M^+] 434.0589; found 434.0568.

1e, synthesized according to the General Procedure A

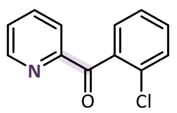

Yield: 94% (0.407 g, 1.87 mmol, 1.99 mmol scale), a white solid,
mp = 51–52 °C (Lit. 50–51°C: *Chem. Eur. J.* **2014**, *20*, 4156.)

Eluent: cyclohexane, then cyclohexane/ethyl acetate 15:1 to 7:1 (+ 0.5% NEt_3(v/v))

^{1}H NMR (400 MHz, $CDCl_3$): δ = 8.68 (ddd, *J*=4.8, 1.7, 0.9 Hz, 1H), 8.17–8.10 (m, 1H), 7.95–7.86 (m, 1H), 7.53–7.35 (m, 5H).

^{13}C NMR (100 MHz, $CDCl_3$): δ = 195.4, 153.9, 149.4, 138.3, 137.1, 132.0, 131.7, 130.04, 130.03, 127.1, 126.7, 123.8.

NMR spectra were consistent with those reported in the literature: *Chem. Eur. J.* **2014**, *20*, 4156.

1f, synthesized according to the General Procedure A

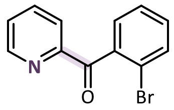

Yield: 96% (0.502 g, 1.91 mmol, 1.99 mmol scale), a yellowish solid,
mp: 54.5–56 °C (Lit. 63.1–63.9 °C: *Org. Lett.* **2006**, *8*, 2523.)

Eluent: cyclohexane, then cyclohexane/ethyl acetate 10:1 (+ 0.5% NEt_3(v/v))

^{1}H NMR (400 MHz, $CDCl_3$): δ = 8.68 (ddd, *J*=4.7, 1.7, 0.9 Hz, 1H), 8.17–8.14 (m, 1H), 7.93–7.88 (m, 1H), 7.63 (ddd, *J*=7.9, 1.2, 0.4 Hz, 1H), 7.50–7.40 (m, 3H), 7.36 (ddd, *J*=7.9, 7.1, 2.1 Hz, 1H).

^{13}C NMR (100 MHz, $CDCl_3$): δ = 195.9, 153.6, 149.5, 140.4, 137.2, 133.2, 131.6, 130.0, 127.2, 127.1, 124.1, 120.2.

NMR spectra were consistent with those reported in the literature: *Org. Lett.* **2006**, *8*, 2523.

1g, synthesized according to the General Procedure A

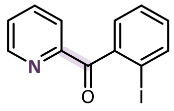

Yield: 89% (0.550 g, 1.78 mmol, 2.00 mmol scale), a yellowish solid, **mp:** 86–87 °C (Lit. 85–86 °C: *Org. Mass. Spectrom.* **1980**, *15*, 122.)

Eluent: cyclohexane/ethyl acetate 10:1 to 6:1 (+ 0.5% NEt_3(v/v))

^{1}H NMR (400 MHz, $CDCl_3$): δ = 8.68 (ddd, *J*=4.7, 1.7, 0.9 Hz, 1H), 8.21–8.14 (m, 1H), 7.95–7.86 (m, 2H), 7.57–7.35 (m, 3H), 7.18 (ddd, *J*=7.9, 7.3, 1.9 Hz, 1H).

^{13}C NMR (100 MHz, $CDCl_3$): δ = 196.9, 153.0, 149.4, 143.9, 139.7, 137.2, 131.6, 129.6, 127.8, 127.1, 124.6, 92.8.

NMR spectra were consistent with those reported in the literature: *Chem. Eur. J.* **2014**, *20*, 9910.

1h, synthesized according to the General Procedure A

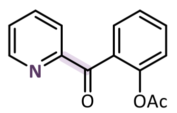

Yield: 81% (0.387 g, 1.60 mmol, 1.98 mmol scale), a white solid, **mp:** 80–81.5 °C

Eluent: cyclohexane/ethyl acetate 6:1 to 3:1 (+ 0.5% NEt_3(v/v))

^{1}H NMR (400 MHz, $CDCl_3$): δ = 8.56 (ddd, *J*=4.8, 1.8, 1.0 Hz, 1H), 7.83 (dd, *J*=7.8, 1.7 Hz, 1H), 7.71–7.64 (m, 1H), 7.61–7.55 (m, 1H), 7.51 – 7.45 (m, 1H), 7.20 (ddd, *J*=7.5, 4.8, 1.2 Hz, 1H), 7.08 (dd, *J*=8.3, 1.1 Hz, 1H), 7.04–6.97 (m, 1H), 2.05 (s, 3H).

^{13}C NMR (100 MHz, $CDCl_3$): δ = 161.3, 157.8, 156.4, 149.5, 137.1, 136.5, 129.6, 123.9, 122.9, 121.1, 117.3, 114.6, 105.9, 27.4.

NMR spectra were partially consistent with those reported in the literature: *J. Am. Chem. Soc.* **2010**, *132*, 14400.

1i, synthesized according to the General Procedure A

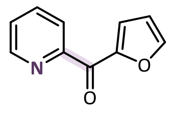

Yield: 73% (0.253 g, 1.46 mmol, 2.01 mmol scale), a pale yellow oil

Eluent: cyclohexane, then cyclohexane/ethyl acetate 10:1 to 1:1 (+ 0.5% NEt_3(v/v))

^{1}H NMR (400 MHz, $CDCl_3$): δ = 8.73 (ddd, *J*=4.7, 1.8, 0.9 Hz, 1H), 8.20–8.13 (m, 1H), 8.05 (dd, *J*=3.6, 0.8 Hz, 1H), 7.91–7.85 (m, 1H), 7.75 (dd, *J*=1.7, 0.8 Hz, 1H), 7.49 (ddd, *J*=7.6, 4.8, 1.3 Hz, 1H), 6.62 (dd, *J*=3.6, 1.7 Hz, 1H).

^{13}C NMR (100 MHz, $CDCl_3$): δ = 179.1, 153.8, 151.1, 148.7, 147.7, 137.0, 126.7, 124.3, 123.8, 112.4.

NMR spectra were consistent with those reported in the literature: *Adv. Synth. Catal.* **2017**, *359*, 1588.

In this reaction an O-silylated double addition byproduct **4g** was isolated as a more polar fraction:

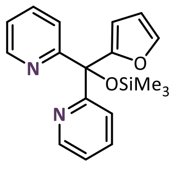

4g

Yield: 13% (0.086 g, 0.27 mmol, 2.01 mmol scale), a yellowish oil

Eluent: cyclohexane, then cyclohexane/ethyl acetate 10:1 to 1:1 (+ 0.5% NEt_3(v/v))

^{1}H NMR (400 MHz, $CDCl_3$): δ = = 8.52 (ddd, *J* = 4.8, 1.8, 1.0 Hz, 2H), 7.72 – 7.61 (m, 4H), 7.39 (dd, *J* = 1.8, 0.9 Hz, 1H), 7.10 (ddd, *J* = 7.2, 4.8, 1.4 Hz, 2H), 6.34 (dd, *J* = 3.3, 1.9 Hz, 1H), 6.22 (dd, *J* = 3.3, 0.9 Hz, 1H), –0.05 (s, 9H).

^{13}C NMR (100 MHz, $CDCl_3$): δ = 163.2, 156.4, 148.5, 141.9, 136.3, 122.2, 121.6, 110.9, 110.5, 81.3, 1.4.

MS (EI) m/z: (%) 324 (45, [M^+]), 309 (93), 246 (100), 235 (25), 205 (53), 78 (27), 74 (68).

HRMS (ESI) m/z: calcd. for $C_{18}H_{20}N_2O_2Si$ [M^+] 324.1294; found 324.1297

1j, synthesized according to the General Procedure A, using 2-(trimethylsilyl)pyridine (**2 equiv.**, **4 mmol**)

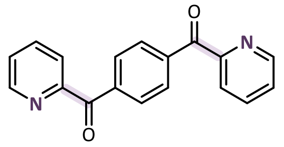

Yield: 61% (0.354 g, 1.23 mmol, 2.01 mmol scale), a white solid,
mp: 184.5–186 °C (Lit. 184–186 °C: *Tetrahedron* **2004**, *60*, 3421.)

Eluent: cyclohexane/ethyl acetate 20:1 to 1:5 (+ 0.5% NEt_3(v/v))

^{1}H NMR (400 MHz, $CDCl_3$): δ = 8.73 (ddd, *J*=4.7, 1.7, 0.9 Hz, 2H), 8.17 (s, 4H), 8.12–8.07 (m, 2H), 7.98–7.87 (m, 2H), 7.51 (ddd, *J*=7.6, 4.8, 1.3 Hz, 2H).

^{13}C NMR (100 MHz, $CDCl_3$): δ = 193.4, 154.6, 148.8, 139.6, 137.3, 130.7, 126.7, 124.8.

NMR spectra were consistent with those reported in the literature: *Chem. Eur. J.* **2022**, *28*, e202200648.

1k, synthesized according to the General Procedure A, using 2-(trimethylsilyl)pyridine (**2 equiv.**, **4 mmol**)

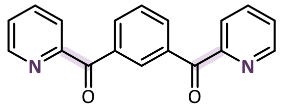

Yield: 54% (0.313 g, 1.09 mmol, 2.01 mmol scale), a white solid,
mp: 133.5–134 °C (Lit. 128–130 °C: *Tetrahedron* **2004**, *60*, 3421.)

Eluent: cyclohexane/ethyl acetate 10:1 to 1:1 (+ 0.5% NEt_3(v/v))

^{1}H NMR (400 MHz, $CDCl_3$): δ = 8.84–8.78 (m, 1H), 8.72 (ddd, *J*=4.8, 1.8, 0.9 Hz, 2H), 8.34 (dd, *J*=7.7, 1.8 Hz, 2H), 8.09–8.06 (m, 2H), 7.92–7.88 (m, 2H), 7.66–7.58 (m, 1H), 7.49 (ddd, *J*=7.6, 4.8, 1.2 Hz, 2H).

^{13}C NMR (100 MHz, $CDCl_3$): δ = 192.9, 154.5, 148.6, 137.1, 136.2, 135.0, 133.9, 128.1, 126.5, 124.7.

NMR spectra were consistent with those reported in the literature: *Tetrahedron* **2004**, *60*, 3421.

1l, synthesized according to the General Procedure A

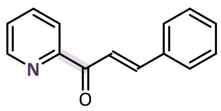

Yield: 71% (0.297 g, 1.42 mmol, 2.00 mmol scale), a brown solid,
mp: 61–62.5 °C (Lit. 65.4–67.5 °C: *Chem. Eur. J.* **2013**, *19*, 5242.)

Eluent: cyclohexane, then cyclohexane/ethyl acetate 50:1 to 20:1 (+ 0.5% NEt_3(v/v))

^{1}H NMR (400 MHz, $CDCl_3$): δ = 8.75 (ddd, *J*=4.8, 1.8, 0.9 Hz, 1H), 8.31 (d, *J*=16.1 Hz, 1H), 8.20 (ddd, *J*=7.9, 0.9 Hz, 1H), 7.95 (d, *J*=16.1 Hz, 1H), 7.92–7.83 (m, 1H), 7.76–7.72 (m, 2H), 7.49 (ddd, *J*=7.6, 4.7, 1.3 Hz, 1H), 7.44–7.39 (m, 3H).

^{13}C NMR (100 MHz, $CDCl_3$): δ = 189.6, 154.3, 149.0, 144.9, 137.1, 135.3, 130.7, 128.96, 128.94, 127.0, 123.0, 121.0.

NMR spectra were consistent with those reported in the literature: *Org. Lett.* **2018**, *20*, 1906.

1m, synthesized according to the General Procedure A

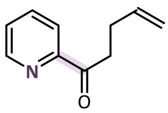

Yield: 68% (0.219 g, 1.35 mmol, 2.00 mmol scale), a pale yellow oil

Eluent: cyclohexane, then cyclohexane/ethyl acetate 50:1 to 20:1 (+ 0.5% NEt_3(v/v))

^{1}H NMR (400 MHz, $CDCl_3$): δ = 8.60 (ddd, *J*=4.8, 1.8, 0.9 Hz, 1H), 7.97–7.93 (m, 1H), 7.79–7.71 (m, 1H), 7.39 (ddd, *J*=7.5, 4.8, 1.3 Hz, 1H), 5.84 (ddt, *J*=16.8, 10.2, 6.5 Hz, 1H), 5.06–4.96 (m, 1H), 4.94–4.88 (m, 1H), 3.30–3.21 (m, 2H), 2.46–2.36 (m, 2H).

^{13}C NMR (100 MHz, $CDCl_3$): δ = 201.1, 153.4, 148.9, 137.4, 136.8, 127.1, 121.7, 115.0, 36.8, 27.9.

NMR spectra were consistent with those reported in the literature: *Org. Biomol. Chem.* **2012**, *10*, 70.

1n, synthesized according to the General Procedure A

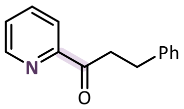

Yield: 70% (0.295 g, 1.40 mmol, 2.00 mmol scale), a pale yellow oil

Eluent: cyclohexane, then cyclohexane/ethyl acetate 50:1 to 20:1 (+ 0.5% NEt_3(v/v))

^{1}H NMR (400 MHz, $CDCl_3$): δ = 8.66 (ddd, *J*=4.8, 1.7, 0.9 Hz, 1H), 8.06–8.03 (m, 1H), 7.84–7.78 (m, 1H), 7.47–7.41 (m, 1H), 7.31–7.27 (m, 4H), 7.22–7.16 (m, 1H), 3.63–3.54 (m, 2H), 3.13–3.04 (m, 2H).

^{13}C NMR (100 MHz, $CDCl_3$): δ = 201.0, 153.4, 149.0, 141.5, 136.9, 128.55, 128.45, 127.2, 126.0, 121.8, 39.5, 29.9.

NMR spectra were consistent with those reported in the literature: *Org. Lett.* **2020**, *22*, 493.

1o, synthesized according to the General Procedure A

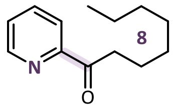

Yield: 87% (0.355 g, 1.73 mmol, 1.99 mmol scale), a brown oil

Eluent: cyclohexane, then cyclohexane/ethyl acetate 10:1 (+ 0.5% NEt_3(v/v))

^{1}H NMR (400 MHz, $CDCl_3$): δ = 8.68 (ddd, *J*=4.8, 1.7, 0.9 Hz, 1H), 8.07–8.01 (m, 1H), 7.87–7.78 (m, 1H), 7.45 (ddd, *J*=7.5, 4.8, 1.3 Hz, 1H), 3.21 (dd, *J*=7.8, 7.1 Hz, 2H), 1.78–1.68 (m, 2H), 1.44–1.32 (m, 4H), 1.32–1.23 (m, 4H), 0.93–0.83 (m, 3H).

^{13}C NMR (100 MHz, $CDCl_3$): δ = 202.4, 153.7, 149.0, 137.0, 127.1, 121.9, 37.9, 31.9, 29.5, 29.3, 24.1, 22.8, 14.2.

MS (EI) m/z: (%) 205 (18, [M+]), 177 (10), 148 (22), 134 (69), 121 (29), 106 (50), 79 (100).

HRMS (ESI) m/z: calc. for $C_{13}H_{19}NO$ [M^+] 205.1467; found 205.1476.

1p, synthesized according to the General Procedure A

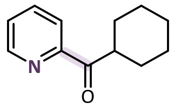

Yield: 84% (0.318 g, 1.68 mmol, 2.01 mmol scale), a yellowish oil

Eluent: cyclohexane, then cyclohexane/ethyl acetate 10:1 (+ 0.5% NEt_3(v/v))

^{1}H NMR (400 MHz, $CDCl_3$): δ = 8.67 (ddd, *J*=4.8, 1.8, 0.9 Hz, 1H), 8.02–7.99 (m, 1H), 7.85–7.76 (m, 1H), 7.43 (ddd, *J*=7.6, 4.8, 1.3 Hz, 1H), 3.93–3.80 (m, 1H), 1.96–1.86 (m, 2H), 1.84–1.77 (m, 2H), 1.76–1.68 (m, 1H), 1.50–1.36 (m, 4H), 1.32–1.17 (m, 1H).

^{13}C NMR (100 MHz, $CDCl_3$): δ = 205.1, 153.2, 149.0, 137.0, 126.9, 122.6, 44.1, 29.0, 26.2, 25.9.

NMR spectra were consistent with those reported in the literature: *Chem. Eur. J.* **2014**, *20*, 4156.

1q, synthesized according to the General Procedure A, at **180 °C**, using 2-(trimethylsilyl)pyridine (**2.0 equiv.**, **2.0 mmol**), **1.0 mmol scale**

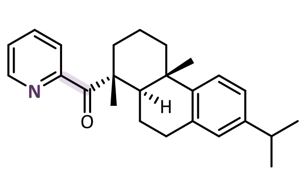

Yield: 78% (0.284 g, 0.78 mmol, 1.00 mmol scale), a yellowish oil

Eluent: cyclohexane/ethyl acetate 10:1 to 8:1 (+ 0.5% NEt_3(v/v))

^{1}H NMR (400 MHz, $CDCl_3$): δ = 8.54 (d, *J*=4.8 Hz, 1H), 7.81–7.73 (m, 1H), 7.70 (d, *J*=7.9 Hz, 1H), 7.36–7.29 (m, 1H), 7.22 (d, *J*=8.2 Hz, 1H), 7.03 (d, *J*=8.1 Hz, 1H), 6.87 (s, 1H), 2.95–2.64 (m, 4H), 2.51–2.39 (m, 1H), 2.35 (d, *J*=12.7 Hz, 1H), 2.02–1.75 (m, 4H), 1.69–1.57 (m, 1H), 1.50–1.39 (m, 4H), 1.30 (s, 3H), 1.24 (d, *J*=7.0 Hz, 6H).

^{13}C NMR (100 MHz, $CDCl_3$): δ = 208.4, 157.1, 147.7, 147.3, 145.6, 136.7, 135.1, 127.0, 125.1, 124.4, 123.9, 123.5, 53.1, 43.3, 37.8, 37.3, 35.3, 33.5, 30.4, 25.8, 24.10, 24.08, 22.1, 18.9, 16.9.

MS (EI) m/z: (%) 361 (44, [M+]), 239 (17), 185 (18), 173 (42), 107 (71), 79 (100).

HRMS (EI) m/z: calcd. for $C_{25}H_{31}NO$ 361.2406 [M+]; found 361.2401.

1r, synthesized according to the General Procedure A, at **120 °C**

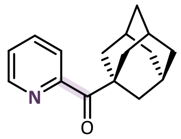

Yield: 72% (0.345 g, 1.43 mmol, 1.98 mmol scale), a yellowish solid, **mp:** 120.5–121.5 °C

Eluent: cyclohexane, then cyclohexane/ethyl acetate 20:1 to 15:1 (+ 0.5% NEt_3(v/v))

^{1}H NMR (400 MHz, $CDCl_3$): δ = 8.65–8.58 (m, 1H), 7.81–7.72 (m, 2H), 7.41–7.30 (m, 1H), 2.24–2.19 (m, 6H), 2.10–2.04 (m, 3H), 1.80–1.76 (m, 6H).

^{13}C NMR (100 MHz, $CDCl_3$): δ = 206.8, 155.8, 147.8, 136.7, 125.6, 123.5, 47.0, 38.7, 36.9, 28.4.

MS (EI) m/z: (%) 241 (100, [M^+]), 213 (18), 135 (54), 93 (22), 79 (34).

HRMS (EI) m/z: calcd. for $C_{16}H_{19}NO$ 241.1467 [M^+]; found 241.1472.

NMR spectra were consistent with those reported in the literature: *J. Flow Chem.* **2021**, *11*, 91.

3.2. General procedure B: Synthesis of ketones 2a-l

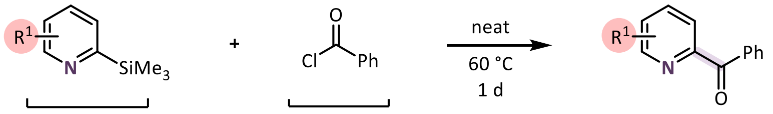

2a-2l

A 20 mL Schlenk flask was charged with corresponding substituted 2-(trimethylsilyl)pyridine (2.0 mmol, 1.0 equiv.) and flushed with argon. Then, benzoyl chloride (0.24 mL, 2.0 mmol, 1.0 equiv.) was added. The flask was sealed, placed in an oil bath (60 °C) and the mixture was stirred for 24 h. Then, the Schlenk flask was taken out of the bath, the mixture was allowed to cool to room temperature and it was directly separated with column chromatography (L=ca. 15-25 cm, ø=3 cm, 100-200 mL of silica gel), and eluted with eluents indicated hereinafter (typically cyclohexane/ethyl acetate).

2a, synthesized according to the General Procedure B

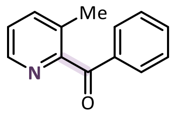

Yield: 79% (0.313 g, 1.59 mmol, 2.01 mmol scale), a yellowish oil

Eluent: cyclohexane, then cyclohexane/ethyl acetate 10:1 to 8:1 (+ 0.5% NEt_3 (v/v))

^{1}H NMR (400 MHz, $CDCl_3$): δ = 8.49 (ddd, *J*=4.8, 1.5, 0.7 Hz, 1H), 7.90–7.82 (m, 2H), 7.64 (ddq, *J*=7.8, 1.5, 0.7 Hz, 1H), 7.62–7.53 (m, 1H), 7.48–7.42 (m, 2H), 7.32 (dd, *J*=7.8, 4.7 Hz, 1H), 2.40 (s, 3H).

^{13}C NMR (100 MHz, $CDCl_3$): δ = 195.3, 155.0, 146.0, 139.0, 136.4, 133.4, 132.8, 130.5, 128.4, 124.7, 18.4.

NMR spectra were consistent with those reported in the literature: *J. Org. Chem.* **2018**, *83*, 12420.

2b, synthesized according to the General Procedure B, using benzoyl chloride (**2.0 equiv., 4.0 mmol**), stirring for **48 h**

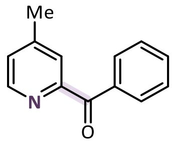

Yield: 49% (0.194 g, 0.98 mmol, 2.01 mmol scale), a colorless oil

Eluent: toluene, then toluene/ethyl acetate 20:1 to 10:1 (+ 0.5% NEt_3 (v/v))

^{1}H NMR (400 MHz, $CDCl_3$): δ = 8.55 (d, *J*=5.0 Hz, 1H), 8.07–8.00 (m, 2H), 7.86–7.81 (m, 1H), 7.59–7.52 (m, 1H), 7.50–7.42 (m, 2H), 7.30–7.24 (m, 1H), 2.43 (s, 3H).

^{13}C NMR (100 MHz, $CDCl_3$): δ = 194.3, 155.0, 148.5, 148.4, 136.5, 132.9, 131.0, 128.2, 127.1, 125.4, 21.2.

NMR spectra were consistent with those reported in the literature: *J. Org. Chem.* **2018**, *83*, 12420.

2c, synthesized according to the General Procedure B

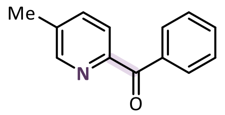

Yield: 92% (0.365 g, 1.85 mmol, 2.01 mmol scale), a yellowish oil

Eluent: cyclohexane/ethyl acetate 15:1 to 6:1 (+ 0.5% NEt_3(v/v))

^{1}H NMR (400 MHz, $CDCl_3$): δ = 8.56–8.51 (m, 1H), 8.08–8.01 (m, 2H), 7.95 (d, *J*=8.0 Hz, 1H), 7.70–7.65 (m, 1H), 7.60–7.53 (m, 1H), 7.51–7.42 (m, 2H), 2.43 (s, 3H).

^{13}C NMR (100 MHz, $CDCl_3$): δ = 193.8, 152.5, 149.1, 137.5, 136.7, 136.6, 132.8, 131.0, 128.2, 124.5, 18.8.

NMR spectra were consistent with those reported in the literature: *J. Org. Chem.* **2020**, *85*, 3942.

2d, synthesized according to the General Procedure B

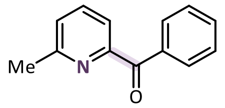

Yield: 90% (0.357 g, 1.81 mmol, 2.00 mmol scale), a colorless oil

Eluent: cyclohexane/ethyl acetate 20:1 to 10:1 (+ 0.5% NEt_3(v/v))

^{1}H NMR (400 MHz, $CDCl_3$): δ = 8.12–8.06 (m, 2H), 7.79–7.72 (m, 2H), 7.62–7.50 (m, 1H), 7.54–7.42 (m, 2H), 7.33 (dd, *J*=6.7, 2.2 Hz, 1H), 2.63 (s, 3H).

^{13}C NMR (100 MHz, $CDCl_3$): δ = 193.8, 157.6, 154.5, 137.0, 136.2, 132.8, 131.1, 128.0, 125.7, 121.6, 24.4.

NMR spectra were consistent with those reported in the literature: *J. Org. Chem.* **2018**, *83*, 12420.

2e, synthesized according to the General Procedure B

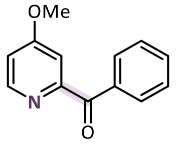

Yield: 88% (0.380 g, 1.78 mmol; 2.01 mmol scale), a yellowish solid,
mp: 52.5–53 °C (Lit. 45–47 °C: *Org. Lett.* **2021**, *23*, 6099.)

Eluent: cyclohexane/ethyl acetate 20:1 to 5:1 (+ 0.5% NEt_3(v/v))

^{1}H NMR (400 MHz, $CDCl_3$): δ = 8.54 (dd, *J*=5.8, 2.5 Hz, 1H), 8.08–8.03 (m, 2H), 7.63–7.54 (m, 2H), 7.48 (dd, *J*=8.4, 7.1 Hz, 2H), 7.01 (dd, *J*=5.7, 2.5 Hz, 1H), 3.95 (s, 3H).

^{13}C NMR (100 MHz, $CDCl_3$): δ = 193.8, 166.8, 156.7, 149.8, 136.3, 133.1, 131.1, 128.3, 112.9, 110.2, 55.7.

HRMS (APCI) m/z: calc. for $C_{13}H_{11}NO_2$ [M+H$^+$] 214.0868; found 214.0876.

NMR spectra were consistent with those reported in the literature: *Org. Lett.* **2021**, *23*, 6099.

2f, synthesized according to the General Procedure B

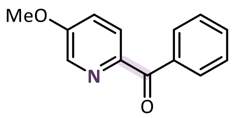

Yield: 92% (0.388 g, 1.82 mmol, 1.99 mmol scale), a white solid,
mp: 79.5–80.5 °C (Lit. 75 °C: *J. Org. Chem.* **1971**, *36*, 2002.)

Eluent: cyclohexane/ethyl acetate 15:1 to 6:1 (+ 0.5% NEt_3(v/v))

^{1}H NMR (400 MHz, $CDCl_3$): δ = 8.38 (d, *J*=2.9 Hz, 1H), 8.11 (d, *J*=8.7 Hz, 1H), 8.08–8.01 (m, 2H), 7.60–7.53 (m, 1H), 7.51–7.43 (m, 2H), 7.33 (dd, *J*=8.7, 2.9 Hz, 1H), 3.94 (s, 3H).

^{13}C NMR (100 MHz, $CDCl_3$): δ = 193.0, 157.8, 147.7, 137.0, 136.7, 132.6, 131.0, 128.1, 126.4, 120.3, 55.9.

HRMS (APCI) m/z: calc. for $C_{13}H_{11}NO_2$ [M+H$^+$] 214.0868; found 214.0873.

NMR spectra were consistent with those reported in the literature: *J. Org. Chem.* **2020**, *85*, 3942.

2g, synthesized according to the General Procedure B, at **100 °C**

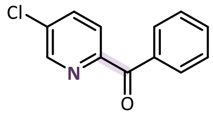

Yield: 75% (0.329 g, 1.51 mmol, 2.01 mmol scale), a white solid, **mp:** 60.5–62 °C (Lit. 62 °C: *Beilstein J. Org. Chem.* **2016,** *12,* 144.)

Eluent: cyclohexane : ethyl acetate 20:1 to 5:1 (+ 0.5% NEt_3(v/v))

^{1}H NMR (400 MHz, $CDCl_3$): δ = 8.67 (dd, *J*=2.4, 0.7 Hz, 1H), 8.08–8.02 (m, 3H), 7.88 (dd, *J*=8.4, 2.4 Hz, 1H), 7.64–7.58 (m, 1H), 7.52–7.47 (m, 2H).

^{13}C NMR (100 MHz, $CDCl_3$): δ = 192.6, 152.9, 147.5, 136.9, 136.0, 135.2, 133.2, 131.0, 128.3, 125.7.

NMR spectra were consistent with those reported in the literature: *Beilstein J. Org. Chem.* **2016,** *12,* 144.

2h, synthesized according to the General Procedure B, at **100 °C**

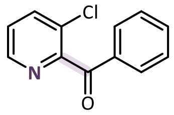

Yield: 78% (0.344 g, 1.58 mmol, 2.02 mmol scale), a yellowish oil

Eluent: cyclohexane/ethyl acetate 10:1 to 2:1 (+ 0.5% NEt_3(v/v))

^{1}H NMR (400 MHz, $CDCl_3$): δ = 8.59 (dd, *J*=4.7, 1.4 Hz, 1H), 7.87–7.83 (m, 3H), 7.64–7.59 (m, 1H), 7.51–7.45 (m, 2H), 7.40 (dd, *J*=8.2, 4.7 Hz, 1H).

^{13}C NMR (100 MHz, $CDCl_3$): δ = 192.5, 154.3, 147.0, 137.9, 135.1, 134.0, 130.2, 129.4, 128.6, 125.5.

NMR spectra were consistent with those reported in the literature: *Beilstein J. Org. Chem.* **2016,** *12,* 144.

2i, synthesized according to the General Procedure B, at **120 °C**

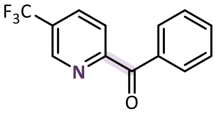

Yield: 61% (0.305 g, 1.21 mmol, 2.00 mmol scale), a yellowish oil

Eluent: DCM (+ 0.5% NEt_3(v/v))

^{1}H NMR (400 MHz, $CDCl_3$): δ = 8.98 (s, 1H), 8.14 (d, *J*=1.6 Hz, 2H), 8.09–8.05 (m, 2H), 7.65–7.59 (m, 1H), 7.53–7.46 (m, 2H).

^{13}C NMR (100 MHz, $CDCl_3$): δ = 192.5, 158.0 (q, *J*=1.4 Hz), 145.6 (q, *J*=3.9 Hz), 135.6, 134.5 (q, *J*=3.5 Hz), 133.6, 131.1, 128.5 (q, *J*=33.2 Hz), 128.4, 124.4, 123.2 (q, *J*=273 Hz).

^{19}F NMR (376 MHz, $CDCl_3$): δ = –63.1.

MS (EI) m/z: (%) 251 (32, [M^+]), 223 (53), 105 (100), 77 (79), 52 (23).

HRMS (EI) m/z: calc. for $C_{13}H_8F_3NO$ [M^+] 251.0558; found 251.0556.

In this reaction a double addition-acylation byproduct **4h** was isolated as a less polar fraction:

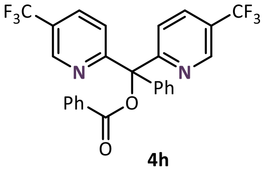

4h

Yield: 23% (0.116 g, 0.23 mmol, 2.00 mmol scale), a colorless oil

Eluent: DCM (+ 0.5% NEt_3(v/v))

^{1}H NMR (400 MHz, $CDCl_3$): δ = 8.84 (s, 2H), 8.25–8.19 (m, 2H), 8.05 (d, *J*=8.4 Hz, 2H), 7.97 (dd, *J*=8.5, 2.3 Hz, 2H), 7.68–7.63 (m, 1H), 7.58–7.49 (m, 4H), 7.41–7.30 (m, 3H).

^{13}C NMR (100 MHz, $CDCl_3$): δ = 164.9, 164.1 (q, *J*=1.5 Hz), 145.7 (q, *J*=4.1 Hz), 141.2, 133.7, 133.6 (q, *J*=3.4 Hz), 130.3, 130.1, 128.8, 128.45, 128.36, 127.9, 125.3 (q, *J*=33.2 Hz), 123.5 (q, *J*=273 Hz), 123.2, 88.7.

^{19}F NMR (376 MHz, $CDCl_3$): δ = –62.9.

MS (EI) m/z: (%) 502 (1, [M^+]), 397 (100), 380 (73), 235 (20), 105 (25), 77 (23).

HRMS (EI) m/z: calc. for $C_{26}H_{16}F_6N_2O_2$ [M^+] 502.1116; found 502.1126.

2j, synthesized according to the General Procedure B, at **100 °C**

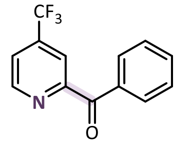

Yield: 63% (0.317 g, 1.26 mmol, 1.99 mmol scale), a yellowish oil

Eluent: cyclohexane/ethyl acetate 20:1 to 6:1 (+ 0.5% NEt_3(v/v))

^{1}H NMR (400 MHz, $CDCl_3$): δ = 8.95–8.89 (m, 1H), 8.32–8.27 (m, 1H), 8.11–8.07 (m, 2H), 7.72 (ddq, *J*=5.1, 1.9, 0.6 Hz, 1H), 7.66–7.61 (m, 1H), 7.54–7.48 (m, 2H).

^{13}C NMR (100 MHz, $CDCl_3$): δ = 192.3, 156.3, 149.6, 139.7 (q, *J*=34.7 Hz), 135.6, 133.5, 131.1, 128.4, 122.6 (q, *J*=274 Hz), 121.6 (q, *J*=3.4 Hz), 120.5 (q, *J*=3.5 Hz).

^{19}F NMR (376 MHz, $CDCl_3$): δ = –65.3.

NMR spectra were consistent with those reported in the literature: *J. Org. Chem.* **2018**, *83*, 12420.

In this case also double addition-acylation byproduct **4i** was isolated as a more polar fraction:

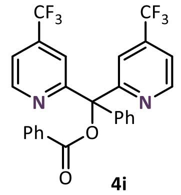

4i

Yield: 27% (0.135 g, 0.27 mmol, 1.99 mmol scale), a yellowish oil

Eluent: cyclohexane/ethyl acetate 20:1 to 6:1 (+ 0.5% NEt_3(v/v))

^{1}H NMR (400 MHz, $CDCl_3$): δ = 8.76–8.69 (m, 2H), 8.20–8.16 (m, 2H), 8.15–8.10 (m, 2H), 7.67–7.61 (m, 1H), 7.56–7.49 (m, 4H), 7.42 (ddt, *J*=5.0, 1.6, 0.7 Hz, 2H), 7.40–7.29 (m, 3H).

^{13}C NMR (100 MHz, $CDCl_3$): δ = 164.8, 162.3, 149.7, 141.0, 138.6 (q, *J*=34.1 Hz), 133.6, 130.4, 130.0, 128.8, 128.5, 128.3, 127.9, 122.9 (q, *J*=273 Hz), 119.0 (q, *J*=3.8 Hz), 118.2 (q, *J*=3.4 Hz), 88.7.

^{19}F NMR (376 MHz, $CDCl_3$): δ = –65.1.

MS (EI) m/z: (%) 502 (1, [M^+]), 397 (100), 380 (85), 235 (16), 209 (13), 105 (29), 77 (24).

HRMS (EI) m/z: calc. for $C_{26}H_{16}F_6N_2O_2$ [M^+] 502.1116; found 502.1129.

2k, synthesized according to the General Procedure B

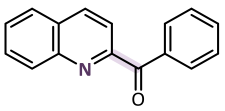

Yield: 78% (0.363 g, 1.56 mmol, 2.00 mmol scale), a white solid,
mp: 105.5–107.5 °C (Lit. 105.3–106.7 °C: *Chem. Commun.* **2021**, *57*, 10234.)

Eluent: cyclohexane, then cyclohexane : ethyl acetate 20:1 to 10:1 (+ 0.5% NEt_3(v/v))

^{1}H NMR (400 MHz, $CDCl_3$): δ = 8.35 (dd, *J*=8.6, 1.0 Hz, 1H), 8.27–8.18 (m, 3H), 8.11 (dd, *J*=8.4, 1.2 Hz, 1H), 7.91 (dd, *J*=8.0, 1.5 Hz, 1H), 7.84–7.75 (m, 1H), 7.71–7.59 (m, 2H), 7.56–7.47 (m, 2H).

^{13}C NMR (100 MHz, $CDCl_3$): δ = 193.9, 154.8, 146.8, 137.2, 136.2, 133.2, 131.6, 130.6, 130.2, 129.0, 128.5, 128.3, 127.8, 120.9.

NMR spectra were consistent with those reported in the literature: *Chem. Commun.* **2021**, *57*, 10234.

2l, synthesized according to the General Procedure B

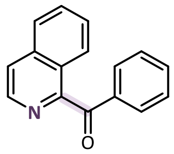

Yield: 89% (0.411 g, 1.76 mmol, 1.98 mmol scale), a white solid,
mp: 75.5–77 °C (Li. 74–76 °C: *J*. Org. *Chem.* **2022**, *87*, 15224.)

Eluent: cyclohexane/ethyl acetate 20:1 to 10:1 (+ 0.5% NEt_3(v/v))

^{1}H NMR (400 MHz, $CDCl_3$): δ = 8.61 (d, *J*=5.6 Hz, 1H), 8.22 (dd, *J*=8.5, 1.1 Hz, 1H), 7.98–7.91 (m, 3H), 7.83 (d, *J*=5.7 Hz, 1H), 7.76 (ddd, *J*=8.2, 6.8, 1.2 Hz, 1H), 7.66–7.59 (m, 2H), 7.57–7.44 (m, 2H).

^{13}C NMR (100 MHz, $CDCl_3$): δ = 194.8, 156.5, 141.2, 136.8, 136.7, 133.8, 130.9, 130.8, 128.6, 128.4, 127.2, 126.5, 126.3, 122.7.

NMR spectra were consistent with those reported in the literature: *J*. Org. *Chem.* **2022**, *87*, 15224.

3.3. General procedure C: Synthesis of ketones 3a-q

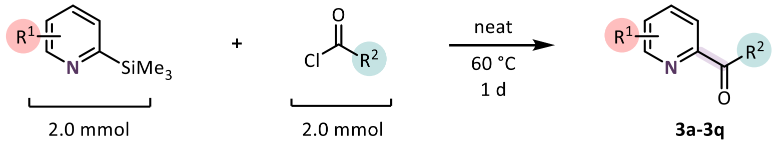

A 20 mL Schlenk flask was charged with the corresponding substituted 2-(trimethylsilyl)pyridine (2.0 mmol, 1.0 equiv.), acyl chloride (2.0 mmol, 1.0 equiv.) and flushed with argon. The flask was sealed, placed in an oil bath (60 °C) and the mixture was stirred for 24 h. Then, the Schlenk flask was taken out of the bath, the mixture was allowed to cool to room temperature and it was directly separated with column chromatography (L=ca. 15-25 cm, ø=3 cm, 100-200 mL of silica gel), and eluted with eluents indicated hereinafter (typically cyclohexane/ethyl acetate).

3a, synthesized according to the General Procedure C

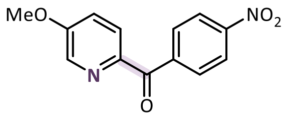

Yield: 98% (0.511 g, 1.980 mmol, 2.02 mmol scale), a yellowish solid,
mp: 165–167 °C

Eluent: cyclohexane/ethyl acetate 10:1 to 6:1 (+ 0.5% NEt_3(v/v)), then cyclohexane/ethyl acetate/DCM 4:1:1 and 1:1:1 (+ 0.5% NEt_3(v/v))

^{1}H NMR (400 MHz, $CDCl_3$): δ = 8.35 (d, *J*=2.9 Hz, 1H), 8.32–8.27 (m, 2H), 8.24–8.18 (m, 3H), 7.37 (dd, *J*=8.8, 2.9 Hz, 1H), 3.96 (s, 3H).

^{13}C NMR (100 MHz, $CDCl_3$): δ = 191.0, 158.5, 149.8, 146.5, 142.5, 137.0, 131.9, 126.7, 123.1, 120.5, 56.0.

MS (EI) m/z: (%) 258 (57, [M^+]), 230 (100), 211 (33), 200 (27), 184 (39), 150 (28), 108 (63), 104 (50).

HRMS (EI) m/z: calcd. for $C_{13}H_{10}N_2O_4$ 258.0641 [M^+]; found. 258.0644.

3b, synthesized according to the General Procedure C

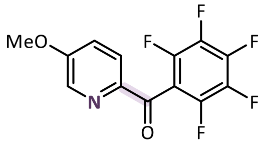

Yield: 65% (0.398 g, 1.31 mmol, 2.01 mmol scale), a yellowish solid,
mp: 68.5–70 °C

Eluent: cyclohexane/ethyl acetate 15:1 to 10:1 (+ 0.5% NEt_3(v/v))

^{1}H NMR (400 MHz, $CDCl_3$): δ = 8.27 (d, *J*=2.8 Hz, 1H), 8.17 (d, *J*=8.7 Hz, 1H), 7.33 (dd, *J*=8.8, 2.8 Hz, 1H), 3.93 (s, 3H).

^{13}C NMR (100 MHz, $CDCl_3$): δ = 185.2, 159.5, 145.3, 144.1 (d of m, *J*=255 Hz), 142.4 (d of m, *J*=256 Hz), 137.5 (d of m, *J*=254 Hz), 138.1, 125.2, 120.1, 114.94 to 114.32 (m), 56.1.

^{19}F NMR (376 MHz, $CDCl_3$): δ = –140.1 to –140.3 (m, 2F), –152.3 (t, *J*=20.3 Hz, 1F), –161.7 to –162.1 (m, 2F).

MS (EI) m/z: (%) 303 (100, [M^+]), 284 (86), 275 (92), 232 (67), 205 (47), 195 (48), 167 (34), 108 (53).

HRMS (EI) m/z: calcd. for $C_{13}H_6NO_2F_5$ 303.0319 [M^+]; found. 303.0315.

3c, synthesized according to the General Procedure C, at **100 °C**

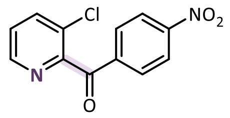

Yield: 68% (0.363 g, 1.38 mmol, 2.02 mmol scale), a yellowish solid,
mp: 150.5–152 °C

Eluent: cyclohexane/ethyl acetate 20:1 to 1:1 (+ 0.5% NEt_3(v/v))

^{1}H NMR (400 MHz, $CDCl_3$): δ = 8.58 (dd, *J*=4.7, 1.4 Hz, 1H), 8.32–8.25 (m, 2H), 8.05–7.98 (m, 2H), 7.89 (dd, *J*=8.3, 1.4 Hz, 1H), 7.46 (dd, *J*=8.2, 4.7 Hz, 1H).

^{13}C NMR (100 MHz, $CDCl_3$): δ = 190.5, 152.6, 150.6, 147.2, 140.1, 138.7, 131.4, 130.4, 126.4, 123.8.

HRMS (APCI) m/z: calcd. for $C_{12}H_8N_2O_3$[^{35}Cl] 263.0223 [$M+H^+$]; found. 263.0222.

3d, synthesized according to the General Procedure C, at **80 °C**

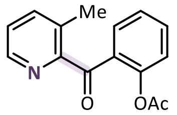

Yield: 52% (0.265 g, 1.04 mmol, 2.01 mmol scale), a white solid,
mp: 120.5–122 °C

Eluent: cyclohexane/ethyl acetate 10:1 to 6:1 (+ 0.5% NEt_3(v/v))

^{1}H NMR (400 MHz, $CDCl_3$): δ = 8.25 (dd, *J*=5.0, 1.5 Hz, 1H), 7.74 (dd, *J*=7.8, 1.7 Hz, 1H), 7.42–7.32 (m, 2H), 7.01 (dd, *J*=7.7, 4.7 Hz, 1H), 6.97 (dd, *J*=8.3, 1.1 Hz, 1H), 6.94–6.88 (m, 1H), 2.46 (s, 3H), 2.01 (s, 3H).

^{13}C NMR (100 MHz, $CDCl_3$): δ = 161.3, 156.0, 154.4, 145.6, 140.6, 136.0, 131.4, 129.3, 123.9, 122.7, 116.6, 114.8, 106.9, 25.1, 19.5.

MS (EI) m/z: (%) 255 (7, [M^+]), 212 (47), 120 (100), 92 (51), 66 (15).

HRMS (EI) m/z: calcd. for $C_{15}H_{13}NO_3$ 255.0895 [M^+]; found. 255.0904.

3e, synthesized according to the General Procedure C, stirring for **48 h**

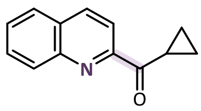

Yield: 87% (0.341 g, 1.73 mmol, 1.99 mmol scale), a white solid,
mp: 65–66 °C

Eluent: cyclohexane/ethyl acetate 20:1 to 10:1 (+ 0.5% NEt_3(v/v))

^{1}H NMR (400 MHz, $CDCl_3$): δ = 8.17 (d, *J*=8.5 Hz, 1H), 8.13 (d, *J*=8.5 Hz, 1H), 8.05 (d, *J*=8.7 Hz, 1H), 7.75 (d, *J*=8.2 Hz, 1H), 7.72–7.67 (m, 1H), 7.58–7.50 (m, 1H), 3.82–3.71 (m, 1H), 1.32–1.21 (m, 2H), 1.16–1.07 (m, 2H).

^{13}C NMR (100 MHz, $CDCl_3$): δ = 201.5, 153.1, 147.1, 136.7, 130.5, 129.8, 129.4, 128.3, 127.5, 118.0, 15.6, 12.9.

MS (EI) m/z: (%) 197 (30, [M^+]), 168 (100), 143 (21), 128 (51), 101 (18).

HRMS (EI) m/z: calcd. for $C_{13}H_{11}NO$ 197.0841 [M^+]; found. 197.0839.

3f, synthesized according to the General Procedure C, stirring at **60 °C** for **24 h**, then at **80 °C** for **24 h**

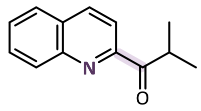

Yield: 58% (0.233 g, 1.17 mmol, 2.01 mmol scale), a colorless oil

Eluent: cyclohexane, then cyclohexane/ethyl acetate 20:1 to 15:1 (+ 0.5% NEt_3(v/v))

^{1}H NMR (400 MHz, $CDCl_3$): δ = 8.22–8.13 (m, 2H), 8.09 (d, *J*=8.5 Hz, 1H), 7.80 (d, *J* = 8.1 Hz, 1H), 7.76–7.69 (m, 1H), 7.62–7.55 (m, 1H), 4.39 (hept, *J*=6.9 Hz, 1H), 1.27 (d, *J*=6.9 Hz, 6H).

^{13}C NMR (100 MHz, $CDCl_3$): δ = 206.2, 152.4, 147.2, 136.8, 130.6, 129.9, 129.5, 128.4, 127.6, 118.8, 34.0, 18.9.

NMR spectra were consistent with those reported in the literature: *Chem. Eur. J.* **2024**, *30*, e202303993.

3g, synthesized according to the General Procedure C

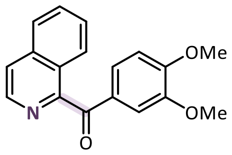

Yield: 64% (0.373 g, 1.27 mmol, 2.00 mmol scale), a white solid,
mp: 139.5–143 °C (Lit. 140–142 °C: *J. Org. Chem.* **2014**, *79*, 3856.)

Eluent: cyclohexane/ethyl acetate 6:1 to 2:1 (+ 0.5% NEt_3(v/v))

^{1}H NMR (400 MHz, $CDCl_3$): δ = 8.60 (d, *J*=5.6 Hz, 1H), 8.16 (d, *J*=8.5 Hz, 1H), 7.92 (d, *J*=8.4 Hz, 1H), 7.79 (d, *J*=5.7 Hz, 1H), 7.76–7.71 (m, 2H), 7.65–7.56 (m, 1H), 7.37 (dd, *J*=8.4, 2.0 Hz, 1H), 6.84 (d, *J*=8.4 Hz, 1H), 3.96 (s, 3H), 3.93 (s, 3H).

^{13}C NMR (100 MHz, CDCl$_3$): δ = 193.0, 156.7, 153.7, 148.8, 140.8, 136.2, 130.3, 129.3, 127.7, 126.7, 126.6, 125.9, 125.7, 121.9, 111.2, 109.7, 55.7, 55.6.

NMR spectra were consistent with those reported in the literature: *J. Org. Chem.* **2014**, *79*, 3856.

3h, synthesized according to the General Procedure C, using 1-(trimethylsilyl)isoquinoline (**2.0 equiv., 4.0 mmol**)

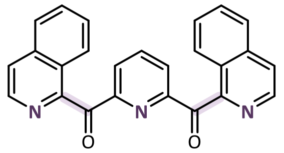

Yield: 56% (0.431 g, 1.11 mmol, 1.99 mmol scale), a yellowish malleable solid

Eluent: cyclohexane/ethyl acetate 1:1 to 1:5 (+ 0.5% NEt_3(v/v))

^{1}H NMR (400 MHz, CDCl$_3$): δ = 8.41 (d, *J*=7.8 Hz, 2H), 8.18 (dd, *J*=8.1, 7.5 Hz, 1H), 8.10 (d, *J*=5.6 Hz, 2H), 7.85–7.79 (m, 2H), 7.72–7.67 (m, 2H), 7.59 (ddd, *J*=8.2, 6.8, 1.2 Hz, 2H), 7.43–7.37 (m, 4H).

^{13}C NMR (100 MHz, CDCl$_3$): δ = 194.1, 156.0, 153.5, 140.6, 138.0, 135.4, 130.0, 127.6, 126.6, 126.5, 125.6, 125.4, 121.6.

MS (EI) m/z: (%) 389 (50, [M$^+$]), 371 (30), 360 (56), 332 (28), 233 (59), 205 (58), 128 (100), 101 (23).

HRMS (EI) m/z: calcd. for $C_{25}H_{15}N_3O_2$ 389.1164 [M$^+$]; found. 389.1171.

3i, synthesized according to the General Procedure C, at **0.9 mmol scale**

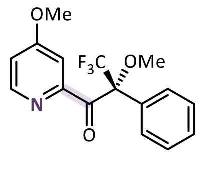

Yield: 59% (0.172 g, 0.53 mmol, 0.90 mmol scale), a colorless oil

Eluent: cyclohexane/ethyl acetate 6:1 to 3:1 (+ 0.5% NEt_3(v/v))

^{1}H NMR (400 MHz, CDCl$_3$): δ = 8.39 (d, *J*=5.7 Hz, 1H), 7.55–7.49 (m, 2H), 7.40 (d, *J*=2.5 Hz, 1H), 7.39–7.30 (m, 3H), 6.83 (dd, *J*=5.6, 2.6 Hz, 1H), 3.78 (s, 3H), 3.60 (q, *J*=1.7 Hz, 3H).

^{13}C NMR (100 MHz, CDCl$_3$): δ = 193.4, 166.1, 152.8, 151.0, 133.9, 129.2, 128.4, 126.6, 123.8 (q, *J*=291 Hz), 112.8, 111.6, 86.8 (q, *J*=25.2 Hz), 56.4 (q, *J*=2.1 Hz), 55.4.

^{19}F NMR (376 MHz, CDCl$_3$): δ = –71.3.

MS (EI) m/z: (%) 325 (54, [M$^+$]), 310 (90), 189 (47), 136 (62), 108 (100).

HRMS (EI) m/z: calcd. for $C_{16}H_{14}NO_3F_3$ 325.0926 [M$^+$]; found. 325.0934.

3j, synthesized according to the General Procedure C, at **0.57 mmol scale**, using 2-(trimethylsilyl)-5-methylpyridine (**1.75 equiv.**, **1.00 mmol**)

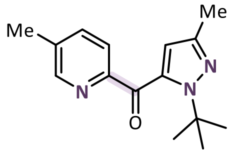

Yield: 55% (0.082 g, 0.32 mmol, 0.57 mmol scale), a colorless oil

Eluent: cyclohexane : ethyl acetate 10:1 to 6:1 (+ 0.5% NEt_3(v/v))

^{1}H NMR (400 MHz, $CDCl_3$): δ = 8.50 (d, *J*=2.1 Hz, 1H), 7.91 (d, *J*=8.0 Hz, 1H), 7.64 (dd, *J*=7.9, 2.2 Hz, 1H), 6.42 (s, 1H), 2.40 (s, 3H), 2.24 (s, 3H), 1.65 (s, 9H).

^{13}C NMR (100 MHz, $CDCl_3$): δ = 186.2, 152.9, 149.5, 144.6, 139.2, 137.3, 137.2, 123.9, 114.8, 61.7, 30.1, 18.7, 13.4.

MS (EI) m/z: (%) 257 (58, [M^+]), 224 (24), 202 (28), 173 (100), 160 (42), 132 (28), 109 (38), 92 (58).

HRMS (EI) m/z: calcd. for $C_{15}H_{19}N_3O$ 257.1528 [M^+]; found. 257.1532.

3k, synthesized according to the General Procedure C, at **room temperature**

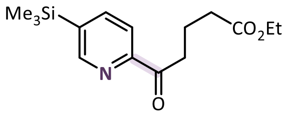

Yield: 73% (0.425 g, 1.45 mmol, 1.99 mmol scale), a yellowish oil

Eluent: cyclohexane/ethyl acetate 15:1 to 10:1 (+ 0.5% NEt_3(v/v))

^{1}H NMR (400 MHz, $CDCl_3$): δ = 8.69 (s, 1H), 7.94–7.87 (m, 2H), 4.08 (q, *J*=7.1 Hz, 2H), 3.23 (t, *J*=7.2 Hz, 2H), 2.38 (t, *J*=7.4 Hz, 2H), 2.02 (p, *J*=7.3 Hz, 2H), 1.20 (t, *J*=7.1 Hz, 3H), 0.28 (s, 9H).

^{13}C NMR (100 MHz, $CDCl_3$): δ = 201.5, 173.3, 153.2, 153.1, 142.1, 140.3, 120.7, 60.3, 36.9, 33.7, 19.3, 14.3, –1.4.

MS (EI) m/z: (%) 293 (10, [M^+]), 248 (27), 206 (57), 192 (20), 151 (100), 73 (35).

HRMS (EI) m/z: calcd. for $C_{15}H_{23}NO_3Si$ 293.1447 [M^+]; found. 293.1454.

3l, synthesized according to the General Procedure C, at **room temperature**

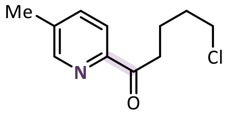

Yield: 70% (0.296 g, 1.40 mmol, 2.00 mmol scale), an orange oil

Eluent: cyclohexane/ethyl acetate 10:1 to 2:1 (+ 0.5% NEt_3(v/v))

^{1}H NMR (400 MHz, $CDCl_3$): δ = 8.44 (d, *J*=1.8 Hz, 1H), 7.90 (d, *J*=8.2 Hz, 1H), 7.58 (dd, *J*=8.0, 1.3 Hz, 1H), 3.57–3.51 (m, 2H), 3.23–3.15 (m, 2H), 2.37 (s, 3H), 1.87–1.80 (m, 4H).

^{13}C NMR (100 MHz, CDCl$_3$): δ = 200.9, 151.0, 149.3, 137.5, 137.1, 121.3, 44.6, 36.5, 32.0, 21.2, 18.6.

MS (EI) m/z: (%) 211 (6, [M$^+$]), 183 (10), 162 (13), 148 (58), 134 (20), 120 (32), 93 (100), 66 (31).

HRMS (EI) m/z: calcd. for $C_{11}H_{14}NO[^{35}Cl]$ 211.0764 [M$^+$]; found. 211.0765.

In reaction with 5-bromopentanoyl chloride gave a complex mixture, likely due to *N*-quaternization with alkyl bromide. Contaminated ketone fraction contained only ≤10% yield.

3m, synthesized according to the General Procedure C, at **10 mmol scale**

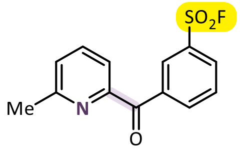

Yield: 62% (1.738 g, 6.22 mmol, 10.01 mmol scale), a yellowish solid,
mp: 88–89.5 °C

Eluent: cyclohexane/ethyl acetate 10:1 to 2:1 (+ 0.5% NEt_3(v/v))

^{1}H NMR (400 MHz, CDCl$_3$): δ = 8.96–8.94 (m, 1H), 8.58–8.55 (m, 1H), 8.23–8.17 (m, 1H), 7.97 (d, *J*=7.8 Hz, 1H), 7.85–7.80 (m, 1H), 7.81–7.72 (m, 1H), 7.41 (d, *J*=7.8 Hz, 1H), 2.63 (s, 3H).

^{13}C NMR (100 MHz, CDCl$_3$): δ = 190.3, 157.9, 152.9, 137.8, 137.8, 137.5, 132.9 (d, *J*=25.2 Hz), 131.5, 131.4, 129.5, 126.8, 121.9, 24.3.

^{19}F NMR (376 MHz, CDCl$_3$): δ = 65.39, 65.34 (resonance of ^{34}S molecule, ca. 5%).

HRMS (ESI) m/z: calcd. for $C_{13}H_{11}NO_3FS$ 280.0444 [M+H]$^+$; found. 280.0443.

3n, synthesized according to the General Procedure C

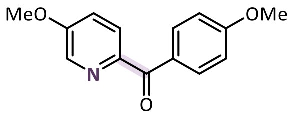

Yield: 86% (0.418 g, 1.72 mmol, 1.99 mmol scale), a white solid,
mp: 119.5–120.5 °C

Eluent: cyclohexane/ethyl acetate 10:1 to 1:1 (+ 0.5% NEt_3(v/v))

^{1}H NMR (400 MHz, CDCl$_3$): δ = 8.37 (dd, *J*=2.9, 0.6 Hz, 1H), 8.15–8.10 (m, 2H), 8.08 (dd, *J*=8.7, 0.6 Hz, 1H), 7.33 (dd, *J*=8.7, 2.9 Hz, 1H), 7.00–6.93 (m, 2H), 3.95 (s, 3H), 3.88 (s, 3H).

^{13}C NMR (100 MHz, CDCl$_3$): δ = 191.4, 163.3, 157.6, 148.3, 136.5, 133.5, 129.6, 126.3, 120.3, 113.4, 55.9, 55.5.

MS (EI) m/z: (%) 243 (52, [M^+]), 214 (40), 185 (7), 135 (100), 107 (9), 92 (14), 77 (18).

HRMS (EI) m/z: calc. for $C_{14}H_{13}NO_3$ 243.0895 [M^+]; found 243.0896.

3o, synthesized according to the General Procedure C

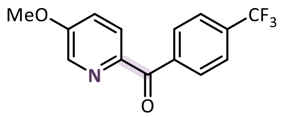

Yield: 75% (0.424 g, 1.51 mmol, 2.00 mmol scale), a white solid,
mp: 48–49.5 °C

Eluent: cyclohexane : ethyl acetate 10:1 to 1:2 (+ 0.5% NEt_3(v/v))

^{1}H NMR (400 MHz, $CDCl_3$): δ = 8.37 (d, *J*=2.9 Hz, 1H), 8.20 (d, *J*=8.8 Hz, 1H), 8.16 (d, *J*=8.0 Hz, 2H), 7.74 (d, *J*=8.1 Hz, 2H), 7.37 (dd, *J*=8.7, 2.9 Hz, 1H), 3.97 (s, 3H).

^{13}C NMR (100 MHz, $CDCl_3$): δ = 191.6, 158.2, 146.7, 140.2 (q, *J*=1.3 Hz), 136.9, 133.5 (q, *J*=32.5 Hz), 131.1, 126.5, 124.9 (q, *J*=3.9 Hz), 123.9 (q, *J*=273 Hz), 120.3, 55.9.

^{19}F NMR (376 MHz, $CDCl_3$): δ = –63.6.

MS (EI) m/z: (%) 281 (56, [M^+]), 252 (100), 223 (20), 212 (20), 173 (57), 145 (83), 108 (20).

HRMS (EI) m/z: calc. for $C_{14}H_{10}F_3NO_2$ 281.0664 [M^+]; found 281.0676.

3p, synthesized according to the General Procedure C, at **120 °C**

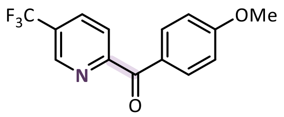

Yield: 66% (0.372 g, 1.32 mmol, 2.01 mmol scale), a yellowish solid,
mp: 98–99 °C

Eluent: cyclohexane/ethyl acetate 10:1 to 3:1 (+ 0.5% NEt_3(v/v))

^{1}H NMR (400 MHz, $CDCl_3$): δ = 8.96 (s, 1H), 8.16–8.07 (m, 4H), 7.00–6.96 (m, 2H), 3.89 (s, 3H).

^{13}C NMR (100 MHz, $CDCl_3$): δ =190.6, 164.0, 158.6 (q, *J*=1.5 Hz), 145.2 (q, *J*=3.9 Hz), 134.3 (q, *J*=3.4 Hz), 133.5, 128.2, 128.0 (d, *J*=33.5 Hz), 124.2, 123.2 (q, *J*=273 Hz), 113.6, 55.4.

^{19}F NMR (376 MHz, $CDCl_3$): δ = –63.1.

MS (EI) m/z: (%) 281 (26, [M^+]), 253 (7), 135 (100), 107 (9), 92 (14), 77 (16).

HRMS (EI) m/z: calc. for $C_{14}H_{10}F_3NO_2$ 281.0664 [M^+]; found 281.0667.

In this reaction a double addition-acylation byproduct **4c** was isolated as a more polar fraction:

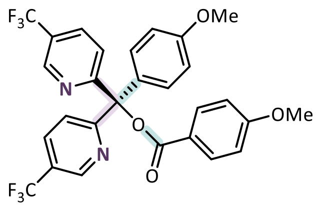

Yield: 20% (0.112 g, 0.20 mmol, 2.01 mmol scale), a yellowish oil

Eluent: cyclohexane/ethyl acetate 10:1 to 3:1 (+ 0.5% NEt_3(v/v))

^{1}H NMR (400 MHz, $CDCl_3$): δ = 8.85–8.80 (m, 2H), 8.19–8.13 (m, 2H), 8.03 (d, *J*=8.4 Hz, 2H), 7.96 (dd, *J*=8.4, 2.4 Hz, 2H), 7.45–7.40 (m, 2H), 7.03–6.97 (m, 2H), 6.92–6.86 (m, 2H), 3.89 (s, 3H), 3.79 (s, 3H).

^{13}C NMR (100 MHz, $CDCl_3$): δ = 164.6, 164.0, 159.3, 145.6 (q, *J*=4.1 Hz), 133.5 (q, *J*=3.4 Hz), 133.3, 132.2, 129.5, 125.1 (q, *J*=33.2 Hz), 123.5 (q, *J*=273 Hz), 123.0, 122.6, 114.0, 113.7, 88.2, 55.6, 55.3.

^{19}F NMR (376 MHz, $CDCl_3$): δ = –62.9.

MS (EI) m/z: (%) 562 (3, [M^+]), 427 (100), 411 (56), 395 (13), 250 (14), 135 (39).

HRMS (EI) m/z: calc. for $C_{28}H_{20}F_6N_2O_4$ 562.1327 [M^+]; found 562.1313.

3q, synthesized according to the General Procedure C, at **120 °C**, stirring for **48 h**

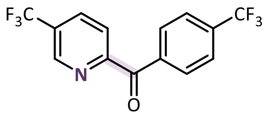

Yield: 46% (0.290 g, 0.91 mmol, 2.00 mmol scale), a yellowish solid,
mp: 62.5–63.5 °C

Eluent: DCM (+ 0.5% NEt_3(v/v))

^{1}H NMR (400 MHz, $CDCl_3$): δ = 8.99 (s, 1H), 8.28–8.15 (m, 4H), 7.76 (d, *J*=8.2 Hz, 2H).

^{13}C NMR (100 MHz, $CDCl_3$): δ = 191.4, 156.9 (q, *J*=1.4 Hz), 145.7 (q, *J*=4.1 Hz), 138.6 (q, *J*=1.4 Hz), 134.8 (q, *J*=3.5 Hz), 134.6 (q, *J*=32.8 Hz), 131.5, 129.2 (q, *J*=33.4 Hz), 125.4 (q, *J*=3.8 Hz), 124.6, 123.7 (q, *J*=273 Hz), 123.1 (q, *J*=273 Hz).

^{19}F NMR (376 MHz, $CDCl_3$): δ = –63.2 (s, 3F), –63.7 (s, 3F).

MS (EI) m/z: (%) 319 (27, [M^+]), 300 (18), 291 (56), 250 (16), 222 (13), 173 (100), 145 (83).

HRMS (EI) m/z: calc. for $C_{14}H_7F_6NO$ 319.0432 [M^+]; found 319.0424.

In this reaction a double addition-acylation byproduct **4d** was isolated as a less polar fraction:

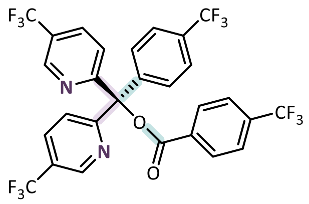

Yield: 28% (0.178 g, 0.28 mmol, 2.00 mmol scale), a yellowish solid, **mp:** 162–163.5 °C

Eluent: DCM (+ 0.5% NEt_3(v/v))

^{1}H NMR (400 MHz, $CDCl_3$): δ = 8.86 (s, 2H), 8.32 (d, *J*=8.1 Hz, 2H), 8.08–7.98 (m, 4H), 7.82 (d, *J*=8.2 Hz, 2H), 7.72–7.62 (m, 4H).

^{13}C NMR (100 MHz, $CDCl_3$): δ = 163.7, 163.0 (q, *J*=1.5 Hz), 146.0 (q, *J*=4.0 Hz), 144.7 (q, *J*=1.4 Hz), 135.4 (q, *J*=32.8 Hz), 134.0 (q, *J*=3.4 Hz), 133.3 (q, *J*=1.2 Hz), 130.7 (q, *J*=32.7 Hz), 130.5, 128.6, 125.93 (q, *J*=3.7 Hz), 125.87 (q, *J*=33.2 Hz), 125.5 (q, *J*=3.7 Hz), 124.0 (q, *J*=272 Hz), 123.7 (q, *J*=273 Hz), 123.4 (q, *J*=272 Hz), 123.1, 88.7.

^{19}F NMR (376 MHz, $CDCl_3$): δ = –63.0 (s, 6F), –63.3 (s, 3F), –63.7 (s, 3F).

MS (EI) m/z: (%) 639 (2, [M+]), 465 (100), 449 (87), 379 (24), 303 (9), 173 (23), 145 (22).

HRMS (EI) m/z: calc. for $C_{28}H_{14}F_{12}N_2O_2$ 638.0864 [M^+]; found 638.0872.

Crystals of **4d** suitable for X-Ray studies were obtained by slow evaporation of its solution in dichloromethane/*n*-heptane (layered), for X-Ray studies details see section 5.

3.4. General procedure D: Fluoride-catalyzed synthesis of ketones

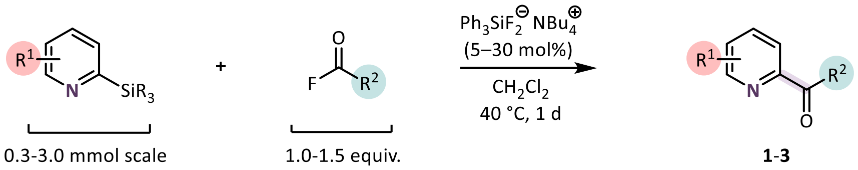

A 20 mL Schlenk flask was charged with corresponding 2-(trialkylsilyl)pyridine (0.3-3.0 mmol), acyl fluoride (1.0 or 1.5 equiv.) and flushed with argon. Then, anhydrous DCM (0.5 or 1.0 mL/mmol) and TBAT (5 or 30 mol%) were added. The flask was sealed, placed in an oil bath (40 °C) and the mixture was stirred for 24 h. Then, the Schlenk flask was taken out of the bath, the mixture was allowed to cool to room temperature and it was directly separated with column chromatography (L=ca. 15-25 cm, ø=3 cm, 100-200 mL of silica gel), and eluted with eluents indicated hereinafter (typically cyclohexane/ethyl acetate).

1a, synthesized according to the General Procedure D, using 2-(trimethylsilyl)pyridine (**1.0 mmol**), acyl fluoride (**1.0 equiv.**), DCM (**0.5 mL**), TBAT (**30 mol%**)

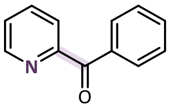

Yield: 77% (0.142 g, 0.78 mmol, 1.01 mmol scale), a yellowish oil

Eluent: cyclohexane, then cyclohexane/ethyl acetate 10:1 to 5:1 (+ 0.5% NEt_3(v/v).

In this reaction a double addition-acylation byproduct **4e** was isolated as a more polar fraction:

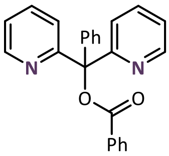

4e

Yield: 22% (0.040 g, 0.11 mmol, 1.01 mmol scale), a white solid, **mp:** 170 °C (dec.)

Eluent: cyclohexane, then cyclohexane/ethyl acetate 10:1 to 5:1 (+ 0.5% NEt_3(v/v))

1e, synthesized according to the General Procedure D, using 2-(trimethylsilyl)pyridine (**1.0 mmol**), acyl fluoride (**1.0 equiv.**), DCM (**0.5 mL**), TBAT (**30 mol%**), stirring at **room temperature**

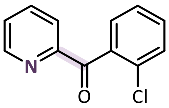

Yield: 89% (0.194 g, 0.89 mmol, 1.00 mmol scale), a white solid

Eluent: cyclohexane, then cyclohexane/ethyl acetate 10:1 to 3:1 (+ 0.5% NEt_3(v/v))

1s, synthesized according to the General Procedure D, using 2-(trimethylsilyl)pyridine (**1.0 mmol**), acyl fluoride (**1.0 equiv.**), DCM (**0.5 mL**), TBAT (**30 mol%**)

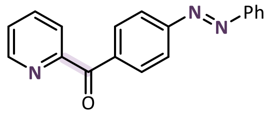

Yield: 63% (0.179 g, 0.62 mmol, 0.99 mmol scale), an orange solid, **mp:** 104–106 °C

Eluent: cyclohexane/ethyl acetate to (+ 0.5% NEt_3(v/v))

^{1}H NMR (400 MHz, $CDCl_3$): δ = 8.75–8.69 (m, 1H), 8.25 (d, *J*=8.5 Hz, 2H), 8.07 (d, *J*=7.9 Hz, 1H), 8.00 (d, *J*=8.5 Hz, 2H), 7.95 (dd, *J*=8.0, 1.8 Hz, 2H), 7.90–7.83 (m, 1H), 7.55–7.42 (m, 4H).

^{13}C NMR (100 MHz, $CDCl_3$): δ = 193.0, 154.8, 154.7, 152.5, 148.6, 137.8, 137.1, 132.1, 131.7, 129.2, 126.4, 124.7, 123.2, 122.4.

MS (EI) m/z: (%) 287 (93, [M$^+$]), 210 (7), 182 (100), 154 (17), 105 (29), 77 (77).

HRMS (EI) m/z: calcd. for $C_{18}H_{13}N_3O$ 287.1059 [M$^+$]; found. 287.1056.

1t, synthesized according to the General Procedure D, using 2-(trimethylsilyl)pyridine (**0.5 mmol**), acyl fluoride (**1.5 equiv.**), DCM (**0.5 mL**), TBAT (**5 mol%**), stirring for **48 h**

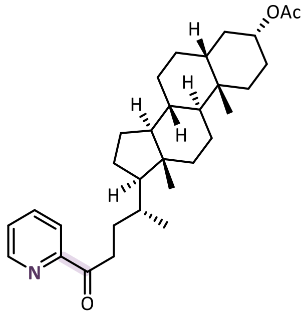

Yield: 76% (0.182 g, 0.38 mmol, 0.50 mmol scale), a white solid, **mp:** 158–159.5 °C

Eluent: cyclohexane/ethyl acetate 10:1 to 3:1 (+ 0.5% NEt_3(v/v))

^{1}H NMR (400 MHz, $CDCl_3$): δ = 8.67–8.63 (m, 1H), 8.02–7.97 (m, 1H), 7.84–7.75 (m, 1H), 7.43 (ddd, *J*=7.5, 4.7, 1.3 Hz, 1H), 4.74–4.62 (m, 1H), 3.26–3.07 (m, 2H), 2.00 (s, 3H), 1.98–1.92 (m, 1H), 1.93–1.73 (m, 5H), 1.69–1.60 (m, 1H), 1.58–1.46 (m, 3H), 1.45–1.31 (m, 7H), 1.31–1.10 (m, 5H), 1.10–0.97 (m, 4H), 0.95 (d, *J*=6.2 Hz, 3H), 0.89 (s, 3H), 0.62 (s, 3H).

^{13}C NMR (100 MHz, $CDCl_3$): δ = 202.7, 170.7, 153.6, 149.0, 136.9, 127.0, 121.8, 74.5, 56.6, 56.1, 42.8, 42.0, 40.5, 40.2, 35.9, 35.6, 35.1, 34.65, 34.61, 32.3, 30.0, 28.3, 27.1, 26.7, 26.4, 24.3, 23.4, 21.6, 20.9, 18.7, 12.1.

MS (EI) m/z: (%) 479 (83, [M$^+$]), 419 (7), 315 (12), 255 (24), 162 (16), 134 (45), 122 (100), 109 (37).

HRMS (EI) m/z: calcd. for $C_{31}H_{45}NO_3$ 479.3399 [M$^+$]; found. 479.3400.

1u, synthesized according to the General Procedure D, using 2-(trimethylsilyl)pyridine (**1.0 mmol**), acyl fluoride (**1.0 equiv.**), DCM (**0.5 mL**), TBAT (**30 mol%**)

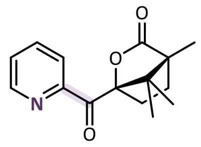

Yield: 92 % (0.226 g, 0.92 mmol, 1.00 mmol scale), a white solid,
mp: 117.5–120 °C

Eluent: toluene, then toluene/ethyl acetate 10:1 to 3:1 (+ 0.5% NEt_3(v/v))

^{1}H NMR (400 MHz, $CDCl_3$): δ = 8.69–8.63 (m, 1H), 7.91 (d, *J*=7.8 Hz, 1H), 7.82–7.76 (m, 1H), 7.42 (ddd, *J*=7.6, 4.7, 1.3 Hz, 1H), 2.76 (ddd, *J*=13.5, 10.8, 4.2 Hz, 1H), 2.49 (ddd, *J*=13.8, 9.4, 4.7 Hz, 1H), 1.96 (ddd, *J*=13.0, 10.8, 4.7 Hz, 1H), 1.76 (ddd, *J*=13.3, 9.4, 4.2 Hz, 1H), 1.12 (s, 3H), 1.09 (s, 3H), 0.94 (s, 3H).

^{13}C NMR (100 MHz, $CDCl_3$): δ = 196.4, 178.7, 153.7, 149.1, 136.8, 126.9, 123.9, 96.3, 55.2, 54.8, 32.2, 29.6, 17.5, 17.0, 9.6.

MS (EI) m/z: (%) 259 (8, [M^+]), 215 (51), 200 (45), 148 (44), 106 (40), 83 (54), 78 (100).

HRMS (EI) m/z: calcd. for $C_{15}H_{17}NO_3$ 259.1208 [M^+]; found. 259.1210.

1v, synthesized according to the General Procedure D, using 2-(trimethylsilyl)pyridine (**1.0 mmol**), acyl fluoride (**1.0 equiv.**), DCM (**0.5 mL**), TBAT (**30 mol%**)

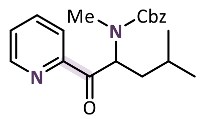

Yield: 70% (0.241 g, 0.71 mmol, 1.01 mmol scale), a colorless oil

Eluent: cyclohexane/ethyl acetate 10:1 to 1:1 (+ 0.5% NEt_3(v/v))

^{1}H NMR (400 MHz, $CDCl_3$): δ = (two rotamers) 8.64 and 8.56 (d, *J*=4.7 Hz, 1H), 7.96 and 7.87 (d, *J*=7.9 Hz, 1H), 7.83–7.73 (m, 1H), 7.46–7.39 (m, 1H), 7.36–7.25 (m, 5H), 6.10–6.00 (m, 1H), 5.12 (s, 2H), 3.00 and 2.98 (s, 3H), 1.90–1.57 (m, 3H), 1.12–0.87 (m, 6H).

^{13}C NMR (100 MHz, $CDCl_3$): δ = (two rotamers) 200.66 and 200.05, 156.72 and 156.42, 152.68 and 152.58, 148.98 and 148.87, 136.90 and 136.71, 136.87 and 136.78, 128.39 and 128.29, 127.81 and 127.71, 127.64 and 127.62, 127.12 and 127.01, 122.44, 67.13 and 67.06, 58.56 and 57.77, 37.13 and 36.99, 31.51 and 31.31, 25.36 and 25.09, 23.43 and 23.39, 21.57 and 21.54.

MS (EI) m/z: (%) 340 (3, [M^+]), 234 (21), 190 (40), 91 (100), 78 (9).

HRMS (EI) m/z: calcd. for $C_{20}H_{24}N_2O_3$ 340.1787 [M^+]; found. 340.1799.

Reaction with *N*-Cbz-Leucine gave complex mixture, with huge amounts of pyridine, suggesting a proto-desilylation pathway.

1w, synthesized according to the General Procedure D, using 2-(trimethylsilyl)pyridine (**1.0 mmol**), acyl fluoride (**1.0 equiv.**), DCM (**1.0 mL**), TBAT (**30 mol%**), stirring for **48 h**

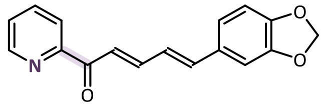

Yield: 70% (0.194 g, 0.69 mmol, 0.99 mmol scale), a white solid,
mp: 166 °C – dec.

Eluent: cyclohexane, then cyclohexane/ethyl acetate 10:1 to 1:1 (+ 0.5% NEt_3(v/v)); after column chromatography the product was crystallized from DCM/n-heptane to remove contamination

^{1}H NMR (400 MHz, $CDCl_3$): δ = 8.70 (ddd, *J*=4.8, 1.8, 0.9 Hz, 1H), 8.14 (d, *J*=7.8 Hz, 1H), 7.87–7.81 (m, 1H), 7.78–7.63 (m, 2H), 7.45 (ddd, *J*=7.6, 4.8, 1.3 Hz, 1H), 7.02 (d, *J*=1.7 Hz, 1H), 6.98–6.86 (m, 3H), 6.79 (d, *J*=8.0 Hz, 1H), 5.98 (s, 2H).

^{13}C NMR (100 MHz, $CDCl_3$): δ = 189.6, 154.5, 148.9, 148.8, 148.4, 145.2, 142.1, 137.1, 130.9, 126.8, 125.9, 123.8, 123.4, 122.9, 108.7, 106.0, 101.5.

MS (EI) m/z: (%) 279 (33, [M^+]), 250 (31), 201 (15), 173 (27), 143 (21), 115 (50), 107 (30), 79 (100).

HRMS (EI) m/z: calcd. for $C_{17}H_{13}NO_3$ 279.0895 [M^+]; found. 279.0891.

1x, synthesized according to the General Procedure D, using 2-(trimethylsilyl)pyridine (**1.0 mmol**), acyl fluoride (**1.0 equiv.**), DCM (**0.5 mL**), TBAT (**30 mol%**)

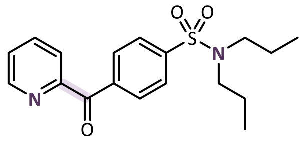

Yield: 61% (0.214 g, 0.62 mmol, 1.01 mmol scale), a white solid,
mp: 73–75.5 °C

Eluent: cyclohexane/ethyl acetate 10:1 to 2:1 (+ 0.5% NEt_3(v/v))

^{1}H NMR (400 MHz, $CDCl_3$): δ = 8.69 (dd, *J*=4.8, 1.6 Hz, 1H), 8.19 (d, *J*=8.3 Hz, 2H), 8.08 (dd, *J*=7.9, 1.3 Hz, 1H), 7.94–7.84 (m, 3H), 7.51 (dd, *J*=7.7, 4.6 Hz, 1H), 3.10–3.04 (m, 4H), 1.61–1.48 (m, 4H), 0.85 (t, *J*=7.4 Hz, 6H).

^{13}C NMR (100 MHz, $CDCl_3$): δ = 192.4, 154.1, 148.6, 143.5, 139.3, 137.4, 131.6, 126.9, 126.7, 124.8, 50.3, 22.2, 11.2.

HRMS (ESI) m/z: calcd. for $C_{18}H_{22}N_2O_3NaS$ 369.1249 [$M+Na^+$]; found. 369.1250.

1y, synthesized according to the modified procedure, using 2-(trimethylsilyl)pyridine (**0.3 mmol**), acyl fluoride (**1.5 equiv.**), anhydrous **DMSO** (**0.5 mL**), TBAT (**30 mol%**), stirring at **120 °C**.

After 24 h the mixture was cooled to rt, transferred into separatory funnel where an aqueous solution of $NaHCO_3$ (50 mL, 5%) was added and it was extracted with ethyl acetate (3×50 mL). Combined organic layers were washed with water (50 mL), brine (50 mL), and dried over anhydrous $MgSO_4$. The mixture was filtered, evaporated, and the product was separated by column chromatography.

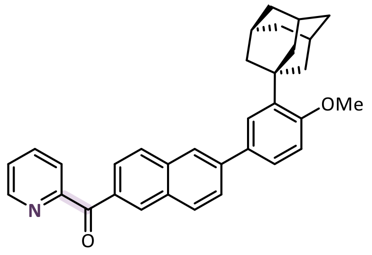

Yield: 70 % (0.100 g, 0.21 mmol, 0.30 mmol scale), a white solid,
mp: 189–191.5 °C

Eluent: cyclohexane/ethyl acetate 20:1 to 6:1 (+ 0.5% NEt_3(v/v))

^{1}H NMR (400 MHz, $CDCl_3$): δ = 8.79 (d, *J*=4.8 Hz, 1H), 8.67 (s, 1H), 8.17 (d, *J*=8.6 Hz, 1H), 8.09 (d, *J*=7.9 Hz, 1H), 8.04 (s, 1H), 8.02–7.96 (m, 2H), 7.96–7.89 (m, 1H), 7.80 (dd, *J*=8.5, 1.8 Hz, 1H), 7.64 (s, 1H), 7.56 (dd, *J*=8.4, 2.3 Hz, 1H), 7.54–7.48 (m, 1H), 7.00 (d, *J*=8.4 Hz, 1H), 3.90 (s, 3H), 2.21 (s, 6H), 2.16–2.08 (m, 3H), 1.82 (s, 6H).

^{13}C NMR (100 MHz, $CDCl_3$): δ = 193.7, 159.0, 155.6, 148.7, 141.7, 139.0, 137.2, 136.1, 133.5, 133.1, 132.6, 131.2, 130.3, 128.2, 126.6, 126.4, 126.2, 126.0, 125.8, 124.8, 124.7, 112.2, 55.2, 40.7, 37.3, 37.2, 29.2.

HRMS (ESI) m/z: calcd. for $C_{33}H_{31}NO_2Na$ 496.2252 [M+Na$^+$]; found. 496.2253.

1z, synthesized according to the General Procedure D, using 2-(trimethylsilyl)pyridine (**1.0 mmol**), acyl fluoride (**1.0 equiv.**), DCM (**1.0 mL**), TBAT (**30 mol%**)

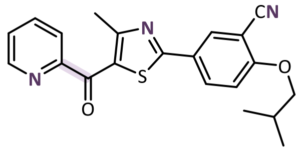

Yield: 80% (0.301 g, 0.80 mmol, 1.00 mmol scale), a white solid,
mp: 172–175.5 °C

Eluent: cyclohexane, then cyclohexane/ethyl acetate 10:1 to 2:1 (+ 0.5% NEt_3(v/v))

^{1}H NMR (400 MHz, $CDCl_3$): δ = 8.71 (d, *J*=4.5 Hz, 1H), 8.26–8.12 (m, 3H), 7.92–7.84 (m, 1H), 7.50 (dd, *J*=7.6, 4.7 Hz, 1H), 6.97 (d, *J*=8.9 Hz, 1H), 3.87 (d, *J*=6.5 Hz, 2H), 2.87 (s, 3H), 2.26–2.09 (m, 1H), 1.07 (d, *J*=6.7 Hz, 6H).

^{13}C NMR (100 MHz, $CDCl_3$): δ = 183.9, 170.9, 165.1, 162.5, 153.5, 147.8, 137.5, 132.7, 132.2, 127.1, 126.4, 123.8, 123.3, 115.6, 112.6, 102.8, 75.7, 28.2, 19.9, 19.1.

MS (EI) m/z: (%) 377 (100, [M$^+$]), 321 (55), 293 (68), 260 (34), 243 (14), 177 (10), 149 (29), 106 (11), 78 (31), 71 (21).

HRMS (EI) m/z: calcd. for $C_{21}H_{19}N_3O_2S$ 377.1198 [M$^+$]; found. 377.1204.

2m, synthesized according to the General Procedure D, using 2-(triethylsilyl)nicotine (**1.0 mmol**), acyl fluoride (**1.0 equiv.**), TBAT (**5 mol%**), stirring in **neat** at **60 °C**

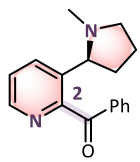

Yield: 80% (0.216 g, 0.81 mmol, 1.01 mmol scale), a colorless oil

Eluent: cyclohexane/ethyl acetate 10:1 to 2:1 (+ 0.5% NEt_3(v/v))

^{1}H NMR (400 MHz, $CDCl_3$): δ = 8.50 (dd, *J*=4.7, 1.6 Hz, 1H), 8.00 (dd, *J*=8.0, 1.6 Hz, 1H), 7.79–7.74 (m, 2H), 7.55–7.48 (m, 1H), 7.43–7.34 (m, 3H), 3.30 (t, *J*=8.1 Hz, 1H), 3.06–2.98 (m, 1H), 2.28–2.13 (m, 2H), 2.01 (s, 3H), 1.88–1.73 (m, 1H), 1.74–1.62 (m, 2H).

^{13}C NMR (100 MHz, $CDCl_3$): δ = 194.5, 155.7, 147.1, 139.3, 136.8, 136.0, 133.1, 129.9, 128.4, 124.8, 66.5, 56.5, 40.7, 35.7, 23.1.

MS (EI) m/z: (%) 266 (100, [M^+]), 251 (41), 222 (12), 159 (14), 85 (24), 77 (23).

HRMS (EI) m/z: calcd. for $C_{17}H_{18}N_2O$ 266.1419 [M^+]; found 266.1423.

Analogous reaction with benzoyl chloride (neat, 80 °C, 24 h) gave a complex mixture, containing only traces of **2m**.

3r, synthesized according to the General Procedure D, using 1-(triethylsilyl)-5-(dimethylamino)--isoquinoline (**1.0 mmol**), acyl fluoride (**1.5 equiv.**), DCM (**0.5 mL**), TBAT (**30 mol%**)

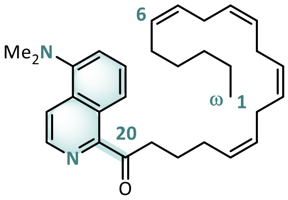

Yield: 69% (0.318 g, 0.69 mmol, 1.00 mmol scale), a colorless oil

Eluent: DCM, then DCM/ethyl acetate 100:1 to 10:1 (+ 0.5% NEt_3(v/v))

^{1}H NMR (400 MHz, $CDCl_3$): δ = 8.54 (d, *J*=6.0 Hz, 1H), 8.40 (d, *J*=8.6 Hz, 1H), 8.17 (d, *J*=5.8 Hz, 1H), 7.59–7.49 (m, 1H), 7.24 (d, *J*=7.6 Hz, 1H), 5.51–5.27 (m, 8H), 3.32 (t, *J*=7.4 Hz, 2H), 2.88 (s, 6H), 2.88–2.77 (m, 5H), 2.27–2.17 (m, 2H), 2.08–1.99 (m, 2H), 1.92–1.81 (m, 2H), 1.40–1.22 (m, 6H), 0.87 (t, *J*=6.7 Hz, 3H).

^{13}C NMR (100 MHz, $CDCl_3$): δ = 204.9, 154.2, 150.3, 140.5, 133.1, 130.5, 129.6, 128.8, 128.65, 128.61, 128.4, 128.2, 128.0, 127.7, 127.0, 120.7, 117.8, 45.2, 40.0, 31.6, 29.4, 27.3, 26.9, 25.76, 25.74, 25.72, 24.2, 22.7, 14.2.

HRMS (ESI) m/z: calcd. for $C_{31}H_{42}N_2ONa$ 481.3195 [$M+Na^+$]; found. 481.3200.

3s, synthesized according to the General Procedure D, using 2-(triethylsilyl)nicotine (**1.0 mmol**), acyl fluoride (**1.5 equiv.**), TBAT (**5 mol%**), stirring in **neat** at **60 °C**

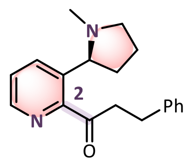

Yield: 83% (0.242 g, 0.82 mmol, 0.99 mmol scale), a yellowish oil

Eluent: cyclohexane/ethyl acetate 6:1 to 4:1 (+ 0.5% NEt_3(v/v))

^{1}H NMR (400 MHz, $CDCl_3$): δ = 8.48 (dd, *J*=4.6, 1.7 Hz, 1H), 8.18 (dd, *J*=8.0, 1.7 Hz, 1H), 7.37 (dd, *J*=8.0, 4.6 Hz, 1H), 7.30–7.21 (m, 4H), 7.21–7.11 (m, 1H), 3.73 (t, *J*=8.2 Hz, 1H), 3.65–3.53 (m, 1H), 3.48–3.35 (m, 1H), 3.17 (ddd, *J*=9.4, 7.5, 2.2 Hz, 1H), 3.05 (t, *J*=7.7 Hz, 2H), 2.53–2.40 (m, 1H), 2.37–2.25 (m, 1H), 2.10 (s, 3H), 1.92–1.73 (m, 2H), 1.60–1.46 (m, 1H).

^{13}C NMR (100 MHz, $CDCl_3$): δ = 203.3, 152.9, 146.6, 141.4, 140.2, 136.2, 128.43, 128.36, 126.3, 125.9, 65.7, 56.9, 41.8, 40.7, 35.2, 30.0, 23.0.

MS (EI) m/z: (%) 294 (35, [M^+]), 203 (100), 160 (8), 146 (5), 91 (8), 84 (6).

HRMS (EI) m/z: calcd. for $C_{19}H_{22}N_2O$ 294.1732 [M^+]; found. 294.1741.

3t, synthesized according to the modified procedure, using 2-(triethylsilyl)nicotine (**0.5 mmol**), acyl fluoride (**1.0 equiv.**), anhydrous **DMSO** (**0.5 mL**), TBAT (**5 mol%**), stirring at **60 °C**.

After 24 h the mixture was cooled to rt, transferred into separatory funnel where an aqueous solution of $NaHCO_3$ (50 mL, 5%) was added and it was extracted with ethyl acetate (3×50 mL). Combined organic layers were washed with water (50 mL), brine (50 mL), and dried over anhydrous $MgSO_4$. The mixture was filtered, evaporated, and the product was separated by column chromatography.

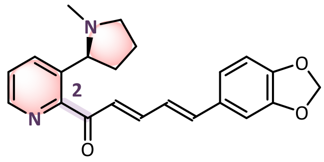

Yield: 58% (0.105 g, 0.29 mmol, 0.50 mmol scale), an orange oil

Eluent: cyclohexane/ethyl acetate 6:1 to 1:1 (+ 0.5% NEt_3(v/v))

^{1}H NMR (400 MHz, $CDCl_3$): δ = 8.54 (dd, *J*=4.6, 1.7 Hz, 1H), 8.18 (dd, *J*=8.0, 1.7 Hz, 1H), 7.46–7.37 (m, 2H), 7.19 (d, *J*=15.3 Hz, 1H), 7.01 (d, *J*=1.7 Hz, 1H), 6.93 (dd, *J*=8.0, 1.7 Hz, 1H), 6.89–6.84 (m, 2H), 6.79 (d, *J*=8.0 Hz, 1H), 5.99 (s, 2H), 3.74–3.65 (m, 1H), 3.24–3.16 (m, 1H), 2.54–2.42 (m, 1H), 2.39–2.27 (m, 1H), 2.14 (s, 3H), 1.95–1.77 (m, 2H), 1.66–1.56 (m, 1H).

^{13}C NMR (100 MHz, $CDCl_3$): δ = 192.7, 154.2, 148.8, 148.4, 146.8, 145.3, 141.6, 140.4, 136.3, 130.8, 127.7, 125.9, 125.6, 123.3, 108.6, 106.0, 101.5, 65.9, 57.0, 40.7, 35.5, 23.0.

MS (EI) m/z: (%) 362 (21, [M^+]), 227 (100), 201 (9), 159 (36), 115 (9), 85 (8).

HRMS (EI) m/z: calcd. for $C_{22}H_{22}N_2O_3$ 362.1630 [M^+]; found. 362.1636.

3u, synthesized according to the General Procedure D, using 6-(triethylsilyl)nicotine (**3.0 mmol**), acyl fluoride (**1.0 equiv.**), DCM (**3.0 mL**), TBAT (**30 mol%**)

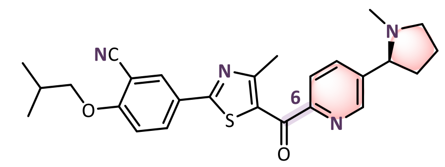

Yield: 80% (1.110 g, 2.41 mmol, 3.00 mmol scale), a yellow solid, **mp:** 126.5–132 °C

Eluent: cyclohexane/ethyl acetate 10:1 to 1:2 (+ 0.5% NEt_3(v/v))

^{1}H NMR (400 MHz, $CDCl_3$): δ = 8.56 (d, *J*=2.1 Hz, 1H), 8.10–7.96 (m, 3H), 7.78 (dd, *J*=8.1, 2.1 Hz, 1H), 6.85 (d, *J*=8.9 Hz, 1H), 3.76 (d, *J*=6.5 Hz, 2H), 3.22–3.09 (m, 2H), 2.74 (s, 3H), 2.32–2.21 (m, 1H), 2.21–2.13 (m, 1H), 2.11 (s, 3H), 2.09–2.00 (m, 1H), 1.95–1.80 (m, 1H), 1.81–1.69 (m, 1H), 1.69–1.57 (m, 1H), 0.98 (d, *J*=6.9 Hz, 6H).

^{13}C NMR (100 MHz, $CDCl_3$): δ = 183.3, 170.3, 164.5, 162.1, 152.2, 147.2, 143.3, 136.0, 132.3, 131.7, 126.1, 123.6, 123.0, 115.3, 112.3, 102.5, 75.4, 68.4, 56.9, 40.4, 35.3, 28.0, 22.8, 19.7, 18.9.

HRMS (ESI) m/z: calcd. for $C_{26}H_{29}N_4O_2S$ 461.2011 [M^+]; found 461.2013.

3.5. Orthogonal transformations of 3m: Synthesis of 4a,b

SuFEx-type synthesis of sulfonamide

Transformation of **3m** into sulfonamide **4a** was performed according to the modified procedure reported earlier by us (*Synthesis* **2025**, *57*, 1885):

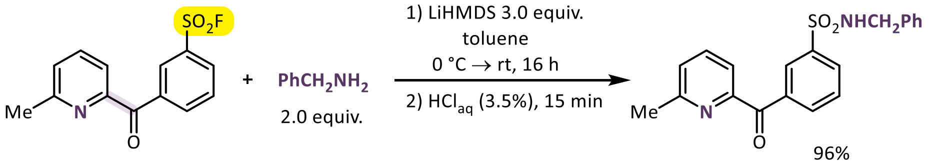

A 30 mL Schlenk flask was charged with **3m** (0.140 g, 0.50 mmol) and flushed with argon. Then, a solution of benzylamine (0.109 g, 1.01 mmol, 2.0 equiv.) in anhydrous toluene (2 mL) was added and the flask was placed in an ice-water bath. Then, 1.0 M solution of LiHMDS in THF (1.5 mL, 1.5 mmol, 3.0 equiv.) was added dropwise over 10 min with vigorous stirring. After 5 min, the ice-water bath was removed and the mixture was stirred at rt. After 16 h the mixture was quenched with an aqueous solution of HCl (3 mL, ca. 3.5%) and stirred at 1400 rpm for 15 min. Then, the mixture was transferred into separatory funnel, where an aqueous solution of $NaHCO_3$ (50 mL, 5%) was added and it was extracted with ethyl acetate (3×50 mL). Combined organic layers were washed with water (50 mL), brine (50 mL), and dried over anhydrous $MgSO_4$. The mixture was filtered, evaporated, and the product was separated by column chromatography.

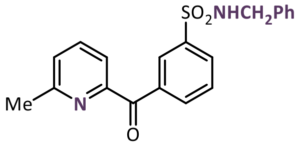

Yield: 96% (0.177 g, 0.48 mmol, 0.50 mmol scale), a white solid, **mp:** 107–110 °C

Eluent: cyclohexane/ethyl acetate 6:1 to 2:1

^{1}H NMR (400 MHz, $CDCl_3$): δ = 8.63 (d, *J*=1.9 Hz, 1H), 8.28 (dd, *J*=7.8, 1.6 Hz, 1H), 8.01 (dd, *J*=8.0, 1.8 Hz, 1H), 7.82 (d, *J*=7.7 Hz, 1H), 7.78–7.71 (m, 1H), 7.60–7.53 (m, 1H), 7.33 (d, *J*=7.7 Hz, 1H), 7.24–7.12 (m, 5H), 5.54 (t, *J*=6.1 Hz, 1H), 4.16 (d, *J*=6.1 Hz, 2H), 2.56 (s, 3H).

^{13}C NMR (100 MHz, $CDCl_3$): δ = 192.0, 157.8, 153.4, 140.0, 137.4, 137.1, 136.1, 134.9, 130.6, 130.1, 129.0, 128.6, 127.9, 127.8, 126.5, 121.9, 47.3, 24.4.

HRMS (ESI) m/z: calcd. for $C_{20}H_{18}N_2O_3SNa$ 389.0936 [M+Na$^+$]; found 389.0934.

Note: When original conditions (1.0 equiv. of amine, 2.1 equiv. of LiHMDS) were applied, we observed low conversion of substrate (ca. 30%), likely due to preferred formation of anionic hemiaminal adduct with the carbonyl group (which hydrolized during aqueous workup, however). Use of two equivalents of benzylamine was sufficient to afford high yield of the main SuFEx reaction.

I_2-Mediated synthesis of imidazo[1,5-*a*]pyridine

Transformation of **3m** into imidazo[1,5-*a*]pyridine **4b** was performed according to the modified procedure reported in the literature: *Org. Biomol. Chem.* **2018**, *16*, 5653:

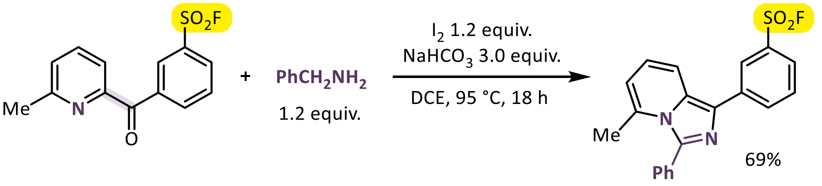

A 30 mL Schlenk flask was charged with **3m** (0.140 g, 0.50 mmol) and flushed with argon. Then, a solution of benzylamine (0.064 g, 0.60 mmol, 1.2 equiv.) in anhydrous DCE (2 mL) was added. Then, a solid I_2 (0.153 g, 0.60 mmol, 1.2 equiv.) and $NaHCO_3$ (0.127 g, 1.51 mmol, 3.0 equiv.) were added. The flask was sealed and placed in an oil bath (95 °C). After 18 h the mixture was cooled to room temperature, and quenched with an aqueous solution of $Na_2S_2O_3$ (10 mL, 5%). Then, the mixture was transferred into separatory funnel, where an aqueous solution of $NaHCO_3$ (40 mL, 5%) was added and it was extracted with ethyl acetate (3×50 mL). Combined organic layers were washed with water (50 mL), brine (50 mL), and dried over anhydrous $MgSO_4$. The mixture was filtered, evaporated, and the product was separated by column chromatography.

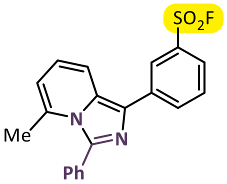

Yield: 69% (0.128 g, 0.35 mmol, 0.51 mmol scale), a yellowish solid,
mp: 179.5–182 °C

Eluent: toluene, then toluene/ethyl acetate 20:1 to 10:1

^{1}H NMR (400 MHz, $CDCl_3$): δ = 8.56–8.53 (m, 1H), 8.33 (d, *J*=8.0 Hz, 1H), 7.85 (d, *J*=8.0 Hz, 1H), 7.76 (d, *J*=9.2 Hz, 1H), 7.69–7.62 (m, 1H), 7.59–7.53 (m, 2H), 7.53–7.41 (m, 3H), 6.87 (dd, *J*=9.2, 6.5 Hz, 1H), 6.40 (d, *J*=6.6 Hz, 1H), 2.13 (s, 3H).

^{13}C NMR (100 MHz, $CDCl_3$): δ = 139.8, 137.4, 134.2, 133.5 (d, *J*=23.7 Hz), 133.4, 133.3, 131.1, 130.0, 129.7, 129.4, 127.8, 127.7, 126.0, 125.4, 122.0, 116.0, 114.6, 22.2.

^{19}F NMR (376 MHz, $CDCl_3$): δ = 65.26, 65.21 (resonance of ^{34}S molecule, ca. 5%).

MS (EI) m/z: (%) 366 (100, [M$^+$]), 283 (4), 180 (21), 152 (5), 120 (4).

HRMS (EI) m/z: calcd. for $C_{20}H_{15}N_2O_2FS$ 366.0838 [M$^+$]; found. 366.0841.

4. Mechanistic studies

4.1. Effect of acyl and silyl substituents

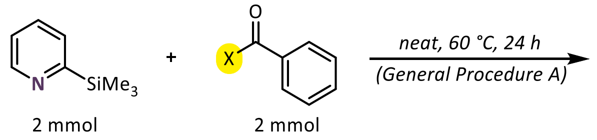

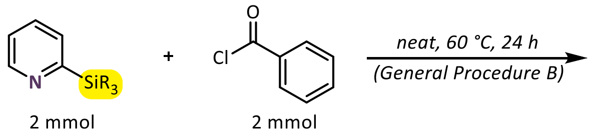

X=	Conversion	Isolated yield	R$_3$=	Conversion	Isolated yield
**Cl**	100%	**88%**	**Me$_3$**	**100%**	**88%**
**F**	ca. 10% (60 °C)<br>ca. 60% (120 °C)	-	**Et$_3$**	ca. 80% (60 °C)<br>100% (80 °C)	**86%**<br>(80 °C)
**Br**	100%	**78%**	***t*BuMe$_2$**	<1% (60 °C)<br>ca. 40% (120 °C, 24 h)<br>ca. 70% (120 °C, 48 h)<br>100% (120 °C, 72 h)	**36%**<br>(120 °C, 72 h)
**OMe**	0% (60 °C)<br>0% (120 °C)	-			
**CN**	0% (60 °C)<br>0% (120 °C)	-	***i*Pr$_3$**	0% (60 °C)<br>ca. 20% (120 °C)	-

4.2. NMR studies of the reaction course

In model reaction of 2-TMS-Py with benzoyl chloride in a CD_3CN solution at 60 °C we observed direct formation of ketone **1a**, without detectable intermediates on ^1H NMR spectrum:

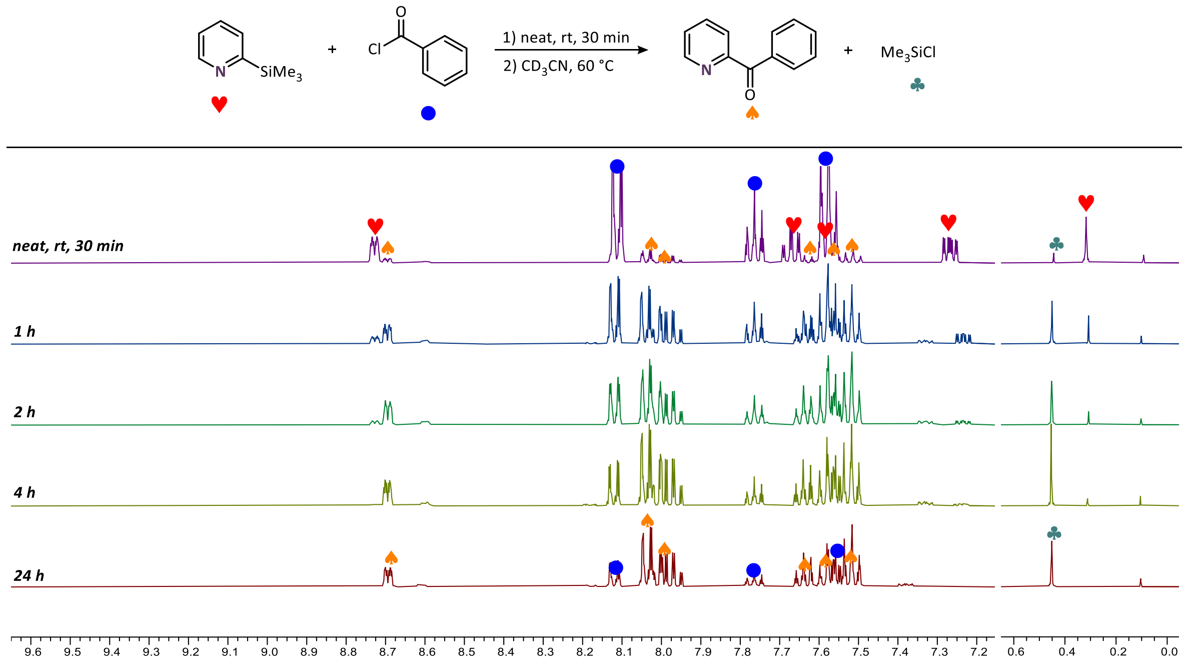

^1H NMR spectra after 1, 2, 4 and 24 h of heating of substrates at 60 °C in CD_3CN NMR tube.

When 2-(trimethylsilyl)pyridine (1 mmol) was mixed with benzoyl bromide (1 mmol) in neat at room temperature, we observed immediate formation of a yellowish solid (assigned by NMR, as an *N*-acylated intermediate (*N*-acylated-2-TMS-Py) :

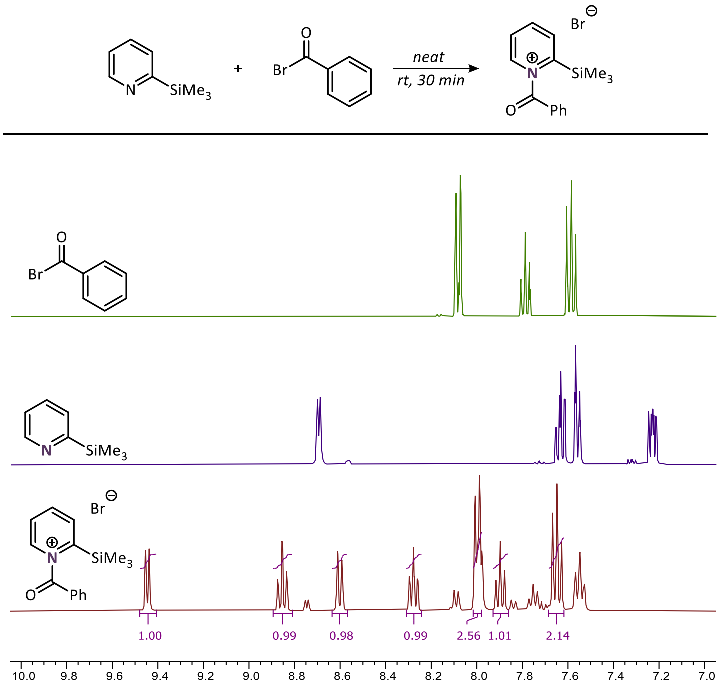

Stack of ^1H NMR spectra of substrates and N-acylated intermediate recorded in CD_3CN.
1 mL of CD_3CN was added to the Schlenk flask after 30 min and the solution was transferred to NMR tube.

After dissolution of the solid in CD_3CN and heating at 60 °C again we observed a direct formation of ketone **1a**:

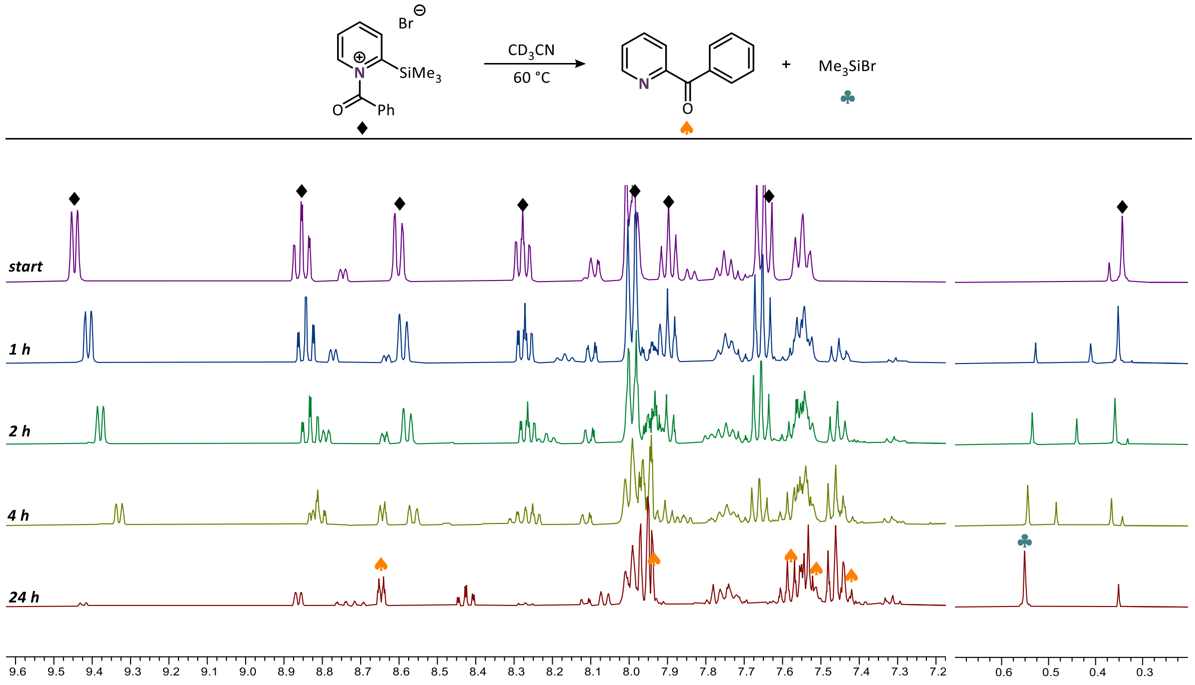

^{1}H NMR spectra after 1, 2, 4 and 24 h of heating of N-acylated intermediate at 60 °C in CD_3CN NMR tube.

In reaction of 2-TMS-Py with benzoyl bromide we tested also different reagents ratios from 1:2 to 2:1:

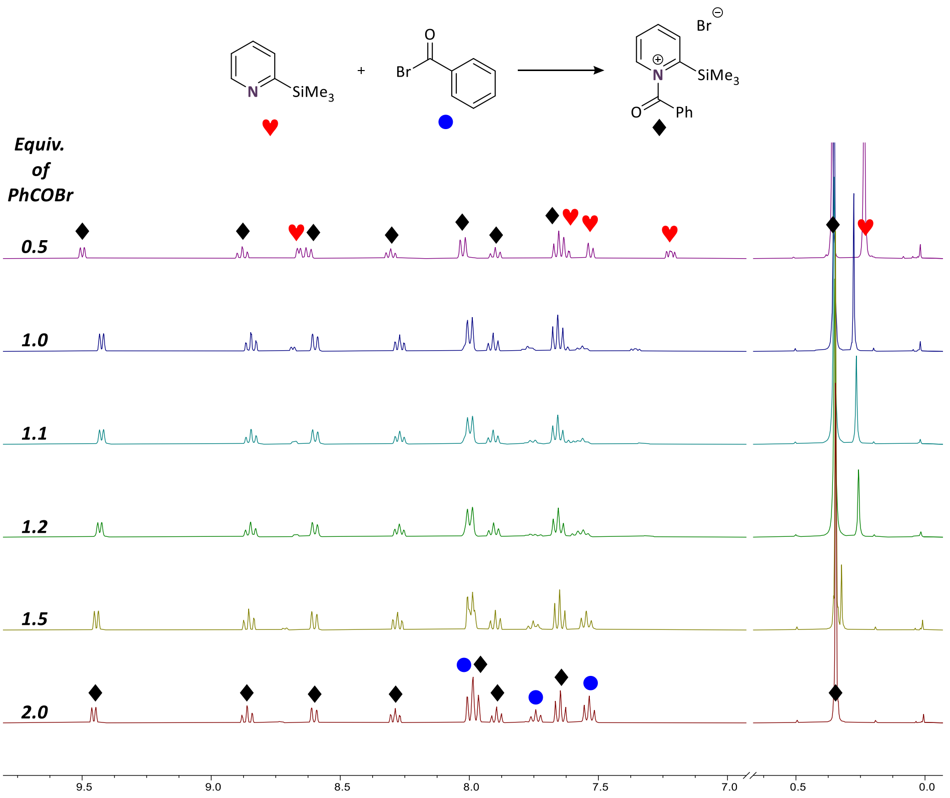

^{1}H NMR spectra (CD_3CN) for different reagent ratios.

^{1}H NMR (400 MHz, CD_3CN):

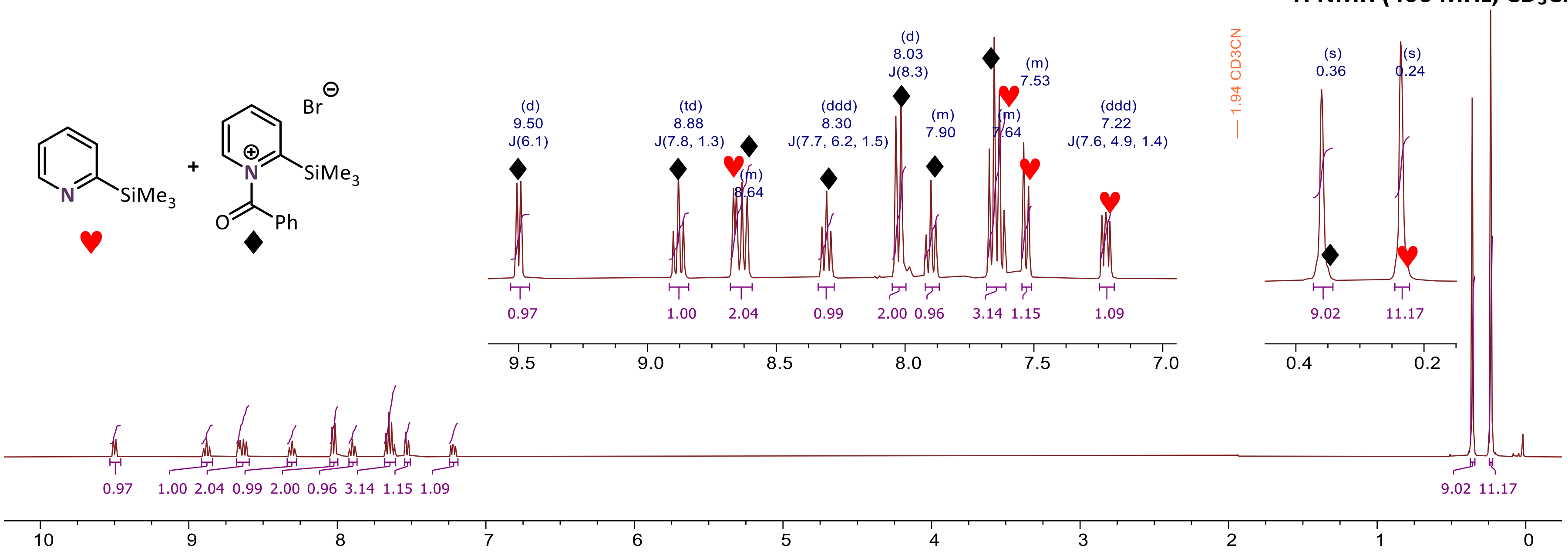

^{13}C NMR (100 MHz, CD_3CN):

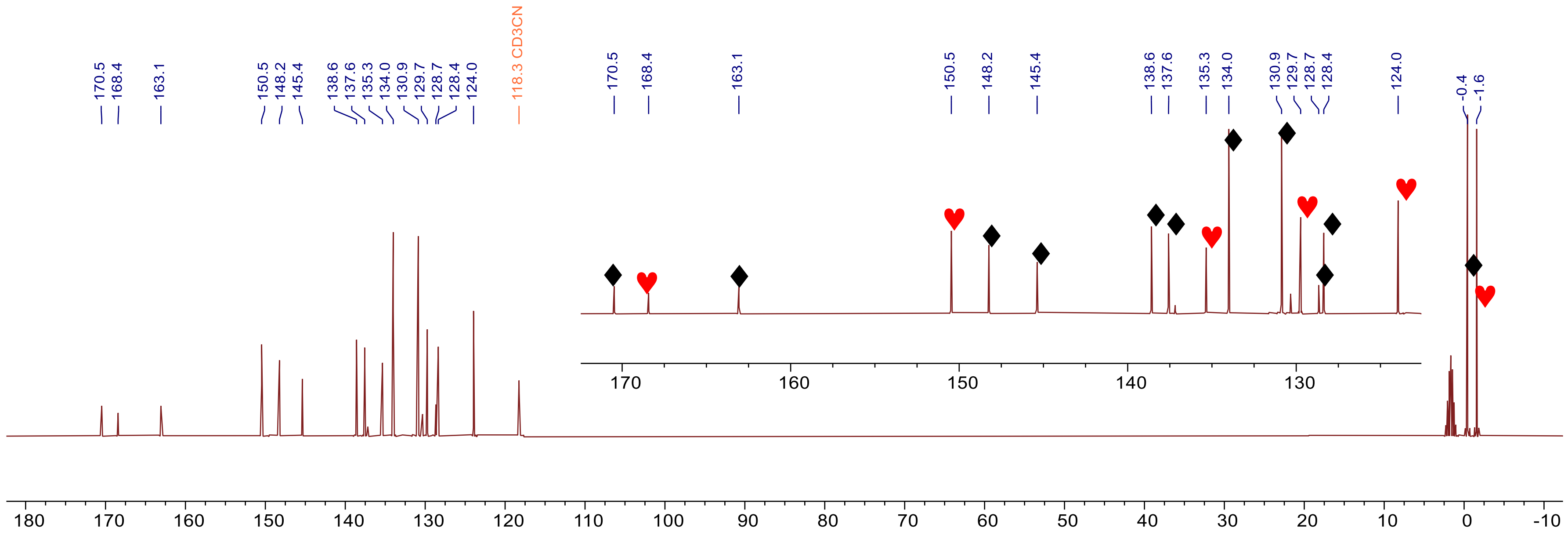

^{1}H NMR (400 MHz, CD_3CN):

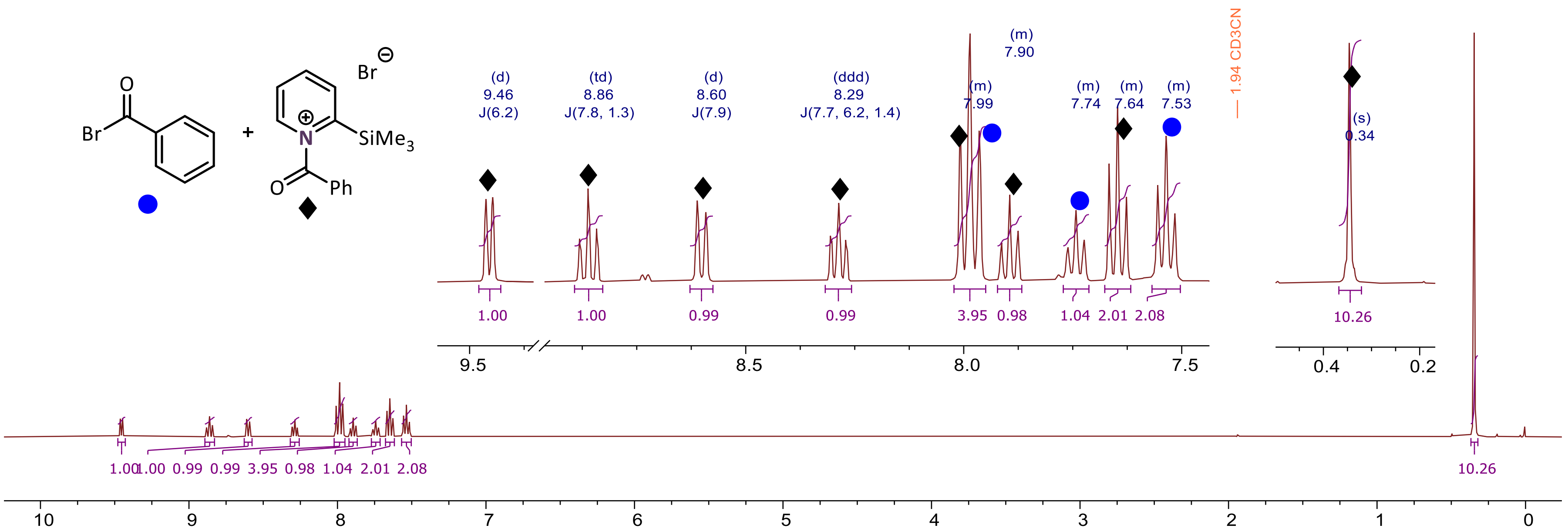

^{13}C NMR (100 MHz, CD_3CN):

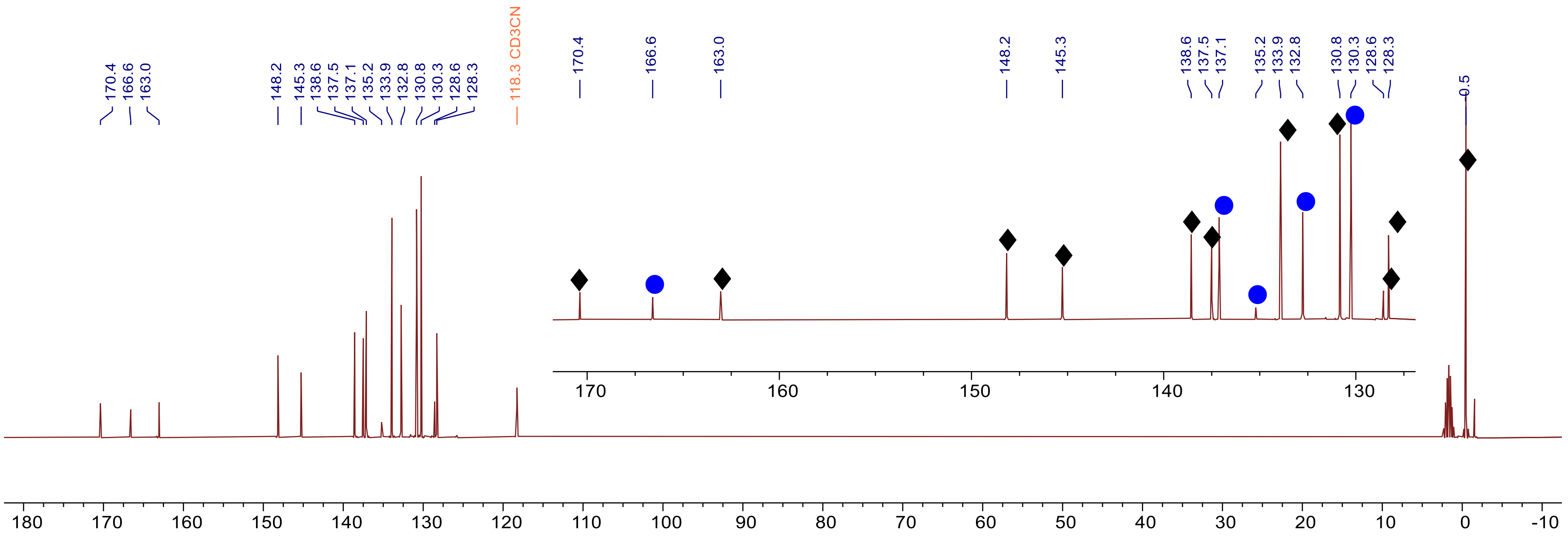

The experiments allowed to characterize *N*-acylated intermediate by NMR:

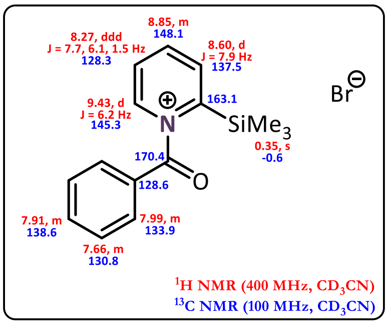

^{1}H NMR (400 MHz, CD_3CN): δ = 9.43 (d, *J*=6.2 Hz, 1H), 8.88–8.82 (m, 1H), 8.60 (d, *J*=7.9 Hz, 1H), 8.27 (ddd, *J*=7.7, 6.1, 1.5 Hz, 1H), 8.05–7.96 (m, 2H), 7.95–7.86 (m, 1H), 7.70–7.61 (m, 2H), 0.35 (s, 9H).

^{13}C NMR (100 MHz, $CDCl_3$): δ = 170.4, 163.1, 148.1, 145.3, 138.6, 137.5, 133.9, 130.8, 128.6, 128.3, -0.6.

For the 1.1 equiv. PhCOBr sample we performed ^{1}H, ^{13}C, COSY, HSQCAD, and HMBCAD experiments:

^{1}H NMR (400 MHz, CD_3CN):

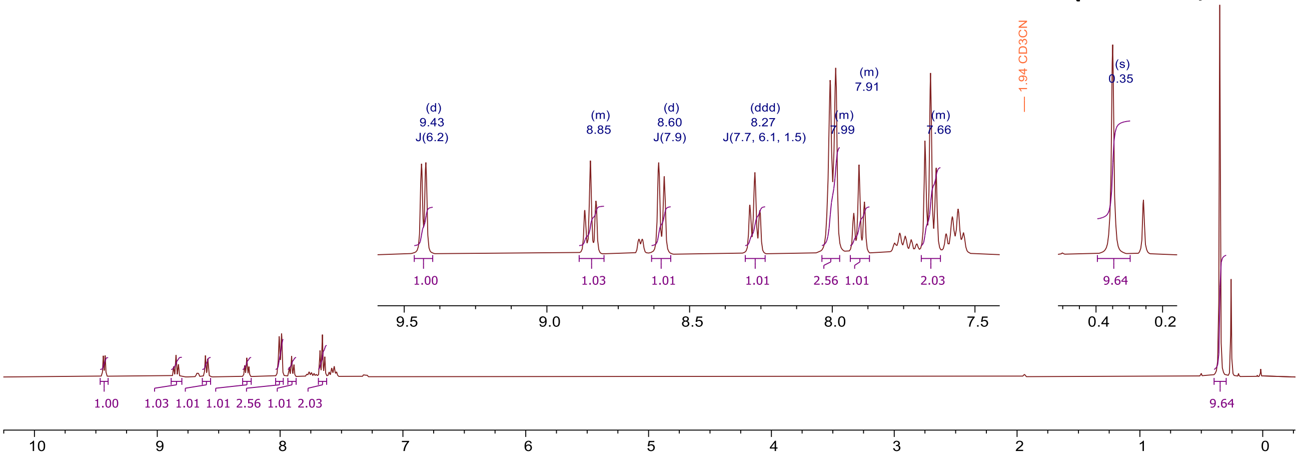

COSY:

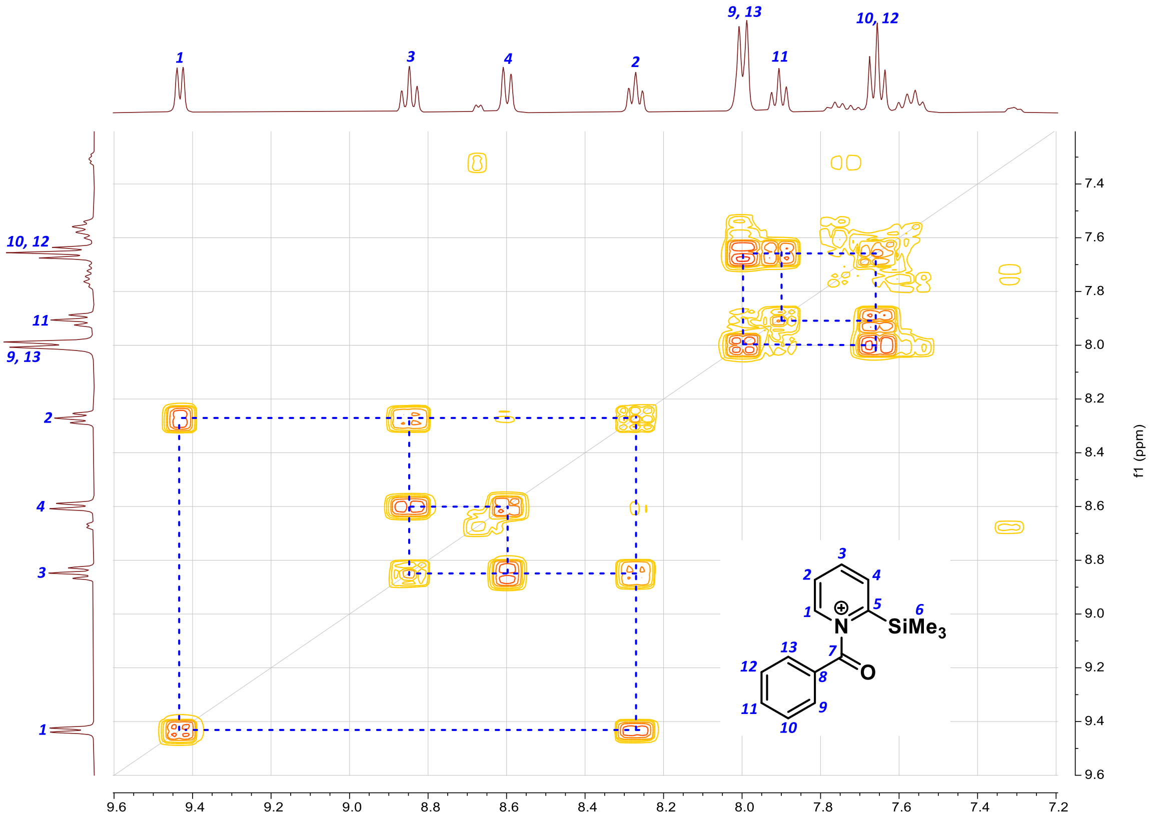

^{13}C NMR (100 MHz, CD_3CN):

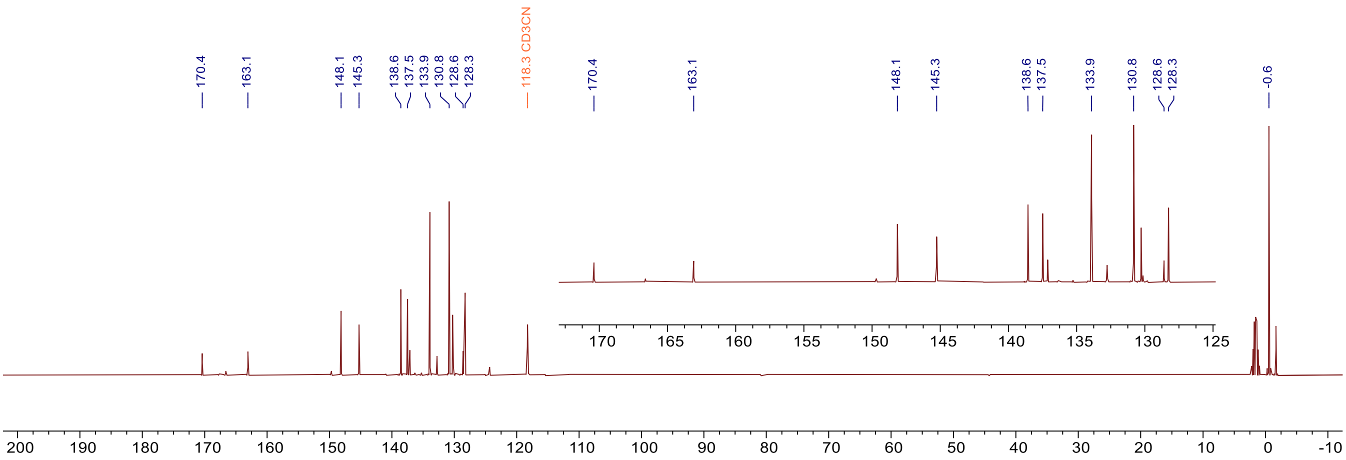

HSQCAD:

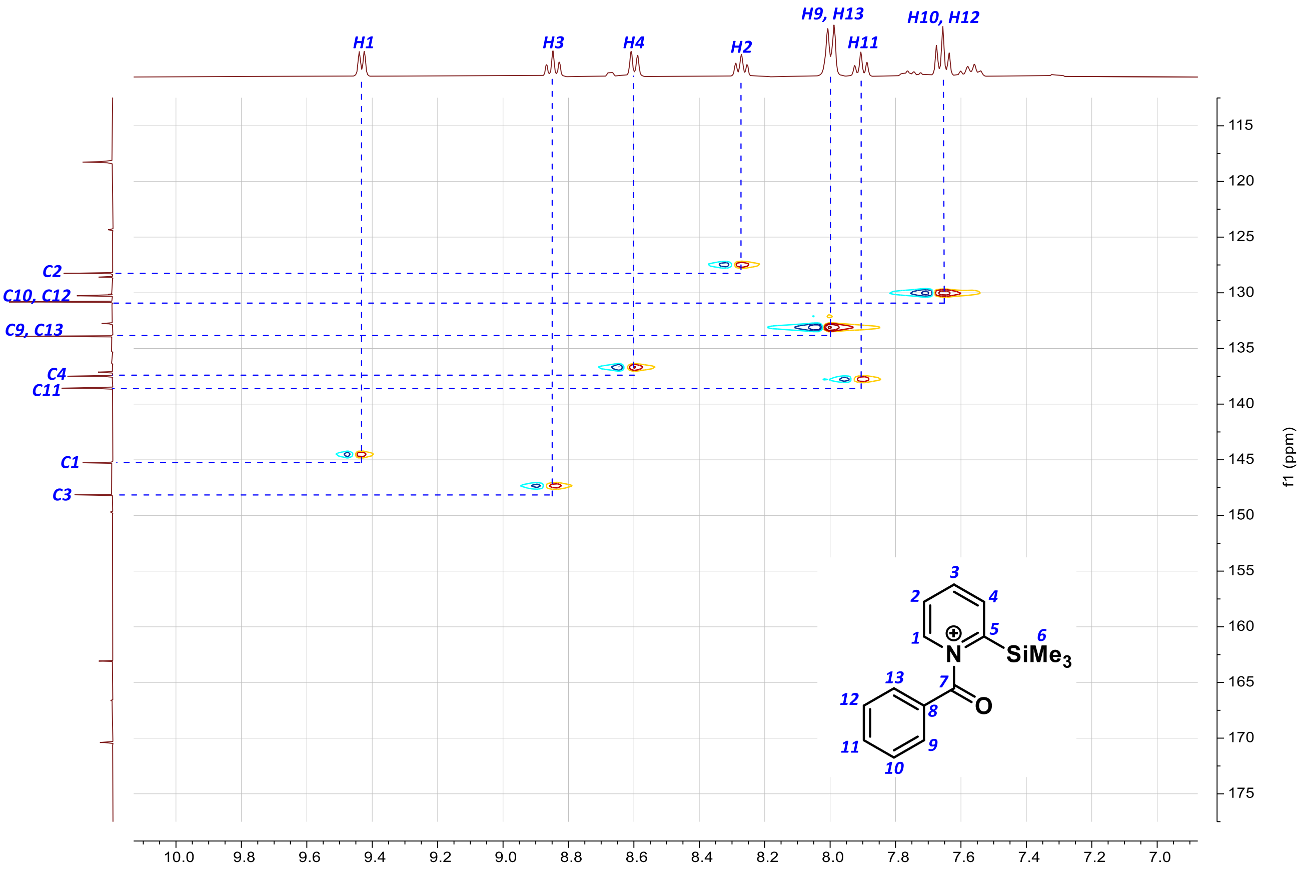

HMBCAD:

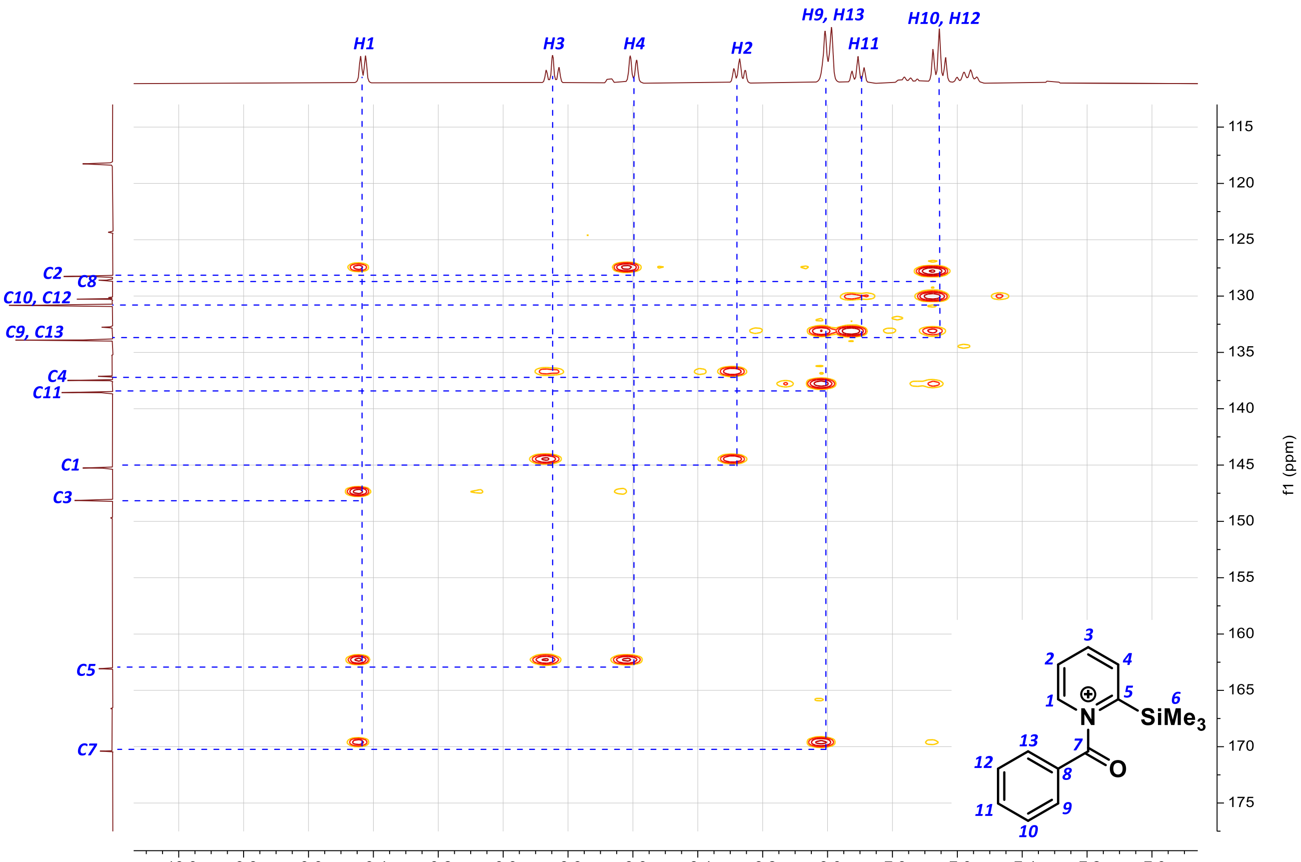

H1
H3
H4
H2
H9, H13
H11
H10, H12
C2
C8
C10, C12
C9, C13
C4
C11
C1
C3
C5
C7
f1 (ppm)
SiMe3
N
O

4.3. Reactivity of isomeric TMS-pyridines

Both, 3- and 4-(trimethylsilyl)pyridine did not form the corresponding ketone, when heated with benzoyl chloride at 60 °C and then at 120 °C. Nevertheless, formation of *N*-acylated species was observed:

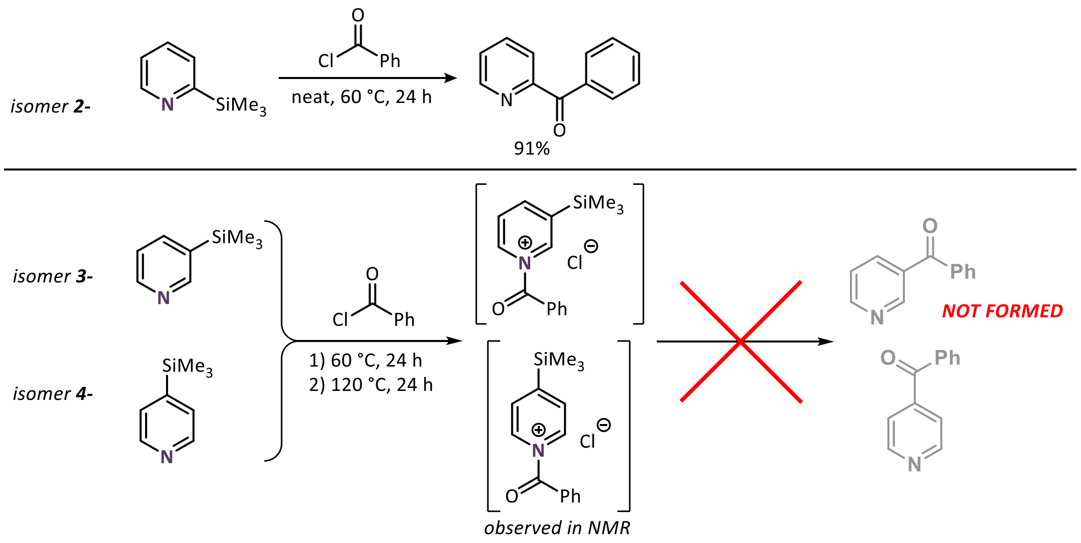

^{1}H NMR spectra ($CDCl_3$) of substrates and reaction mixtures:

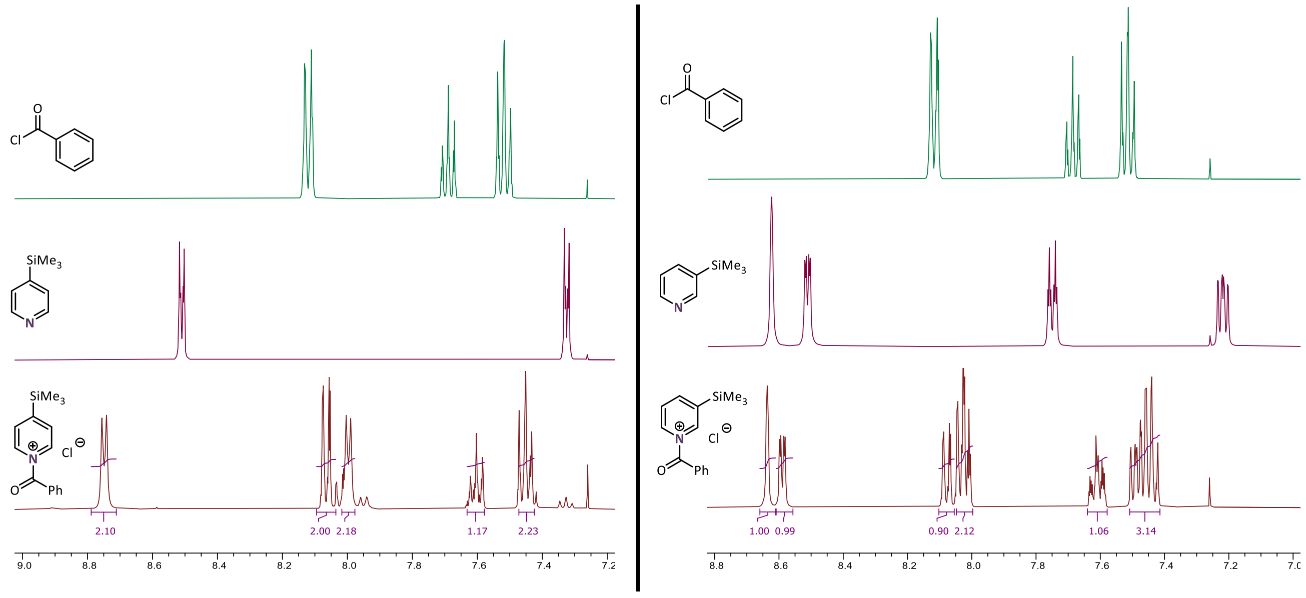

When 2,3- and 4-TMS-Py (1 mmol) with PhCOF (1 mmol), TBAT (30 mol%) in CD_2Cl_2 (0.5 mL) were heated at 40 °C for 1 d , we observed the following conversions of the substrates: 100%, 18% and 3%, respectively (see GC chromatograms below):

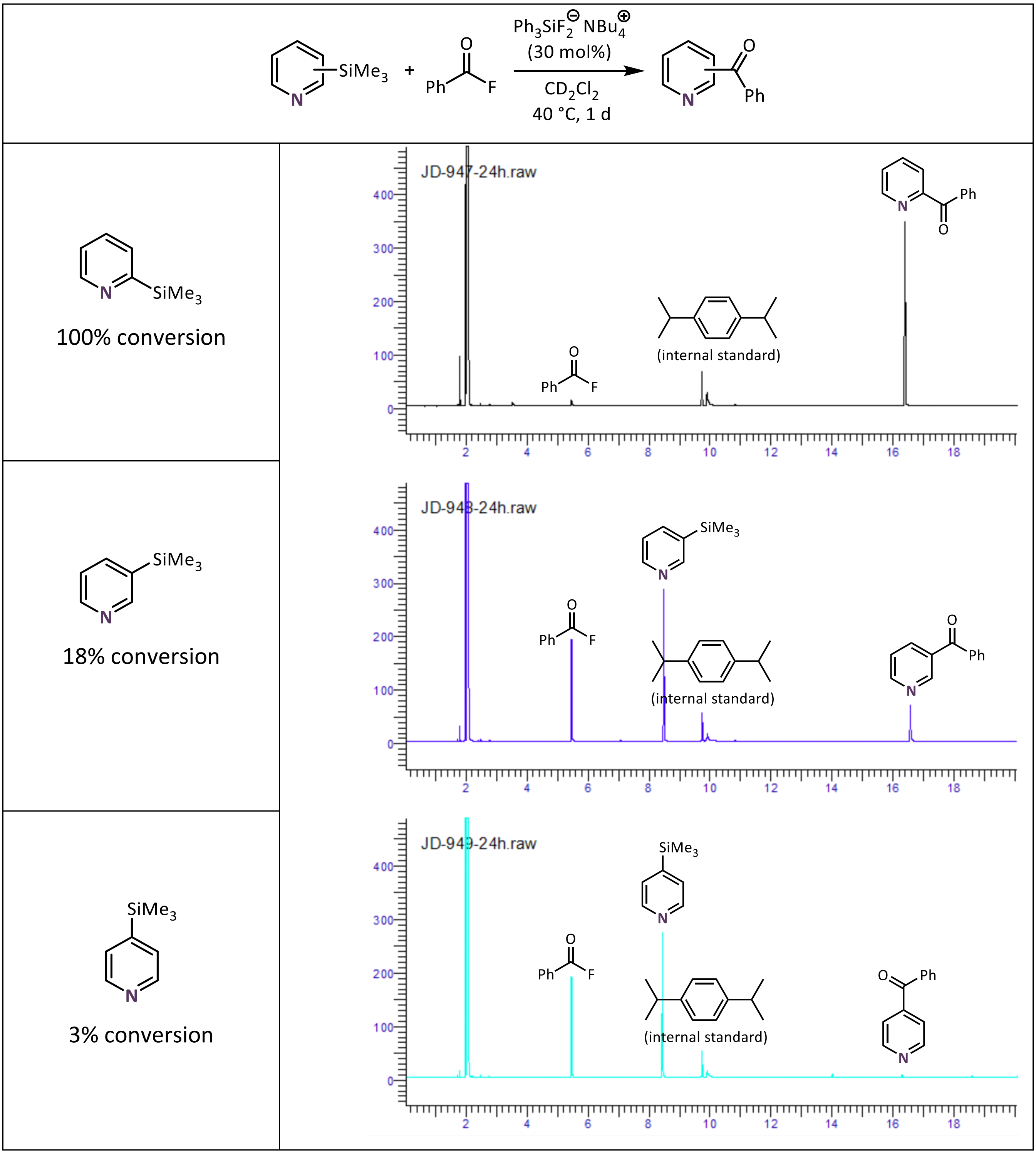

4.4. DFT calculations data

Computational methods

The chemical structures optimization (local minima and transition states) was performed using Gaussian 09[1] suite of programs, while initial cartesian coordinates were prepared using the GaussView 6[2] software. Chemical structure visualization on the main graph was made using CYLview20 software.[3] The M06-2X method[4] within Density functional theory (DFT) was applied to determine the energetics of the key mechanistic reaction steps. The unrestricted DFT computations were performed using a valence split-valence basis set (6-31++g(d,p))[5] including polarization functions for hydrogen and diffuse and polarization functions on oxygen and carbon atoms. The implicit solvation model based on electron density (SMD)[6] was used in all computations to reflect the proper atomic behavior in pyridine solution (dielectric constant for pyridine: ε= 12.978). No geometry restrictions have been applied, while thermal and zero-point vibrational energy (ZPE) corrections have been used in the calculations. Harmonic frequency analysis has been made in order to localize ground state intermediates (IC) and transition state (TS) structures. Intrinsic reaction coordinate (IRC)[7,8] calculations were carried out, to verify the connection between the TS and IC structures.

1. Frisch, M.J., et al., *Gaussian 16 Rev. B.01*. 2016: Wallingford, CT.
2. Dennington, R., T. Keith, and J. Millam, *GaussView ver.5*. 2009, Semichem Inc.; Shawnee Mission, KS.
3. CYLview20; Legault, C.Y., Université de Sherbrooke, 2020 (http://www.cylview.org).
4. Zhao, Y., N.E. Schultz, and D.G. Truhlar, *Design of Density Functionals by Combining the Method of Constraint Satisfaction with Parametrization for Thermochemistry, Thermochemical Kinetics, and Noncovalent Interactions.* Journal of Chemical Theory and Computation, 2006. **2**(2): p. 364-382.
5. Ditchfield, R., W.J. Hehre, and J.A. Pople, *Self-Consistent Molecular-Orbital Methods. IX. An Extended Gaussian-Type Basis for Molecular-Orbital Studies of Organic Molecules.* The Journal of Chemical Physics, 1971. **54**(2): p. 724-728.
6. Marenich, A.V., C.J. Cramer, and D.G. Truhlar, *Universal Solvation Model Based on Solute Electron Density and on a Continuum Model of the Solvent Defined by the Bulk Dielectric Constant and Atomic Surface Tensions.* The Journal of Physical Chemistry B, 2009. **113**(18): p. 6378-6396.
7. Gonzalez, C. and H.B. Schlegel, *Reaction path following in mass-weighted internal coordinates.* The Journal of Physical Chemistry, 1990. **94**(14): p. 5523-5527.
8. Carlos, G. and S. Bernhard, *An improved algorithm for reaction path following.* The Journal of Chemical Physics, 1989. **90**(4): p. 2154-2161.

Potential energy surface plot for all alternative mechanistic pathways:

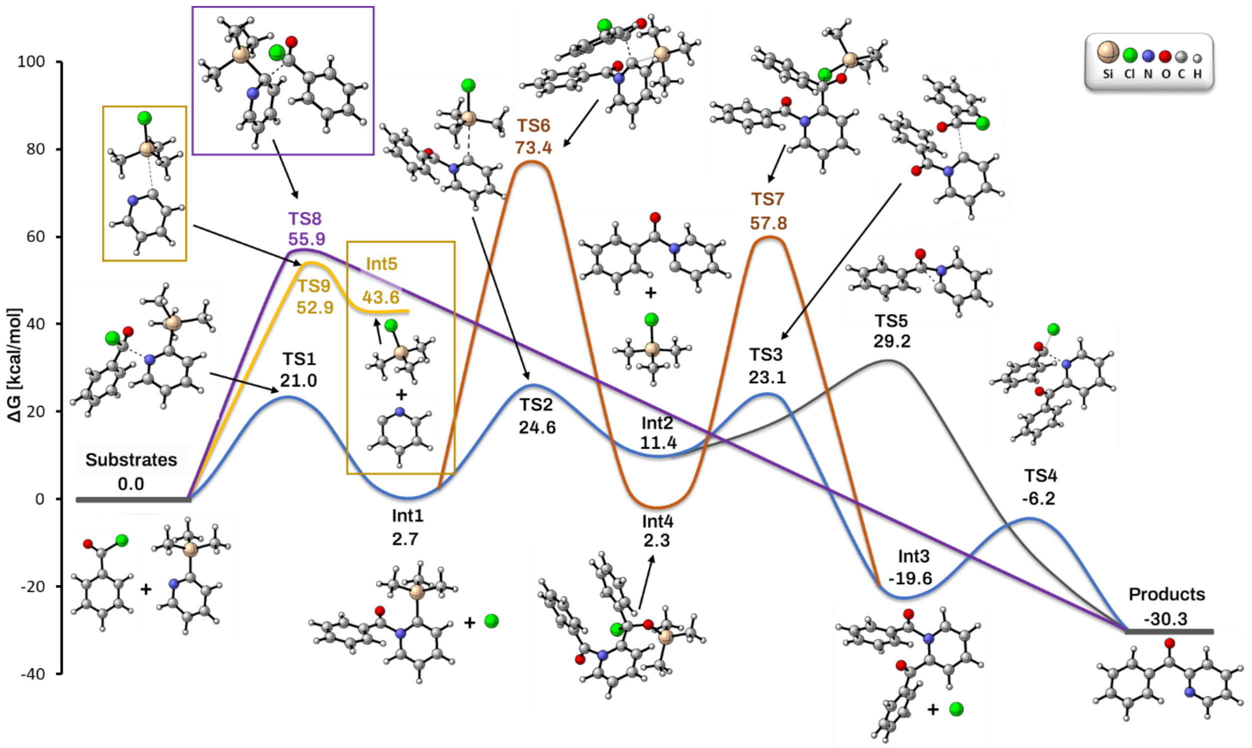

Gibbs free energy profiles for all considered reaction pathways, computed at the M06-2X/6-31++g(d,p) level of theory, with SMD solvation model for Pyridine.

Cartesian coordinates and thermochemistry (M06-2X/6-31++g(d,p) (SMD = Pyridine)):

Substrate_a

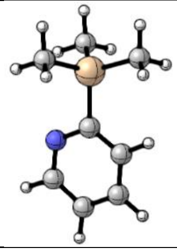

Thermochemistry:

Zero-point correction=	0.190790 (Hartree/Particle)
Thermal correction to Energy=	0.202953
Thermal correction to Enthalpy=	0.203897
Thermal correction to Gibbs Free Energy=	0.152645
Sum of electronic and zero-point Energies=	-656.587504
Sum of electronic and thermal Energies=	-656.575342
Sum of electronic and thermal Enthalpies=	-656.574398
Sum of electronic and thermal Free Energies=	-656.625650

Cartesian coordinates:

0 1

C	0.44089700	0.05152700	-0.00001100
C	1.19341800	1.23212000	-0.00019300
C	2.58625000	1.16433800	-0.00024300

C	3.19207500	-0.08722400	-0.00010100
C	2.37354700	-1.21758800	0.00013600
N	1.03930800	-1.16176800	0.00014600
H	3.18510100	2.07063400	-0.00040400
H	0.69215900	2.19626700	-0.00030500
H	4.27166300	-0.19580300	-0.00016600
H	2.81426900	-2.21226000	0.00018700
C	-2.01681600	-0.91576900	-1.53805300
H	-3.10888900	-1.00535200	-1.56145300
H	-1.69895500	-0.39732800	-2.44926500
H	-1.59340400	-1.92602100	-1.55612400
C	-2.01678100	-0.91527300	1.53845500
H	-1.69908700	-0.39643800	2.44950000
H	-3.10884300	-1.00502100	1.56179100
H	-1.59321500	-1.92545300	1.55693800
C	-2.11972900	1.76404700	-0.00023600
H	-3.21583300	1.75254900	-0.00025000
H	-1.79379800	2.31757400	0.88738400
H	-1.79377600	2.31728800	-0.88802800
Si	-1.45926500	0.00604800	0.00004600

Substrate_b

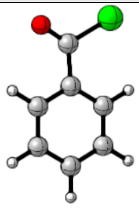

Thermochemistry:

Zero-point correction=	0.101574 (Hartree/Particle)
Thermal correction to Energy=	0.108987
Thermal correction to Enthalpy=	0.109932
Thermal correction to Gibbs Free Energy=	0.067905
Sum of electronic and zero-point Energies=	-804.934063
Sum of electronic and thermal Energies=	-804.926649
Sum of electronic and thermal Enthalpies=	-804.925705
Sum of electronic and thermal Free Energies=	-804.967731

Cartesian coordinates:

0 1

C	1.93307900	-1.40796100	0.00025000
C	0.56822500	-1.13379300	0.00031700
C	0.13279600	0.19531100	0.00008200
C	1.06255100	1.24576800	-0.00021800
C	2.42271000	0.96264900	-0.00031100
C	2.85823600	-0.36421800	-0.00007400

H	2.27293400	-2.43837000	0.00044900
H	-0.14512900	-1.94994300	0.00057700
H	0.71073200	2.27208000	-0.00039500
H	3.14262300	1.77468100	-0.00056800
H	3.92144500	-0.58452800	-0.00014200
C	-1.29536300	0.58691600	0.00019000
O	-1.72390800	1.69851900	0.00049500
Cl	-2.48263300	-0.77471200	-0.00031100

Product (ketone 1a)

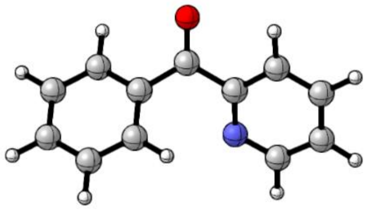

Thermochemistry:

Zero-point correction=	0.180915 (Hartree/Particle)
Thermal correction to Energy=	0.191567
Thermal correction to Enthalpy=	0.192511
Thermal correction to Gibbs Free Energy=	0.143170
Sum of electronic and zero-point Energies=	-592.287623
Sum of electronic and thermal Energies=	-592.276971
Sum of electronic and thermal Enthalpies=	-592.276027
Sum of electronic and thermal Free Energies=	-592.325368

Cartesian coordinates:

0 1

C	1.32218900	0.35018300	-0.02206900
C	2.45425900	0.94843600	0.53345000
C	3.66465700	0.26572700	0.46251900
C	3.69725600	-0.97096600	-0.17497000
C	2.51260200	-1.47824900	-0.71003700
H	2.37363600	1.92056500	1.00722900
H	4.56493200	0.69256600	0.89336100
H	4.61803000	-1.53809800	-0.26029900
H	2.50548300	-2.43732800	-1.22137700
C	0.01431800	1.09968000	0.00974500
O	0.03164900	2.31991400	0.01735000
N	1.34175500	-0.84228500	-0.63038800
C	-1.27827000	0.35177600	0.02654700
C	-1.39789000	-0.91716500	0.60593900
C	-2.41148400	0.98661900	-0.49681400
C	-2.64306300	-1.54016400	0.66266200
H	-0.52682600	-1.41006200	1.02418600

C	-3.64906800	0.35198900	-0.45956400
H	-2.30869400	1.97390700	-0.93685700
C	-3.76578900	-0.91162700	0.12340300
H	-2.73573000	-2.51825300	1.12440500
H	-4.52213700	0.84116900	-0.88034100
H	-4.73247700	-1.40522100	0.15874900

Int1

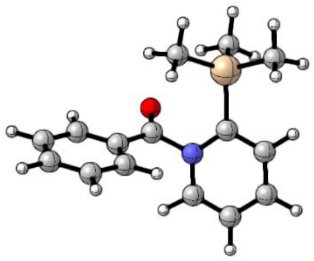

Thermochemistry:

Zero-point correction=	0.295573 (Hartree/Particle)
Thermal correction to Energy=	0.314178
Thermal correction to Enthalpy=	0.315122
Thermal correction to Gibbs Free Energy=	0.248260
Sum of electronic and zero-point Energies=	-1001.191754
Sum of electronic and thermal Energies=	-1001.173149
Sum of electronic and thermal Enthalpies=	-1001.172205
Sum of electronic and thermal Free Energies=	-1001.239068

Cartesian coordinates:

1 1

C	-1.54536800	0.55938700	-0.00026800
C	-2.44059000	1.56061200	0.37040400
C	-2.12721900	2.90924400	0.21287000
C	-0.88994800	3.27421500	-0.31090300
C	-0.00397100	2.27579400	-0.65787000
H	-3.39934400	1.27846800	0.79073800
H	-2.84482400	3.67047400	0.50103200
H	-0.60624100	4.30998500	-0.45121500
H	0.97934400	2.47282800	-1.07205600
Si	-2.01685000	-1.30928100	0.26770000
C	0.62197300	-0.03839100	-0.99577800
C	-0.51924100	-2.25847800	0.87395000
H	0.17049100	-2.54187600	0.07430000
H	0.03553300	-1.70592200	1.64043000
H	-0.88199600	-3.18340900	1.33813800
C	-2.73209600	-1.98122900	-1.32199600
H	-1.97837900	-2.05803300	-2.10952600
H	-3.14221300	-2.98052800	-1.13525300
H	-3.54842000	-1.34482700	-1.68015600

C	-3.32234800	-1.26982500	1.60866300
H	-4.25260800	-0.79909200	1.27519800
H	-3.55924400	-2.30432000	1.88378000
H	-2.97279200	-0.75651800	2.51066200
O	0.24365300	-0.75783600	-1.87817200
N	-0.34768000	0.97768200	-0.50050500
C	1.93509200	-0.05775900	-0.33377100
C	2.14999000	0.56391900	0.90430300
C	2.95996500	-0.78489700	-0.95623600
C	3.39576900	0.45707600	1.51412300
H	1.35238900	1.10814000	1.40172800
C	4.20354400	-0.87470000	-0.34497700
H	2.77387300	-1.26460000	-1.91198200
C	4.41987200	-0.25526100	0.88888300
H	3.56634000	0.92788400	2.47655900
H	5.00405900	-1.42678600	-0.82602100
H	5.39192000	-0.33125400	1.36636700

Int2_a and Int5_b

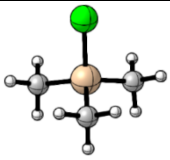

Thermochemistry:

Zero-point correction=	0.112918 (Hartree/Particle)
Thermal correction to Energy=	0.121561
Thermal correction to Enthalpy=	0.122505
Thermal correction to Gibbs Free Energy=	0.080285
Sum of electronic and zero-point Energies=	-869.283645
Sum of electronic and thermal Energies=	-869.275002
Sum of electronic and thermal Enthalpies=	-869.274058
Sum of electronic and thermal Free Energies=	-869.316278

Cartesian coordinates:

0 1

Si	-0.36297600	-0.00026900	0.00017300
C	-0.88201500	1.24140000	1.28984300
H	-1.97613100	1.29061900	1.33862700
H	-0.50881100	0.96108900	2.28010800
H	-0.50625000	2.24073400	1.04771000
C	-0.88275900	0.49610000	-1.71961400
H	-0.51102900	1.49470600	-1.97079800
H	-0.50598800	-0.21170100	-2.46497300
H	-1.97692200	0.51221100	-1.78626700
C	-0.88064200	-1.73839200	0.43037100

H	-1.97470900	-1.80644300	0.44741000
H	-0.50578900	-2.45502800	-0.30748100
H	-0.50528400	-2.02831400	1.41704200
Cl	1.76029900	0.00066100	-0.00043500

Int2_b

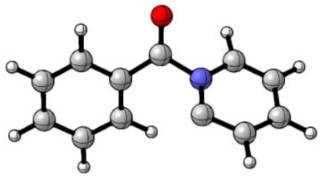

Thermochemistry:

Zero-point correction=	0.180214 (Hartree/Particle)
Thermal correction to Energy=	0.190931
Thermal correction to Enthalpy=	0.191875
Thermal correction to Gibbs Free Energy=	0.142478
Sum of electronic and zero-point Energies=	-592.221272
Sum of electronic and thermal Energies=	-592.210556
Sum of electronic and thermal Enthalpies=	-592.209611
Sum of electronic and thermal Free Energies=	-592.259008

Cartesian coordinates:

0 1

C	1.37682900	-0.69080800	1.00053100
C	2.66684700	-1.28217300	0.98199200
C	3.67624900	-0.90103200	0.11564600
C	3.45765400	0.15127900	-0.79169200
C	2.23595900	0.76664800	-0.77678500
H	2.86053200	-2.08432000	1.68888700
H	4.63982700	-1.40349400	0.13195700
H	4.22281900	0.48356200	-1.48286300
H	1.97458800	1.60207700	-1.41583000
C	0.02325900	1.08382500	0.08120300
O	0.09238600	2.28806800	0.09662300
N	1.26812200	0.31799800	0.07839400
C	-1.24802200	0.32349800	0.02485100
C	-1.32248300	-0.95509700	-0.54007800
C	-2.40861700	0.96865900	0.46597100
C	-2.55865600	-1.58342700	-0.65809600
H	-0.42455400	-1.45145600	-0.89287600
C	-3.63872400	0.32651500	0.36516000
H	-2.33643500	1.96406100	0.89303500
C	-3.71377700	-0.94824000	-0.19872400
H	-2.62005200	-2.57109500	-1.10366800
H	-4.53724300	0.81961700	0.72218900
H	-4.67452000	-1.44736500	-0.28243900

Int3

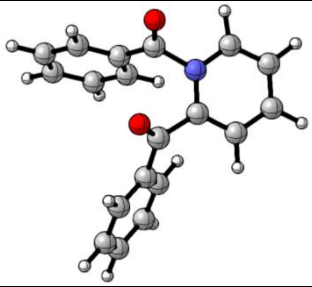

Thermochemistry:

Zero-point correction=	0.285316 (Hartree/Particle)
Thermal correction to Energy=	0.302538
Thermal correction to Enthalpy=	0.303483
Thermal correction to Gibbs Free Energy=	0.238661
Sum of electronic and zero-point Energies=	-936.879371
Sum of electronic and thermal Energies=	-936.862149
Sum of electronic and thermal Enthalpies	-936.861204
Sum of electronic and thermal Free Energies=	-936.926026

Cartesian coordinates:

1 1

C	0.53553200	-2.70265400	-0.24696100
C	1.63084500	-3.37061100	-0.80050500
C	2.86317700	-2.73322100	-0.87155700
C	2.97732400	-1.44180100	-0.38569600
H	-0.42940700	-3.18824200	-0.15309200
H	1.51623700	-4.38601600	-1.16460100
H	3.73484500	-3.21715400	-1.29454400
H	3.90067600	-0.87462900	-0.41461100
C	2.14270100	0.59915700	0.61502200
O	3.02332400	0.75730000	1.40613300
N	1.90858000	-0.81675200	0.14215100
C	1.30377900	1.62421600	-0.02663400
C	0.66342900	1.40094600	-1.25230000
C	1.19042800	2.85978800	0.62521700
C	-0.09692500	2.41854600	-1.82132000
H	0.76965200	0.45530900	-1.77525000
C	0.41988400	3.86427500	0.05460500
H	1.69104200	3.01403600	1.57594600
C	-0.22317900	3.64321900	-1.16650300
H	-0.58941600	2.25408300	-2.77403900
H	0.31630900	4.81857300	0.56016600
H	-0.82418100	4.43129200	-1.60956900
C	0.69071000	-1.41325100	0.22564900
C	-0.38722000	-0.68893600	1.01175200

O	-0.04247600	-0.14829700	2.04596700
C	-1.77955500	-0.70570200	0.51710100
C	-2.80409500	-0.42279600	1.43129100
C	-2.08110900	-0.93301100	-0.83278400
C	-4.12377200	-0.38578100	0.99917700
H	-2.55426800	-0.24220000	2.47213900
C	-3.40434900	-0.88026600	-1.26179100
H	-1.29031300	-1.12539200	-1.55223400
C	-4.42317600	-0.61369200	-0.34673300
H	-4.91990800	-0.17845400	1.70673600
H	-3.63973500	-1.04598200	-2.30798400
H	-5.45495400	-0.58052900	-0.68309300

TS1

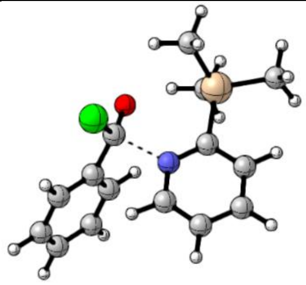

Thermochemistry:

Zero-point correction=	0.294150 (Hartree/Particle)
Thermal correction to Energy=	0.314219
Thermal correction to Enthalpy=	0.315163
Thermal correction to Gibbs Free Energy=	0.244497
Sum of electronic and zero-point Energies=	-1461.510298
Sum of electronic and thermal Energies=	-1461.490229
Sum of electronic and thermal Enthalpies=	-1461.489285
Sum of electronic and thermal Free Energies=	-1461.559951

**Frequency:**	**Infrared:**
-181.17	201.8017

Cartesian coordinates:

0 1

C	-1.48514200	0.84614100	-0.04543600
C	-2.06095800	2.12460400	-0.14590700
C	-1.33978300	3.26676800	0.18933000
C	-0.03243500	3.12804200	0.63933200
C	0.49325000	1.84519100	0.71139700
H	-3.08452000	2.22772800	-0.48592300
H	-1.79720400	4.24779800	0.10456000
H	0.57291500	3.98117200	0.92427500
H	1.51302700	1.67612700	1.04803700
Si	-2.62733800	-0.66361700	-0.46575800

C	0.74645200	-1.00847600	0.47857900
C	-2.83505500	-1.80242200	1.01140000
H	-1.93158600	-2.37065600	1.23683000
H	-3.11232800	-1.21845800	1.89701500
H	-3.65387900	-2.50399700	0.81027400
C	-2.15879300	-1.49913000	-2.08149900
H	-1.28218200	-2.14173800	-1.99128800
H	-3.00631700	-2.10414500	-2.42585700
H	-1.96255500	-0.74361700	-2.85159800
C	-4.33019200	0.09291500	-0.76963800
H	-4.35664400	0.75198300	-1.64446000
H	-5.02703900	-0.73209400	-0.96319400
H	-4.71165100	0.64676900	0.09517300
O	0.02291700	-1.81131800	-0.04816200
N	-0.20665100	0.76028600	0.37364400
Cl	0.76444800	-1.04835000	2.38804100
C	2.06393600	-0.57169400	-0.10332800
C	3.22320700	-0.35238600	0.63775400
C	2.08000400	-0.41681900	-1.49518600
C	4.40088700	0.01884500	-0.01547100
H	3.21700500	-0.47260900	1.71506900
C	3.25223200	-0.03800000	-2.13936900
H	1.16709900	-0.58698700	-2.06010900
C	4.41722300	0.17988200	-1.39856400
H	5.30405900	0.18326300	0.56395900
H	3.25941100	0.08671600	-3.21780900
H	5.33374700	0.47313100	-1.90161000

TS2

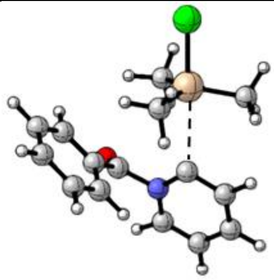

Thermochemistry:

Zero-point correction=	0.295239 (Hartree/Particle)
Thermal correction to Energy=	0.315117
Thermal correction to Enthalpy=	0.316061
Thermal correction to Gibbs Free Energy=	0.246664
Sum of electronic and zero-point Energies=	-1461.505594
Sum of electronic and thermal Energies=	-1461.485716
Sum of electronic and thermal Enthalpies=	-1461.484772
Sum of electronic and thermal Free Energies=	-1461.554169

Frequency:	Infrared:
-104.49	90.9466

Cartesian coordinates:

0 1

C	1.02933500	-1.18651200	-0.36835300
C	1.60005300	-2.31335100	-1.01164300
C	2.93695100	-2.66153800	-0.91348800
C	3.80049900	-1.90130600	-0.10789800
C	3.27599000	-0.81746100	0.54393500
H	0.94693300	-2.93187800	-1.62043900
H	3.32325500	-3.52416100	-1.44989700
H	4.84914800	-2.14768900	0.00686600
H	3.85178400	-0.17032400	1.19604600
Si	-1.83701200	-1.07535900	-0.03818900
C	1.49400800	0.67539400	1.11138100
C	-1.75863100	0.26026400	-1.34995200
H	-0.83742000	0.19755800	-1.93004000
H	-2.61454800	0.14299300	-2.02302200
H	-1.82254500	1.25722300	-0.89793200
C	-1.33265400	-0.78799600	1.74693600
H	-1.36033400	0.27757500	2.00069800
H	-2.04815700	-1.29851900	2.39991300
H	-0.33620200	-1.17773800	1.96433000
C	-1.65958300	-2.84098100	-0.64689400
H	-0.89452000	-3.37628300	-0.07664000
H	-2.60456900	-3.38217500	-0.54649800
H	-1.37129700	-2.85497000	-1.70343700
O	1.69326100	0.72354000	2.29873200
N	1.96010300	-0.49959900	0.36907600
Cl	-4.03237900	-0.96245500	0.29282400

C	0.85705800	1.74832400	0.31582300
C	1.14808200	1.91956700	-1.04206500
C	0.02398000	2.65571500	0.98129600
C	0.60233400	2.99867300	-1.73156600
H	1.79995600	1.21944100	-1.55584000
C	-0.54143100	3.71516500	0.28018300
H	-0.18194000	2.51644800	2.03835600
C	-0.24930000	3.88790000	-1.07514900
H	0.83284600	3.13918200	-2.78276300
H	-1.20407100	4.40856900	0.78814100
H	-0.68667200	4.71925400	-1.61985500

TS3

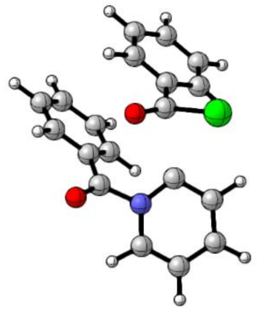

Thermochemistry:

Zero-point correction=	0.282339 (Hartree/Particle)
Thermal correction to Energy=	0.301459
Thermal correction to Enthalpy=	0.302403
Thermal correction to Gibbs Free Energy=	0.232778
Sum of electronic and zero-point Energies=	-1397.158435
Sum of electronic and thermal Energies=	-1397.139315
Sum of electronic and thermal Enthalpies=	-1397.138371
Sum of electronic and thermal Free Energies=	-1397.207997

**Frequency:**	**Infrared:**
-154.63	156.5618

Cartesian coordinates:

0 1

C	-3.12500000	0.18737600	-0.59151300
C	-4.14706300	-0.72727900	-0.40945400
C	-3.90223800	-1.91606100	0.30039500
C	-2.63627800	-2.13321300	0.77365800
H	-3.32941100	1.11324700	-1.12347600
H	-5.14060300	-0.53353700	-0.80489600
H	-4.67860100	-2.65077200	0.47626700
H	-2.34444700	-3.01323200	1.33614300
C	-0.33251100	-1.53015100	1.06096200
O	-0.23122100	-1.72715900	2.24537300

N	-1.66051600	-1.20992000	0.52484700
C	0.76933000	-1.66031700	0.08001900
C	0.55186800	-1.66030300	-1.30452300
C	2.05839100	-1.86158400	0.58941400
C	1.62617700	-1.84845500	-2.16923700
H	-0.44479200	-1.51819100	-1.70811100
C	3.12679600	-2.04962700	-0.28041500
H	2.21167400	-1.86473900	1.66403600
C	2.91158100	-2.03944300	-1.65961000
H	1.45865200	-1.84691000	-3.24155800
H	4.12681800	-2.19699900	0.11540700
H	3.74645600	-2.18328700	-2.33909600
C	-1.80742600	-0.02012800	-0.12393400
C	-0.44933900	1.91426600	-0.87049800
O	-0.54253100	1.80209100	-2.05424000
Cl	-1.63413200	3.10409000	-0.04577500
C	0.76726900	1.68192600	-0.03807200
C	1.97827300	1.56278500	-0.72418800
C	0.73783900	1.62634800	1.35720400
C	3.16531700	1.40740400	-0.00864100
H	1.98608600	1.60296600	-1.80902700
C	1.92367700	1.46089300	2.06490500
H	-0.21047600	1.70023100	1.88103800
C	3.14016700	1.35986800	1.38376900
H	4.10691100	1.31937200	-0.54242200
H	1.89998300	1.41013200	3.14919900
H	4.06464900	1.23634000	1.94022900

TS4

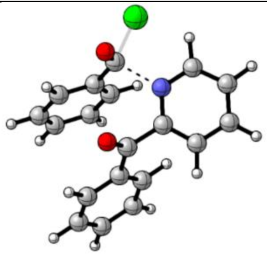

Thermochemistry:

Zero-point correction=	0.283291 (Hartree/Particle)
Thermal correction to Energy=	0.302225
Thermal correction to Enthalpy=	0.303169
Thermal correction to Gibbs Free Energy=	0.234946
Sum of electronic and zero-point Energies=	-1397.206292
Sum of electronic and thermal Energies=	-1397.187358
Sum of electronic and thermal Enthalpies=	-1397.186414
Sum of electronic and thermal Free Energies=	-1397.254637

**Frequency:**	**Infrared:**		
-251.30	510.5559		
**Cartesian coordinates:**			
0 1			
C	-0.89285500	2.85137400	0.15639100
C	-2.08798100	3.38859900	-0.31323900
C	-3.16549200	2.53780000	-0.52622500
C	-3.00181500	1.17760200	-0.29071400
H	-0.03136500	3.47948700	0.36094700
H	-2.17516000	4.45446100	-0.49736900
H	-4.12276800	2.90659900	-0.87571900
H	-3.80559700	0.47045800	-0.45740600
C	-1.79030100	-1.20375400	0.59668900
O	-2.26670500	-1.34106200	1.67262900
N	-1.84699100	0.66267800	0.14250700
C	-0.45707700	-1.64577200	0.08232800
C	-0.02171700	-1.38154600	-1.21628200
C	0.35624300	-2.34321700	0.97833100
C	1.23652600	-1.81731800	-1.62044600
H	-0.66557700	-0.83846100	-1.90198400
C	1.61567300	-2.77549600	0.56771800
H	0.00613800	-2.52855700	1.98877600
C	2.05682600	-2.51237300	-0.72968300
H	1.58027700	-1.60803300	-2.62875300
H	2.25286800	-3.31264500	1.26348900
H	3.04072200	-2.84563000	-1.04650900
C	-0.81138400	1.48246800	0.39372900
C	0.41679600	0.94818700	1.11155000
O	0.27339000	0.55593200	2.25341300
Cl	-3.12628200	-1.85871200	-0.92852300
C	1.74166300	1.00162100	0.43766700
C	2.86176900	0.55336000	1.14981600
C	1.88536200	1.44137300	-0.88408400
C	4.11461800	0.54937100	0.54631400
H	2.73437600	0.20396800	2.16989300
C	3.14197400	1.44050600	-1.48416300
H	1.02060100	1.76730900	-1.45473000
C	4.25488200	0.99426900	-0.77054700
H	4.98103900	0.19843000	1.09811100
H	3.25092100	1.78007100	-2.50931100
H	5.23352600	0.99062200	-1.24133500

TS5

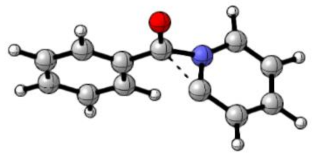

Thermochemistry:

Zero-point correction=	0.178592 (Hartree/Particle)
Thermal correction to Energy=	0.188853
Thermal correction to Enthalpy=	0.189797
Thermal correction to Gibbs Free Energy=	0.140885
Sum of electronic and zero-point Energies=	-592.192837
Sum of electronic and thermal Energies=	-592.182577
Sum of electronic and thermal Enthalpies=	-592.181632
Sum of electronic and thermal Free Energies=	-592.230544

**Frequency:**	**Infrared:**
-327.94	165.5394

Cartesian coordinates:

0 1

C	1.35173700	0.24271900	0.86765800
C	2.54145900	-0.20365700	1.45795500
C	3.53150100	-0.68565300	0.60500900
C	3.38233300	-0.65963400	-0.79435100
C	2.22012200	-0.15072800	-1.34282900
H	2.68495000	-0.18834200	2.53224000
H	4.45371100	-1.07971000	1.02297200
H	4.17381000	-1.01978700	-1.44129500
H	2.01695500	-0.04584400	-2.40219200
C	0.10145900	1.06874700	-0.04279900
O	0.14276300	2.29232500	-0.20046300
N	1.27928600	0.21575900	-0.45848700
C	-1.19757300	0.30068800	-0.00868000
C	-1.26855700	-1.09290000	-0.09623700
C	-2.37212600	1.04697000	0.09871400
C	-2.50647800	-1.73084800	-0.07654100
H	-0.36172100	-1.68553500	-0.17838900
C	-3.61060700	0.40570600	0.12725500
H	-2.30201100	2.12835100	0.15537000
C	-3.68069100	-0.98375000	0.03856800
H	-2.55435800	-2.81315600	-0.14980200
H	-4.51968600	0.99324800	0.21451200
H	-4.64423100	-1.48409800	0.05736100

Cl

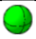

Thermochemistry:

Zero-point correction=	0.000000 (Hartree/Particle)
Thermal correction to Energy=	0.001416
Thermal correction to Enthalpy=	0.002360
Thermal correction to Gibbs Free Energy=	-0.015023
Sum of electronic and zero-point Energies=	-460.335043
Sum of electronic and thermal Energies=	-460.333627
Sum of electronic and thermal Enthalpies=	-460.332683
Sum of electronic and thermal Free Energies=	-460.350066

Cartesian coordinates:

-1 1

Cl	0.00000000	0.00000000	0.00000000

Int4

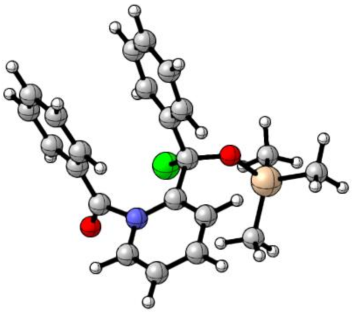

Thermochemistry:

Zero-point correction=	0.399362 (Hartree/Particle)
Thermal correction to Energy=	0.425953
Thermal correction to Enthalpy=	0.426897
Thermal correction to Gibbs Free Energy=	0.342766
Sum of electronic and zero-point Energies=	-1806.150751
Sum of electronic and thermal Energies=	-1806.124160
Sum of electronic and thermal Enthalpies=	-1806.123215
Sum of electronic and thermal Free Energies=	-1806.207347

Cartesian coordinates:

1 1

C	0.67373200	-0.97389500	0.57208200
C	1.50675800	-1.22157400	1.65403100
C	1.49155000	-2.45330100	2.30317500
C	0.64333400	-3.45556000	1.85130100
C	-0.17513100	-3.18128400	0.77554900
H	2.17515400	-0.43760900	1.98660300
H	2.14836700	-2.62374200	3.14965000
H	0.60439500	-4.43456200	2.31240200

H	-0.86907600	-3.90975200	0.37120000
Si	3.51676800	0.78359900	-0.42260100
C	-1.18479400	-1.88427300	-0.95352900
C	4.57428400	1.47753900	0.94405600
H	4.35139200	2.53597000	1.11526200
H	4.41820800	0.93674900	1.88375300
H	5.63430600	1.39420200	0.67888400
C	3.68952600	1.77524400	-1.99351300
H	3.28229800	1.25054900	-2.86240800
H	3.18422400	2.74282100	-1.90414200
H	4.75291500	1.96691700	-2.18073500
C	3.85927500	-1.03233800	-0.70793400
H	3.07579300	-1.52563700	-1.29320700
H	4.79382700	-1.11840100	-1.27609600
H	3.99049400	-1.57906300	0.23139900
O	-0.97670000	-2.56678300	-1.91052600
N	-0.16568600	-1.96904000	0.17402100
Cl	0.62789000	0.18338000	-1.91638900
C	-2.36844600	-1.07019600	-0.65929100
C	-2.74234700	-0.76482000	0.65591500
C	-3.15057600	-0.65468200	-1.74534800
C	-3.91138700	-0.04665100	0.88095600
H	-2.13698800	-1.08968800	1.49739800
C	-4.30400300	0.08244800	-1.51049100
H	-2.83916200	-0.89823700	-2.75662600
C	-4.68498200	0.38063200	-0.19939600
H	-4.21335100	0.18708100	1.89660500
H	-4.90790000	0.42326300	-2.34501100
H	-5.58973900	0.95345600	-0.01908500
H	-1.45962900	2.55001200	3.40091200
C	-1.40751700	2.41051400	2.32557800
H	-3.01746900	3.77980900	1.89862600
H	0.21764700	1.01963600	2.45855700
C	-2.27814400	3.10209500	1.48272000
C	-0.46167300	1.54105900	1.78996400
C	-2.19229900	2.91868900	0.10480200
C	-0.38194700	1.34944800	0.40574300
C	-1.24763400	2.04563300	-0.43599300
C	0.73751600	0.42754300	-0.09325500
H	-2.86475300	3.45118300	-0.56102600
O	1.92830400	1.01343300	0.21328000
H	-1.20344300	1.91366000	-1.51081100

**TS6**	
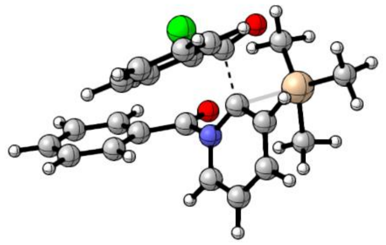	
**Thermochemistry:**	
Zero-point correction=	0.397351 (Hartree/Particle)
Thermal correction to Energy=	0.423946
Thermal correction to Enthalpy=	0.424890
Thermal correction to Gibbs Free Energy=	0.341655
Sum of electronic and zero-point Energies=	-1806.036856
Sum of electronic and thermal Energies=	-1806.010261
Sum of electronic and thermal Enthalpies=	-1806.009317
Sum of electronic and thermal Free Energies=	-1806.092552
**Frequency:**	**Infrared:**
-352.57	745.7852

Cartesian coordinates:

1 1

C	1.03729700	-0.09091800	0.42215300
C	1.91837900	0.48619300	1.33970100
C	1.71967000	0.42273300	2.72637800
C	0.57656300	-0.18967400	3.19962500
C	-0.31700100	-0.74692600	2.28161700
H	2.76957700	1.03166400	0.94128600
H	2.44114800	0.86522900	3.40497100
H	0.35019000	-0.26188300	4.25679500
H	-1.21034900	-1.27190200	2.60545800
Si	2.58779900	-1.77601300	-0.21669200
C	-1.00520800	-1.54823100	0.12114200
C	4.25592500	-1.00287600	0.01420400
H	4.36650500	-0.07452100	-0.54968800
H	4.49276500	-0.83558500	1.06864700
H	4.97632900	-1.72972200	-0.38683100
C	2.21357800	-2.24621900	-1.96044700
H	1.20370500	-2.62522800	-2.10785600
H	2.41513900	-1.42157200	-2.64526300
H	2.93093700	-3.05678400	-2.16428300
C	2.18431100	-2.98530800	1.13009700
H	1.26492500	-3.54320000	0.94833600
H	3.02300000	-3.69508500	1.15486700
H	2.14201800	-2.50763800	2.11477600
O	-0.48955900	-2.48409200	-0.41507700
N	-0.08732600	-0.69146700	0.96427600
Cl	-0.25244300	-0.00492100	-2.31443700
C	-2.43863100	-1.21029200	0.13112200

C	-2.97806700	-0.10985000	0.81156900
C	-3.27297400	-2.06132100	-0.60979800
C	-4.34663600	0.13250800	0.74796200
H	-2.35117500	0.56742900	1.38174000
C	-4.63680500	-1.80969900	-0.66982300
H	-2.84160100	-2.90604800	-1.13672600
C	-5.17387200	-0.71351200	0.00941400
H	-4.76498700	0.98482800	1.27298800
H	-5.28161400	-2.46474000	-1.24614500
H	-6.24055700	-0.51732900	-0.03899700
H	1.56905200	5.34467300	0.03907700
C	0.88516800	4.50911700	-0.07532200
H	-0.84674600	5.62638700	0.55434200
H	2.42315300	3.14591500	-0.74014900
C	-0.47049600	4.66758000	0.21092300
C	1.36969800	3.27767600	-0.51428500
C	-1.34401500	3.59212700	0.04249700
C	0.49381500	2.20026600	-0.67509500
C	-0.86397300	2.36213900	-0.40204500
C	1.13441200	0.88236600	-1.09832400
H	-2.40413000	3.71118000	0.24652900
O	2.25843600	0.85595800	-1.57013900
H	-1.55149100	1.53983900	-0.56256300

TS7

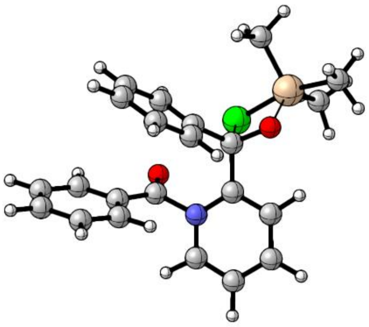

Thermochemistry:

Zero-point correction=	0.399408 (Hartree/Particle)
Thermal correction to Energy=	0.424075
Thermal correction to Enthalpy=	0.425019
Thermal correction to Gibbs Free Energy=	0.345476
Sum of electronic and zero-point Energies=	-1806.065112
Sum of electronic and thermal Energies=	-1806.040445
Sum of electronic and thermal Enthalpies=	-1806.039501
Sum of electronic and thermal Free Energies=	-1806.119044

**Frequency:**	**Infrared:**
-94.30	0.1271

**Cartesian coordinates:**			
1 1			
C	-0.49118200	1.48209000	0.30194700
C	-1.17336900	2.47906300	0.97228600
C	-0.61683000	3.75556700	1.07741200
C	0.62561100	4.01276400	0.51411800
C	1.27889800	2.98940400	-0.14955400
H	-2.12990600	2.23952700	1.41975600
H	-1.15584700	4.53743200	1.60165700
H	1.09439100	4.98720600	0.57257300
H	2.24365300	3.11906500	-0.62944100
Si	-3.53028700	-0.59496800	-0.12134700
C	1.44324500	0.79067700	-1.14838300
C	-4.67526600	-0.56075500	1.42396500
H	-4.25859300	-1.14928400	2.25062400
H	-4.83113400	0.46188500	1.79313800
H	-5.66088800	-0.97472900	1.17725000
C	-3.44968400	-2.44840000	-0.48243000
H	-3.33542100	-2.67589800	-1.54827400
H	-2.59731700	-2.89787900	0.04405000
H	-4.35652600	-2.94110200	-0.11702700
C	-4.67634200	0.38714000	-1.28201100
H	-4.48804800	0.21003900	-2.34860300
H	-5.72309600	0.14933500	-1.06889600
H	-4.54221500	1.46375200	-1.10556100
O	0.87805200	0.51459900	-2.16673800
N	0.72468900	1.76371000	-0.24135900
Cl	-1.96403300	-0.16576500	-1.40032000
C	2.76484400	0.34528100	-0.69467100
C	3.22562300	0.56255400	0.61059200
C	3.54371500	-0.36758600	-1.61986000
C	4.47169700	0.06887000	0.98440900
H	2.61739500	1.09090500	1.33853000
C	4.78734900	-0.85020700	-1.23819200
H	3.16679300	-0.53279300	-2.62426600
C	5.24987700	-0.63178900	0.06297500
H	4.83149600	0.22685800	1.99564300
H	5.39707400	-1.39672600	-1.94989900
H	6.22222900	-1.01294600	0.35972500
H	1.83387000	-1.96218500	3.20217800
C	1.42440100	-1.96842700	2.19689800
H	2.61579700	-3.64016400	1.54177300
H	0.13361900	-0.28616200	2.56224200
C	1.85967700	-2.91215200	1.26465200
C	0.46803200	-1.02459700	1.83787600
C	1.31955800	-2.92221300	-0.01963700
C	-0.05748600	-1.02461800	0.53917800
C	0.35636000	-1.98176900	-0.38530400

C	-1.07064300	0.06749800	0.27144300
H	1.64871600	-3.65818200	-0.74624900
O	-2.20152500	-0.02155600	0.99666800
H	-0.04701700	-1.99107900	-1.39279400

TS9

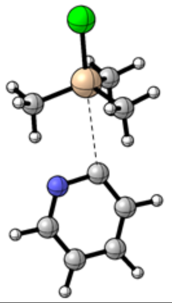

Thermochemistry:

Zero-point correction=	0.210533 (Hartree/Particle)
Thermal correction to Energy=	0.223430
Thermal correction to Enthalpy=	0.224374
Thermal correction to Gibbs Free Energy=	0.169135
Sum of electronic and zero-point Energies=	-1104.086043
Sum of electronic and thermal Energies=	-1104.073146
Sum of electronic and thermal Enthalpies=	-1104.072202
Sum of electronic and thermal Free Energies=	-1104.127441

Frequency: Infrared:

-144.46 21.0543

Cartesian coordinates:

-1 1

C	1.49540400	0.50876600	-0.39988300
C	2.64882500	1.32531000	-0.10584900
C	3.92056700	0.86181200	0.21855700
C	4.13422300	-0.52005700	0.26673900
C	3.03743600	-1.33325700	-0.01716800
H	2.52533500	2.41626100	-0.13398400
H	4.74707900	1.54864200	0.43392300
H	5.10888900	-0.94499600	0.51439400
H	3.16952200	-2.42645900	0.01184900
Si	-1.67976900	0.03845500	-0.01347200
C	-1.03699100	-1.66826300	-0.33245500
H	0.07313500	-1.59525200	-0.37514400
H	-1.32779500	-2.35488600	0.46542800
H	-1.41411500	-2.06298200	-1.27810400
C	-1.41710500	1.22577300	-1.41562300
H	-1.79909700	0.83969600	-2.36290100
H	-1.86914600	2.20091700	-1.22298700

H	-0.32066500	1.31932200	-1.45932300
C	-1.14563600	0.72945600	1.62985400
H	-1.62610600	1.68883000	1.83628300
H	-1.38107300	0.04529500	2.44866000
H	-0.06327100	0.88357800	1.60551600
N	1.78331500	-0.88049500	-0.33375800
Cl	-3.80055600	-0.15941900	0.17507500

TS8

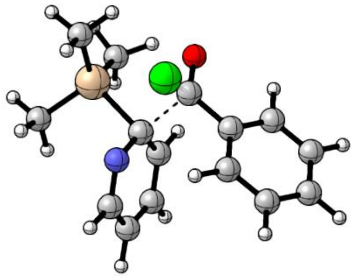

Thermochemistry:

Zero-point correction=	0.292316 (Hartree/Particle)
Thermal correction to Energy=	0.312626
Thermal correction to Enthalpy=	0.313570
Thermal correction to Gibbs Free Energy=	0.244067
Sum of electronic and zero-point Energies=	-1461.456043
Sum of electronic and thermal Energies=	-1461.435733
Sum of electronic and thermal Enthalpies=	-1461.434789
Sum of electronic and thermal Free Energies=	-1461.504293

Frequency: Infrared:

-358.10 1341.0959

Cartesian coordinates:

0 1

C	-0.62464100	0.43414300	-0.03297800
C	-0.27593200	1.29011100	1.04347600
C	-0.00340700	2.63702200	0.82056400
C	-0.07507900	3.09927700	-0.48413000
C	-0.42305600	2.20210100	-1.51739700
H	-0.21321700	0.87625300	2.04770700
H	0.26437400	3.29753500	1.63852900
H	0.13475500	4.13642000	-0.72588900
H	-0.48615300	2.55857800	-2.54268800
Si	-2.51146100	-0.23558400	0.36471100
C	-2.64480300	-0.42929700	2.21735800
H	-1.85199300	-1.05862400	2.62754900
H	-2.63407400	0.54340500	2.71987300
H	-3.60754000	-0.91056300	2.43109700

C	-2.93071900	-1.78936900	-0.56257500
H	-2.82220500	-1.66438600	-1.64210900
H	-2.32757900	-2.63962200	-0.24003900
H	-3.98672700	-1.99431600	-0.33868400
C	-3.56014500	1.20115200	-0.21604900
H	-3.59321800	1.25782000	-1.30836500
H	-4.58497600	1.06581100	0.15028300
H	-3.19058400	2.15700400	0.17270000
N	-0.67582900	0.92465100	-1.31412900
Cl	0.11491300	-2.05455700	-1.68893800
H	4.43388300	-0.59543000	2.28302900
C	3.84570600	-0.40720300	1.38975600
H	5.42438600	0.64609600	0.37060400
H	2.08553900	-1.40375800	2.15320900
C	4.40089700	0.28734400	0.31732000
C	2.52959700	-0.86692400	1.32109200
C	3.63832400	0.51490500	-0.83134400
C	1.77067800	-0.63621200	0.17199100
C	2.32695200	0.05395900	-0.90725900
C	0.32698600	-1.11513500	0.21720300
H	4.06955000	1.04624900	-1.67439600
O	-0.09080300	-1.82898200	1.10094900
H	1.74180100	0.21122700	-1.80728000

Int5_a

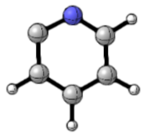

Thermochemistry:

Zero-point correction=	0.075231 (Hartree/Particle)
Thermal correction to Energy=	0.079570
Thermal correction to Enthalpy=	0.080514
Thermal correction to Gibbs Free Energy=	0.047805
Sum of electronic and zero-point Energies=	-247.562482
Sum of electronic and thermal Energies=	-247.558142
Sum of electronic and thermal Enthalpies=	-247.557198
Sum of electronic and thermal Free Energies=	-247.589907

Cartesian coordinates:

-1 1

C	0.25204200	-1.48193900	-0.00000600
C	1.28065700	-0.49645600	0.00002400
C	1.03366000	0.87414100	-0.00004900
C	-0.29259800	1.30912200	-0.00018400

C	-1.27951800	0.32828000	0.00006800
H	2.31811200	-0.83197000	-0.00001700
H	1.85067400	1.59440700	0.00037500
H	-0.55502800	2.36306400	0.00044900
H	-2.33033000	0.62318300	-0.00000700
N	-1.03555500	-0.99251100	0.00001200

5. Single-crystal XRD analysis data for 4d (CCDC 2417407)

X-Ray Diffraction Data Collection and Structure Refinement

Crystals of **4d** suitable for X-Ray studies were obtained by slow evaporation of its solution in dichloromethane/*n*-heptane (layered). Good quality single-crystal of **4d** were selected for the X-ray diffraction experiment at T = 100(2) K. The crystal was mounted with paratone-N oil on the the Hampton Research CryoLoop. Diffraction data were collected on the Agilent Technologies SuperNova Dual Source diffractometer with Cu$K\alpha$ radiation (λ = 1.54184 Å) using CrysAlis Pro software [CrysAlis CCD and CrysAlis RED; Oxford Diffraction Ltd: Yarnton, Oxfordshire, England, **2008**.]. The analytical numeric absorption correction using a multifaceted crystal model based on expressions derived by R.C. Clark & J.S. Reid was applied [Clark, R. C. & Reid, J. S. (1995). Acta Cryst. A51, 887−897.]. The structural determination procedure was carried out using the SHELX package. The structures were solved with intrinsic phasing method using SHELXT program [Sheldrick, G. M. SHELXT - Integrated space-group and crystal-structure determination. Acta Crystallogr. A Found. Adv., **2015**, *71*, 3−8.], and then successive least-squares refinement was carried out based on the full-matrix least-squares method on F^2 using the SHELXL program [Sheldrick, G. M. Crystal Structure Refinement with SHELXL. Acta Crystallogr. C Struct. Chem., **2015**, *71*, 3−8.]. All hydrogen atoms were positioned geometrically and refined with fixed U_{iso}(H) parameters. Additionally, one of the $-CF_3$ groups was refined as disordered in two respective positions with the site occupancy factors of 0.743(4) : 0.257(4). Figures for the publication and SI were prepared using the Mercury program [C. F. Macrae, I. J. Bruno, A. Chisholm, P. R. Edgington, P. McCabe, E. Pidcock, L. Rodriguez-Monge, R. Taylor, J. van de Streek, P. A. Wood, *J. Appl. Cryst.* **2008**, *41*, 466–470.].

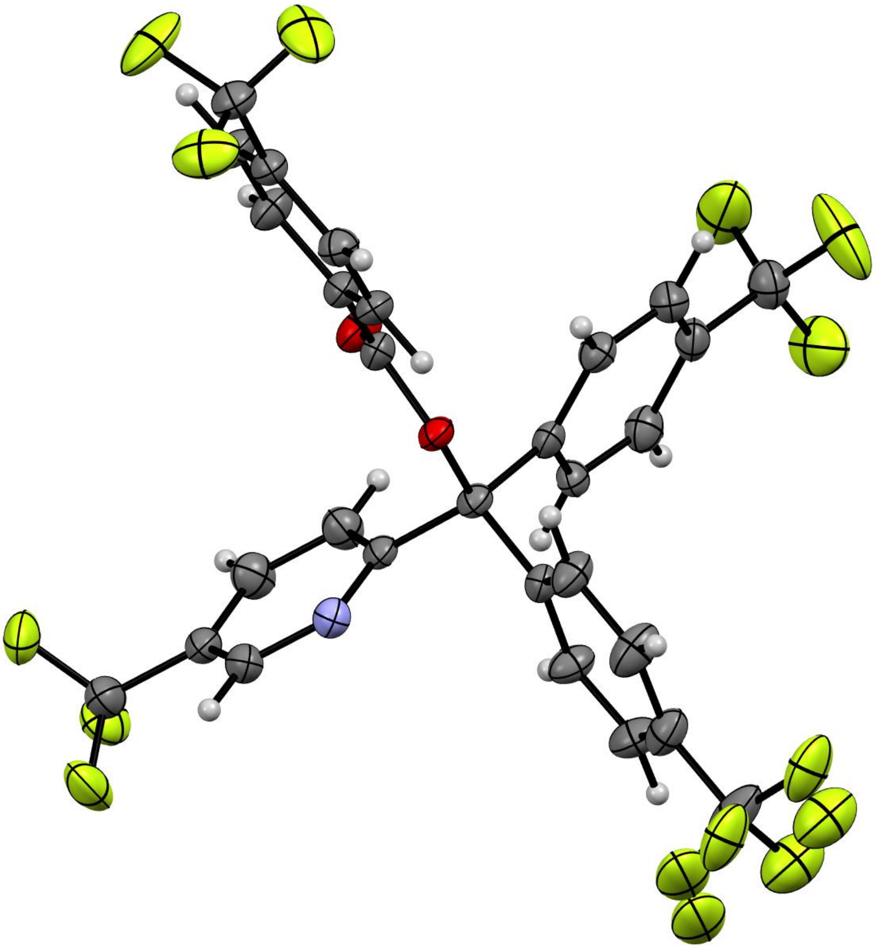

*Ellipsoid plot for **4d** (CCDC 2417407). Ellipsoid contour probability level is 50%.*

Crystal and structure refinement details for **4d**:

**Compound**	**4d**
Empirical formula	$C_{28}H_{14}F_{12}N_2O_2$
Formula weight	638.41
Temperature/K	100(2)
Crystal system	monoclinic
Space group	$P2_1/c$
a/Å	20.2602(4)
b/Å	11.0479(3)
c/Å	11.5301(3)
α/°	90
β/°	95.251(2)
γ/°	90
Volume/Å^3	2569.98(10)
Z	4
ρ_{calc}g/cm^3	1.647
μ/mm^{-1}	1.459
F(000)	1280.0
Crystal size/mm^3	0.204 × 0.135 × 0.116
Radiation	Cu$K\alpha$ (λ = 1.54184)
2Θ range for data collection/°	8.766 to 134.124
Index ranges	$-24 \le h \le 23$, $-12 \le k \le 13$, $-13 \le l \le 13$
Reflections collected	12559
Independent reflections	4581 [R_{int} = 0.0292, R_{sigma} = 0.0356]
Data/restraints/parameters	4581/34/434
Goodness-of-fit on F^2	1.059
Final R indexes [I>=2σ (I)]	R_1 = 0.0495, wR_2 = 0.1308
Final R indexes [all data]	R_1 = 0.0579, wR_2 = 0.1387
Largest diff. peak/hole / e Å^{-3}	0.51/-0.33

Acknowledgments

The XRD data collection was accomplished at the Core Facility for Crystallographic and Biophysical research to support the development of medicinal products sponsored by the Foundation for Polish Science (FNP).

6. NMR spectra reproductions

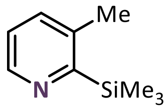

HetSi_01

^{1}H NMR (400 MHz, $CDCl_3$):

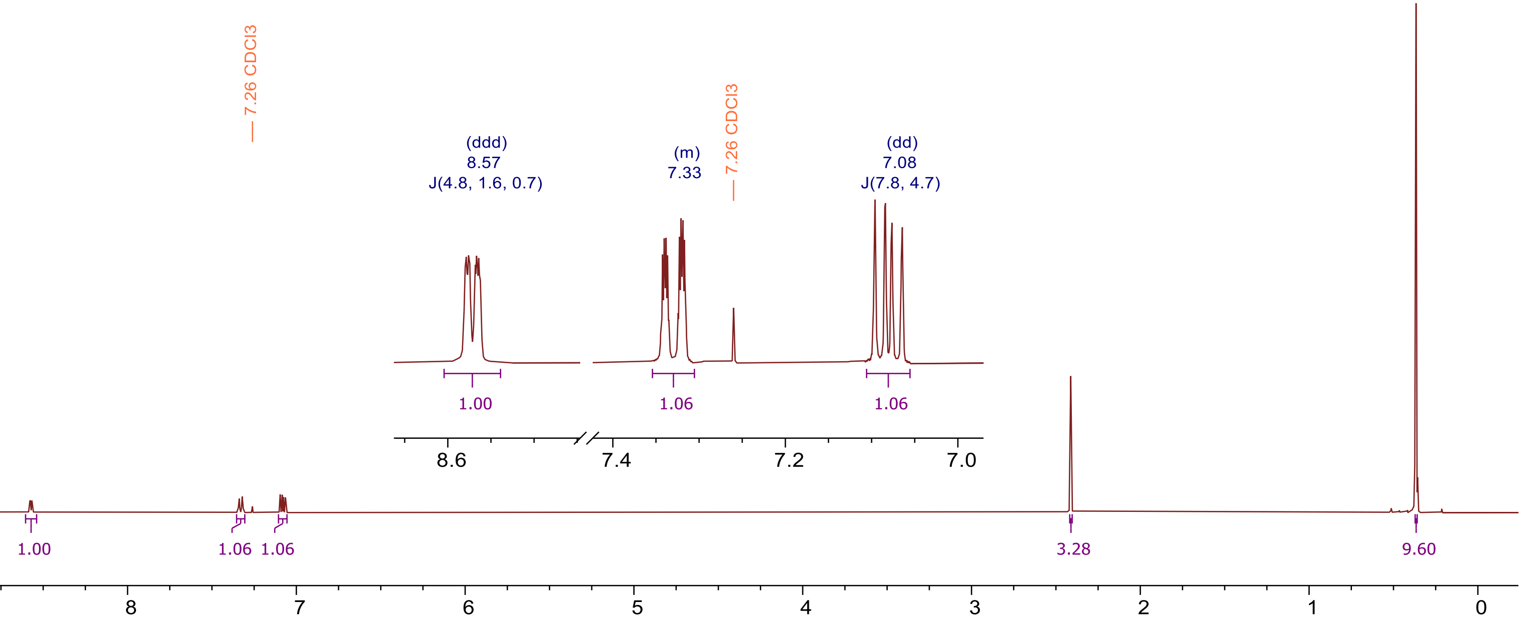

^{13}C NMR (100 MHz, $CDCl_3$):

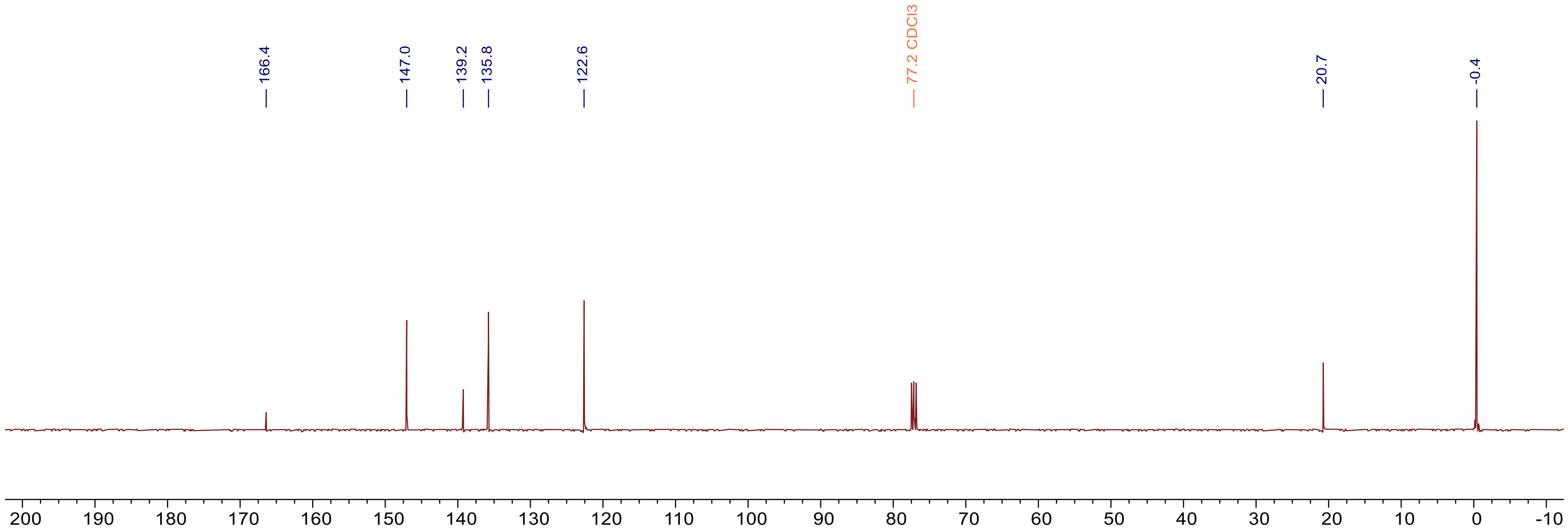

^{1}H NMR (400 MHz, $CDCl_3$):

HetSi_02

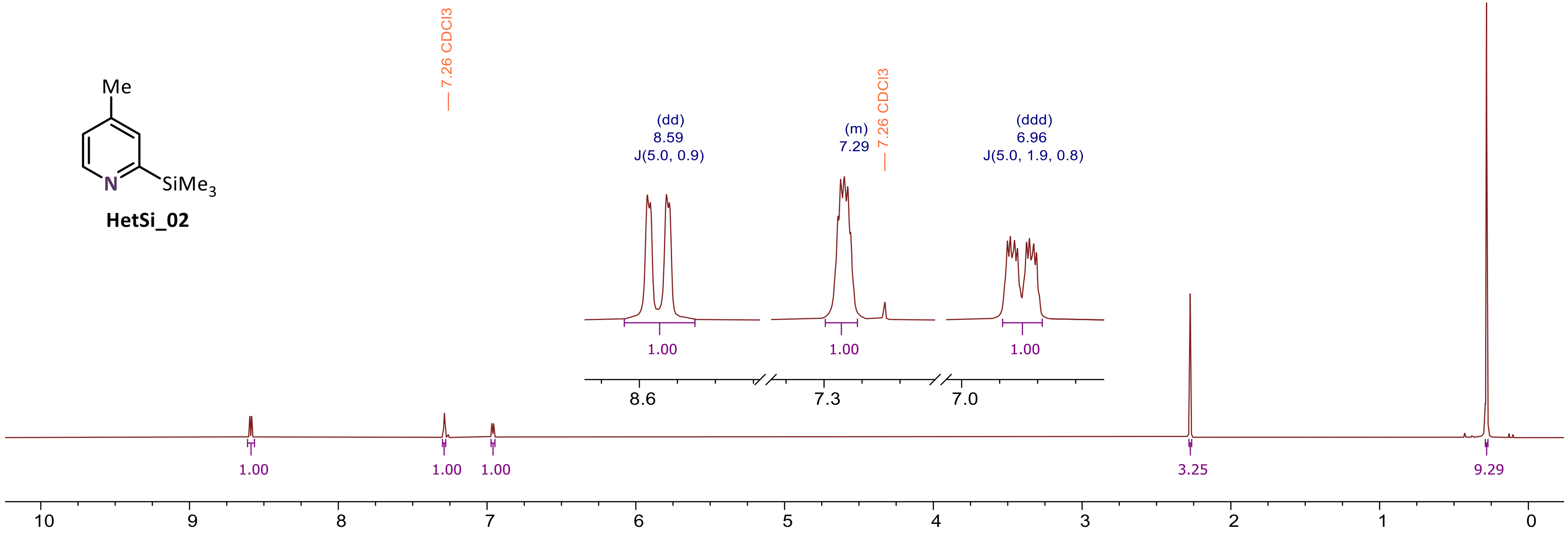

^{13}C NMR (100 MHz, $CDCl_3$):

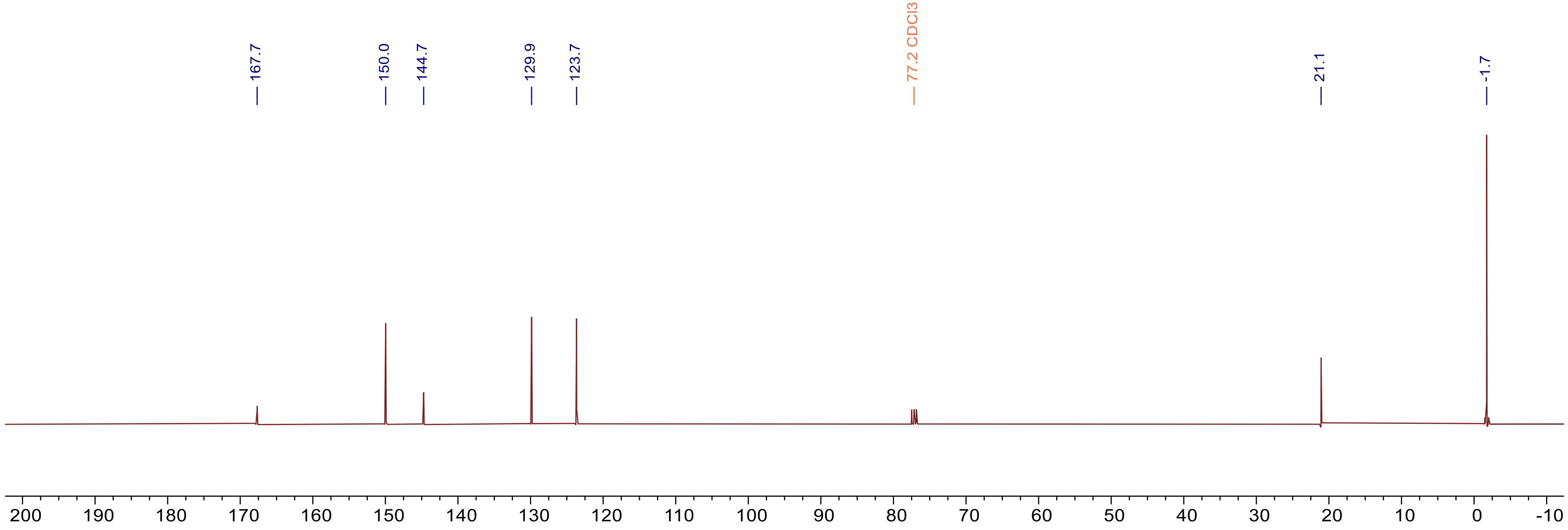

^{1}H NMR (400 MHz, $CDCl_3$):

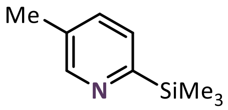

HetSi_03

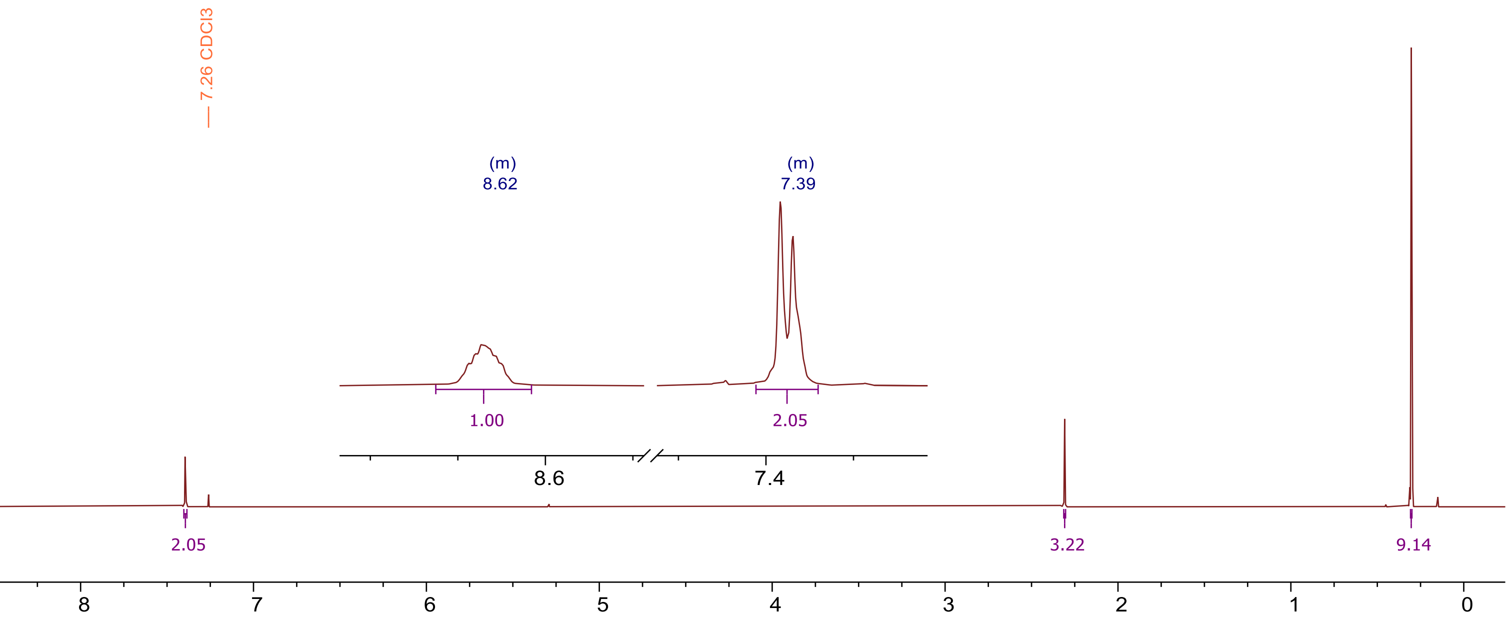

^{13}C NMR (100 MHz, $CDCl_3$):

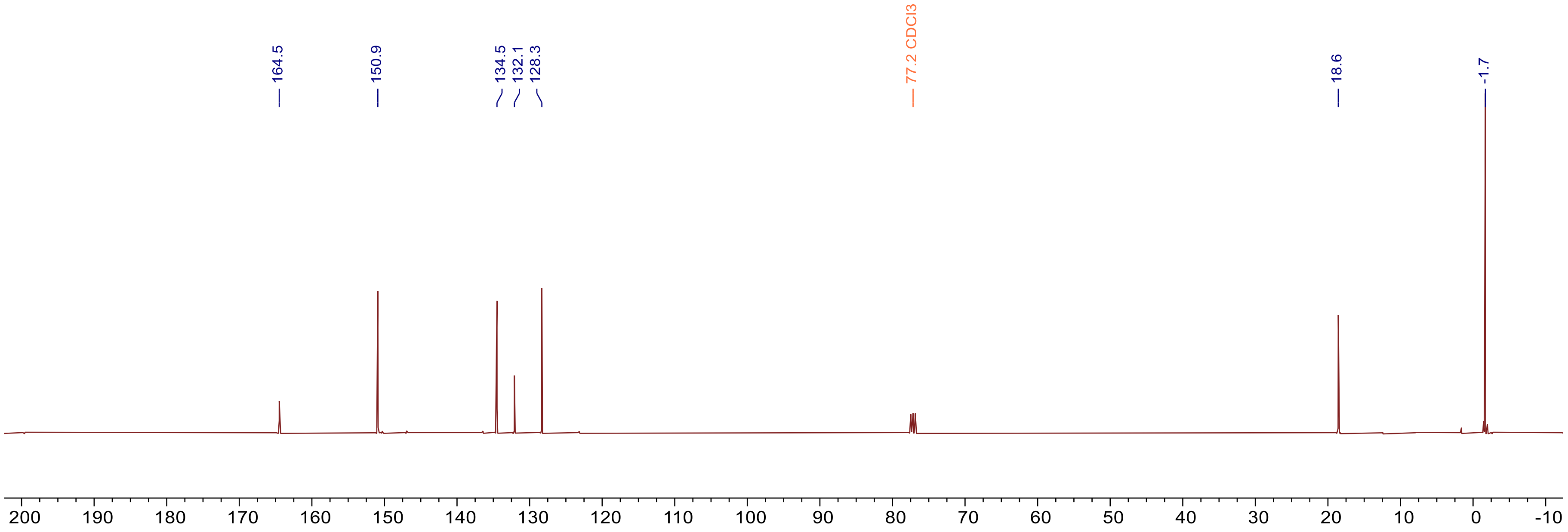

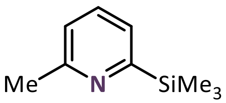

HetSi_04

^{1}H NMR (400 MHz, $CDCl_3$):

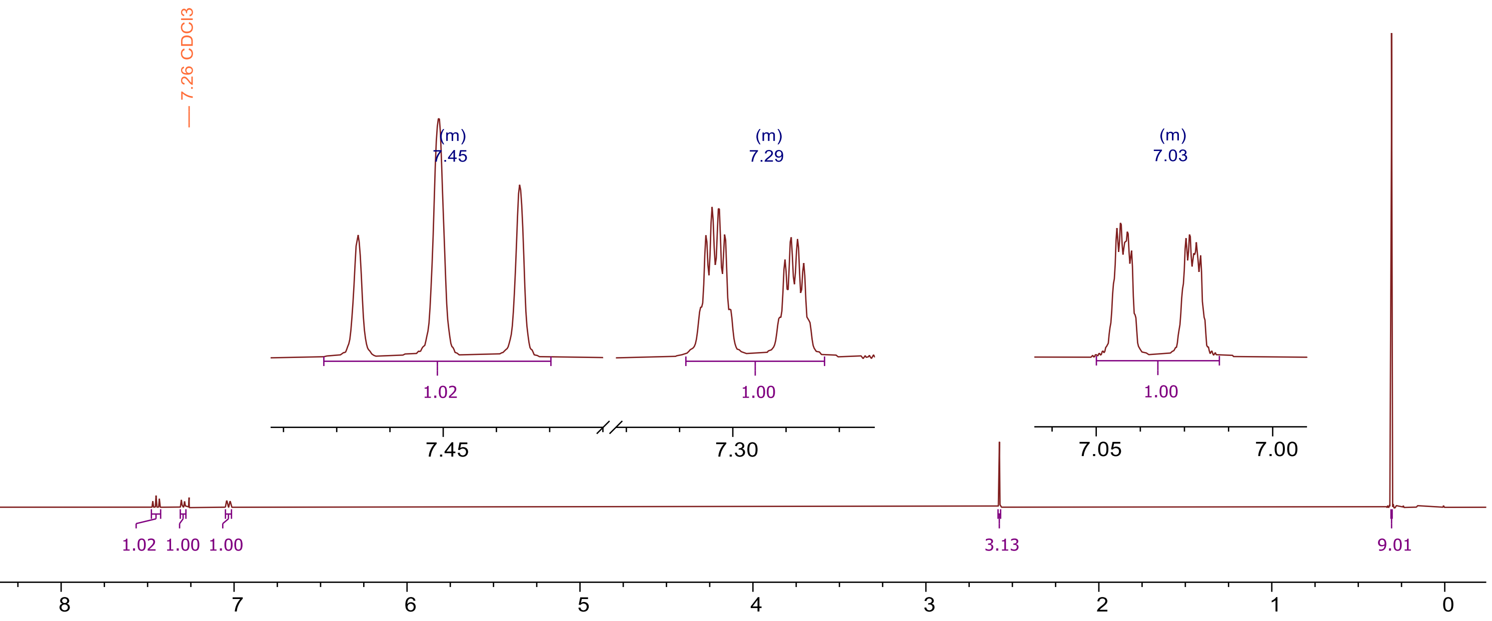

^{13}C NMR (100 MHz, $CDCl_3$):

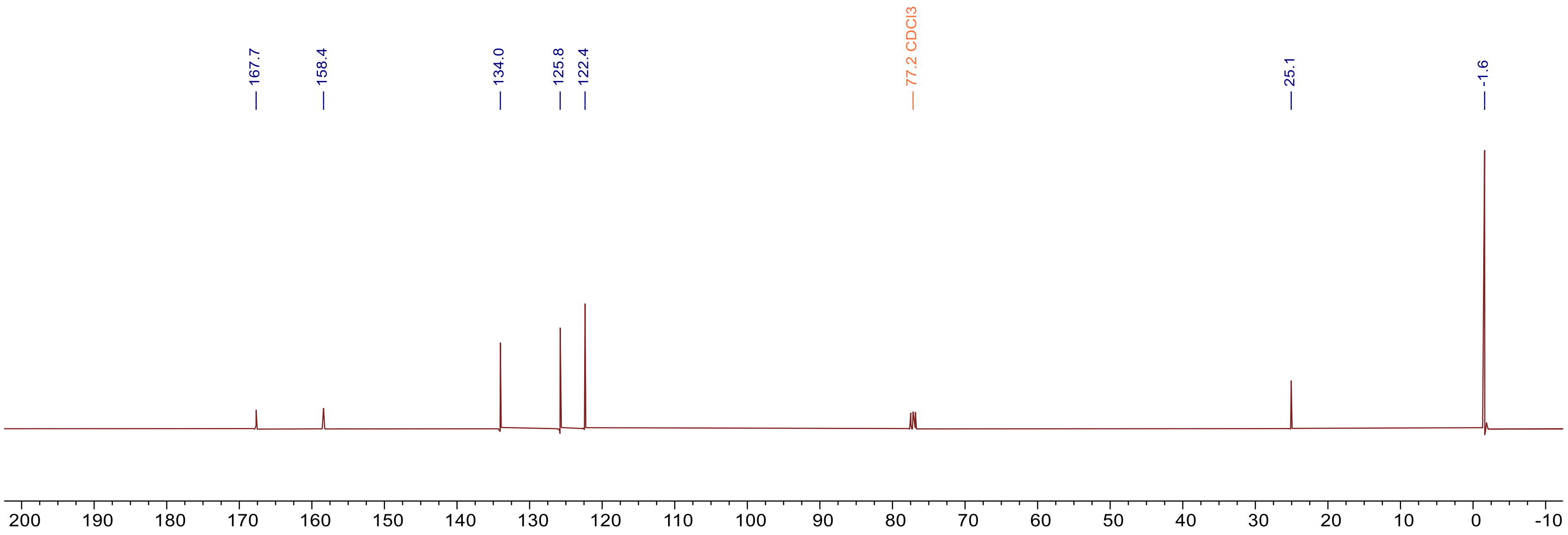

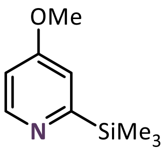

HetSi_05

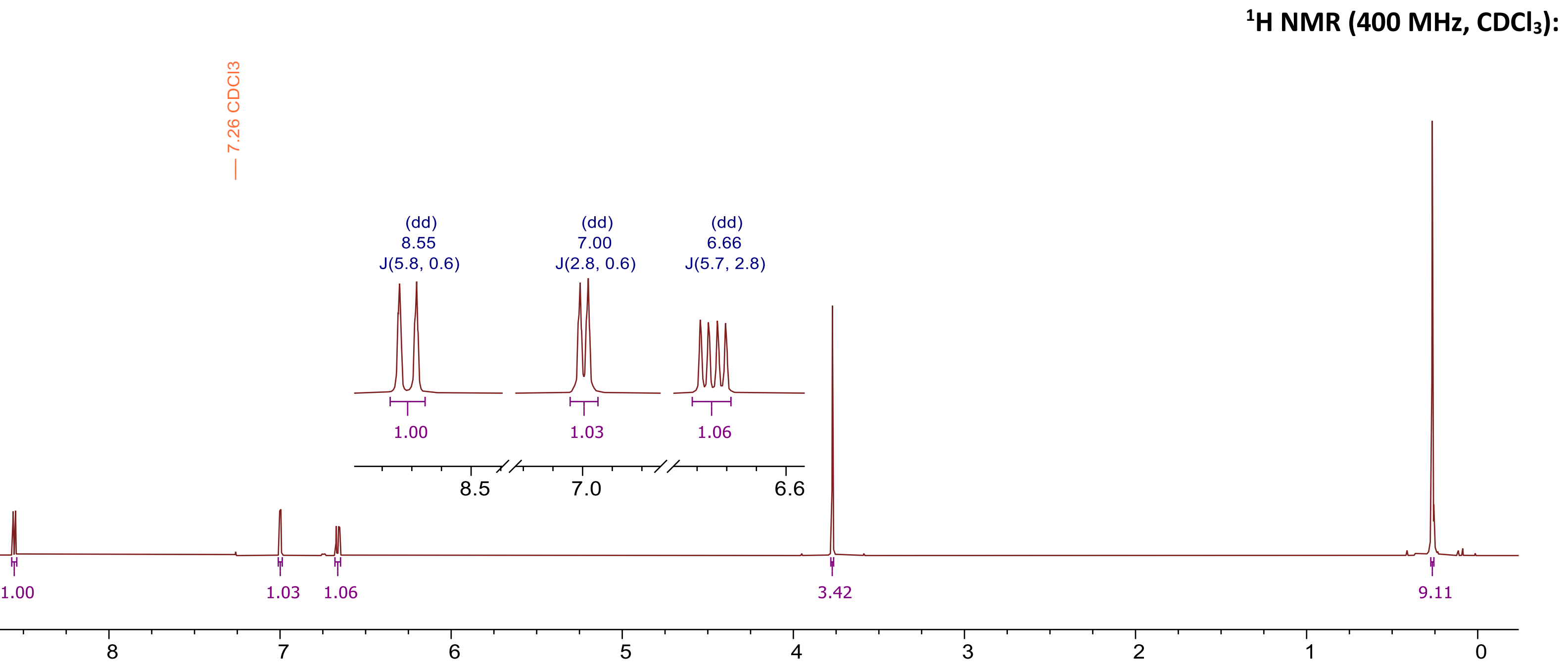

¹H NMR (400 MHz, CDCl₃):
7.26 CDCl3
(dd)
8.55
J(5.8, 0.6)
(dd)
7.00
J(2.8, 0.6)
(dd)
6.66
J(5.7, 2.8)
1.00
1.03
1.06
3.42
9.11

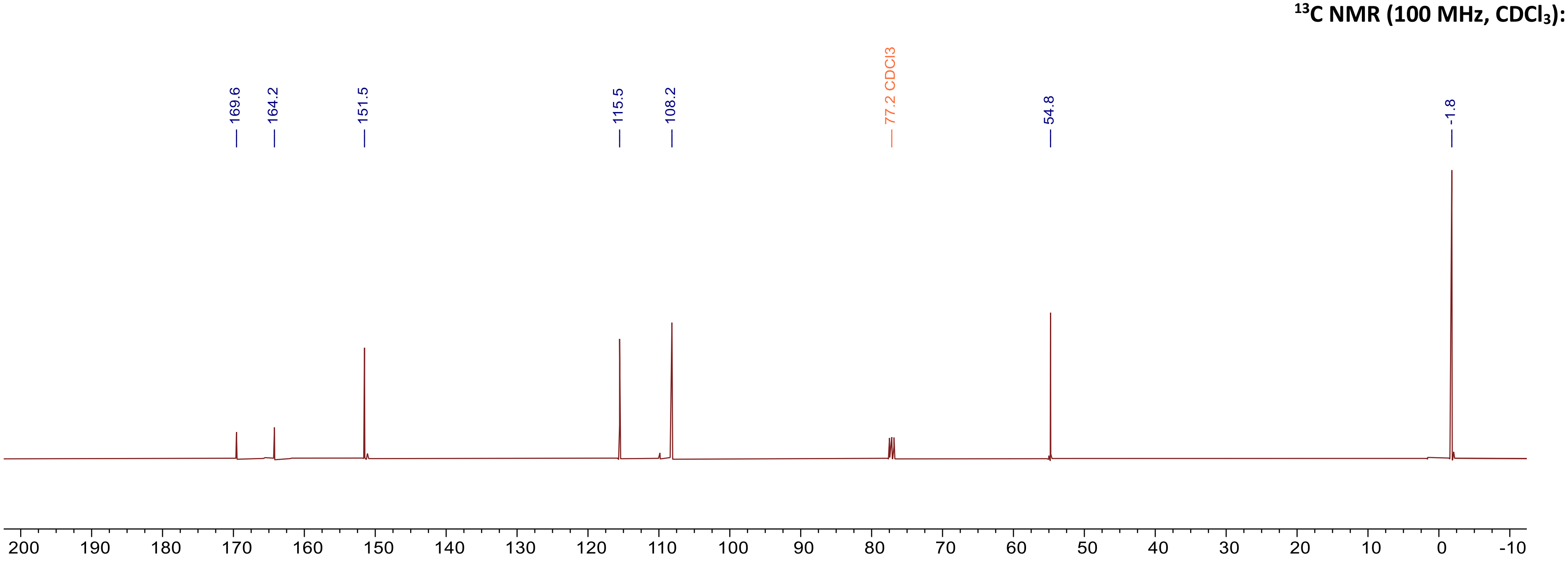

¹³C NMR (100 MHz, CDCl₃):
169.6
164.2
151.5
115.5
108.2
77.2 CDCl3
54.8
-1.8

^{1}H NMR (400 MHz, $CDCl_3$):

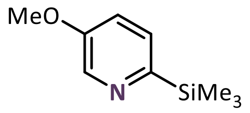

HetSi_06

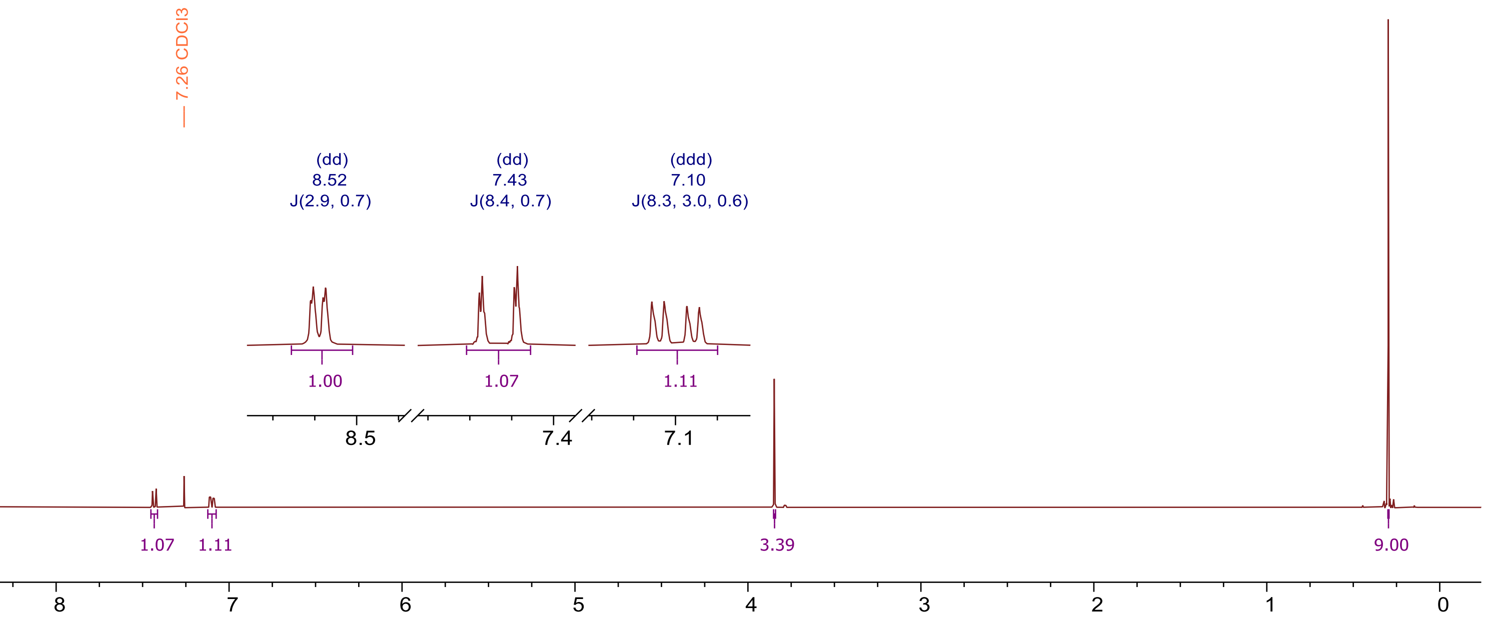

^{13}C NMR (100 MHz, $CDCl_3$):

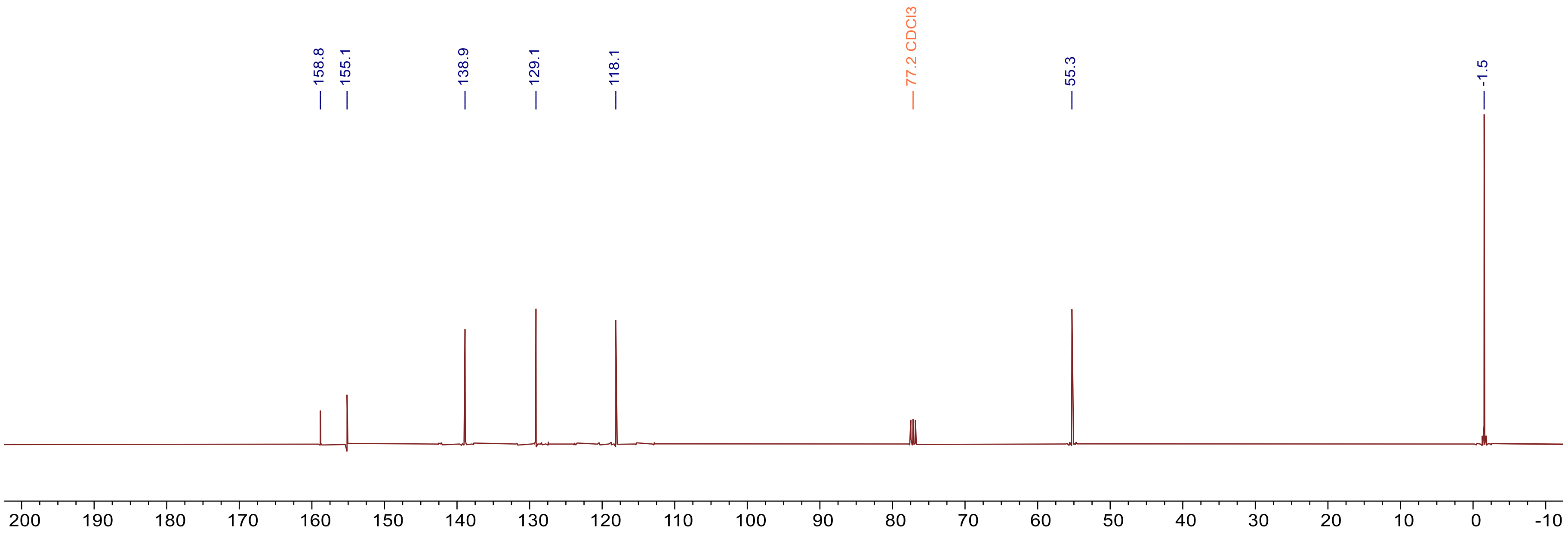

^{1}H NMR (400 MHz, $CDCl_3$):

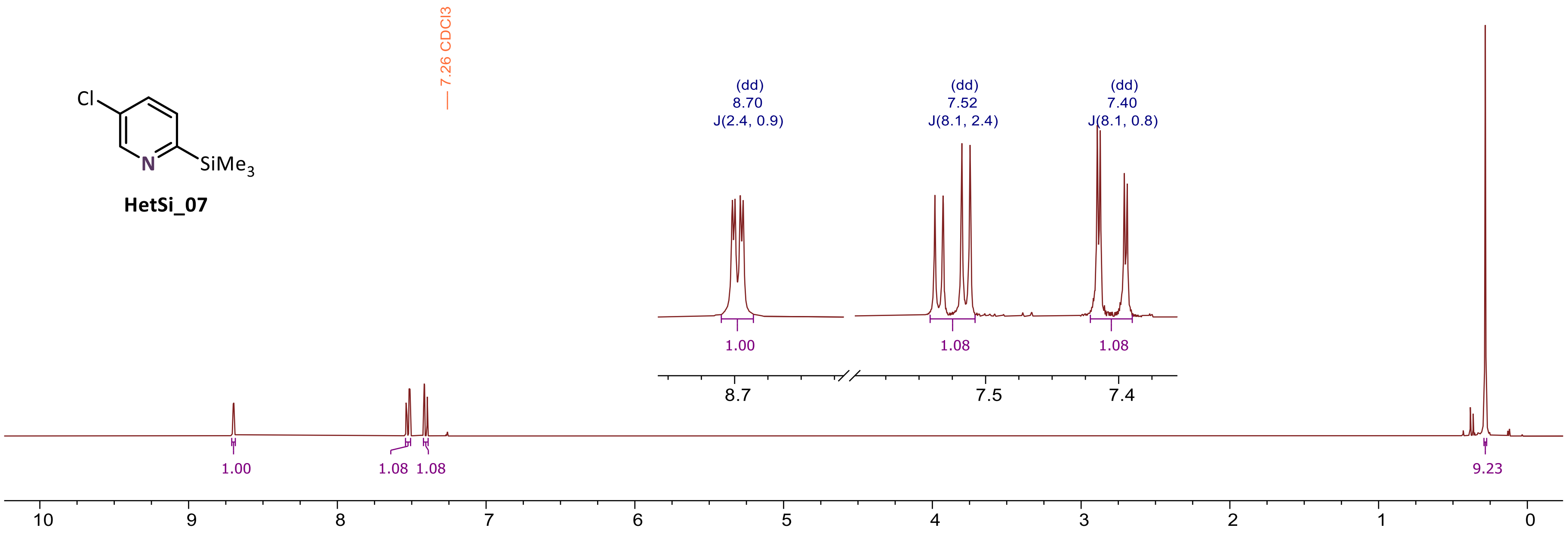

^{13}C NMR (100 MHz, $CDCl_3$):

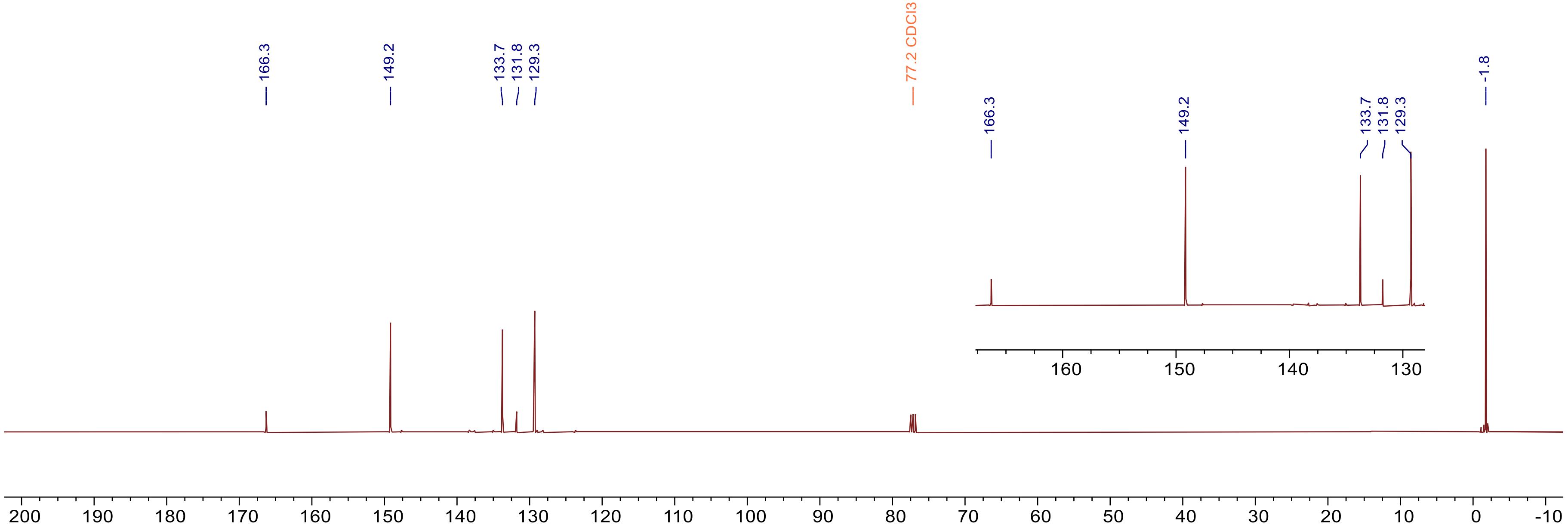

^{1}H NMR (400 MHz, $CDCl_3$):

HetSi_08

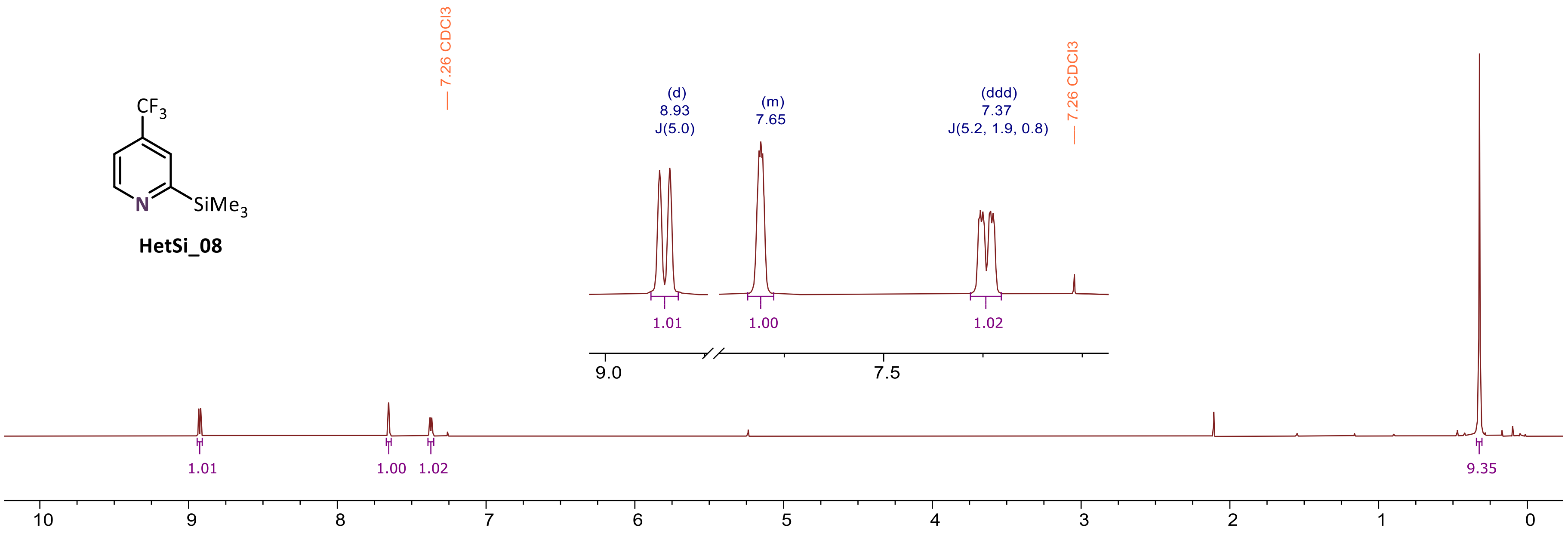

^{13}C NMR (100 MHz, $CDCl_3$):

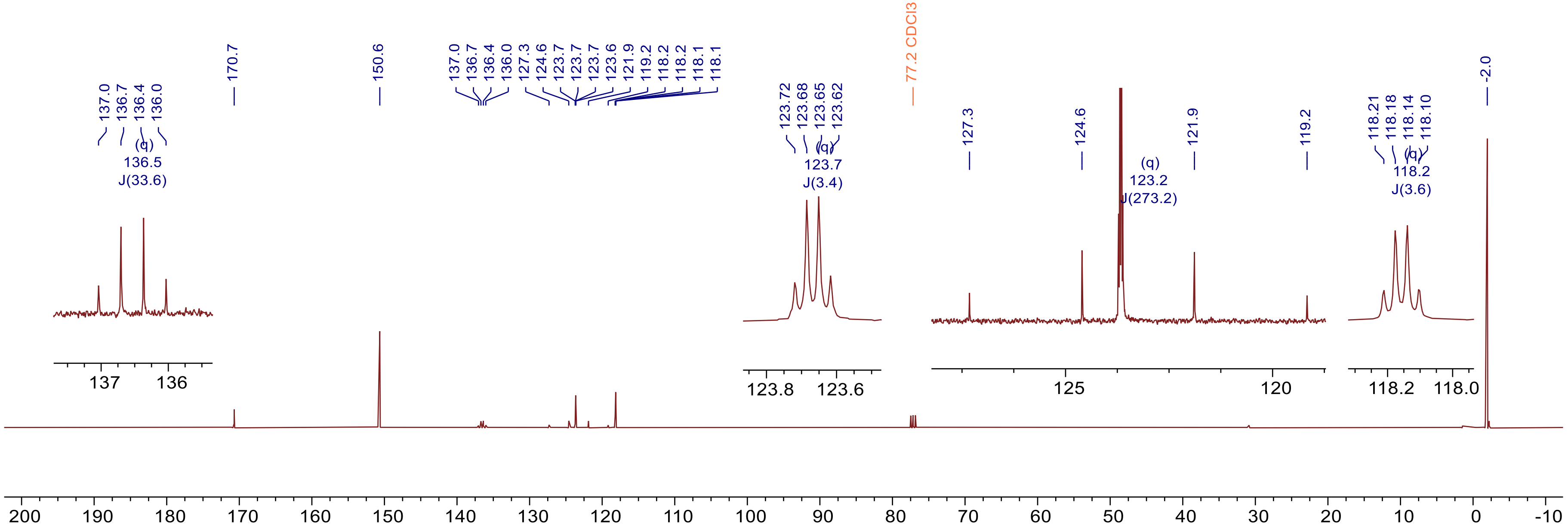

^{19}F NMR (376 MHz, CDCl$_3$):

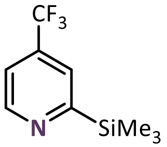

HetSi_08

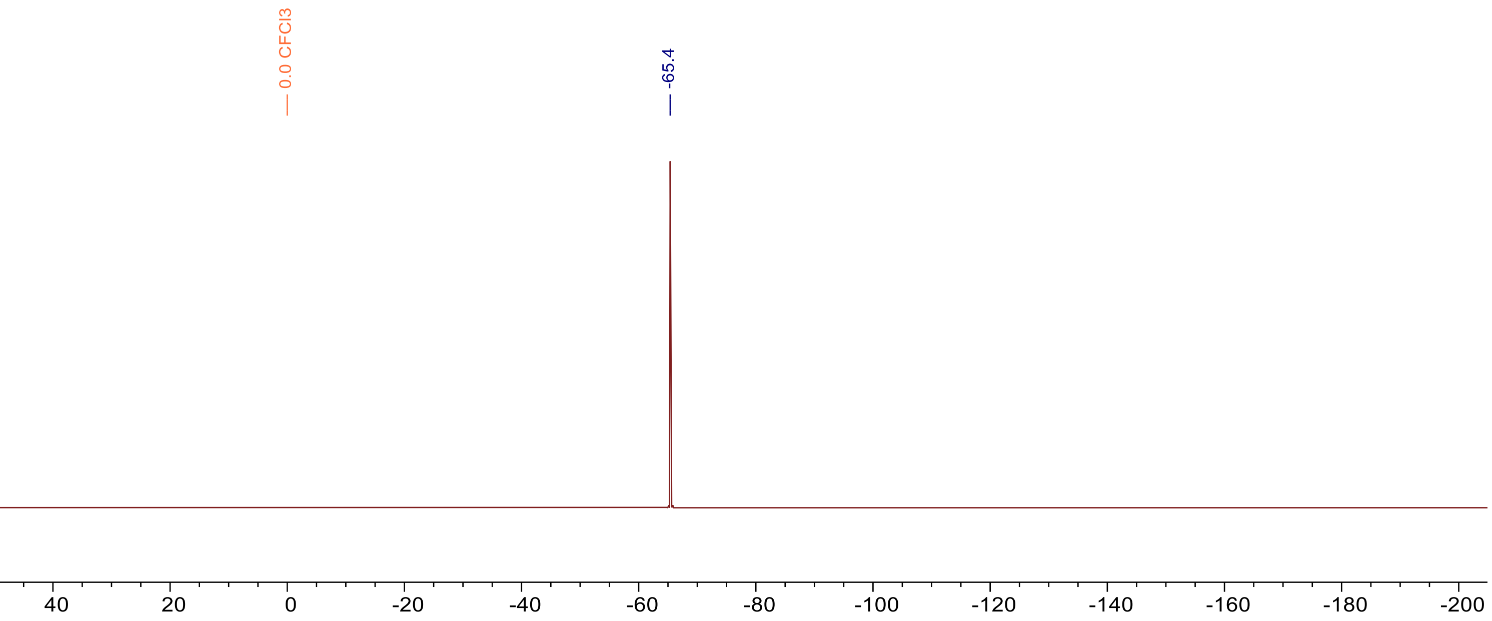

^1H NMR (400 MHz, $CDCl_3$):

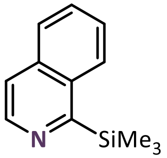

HetSi_09

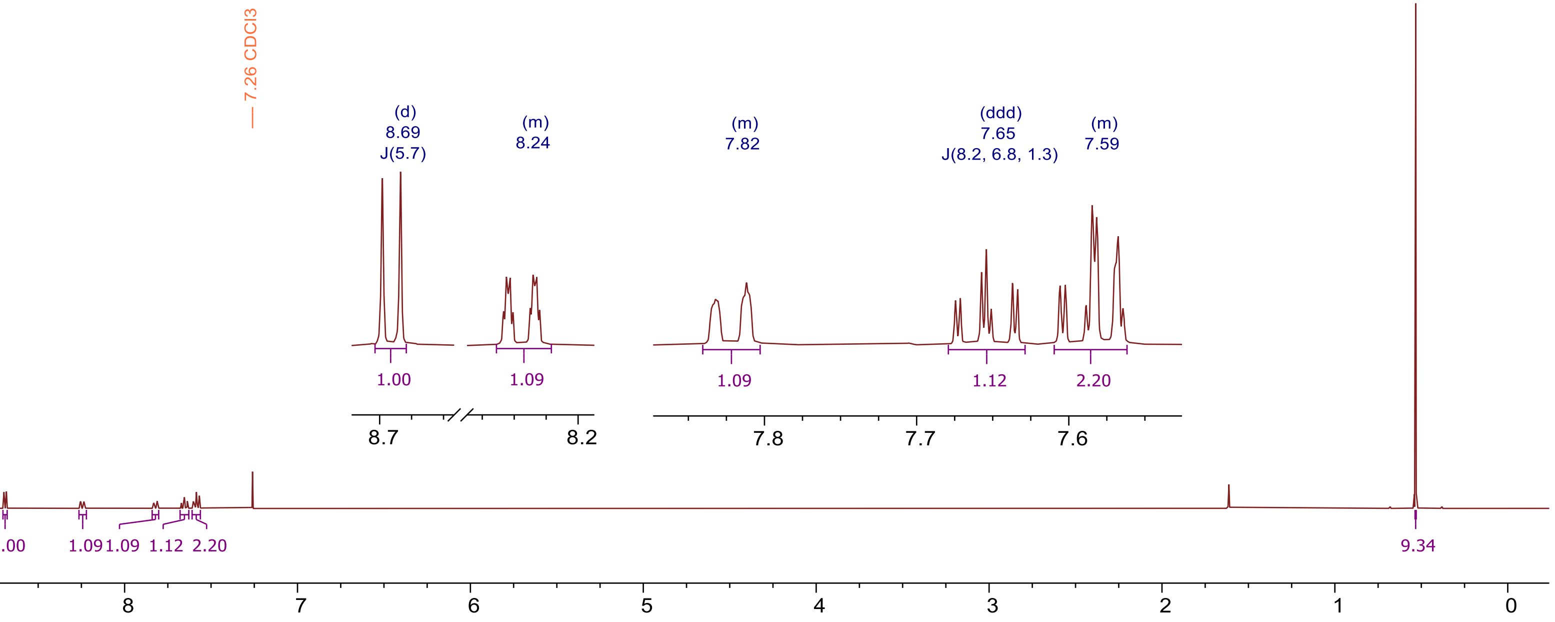

^{13}C NMR (100 MHz, $CDCl_3$):

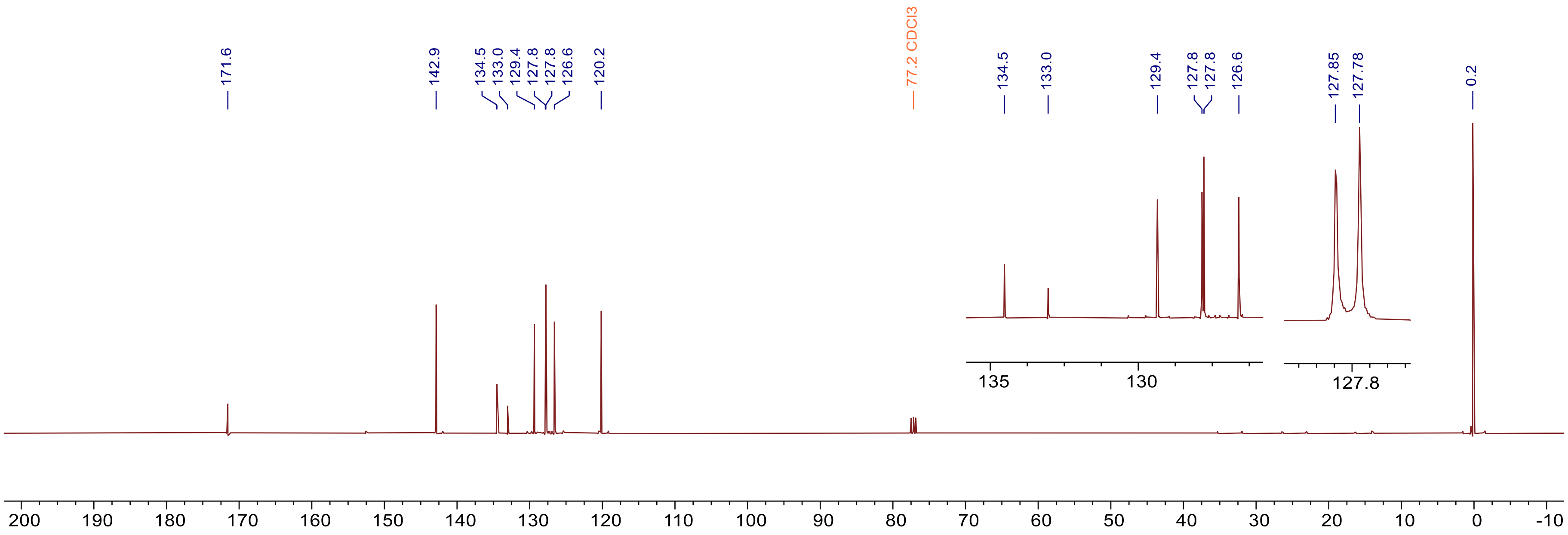

^{1}H NMR (400 MHz, CDCl$_3$):

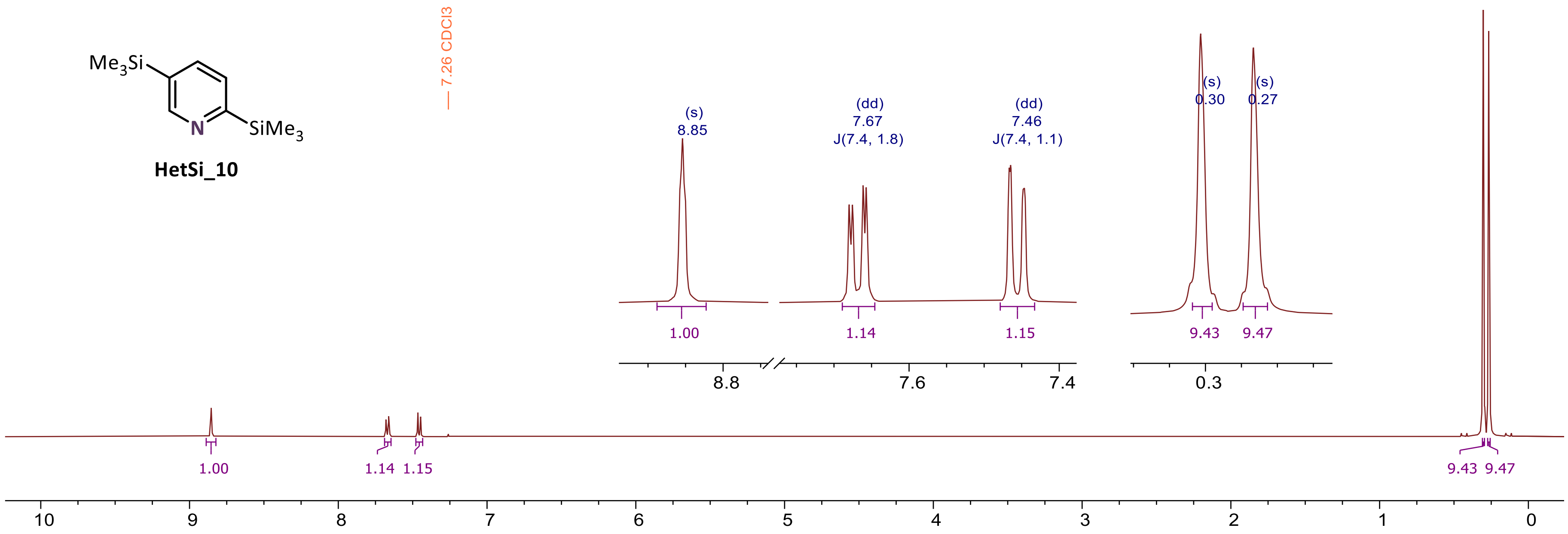

^{13}C NMR (100 MHz, CDCl$_3$):

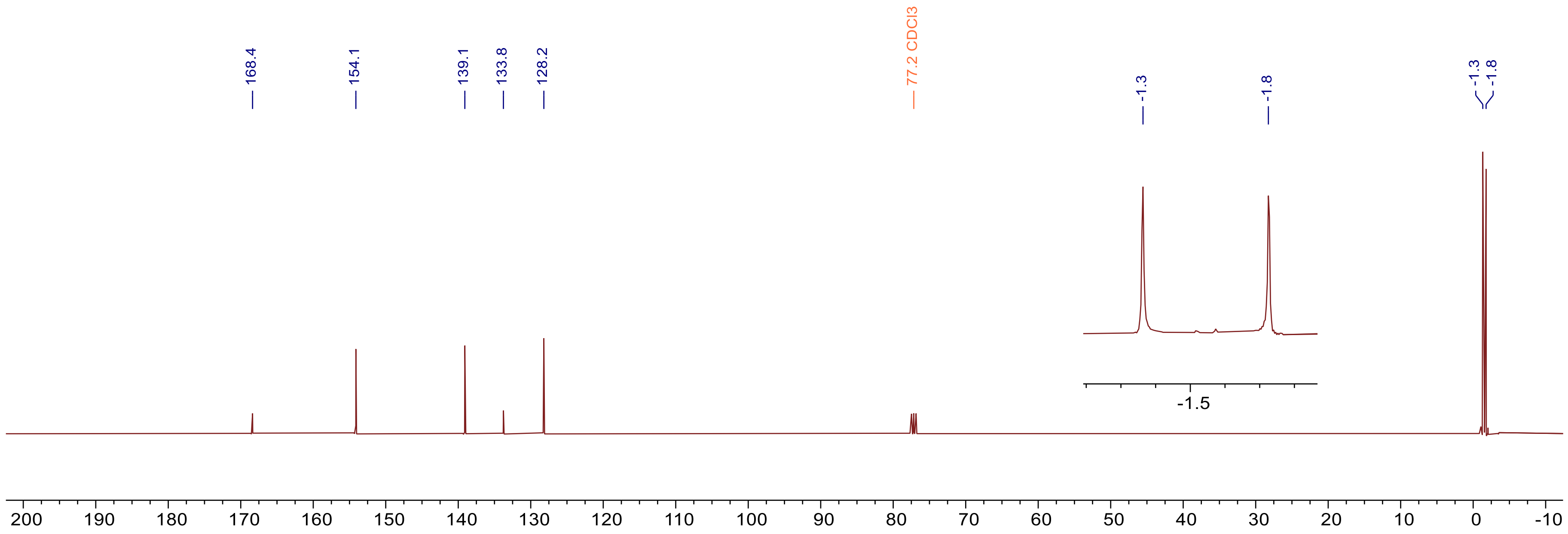

^{1}H NMR (400 MHz, $CDCl_3$):

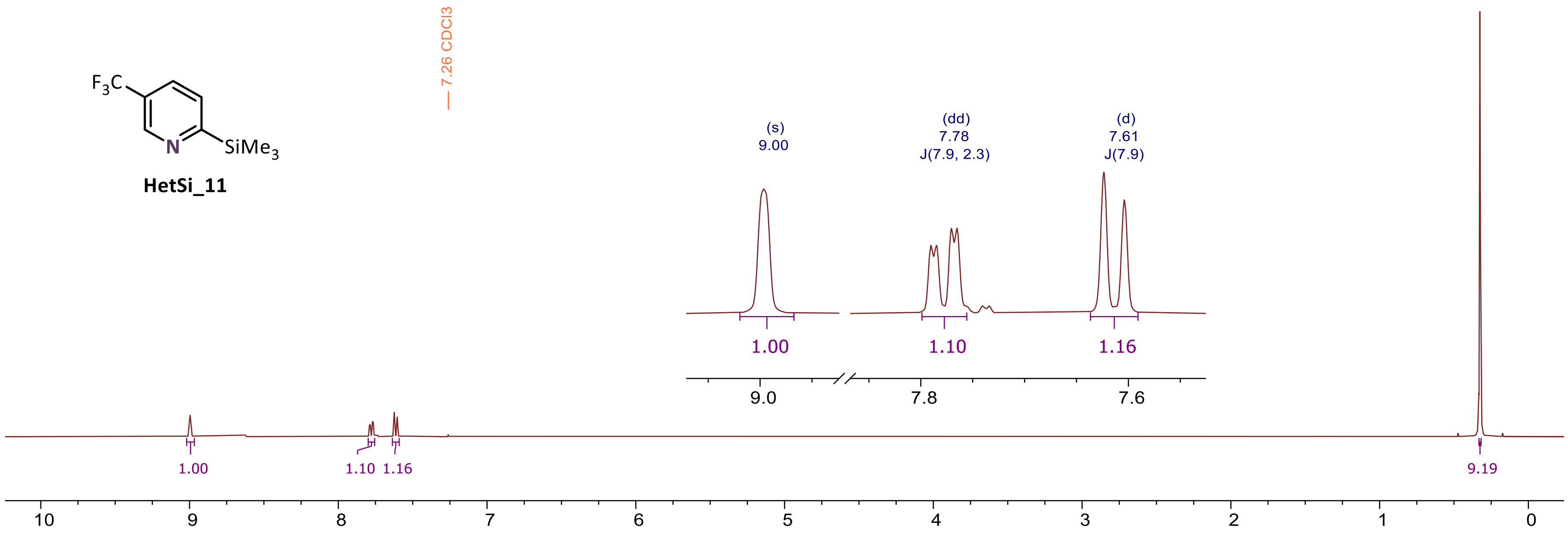

^{13}C NMR (100 MHz, $CDCl_3$):

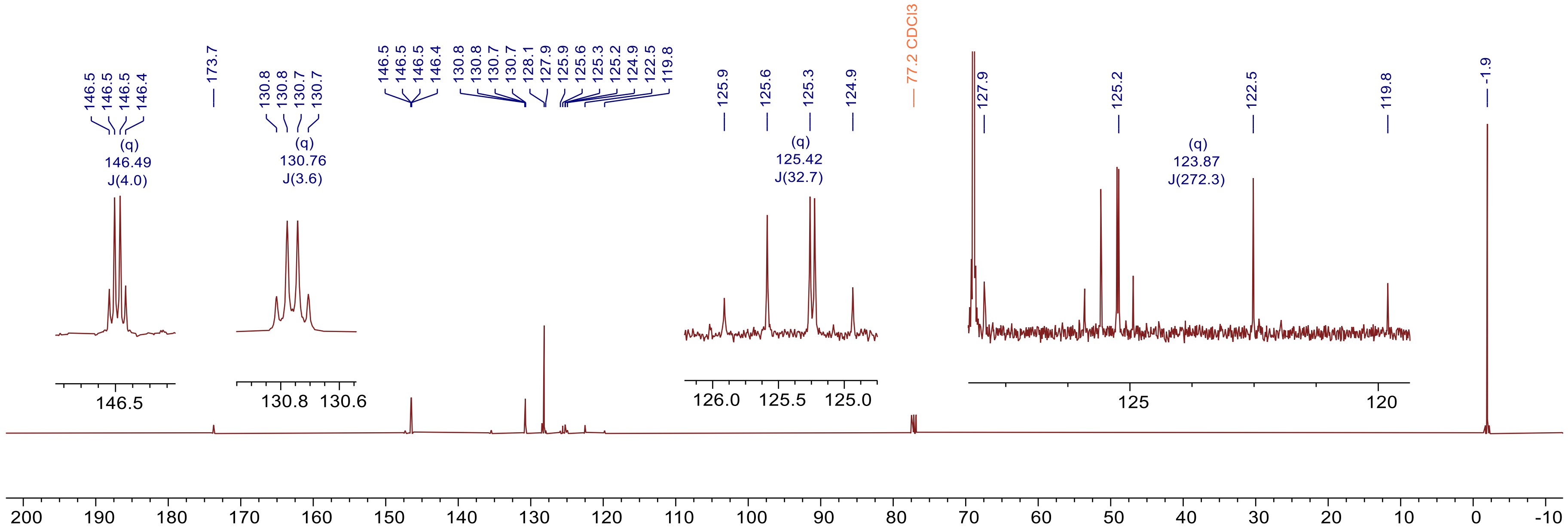

^{19}F NMR (376 MHz, $CDCl_3$):

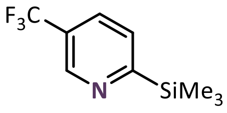

HetSi_11

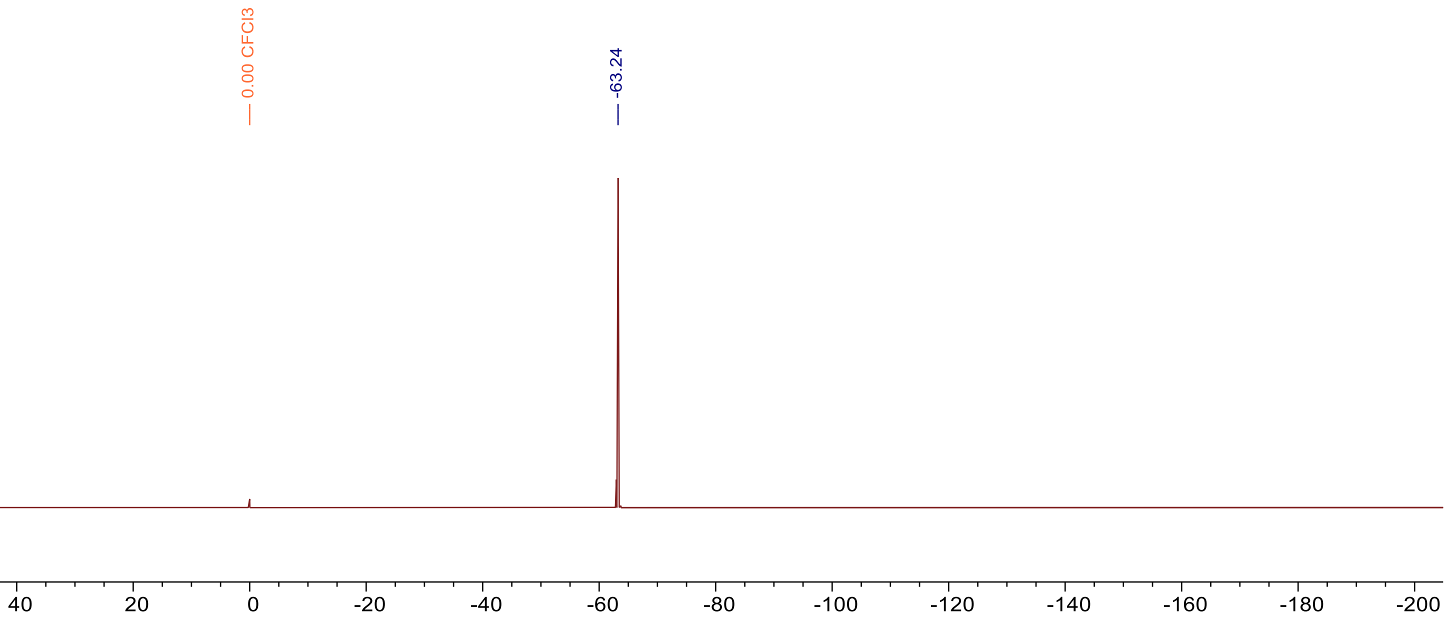

^{1}H NMR (400 MHz, $CDCl_3$):

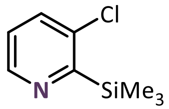

HetSi_12

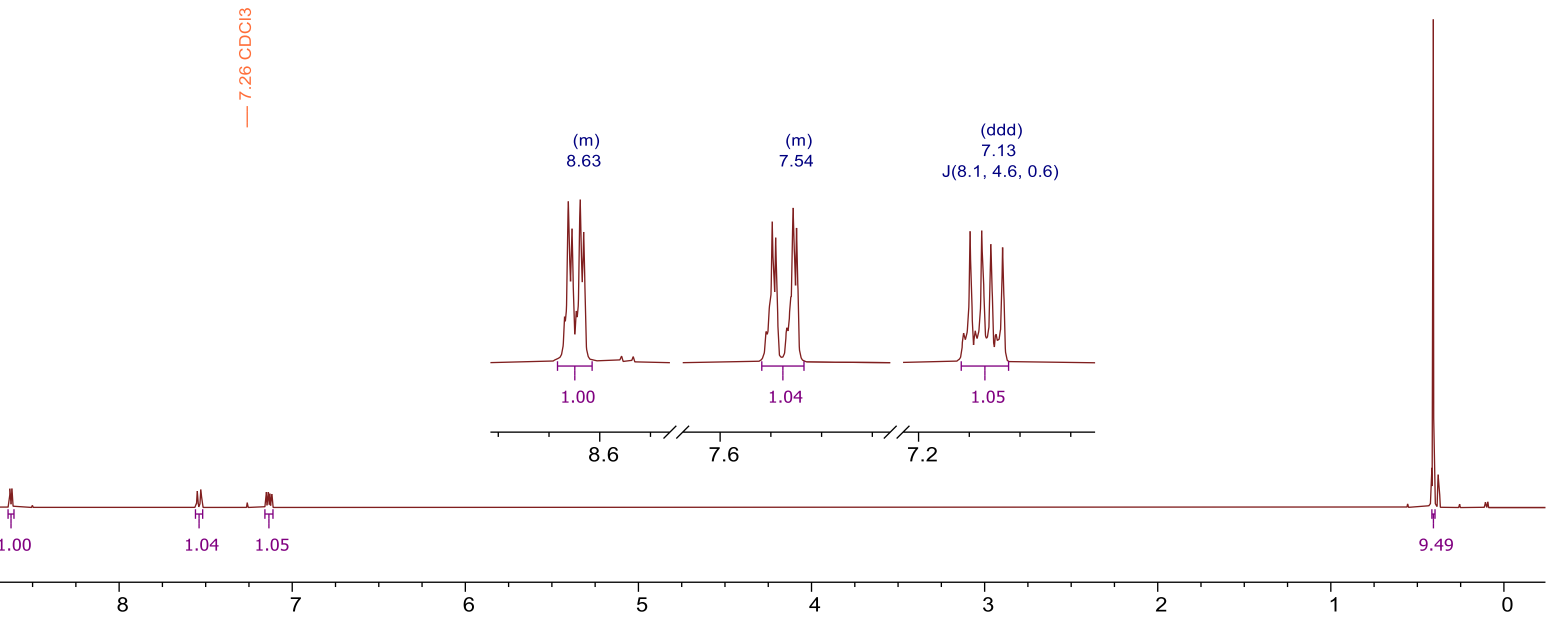

^{13}C NMR (100 MHz, $CDCl_3$):

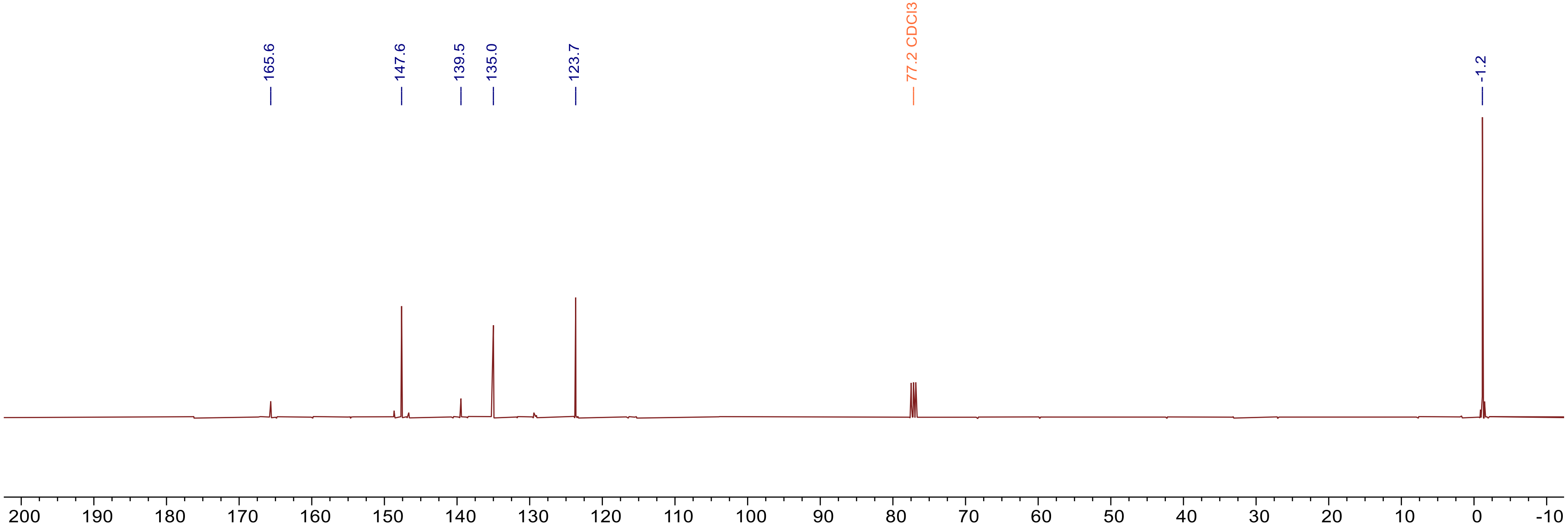

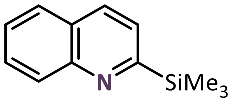

HetSi_13

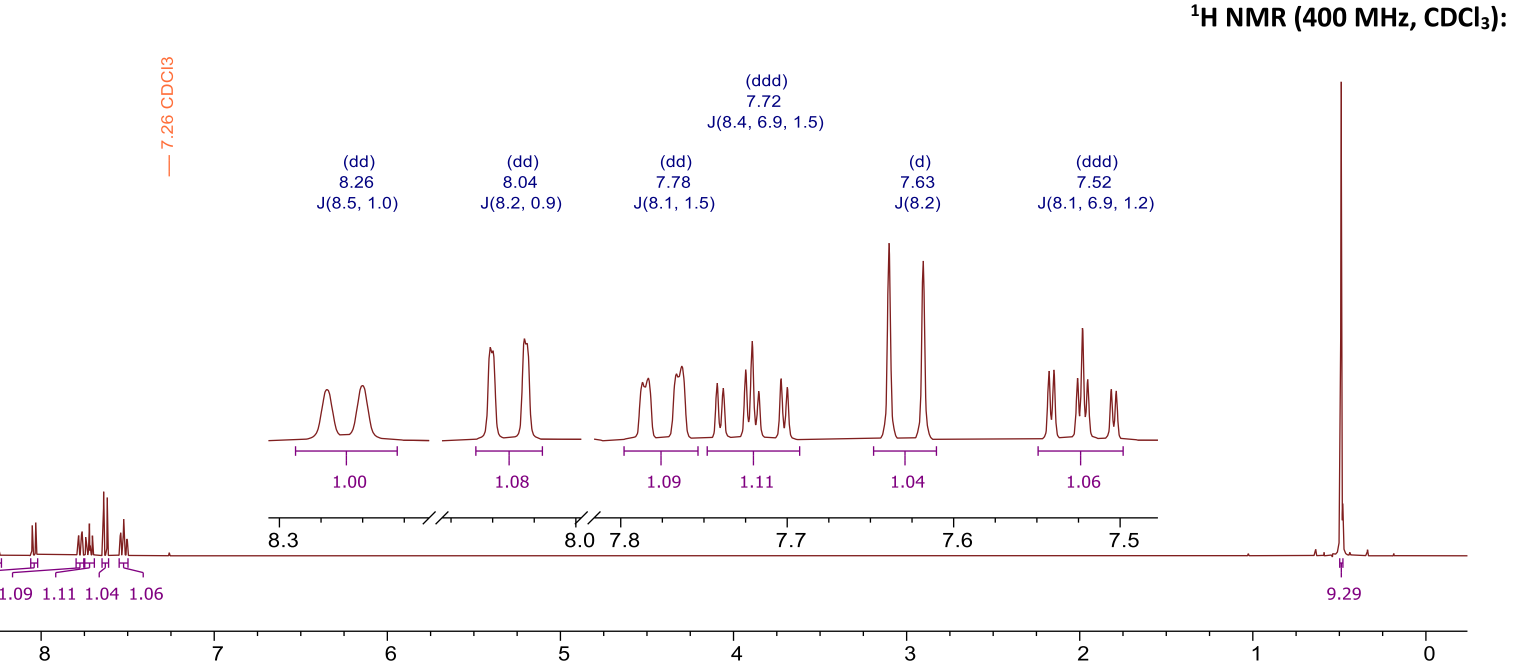

¹H NMR (400 MHz, CDCl₃):
7.26 CDCl3
(dd) 8.26 J(8.5, 1.0)
(dd) 8.04 J(8.2, 0.9)
(dd) 7.78 J(8.1, 1.5)
(ddd) 7.72 J(8.4, 6.9, 1.5)
(d) 7.63 J(8.2)
(ddd) 7.52 J(8.1, 6.9, 1.2)
1.00 1.08 1.09 1.11 1.04 1.06
9.29

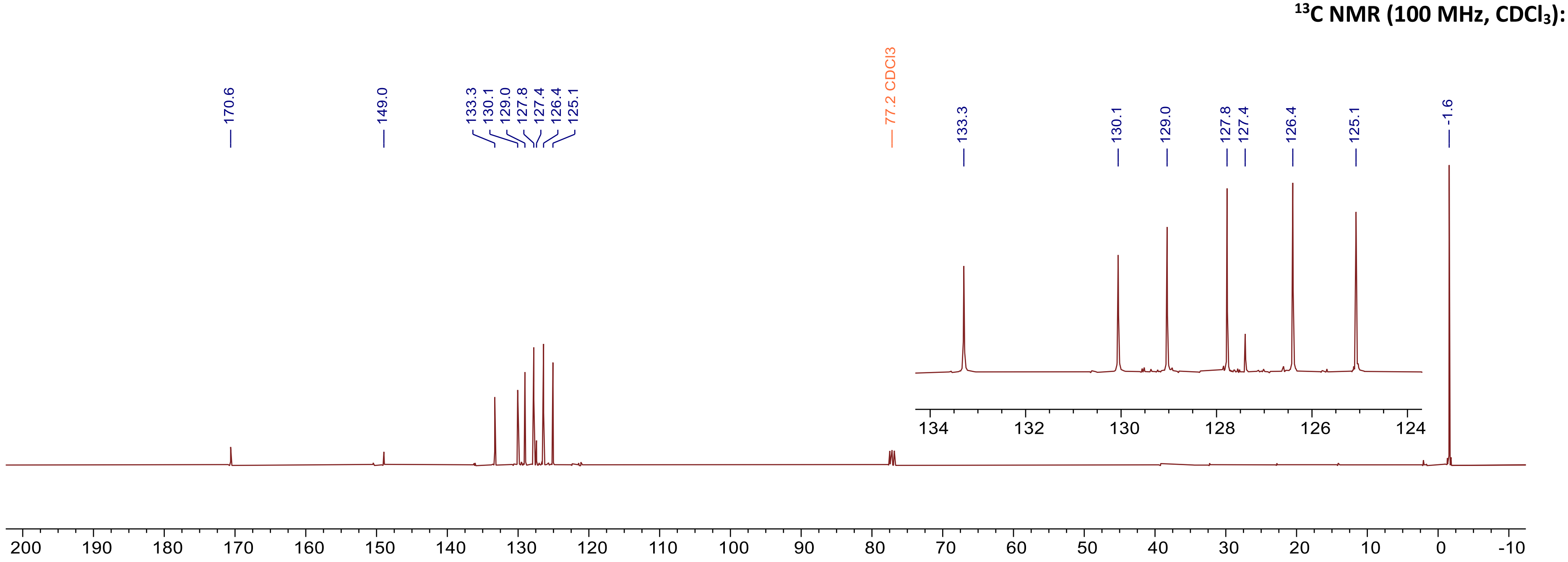

¹³C NMR (100 MHz, CDCl₃):
170.6
149.0
133.3 130.1 129.0 127.8 127.4 126.4 125.1
77.2 CDCl3
-1.6

^{1}H NMR (400 MHz, $CDCl_3$):

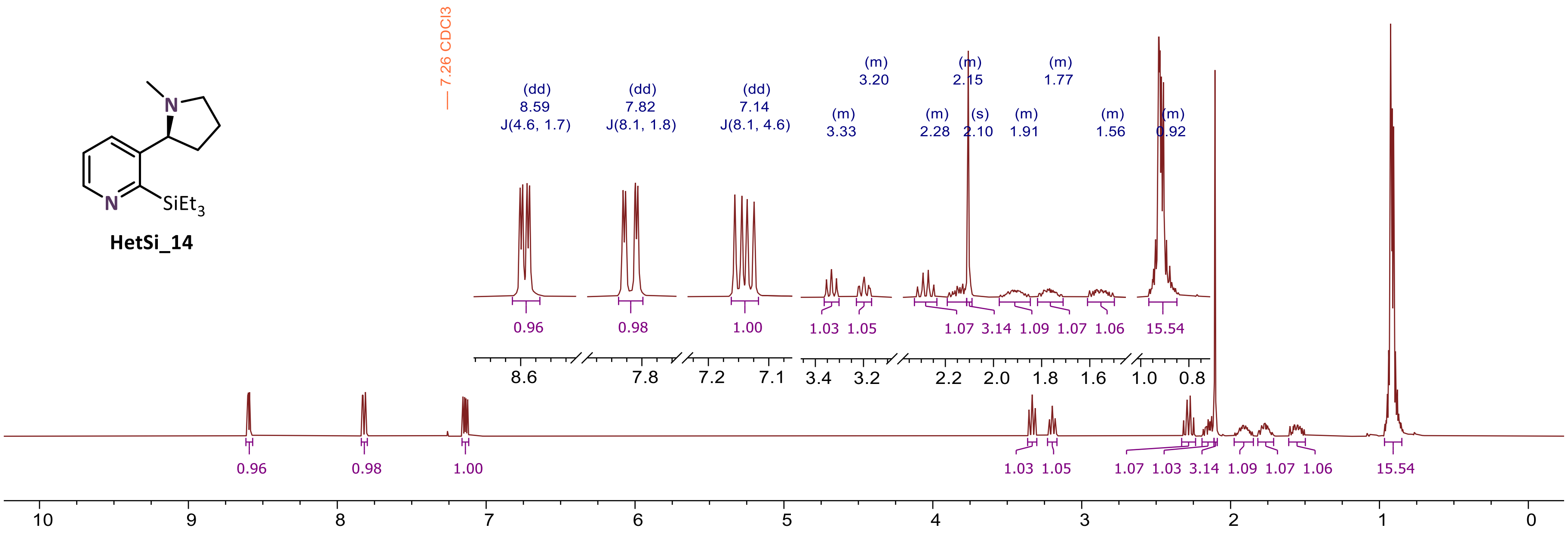

HetSi_14

^{13}C NMR (100 MHz, $CDCl_3$):

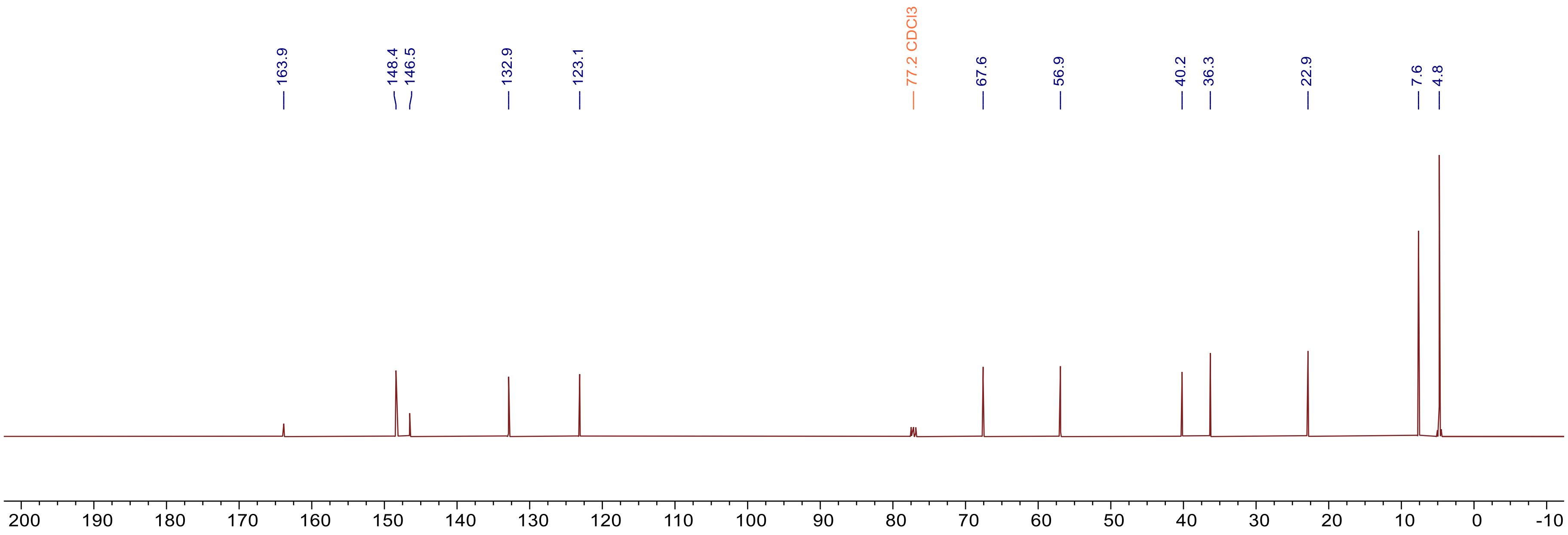

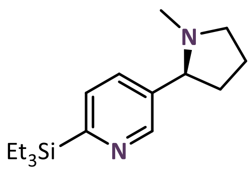
N
Et3Si
N

HetSi_15

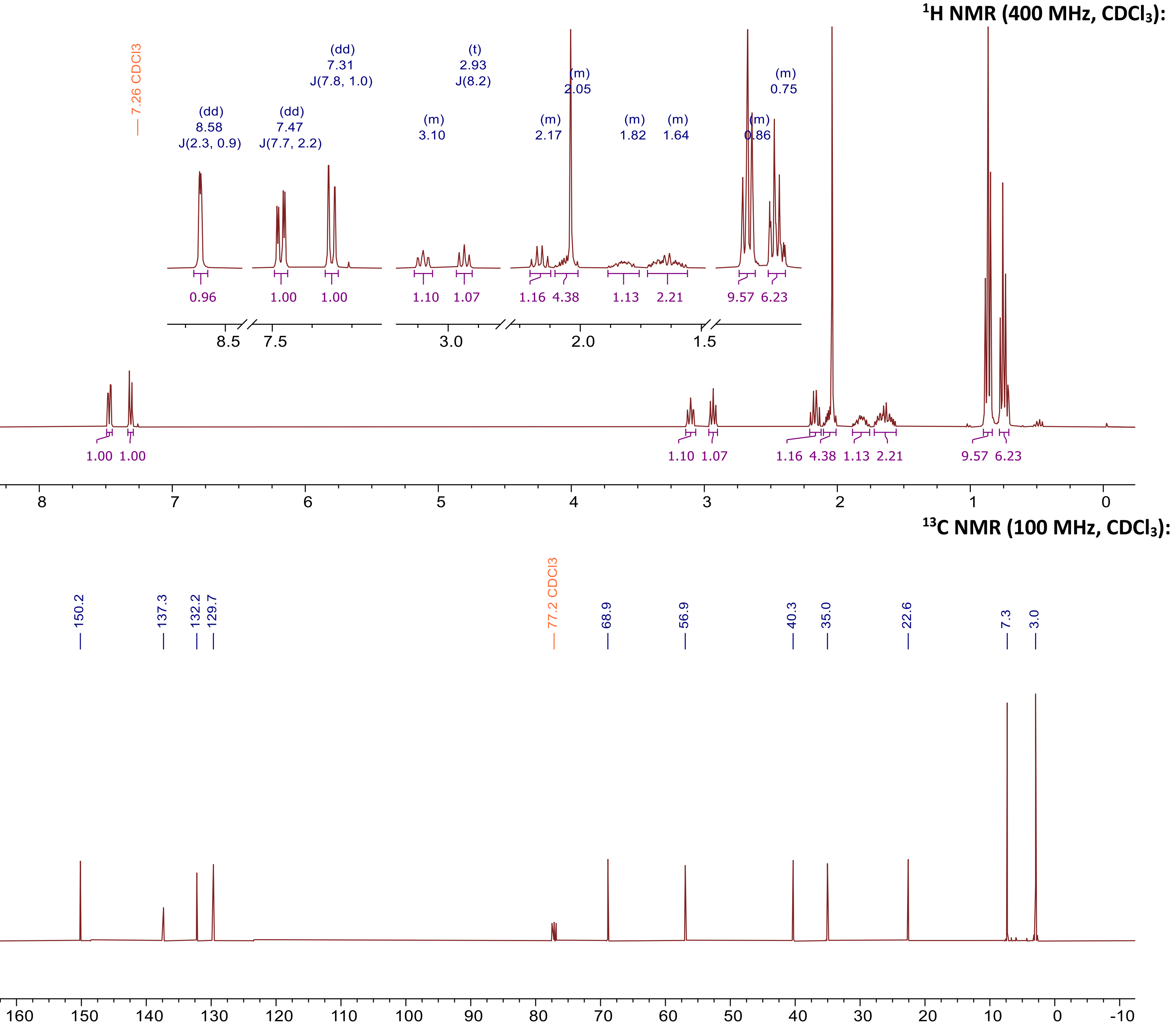
1H NMR (400 MHz, CDCl3):
7.26 CDCl3
(dd) 8.58 J(2.3, 0.9)
(dd) 7.47 J(7.7, 2.2)
(dd) 7.31 J(7.8, 1.0)
(m) 3.10
(t) 2.93 J(8.2)
(m) 2.17
(m) 2.05
(m) 1.82
(m) 1.64
(m) 0.86
(m) 0.75
0.96 1.00 1.00 1.10 1.07 1.16 4.38 1.13 2.21 9.57 6.23
13C NMR (100 MHz, CDCl3):
164.7
150.2
137.3
132.2
129.7
77.2 CDCl3
68.9
56.9
40.3
35.0
22.6
7.3
3.0

^{1}H NMR (400 MHz, $CDCl_3$):

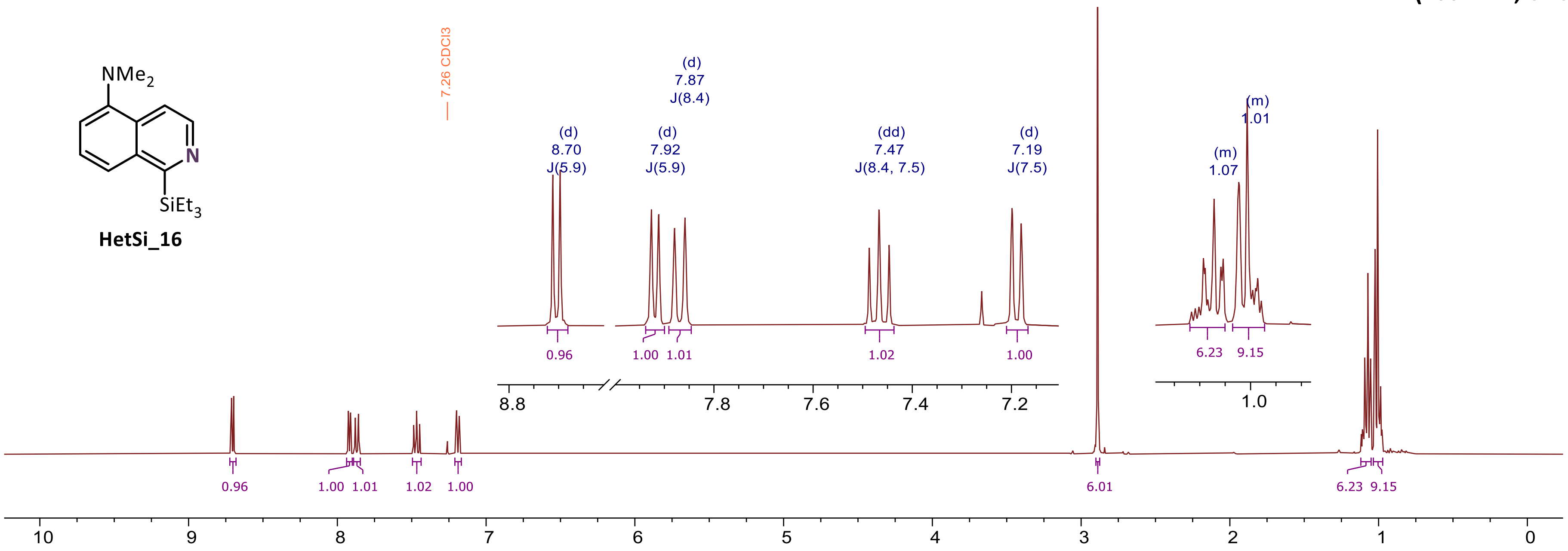

^{13}C NMR (100 MHz, $CDCl_3$):

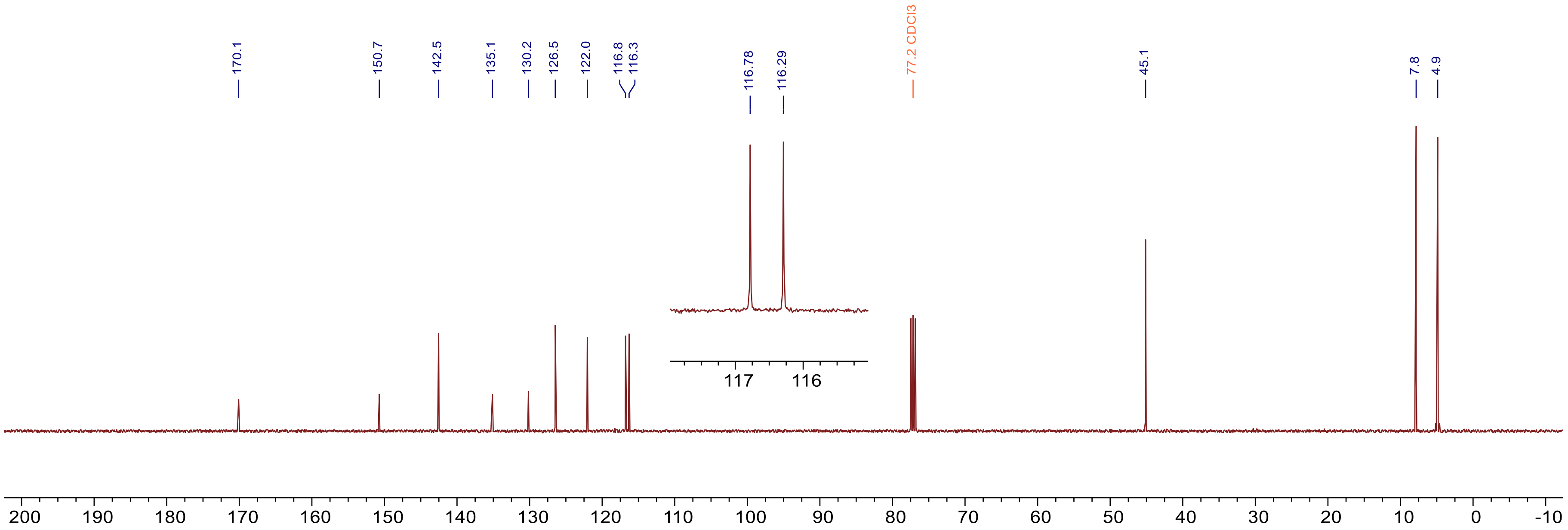

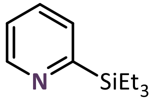

HetSi_17

^{1}H NMR (400 MHz, CDCl$_3$):

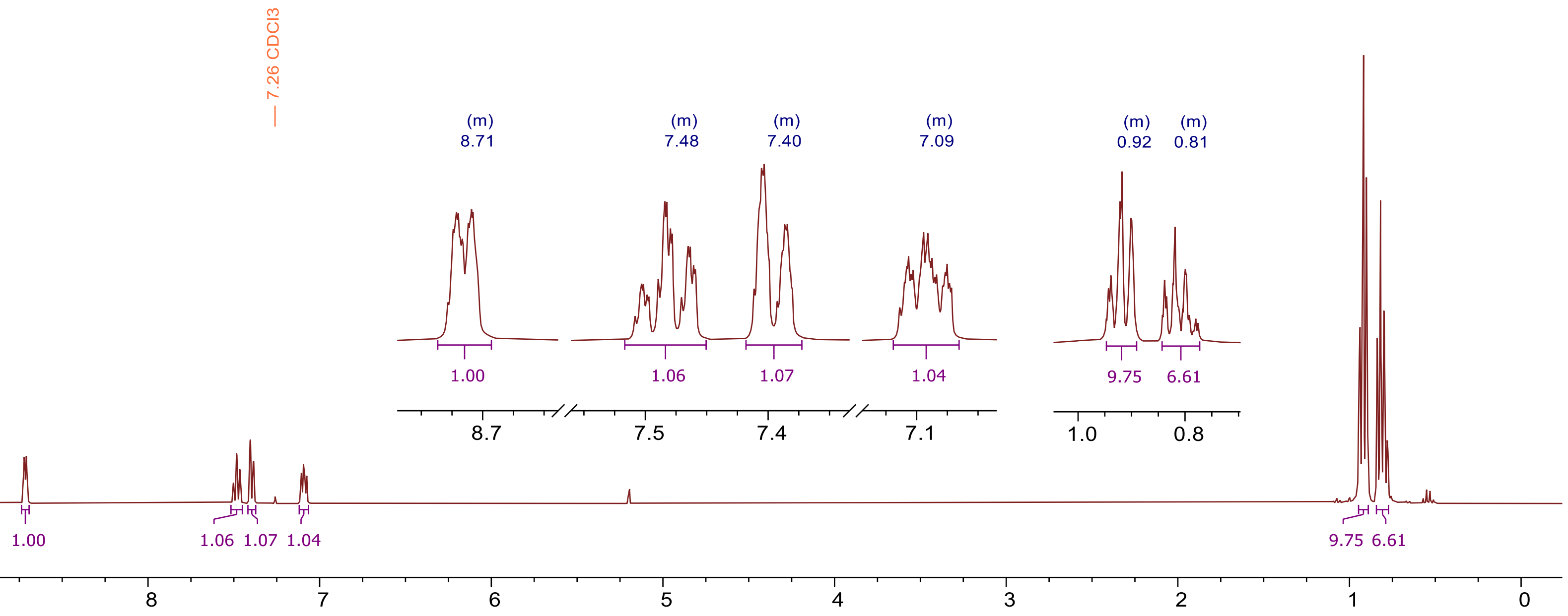

^{13}C NMR (100 MHz, CDCl$_3$):

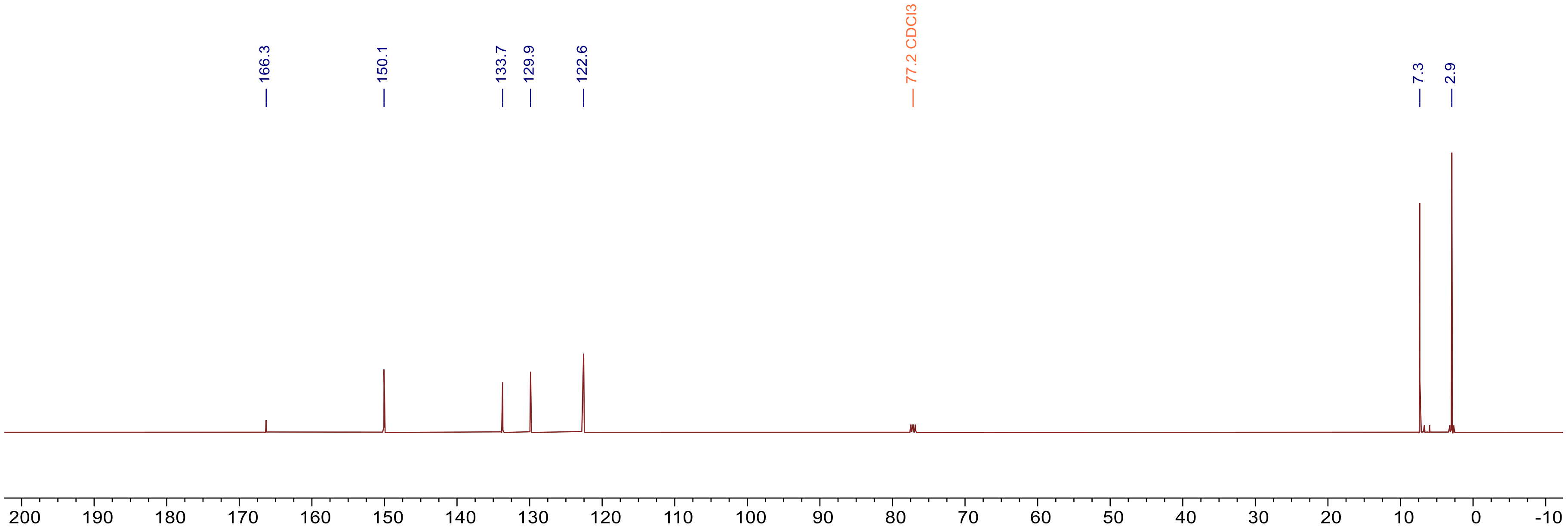

^{1}H NMR (400 MHz, $CDCl_3$):

HetSi_18

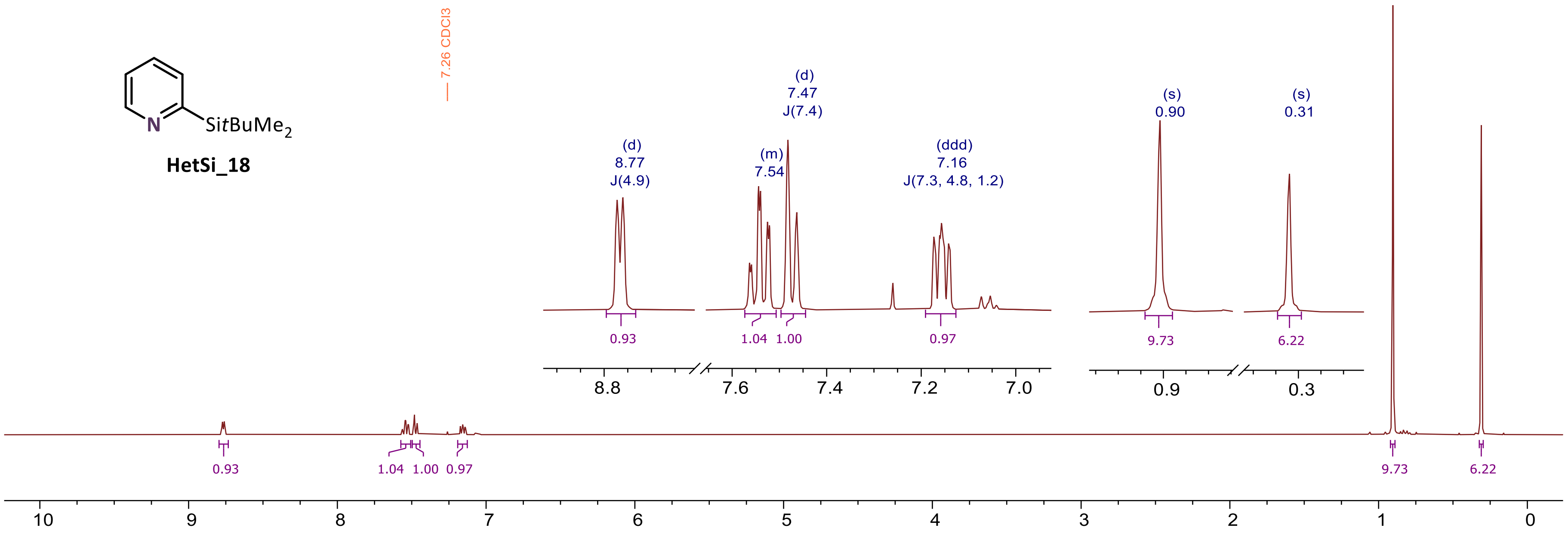

^{13}C NMR (100 MHz, $CDCl_3$):

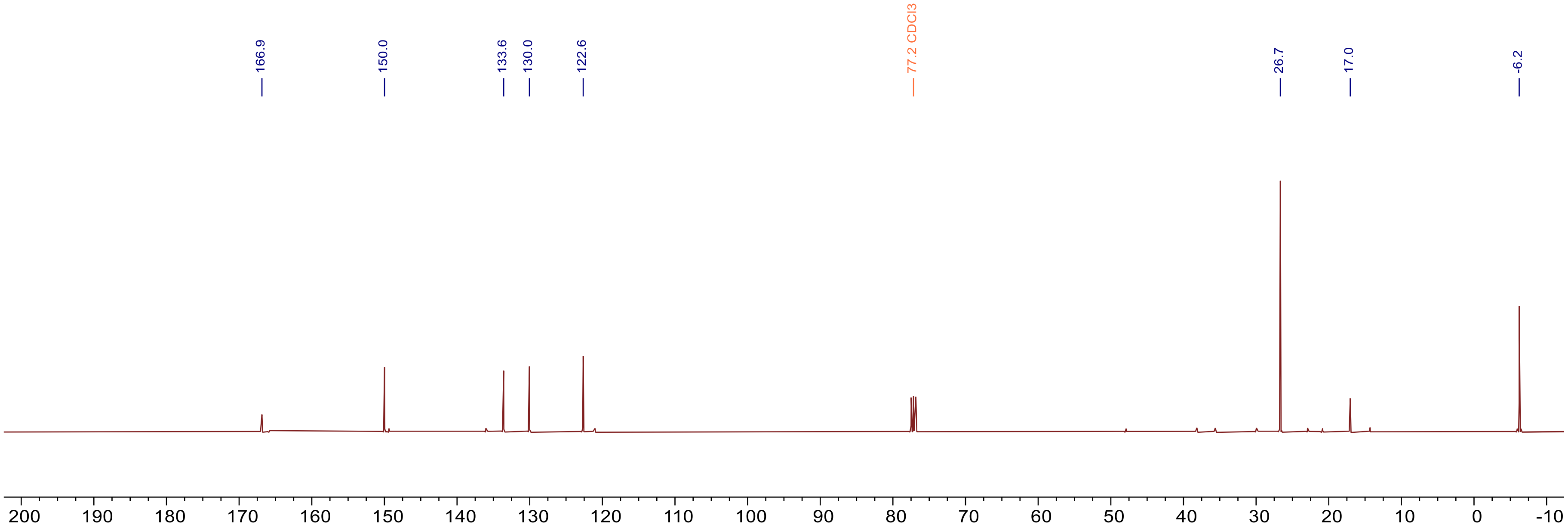

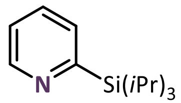

HetSi_19

^{1}H NMR (400 MHz, $CDCl_3$):

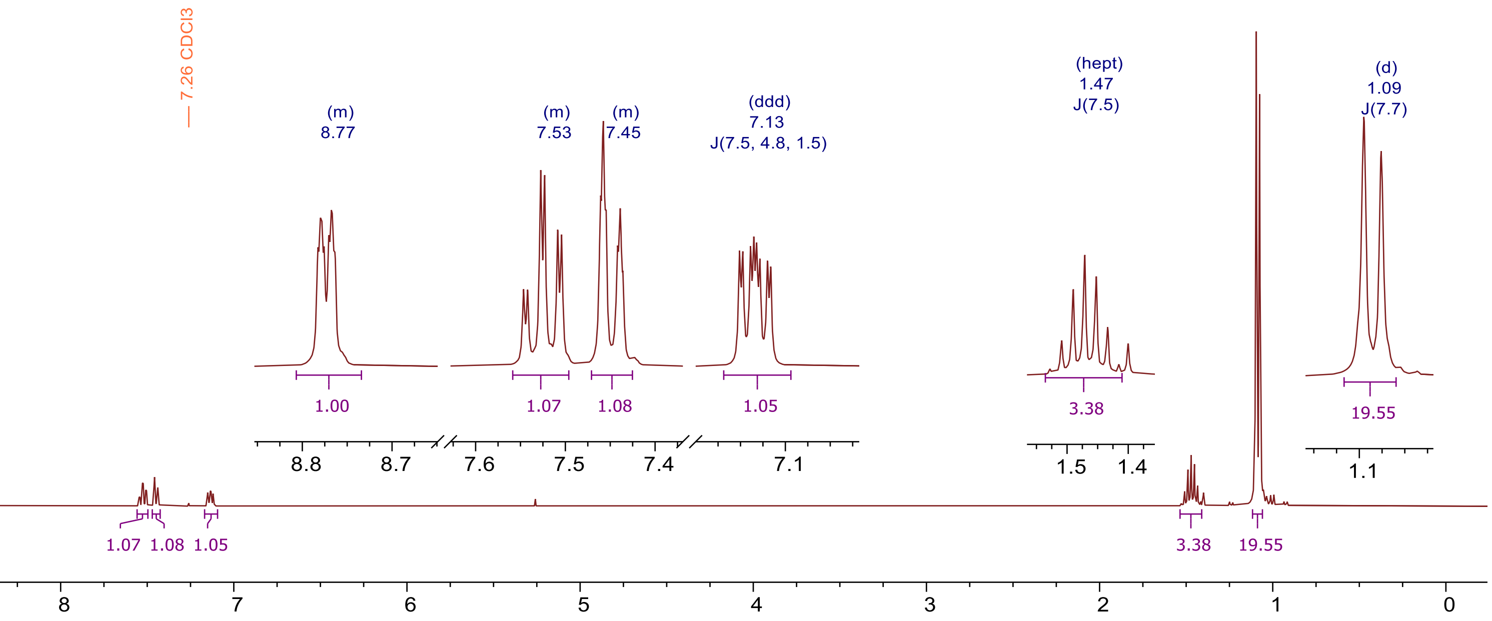

^{13}C NMR (100 MHz, $CDCl_3$):

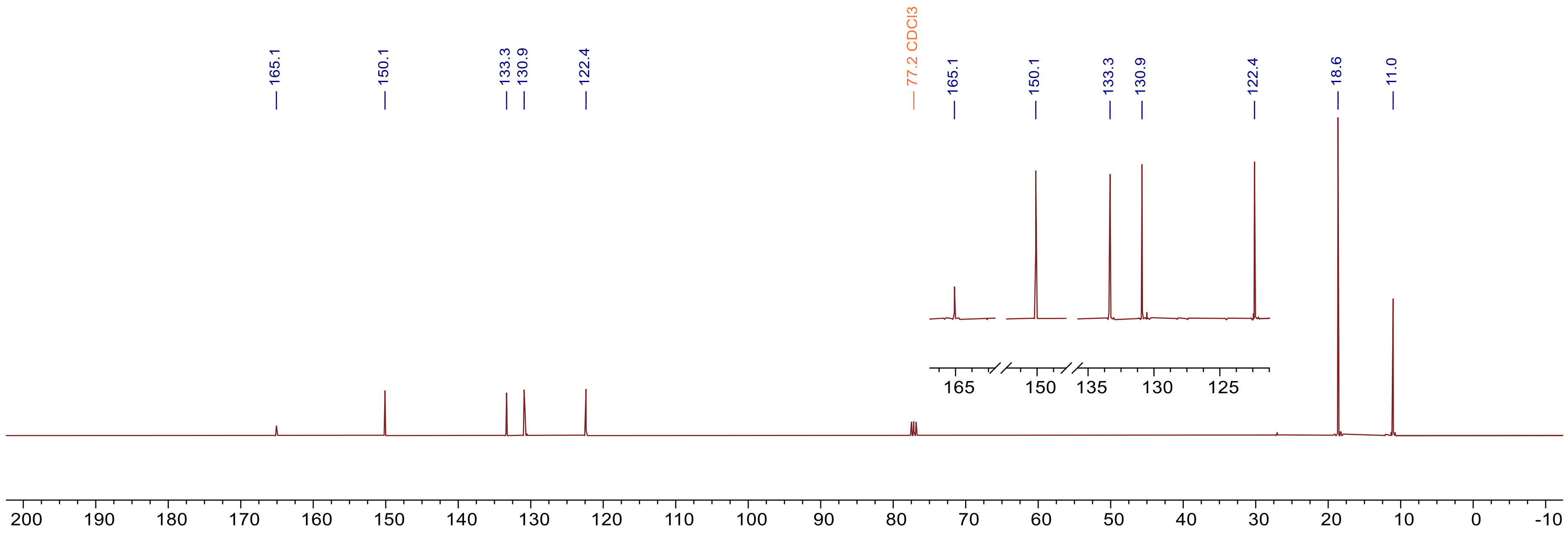

^{1}H NMR (400 MHz, $CDCl_3$):

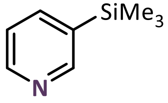

HetSi_20

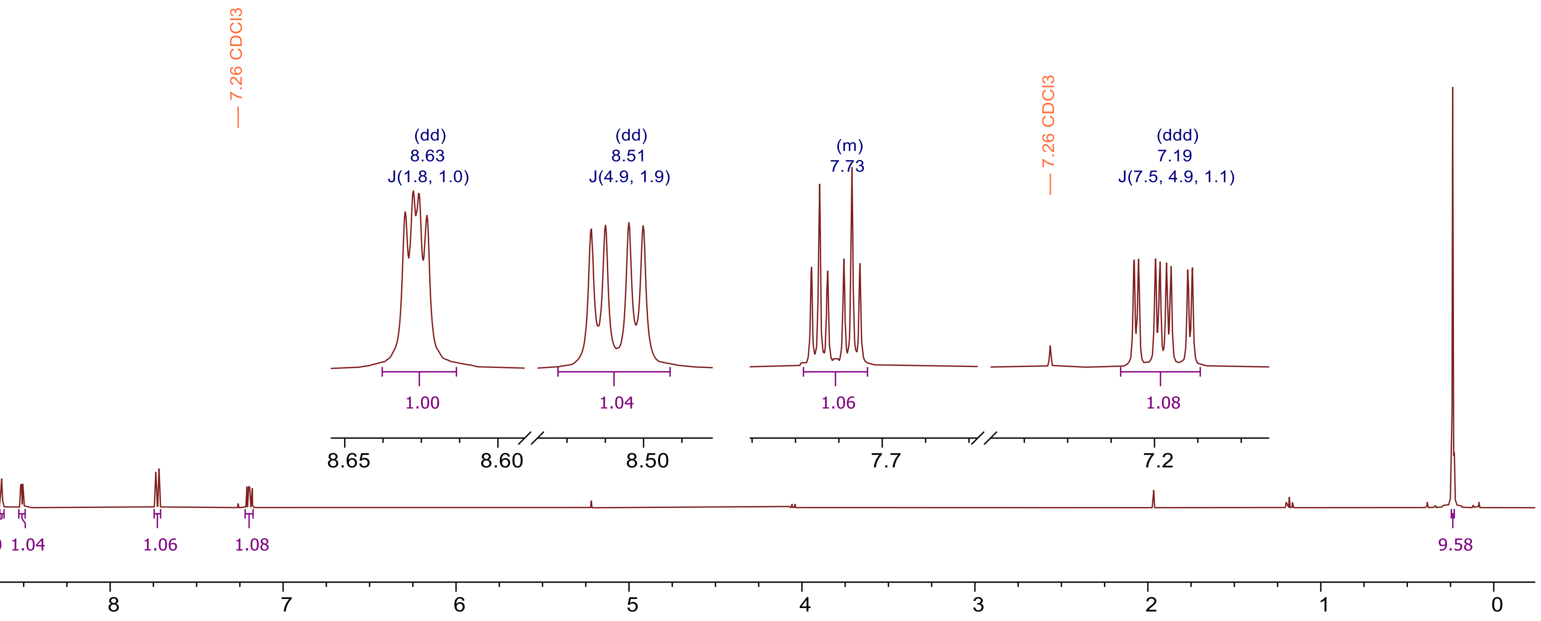

^{13}C NMR (100 MHz, $CDCl_3$):

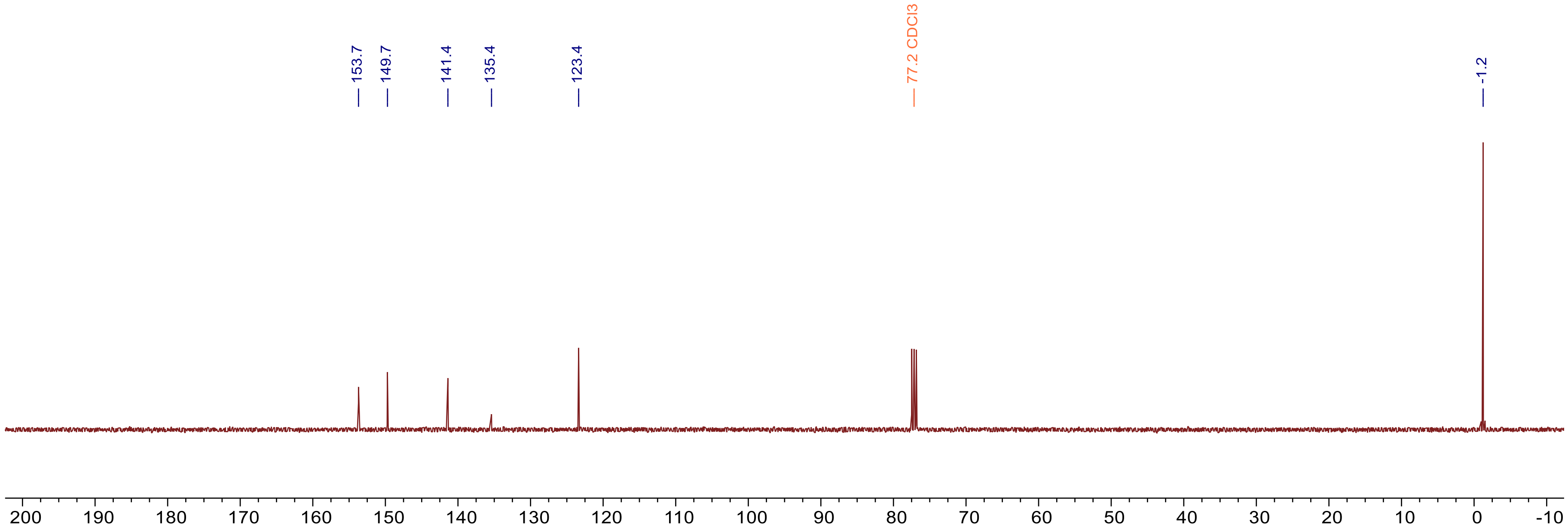

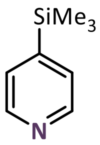

HetSi_21

^{1}H NMR (400 MHz, $CDCl_3$):

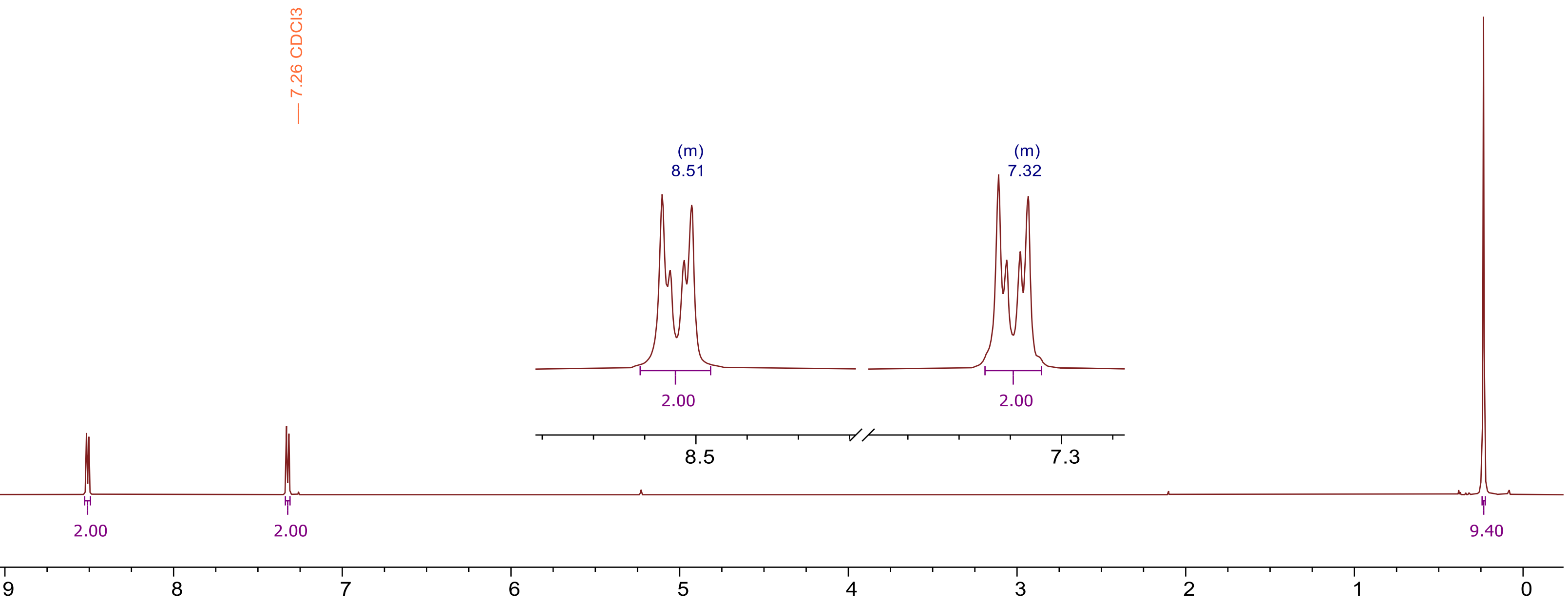

^{13}C NMR (100 MHz, $CDCl_3$):

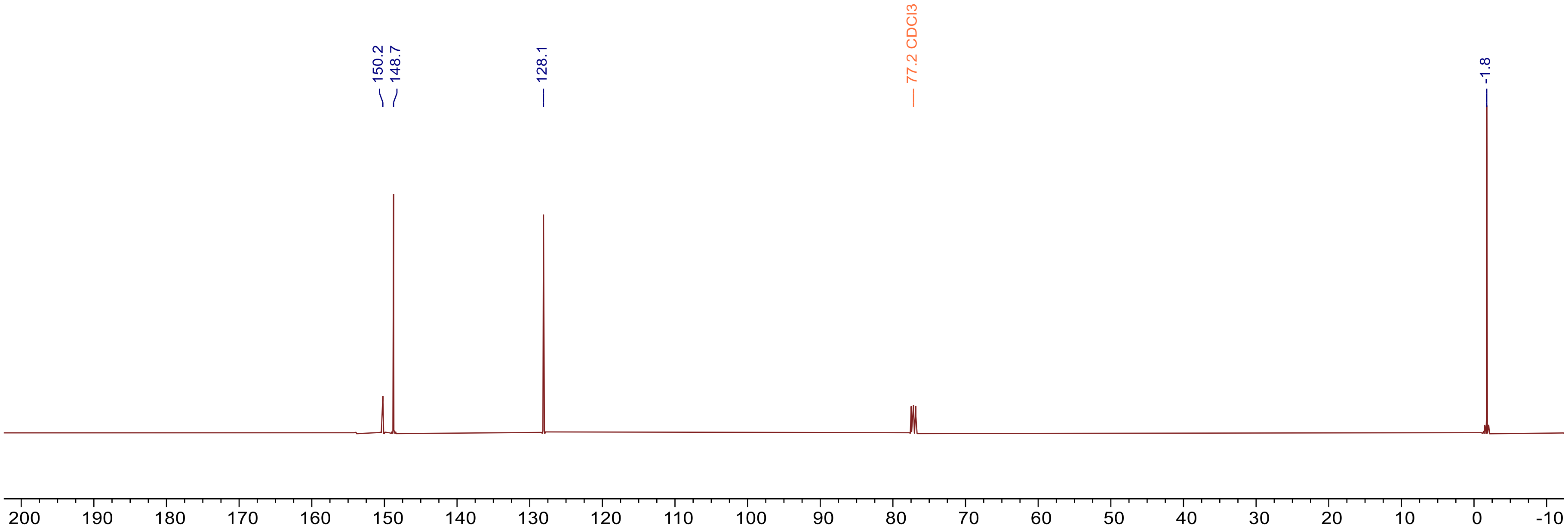

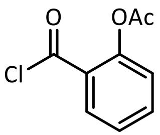

COCl_01

^{1}H NMR (400 MHz, $CDCl_3$):

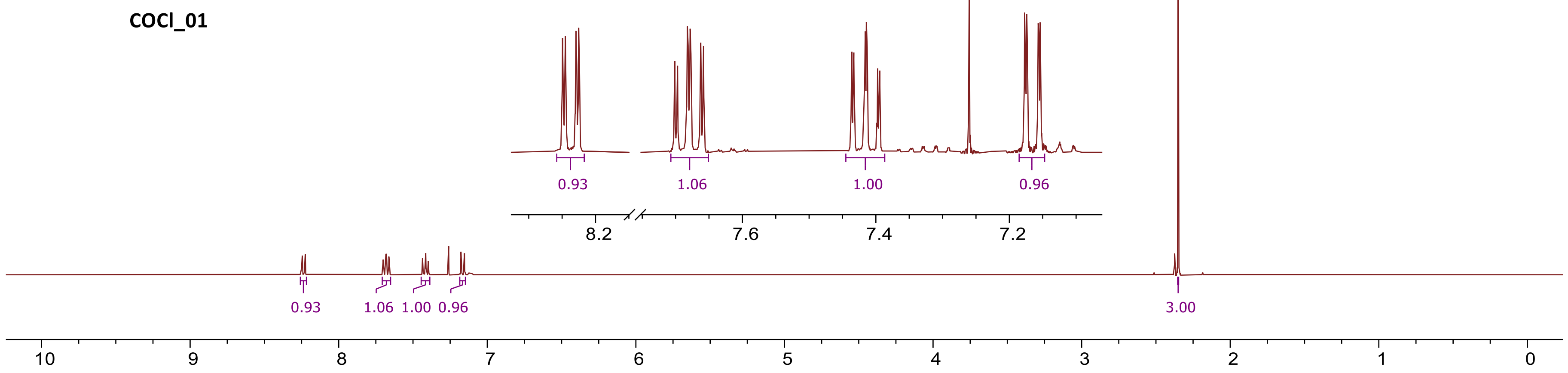

^{13}C NMR (100 MHz, $CDCl_3$):

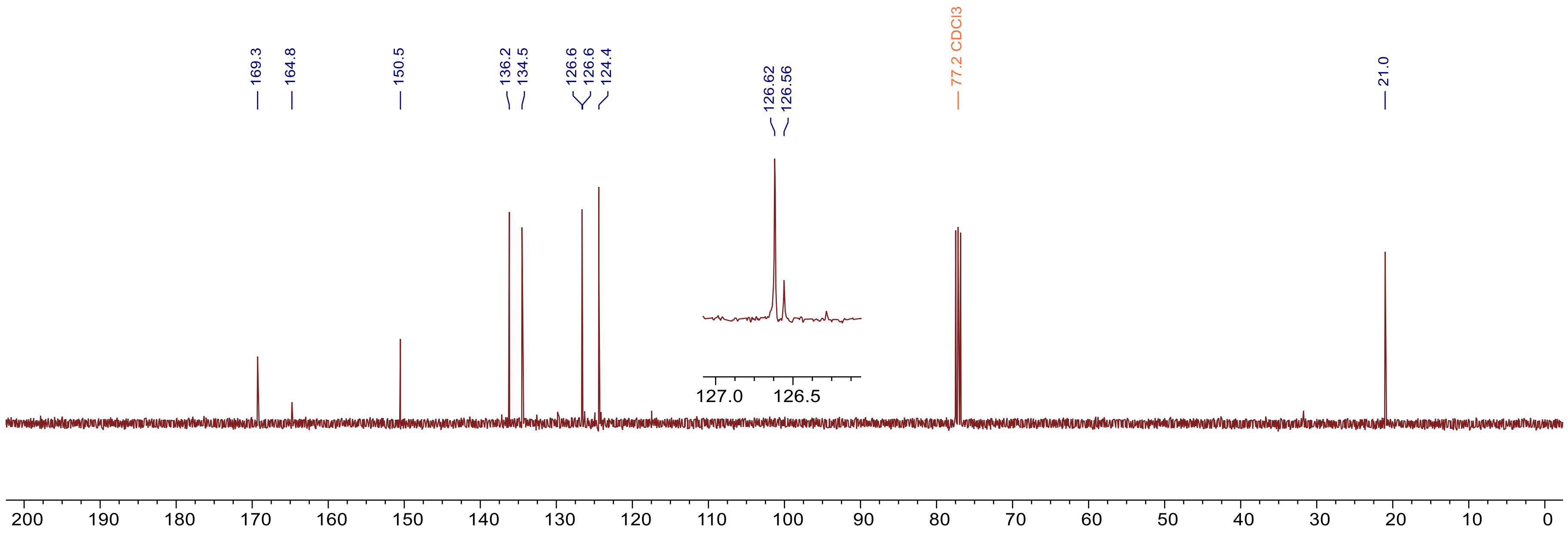

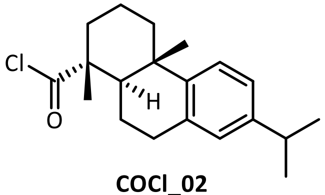

COCl_02

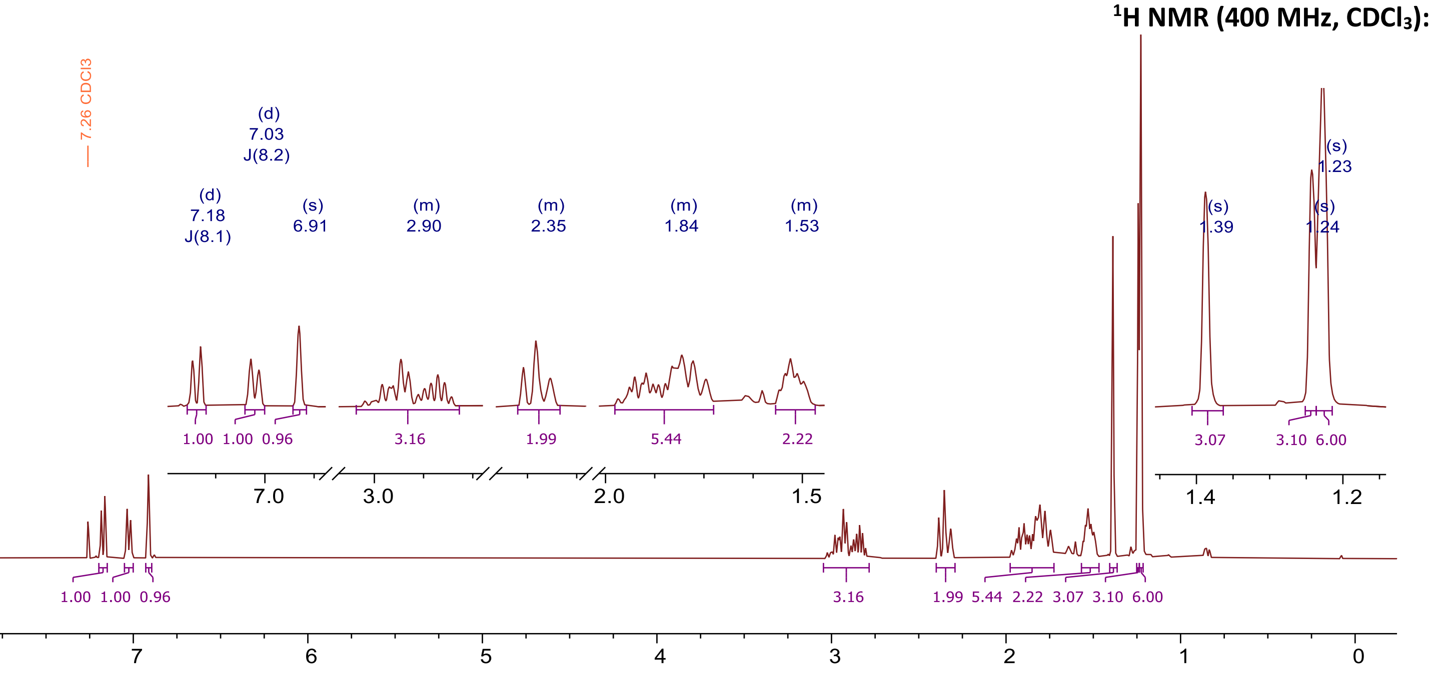

^{1}H NMR (400 MHz, CDCl$_3$):
7.26 CDCl3
(d) 7.18 J(8.1)
(d) 7.03 J(8.2)
(s) 6.91
(m) 2.90
(m) 2.35
(m) 1.84
(m) 1.53
(s) 1.39
(s) 1.24
(s) 1.23
1.00 1.00 0.96 3.16 1.99 5.44 2.22 3.07 3.10 6.00

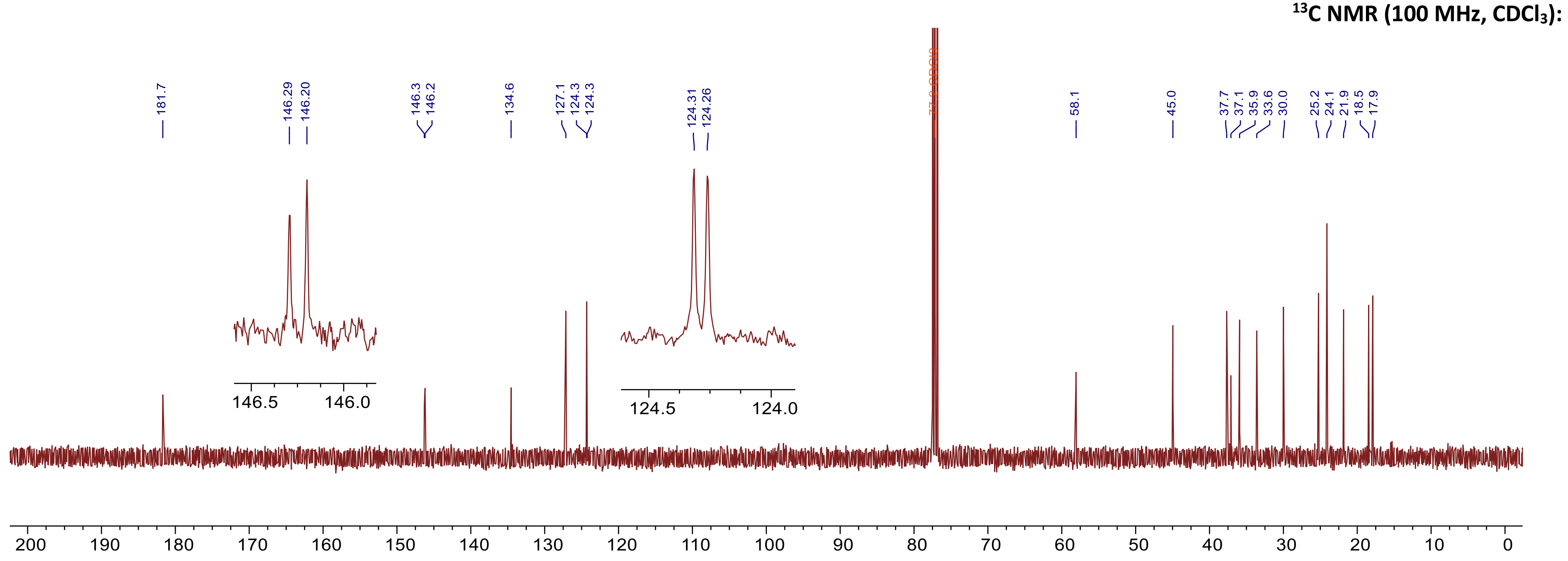

^{13}C NMR (100 MHz, CDCl$_3$):
181.7
146.29
146.20
146.3
146.2
134.6
127.1
124.3
124.3
124.31
124.26
58.1
45.0
37.7
37.1
35.9
33.6
30.0
25.2
24.1
21.9
18.5
17.9

^{1}H NMR (400 MHz, $CDCl_3$):

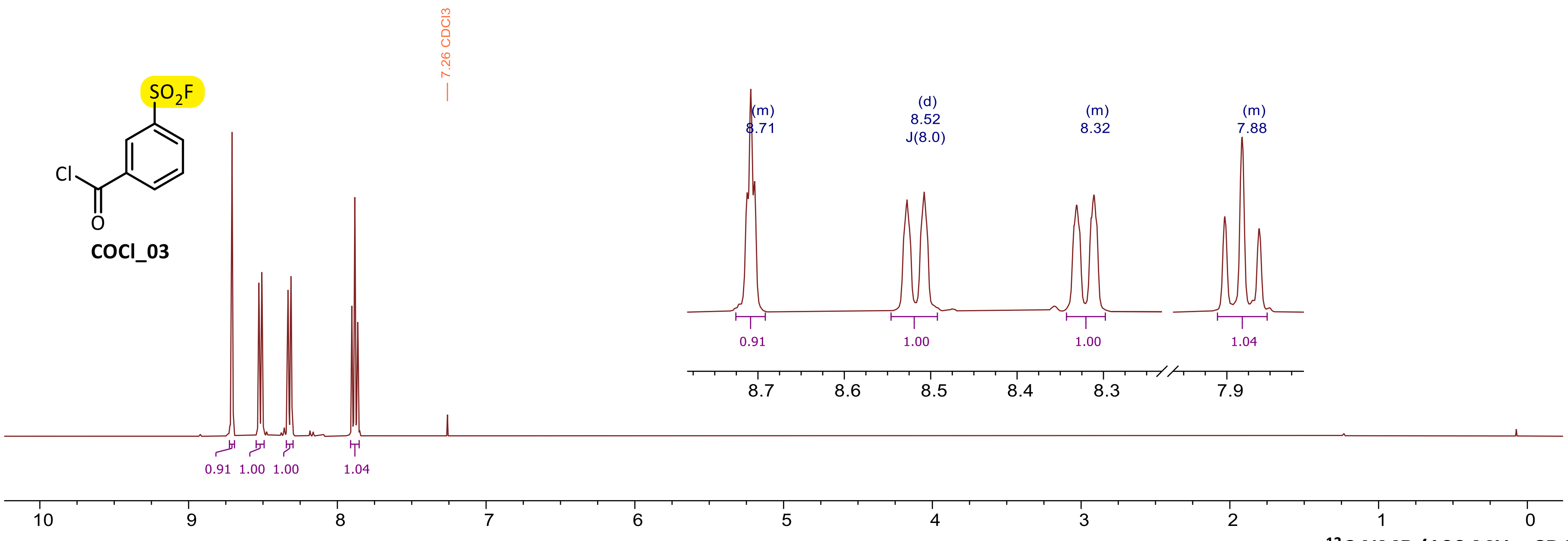

^{13}C NMR (100 MHz, $CDCl_3$):

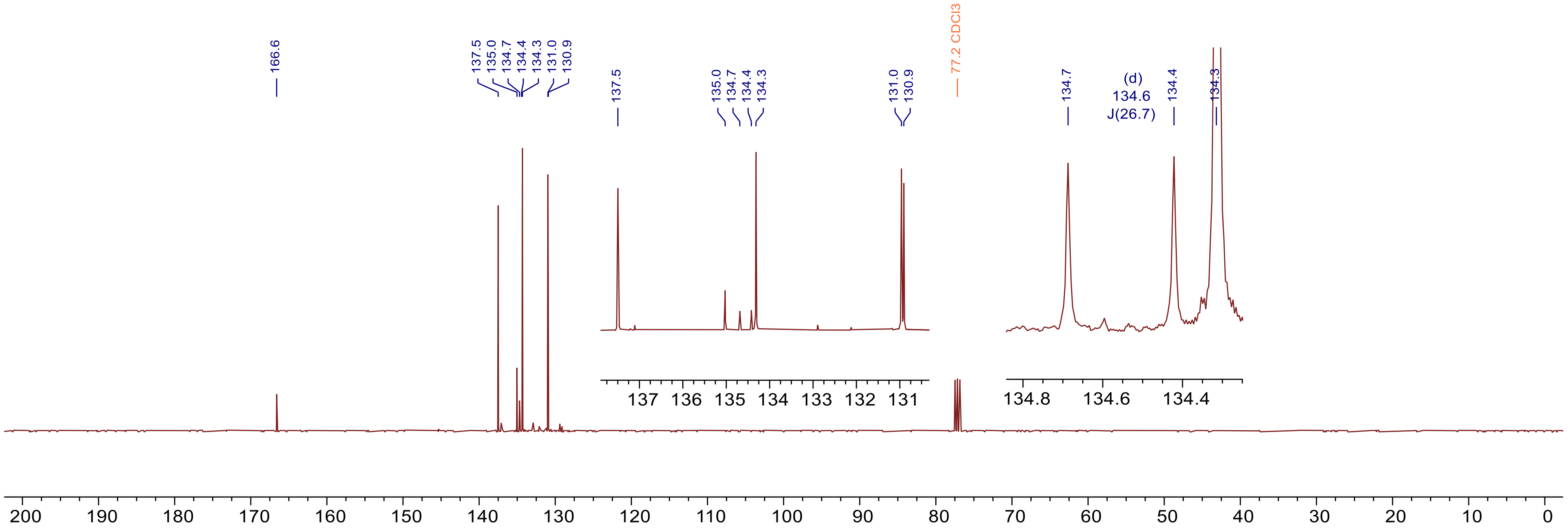

^{19}F NMR (376 MHz, $CDCl_3$):

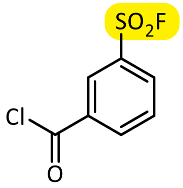

COCl_03

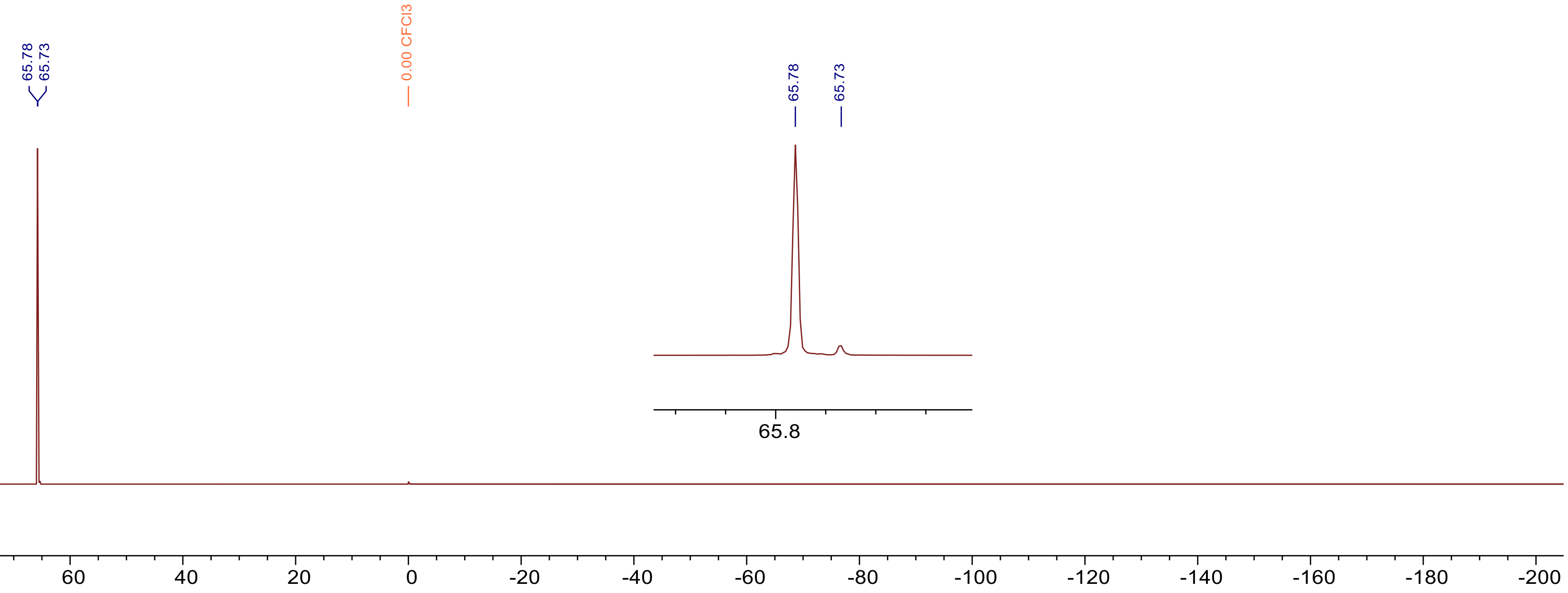

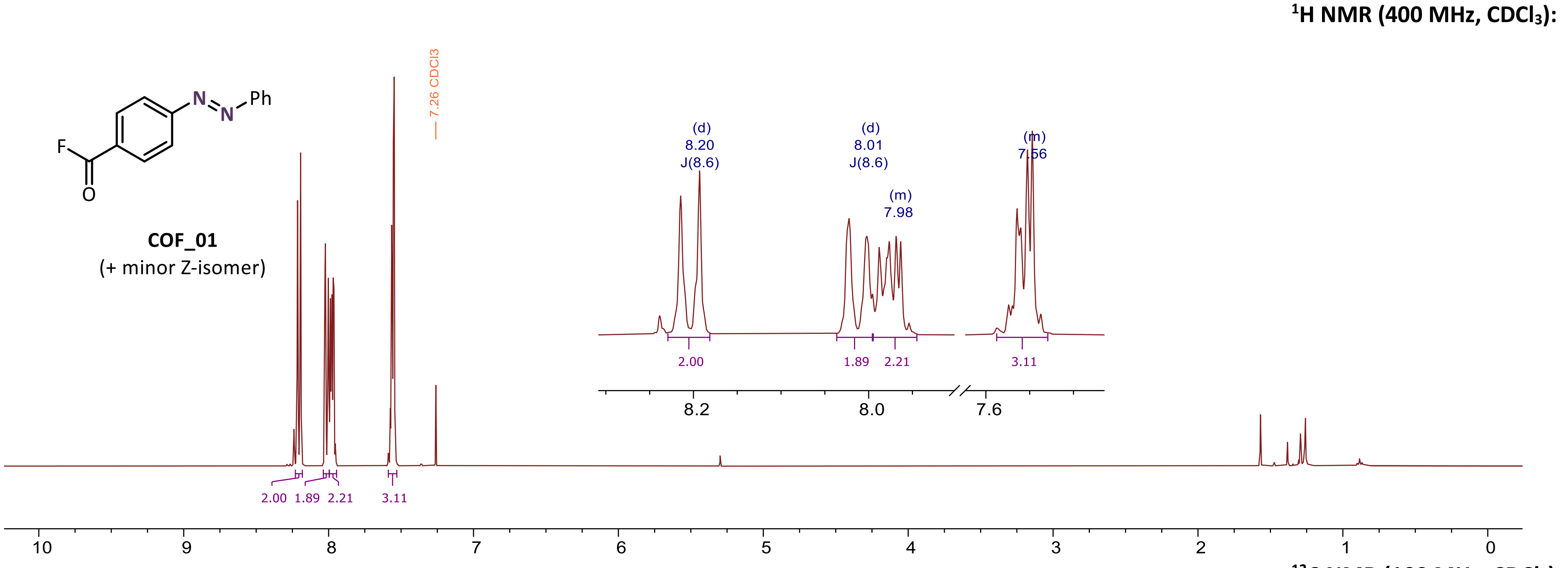

¹H NMR (400 MHz, CDCl3):
COF_01
(+ minor Z-isomer)
7.26 CDCl3
(d) 8.20 J(8.6)
(d) 8.01 J(8.6)
(m) 7.98
(m) 7.56
2.00 1.89 2.21 3.11

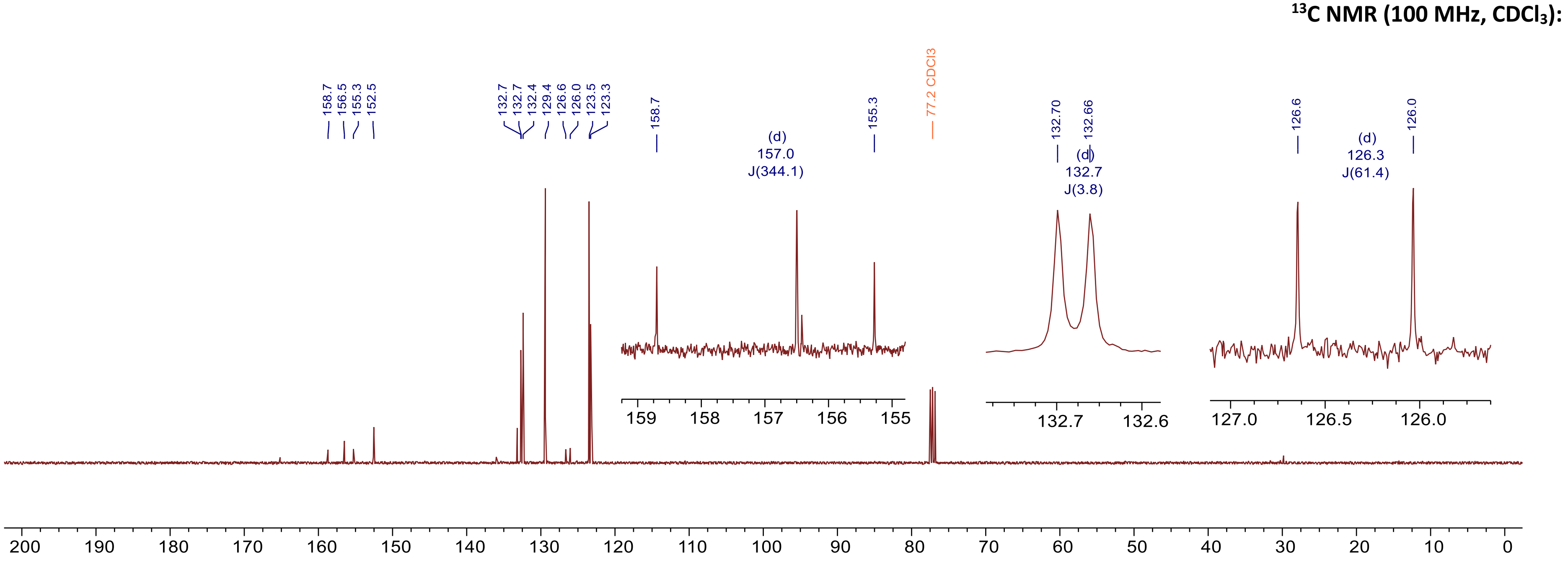

¹³C NMR (100 MHz, CDCl3):
158.7 156.5 155.3 152.5
132.7 132.7 132.4 129.4 126.6 126.0 123.5 123.3
77.2 CDCl3
(d) 157.0 J(344.1)
(d) 132.7 J(3.8)
(d) 126.3 J(61.4)

^{19}F NMR (376 MHz, $CDCl_3$):

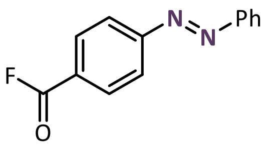

COF_01

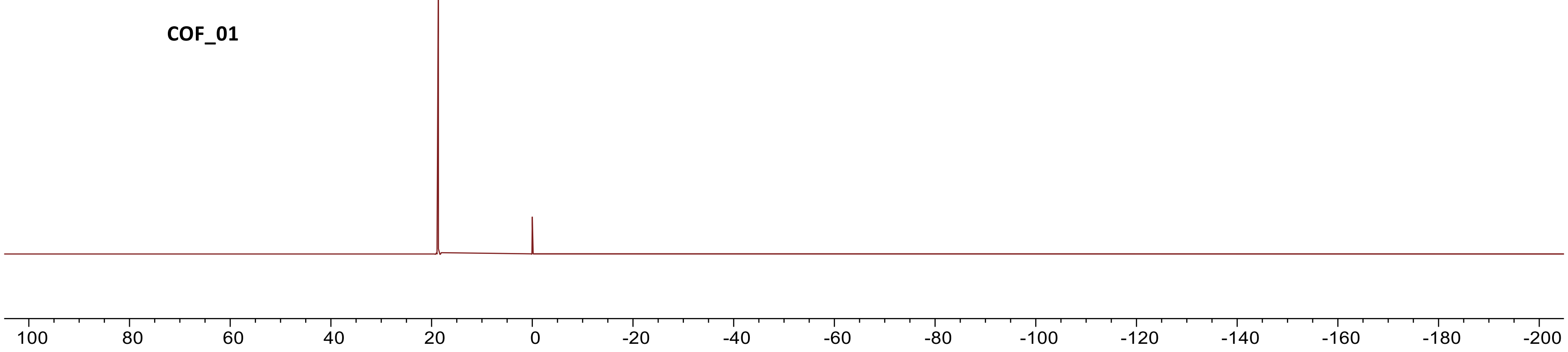

^{1}H NMR (400 MHz, $CDCl_3$):

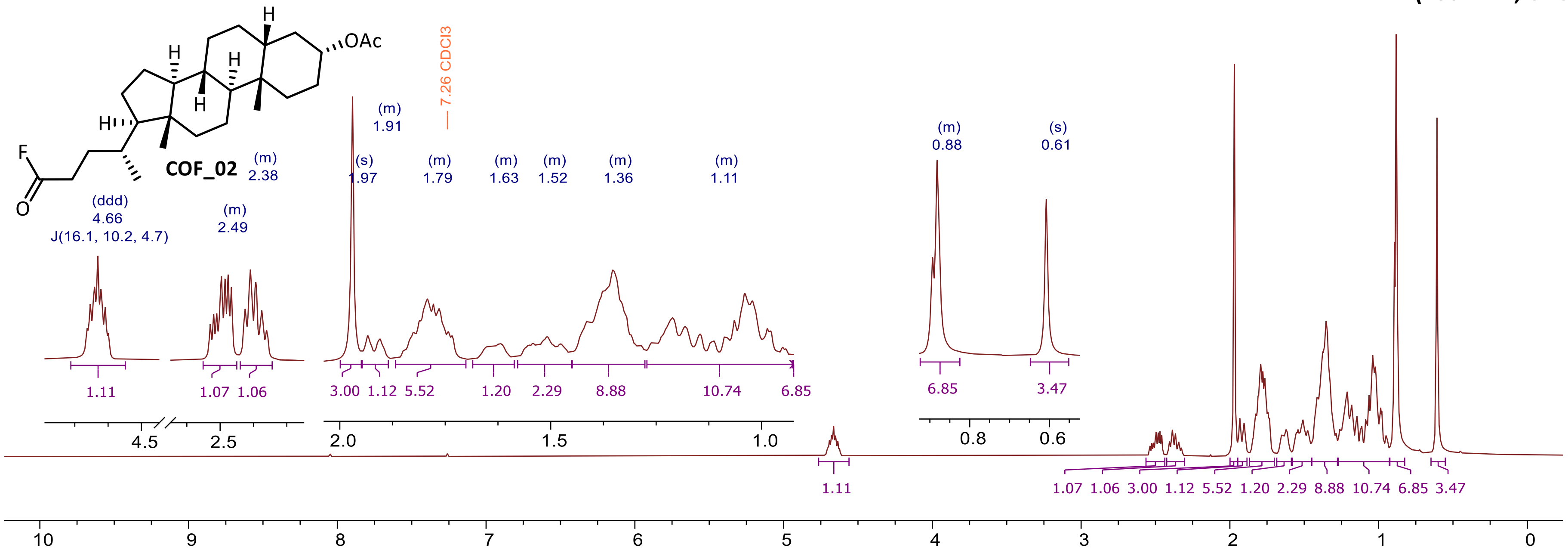

^{13}C NMR (100 MHz, $CDCl_3$):

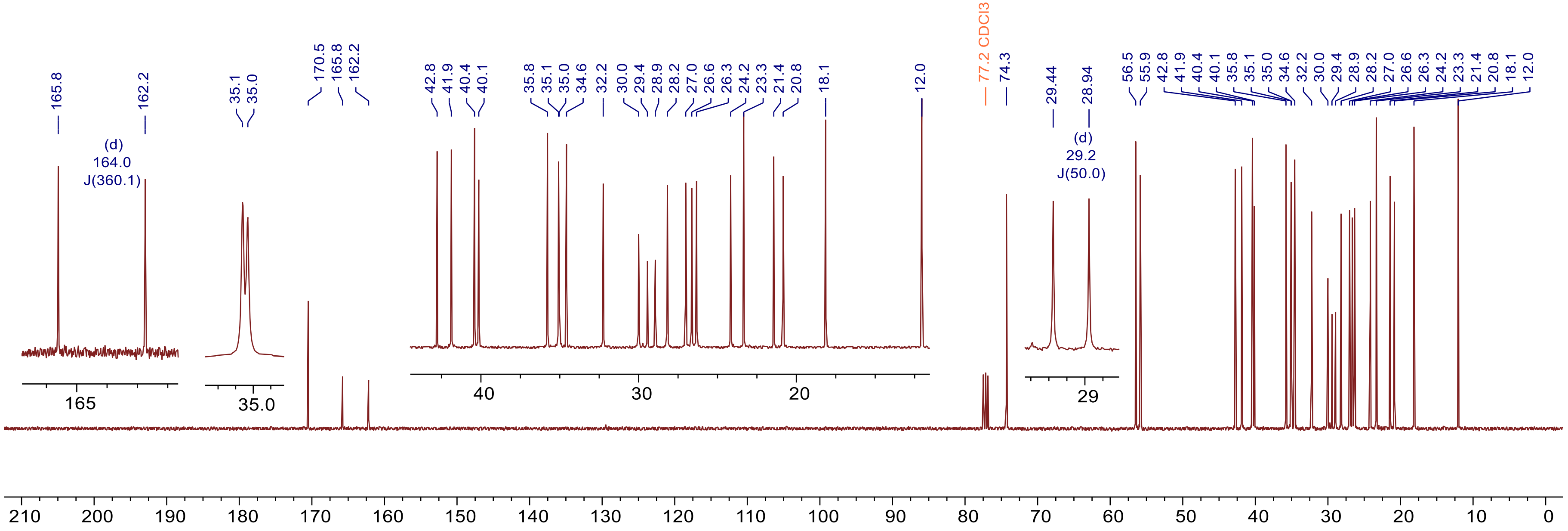

^{19}F NMR (376 MHz, $CDCl_3$):

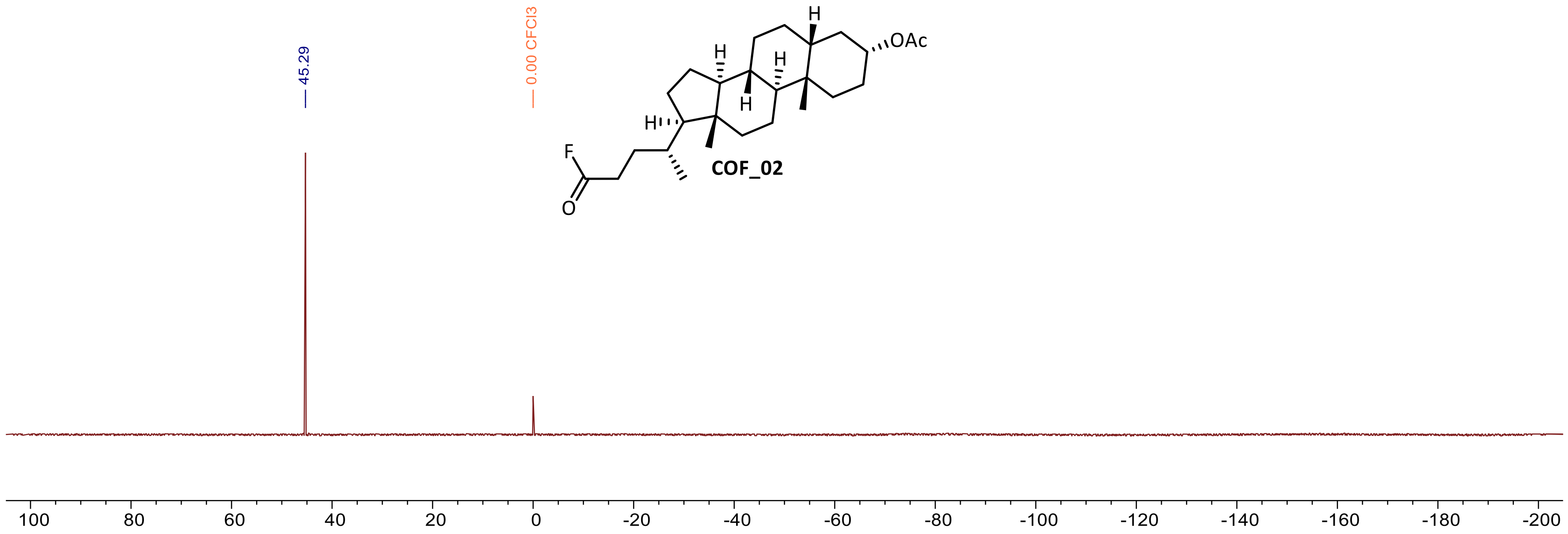

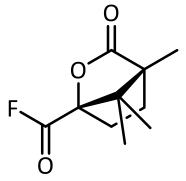

COF_03

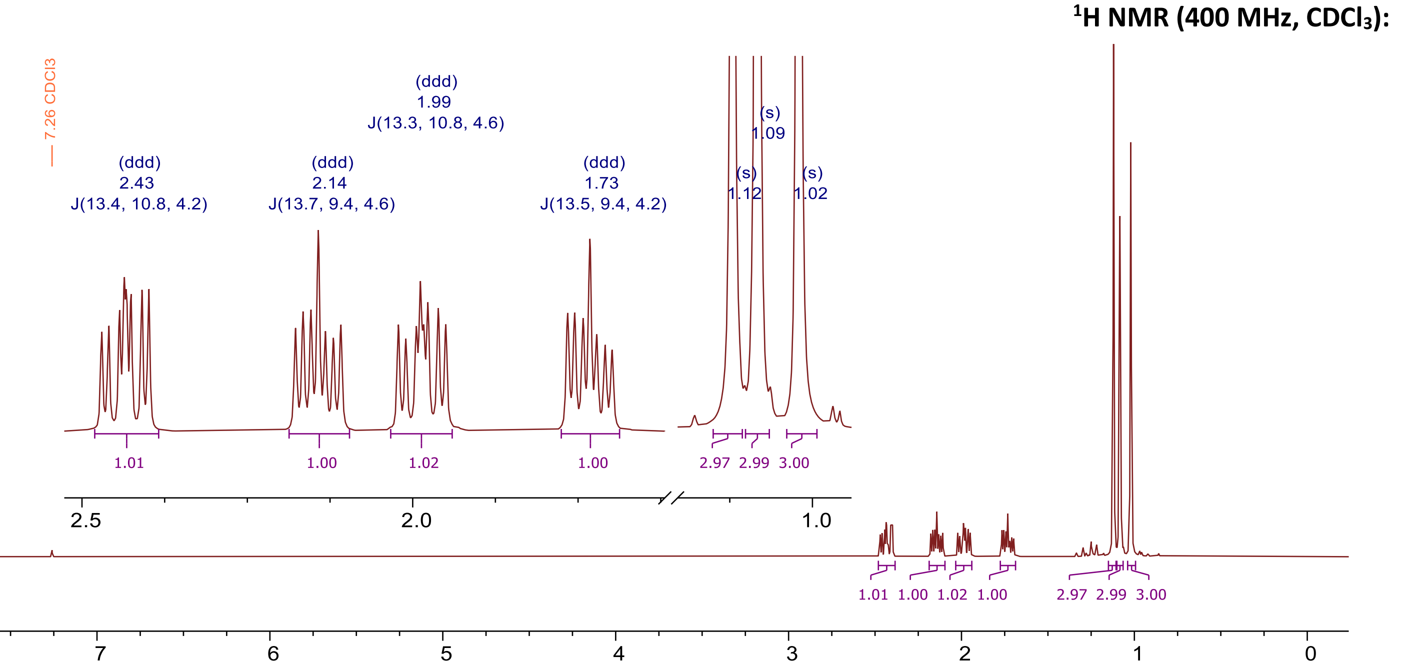

^{1}H NMR (400 MHz, CDCl$_3$):
7.26 CDCl3
(ddd) 2.43 J(13.4, 10.8, 4.2)
(ddd) 2.14 J(13.7, 9.4, 4.6)
(ddd) 1.99 J(13.3, 10.8, 4.6)
(ddd) 1.73 J(13.5, 9.4, 4.2)
(s) 1.12
(s) 1.09
(s) 1.02
1.01 1.00 1.02 1.00 2.97 2.99 3.00

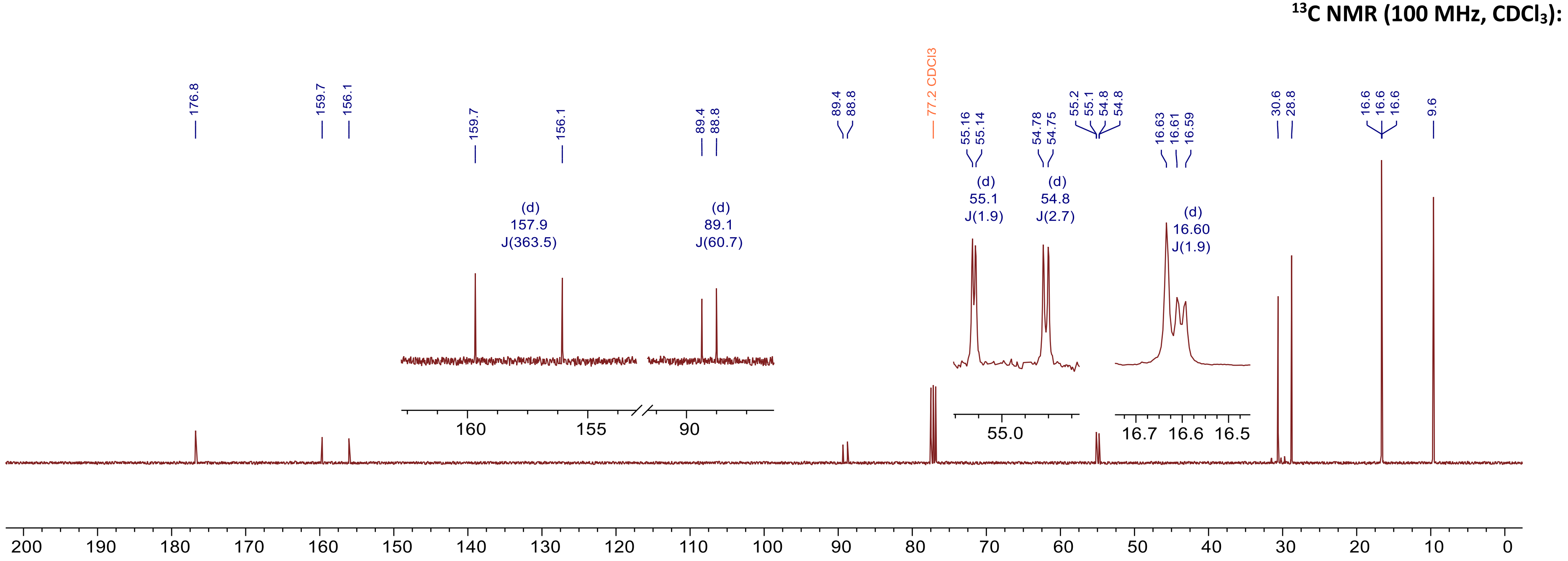

^{13}C NMR (100 MHz, CDCl$_3$):
176.8
159.7
156.1
89.4
88.8
77.2 CDCl3
55.16
55.14
54.78
54.75
55.2
55.1
54.8
54.8
16.63
16.61
16.59
30.6
28.8
16.6
16.6
16.6
9.6
(d) 157.9 J(363.5)
(d) 89.1 J(60.7)
(d) 55.1 J(1.9)
(d) 54.8 J(2.7)
(d) 16.60 J(1.9)

^{19}F NMR (376 MHz, CDCl$_3$):

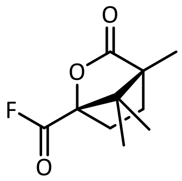

COF_03

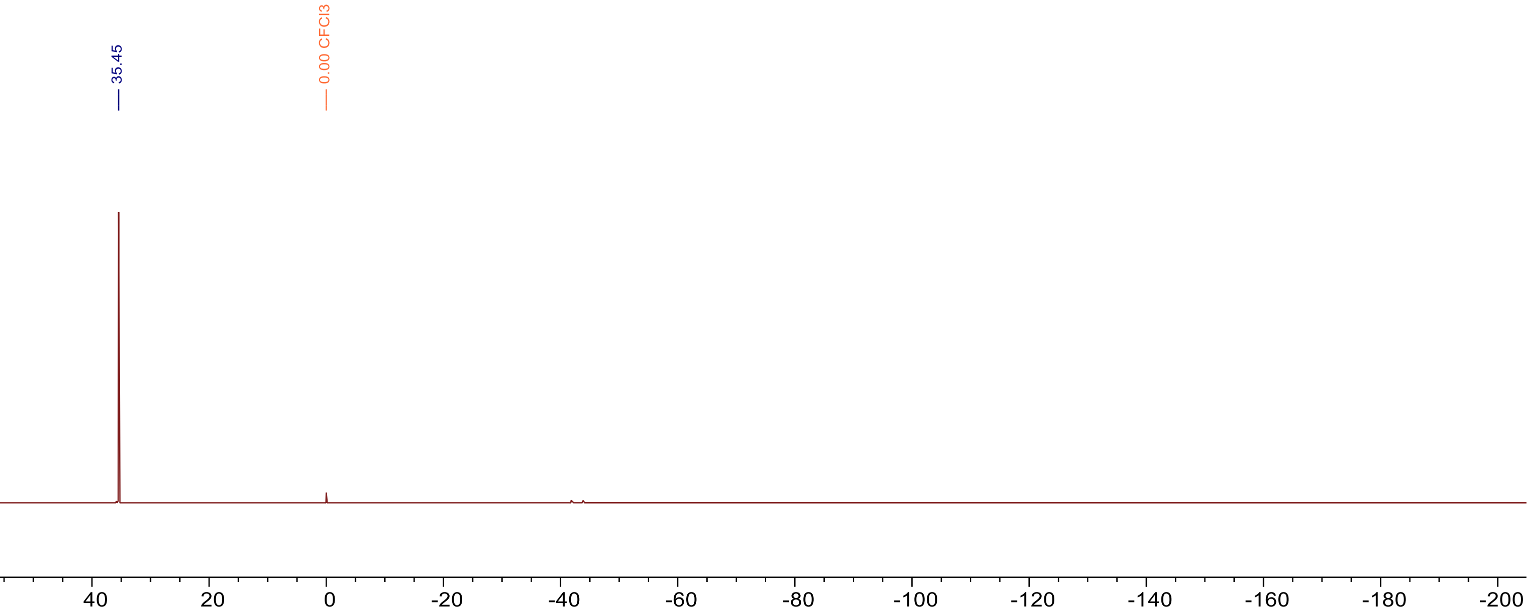

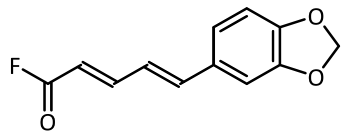

COF_04

^{1}H NMR (400 MHz, $CDCl_3$):

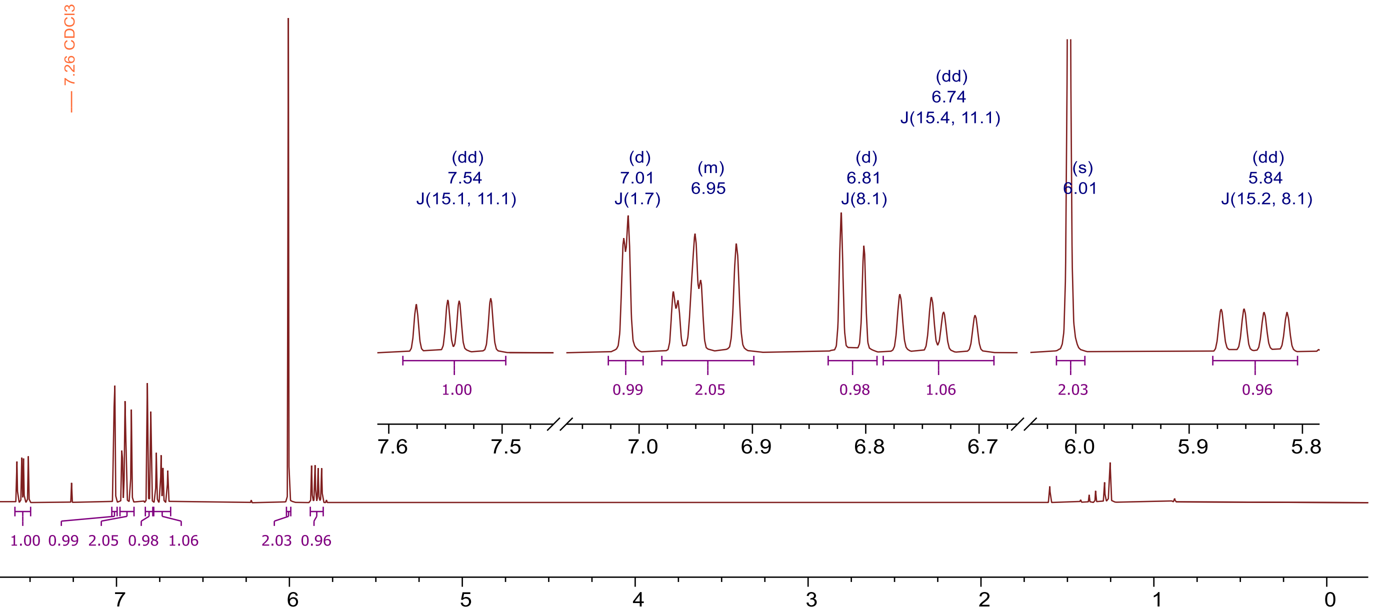

^{13}C NMR (100 MHz, $CDCl_3$):

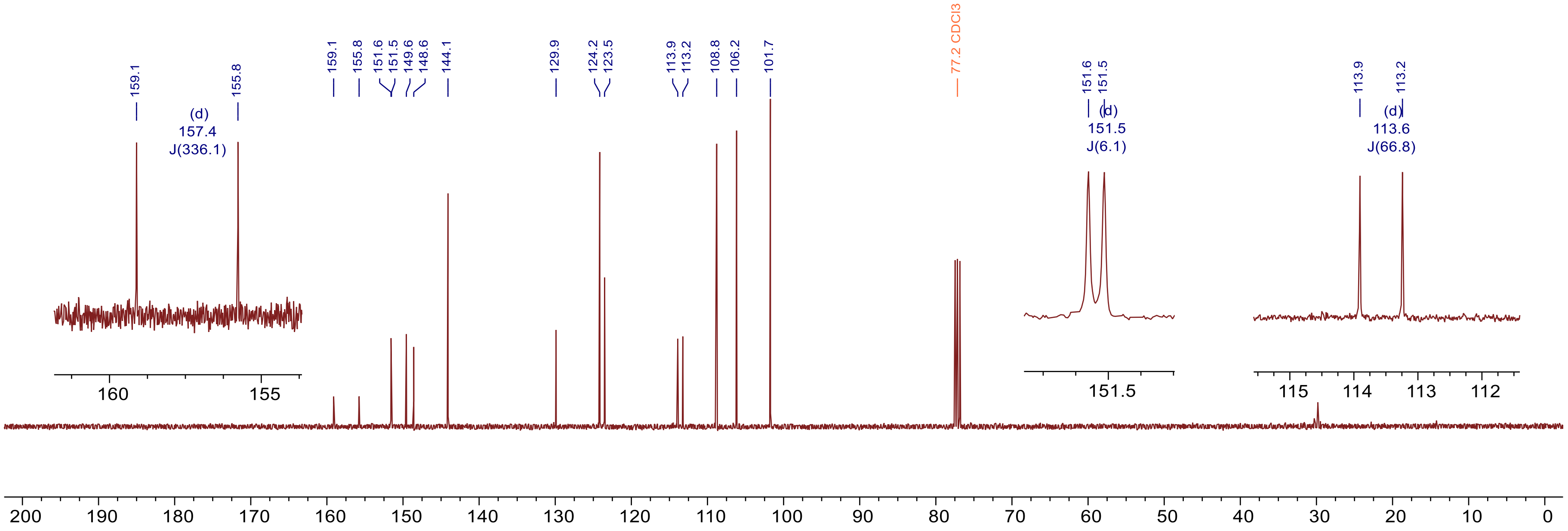

^{19}F NMR (376 MHz, $CDCl_3$):

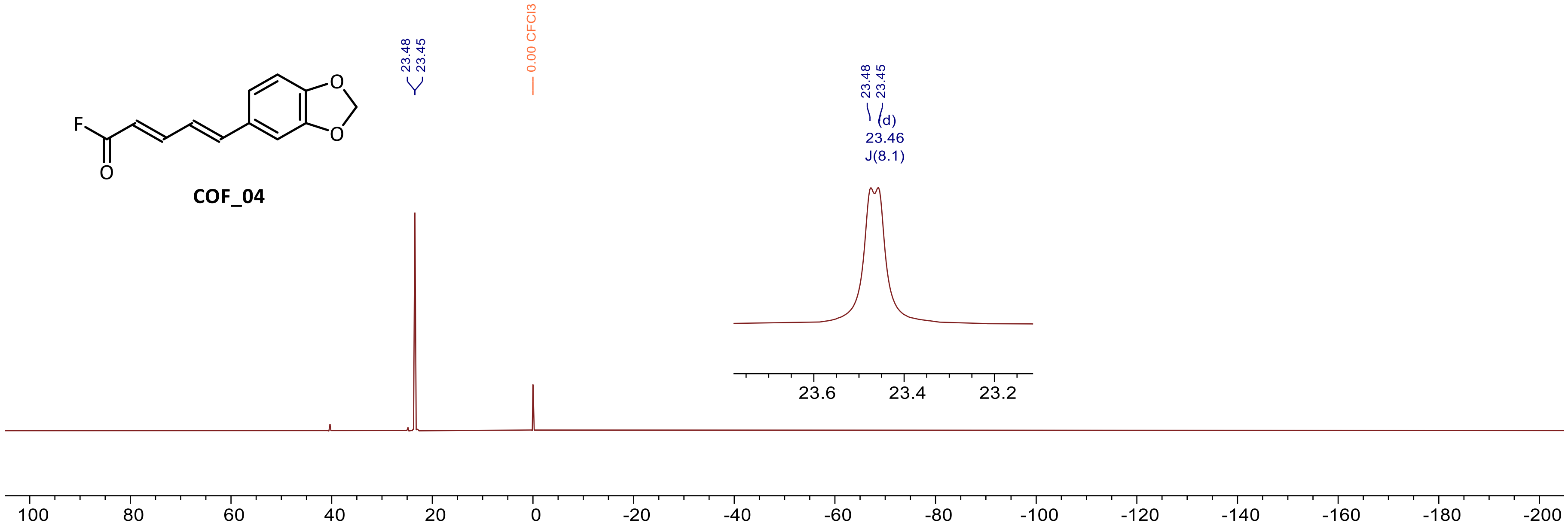

^{1}H NMR (400 MHz, $CDCl_3$):

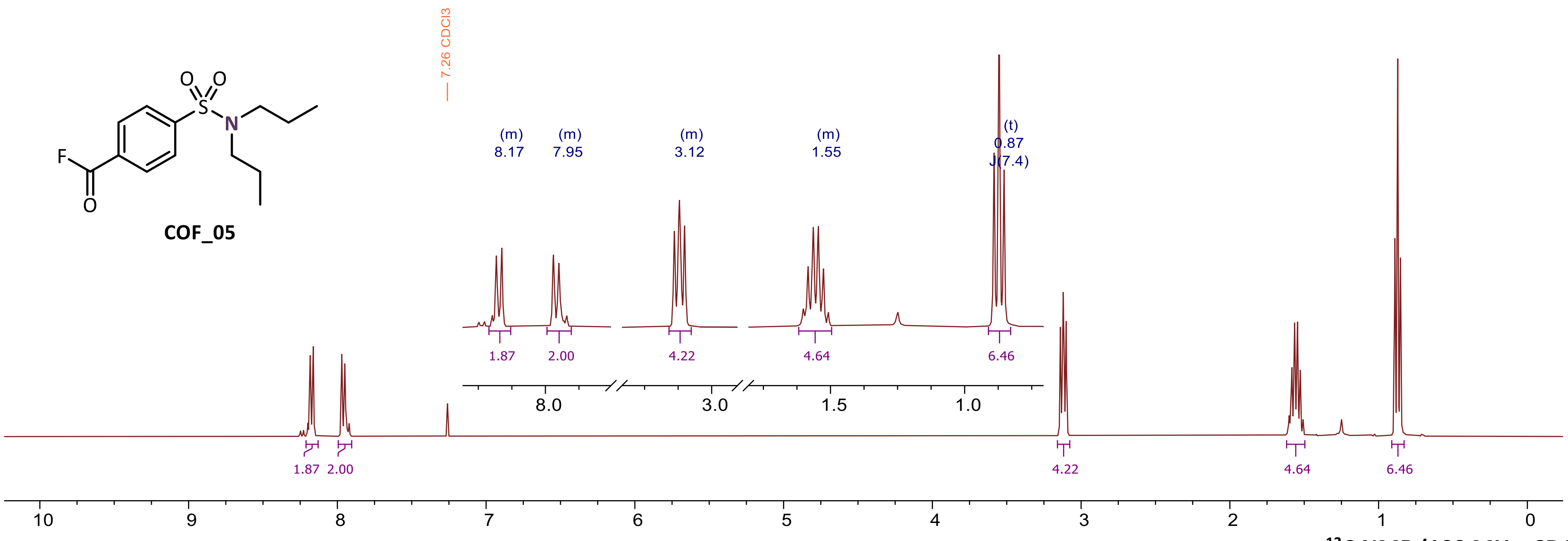

^{13}C NMR (100 MHz, $CDCl_3$):

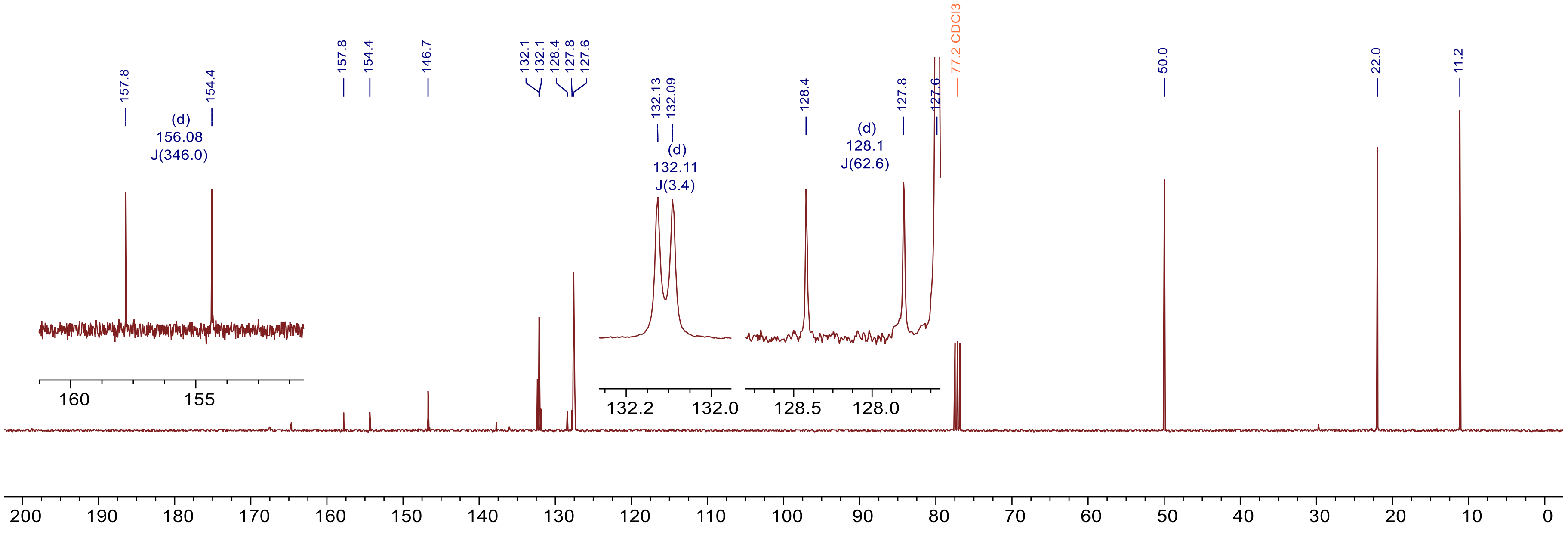

^{19}F NMR (376 MHz, $CDCl_3$):

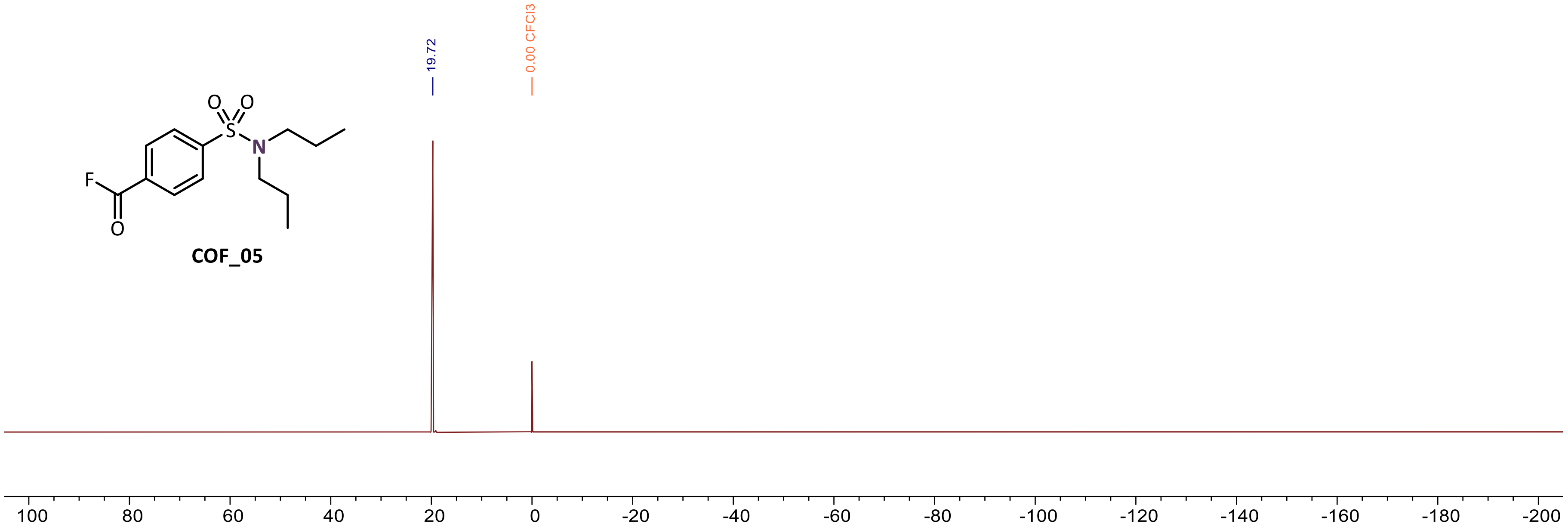

COF_05

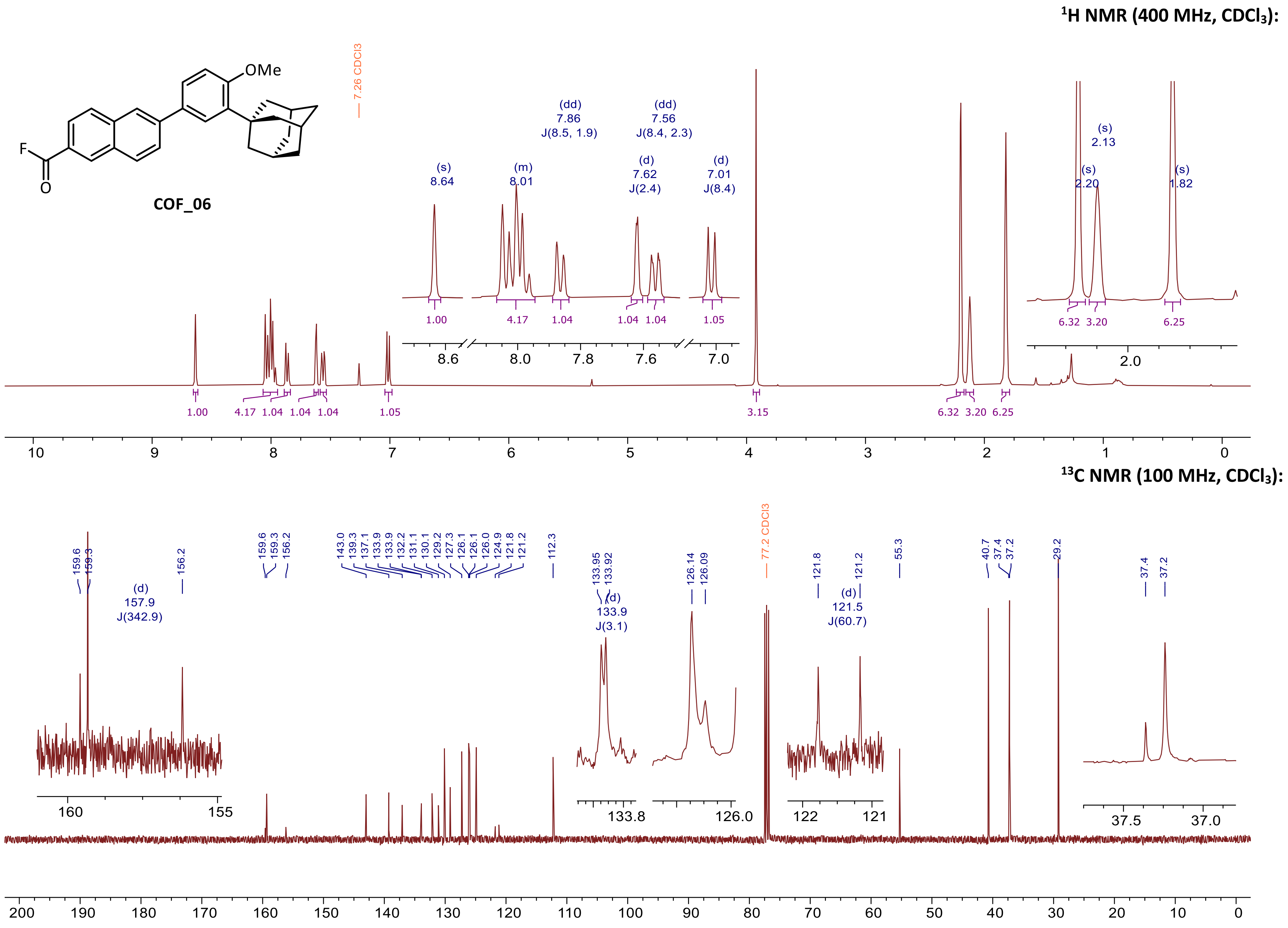

¹H NMR (400 MHz, CDCl₃):
COF_06
OMe
F
O
7.26 CDCl3
(s) 8.64
(m) 8.01
(dd) 7.86 J(8.5, 1.9)
(d) 7.62 J(2.4)
(dd) 7.56 J(8.4, 2.3)
(d) 7.01 J(8.4)
(s) 2.20
(s) 2.13
(s) 1.82
1.00
4.17
1.04
1.04
1.04
1.05
3.15
6.32
3.20
6.25
¹³C NMR (100 MHz, CDCl₃):
159.6
159.3
(d) 157.9 J(342.9)
156.2
143.0
139.3
137.1
133.9
133.9
132.2
131.1
130.1
129.2
127.3
126.1
126.1
126.0
124.9
121.8
121.2
112.3
133.95
133.92
(d) 133.9 J(3.1)
126.14
126.09
77.2 CDCl3
121.8
121.2
(d) 121.5 J(60.7)
55.3
40.7
37.4
37.2
29.2
37.4
37.2

^{19}F NMR (376 MHz, $CDCl_3$):

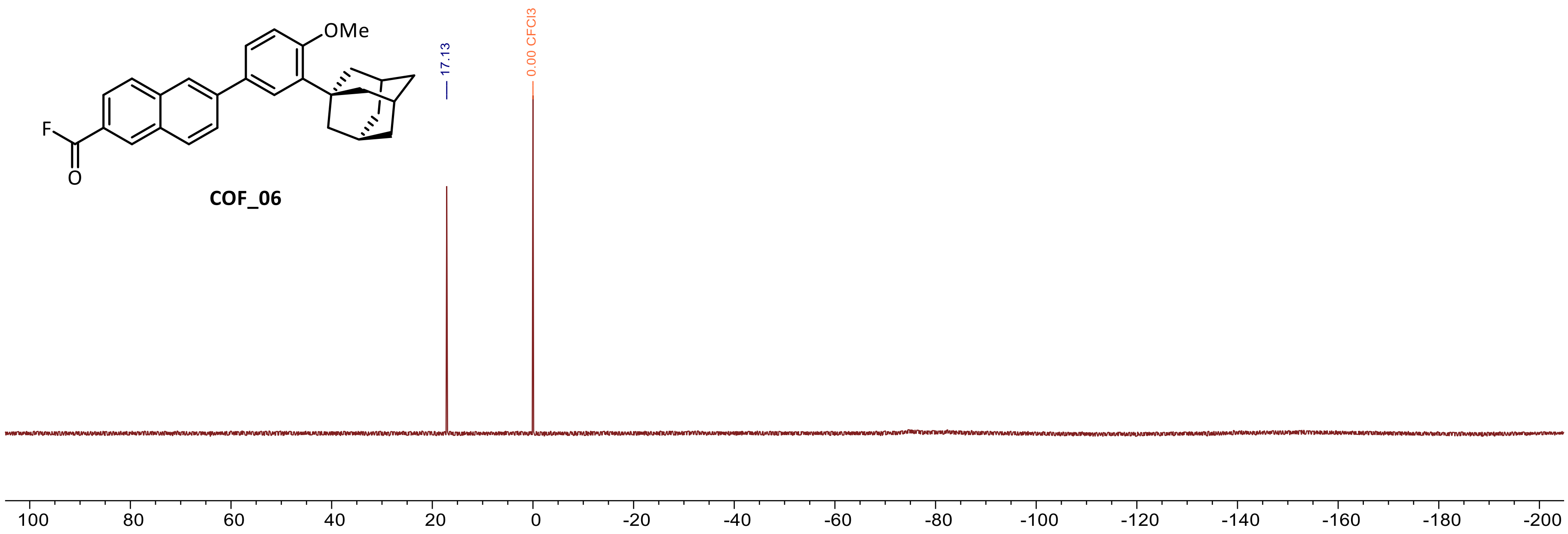

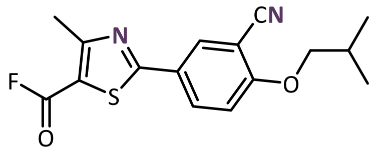

COF_07

^{1}H NMR (400 MHz, $CDCl_3$):

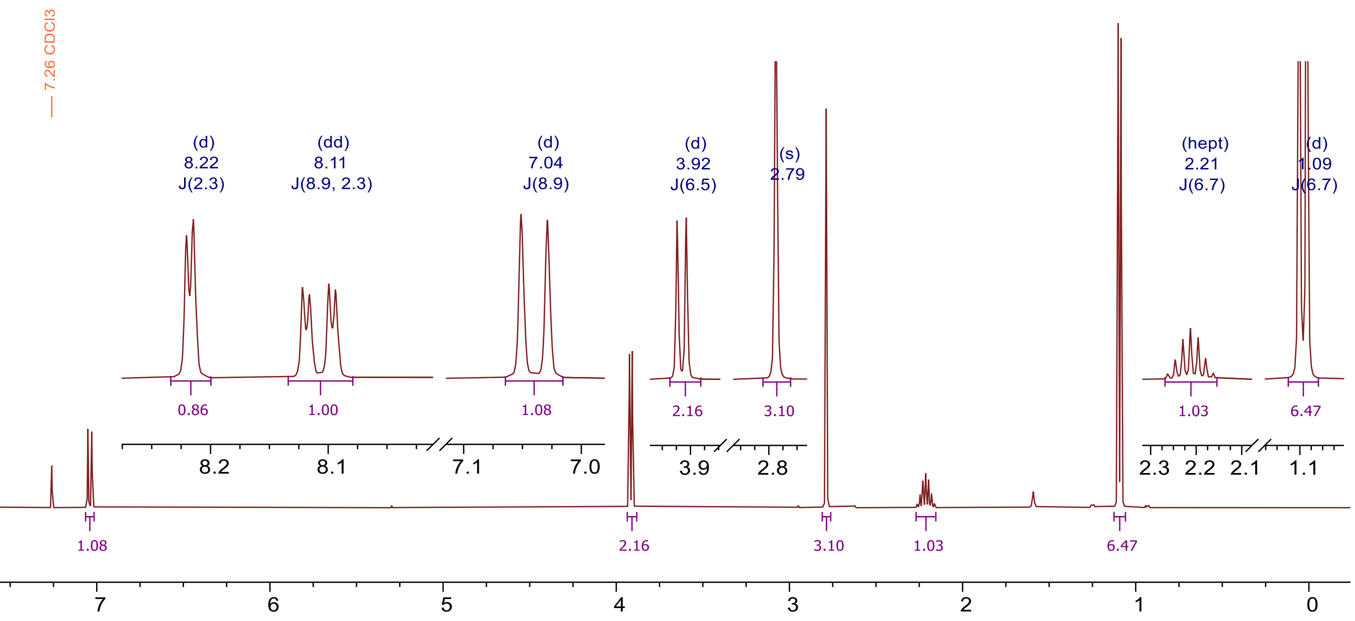

^{13}C NMR (100 MHz, $CDCl_3$):

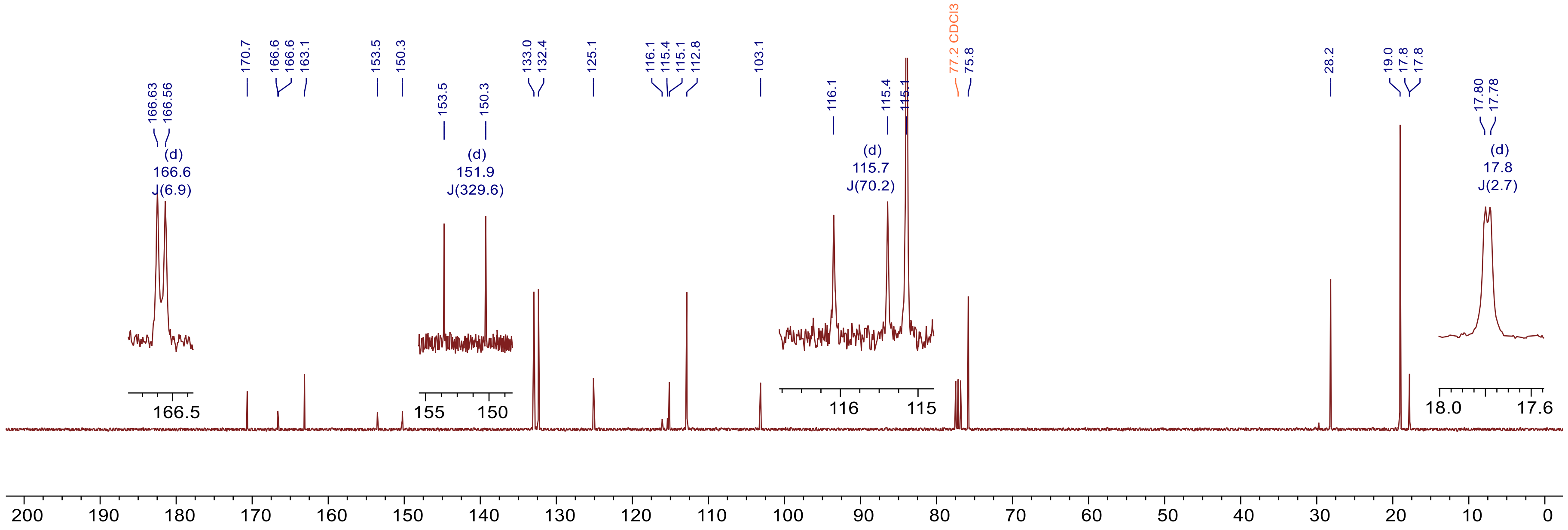

^{19}F NMR (376 MHz, $CDCl_3$):

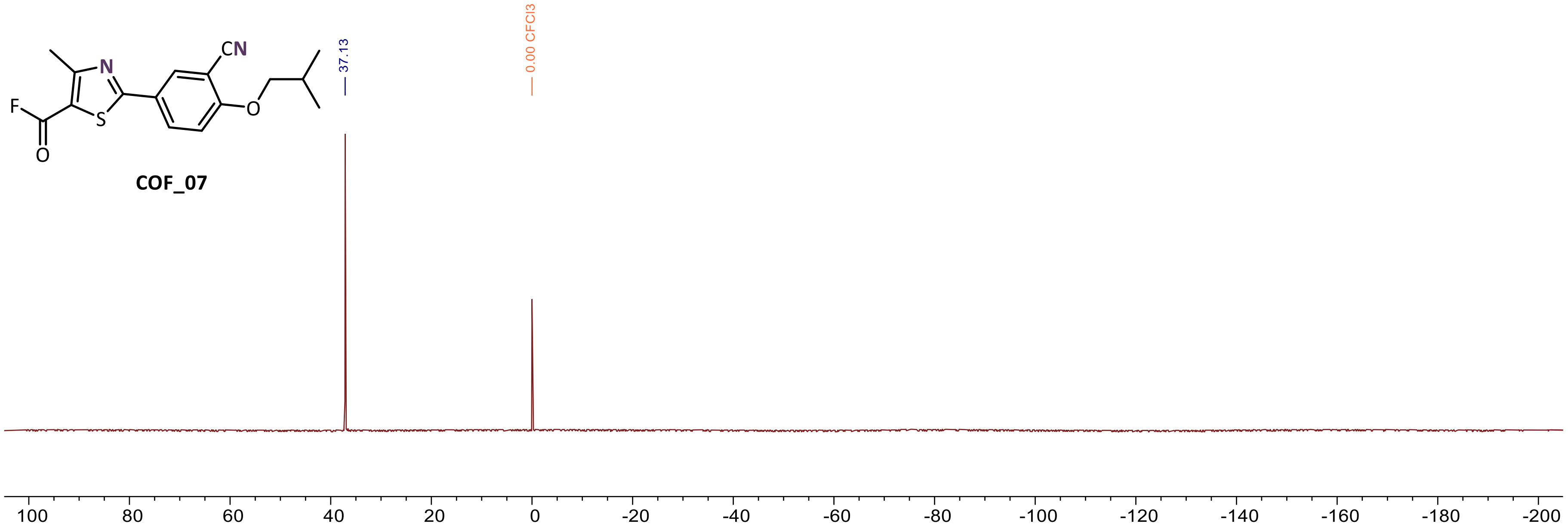

COF_07

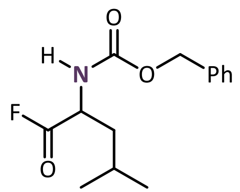

COF_08

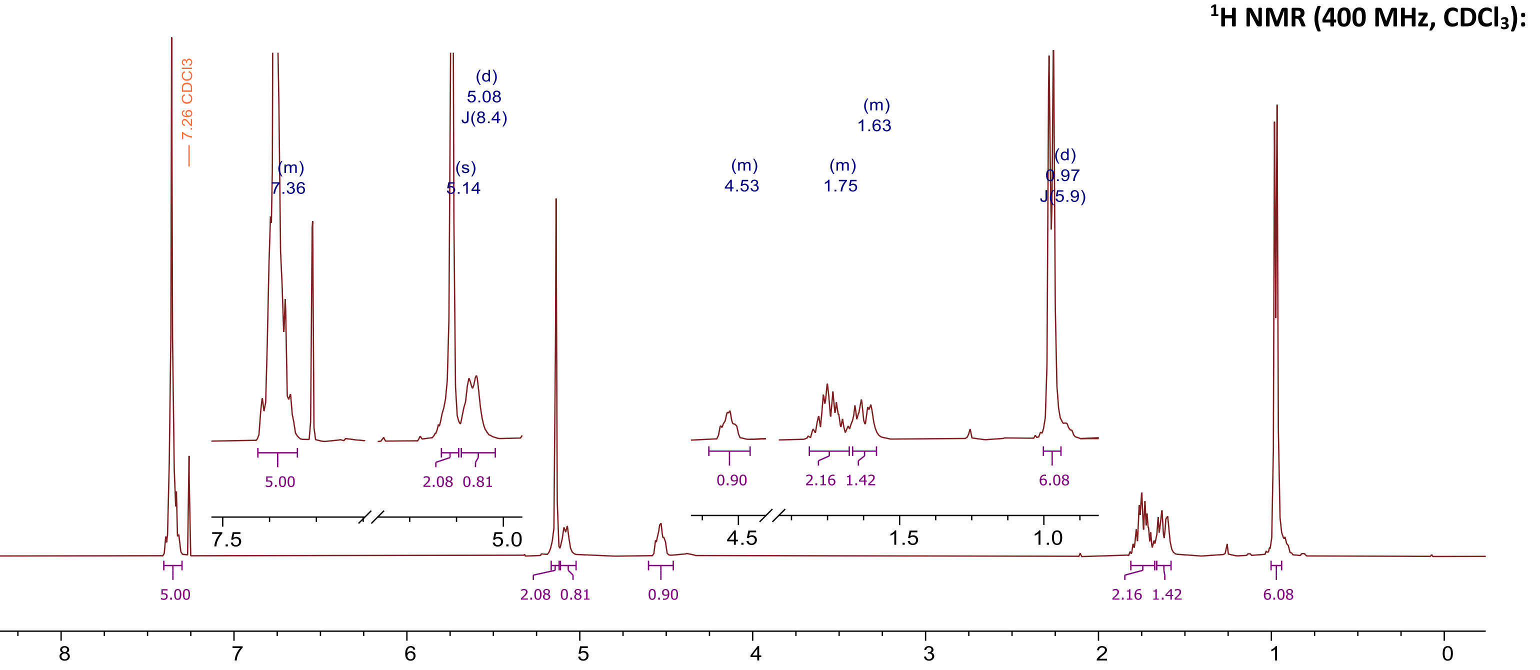

^{1}H NMR (400 MHz, CDCl$_3$):
7.26 CDCl3
(m) 7.36
(d) 5.08 J(8.4)
(s) 5.14
(m) 4.53
(m) 1.75
(m) 1.63
(d) 0.97 J(5.9)
5.00
2.08 0.81
0.90
2.16 1.42
6.08

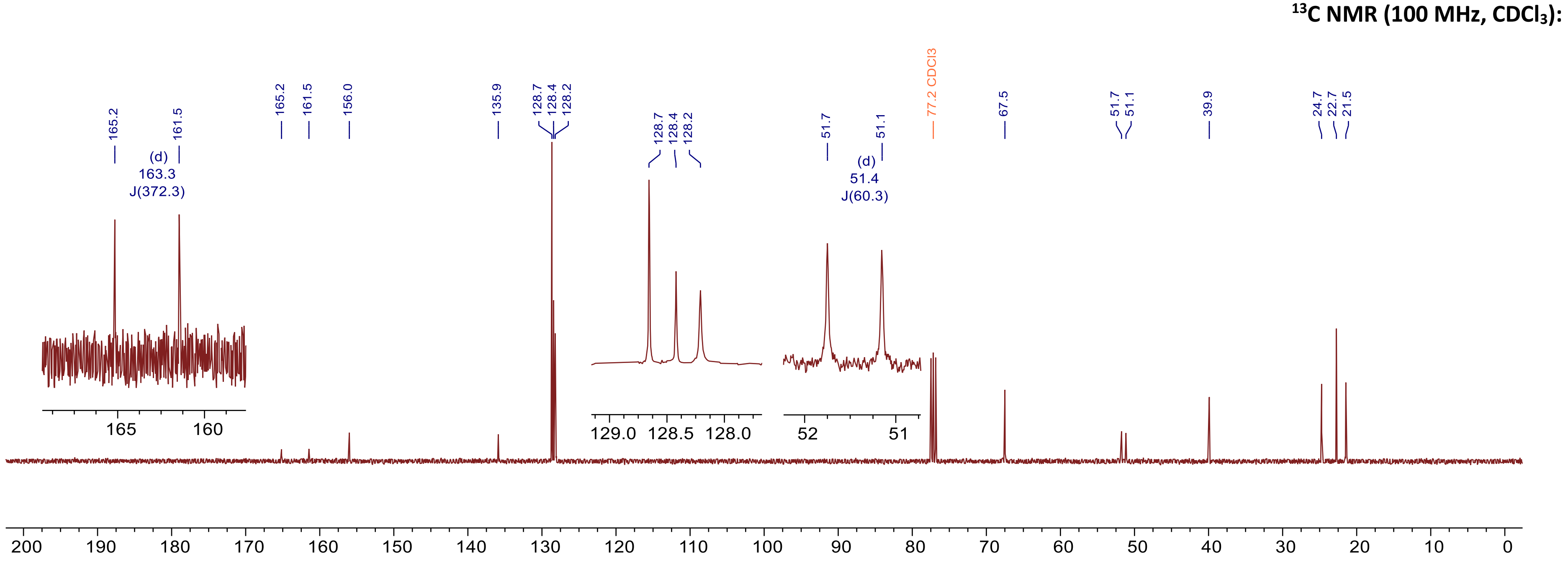

^{13}C NMR (100 MHz, CDCl$_3$):
165.2
161.5
(d) 163.3 J(372.3)
156.0
135.9
128.7 128.4 128.2
51.7
51.1
(d) 51.4 J(60.3)
77.2 CDCl3
67.5
39.9
24.7 22.7 21.5

^{19}F NMR (376 MHz, CDCl$_3$):

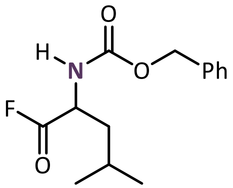

COF_08

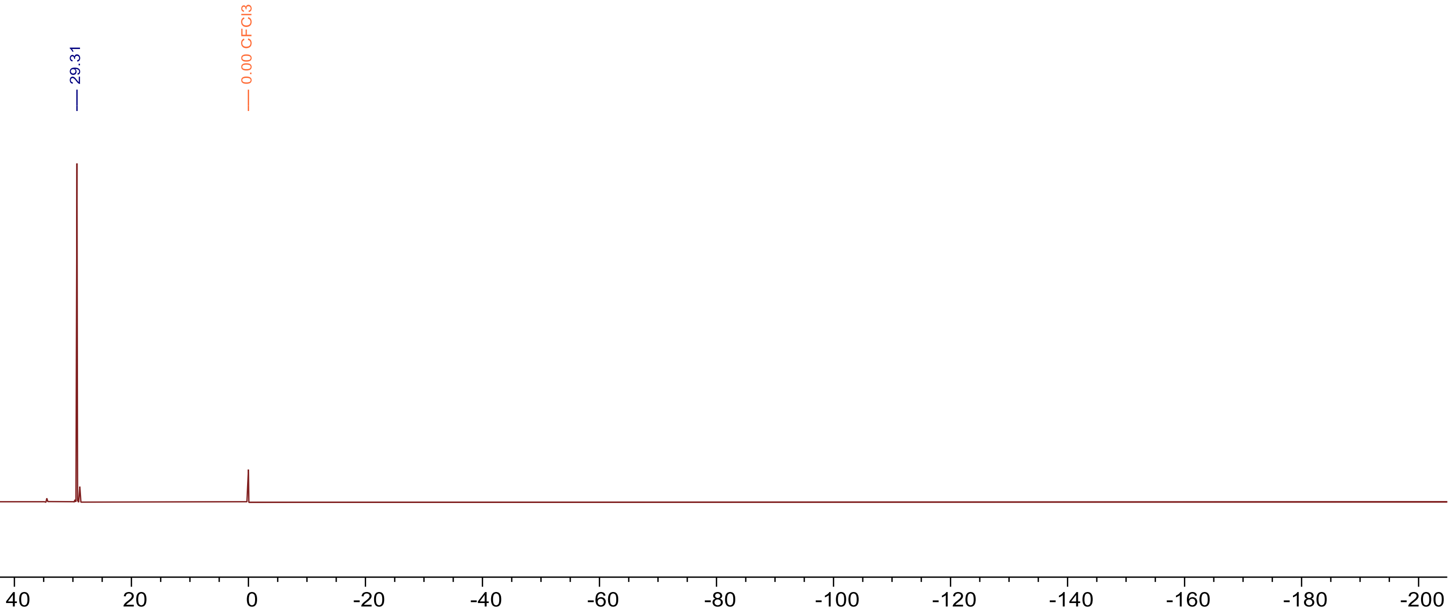

^{1}H NMR (400 MHz, $CDCl_3$):

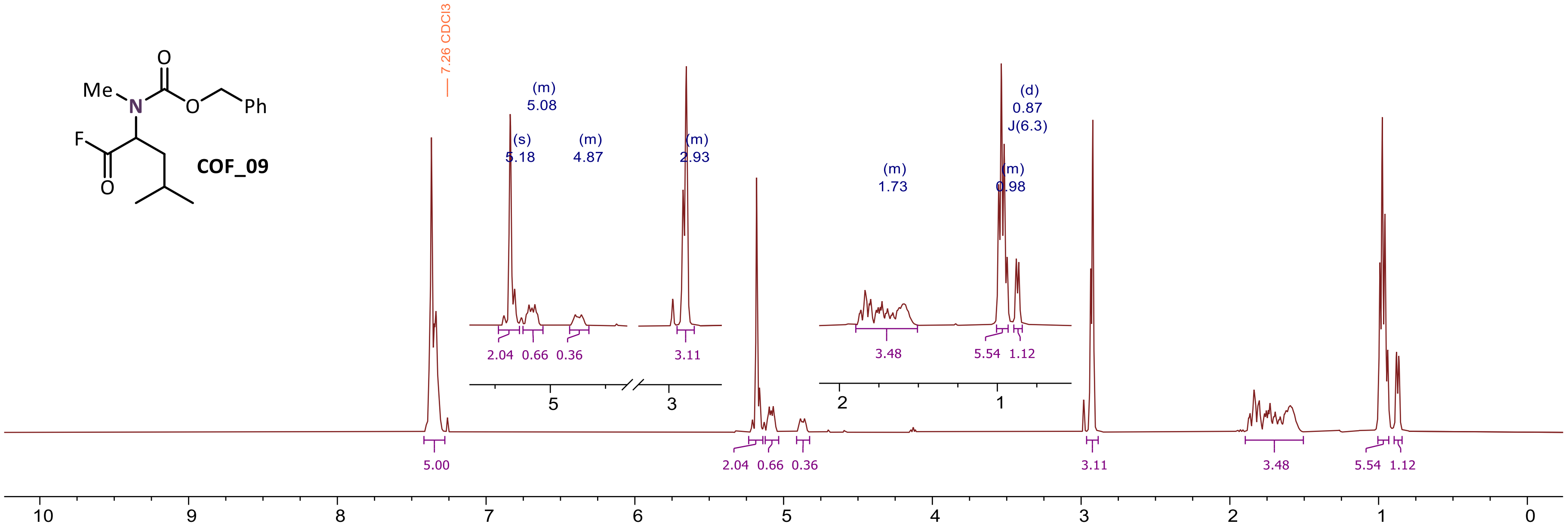

^{13}C NMR (100 MHz, $CDCl_3$):

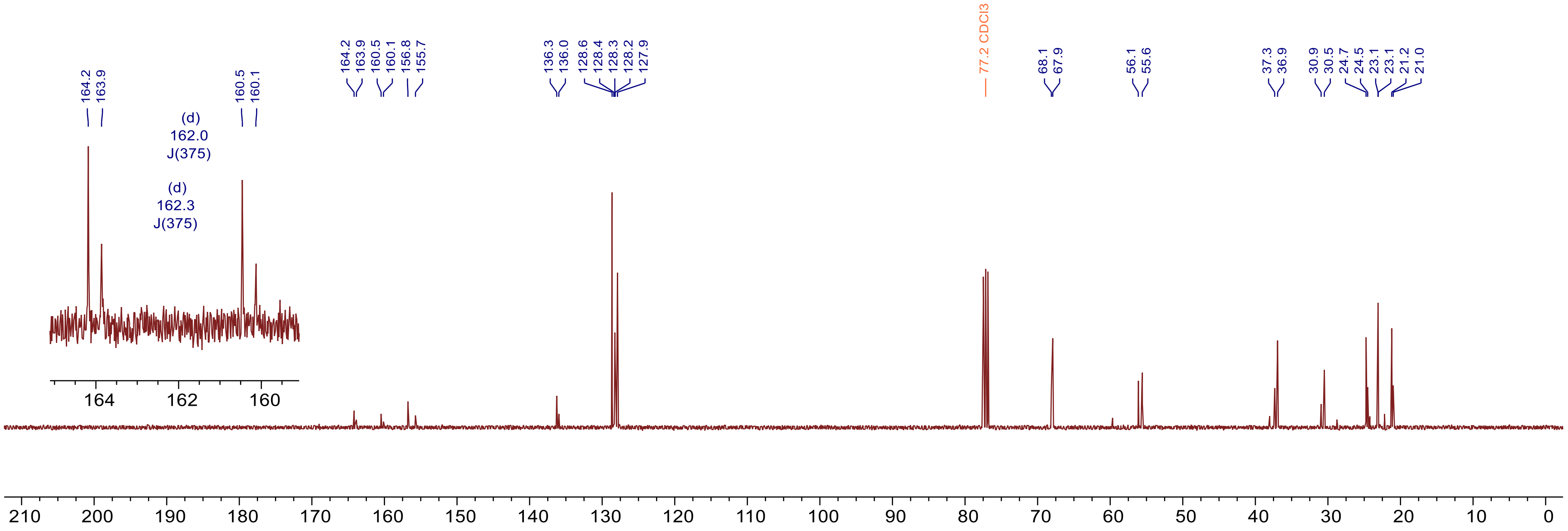

^{19}F NMR (376 MHz, $CDCl_3$):

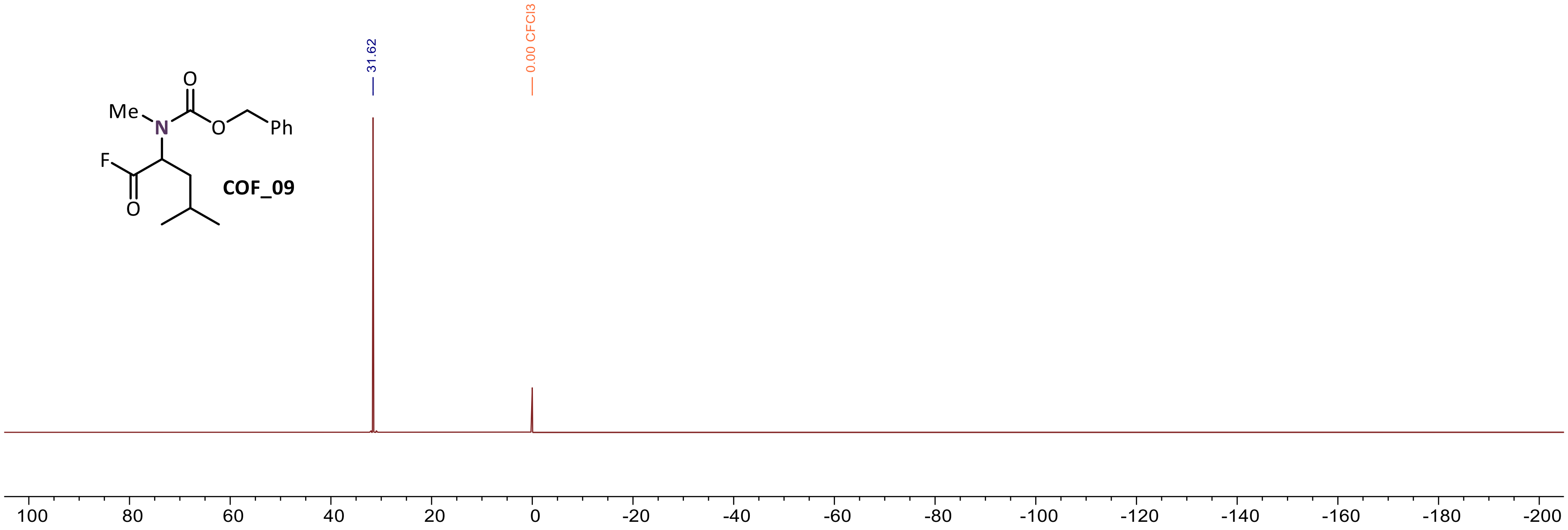

^1H NMR (400 MHz, $CDCl_3$):

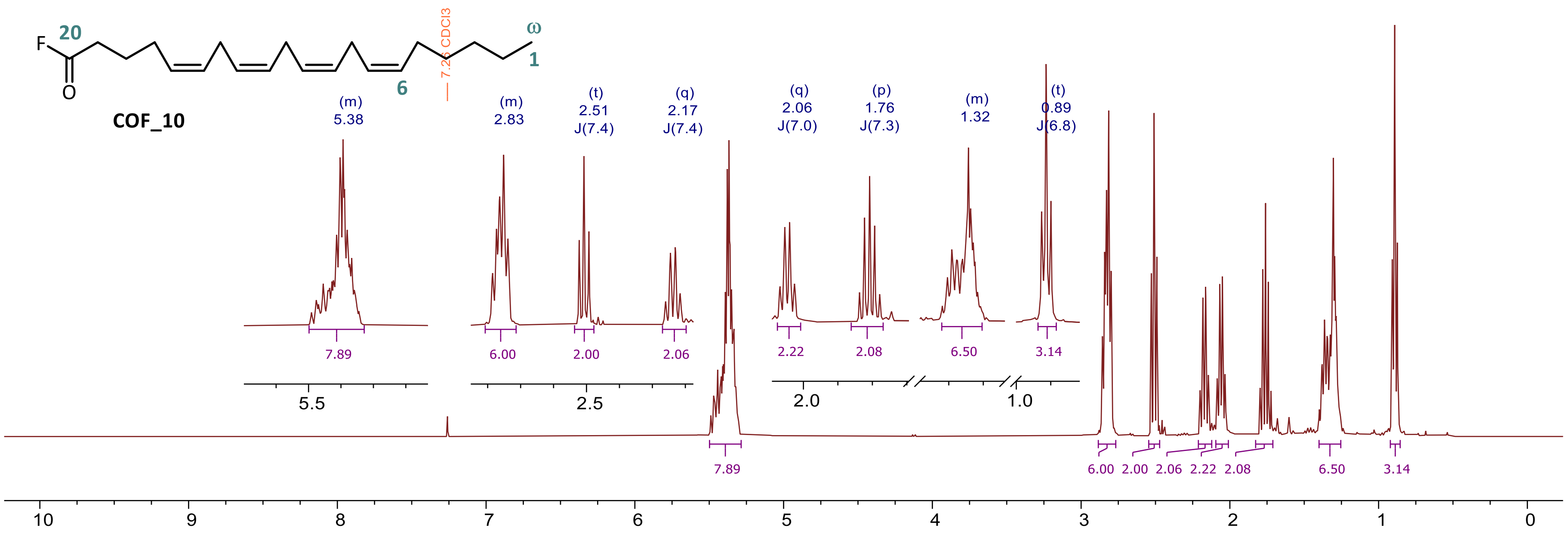

^{13}C NMR (100 MHz, $CDCl_3$):

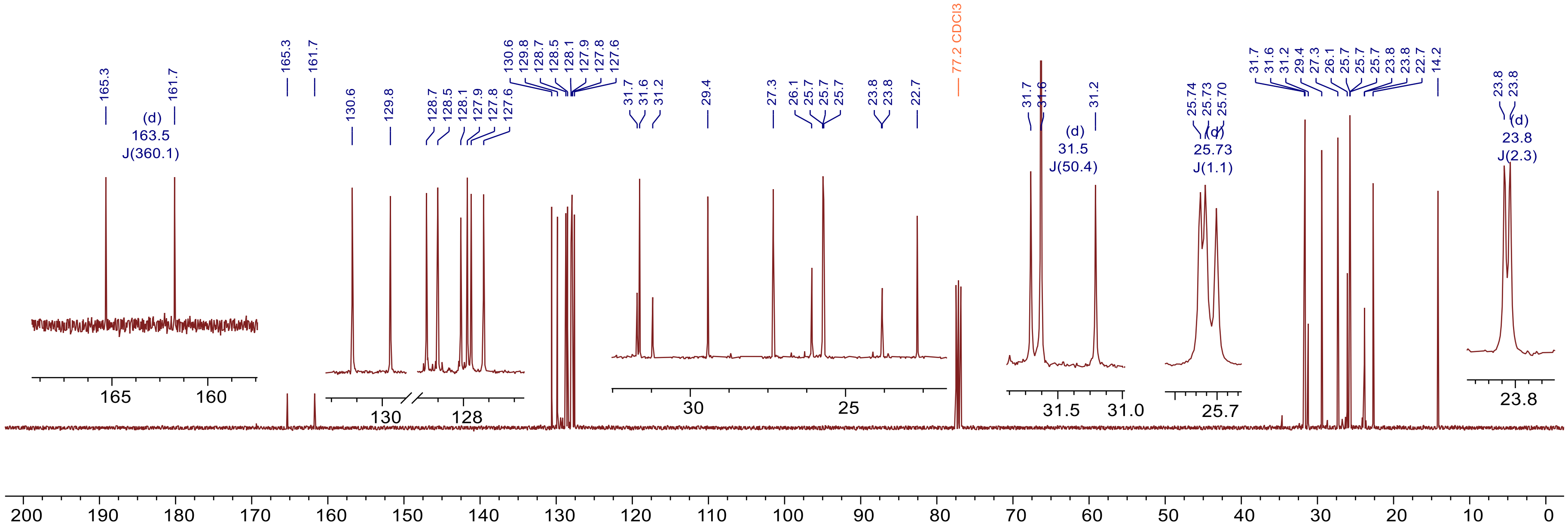

^{19}F NMR (376 MHz, CDCl$_3$):

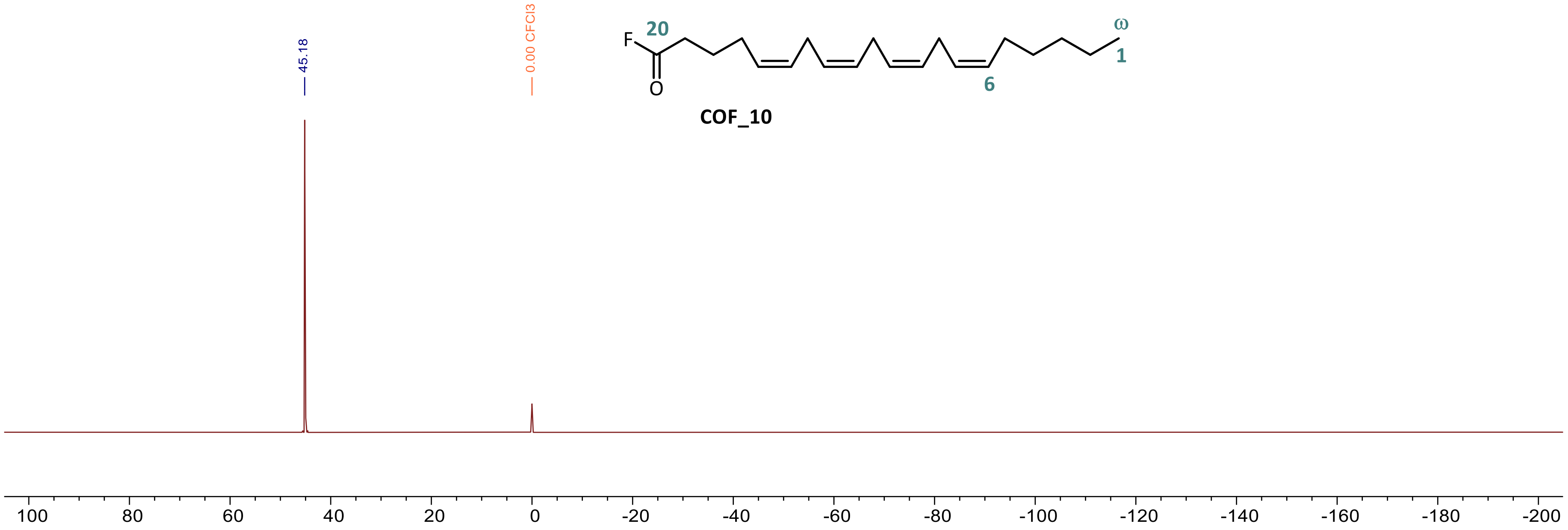

^{1}H NMR (400 MHz, $CDCl_3$):

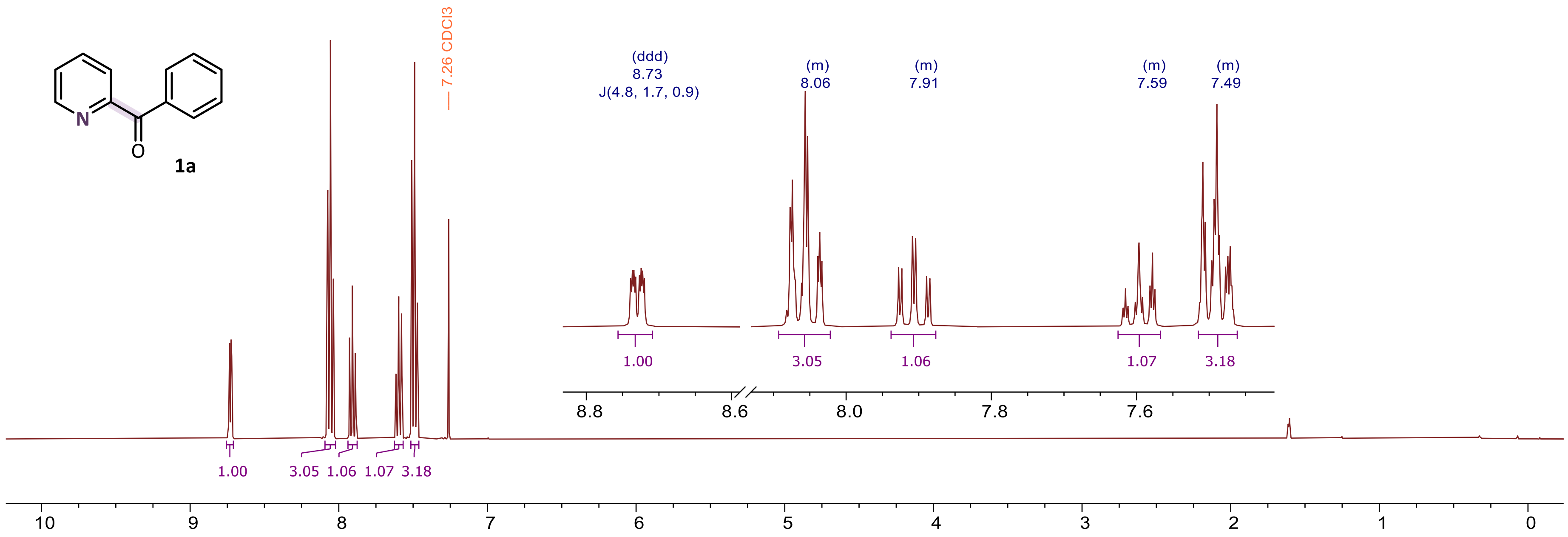

^{13}C NMR (100 MHz, $CDCl_3$):

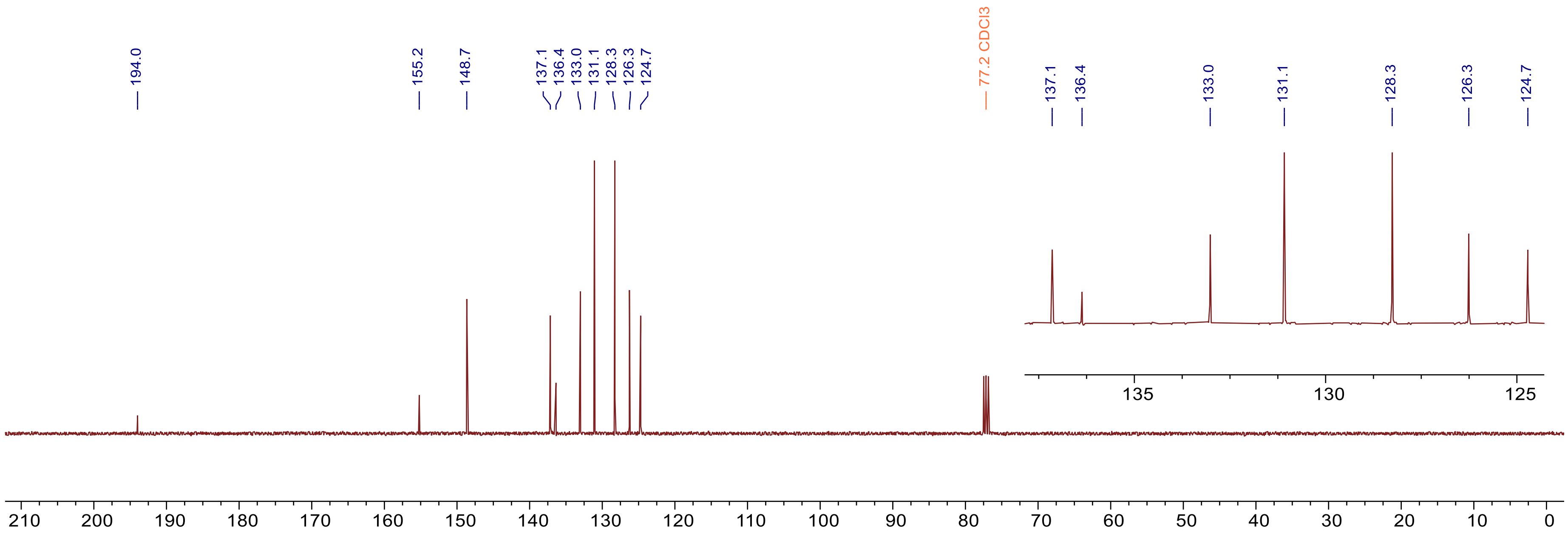

^{1}H NMR (400 MHz, CDCl$_3$):

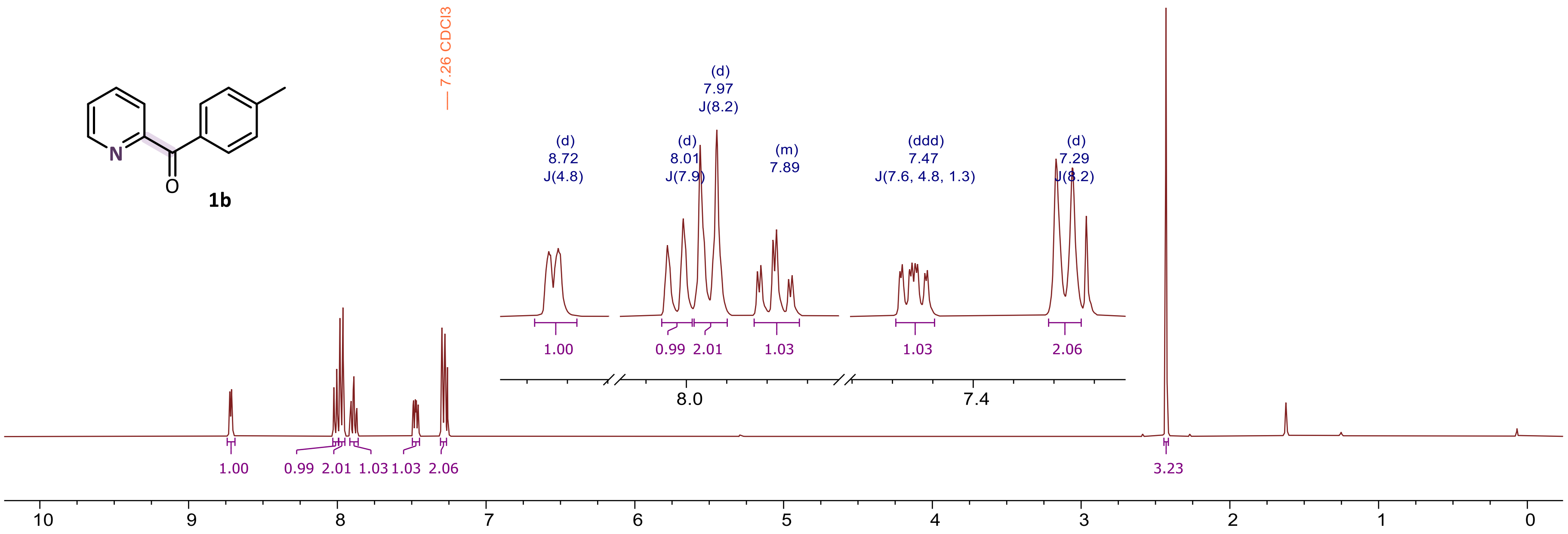

^{13}C NMR (100 MHz, CDCl$_3$):

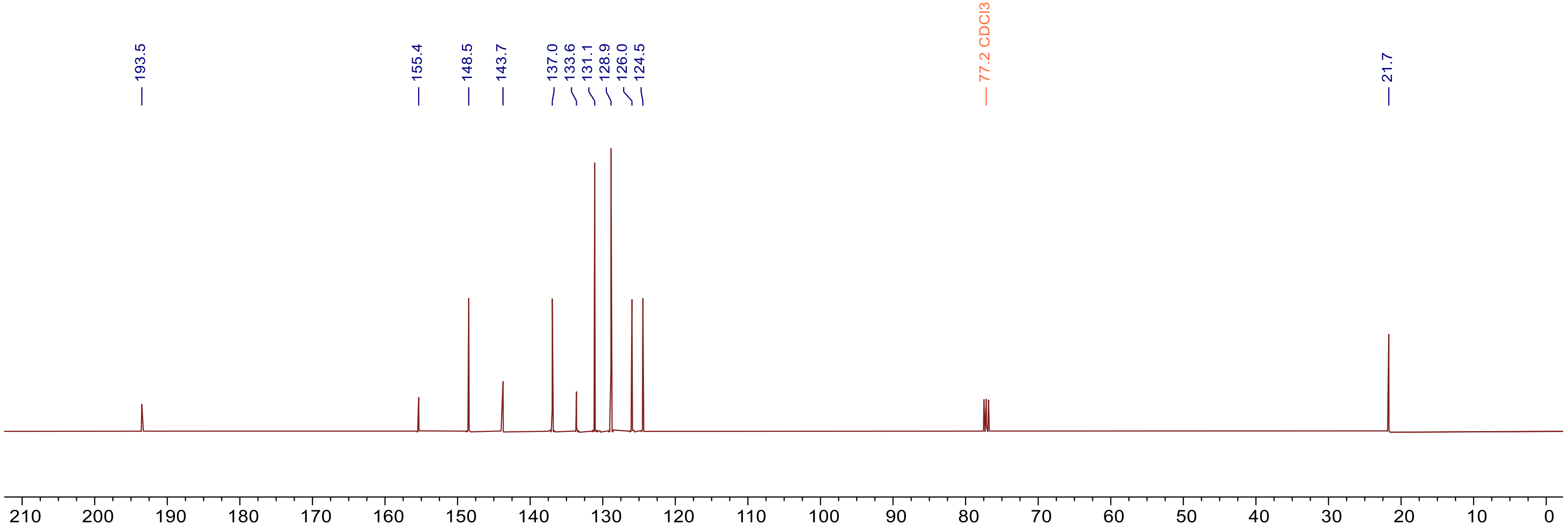

^1H NMR (400 MHz, $CDCl_3$):

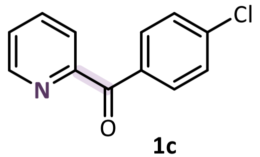

1c

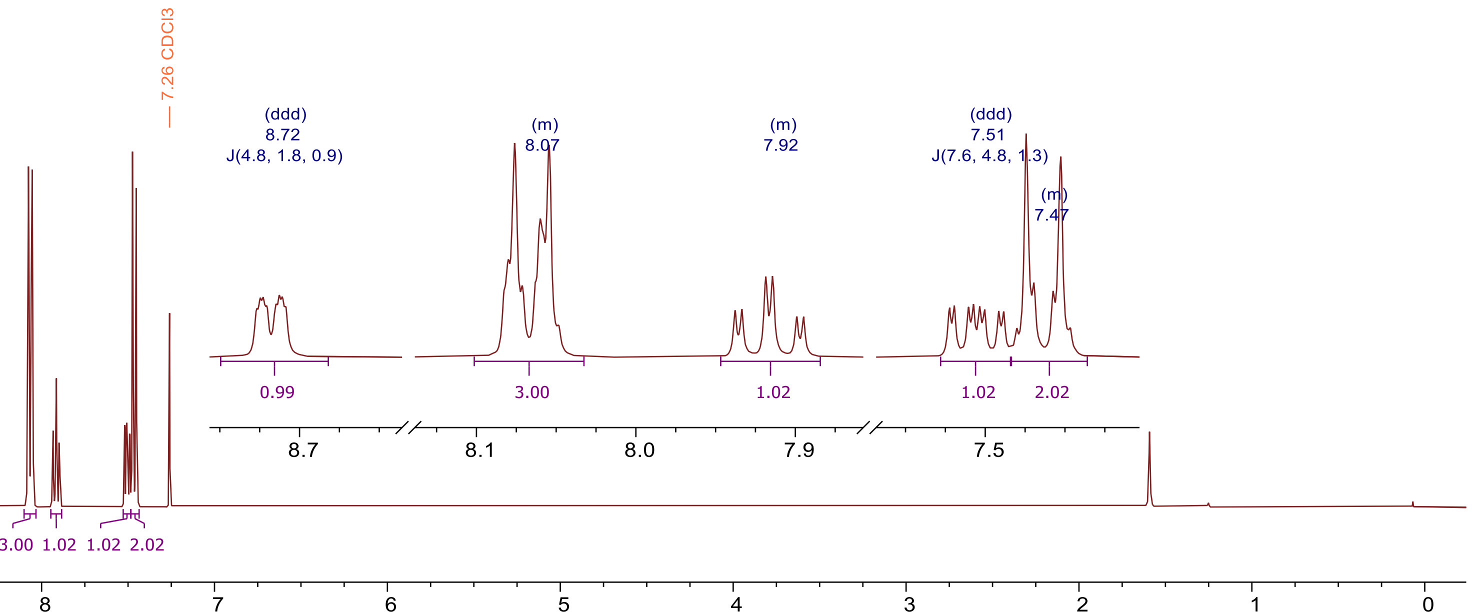

^{13}C NMR (100 MHz, $CDCl_3$):

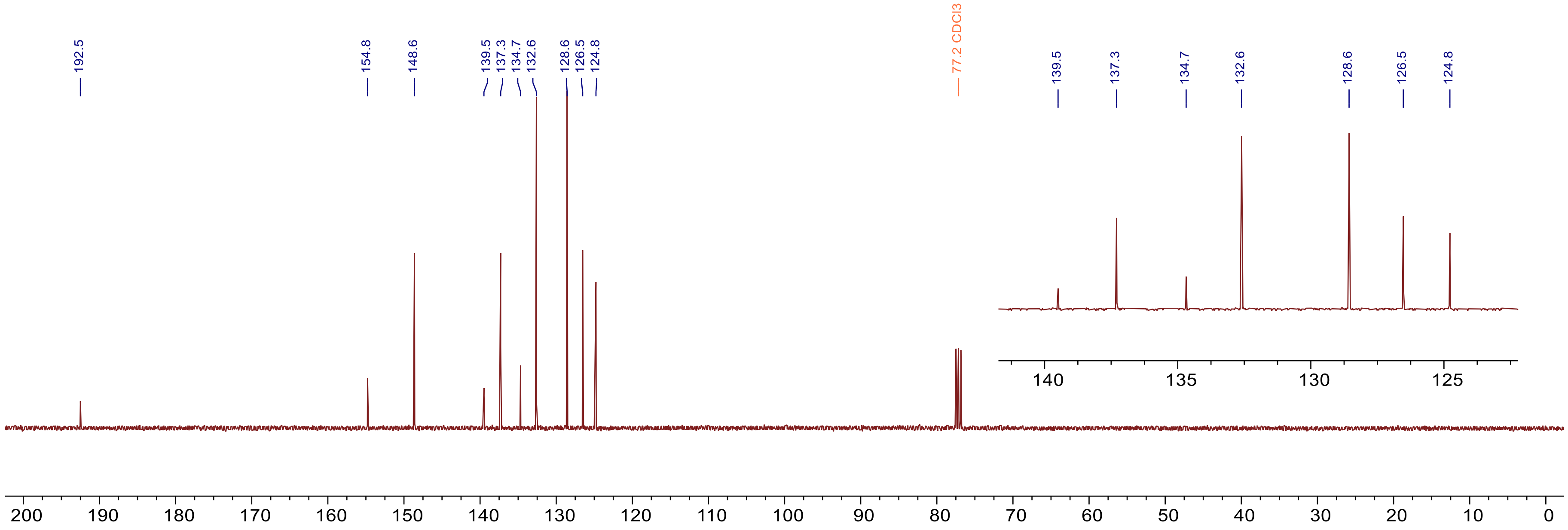

^{1}H NMR (400 MHz, $CDCl_3$):

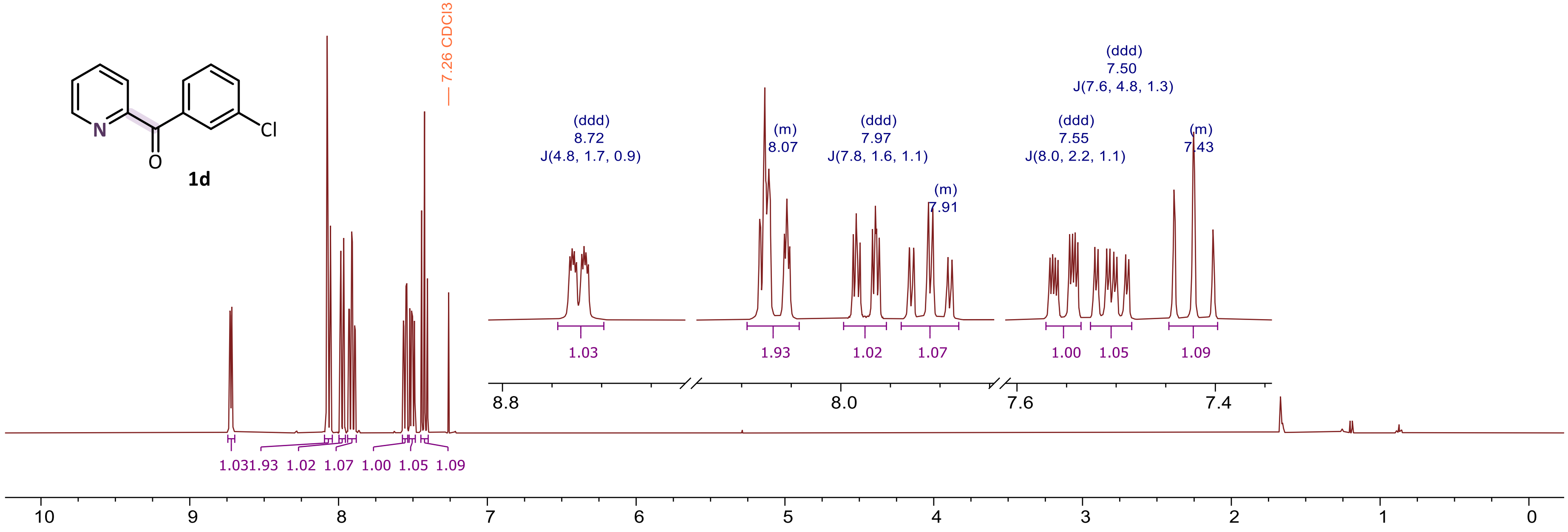

^{13}C NMR (100 MHz, $CDCl_3$):

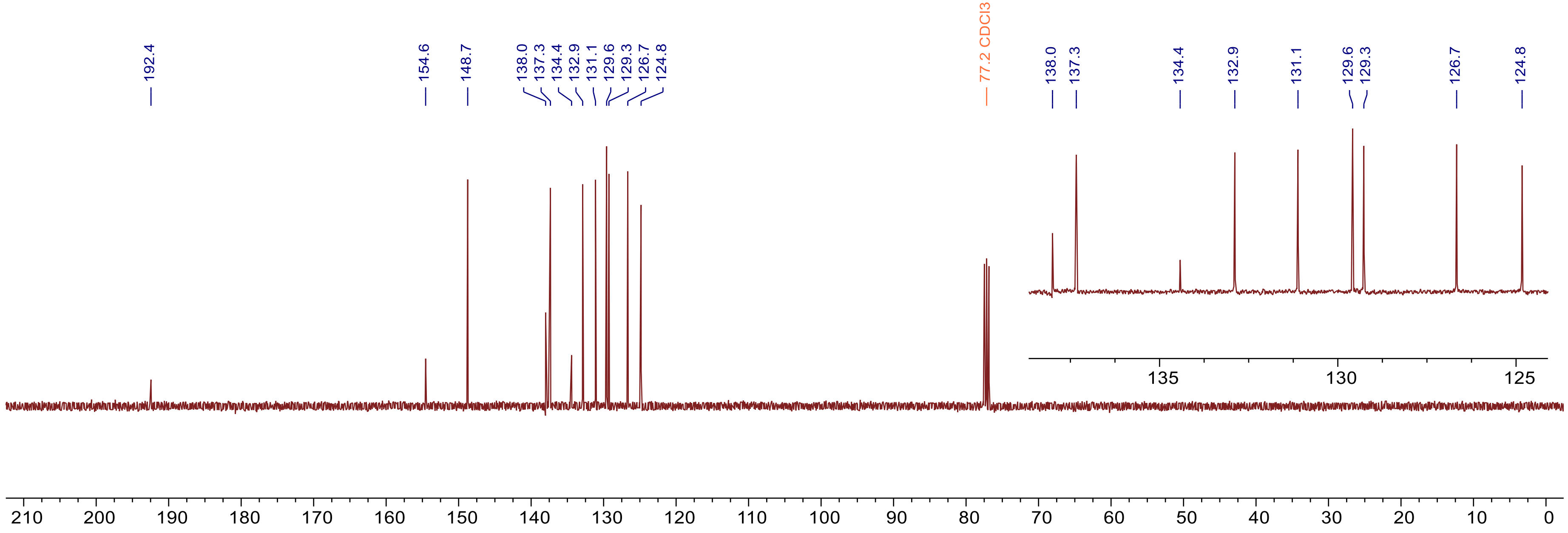

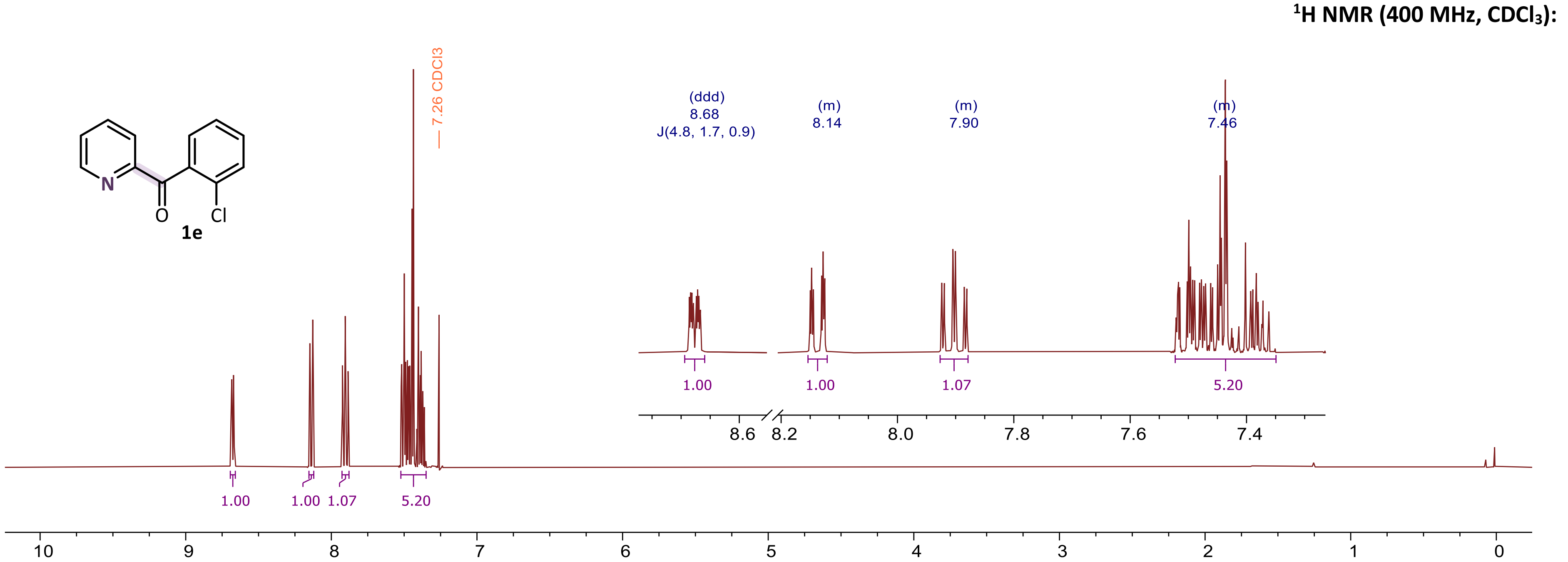

^{1}H NMR (400 MHz, CDCl$_3$):
1e
7.26 CDCl3
(ddd) 8.68 J(4.8, 1.7, 0.9)
(m) 8.14
(m) 7.90
(m) 7.46
1.00
1.00
1.07
5.20

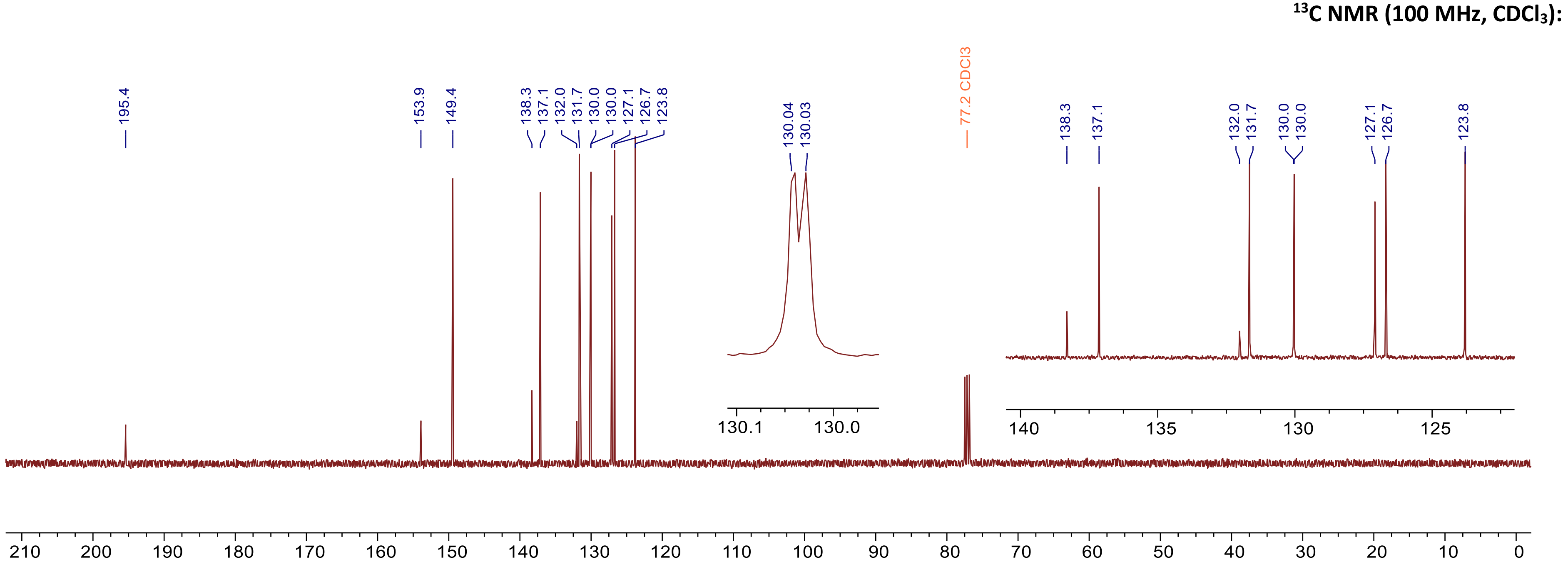

^{13}C NMR (100 MHz, CDCl$_3$):
195.4
153.9
149.4
138.3
137.1
132.0
131.7
130.0
130.0
127.1
126.7
123.8
130.04
130.03
77.2 CDCl3

^{1}H NMR (400 MHz, $CDCl_3$):

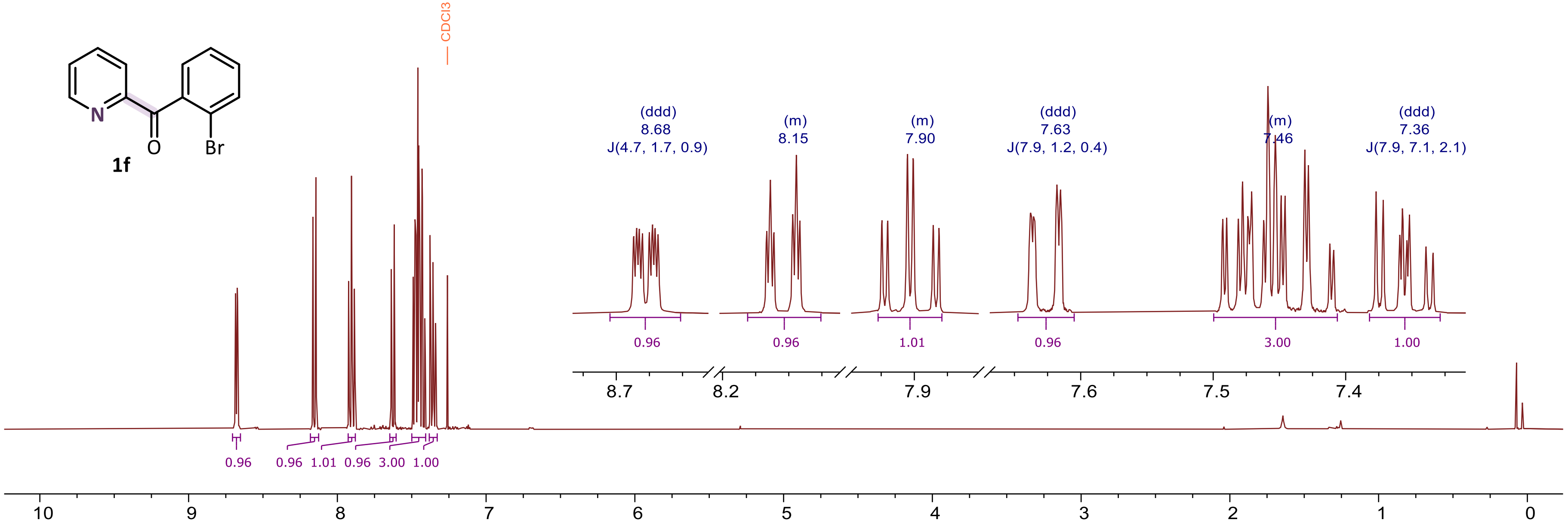

^{13}C NMR (100 MHz, $CDCl_3$):

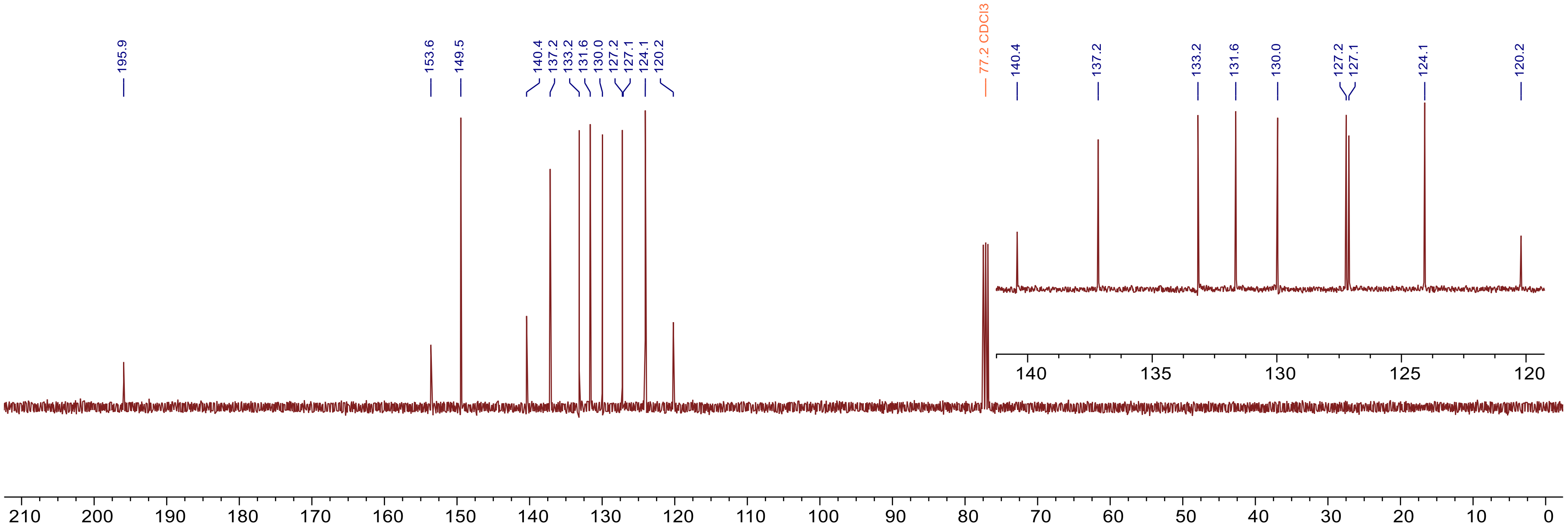

^{1}H NMR (400 MHz, $CDCl_3$):

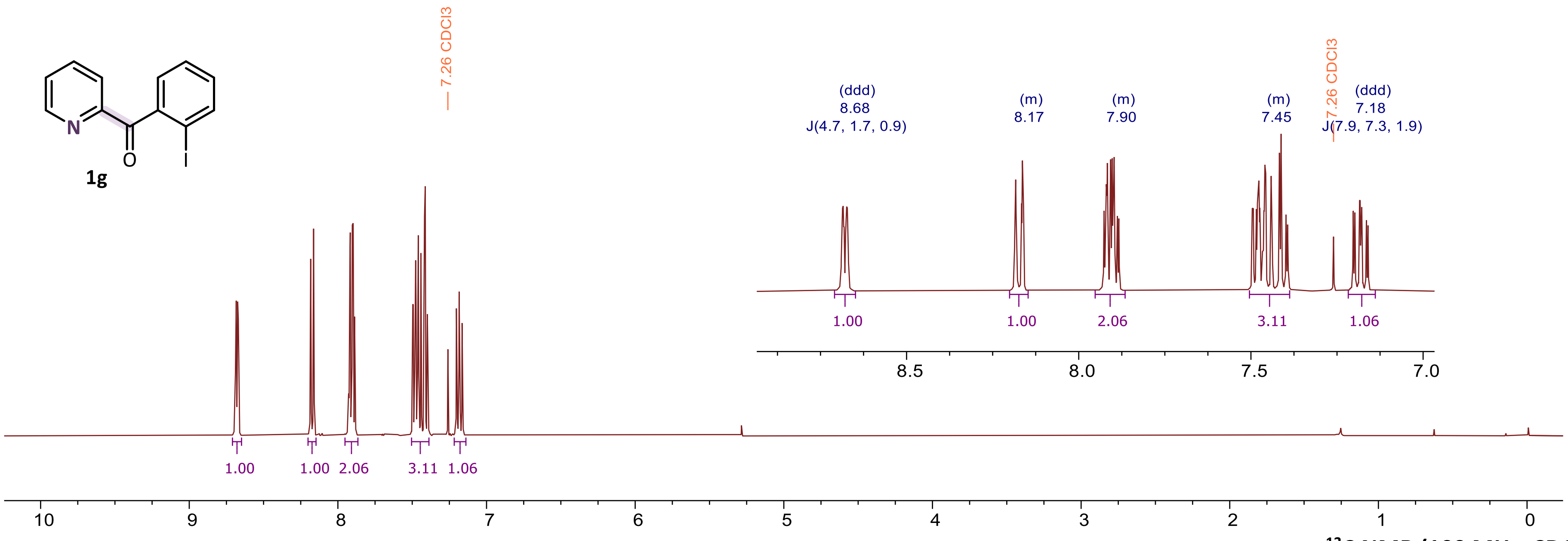

^{13}C NMR (100 MHz, $CDCl_3$):

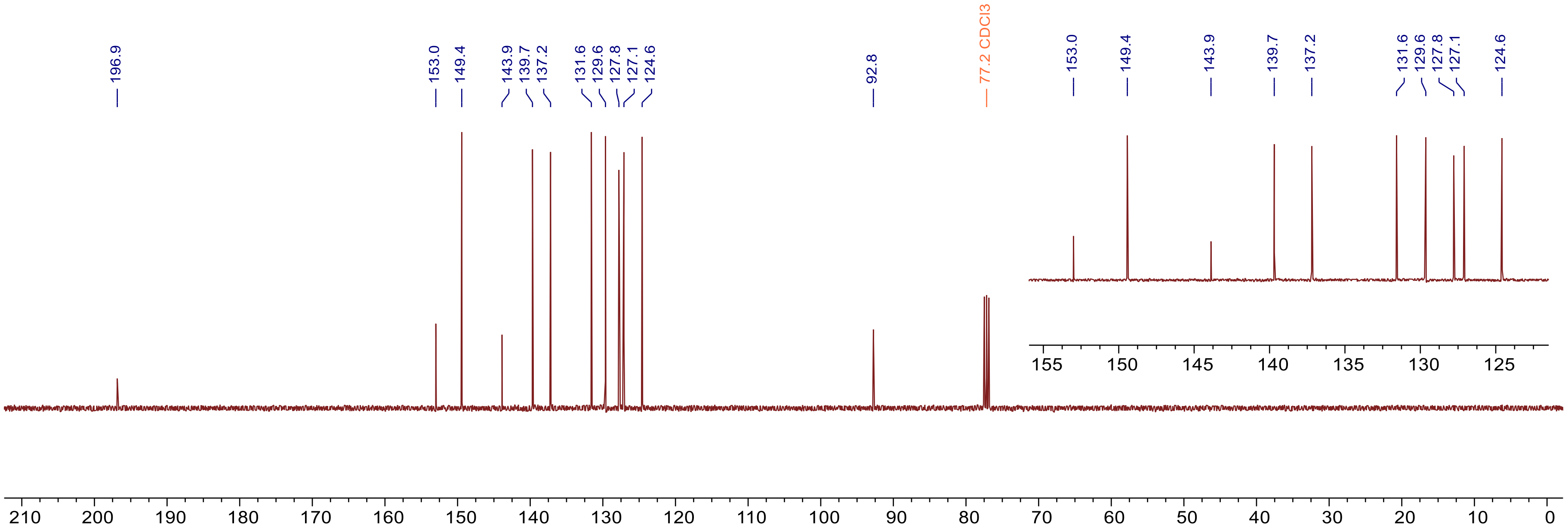

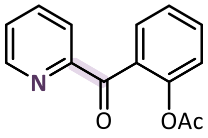

1h

^{1}H NMR (400 MHz, $CDCl_3$):

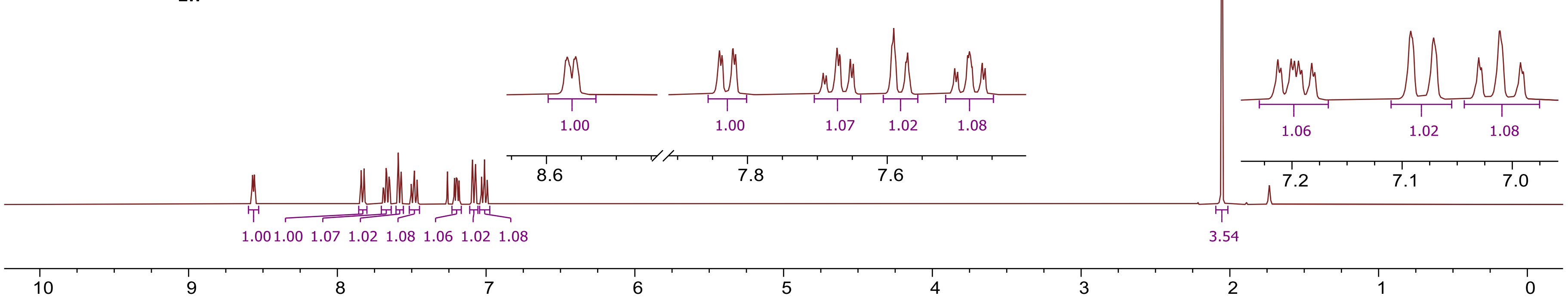

^{13}C NMR (100 MHz, $CDCl_3$):

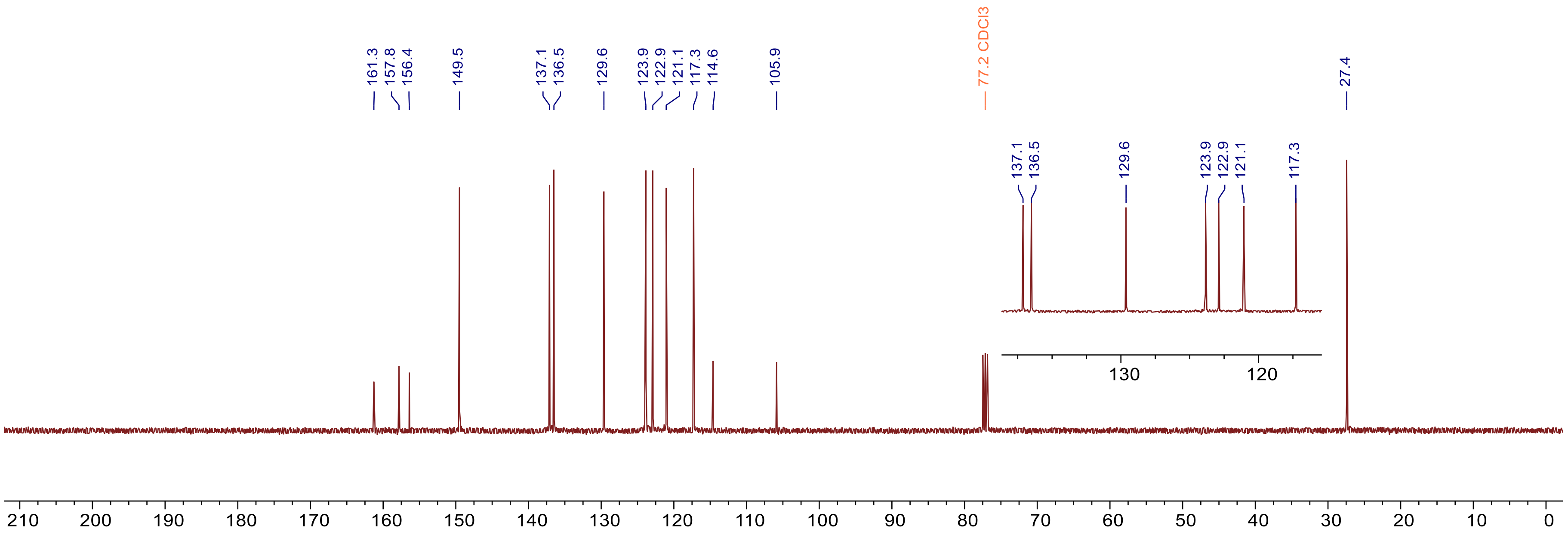

^{1}H NMR (400 MHz, $CDCl_3$):

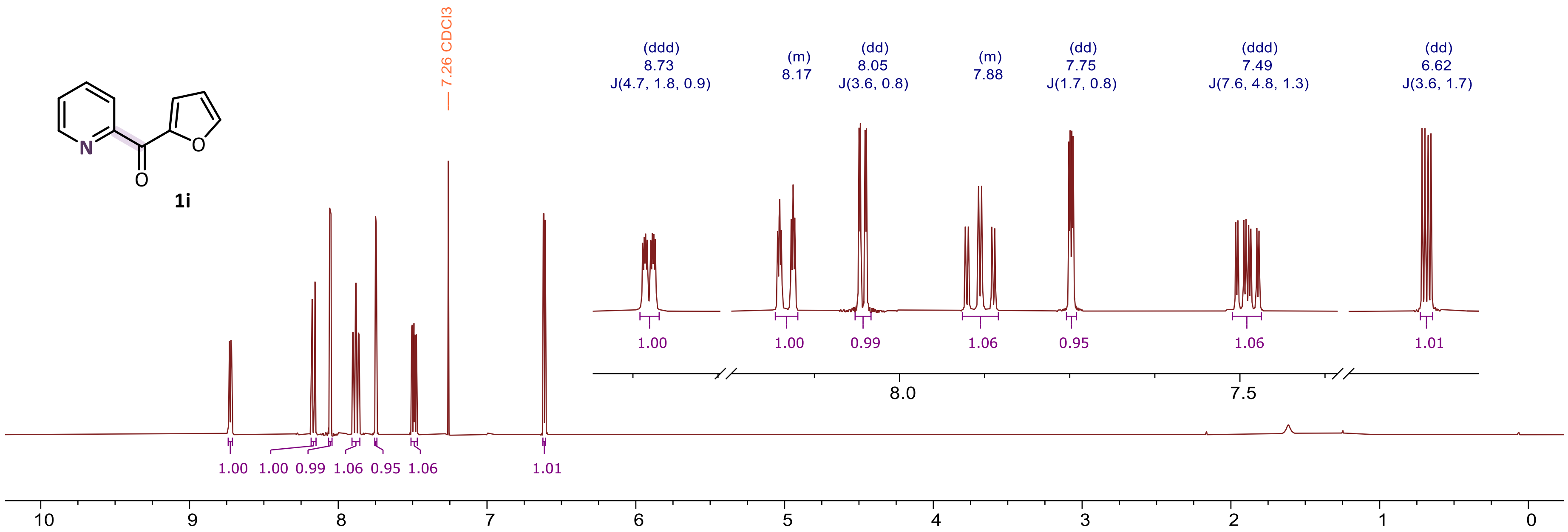

^{13}C NMR (100 MHz, $CDCl_3$):

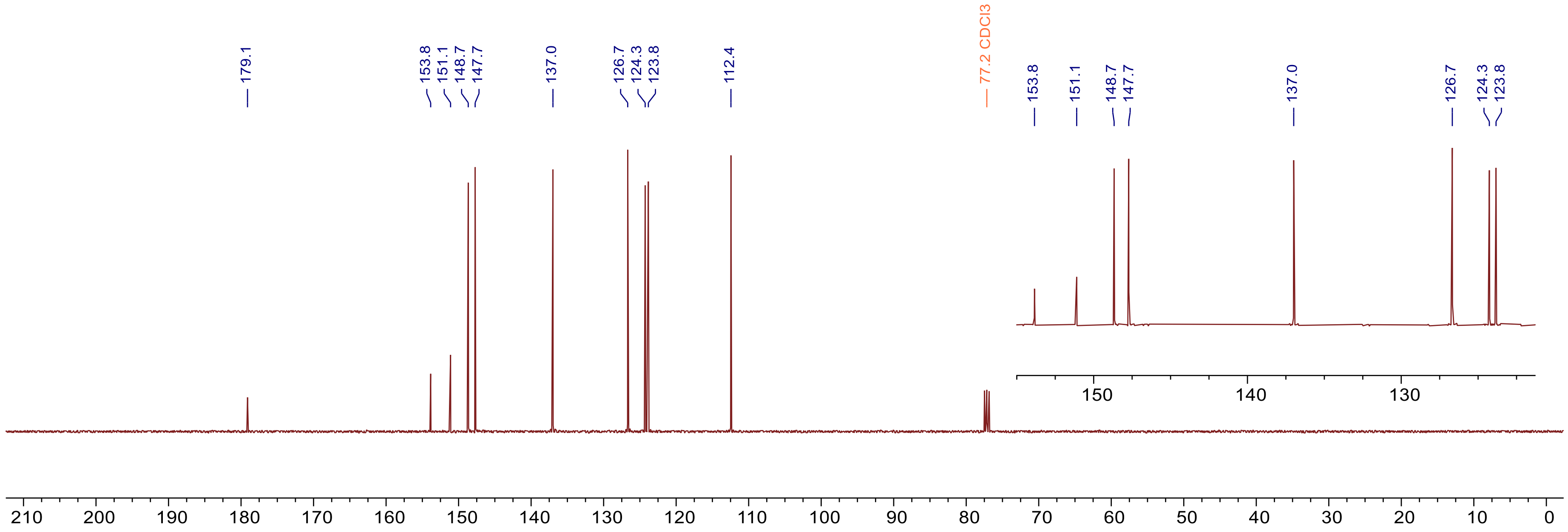

^{1}H NMR (400 MHz, $CDCl_3$):

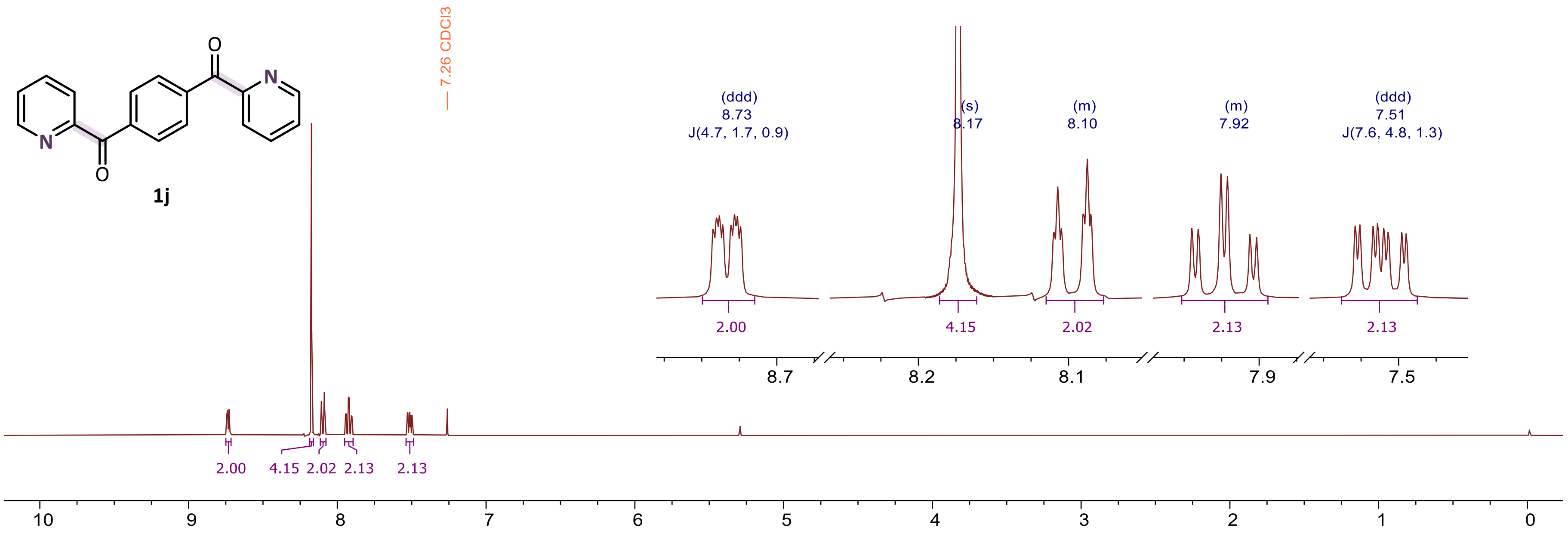

^{13}C NMR (100 MHz, $CDCl_3$):

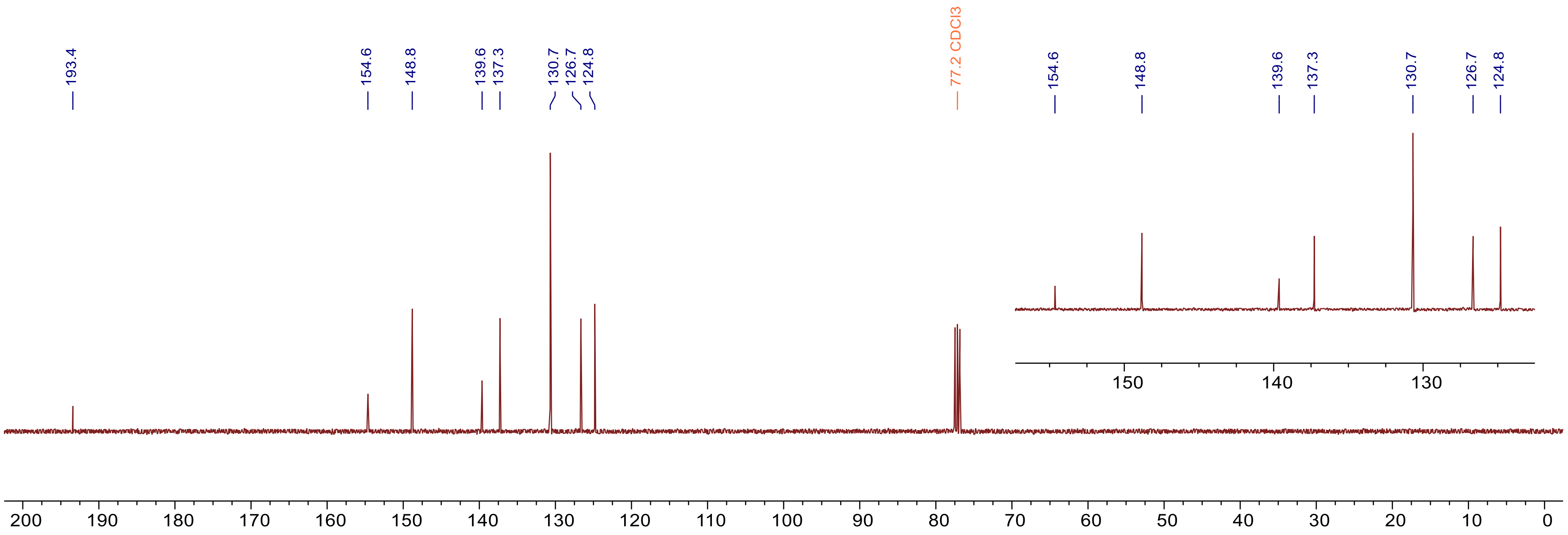

^{1}H NMR (400 MHz, $CDCl_3$):

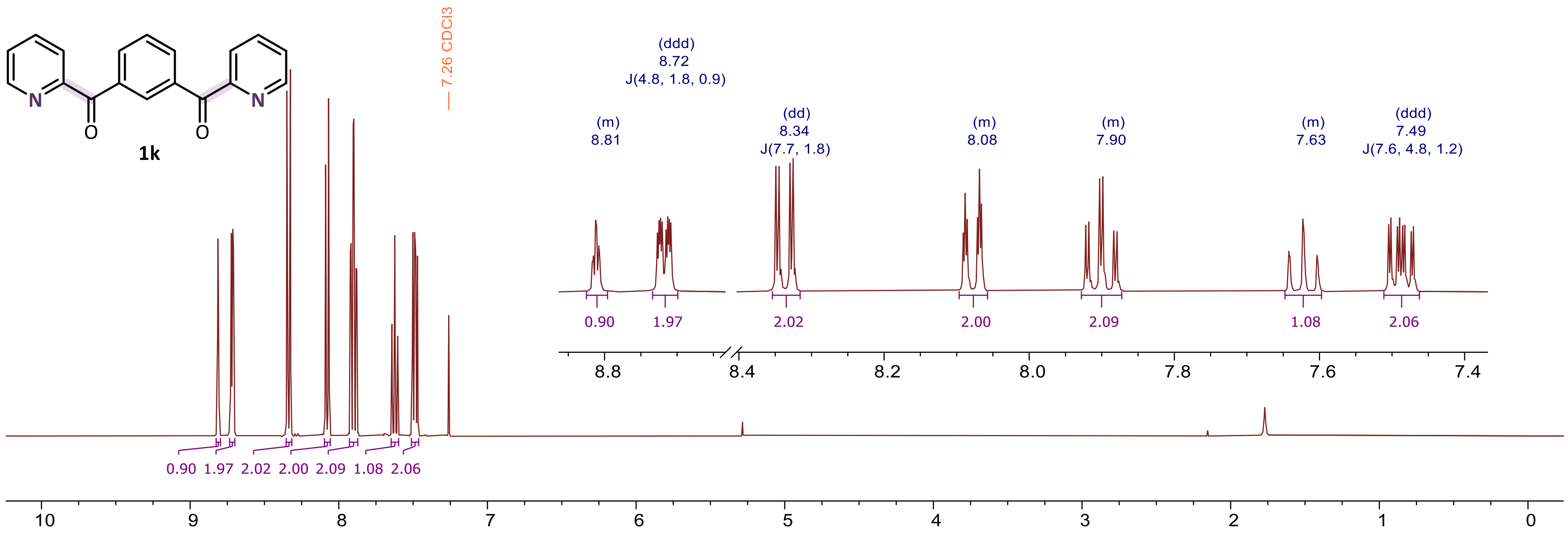

^{13}C NMR (100 MHz, $CDCl_3$):

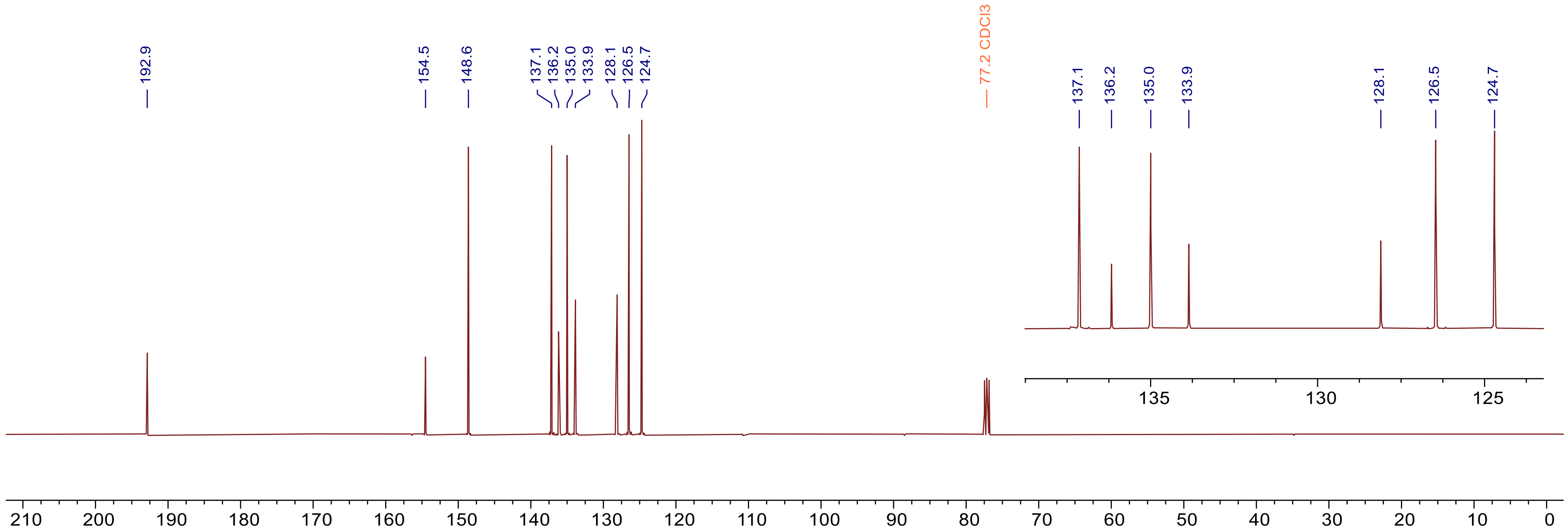

^{1}H NMR (400 MHz, $CDCl_3$):

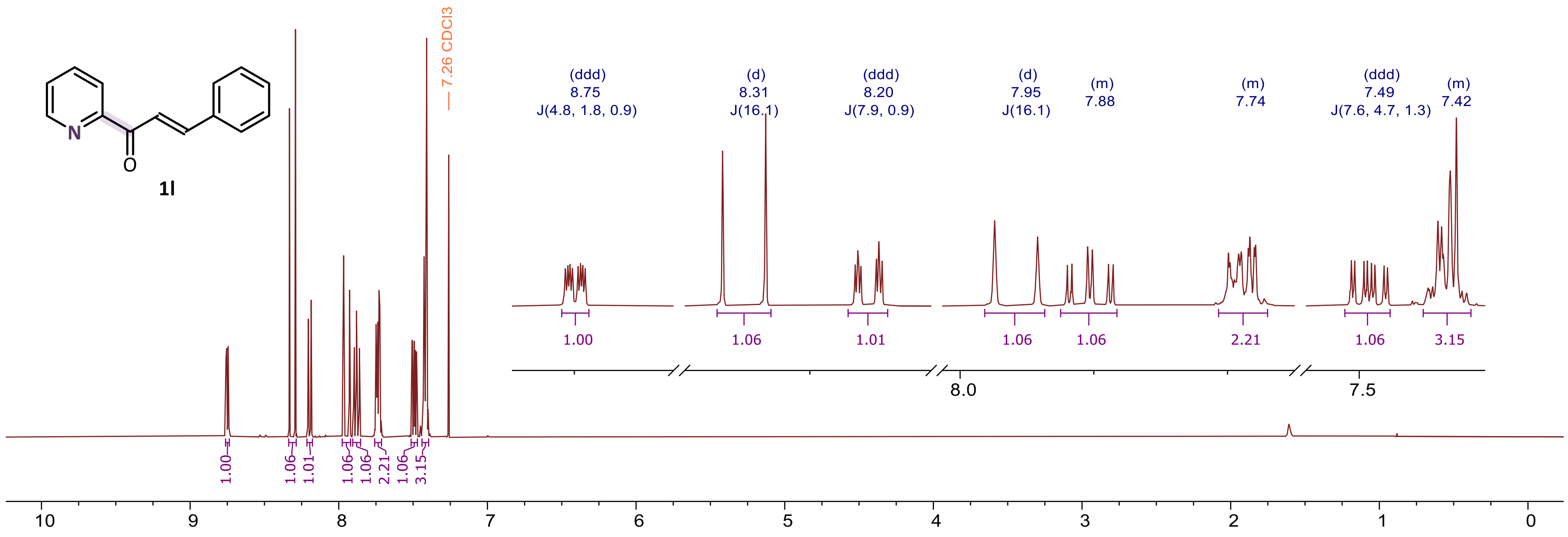

^{13}C NMR (100 MHz, $CDCl_3$):

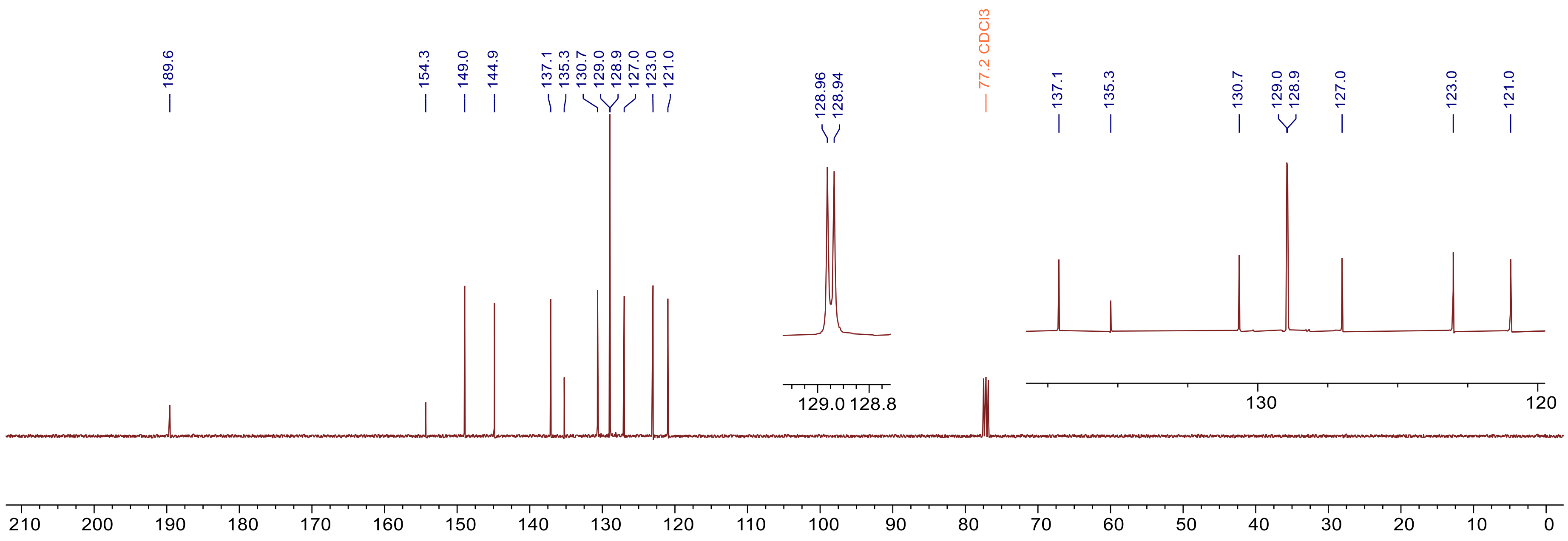

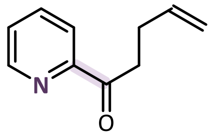

1m

^{1}H NMR (400 MHz, $CDCl_3$):

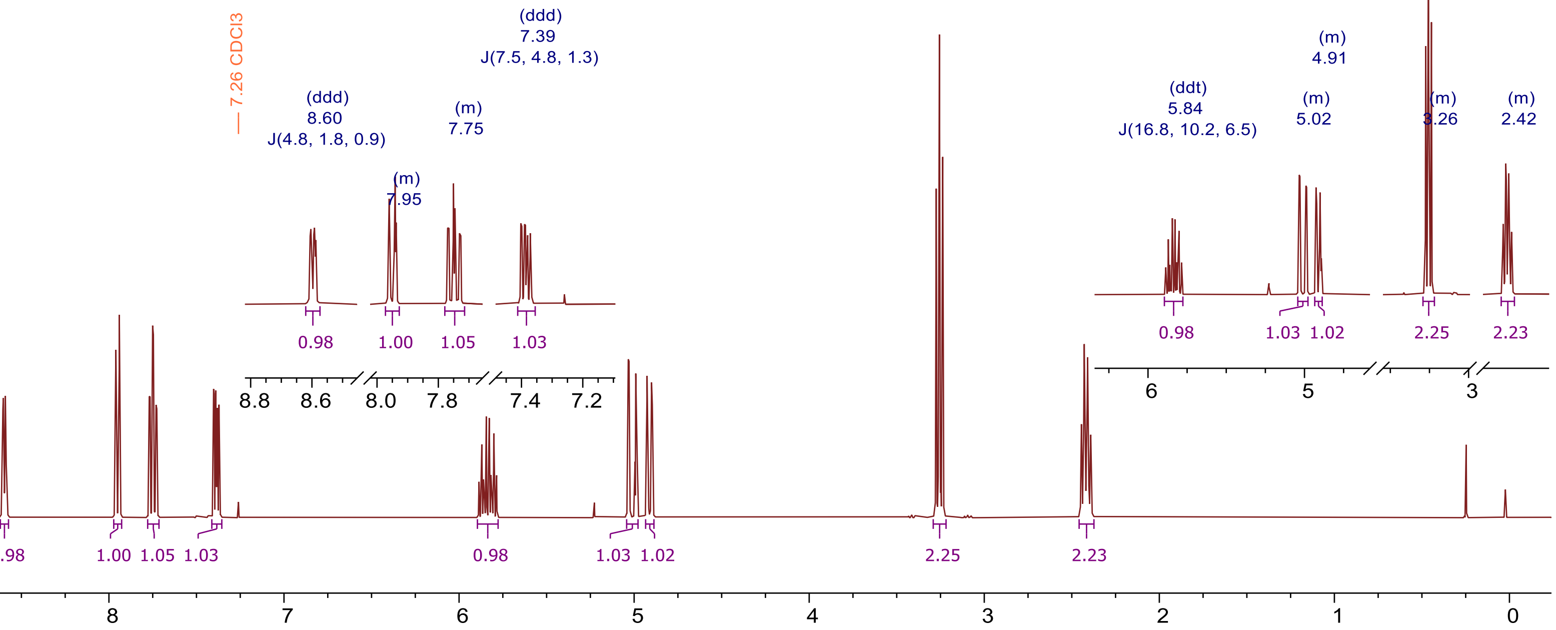

^{13}C NMR (100 MHz, $CDCl_3$):

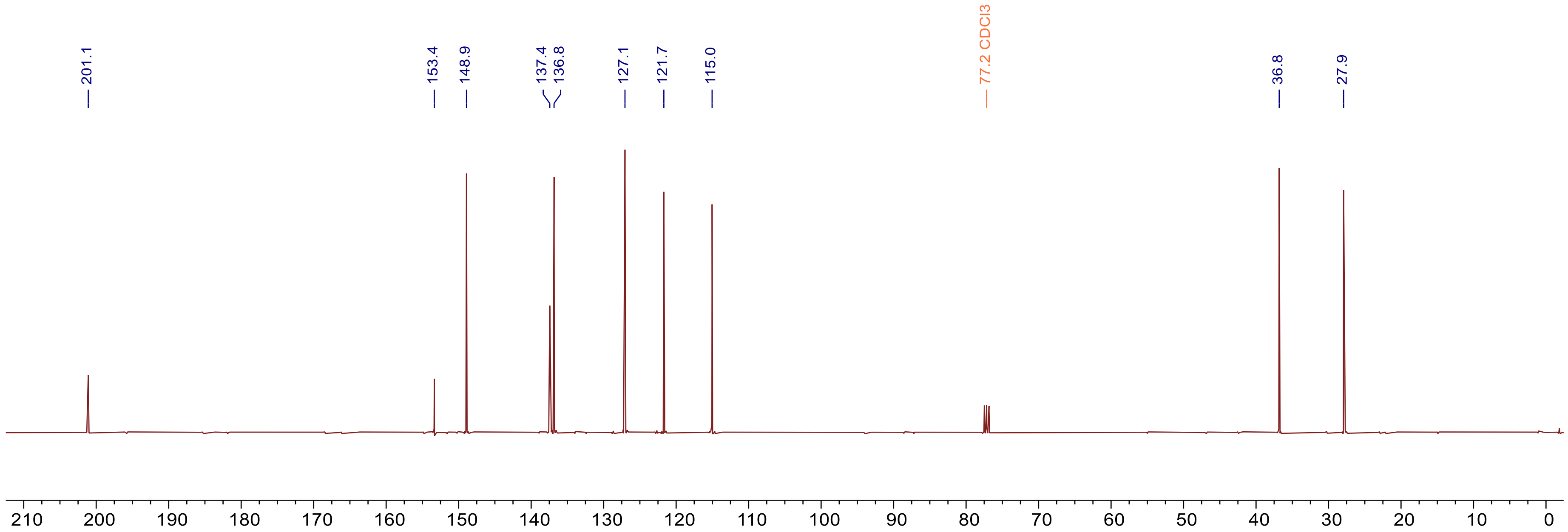

^{1}H NMR (400 MHz, $CDCl_3$):

1n

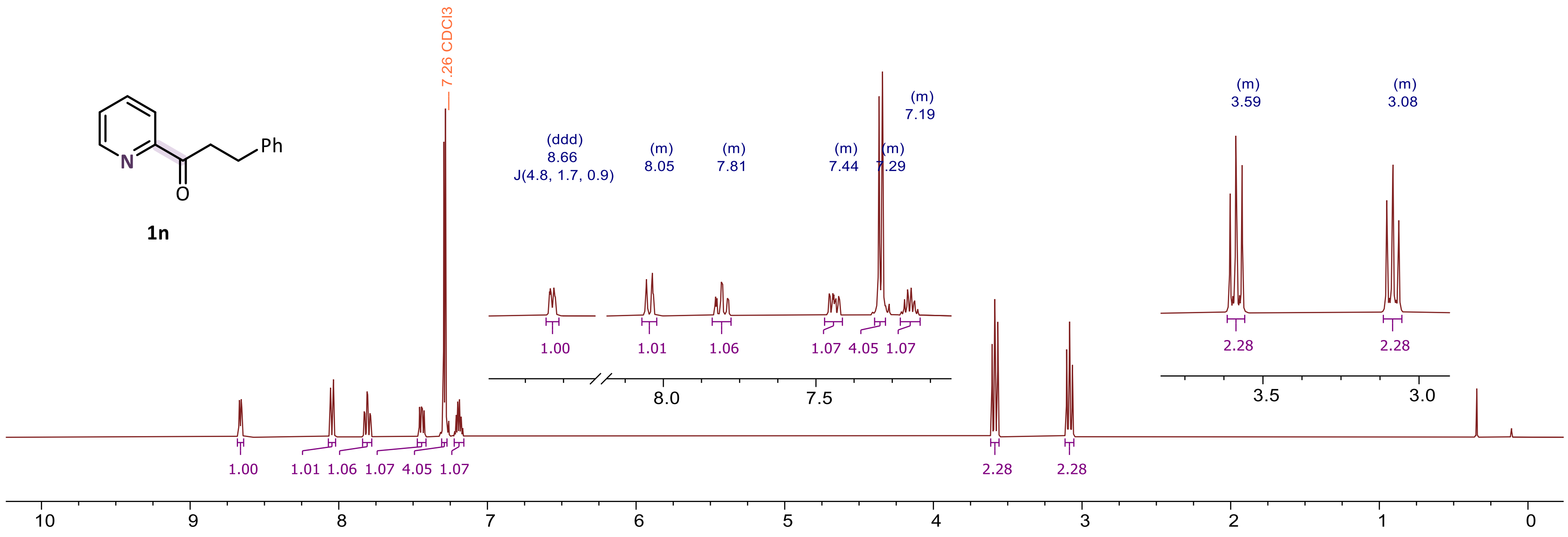

^{13}C NMR (100 MHz, $CDCl_3$):

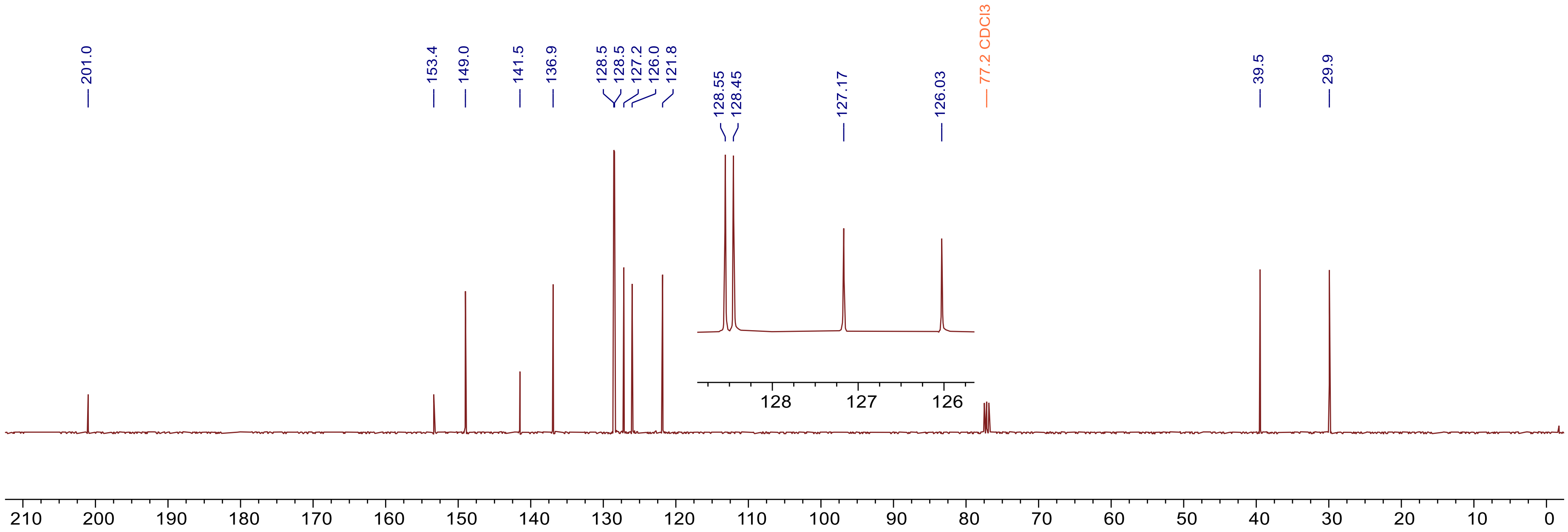

1o

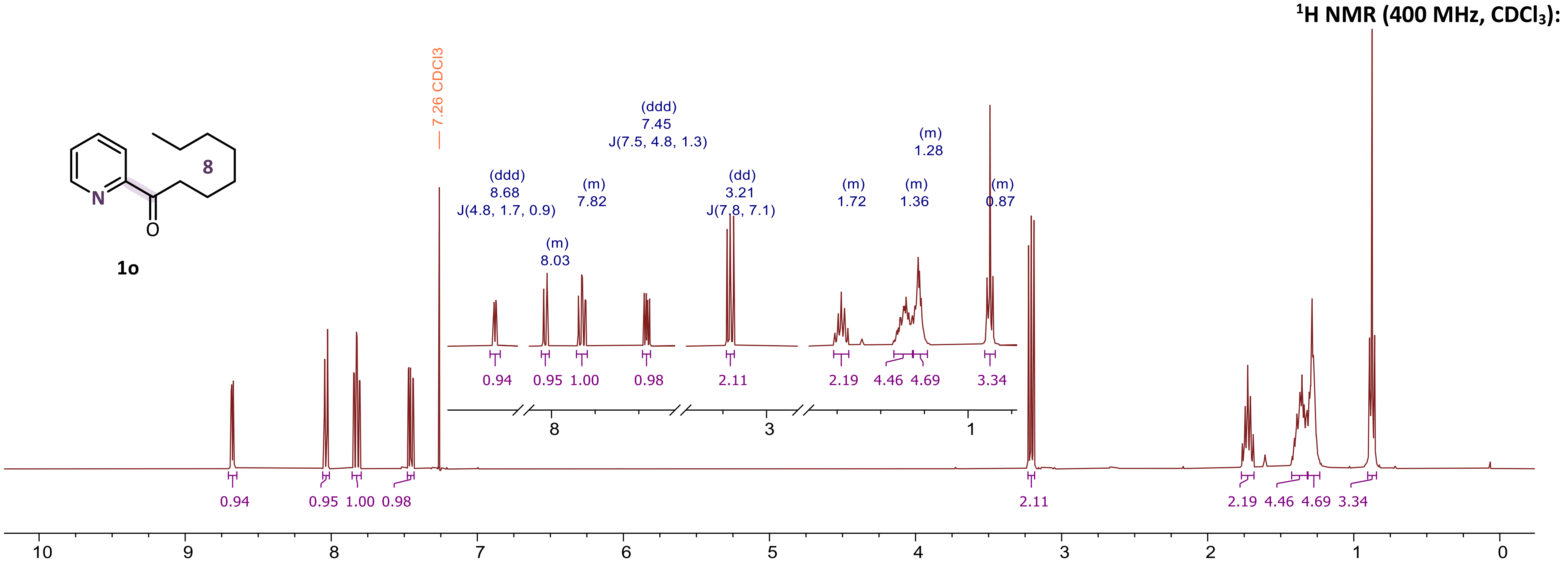
^{1}H NMR (400 MHz, CDCl$_3$):
7.26 CDCl3
(ddd) 8.68 J(4.8, 1.7, 0.9)
(m) 8.03
(m) 7.82
(ddd) 7.45 J(7.5, 4.8, 1.3)
(dd) 3.21 J(7.8, 7.1)
(m) 1.72
(m) 1.36
(m) 1.28
(m) 0.87
0.94 0.95 1.00 0.98 2.11 2.19 4.46 4.69 3.34

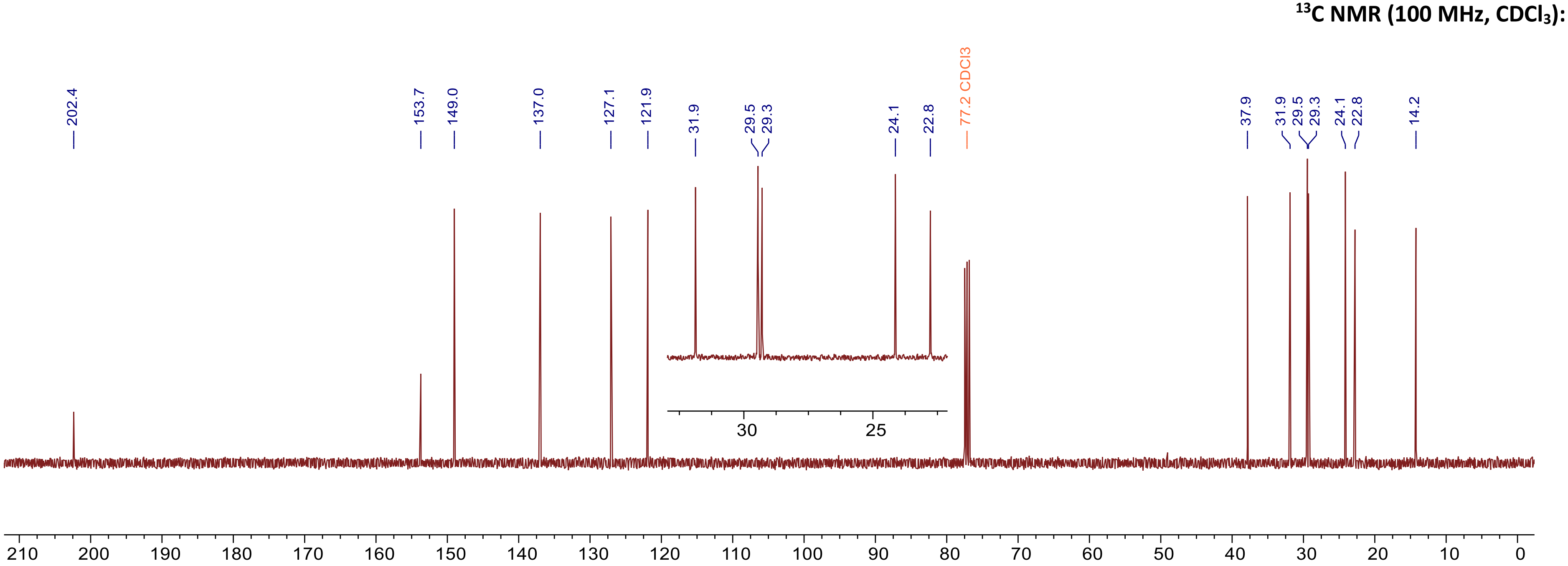
^{13}C NMR (100 MHz, CDCl$_3$):
202.4
153.7
149.0
137.0
127.1
121.9
77.2 CDCl3
37.9
31.9
29.5
29.3
24.1
22.8
14.2

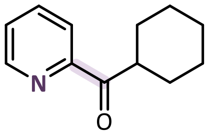

1p

^{1}H NMR (400 MHz, $CDCl_3$):

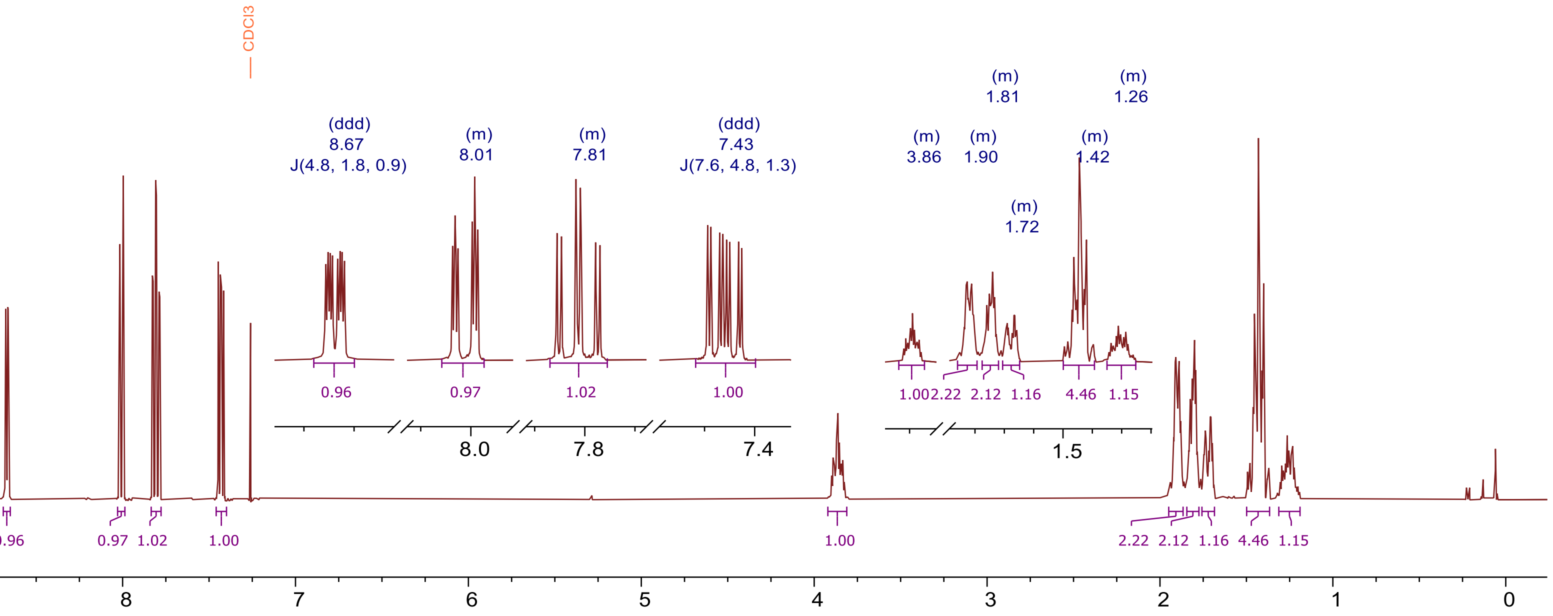

^{13}C NMR (100 MHz, $CDCl_3$):

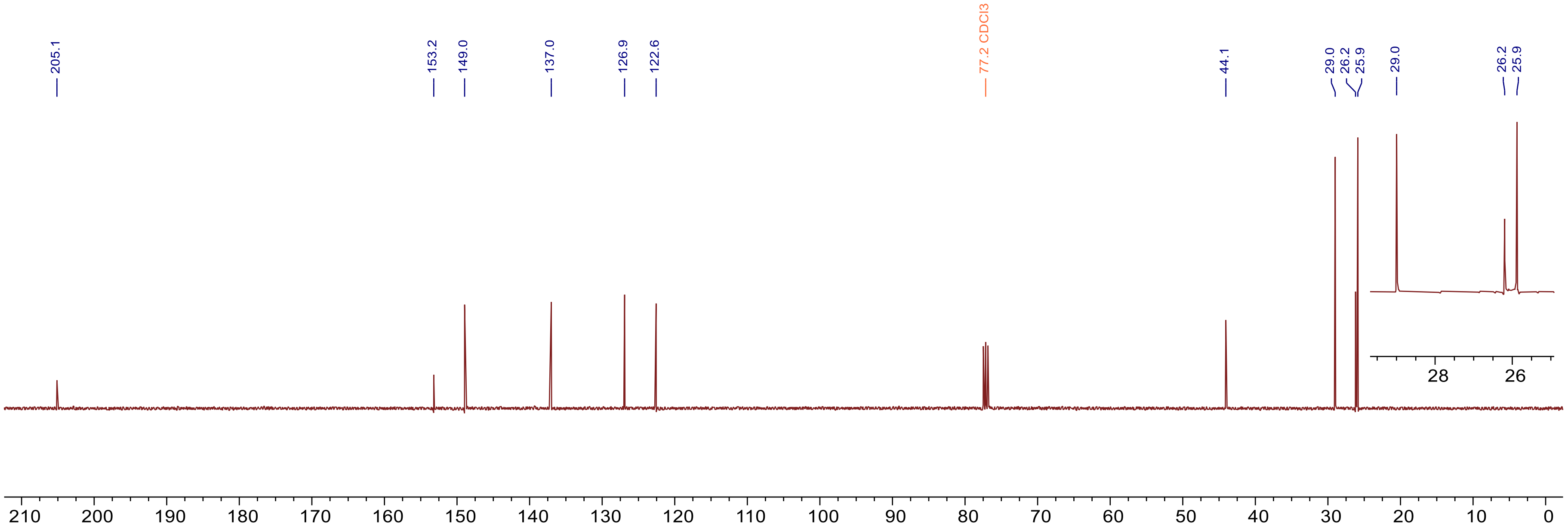

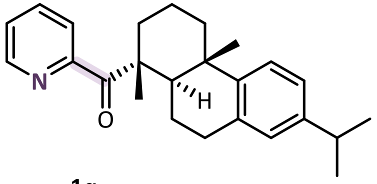

1q

^{1}H NMR (400 MHz, $CDCl_3$):

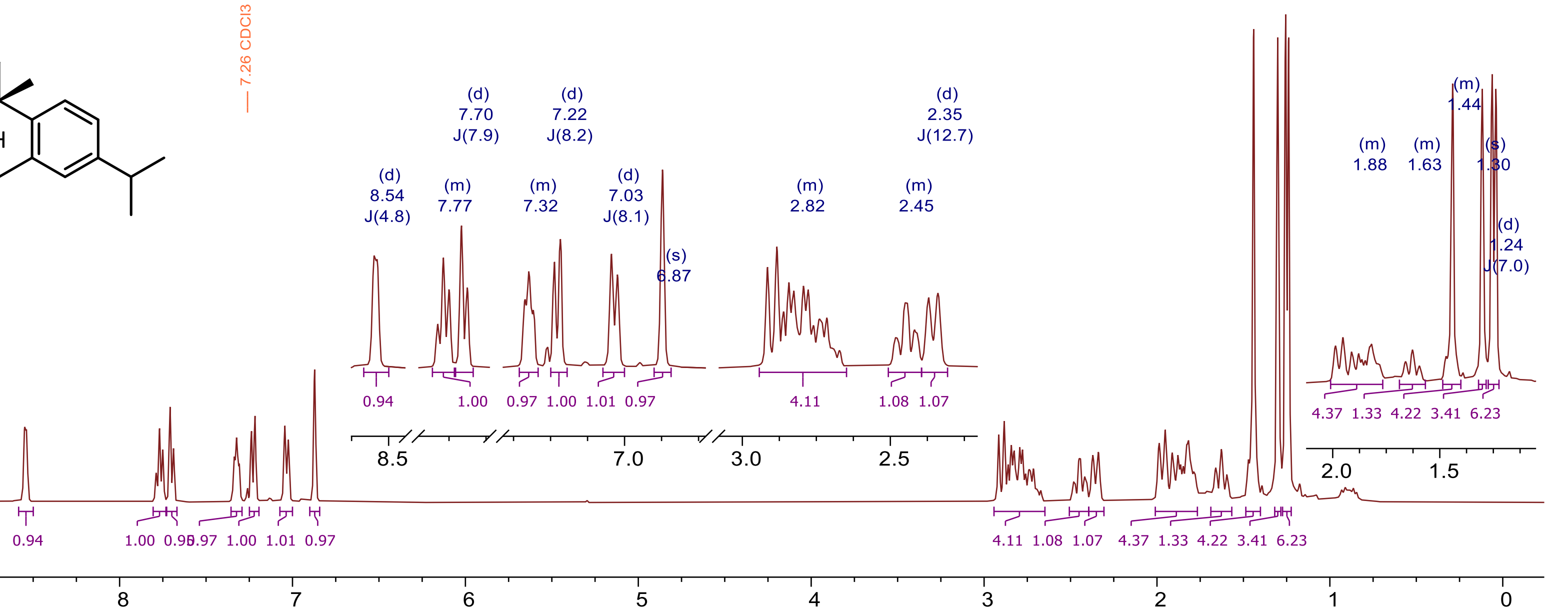

^{13}C NMR (100 MHz, $CDCl_3$):

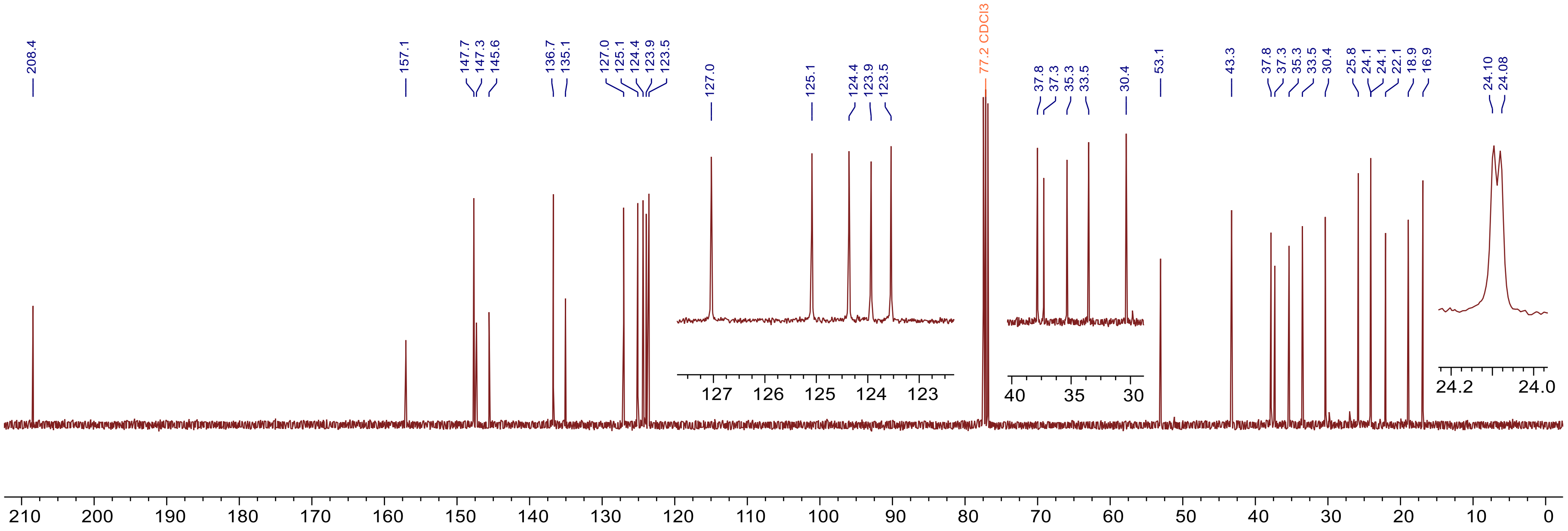

^{1}H NMR (400 MHz, $CDCl_3$):

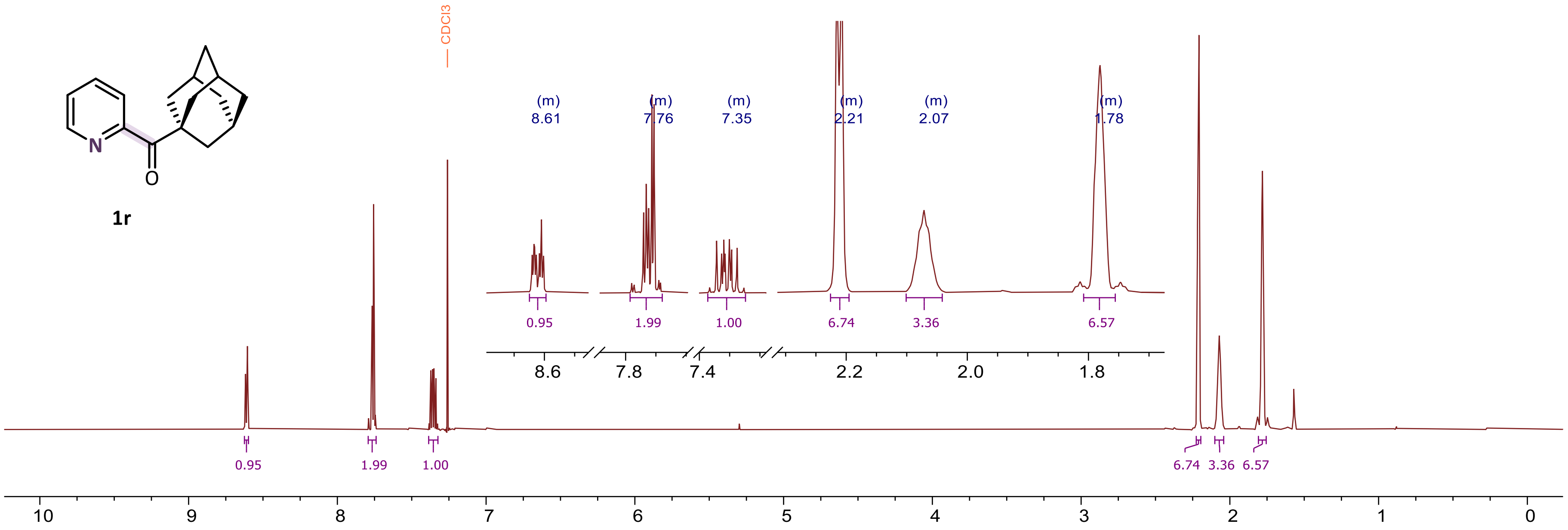

^{13}C NMR (100 MHz, $CDCl_3$):

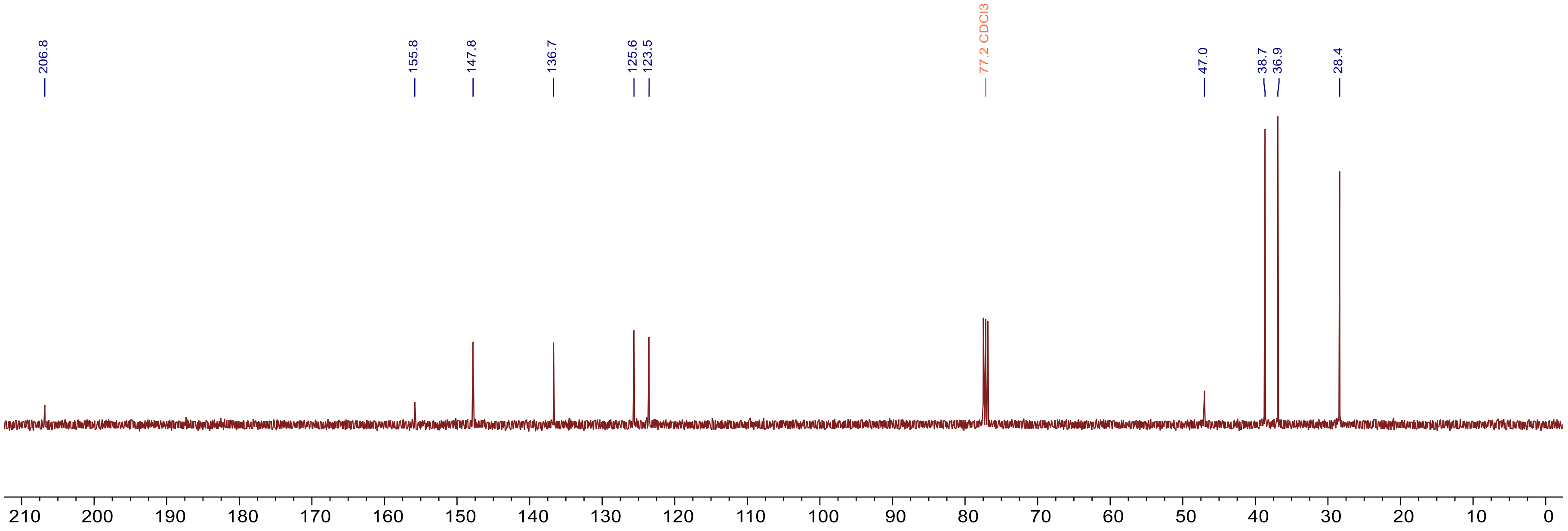

^{1}H NMR (400 MHz, $CDCl_3$):

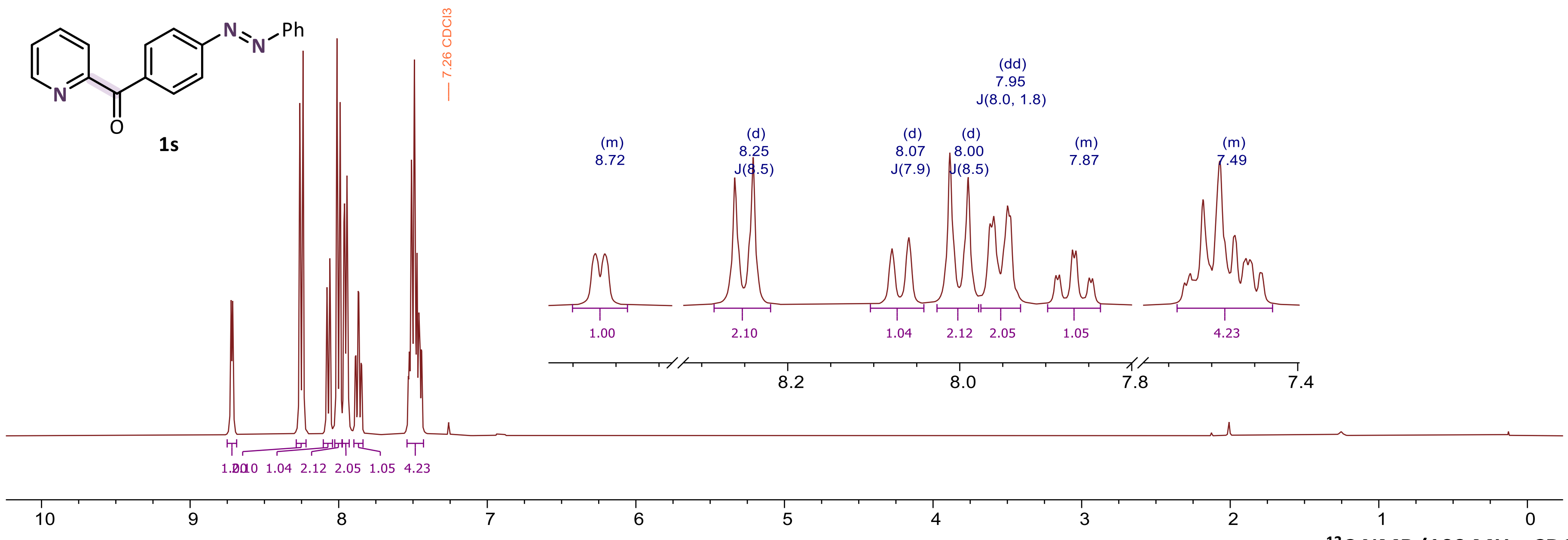

^{13}C NMR (100 MHz, $CDCl_3$):

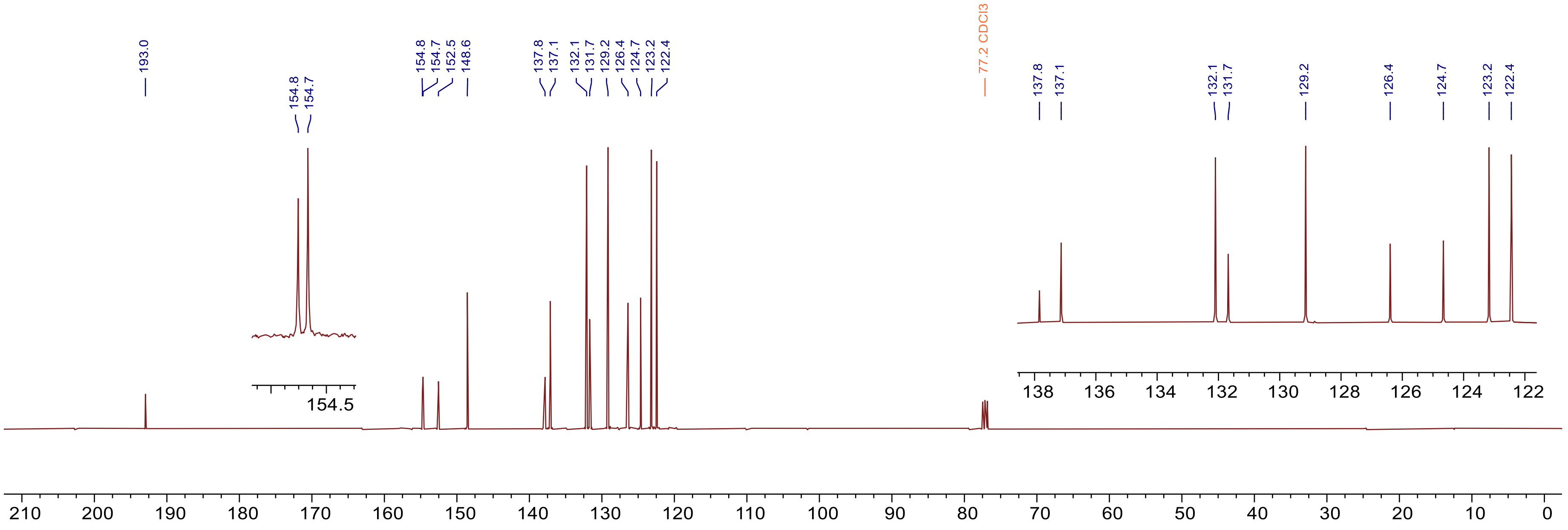

^{1}H NMR (400 MHz, $CDCl_3$):

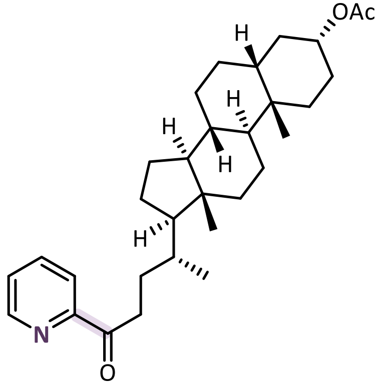

1t

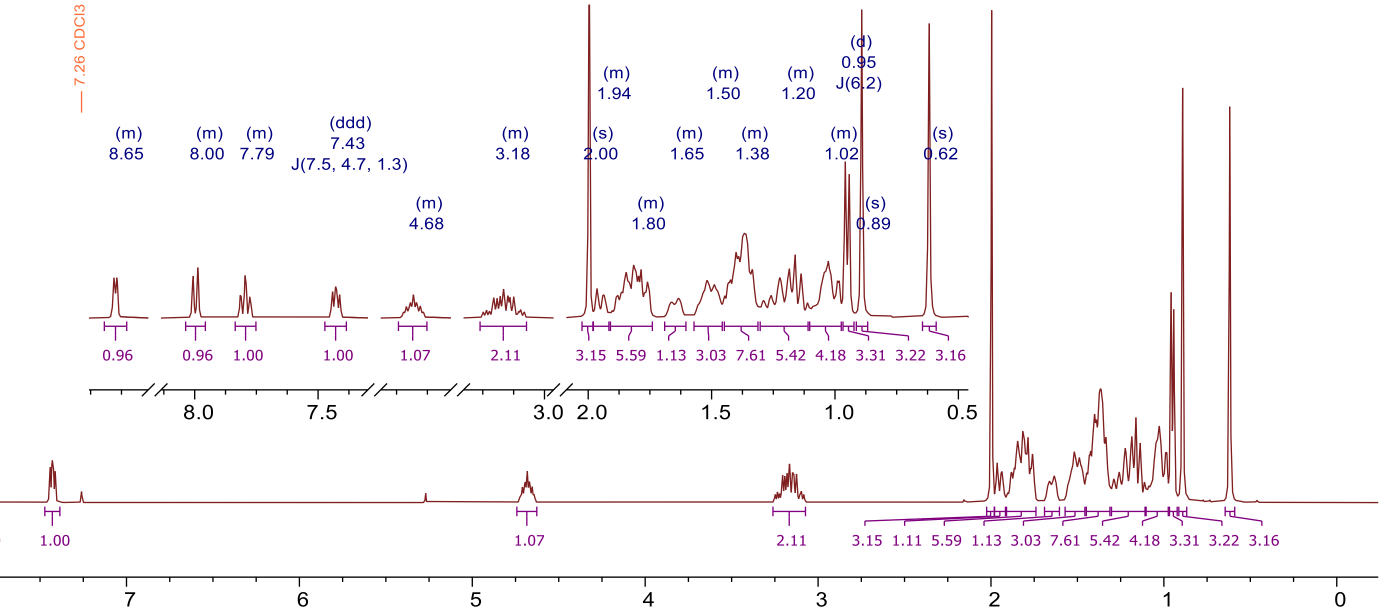

^{13}C NMR (100 MHz, $CDCl_3$):

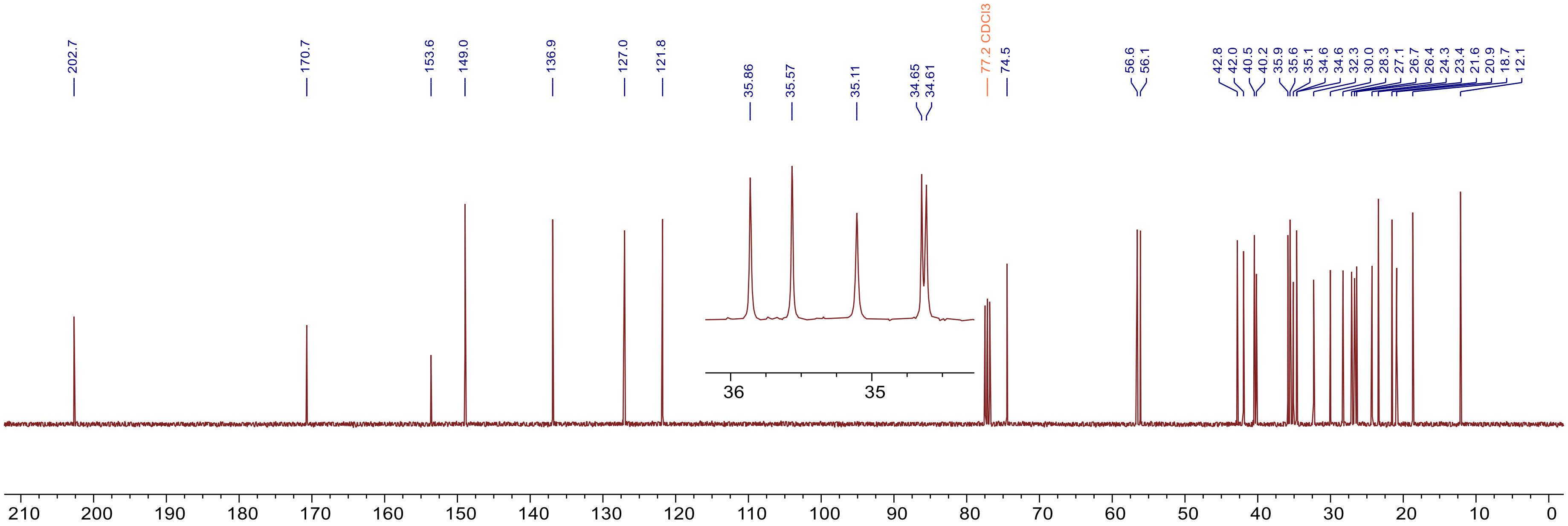

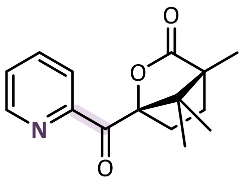

1u

^{1}H NMR (400 MHz, $CDCl_3$):

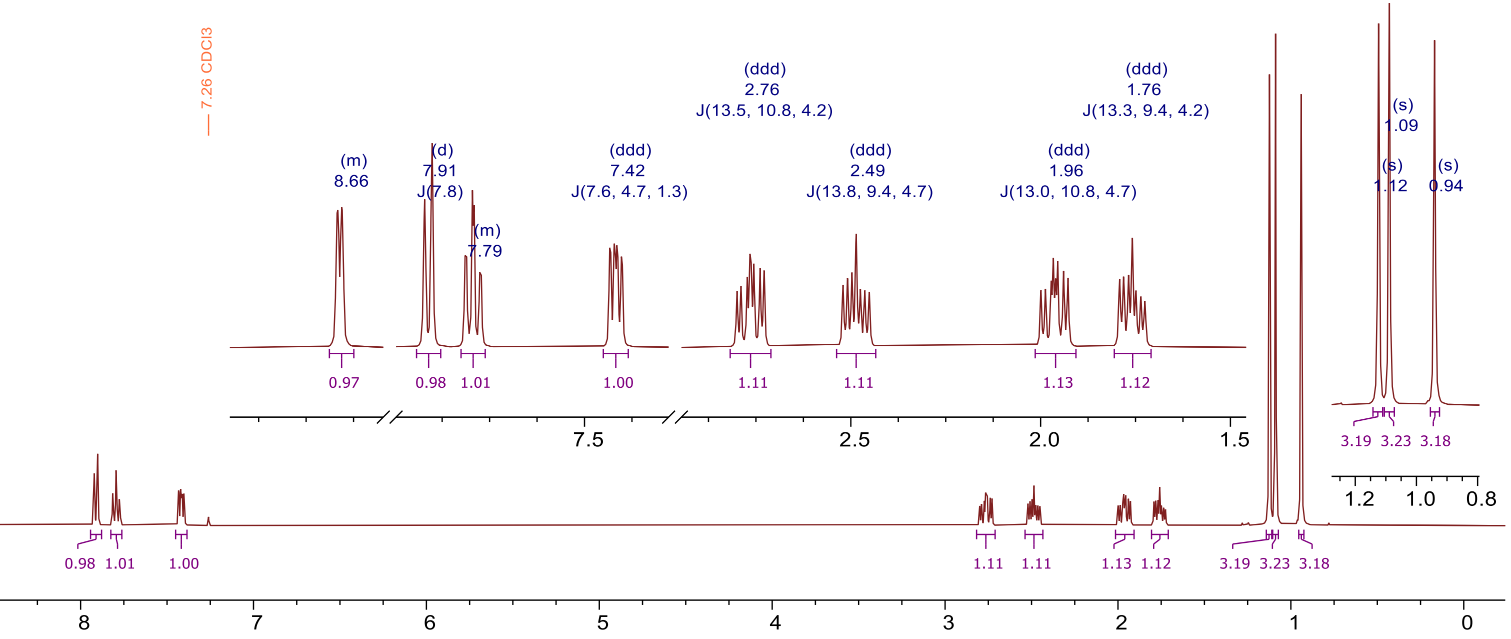

^{13}C NMR (100 MHz, $CDCl_3$):

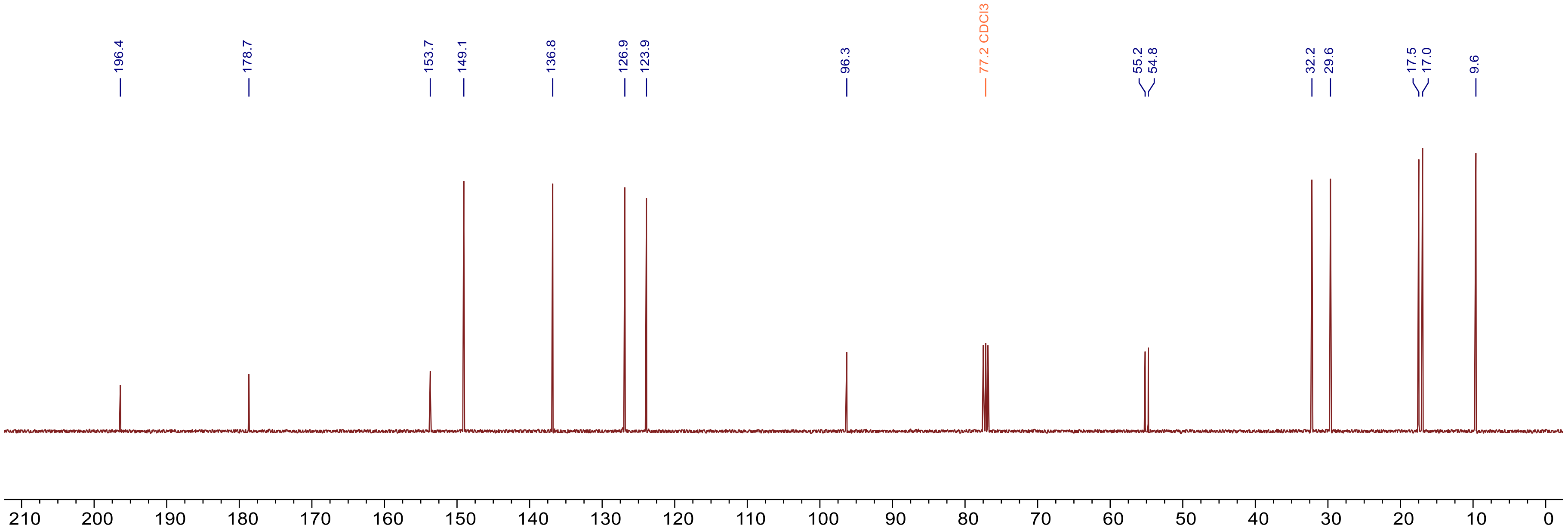

^{1}H NMR (400 MHz, CDCl$_3$):

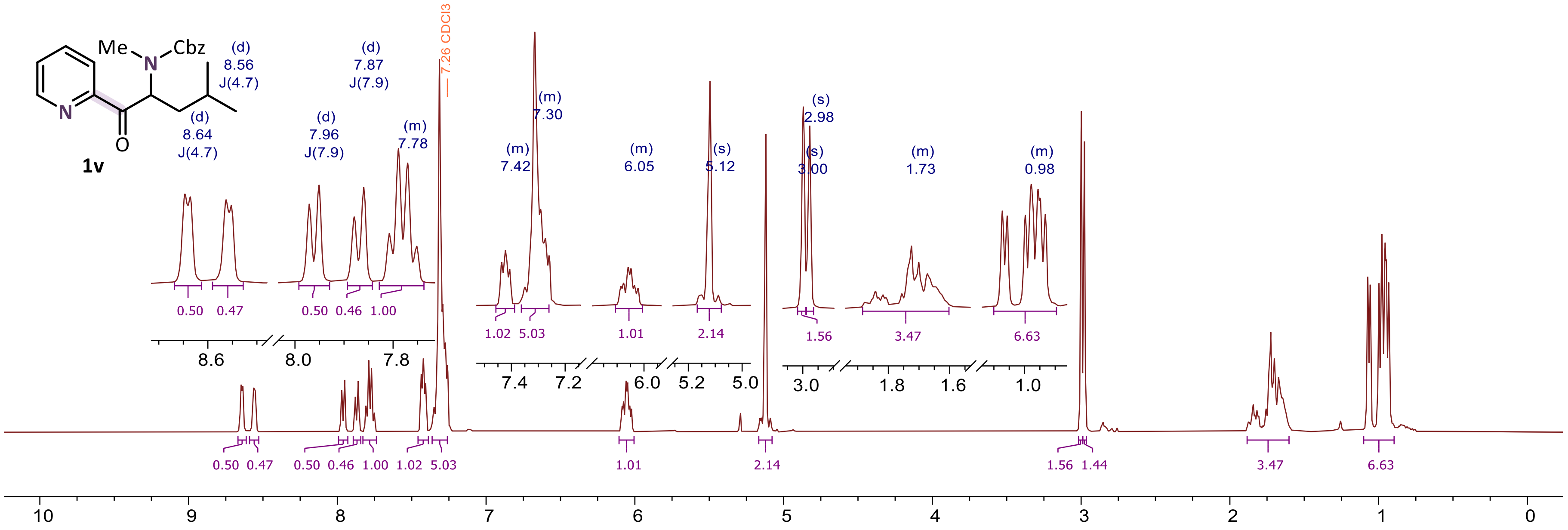

^{13}C NMR (100 MHz, CDCl$_3$):

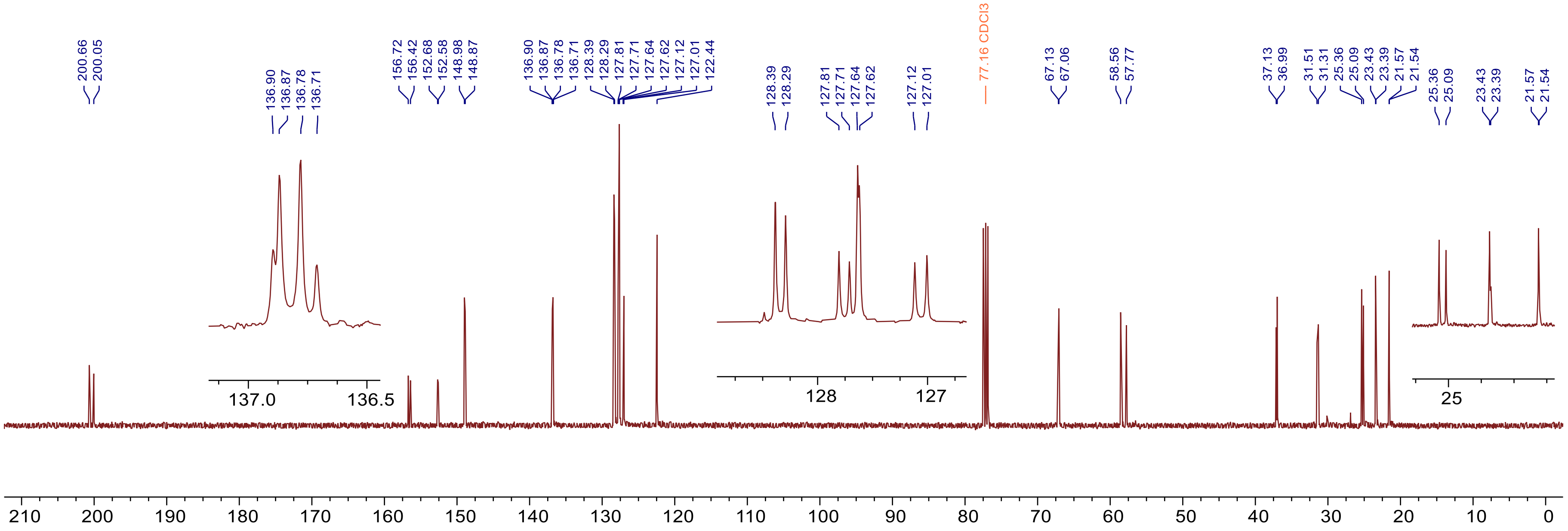

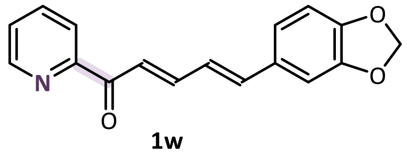

1w

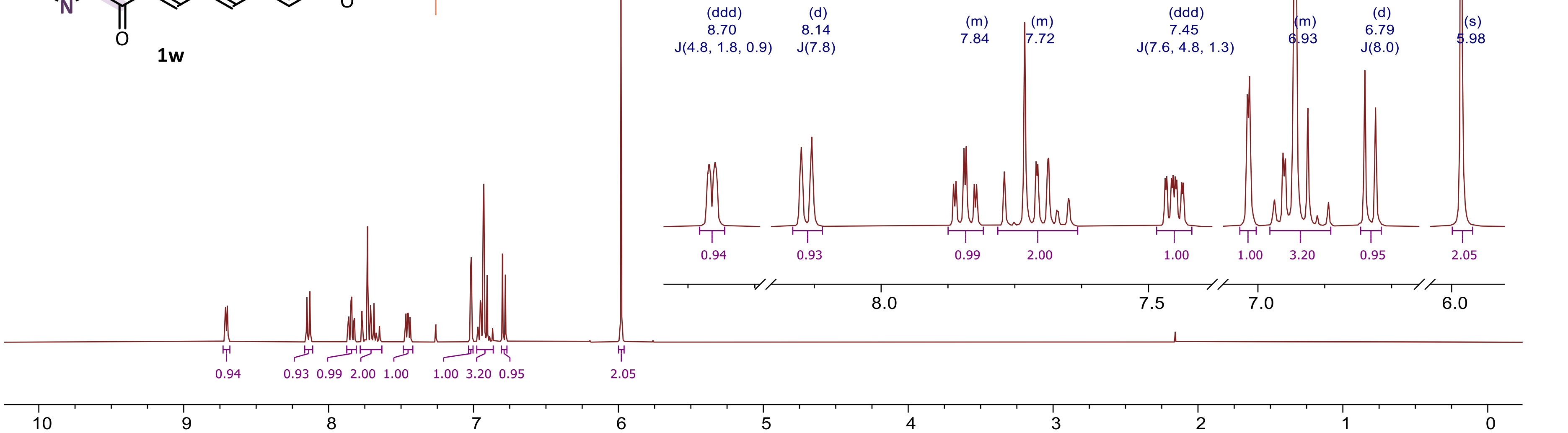

^{1}H NMR (400 MHz, CDCl$_3$):
7.26 CDCl3
(ddd) 8.70 J(4.8, 1.8, 0.9)
(d) 8.14 J(7.8)
(m) 7.84
(m) 7.72
(ddd) 7.45 J(7.6, 4.8, 1.3)
(d) 7.02 J(1.7)
(m) 6.93
(d) 6.79 J(8.0)
(s) 5.98
0.94 0.93 0.99 2.00 1.00 1.00 3.20 0.95 2.05

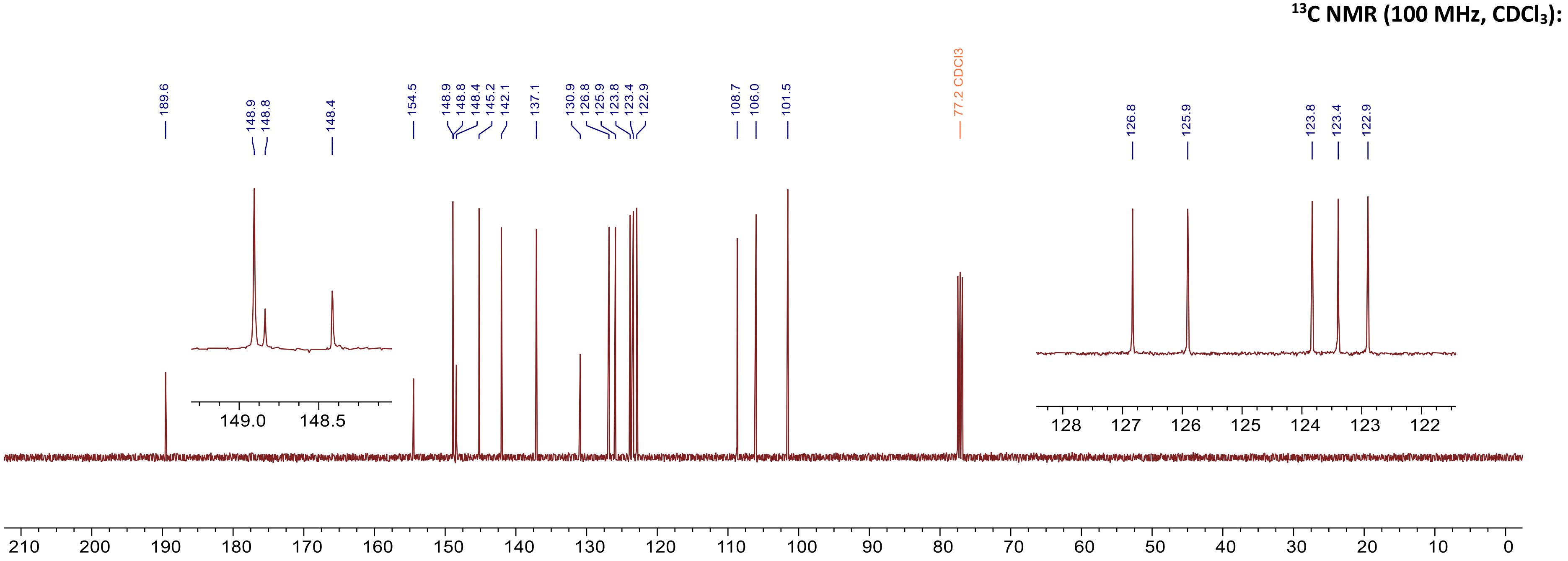

^{13}C NMR (100 MHz, CDCl$_3$):
189.6
148.9 148.8 148.4
154.5
148.9 148.8 148.4 145.2 142.1 137.1 130.9 126.8 125.9 123.8 123.4 122.9
108.7 106.0 101.5
77.2 CDCl3
126.8 125.9 123.8 123.4 122.9

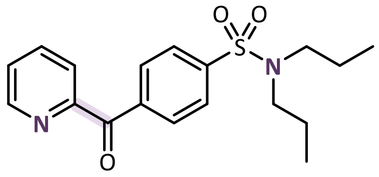

1x

^{1}H NMR (400 MHz, CDCl$_3$):

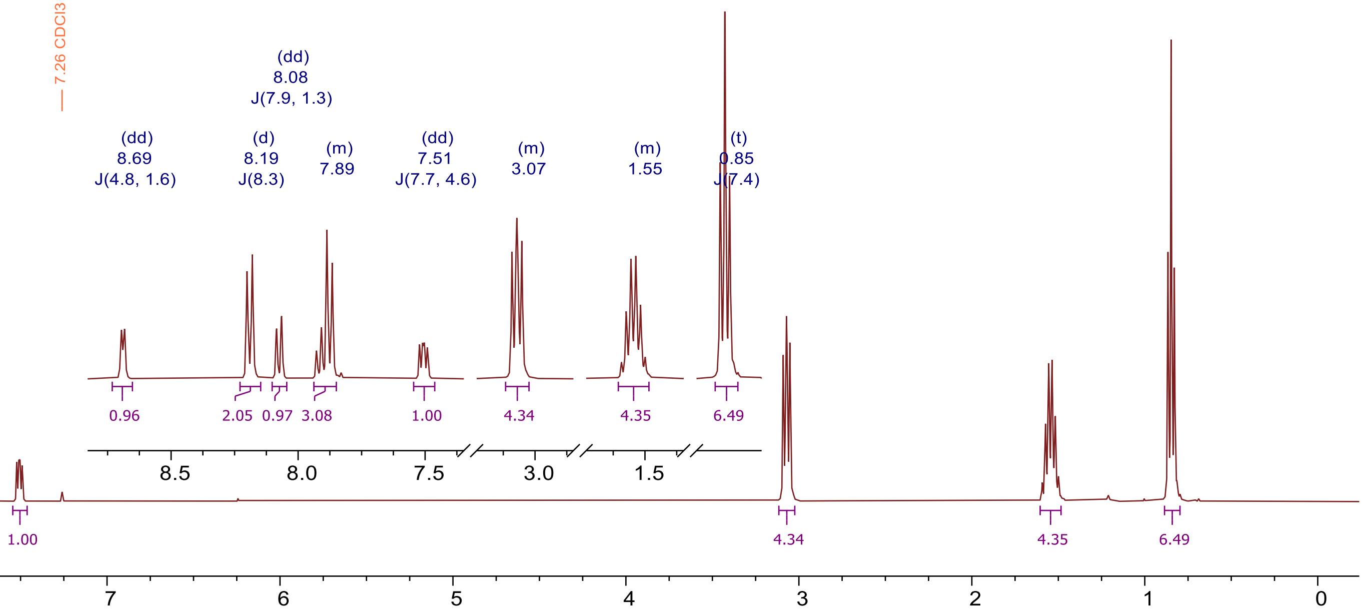

^{13}C NMR (100 MHz, CDCl$_3$):

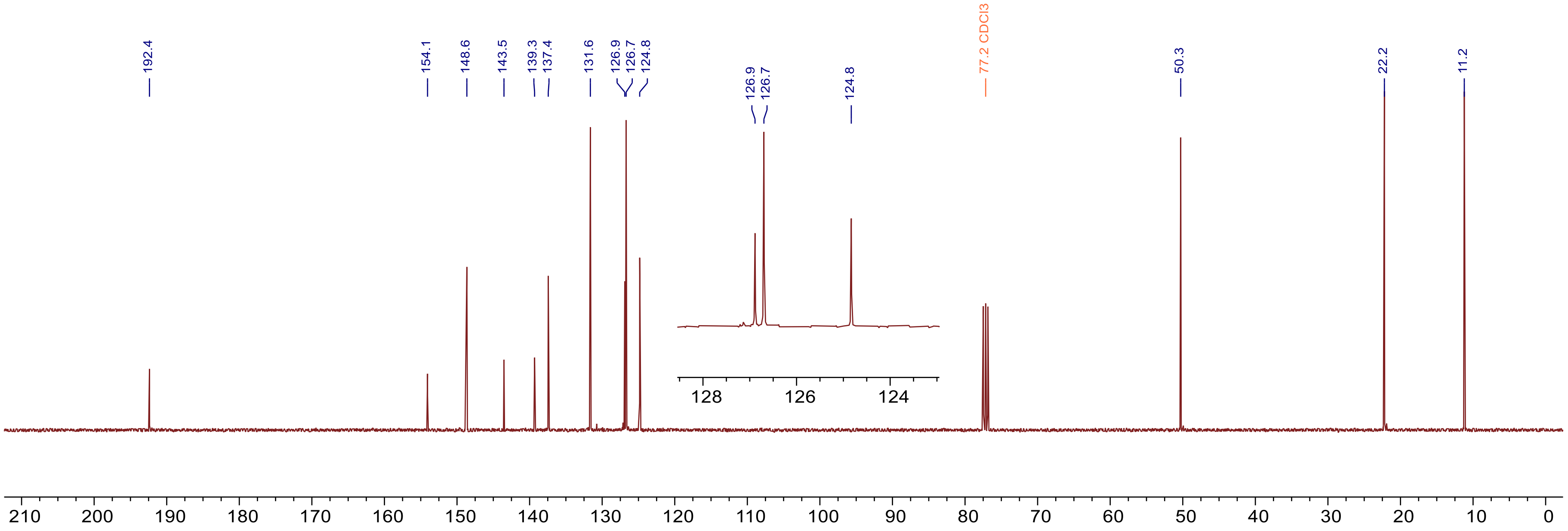

^{1}H NMR (400 MHz, $CDCl_3$):

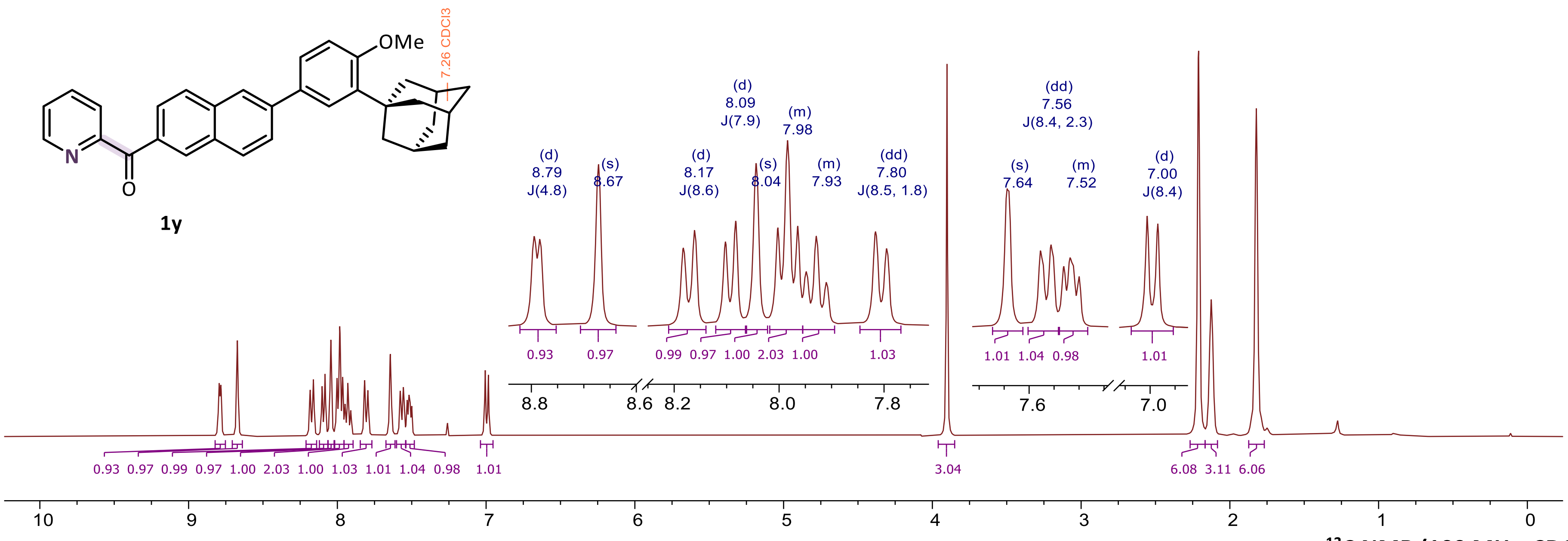

1y

^{13}C NMR (100 MHz, $CDCl_3$):

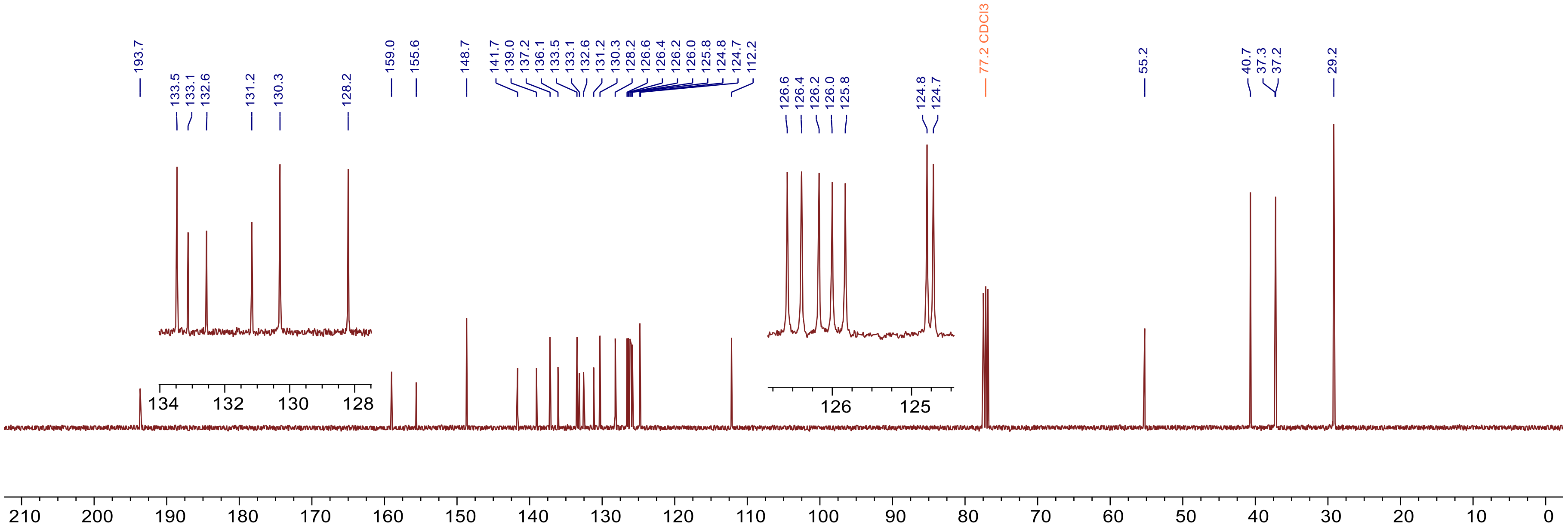

^{1}H NMR (400 MHz, $CDCl_3$):

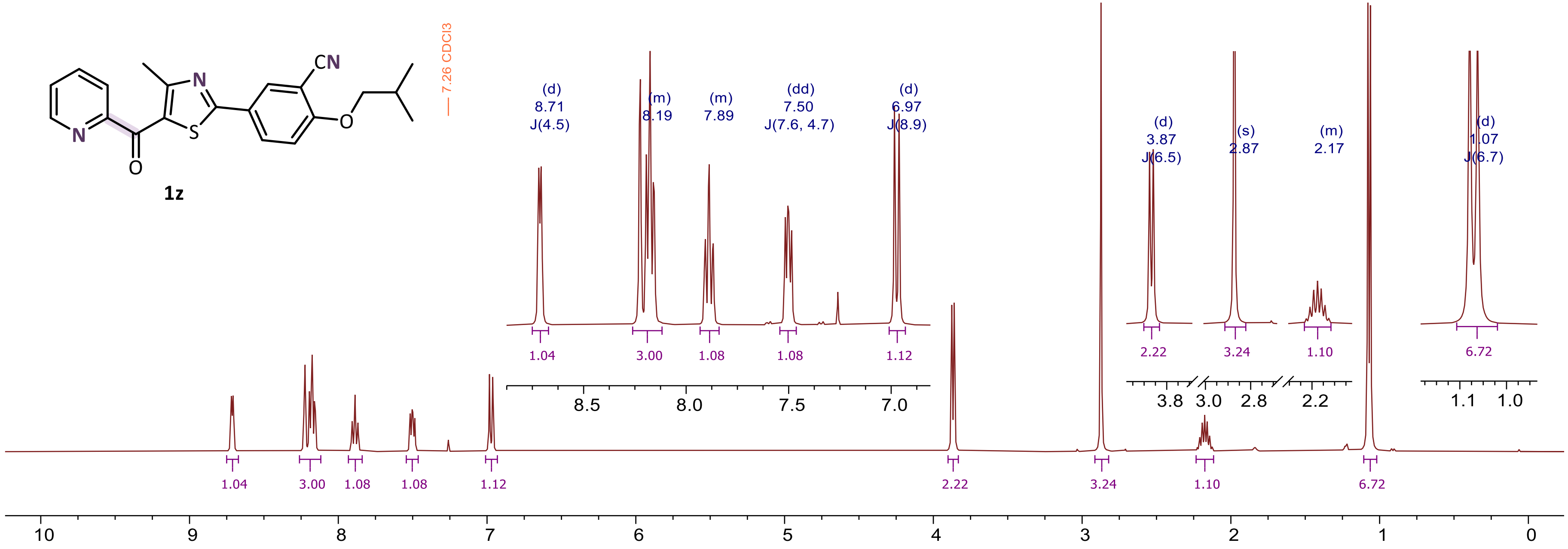

^{13}C NMR (100 MHz, $CDCl_3$):

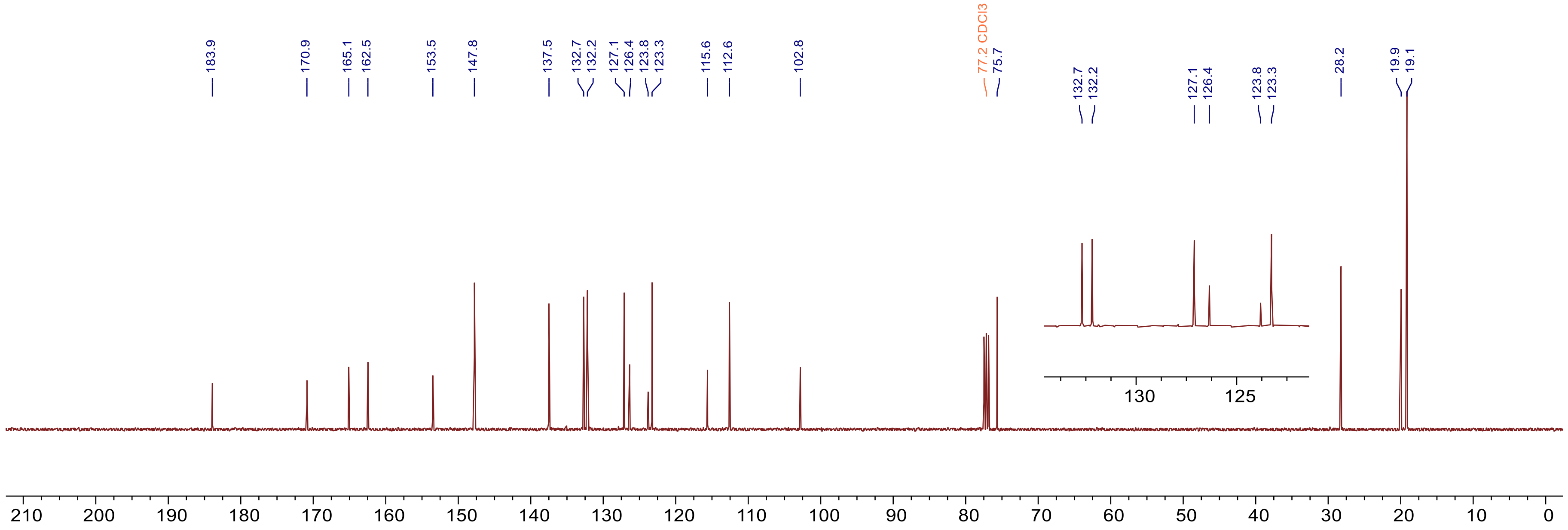

^{1}H NMR (400 MHz, $CDCl_3$):

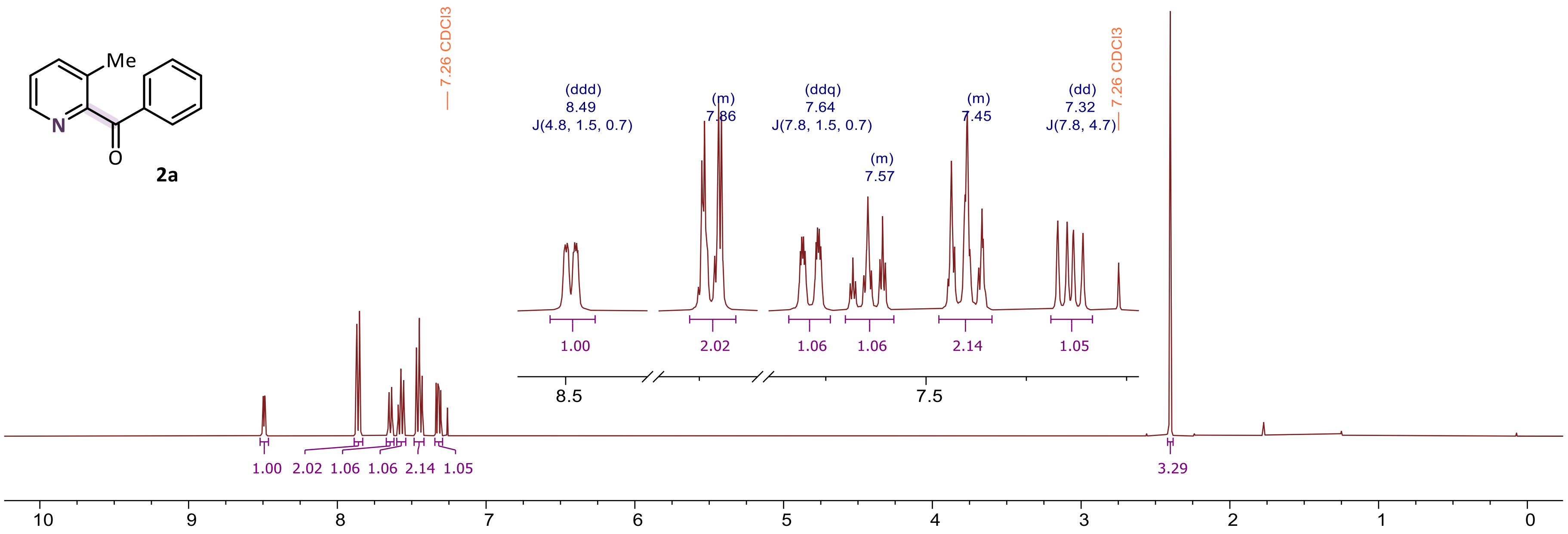

^{13}C NMR (100 MHz, $CDCl_3$):

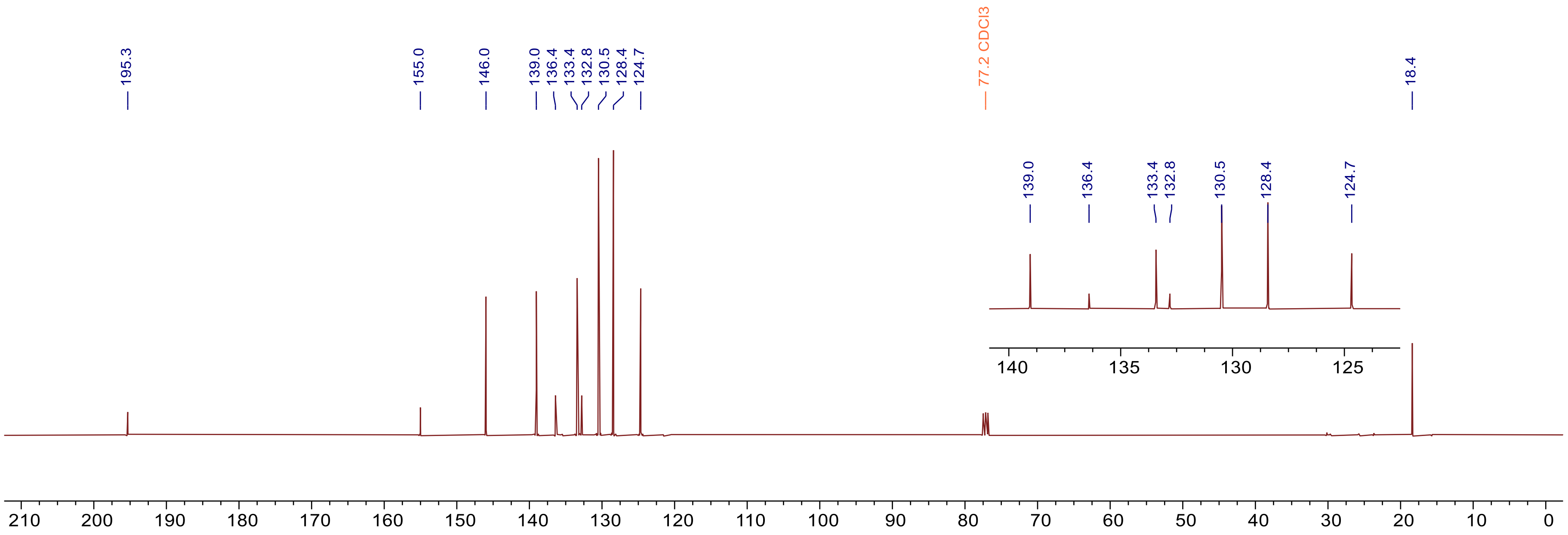

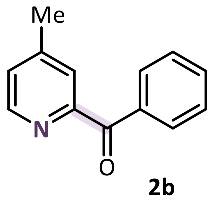

2b

^{1}H NMR (400 MHz, $CDCl_3$):

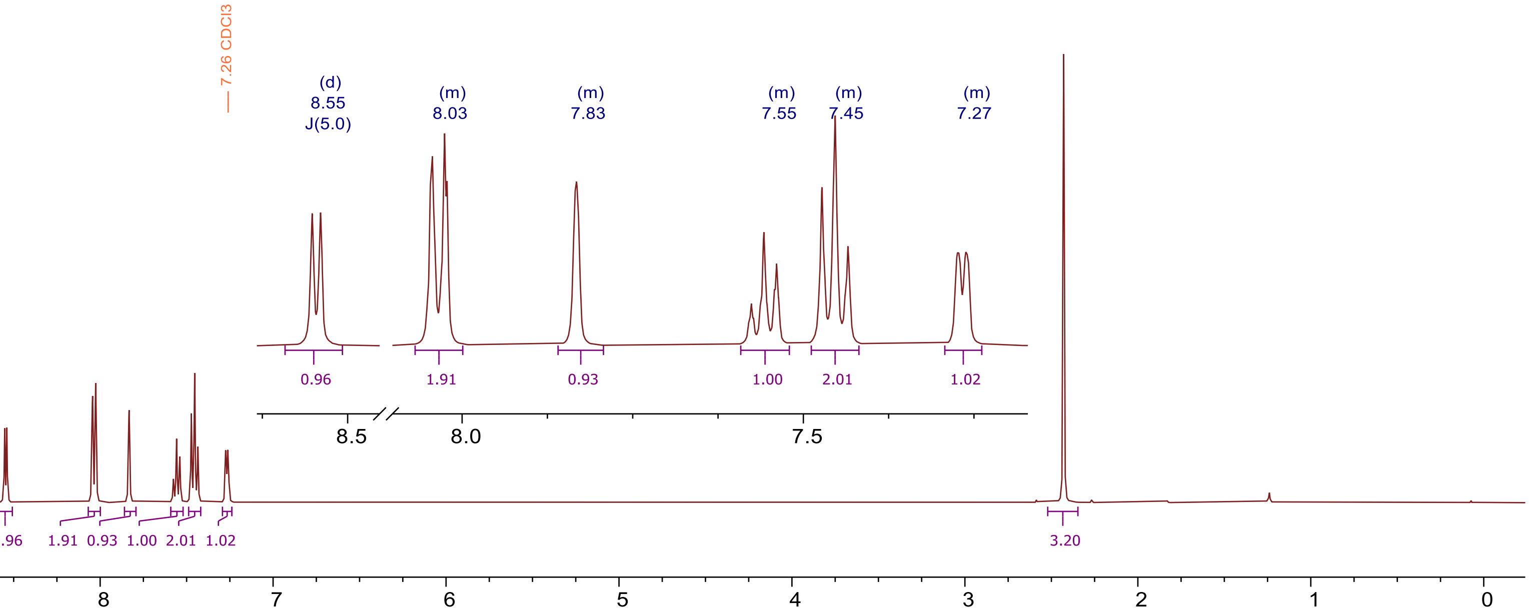

^{13}C NMR (100 MHz, $CDCl_3$):

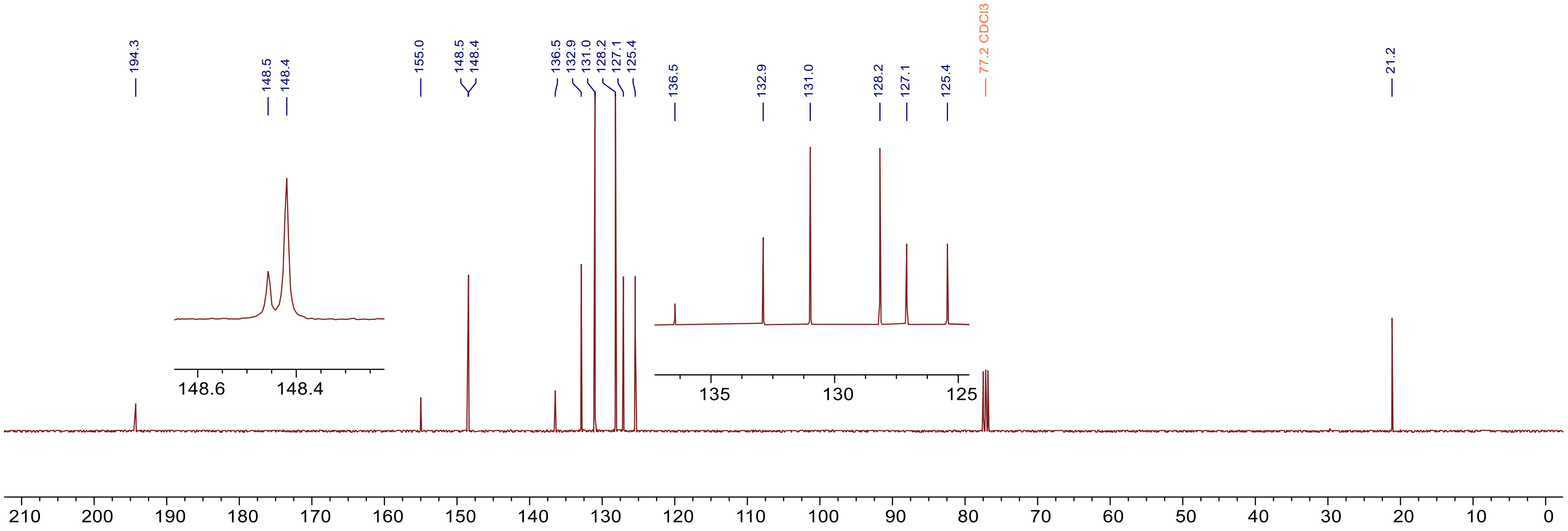

^{1}H NMR (400 MHz, $CDCl_3$):

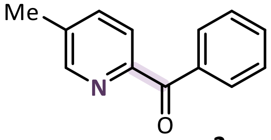

2c

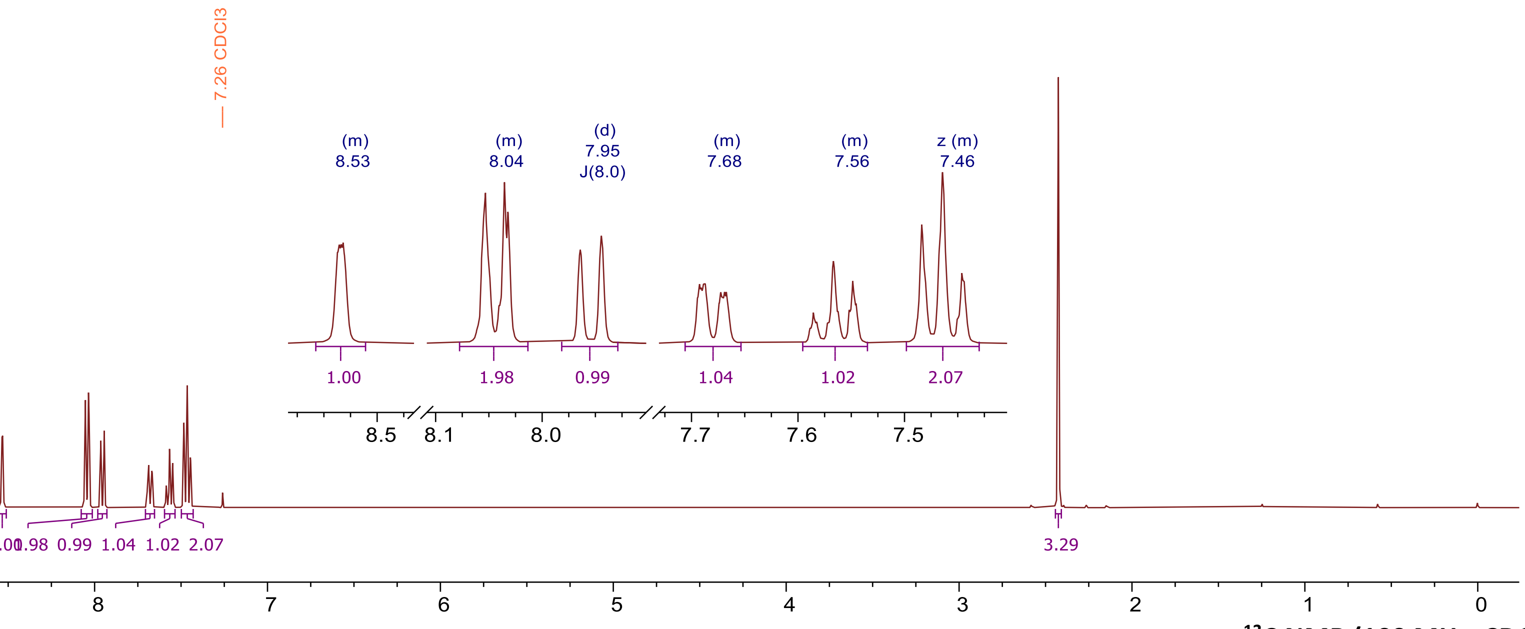

^{13}C NMR (100 MHz, $CDCl_3$):

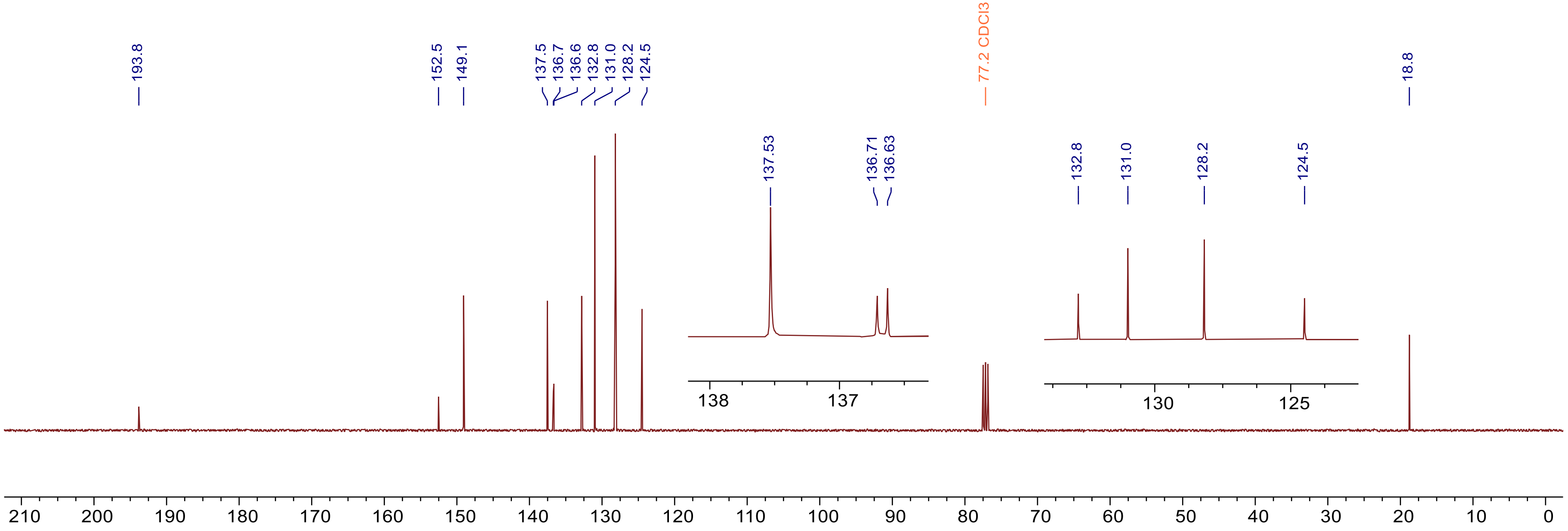

^{1}H NMR (400 MHz, $CDCl_3$):

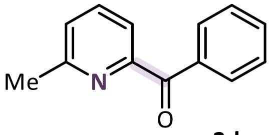

2d

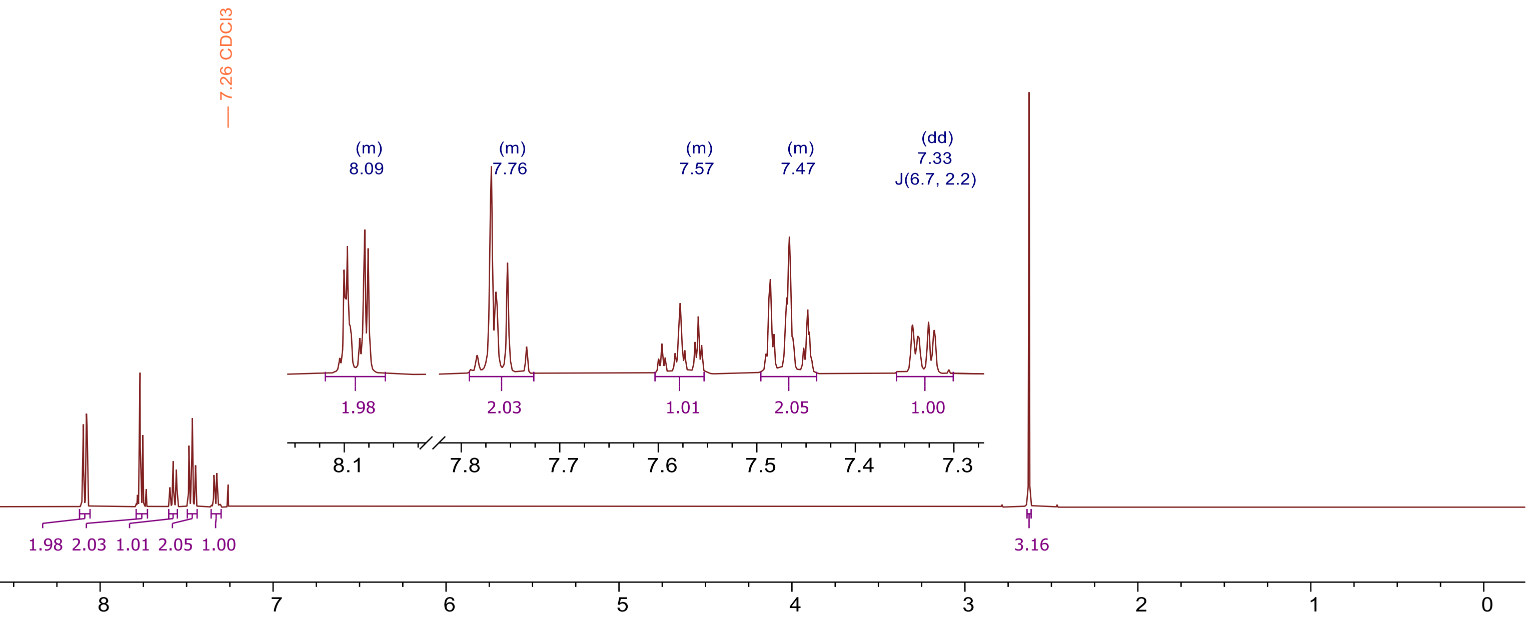

^{13}C NMR (100 MHz, $CDCl_3$):

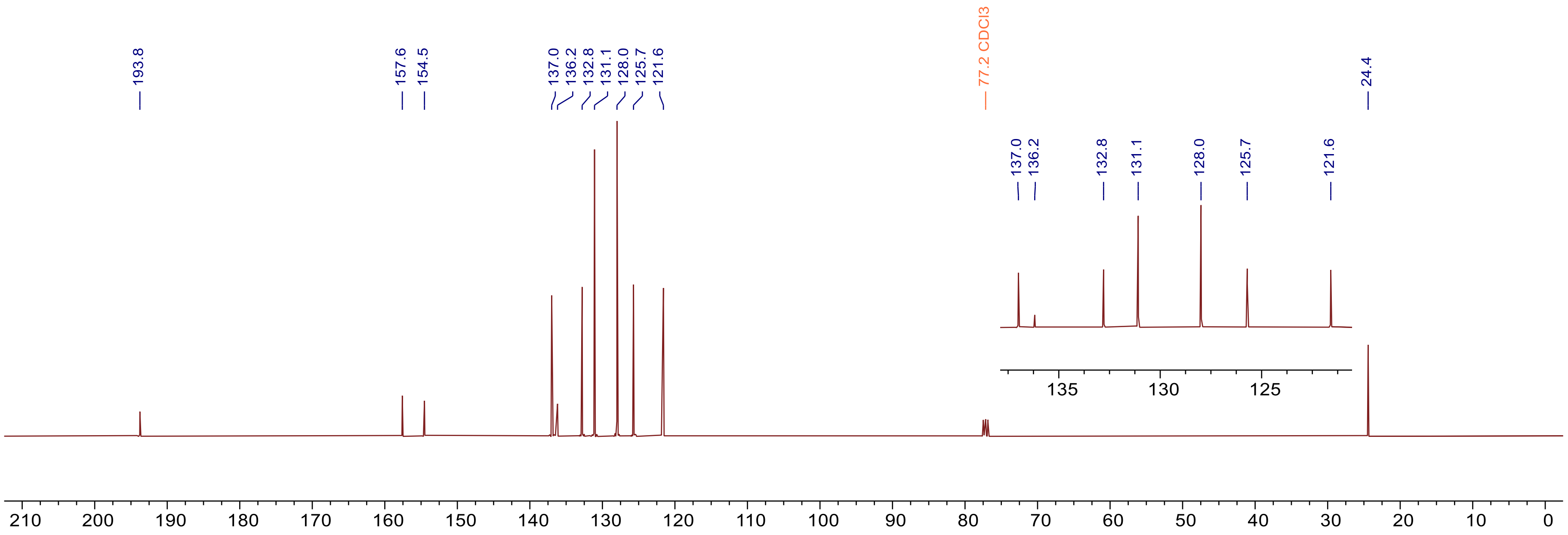

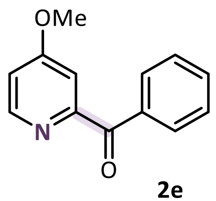

^{1}H NMR (400 MHz, $CDCl_3$):

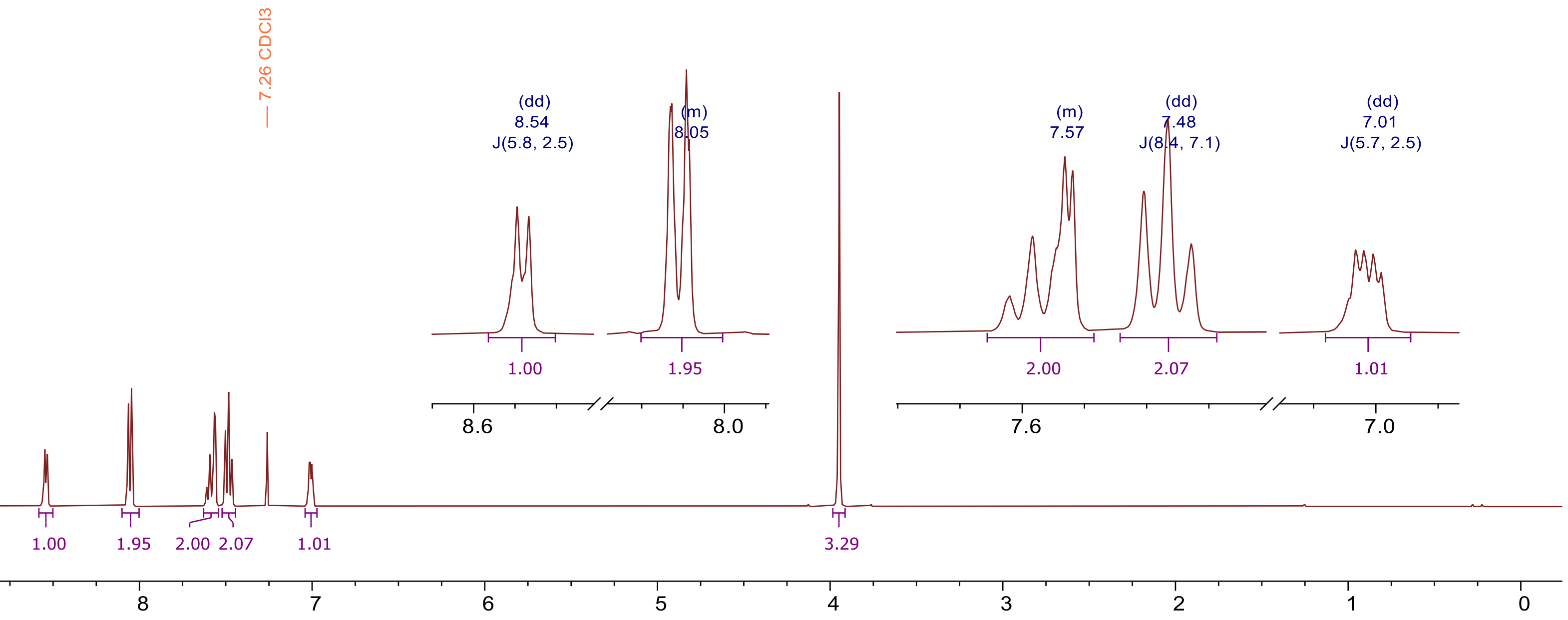

^{13}C NMR (100 MHz, $CDCl_3$):

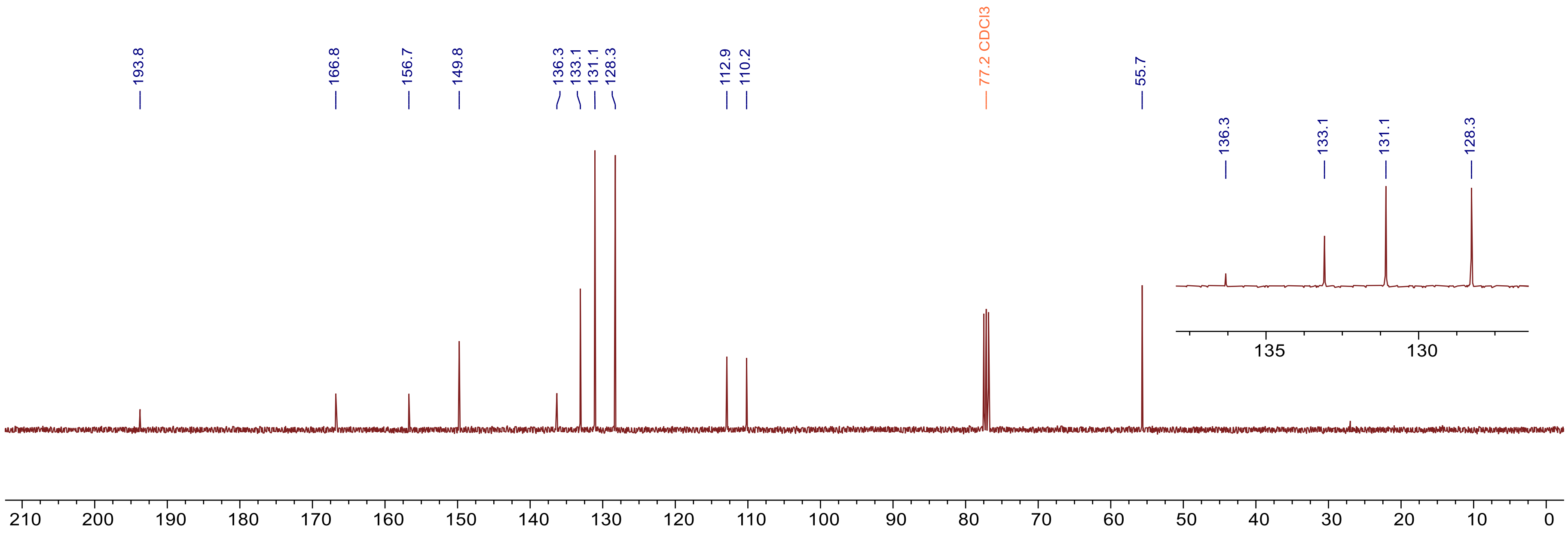

^{1}H NMR (400 MHz, $CDCl_3$):

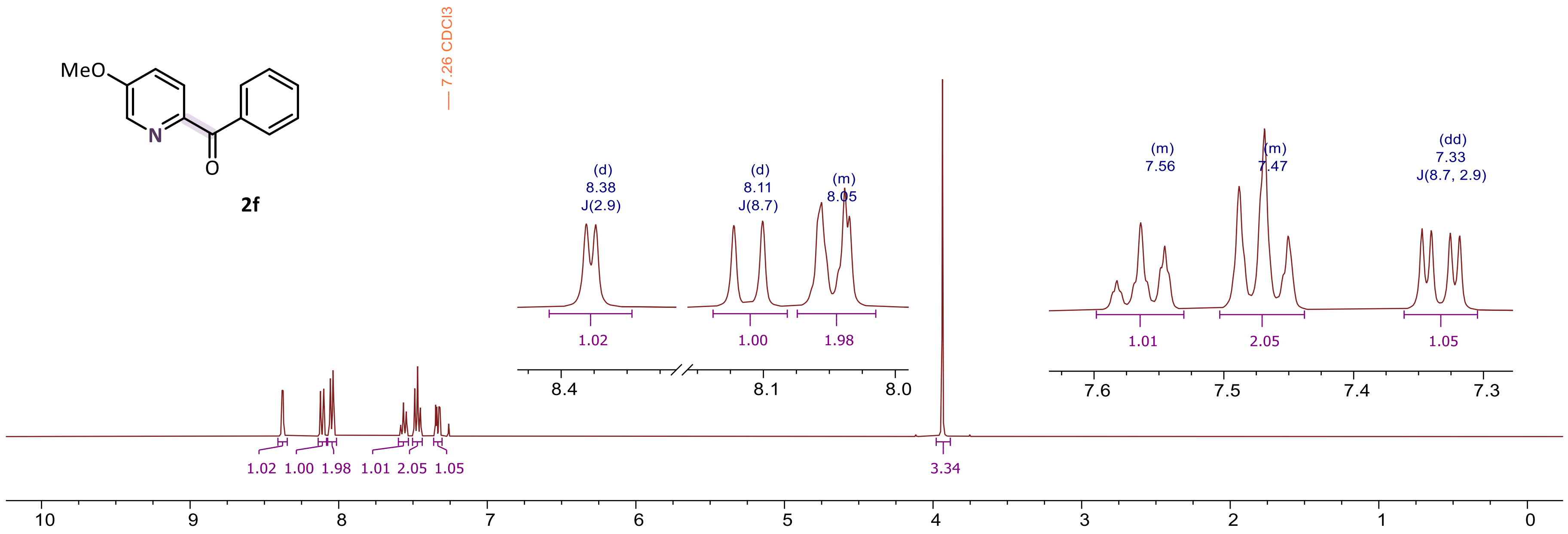

^{13}C NMR (100 MHz, $CDCl_3$):

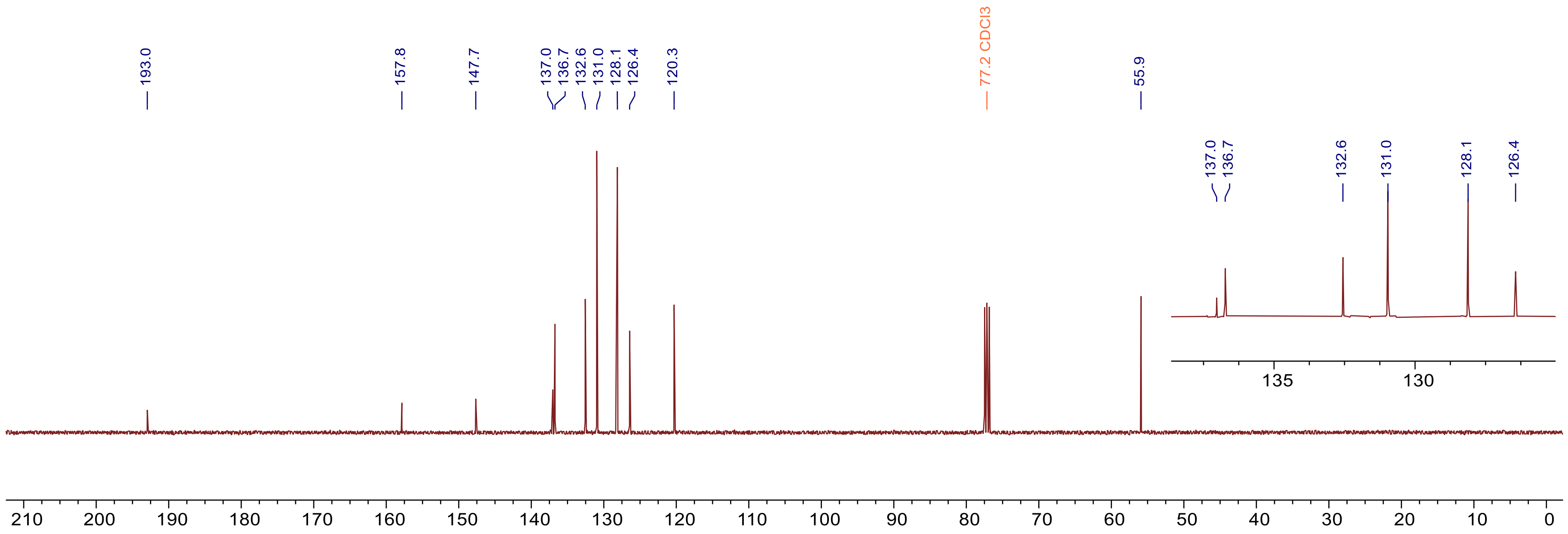

^{1}H NMR (400 MHz, CDCl$_3$):

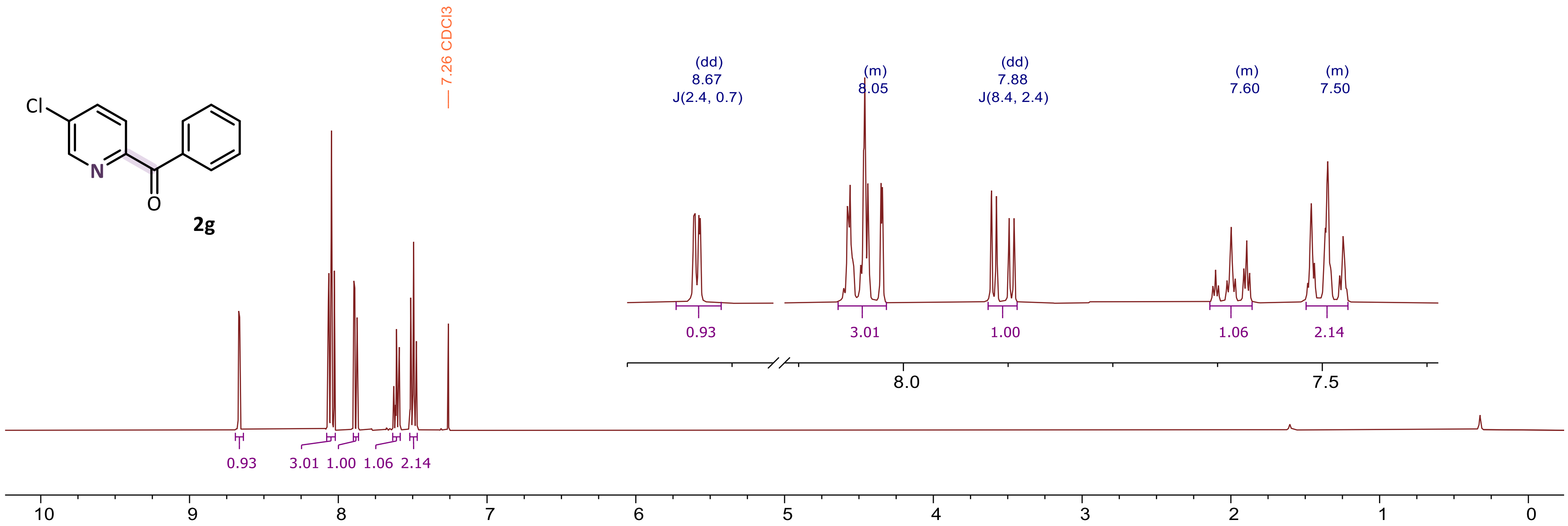

^{13}C NMR (100 MHz, CDCl$_3$):

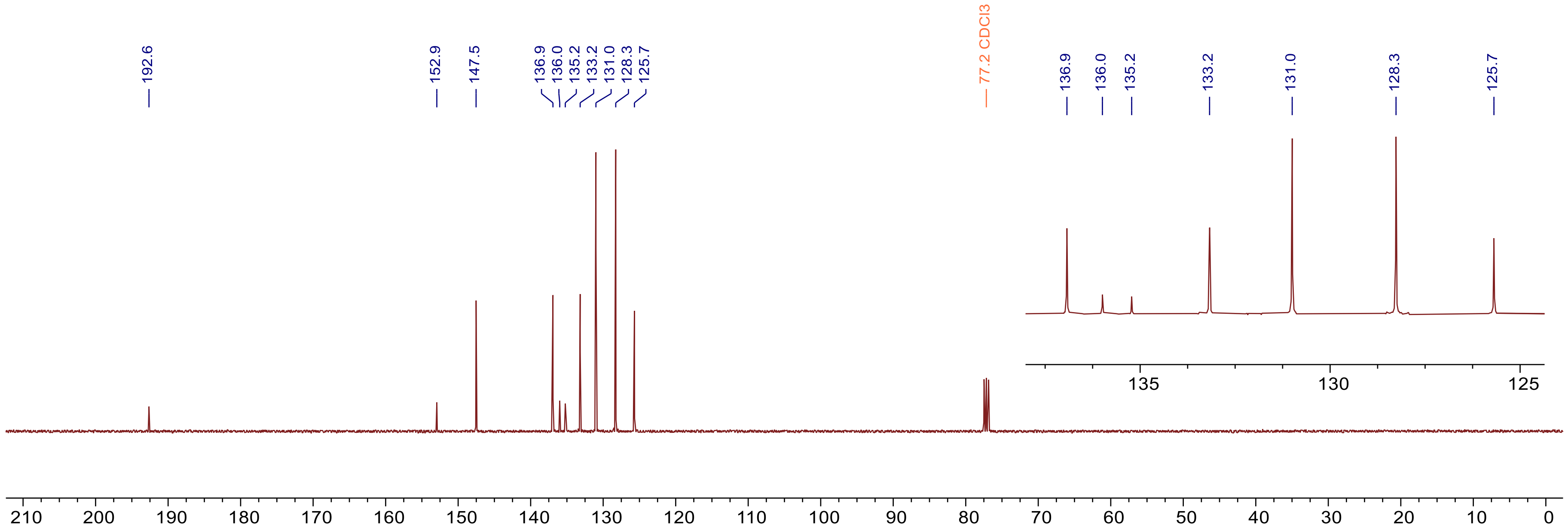

^{1}H NMR (400 MHz, $CDCl_3$):

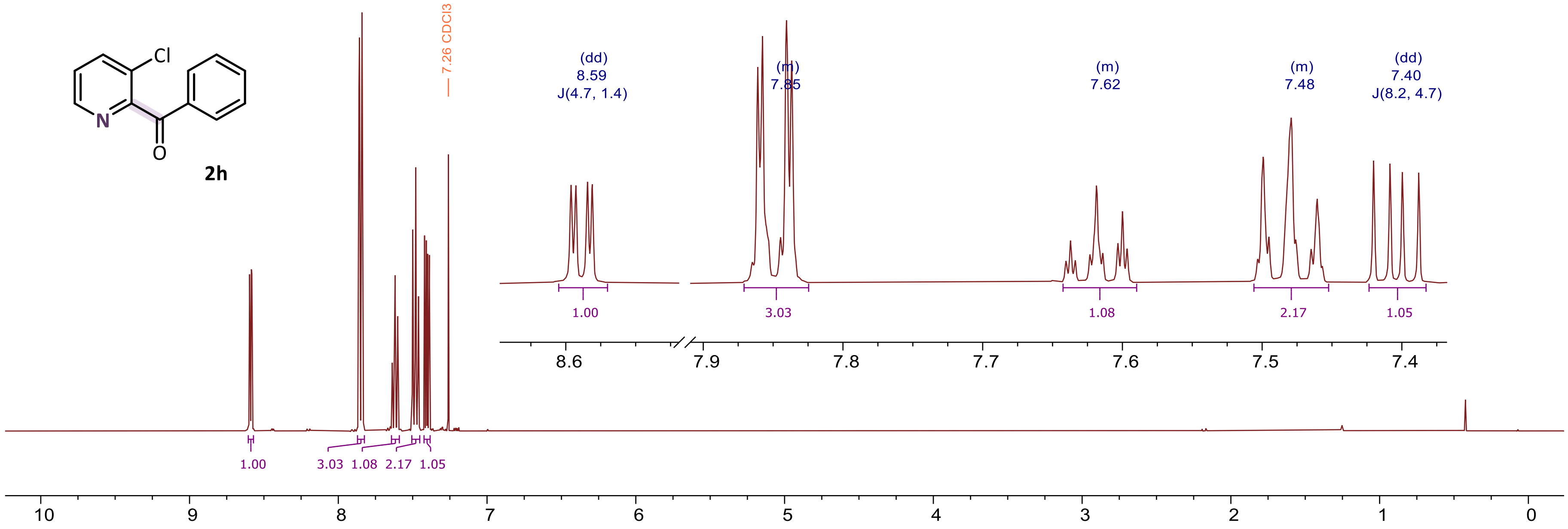

^{13}C NMR (100 MHz, $CDCl_3$):

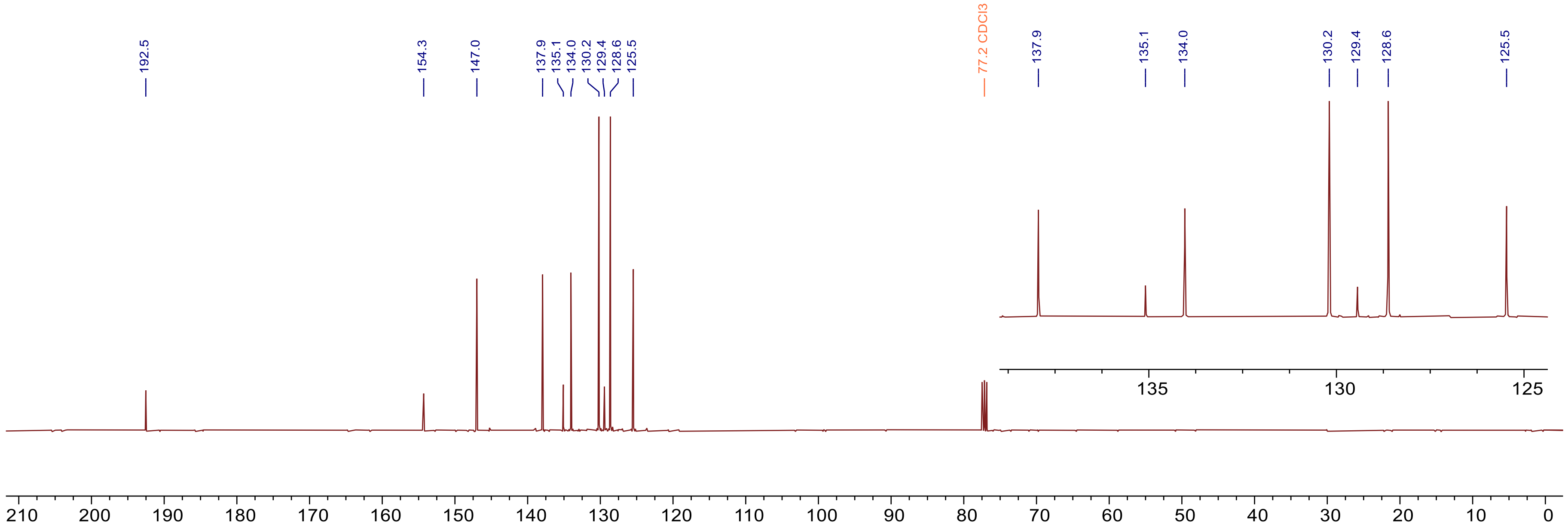

^{1}H NMR (400 MHz, $CDCl_3$):

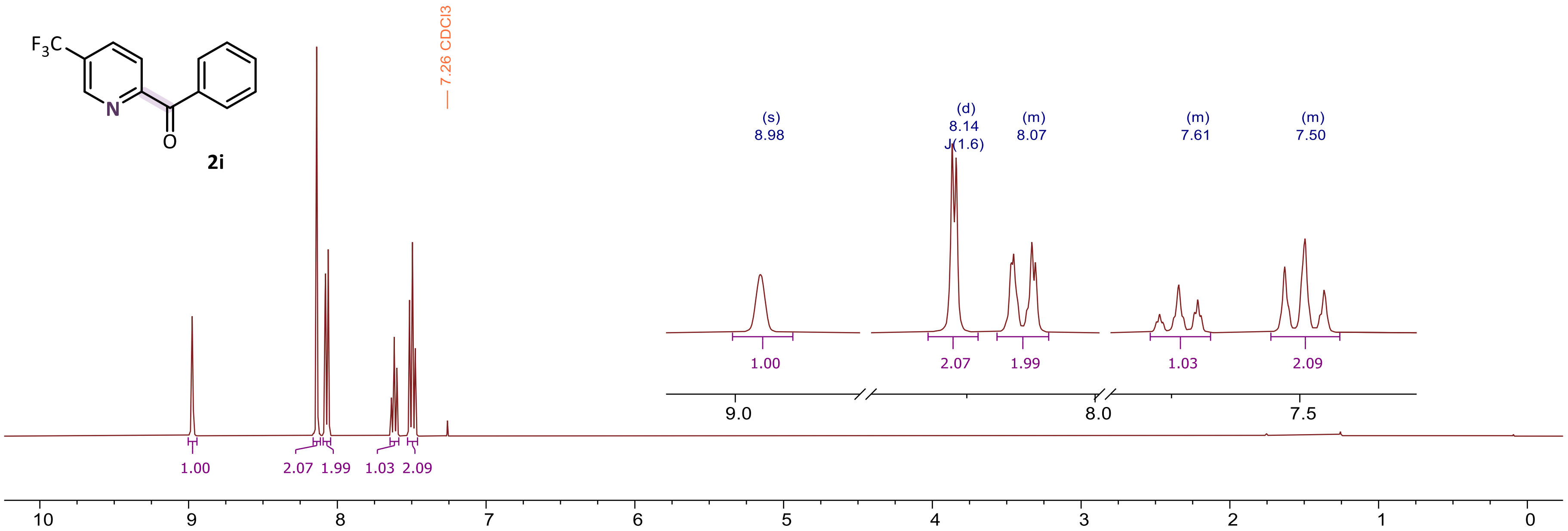

^{13}C NMR (100 MHz, $CDCl_3$):

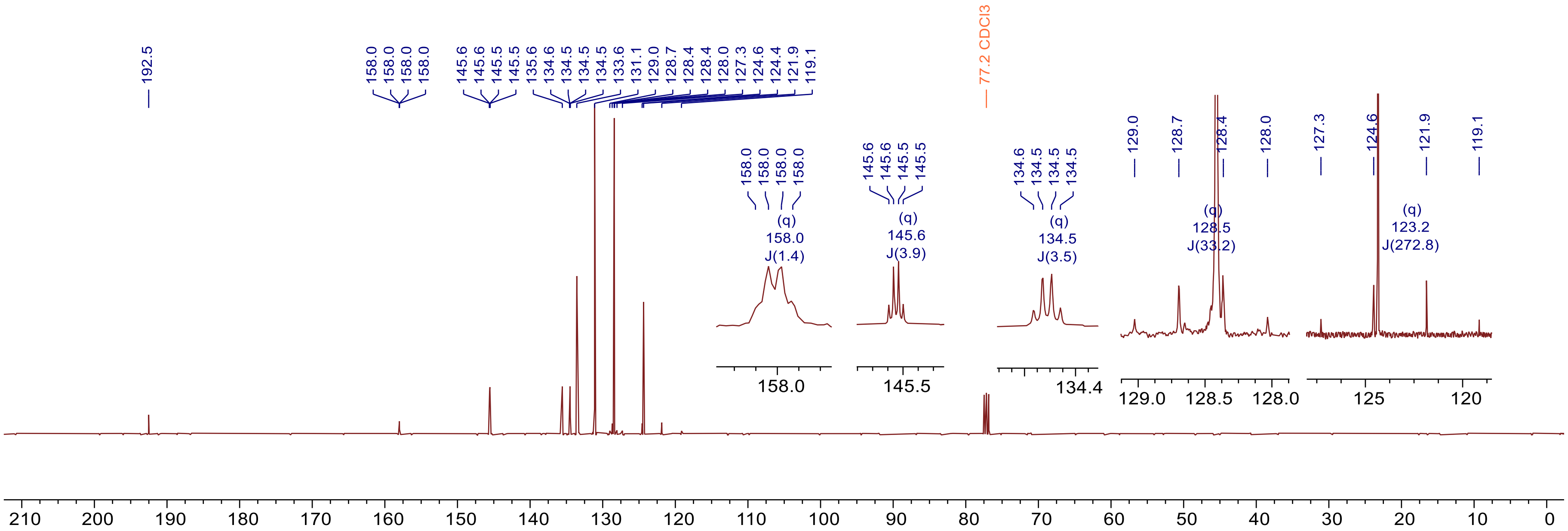

^{19}F NMR (376 MHz, CDCl$_3$):

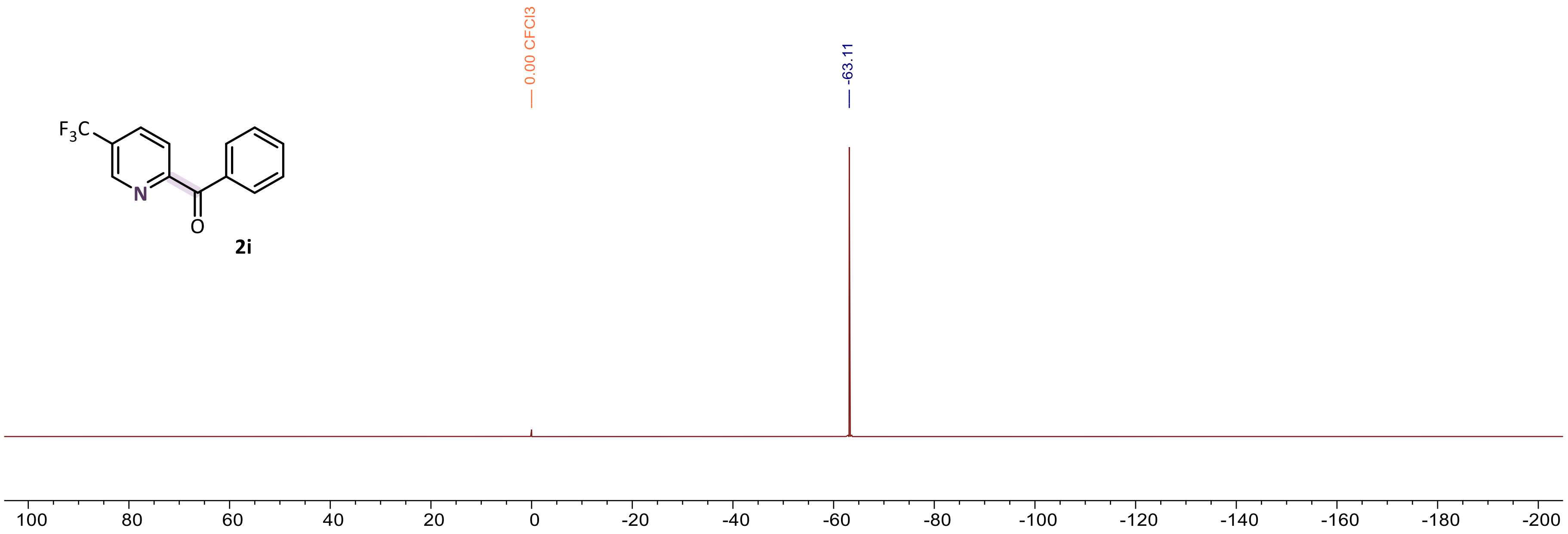

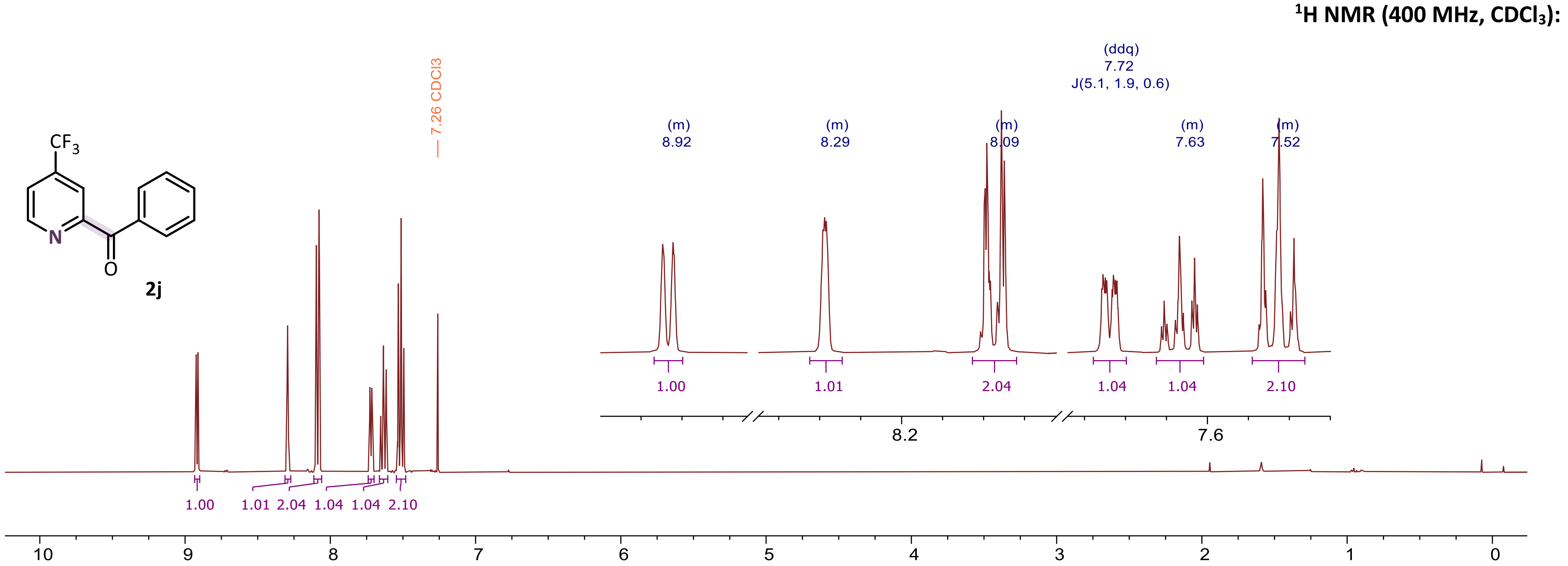

^{1}H NMR (400 MHz, CDCl$_3$):
2j
7.26 CDCl3
(m) 8.92
(m) 8.29
(m) 8.09
(ddq) 7.72 J(5.1, 1.9, 0.6)
(m) 7.63
(m) 7.52
1.00
1.01
2.04
1.04
1.04
2.10

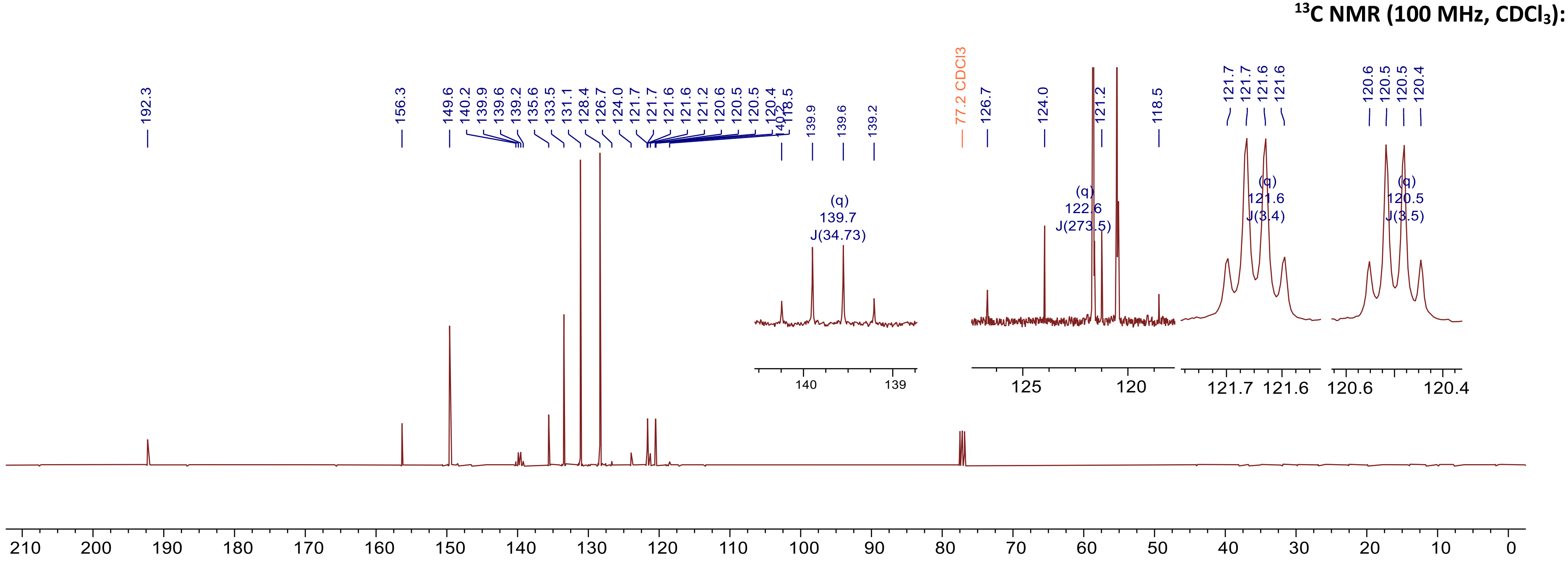

^{13}C NMR (100 MHz, CDCl$_3$):
192.3
156.3
149.6
140.2
139.9
139.6
139.2
135.6
133.5
131.1
128.4
126.7
124.0
121.7
121.7
121.6
121.6
121.2
120.6
120.5
120.5
120.4
118.5
77.2 CDCl3
(q) 139.7 J(34.73)
(q) 122.6 J(273.5)
(q) 121.6 J(3.4)
(q) 120.5 J(3.5)

^{19}F NMR (376 MHz, $CDCl_3$):

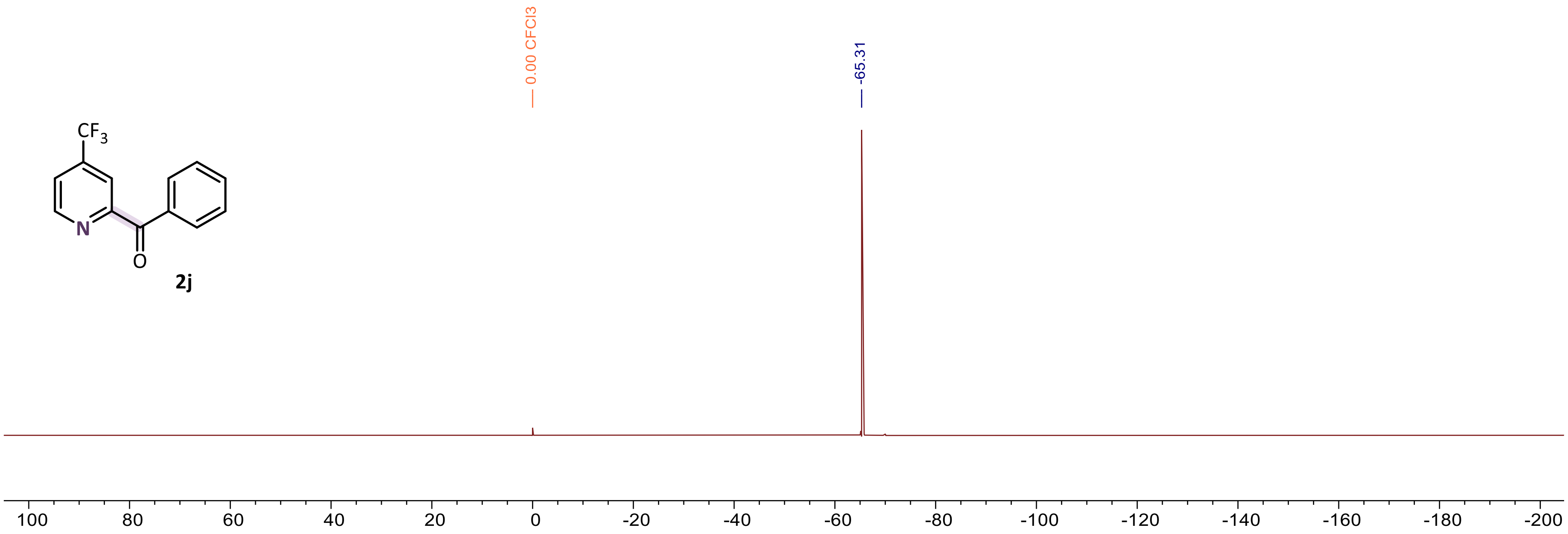

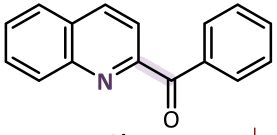

2k

^{1}H NMR (400 MHz, CDCl$_3$):

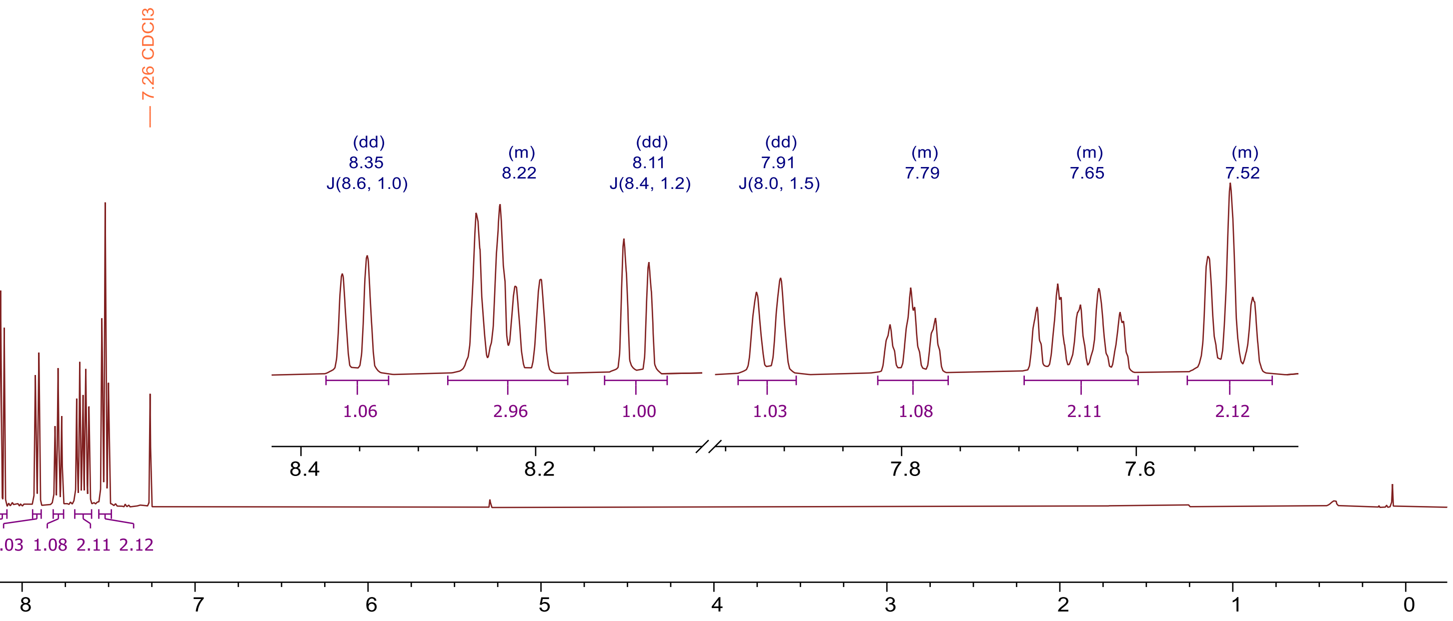

^{13}C NMR (100 MHz, CDCl$_3$):

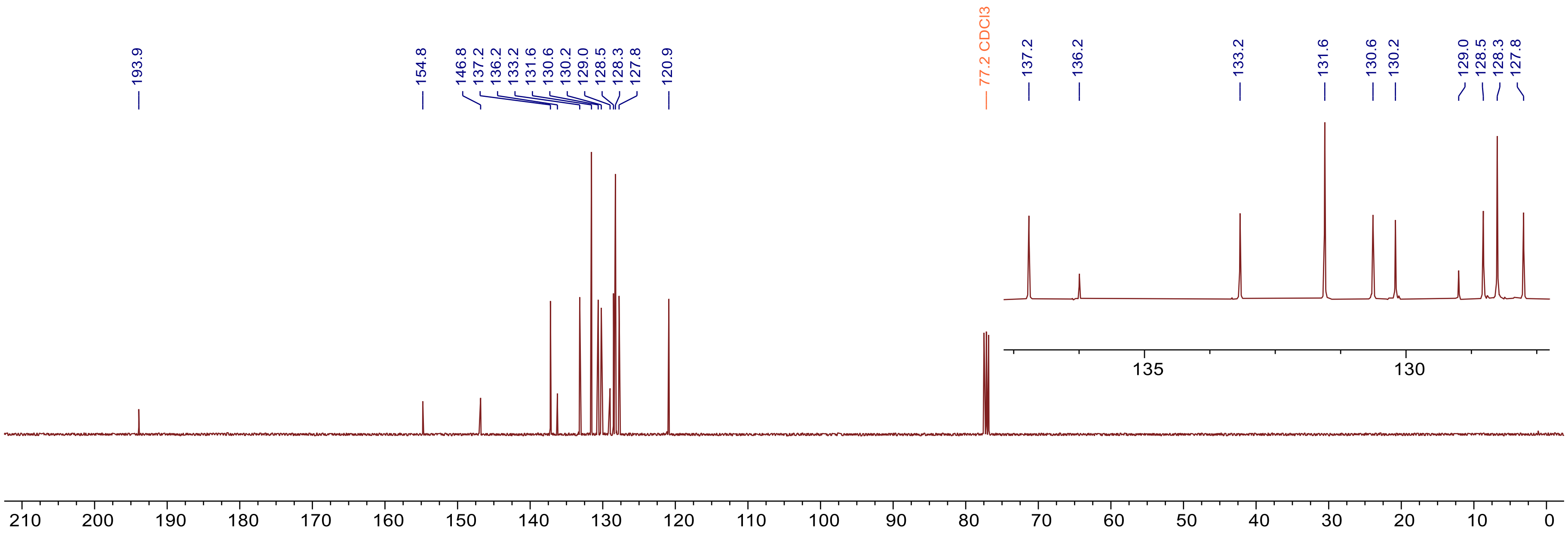

^{1}H NMR (400 MHz, $CDCl_3$):

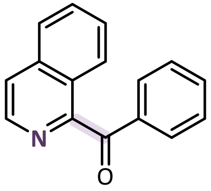

2l

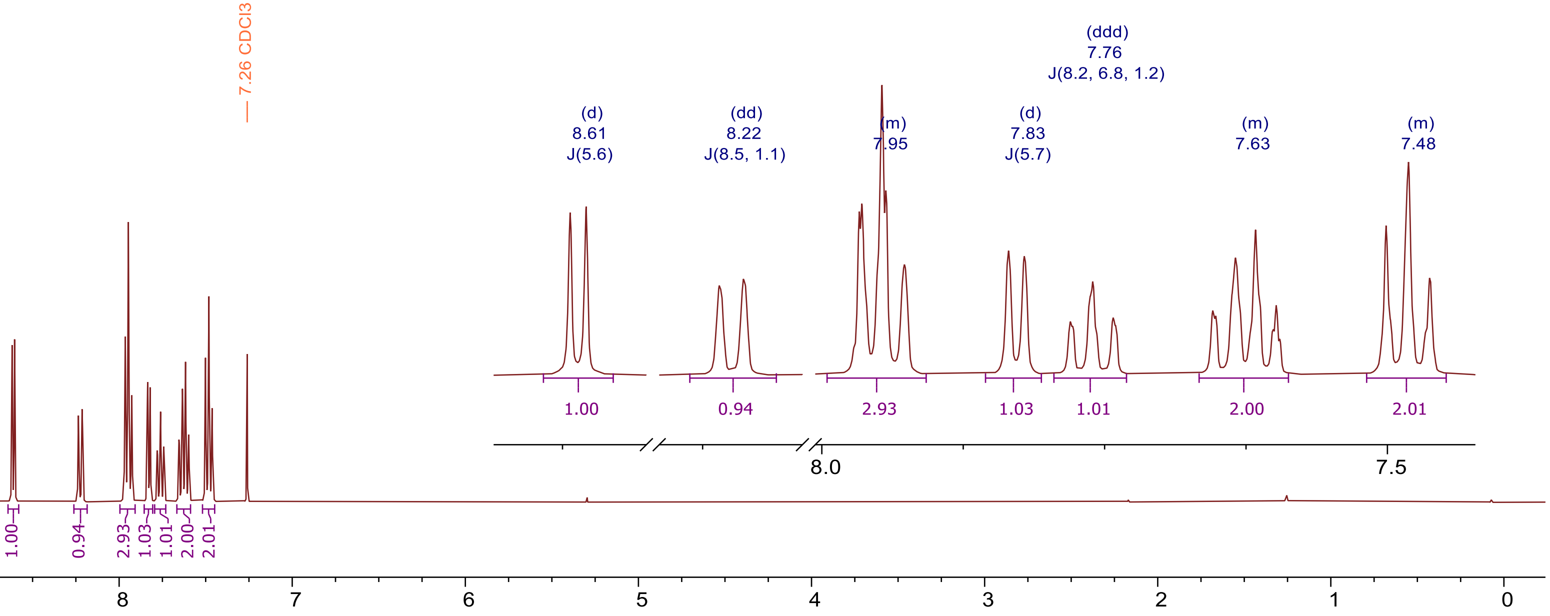

^{13}C NMR (100 MHz, $CDCl_3$):

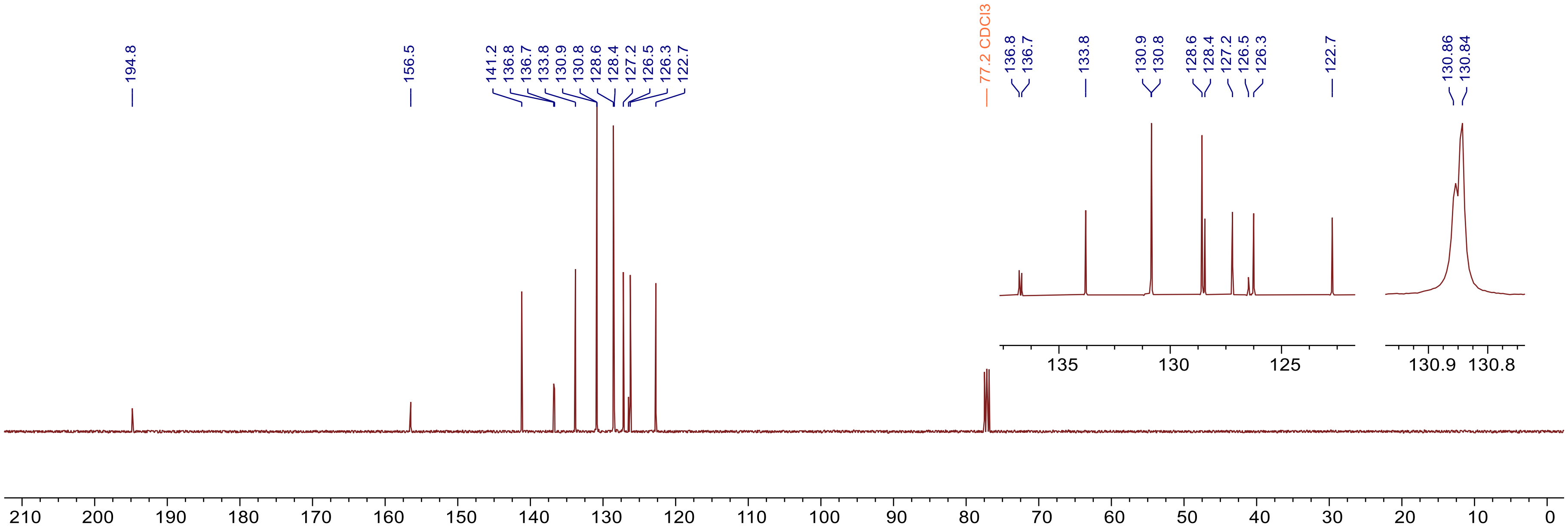

^{1}H NMR (400 MHz, $CDCl_3$):

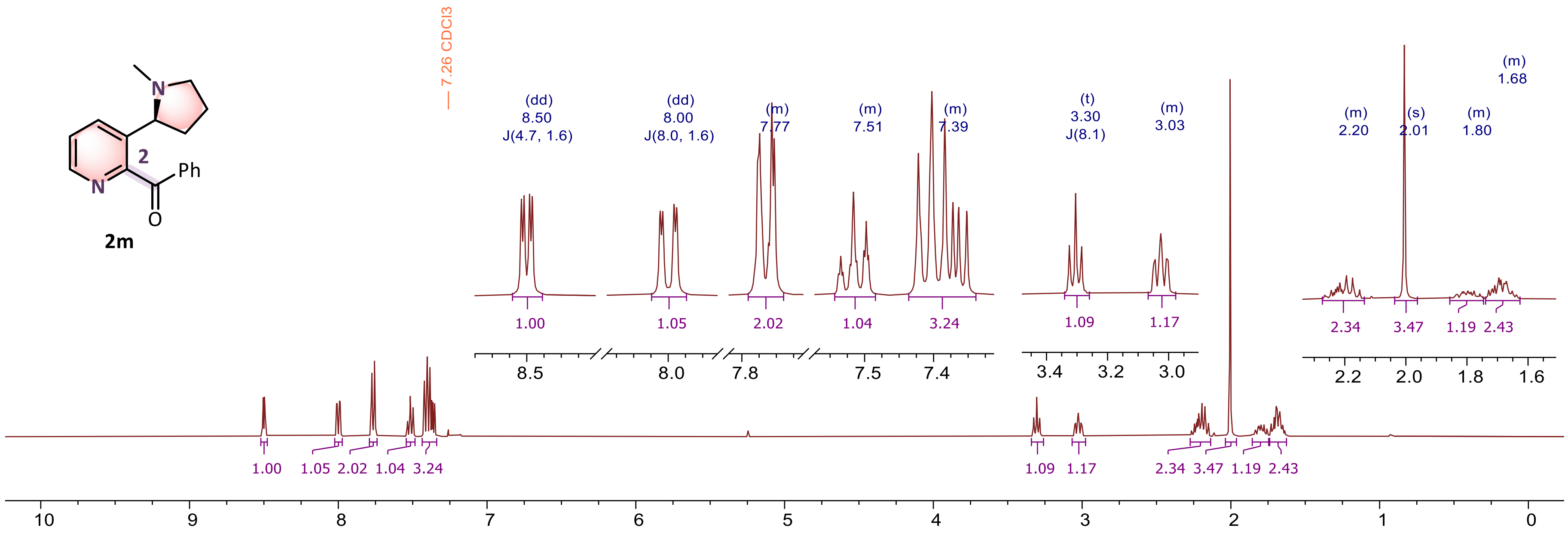

^{13}C NMR (100 MHz, $CDCl_3$):

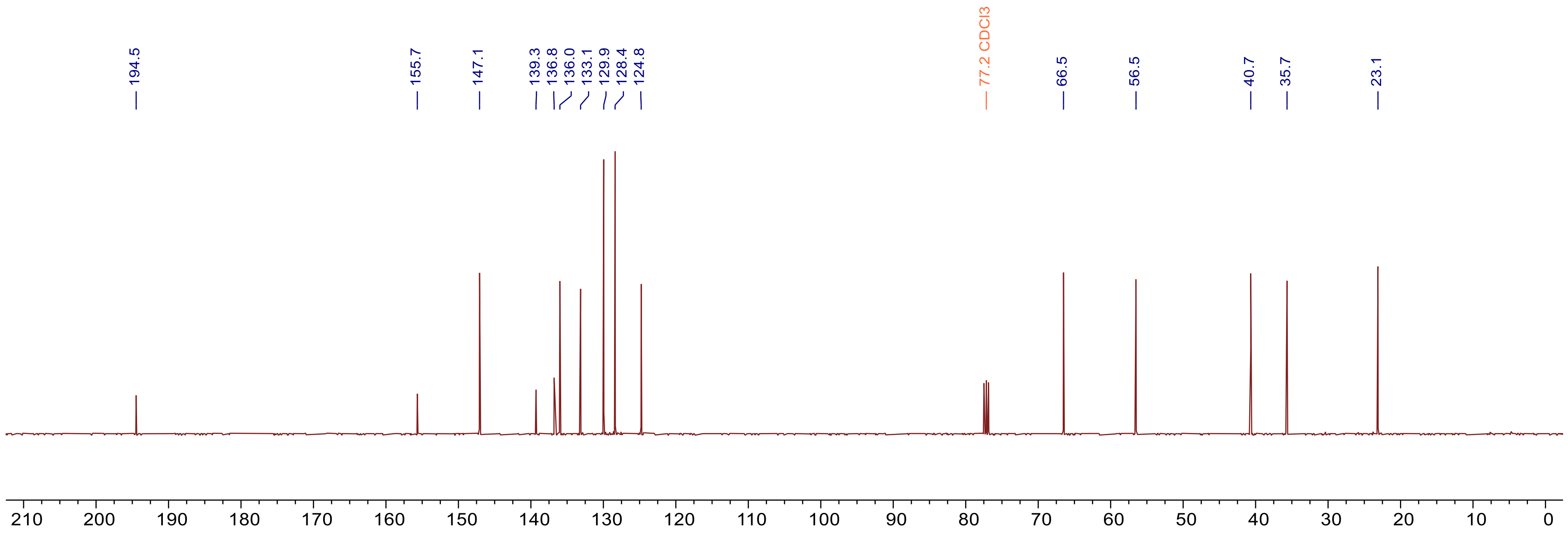

^{1}H NMR (400 MHz, $CDCl_3$):

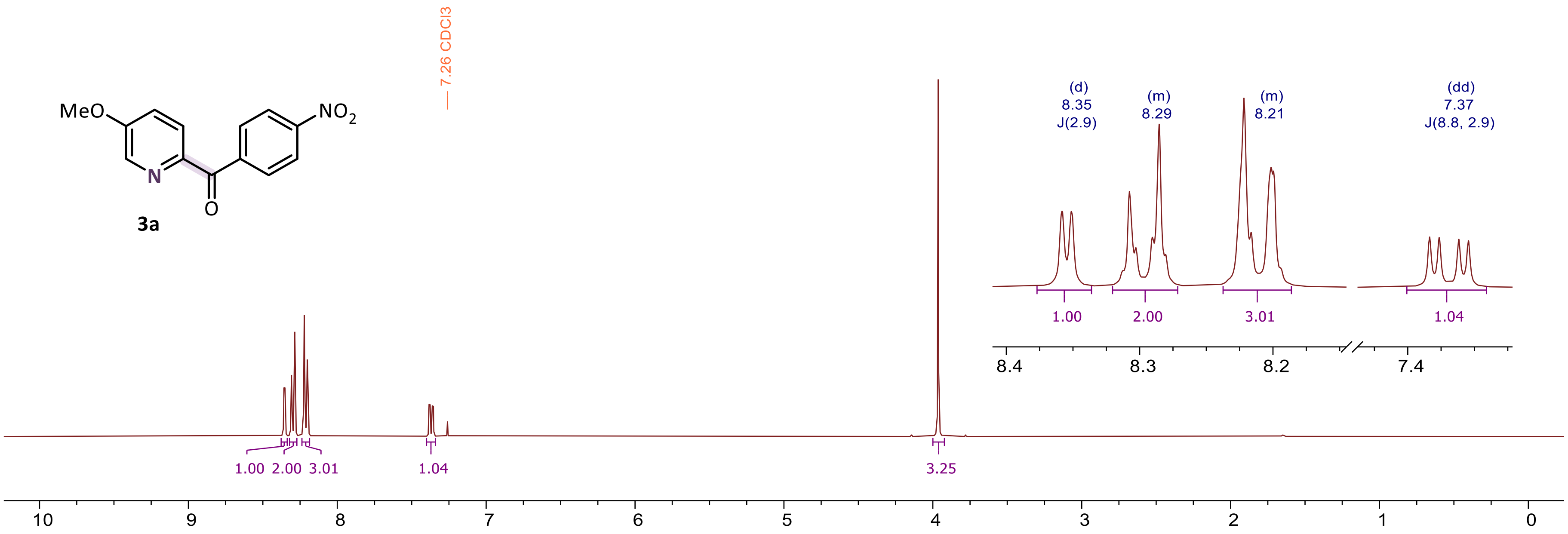

3a

^{13}C NMR (100 MHz, $CDCl_3$):

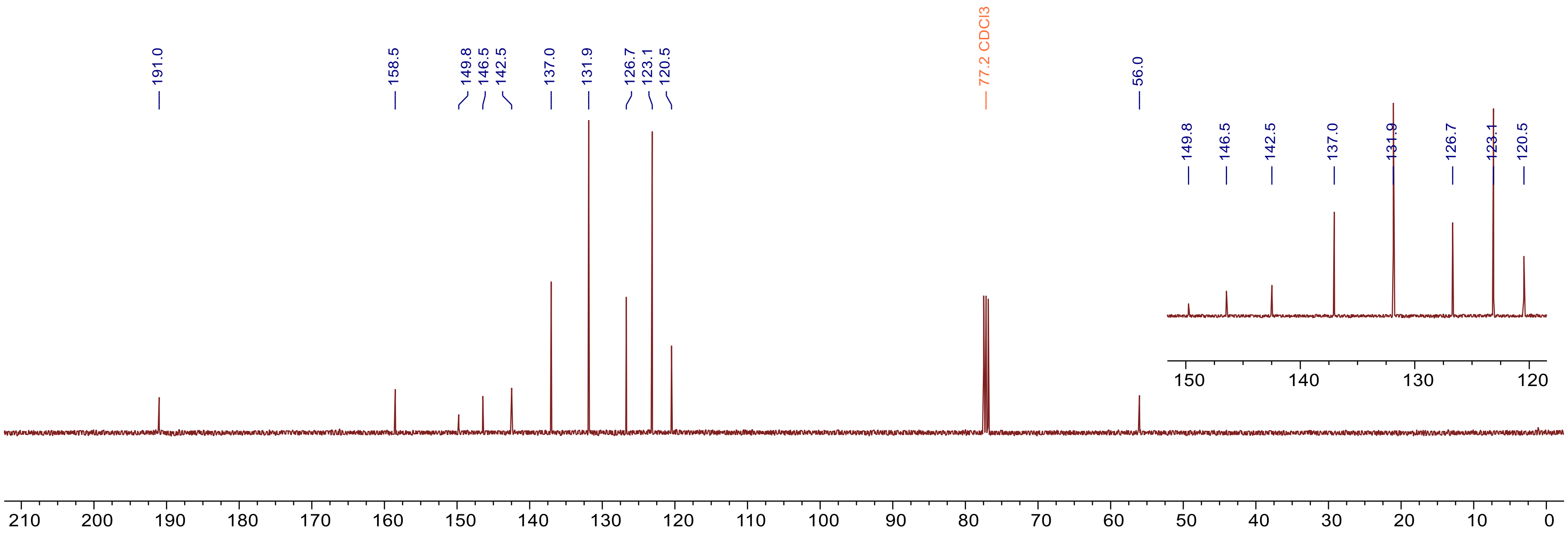

^{1}H NMR (400 MHz, $CDCl_3$):

3b

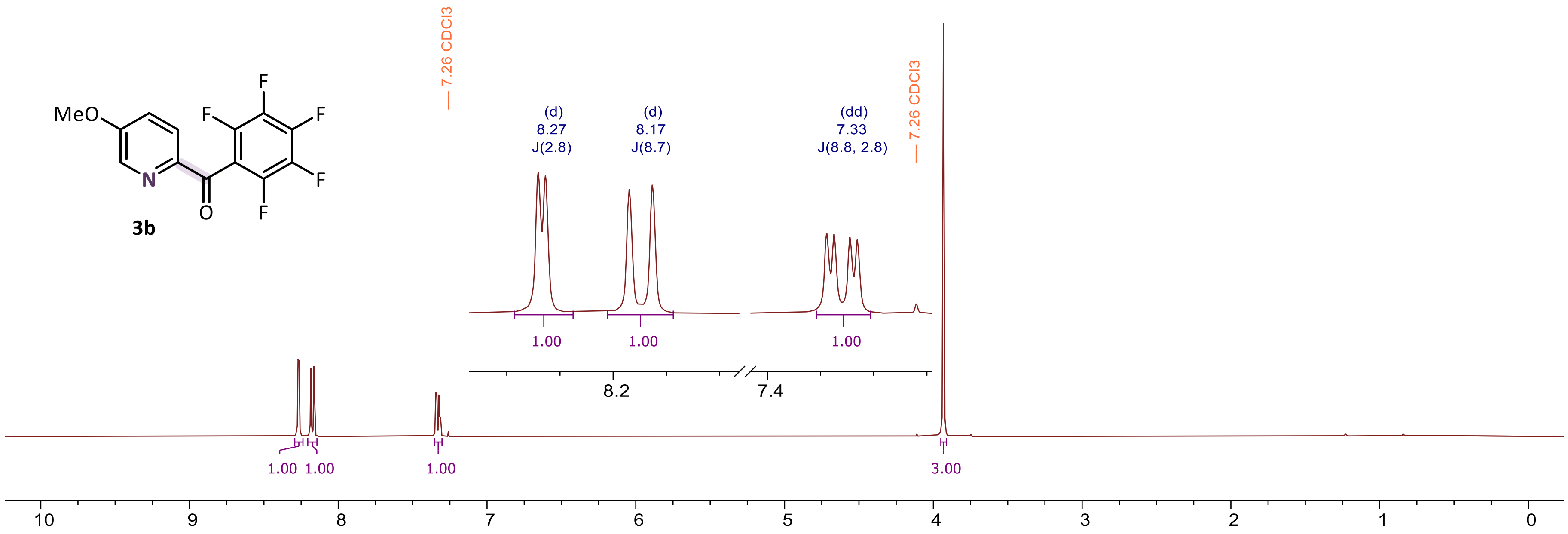

^{13}C NMR (100 MHz, $CDCl_3$):

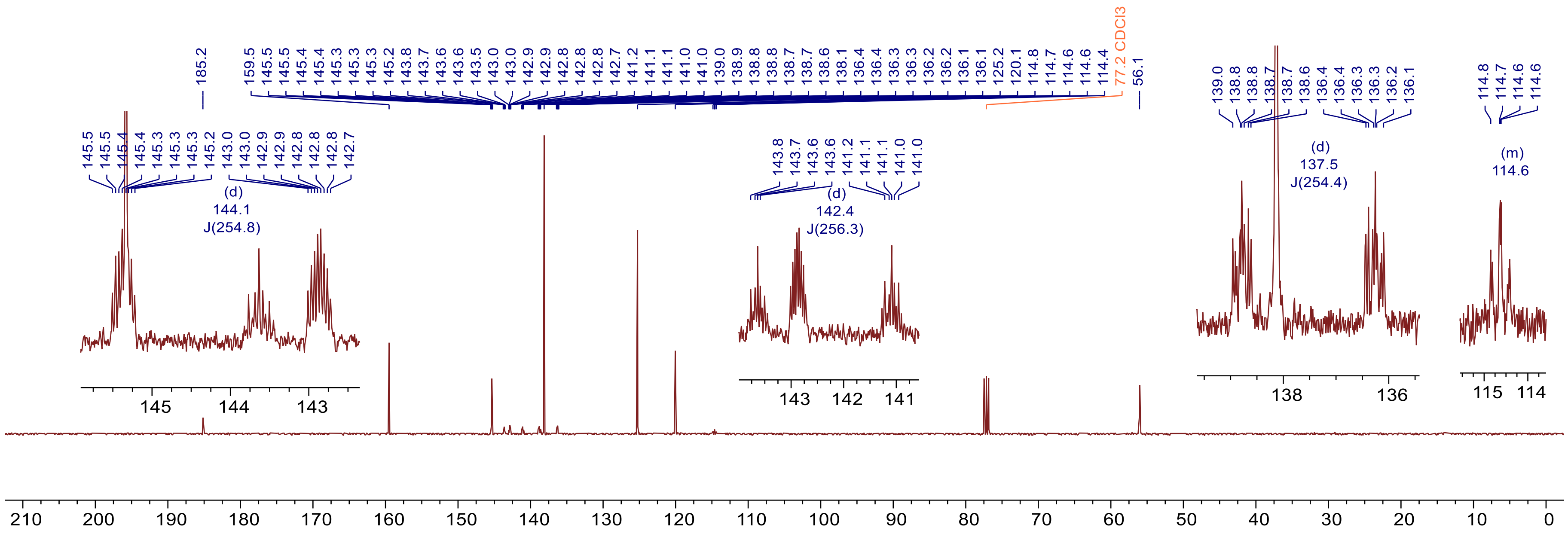

^{19}F NMR (376 MHz, $CDCl_3$):

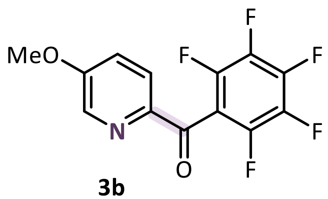

3b

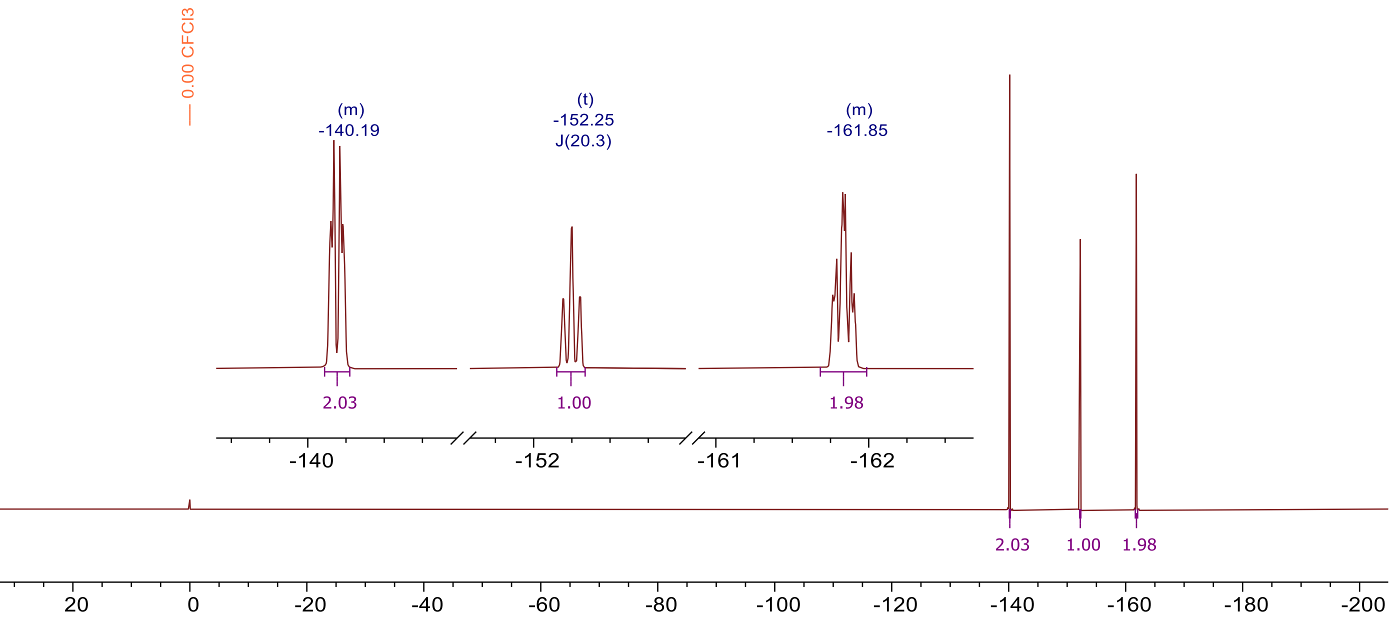

^1H NMR (400 MHz, $CDCl_3$):

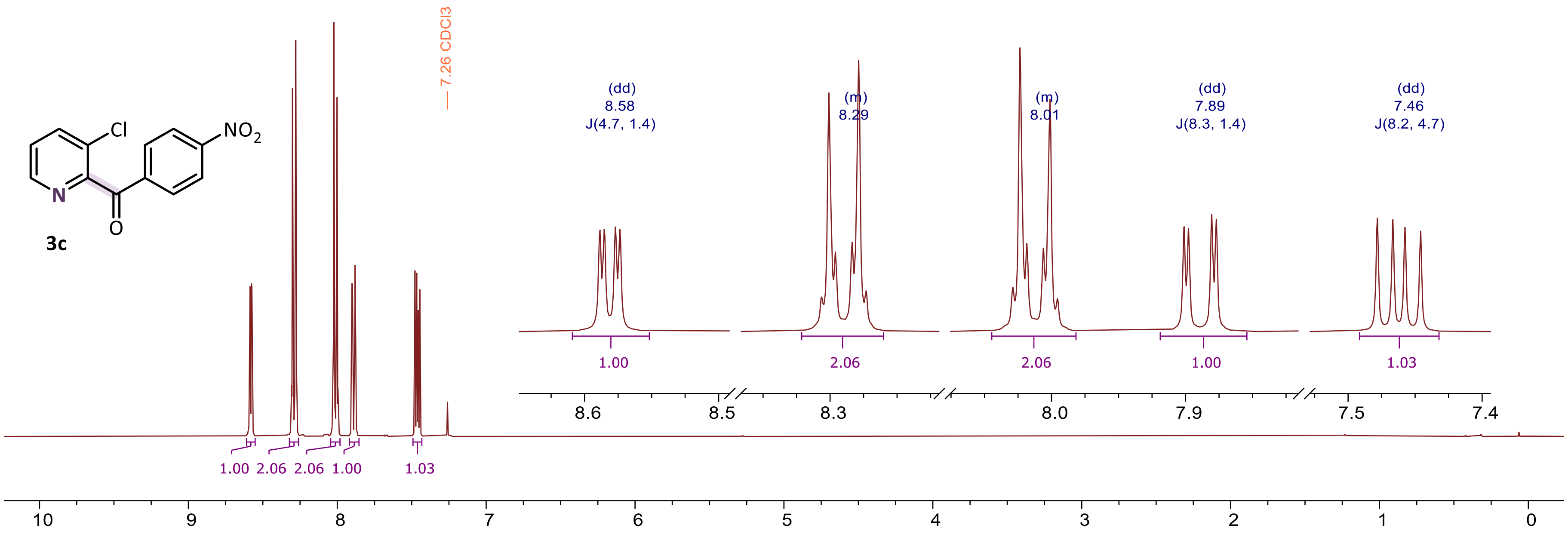

^{13}C NMR (100 MHz, $CDCl_3$):

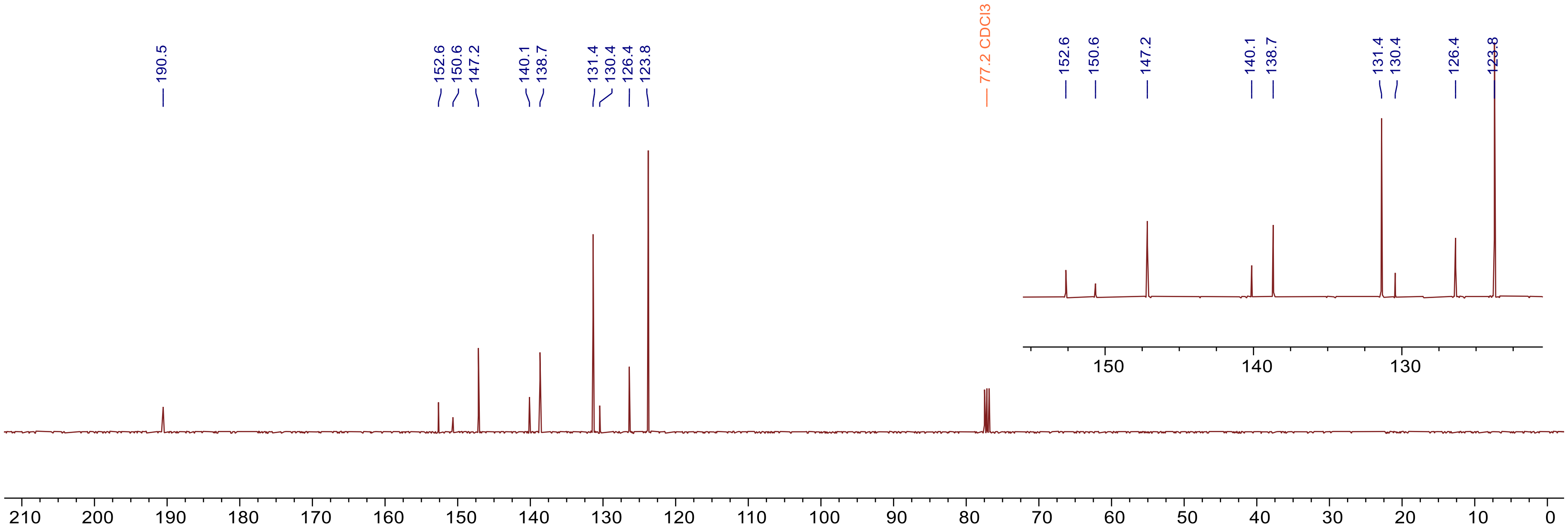

^{1}H NMR (400 MHz, $CDCl_3$):

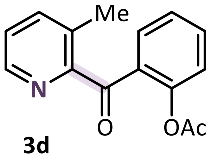

3d

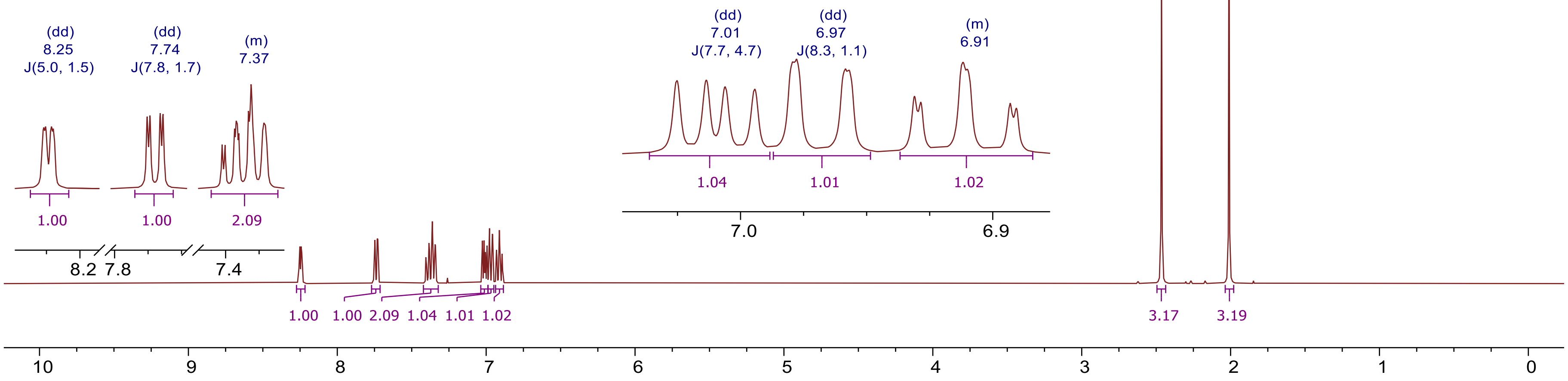

^{13}C NMR (100 MHz, $CDCl_3$):

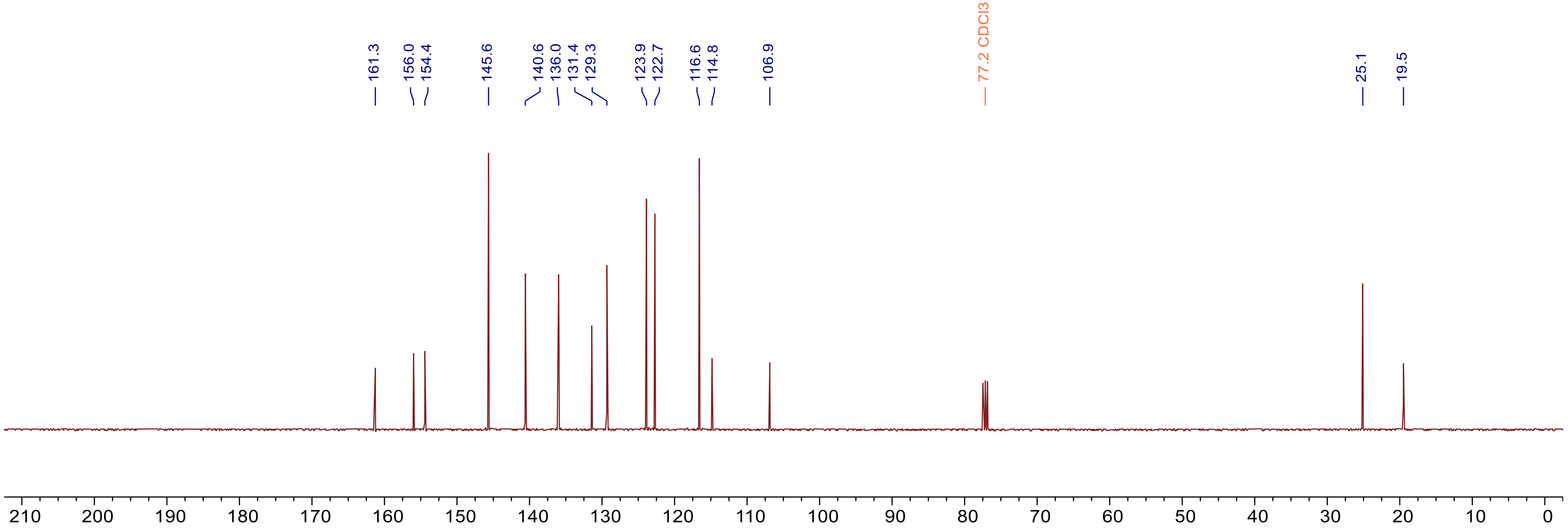

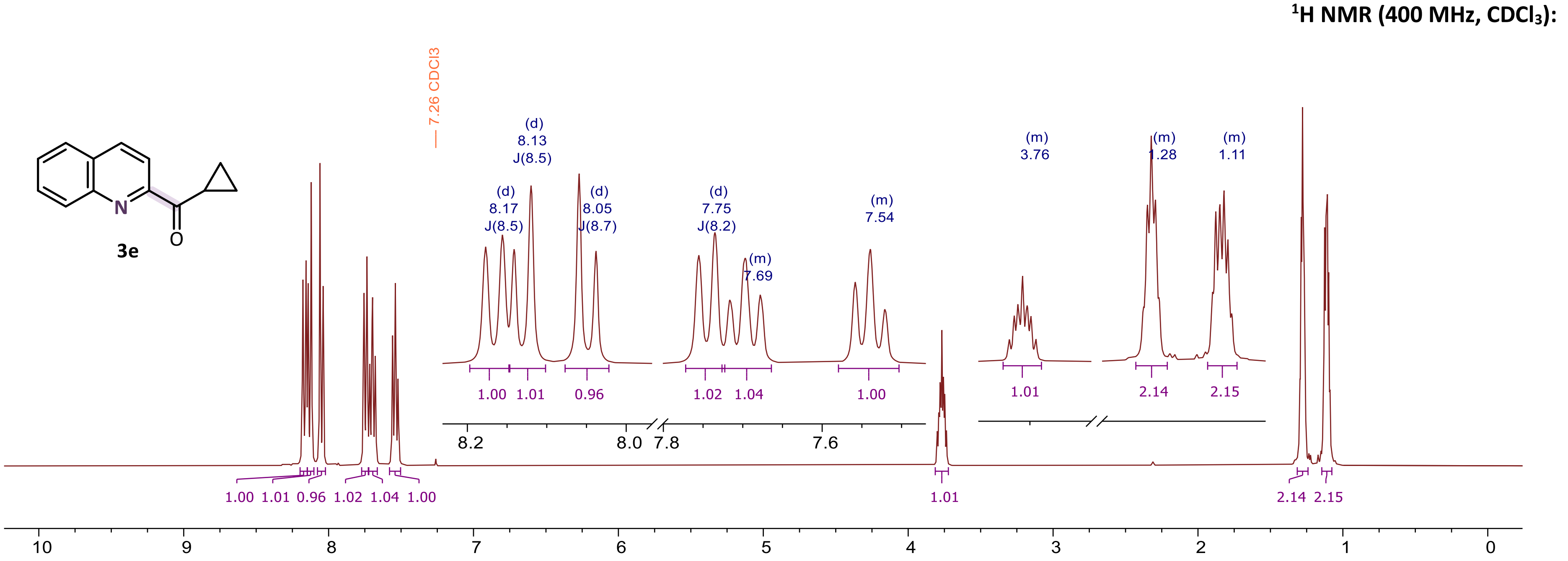

^{1}H NMR (400 MHz, $CDCl_3$):
3e
N
O
7.26 CDCl3
(d) 8.13 J(8.5)
(d) 8.17 J(8.5)
(d) 8.05 J(8.7)
(d) 7.75 J(8.2)
(m) 7.69
(m) 7.54
(m) 3.76
(m) 1.28
(m) 1.11
1.00 1.01 0.96 1.02 1.04 1.00 1.01 2.14 2.15

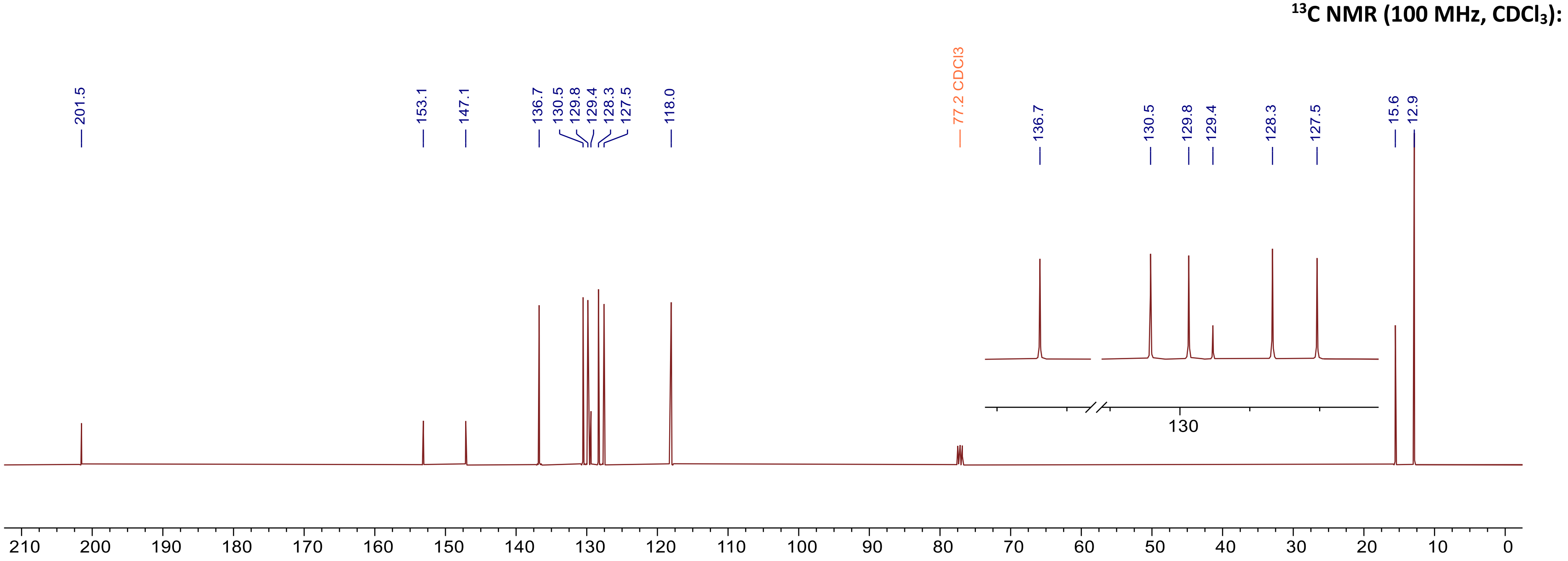

^{13}C NMR (100 MHz, $CDCl_3$):
201.5
153.1
147.1
136.7
130.5
129.8
129.4
128.3
127.5
118.0
77.2 CDCl3
15.6
12.9

^1H NMR (400 MHz, $CDCl_3$):

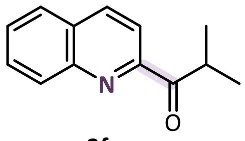

3f

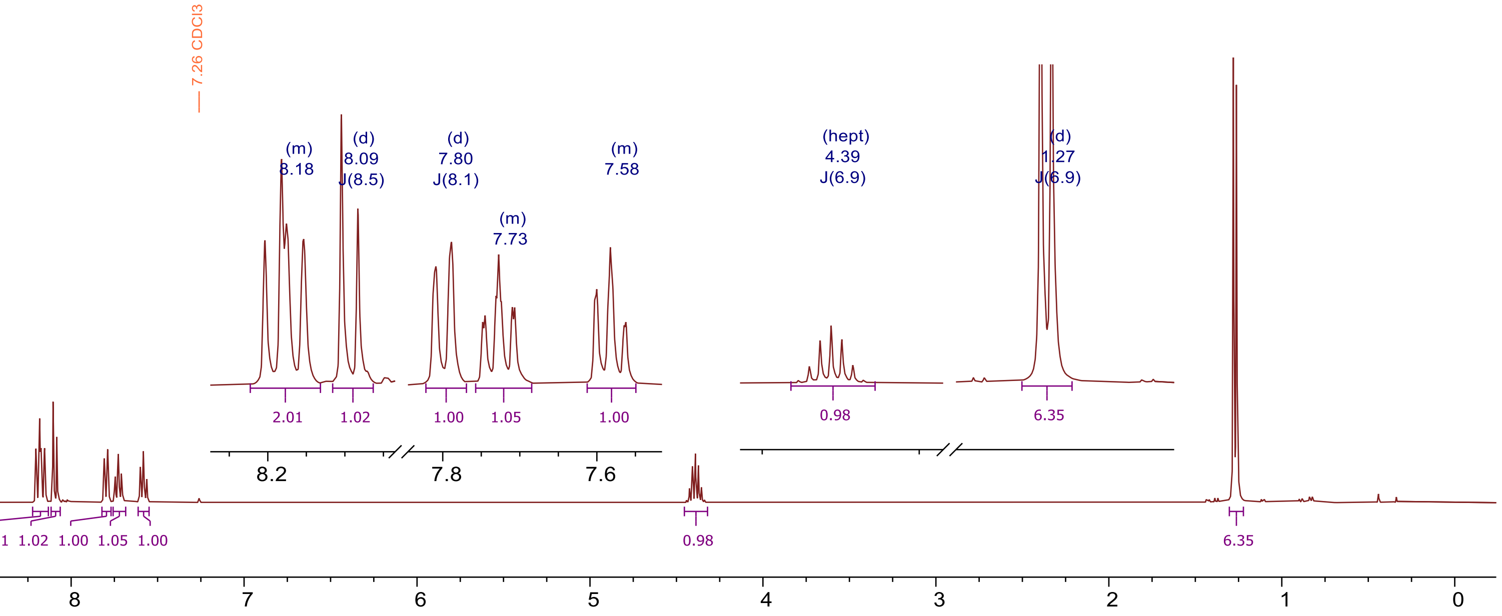

^{13}C NMR (100 MHz, $CDCl_3$):

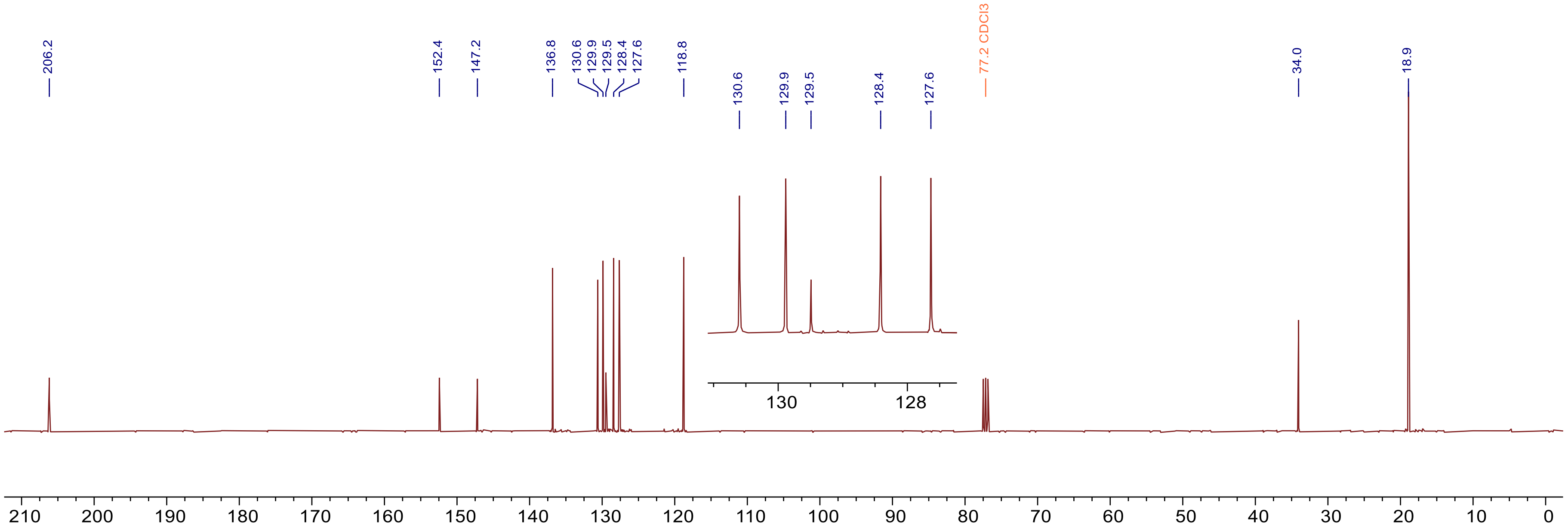

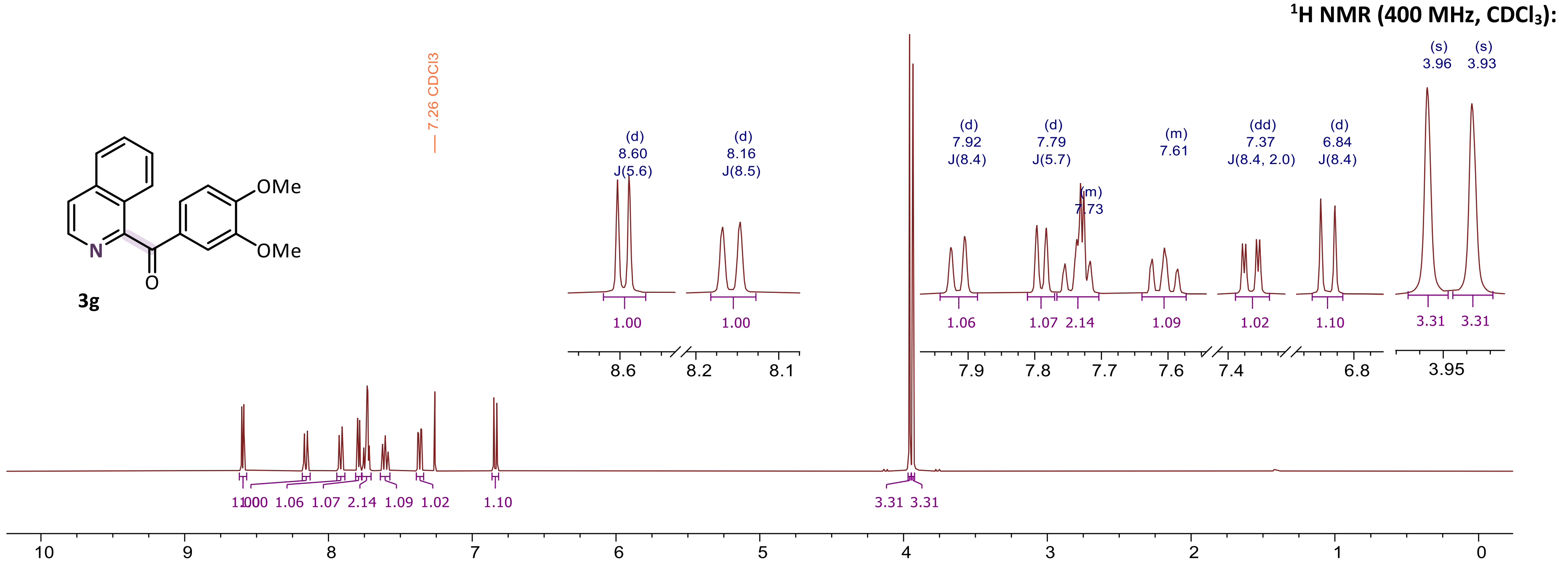

3g

^{1}H NMR (400 MHz, CDCl$_3$):
7.26 CDCl3
(d) 8.60 J(5.6)
(d) 8.16 J(8.5)
(d) 7.92 J(8.4)
(d) 7.79 J(5.7)
(m) 7.73
(m) 7.61
(dd) 7.37 J(8.4, 2.0)
(d) 6.84 J(8.4)
(s) 3.96
(s) 3.93

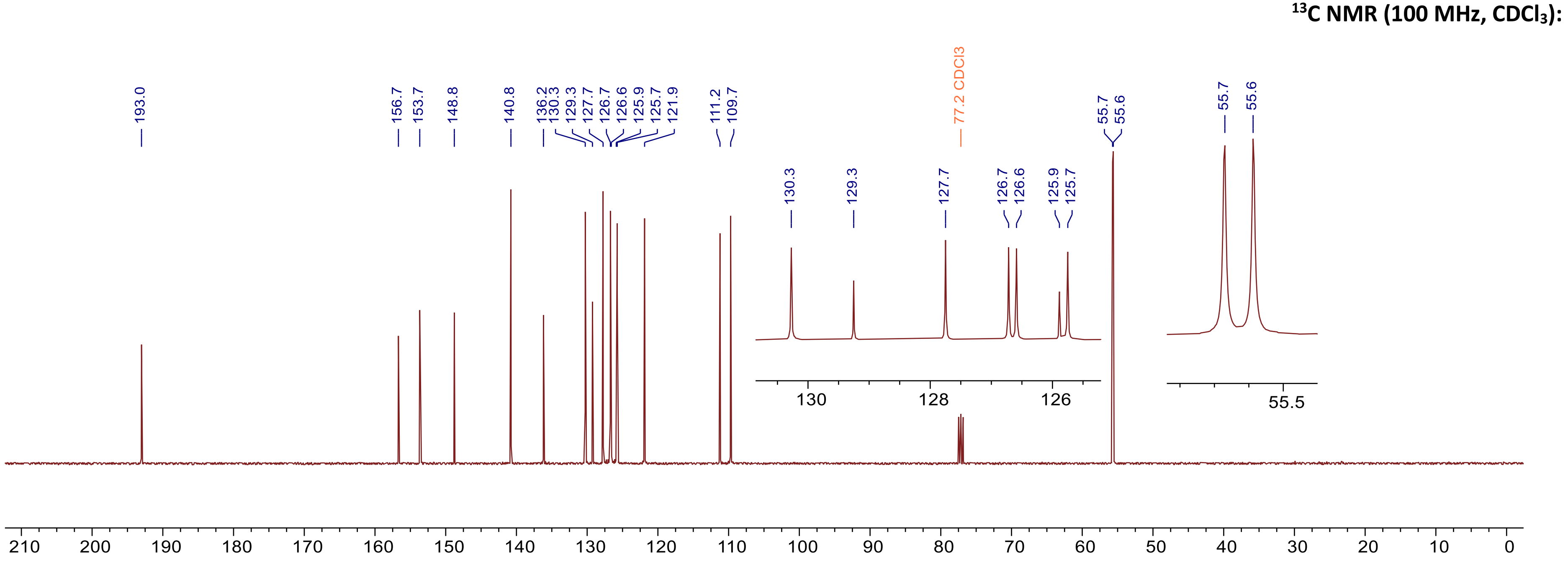

^{13}C NMR (100 MHz, CDCl$_3$):
193.0
156.7
153.7
148.8
140.8
136.2
130.3
129.3
127.7
126.7
126.6
125.9
125.7
121.9
111.2
109.7
77.2 CDCl3
55.7
55.6

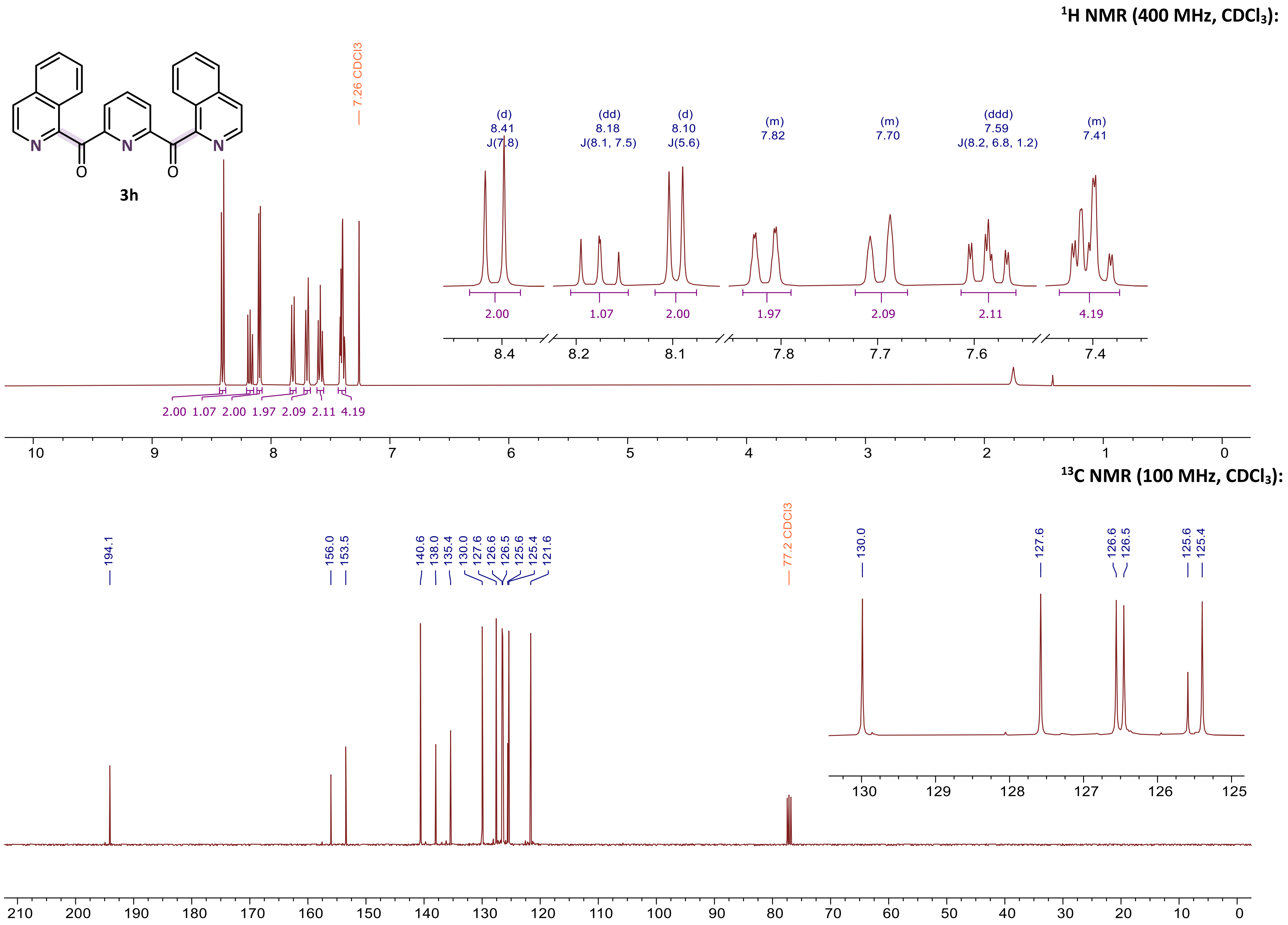

3h
¹H NMR (400 MHz, CDCl₃):
7.26 CDCl3
(d) 8.41 J(7.8)
(dd) 8.18 J(8.1, 7.5)
(d) 8.10 J(5.6)
(m) 7.82
(m) 7.70
(ddd) 7.59 J(8.2, 6.8, 1.2)
(m) 7.41
2.00 1.07 2.00 1.97 2.09 2.11 4.19
¹³C NMR (100 MHz, CDCl₃):
194.1
156.0
153.5
140.6
138.0
135.4
130.0
127.6
126.6
126.5
125.6
125.4
121.6
77.2 CDCl3

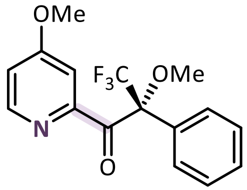

3i

^{1}H NMR (400 MHz, $CDCl_3$):

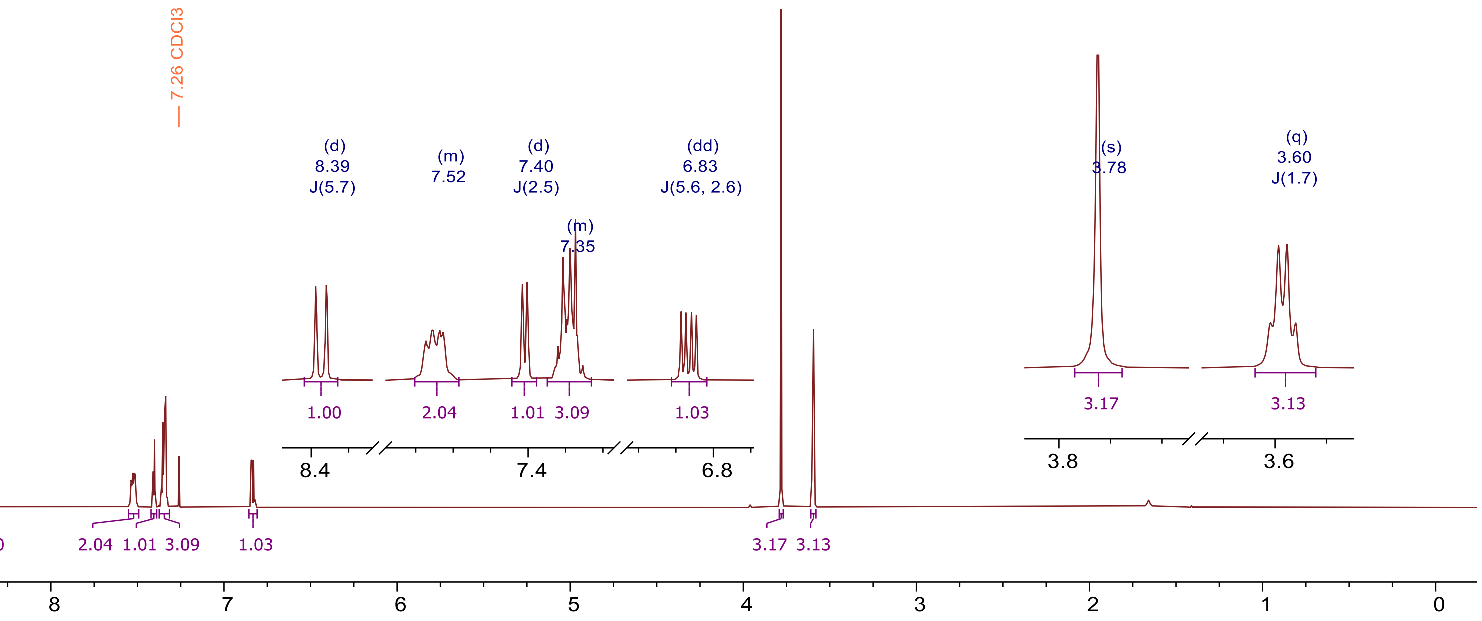

^{13}C NMR (100 MHz, $CDCl_3$):

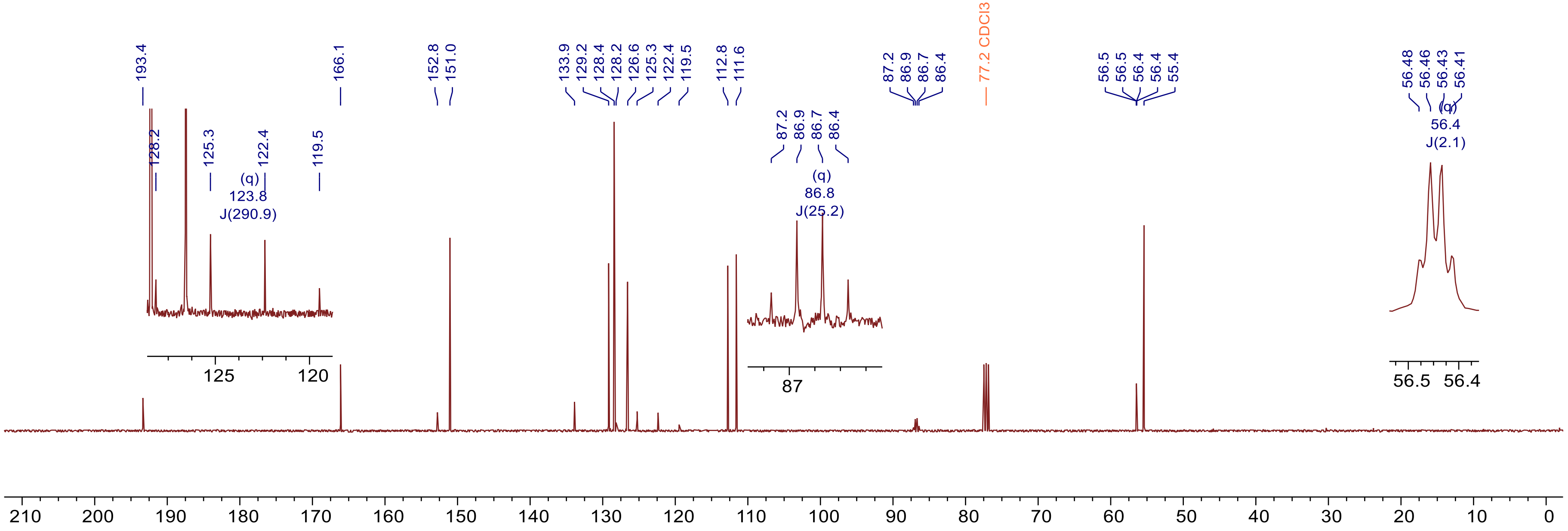

^{19}F NMR (376 MHz, $CDCl_3$):

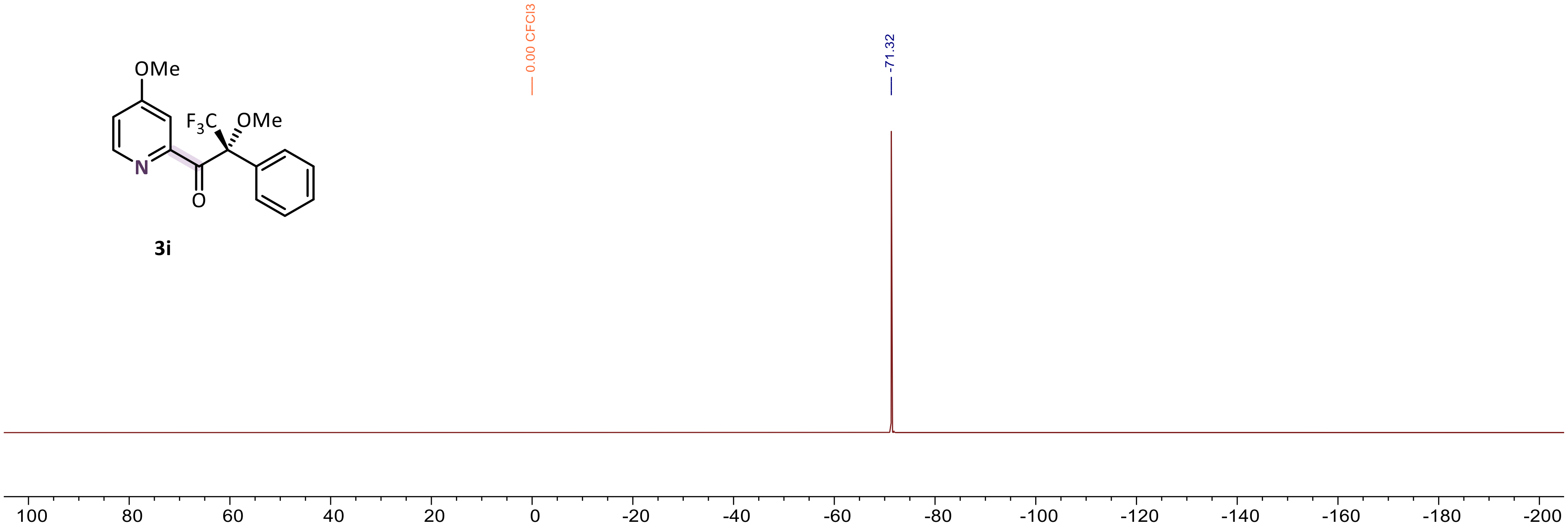

3i

^{1}H NMR (400 MHz, $CDCl_3$):

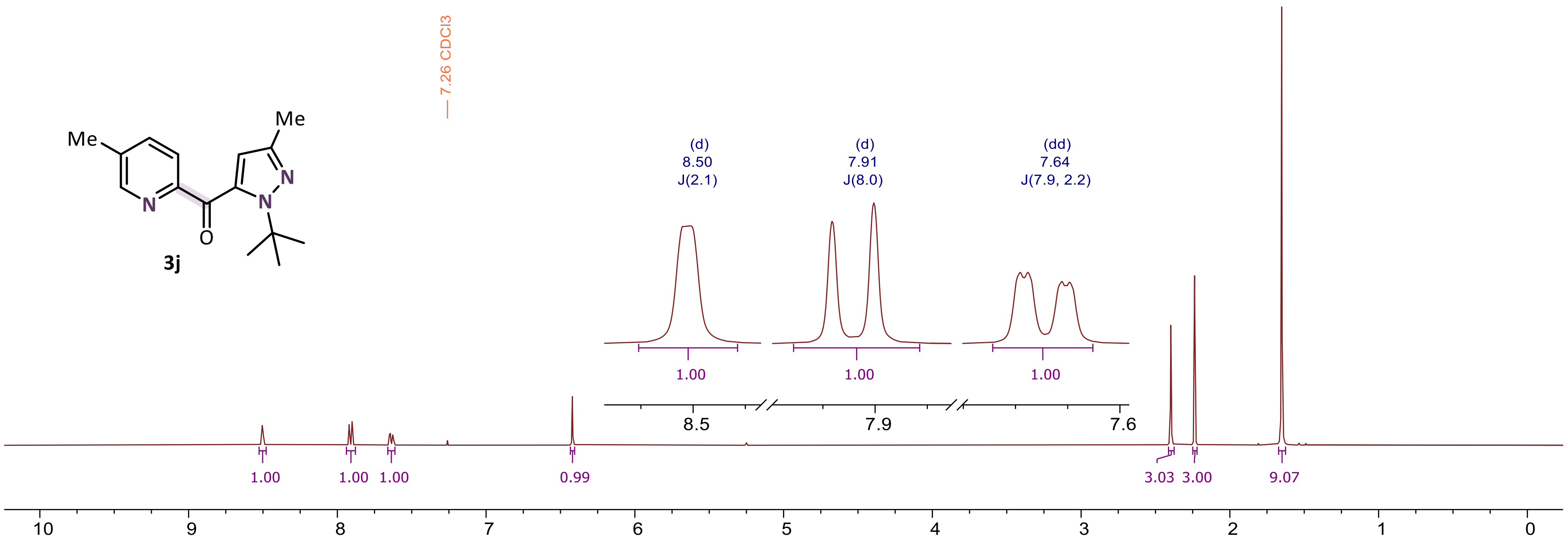

3j

^{13}C NMR (100 MHz, $CDCl_3$):

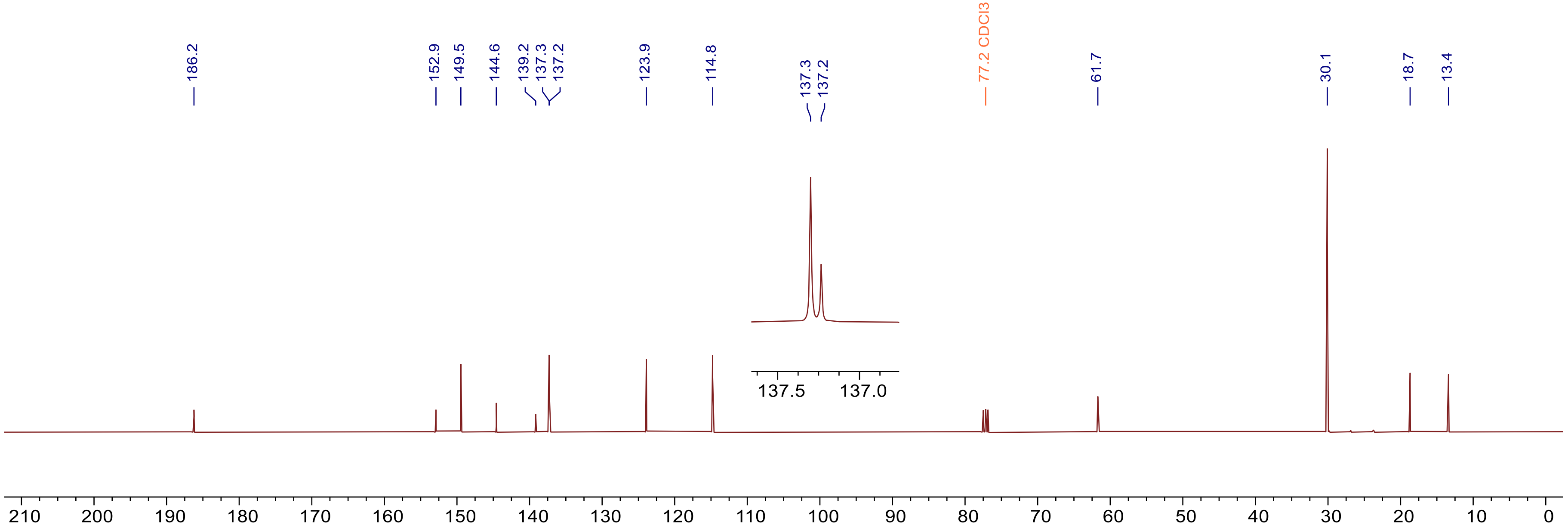

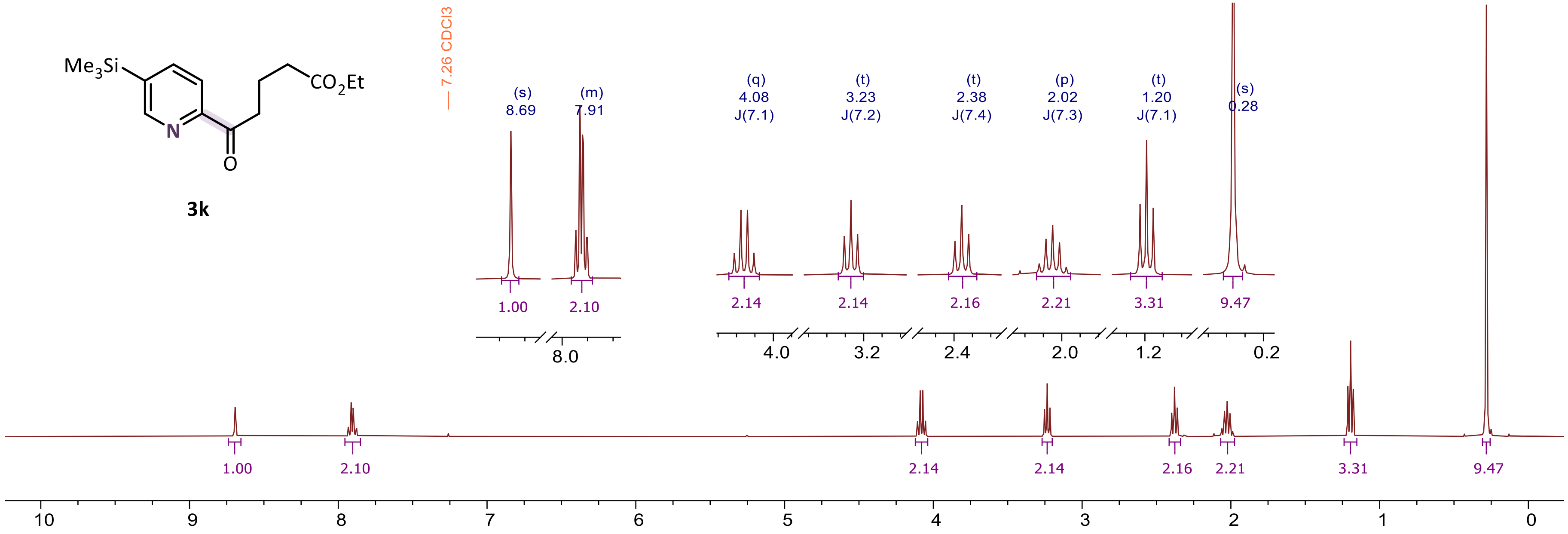

3k
^{1}H NMR (400 MHz, $CDCl_3$):
7.26 CDCl3
(s) 8.69
(m) 7.91
(q) 4.08 J(7.1)
(t) 3.23 J(7.2)
(t) 2.38 J(7.4)
(p) 2.02 J(7.3)
(t) 1.20 J(7.1)
(s) 0.28
1.00 2.10 2.14 2.14 2.16 2.21 3.31 9.47

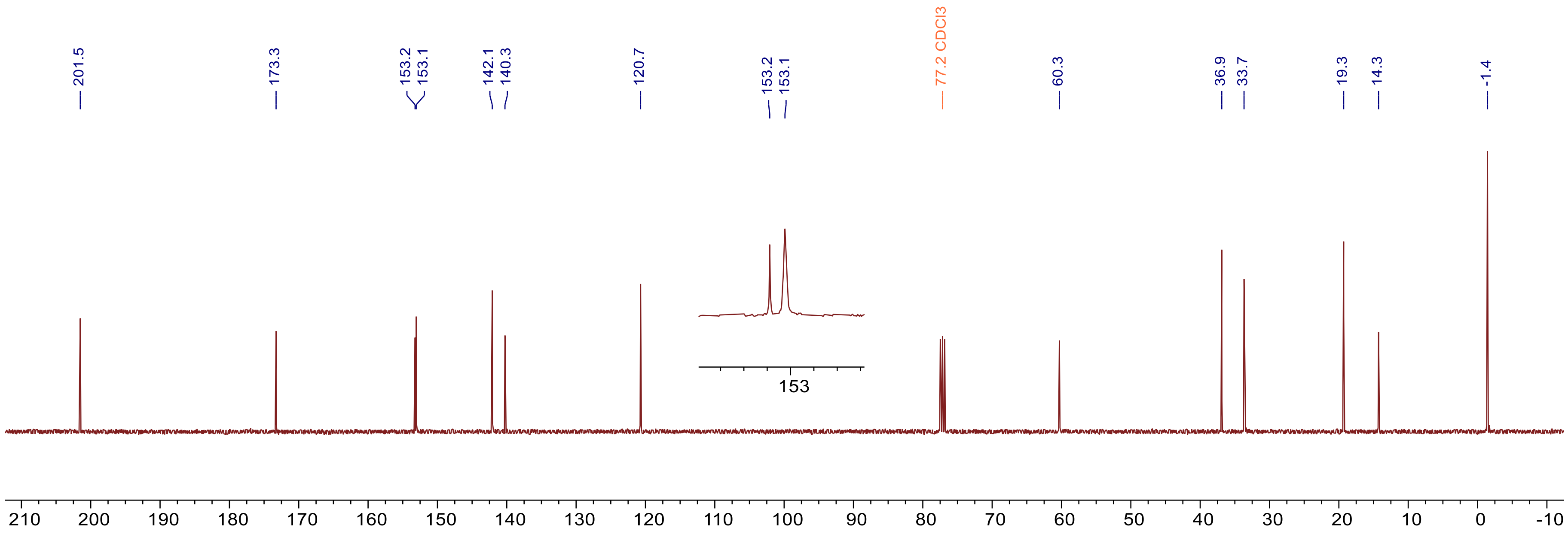

^{13}C NMR (100 MHz, $CDCl_3$):
201.5
173.3
153.2
153.1
142.1
140.3
120.7
77.2 CDCl3
60.3
36.9
33.7
19.3
14.3
-1.4

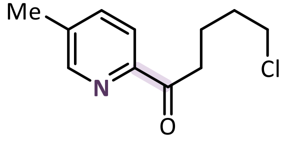

3l

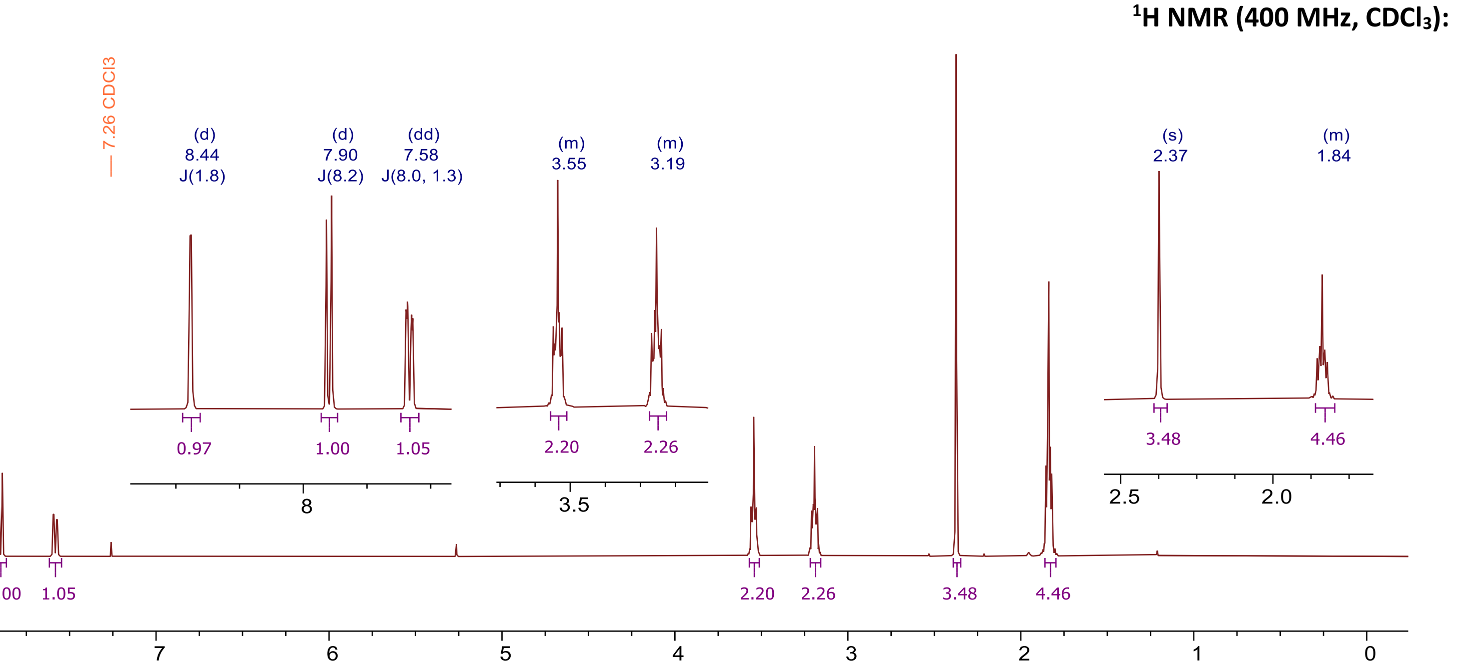
1H NMR (400 MHz, CDCl3):
7.26 CDCl3
(d) 8.44 J(1.8)
(d) 7.90 J(8.2)
(dd) 7.58 J(8.0, 1.3)
(m) 3.55
(m) 3.19
(s) 2.37
(m) 1.84
0.97
1.00
1.05
2.20
2.26
3.48
4.46

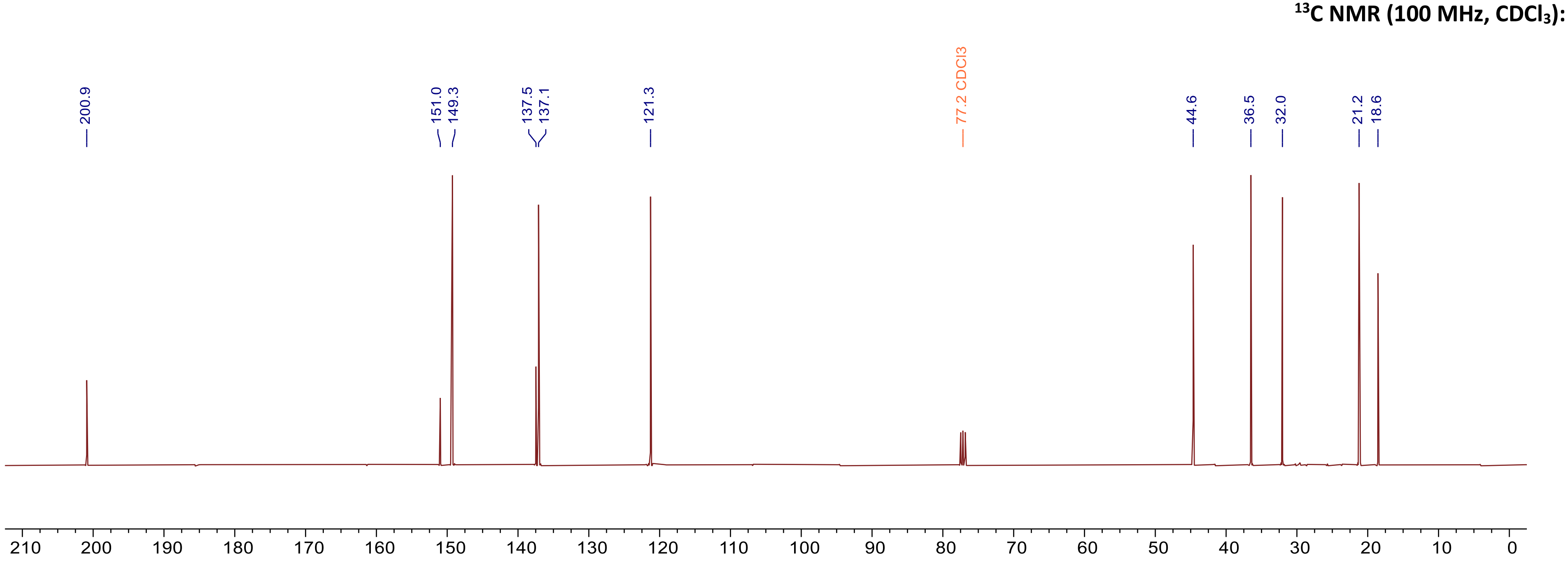
13C NMR (100 MHz, CDCl3):
200.9
151.0
149.3
137.5
137.1
121.3
77.2 CDCl3
44.6
36.5
32.0
21.2
18.6

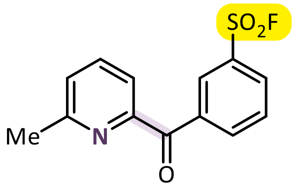

3m

^{1}H NMR (400 MHz, $CDCl_3$):

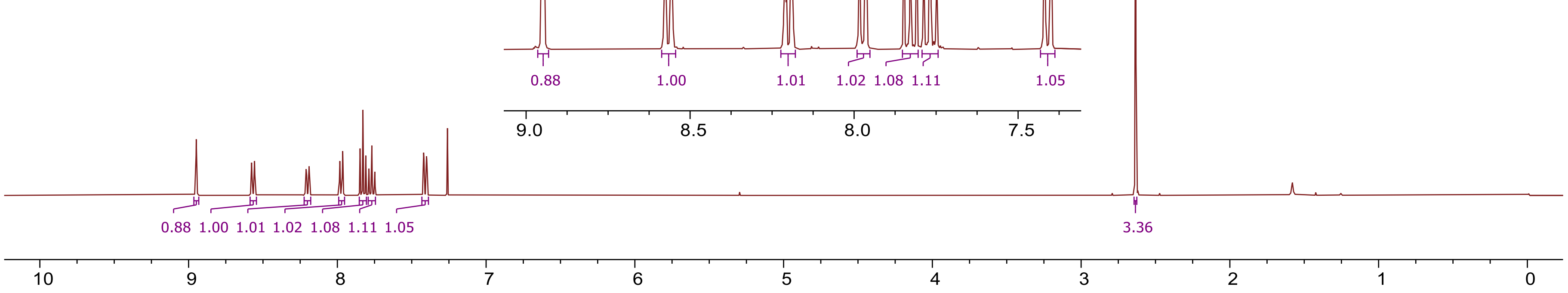

^{13}C NMR (100 MHz, $CDCl_3$):

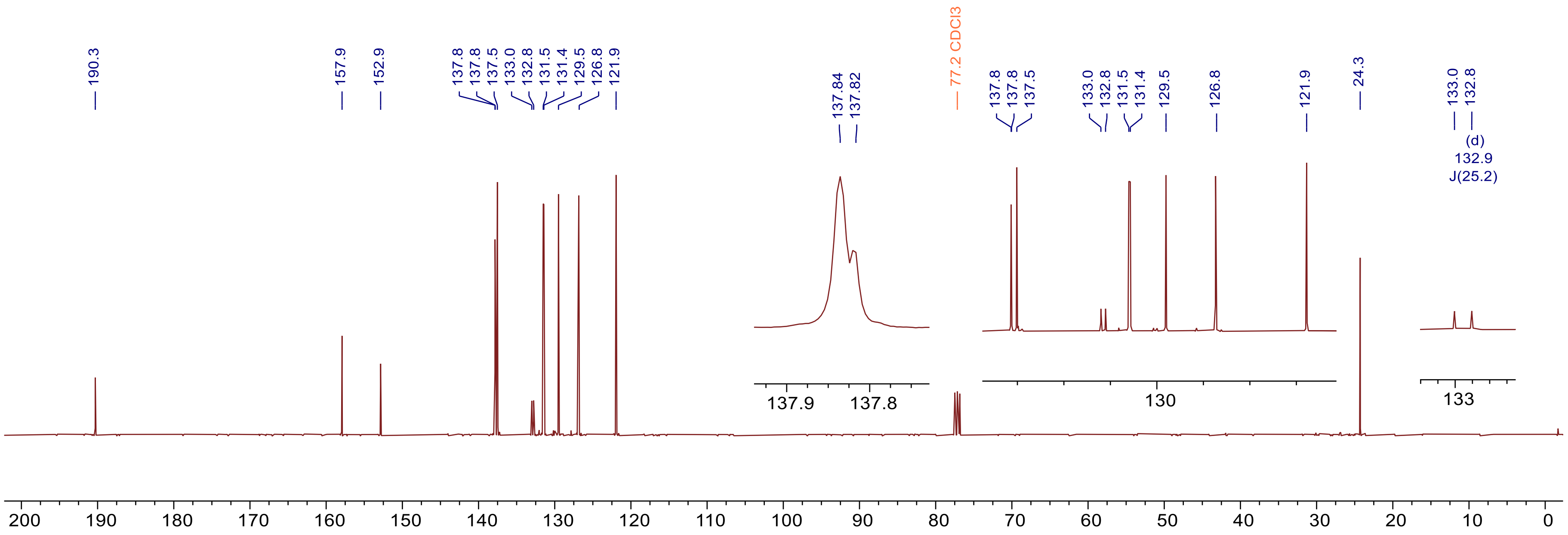

^{19}F NMR (376 MHz, $CDCl_3$):

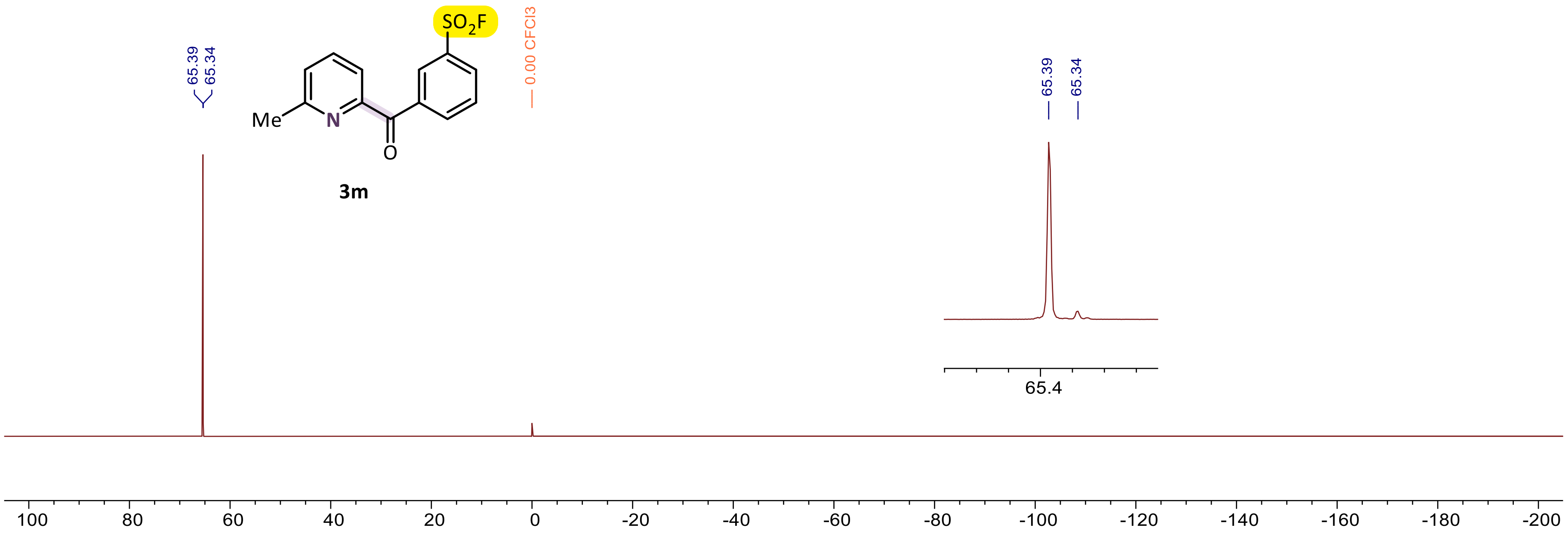

3m

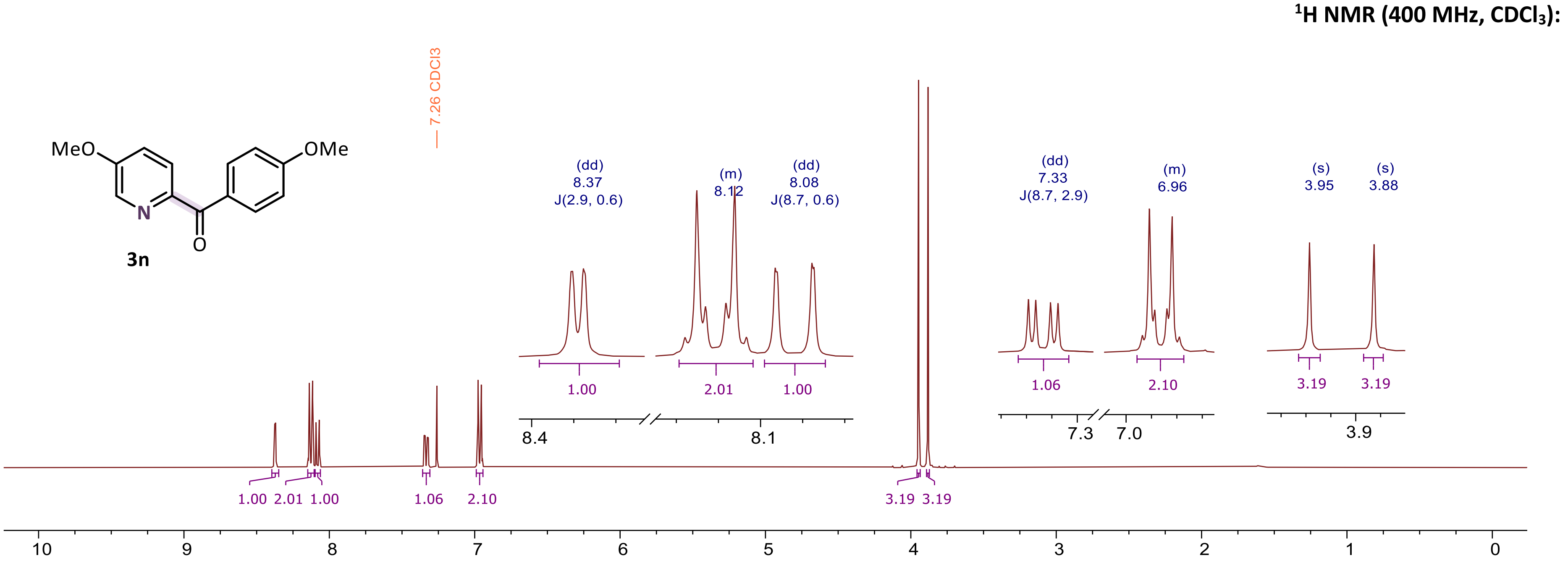

^{1}H NMR (400 MHz, CDCl$_3$):
MeO
OMe
N
O
3n
7.26 CDCl3
(dd) 8.37 J(2.9, 0.6)
(m) 8.12
(dd) 8.08 J(8.7, 0.6)
(dd) 7.33 J(8.7, 2.9)
(m) 6.96
(s) 3.95
(s) 3.88
1.00 2.01 1.00 1.06 2.10 3.19 3.19

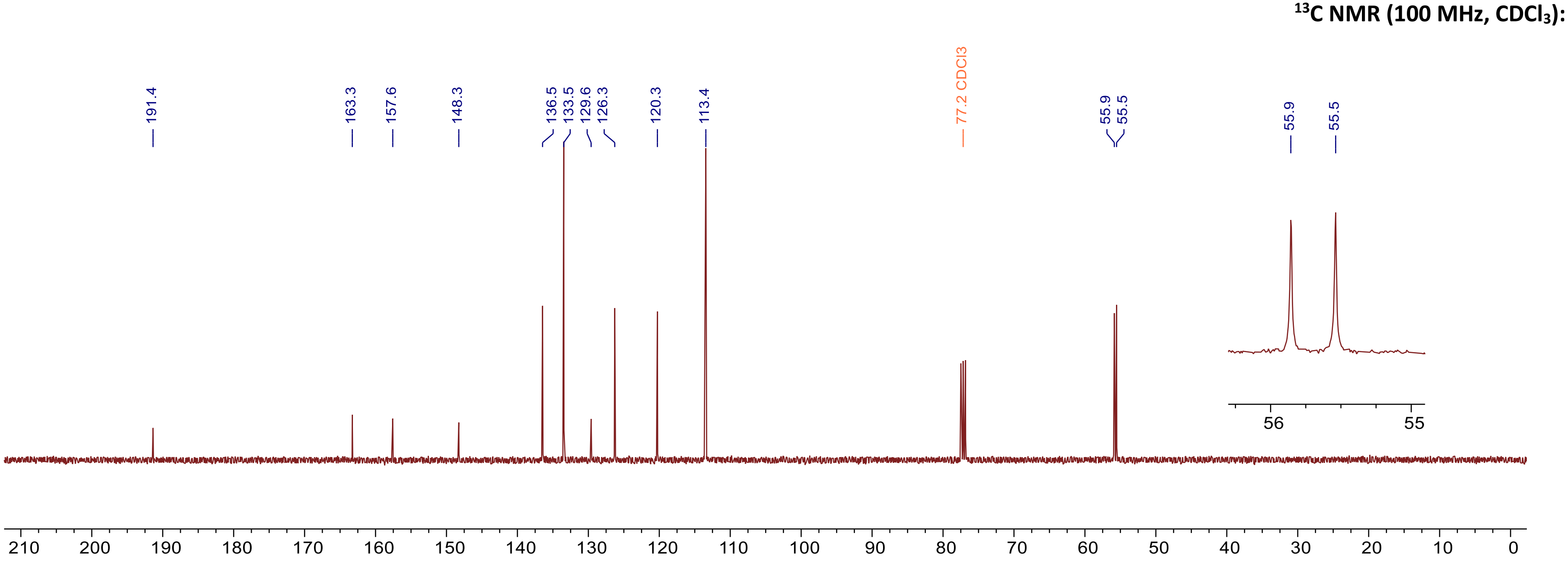

^{13}C NMR (100 MHz, CDCl$_3$):
191.4 163.3 157.6 148.3 136.5 133.5 129.6 126.3 120.3 113.4
77.2 CDCl3
55.9 55.5

^{1}H NMR (400 MHz, $CDCl_3$):

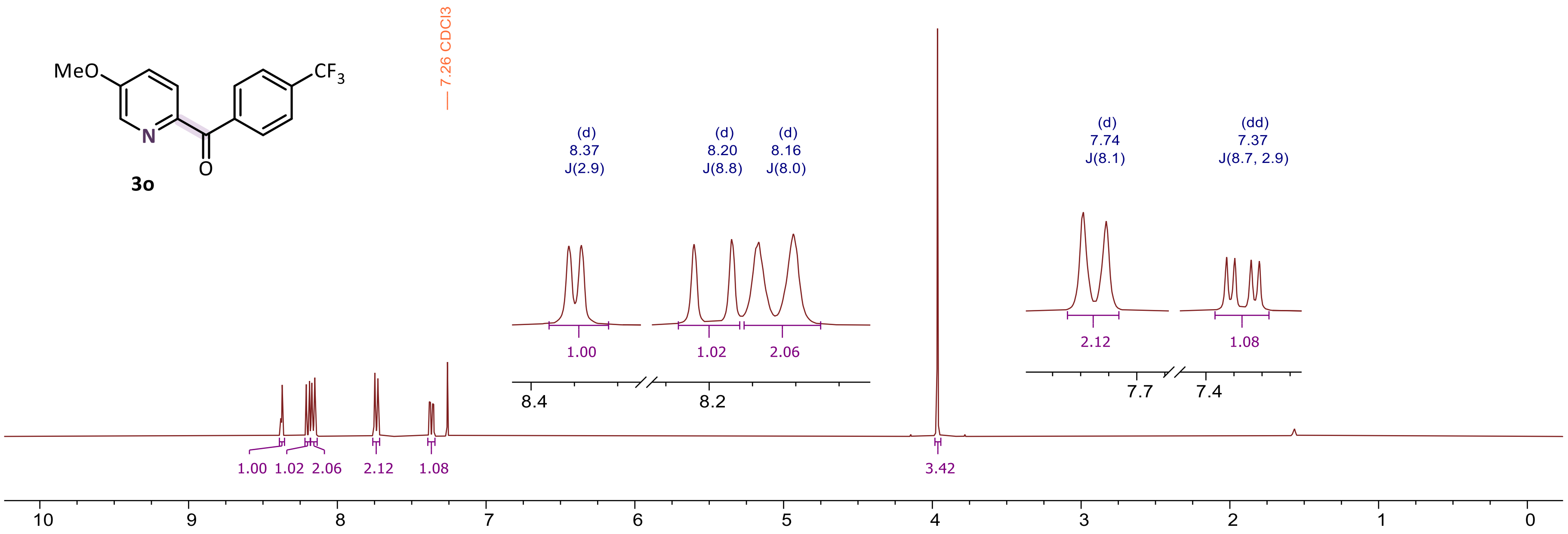

^{13}C NMR (100 MHz, $CDCl_3$):

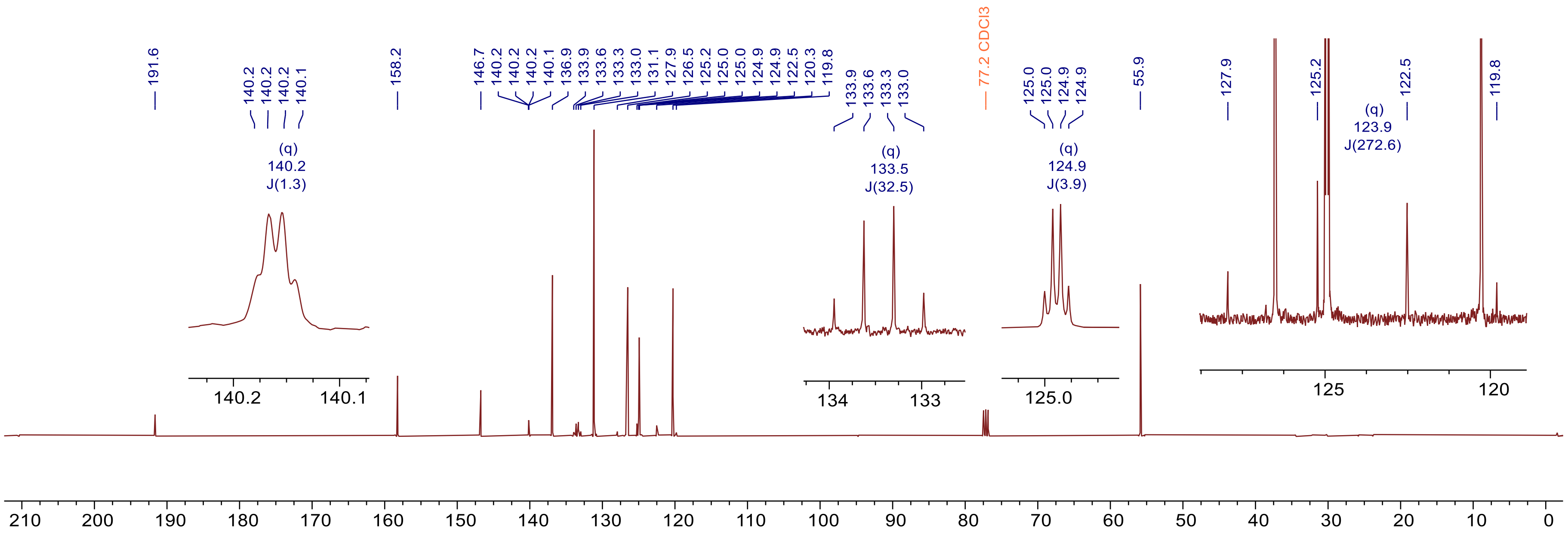

^{19}F NMR (376 MHz, $CDCl_3$):

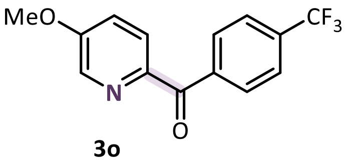

3o

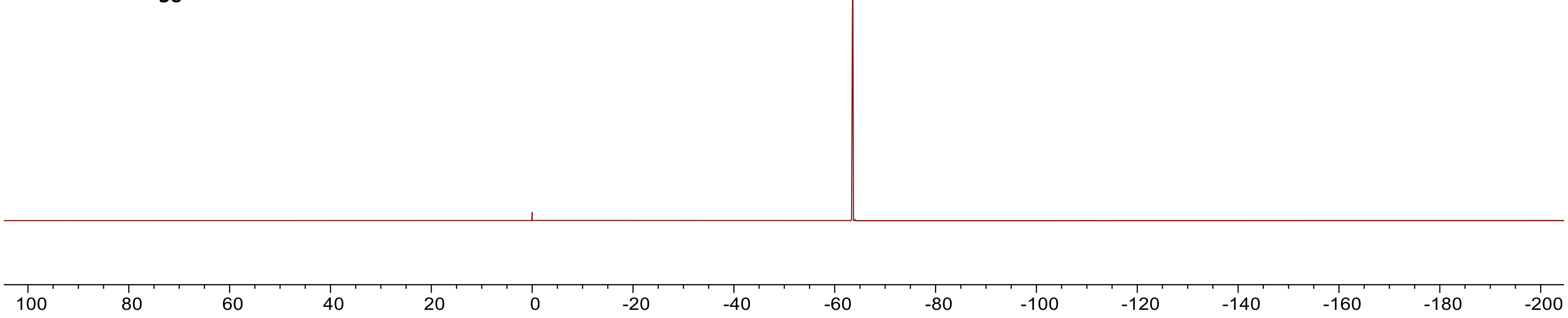

^{1}H NMR (400 MHz, $CDCl_3$):

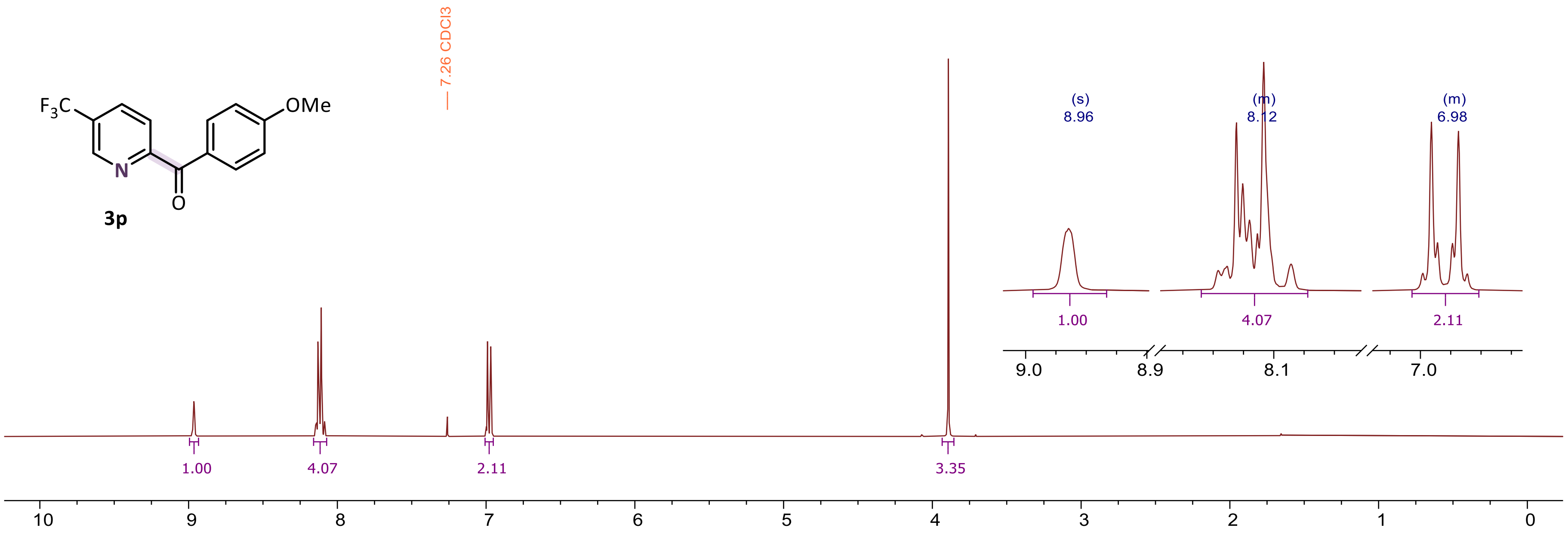

^{13}C NMR (100 MHz, $CDCl_3$):

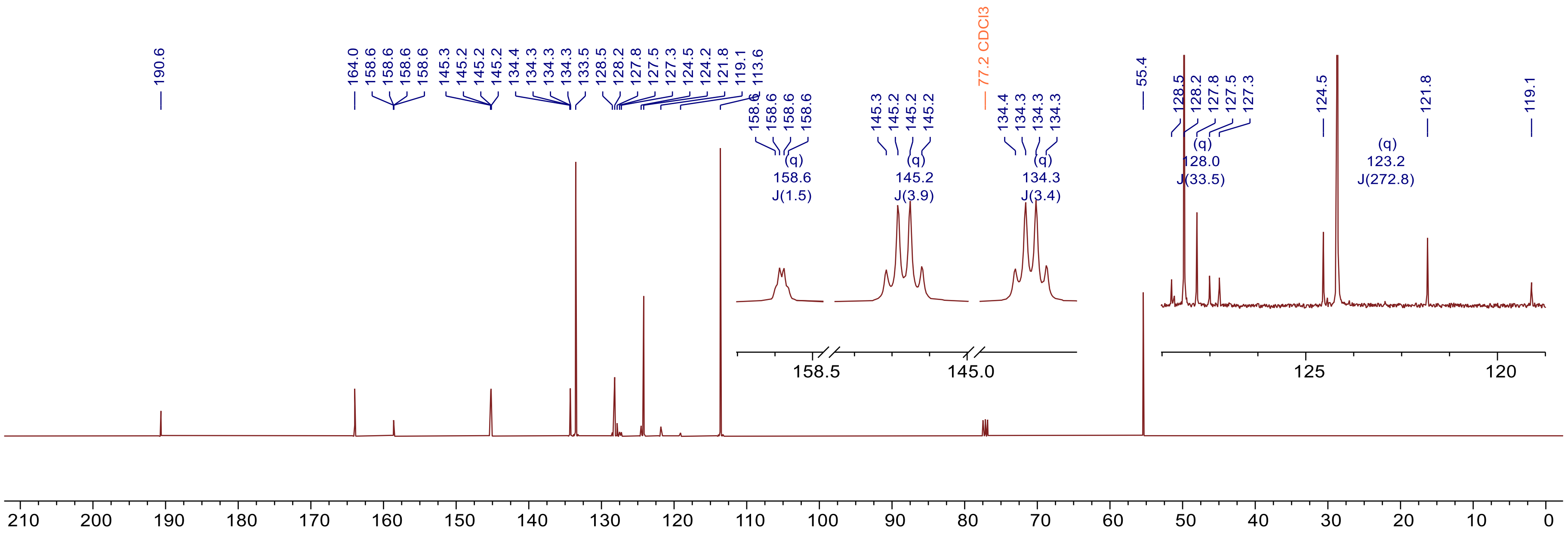

^{19}F NMR (376 MHz, $CDCl_3$):

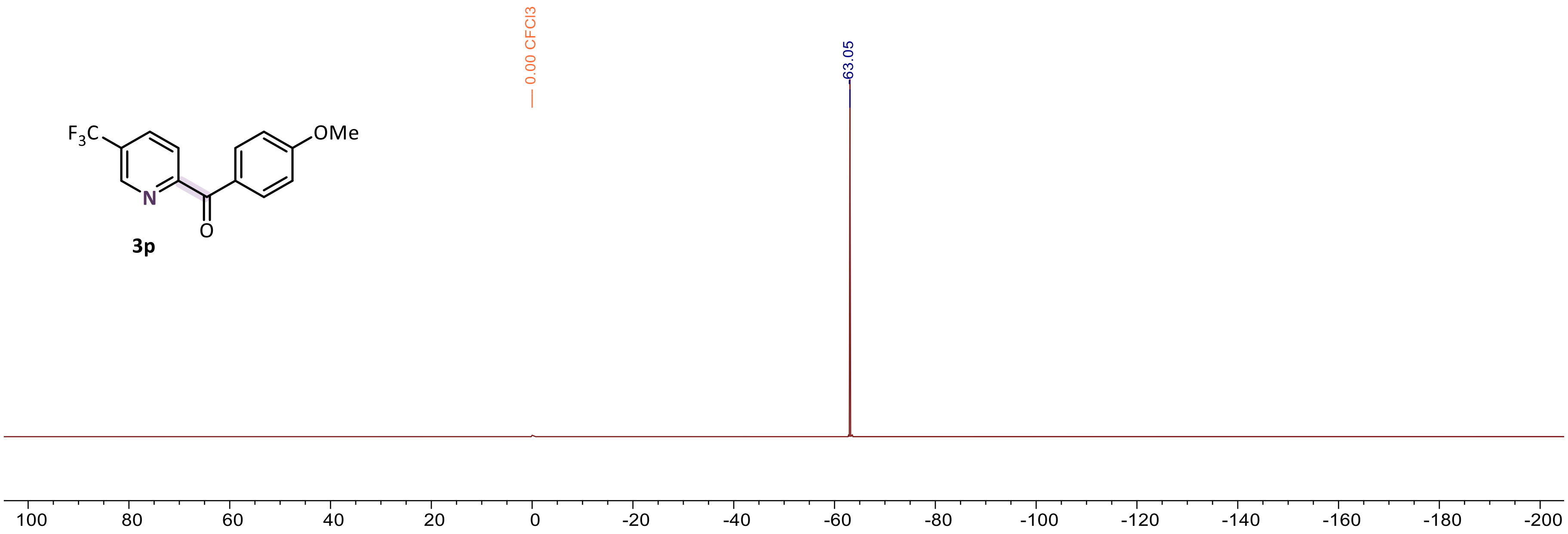

^{1}H NMR (400 MHz, $CDCl_3$):

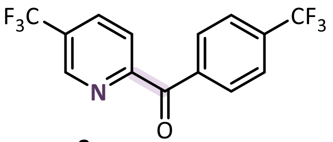

3q

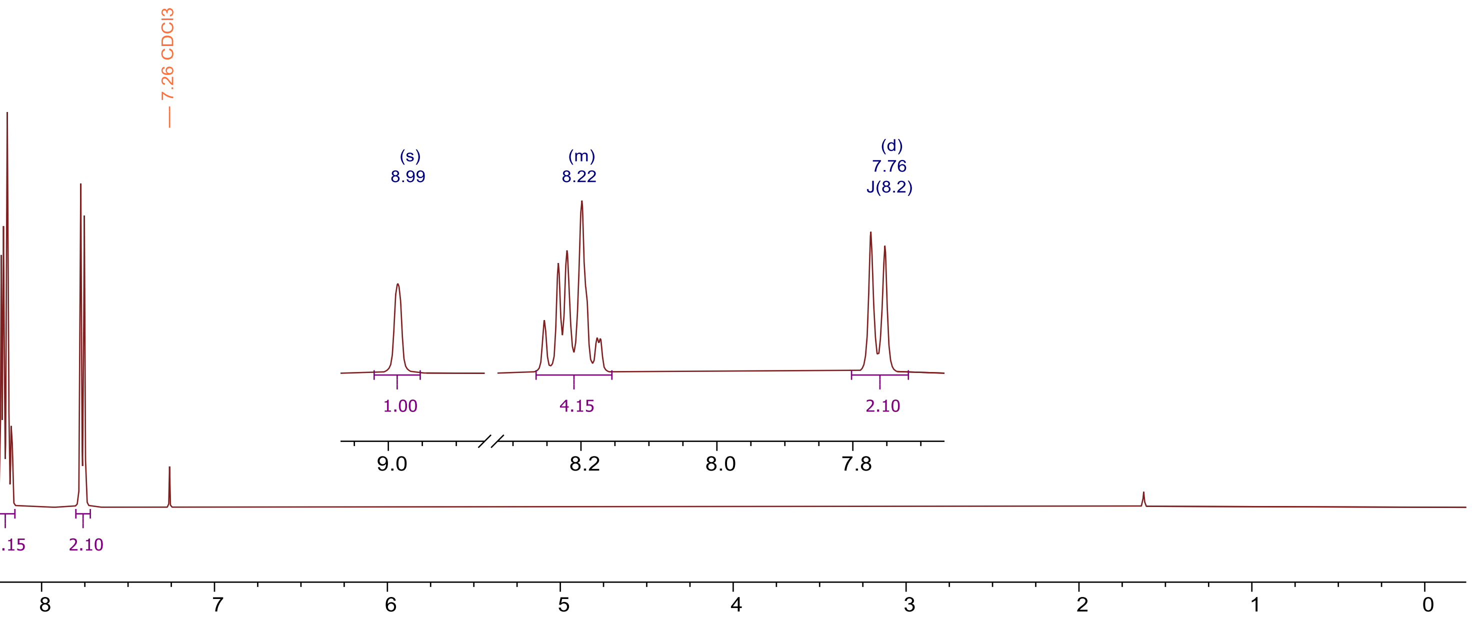

^{13}C NMR (100 MHz, $CDCl_3$):

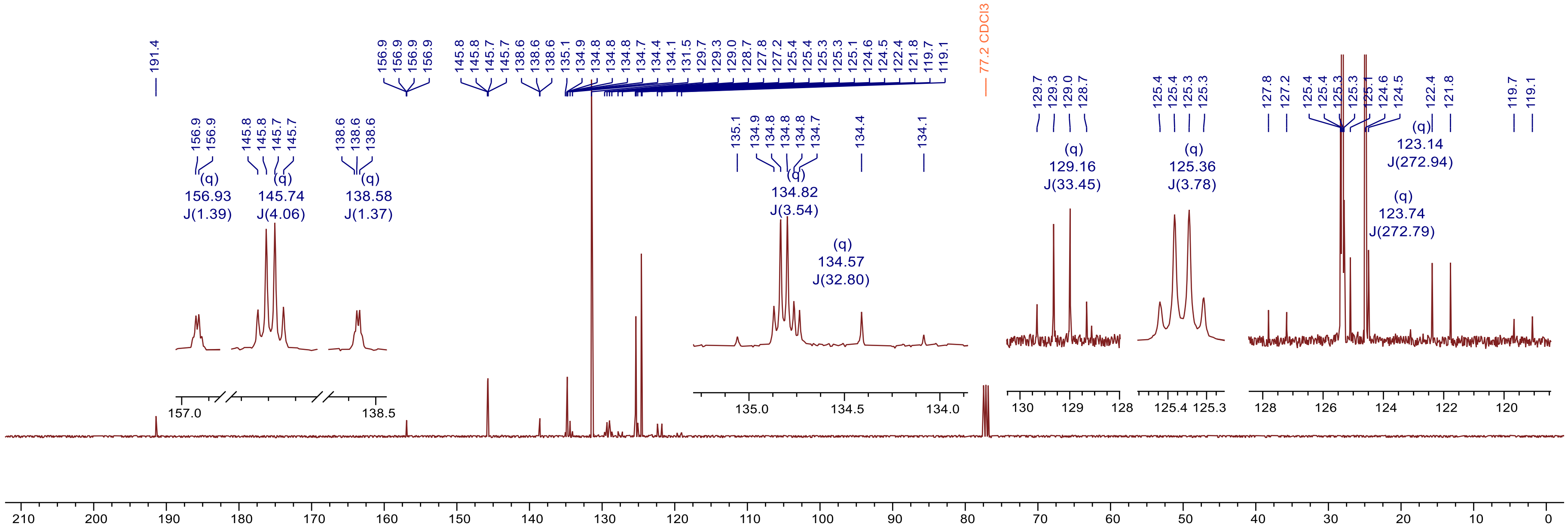

^{19}F NMR (376 MHz, $CDCl_3$):

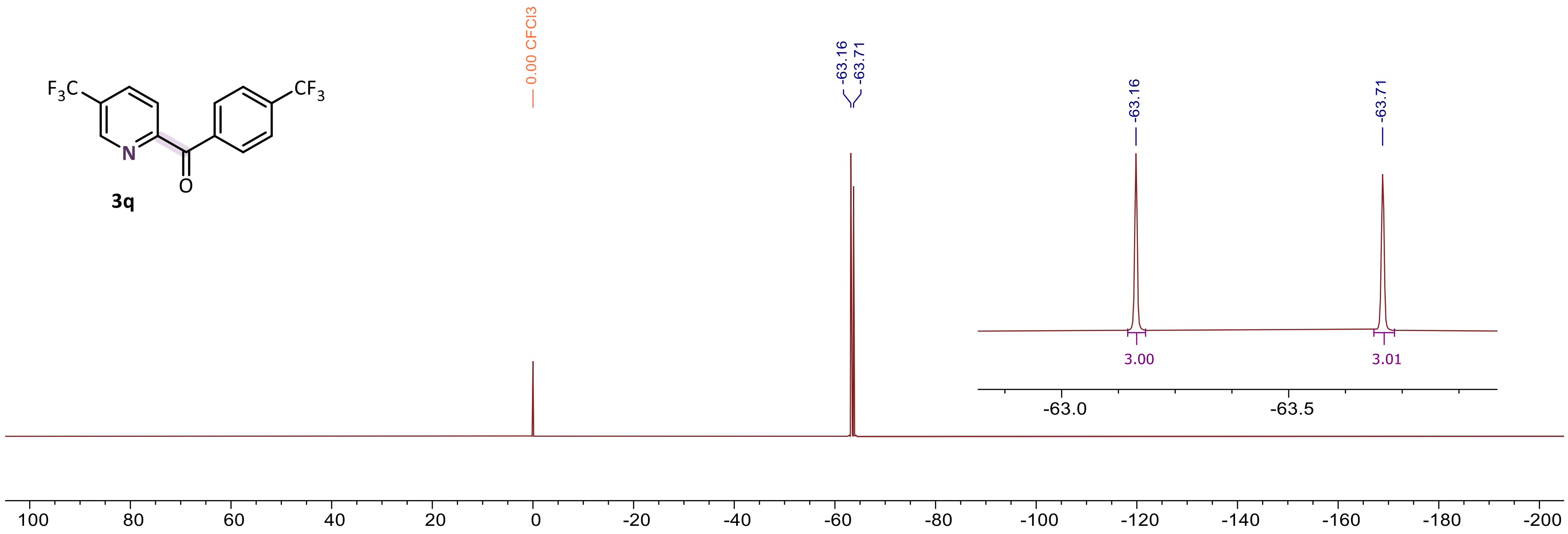

3q

^{1}H NMR (400 MHz, $CDCl_3$):

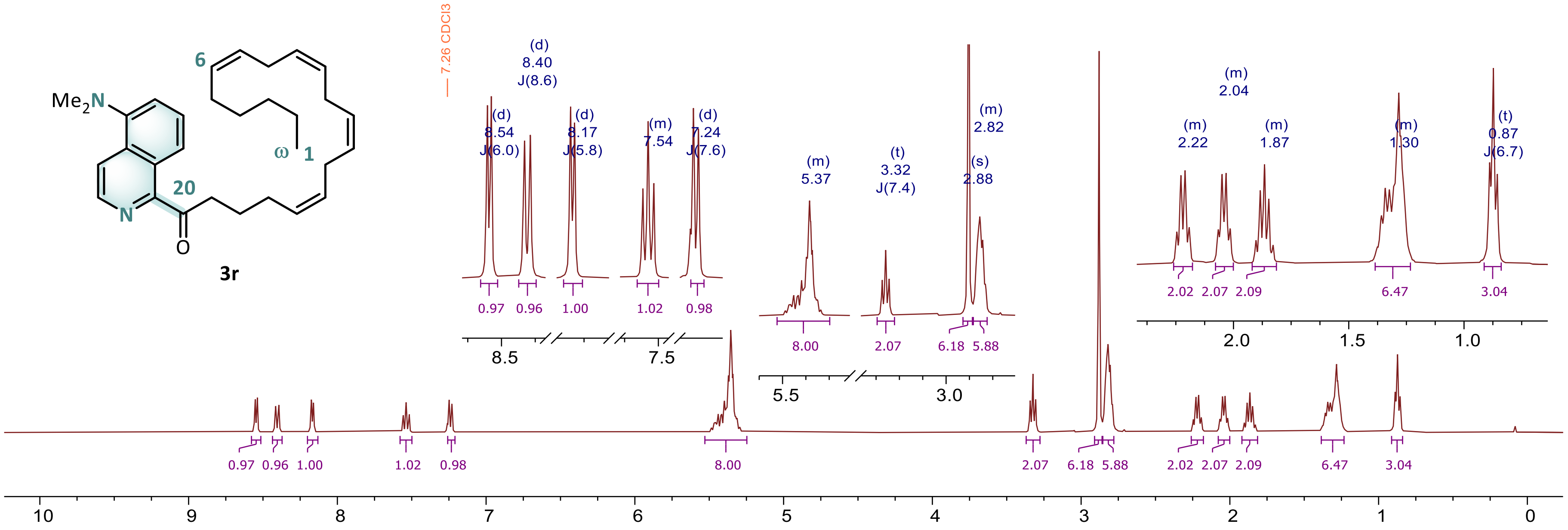

^{13}C NMR (100 MHz, $CDCl_3$):

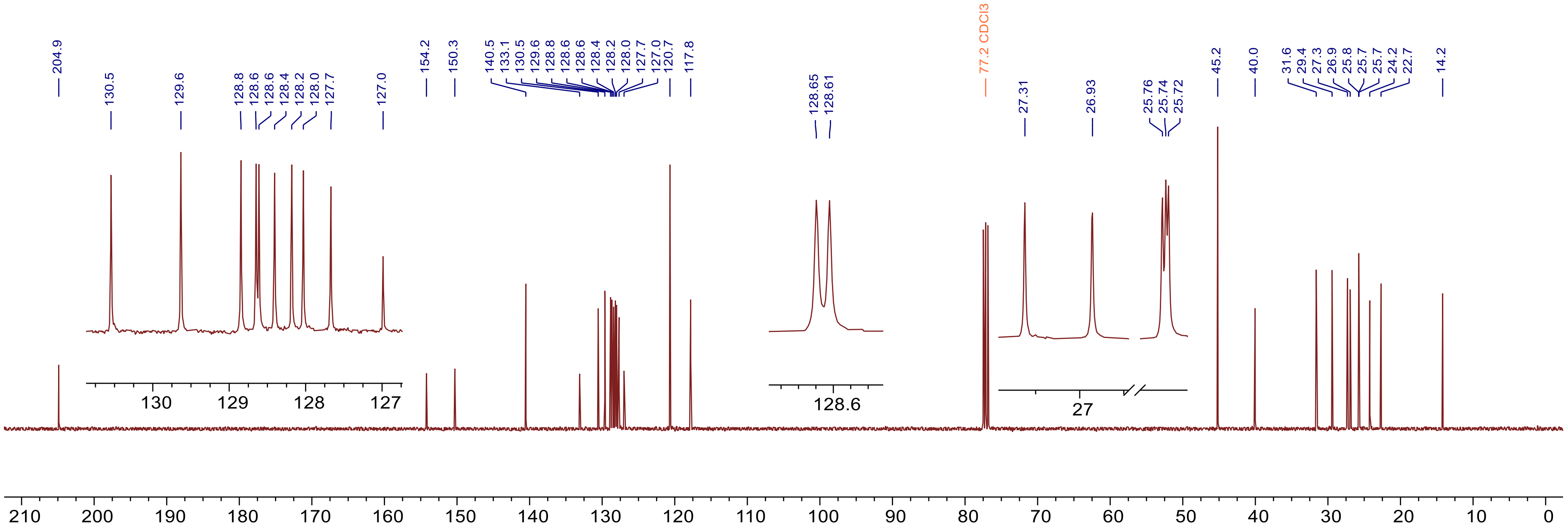

^{1}H NMR (400 MHz, $CDCl_3$):

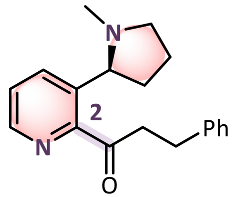

3s

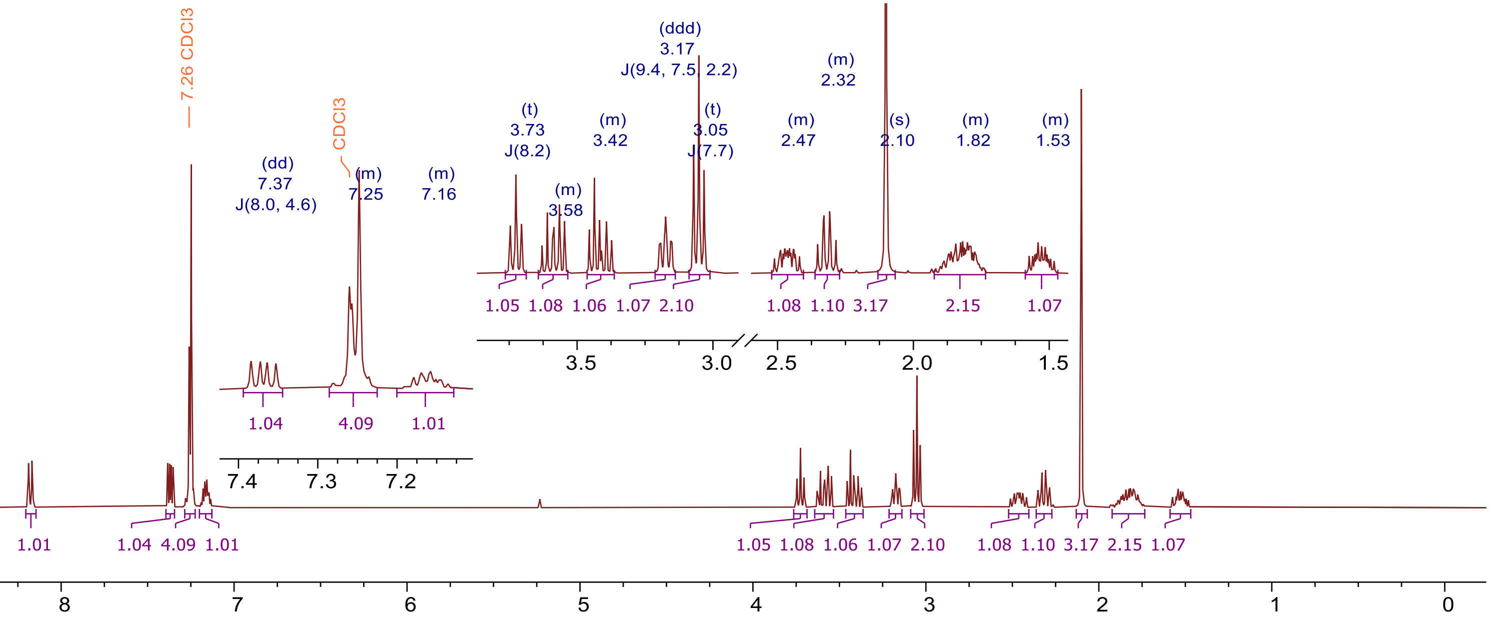

^{13}C NMR (100 MHz, $CDCl_3$):

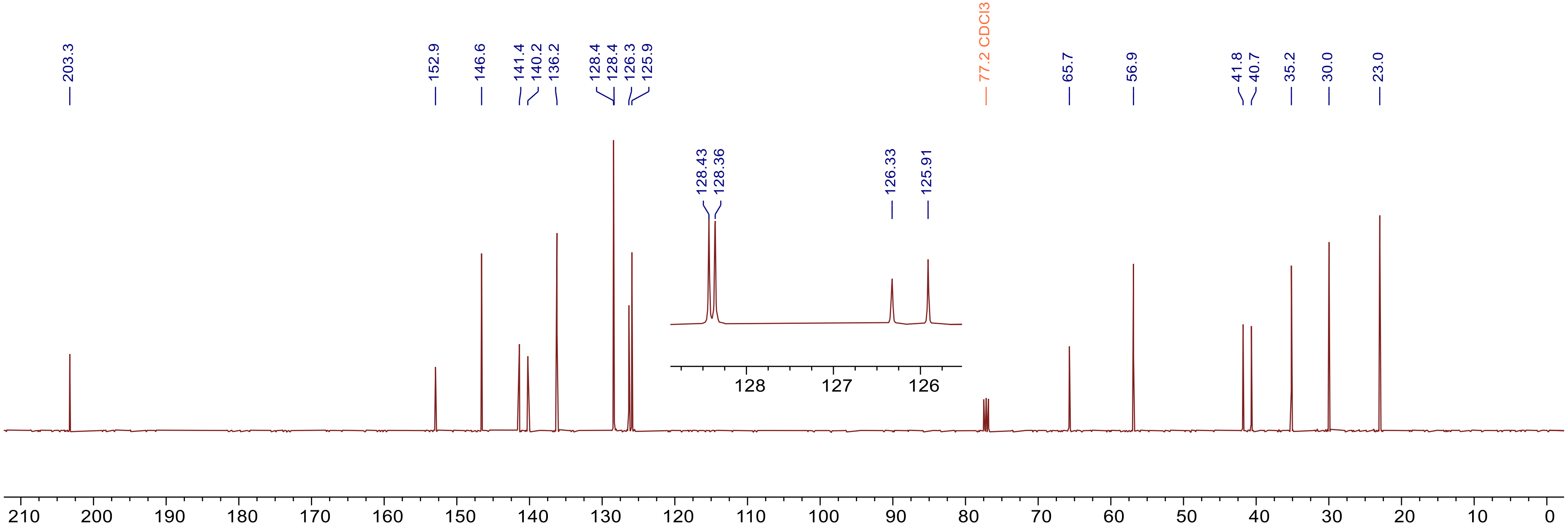

^{1}H NMR (400 MHz, $CDCl_3$):

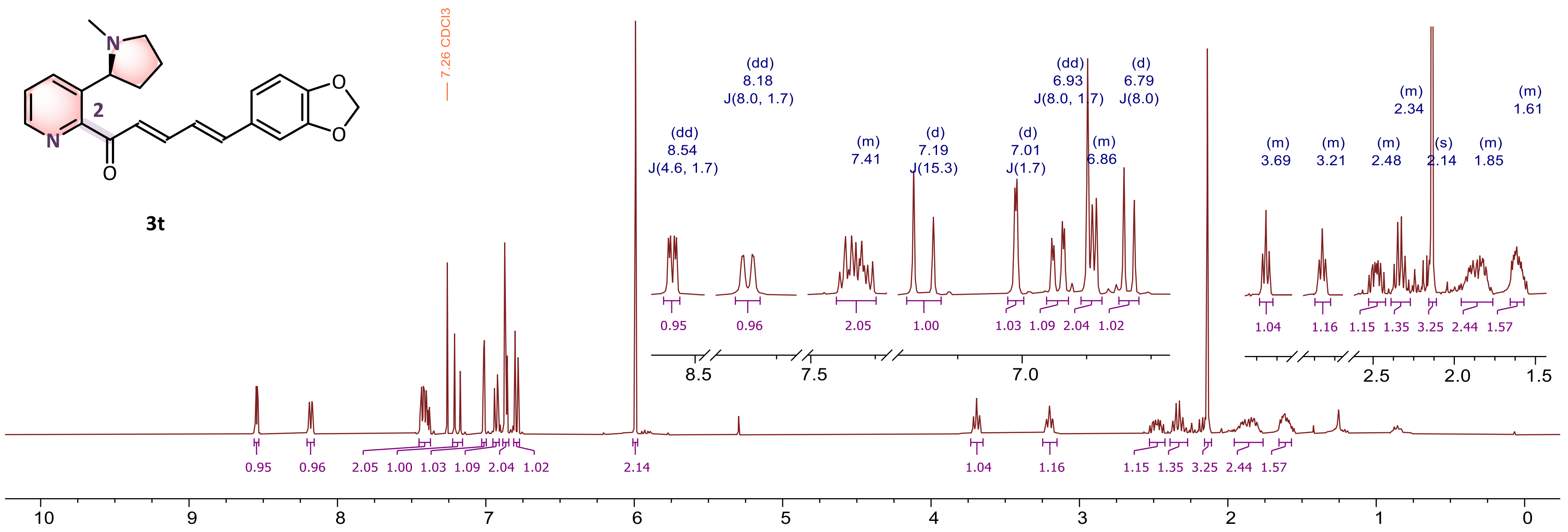

3t

^{13}C NMR (100 MHz, $CDCl_3$):

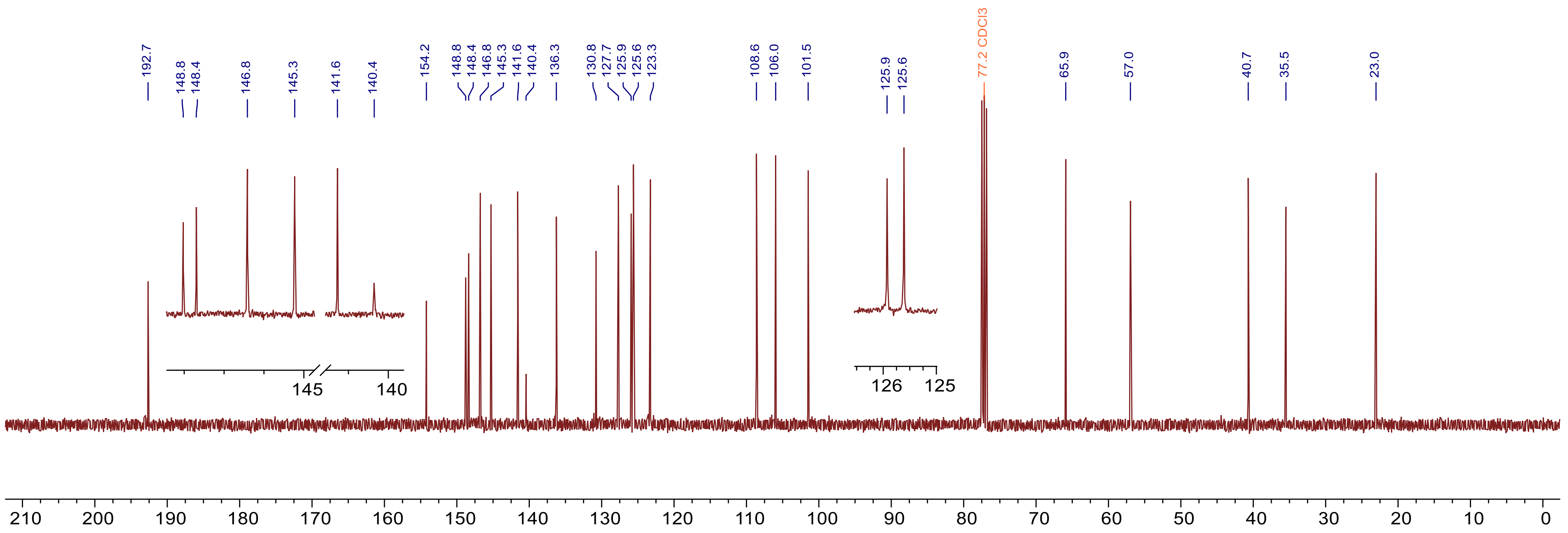

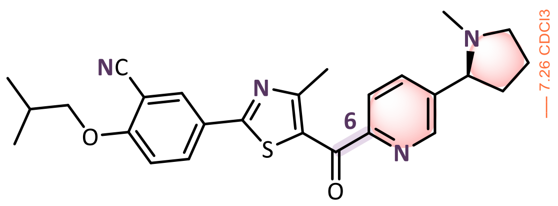

3u

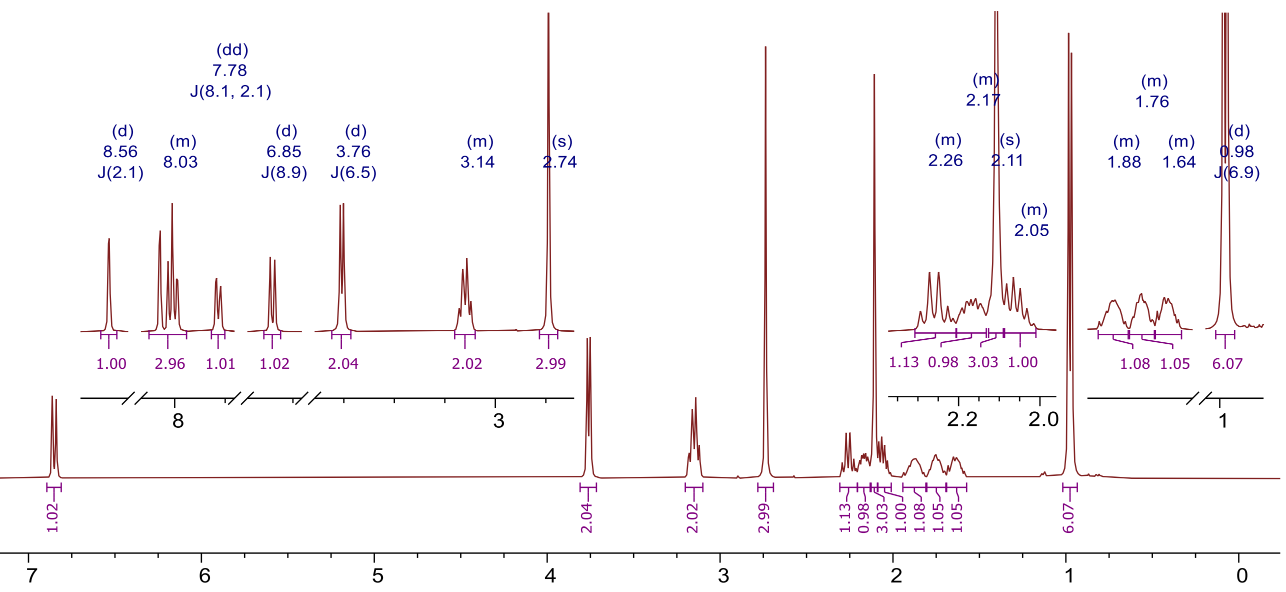

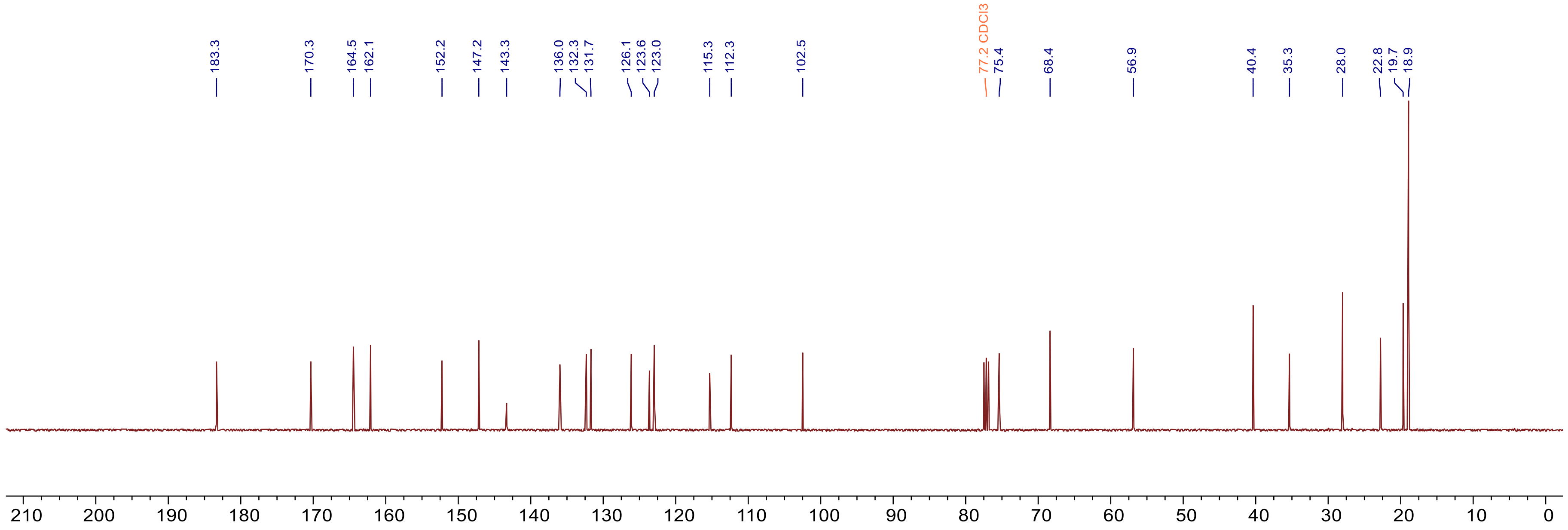

^{1}H NMR (400 MHz, $CDCl_3$):

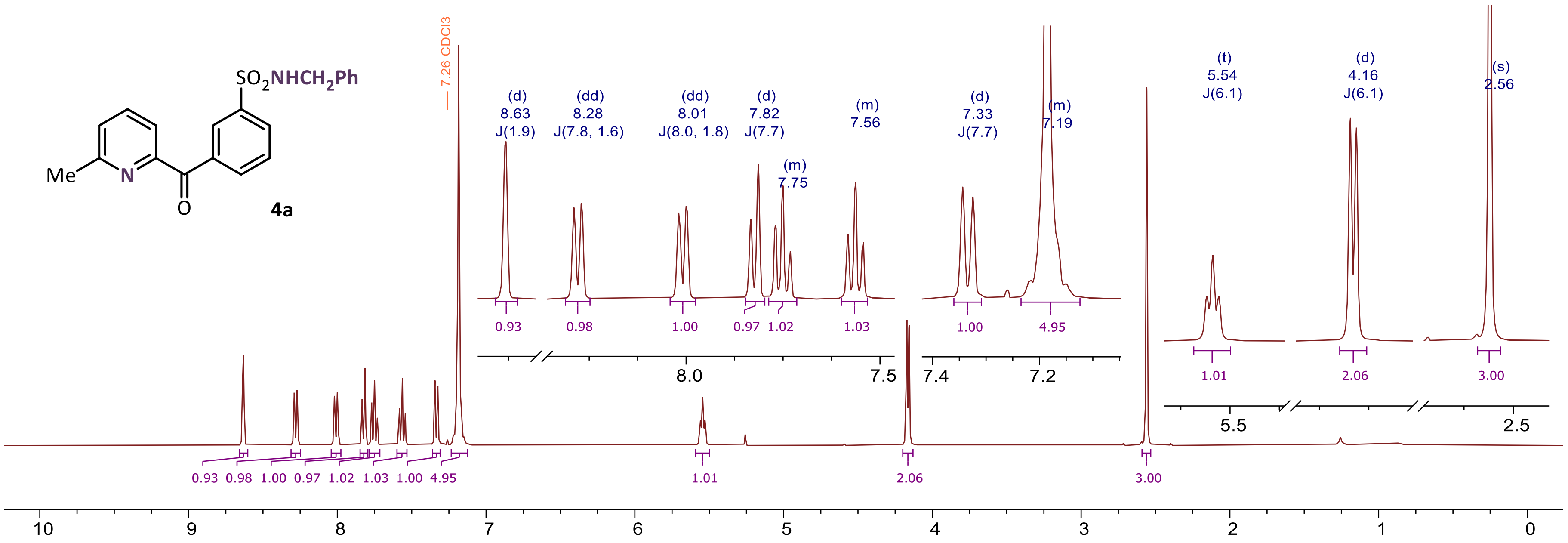

^{13}C NMR (100 MHz, $CDCl_3$):

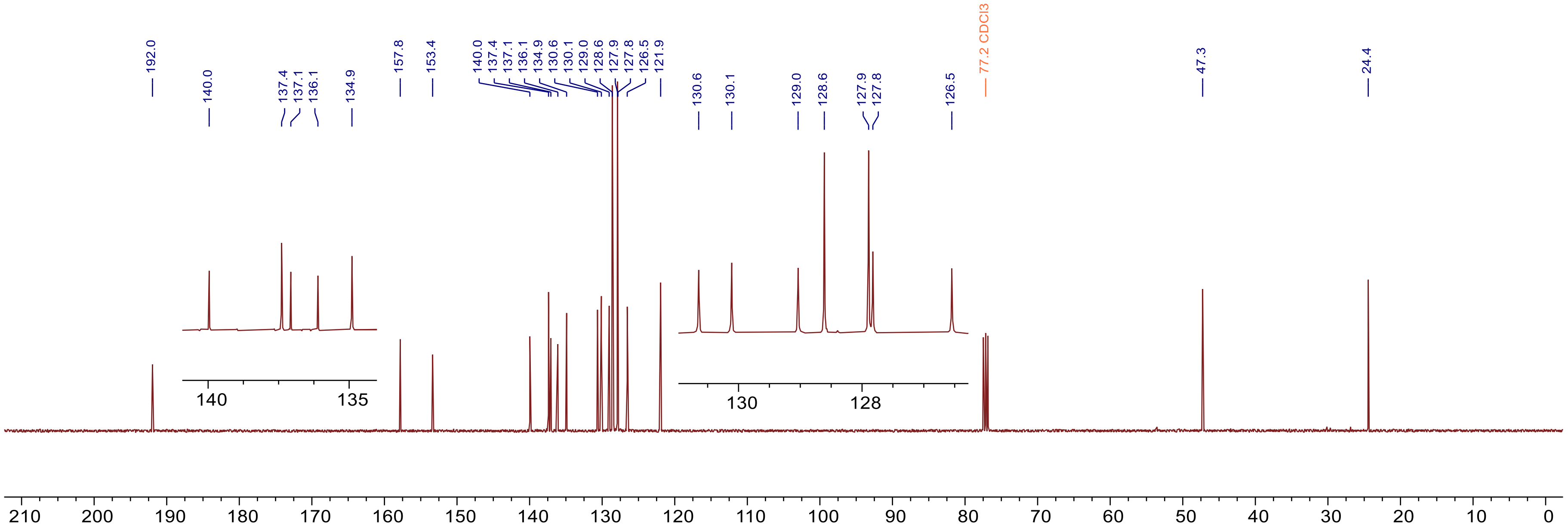

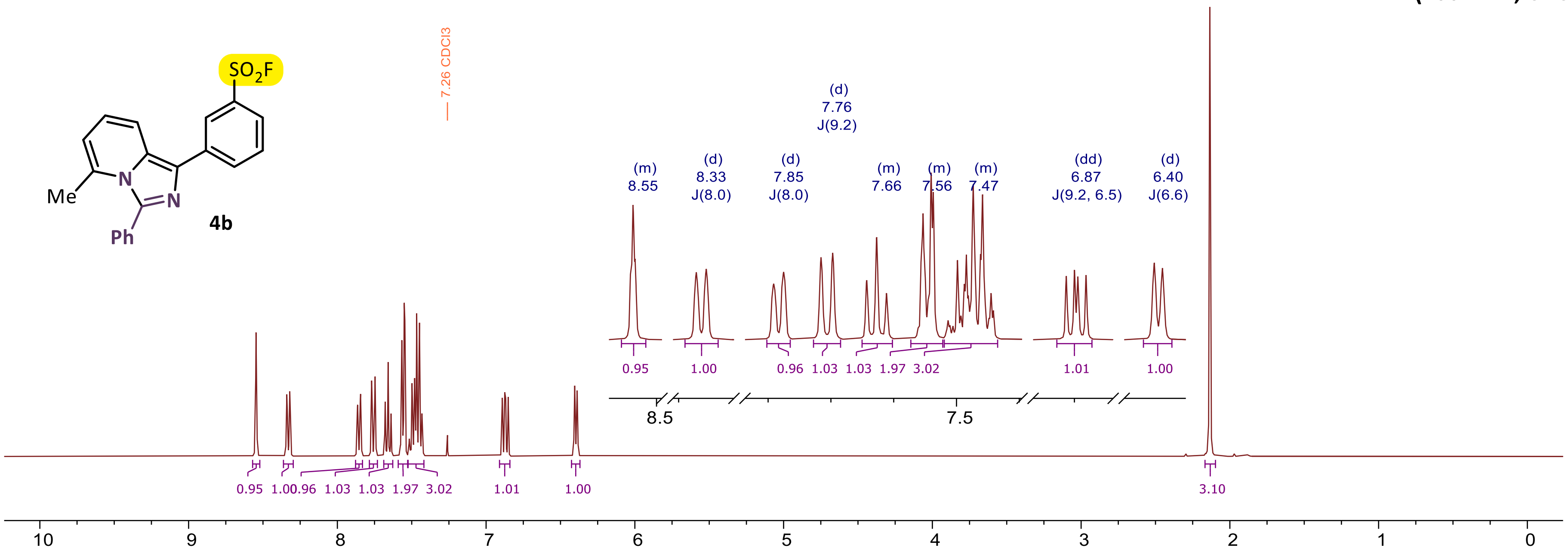

4b

^{1}H NMR (400 MHz, $CDCl_3$):

^{13}C NMR (100 MHz, $CDCl_3$):

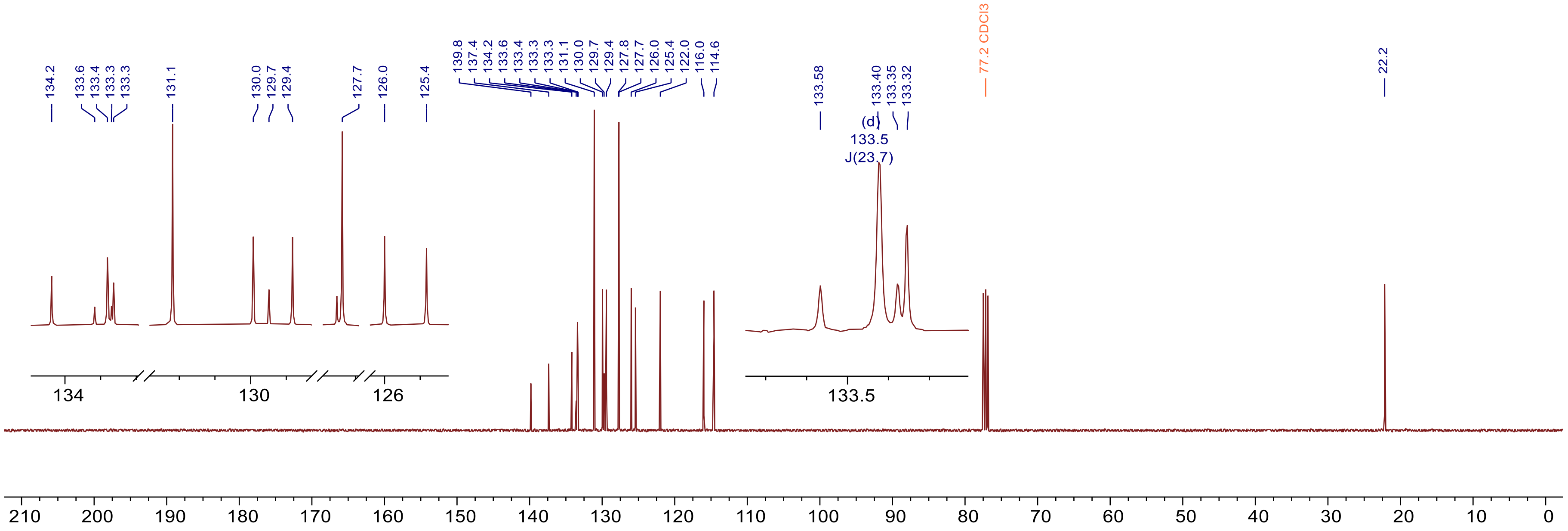

^{19}F NMR (376 MHz, $CDCl_3$):

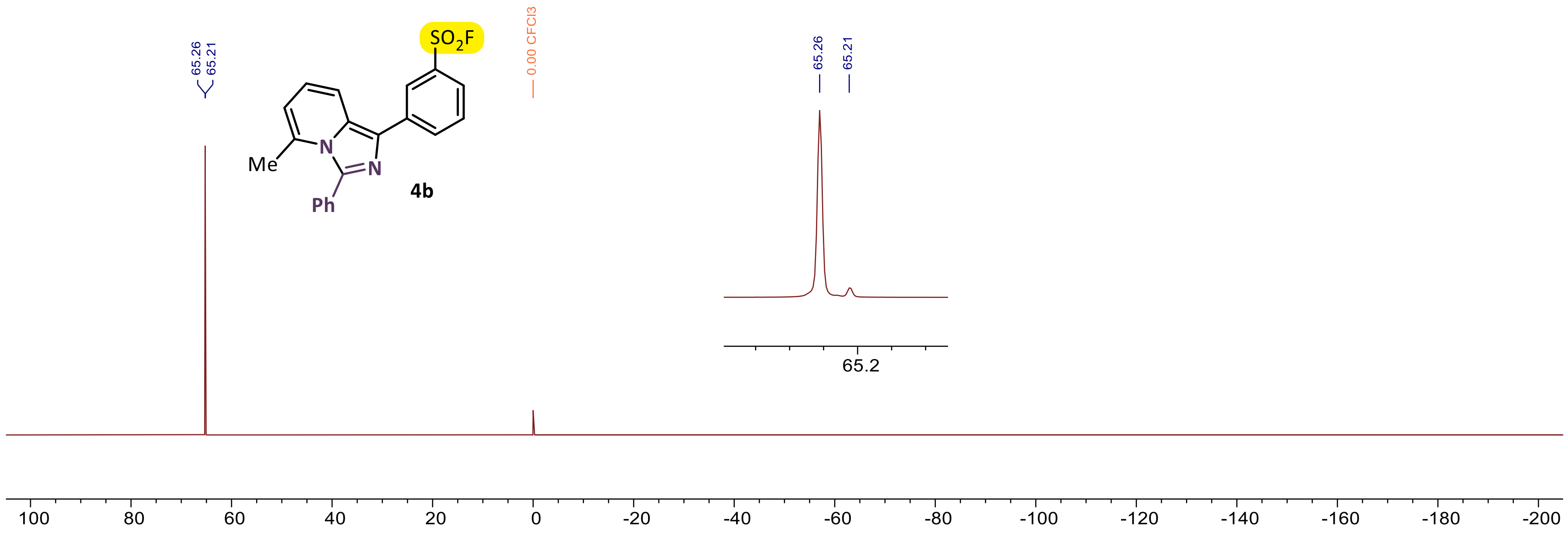

^{1}H NMR (400 MHz, $CDCl_3$):

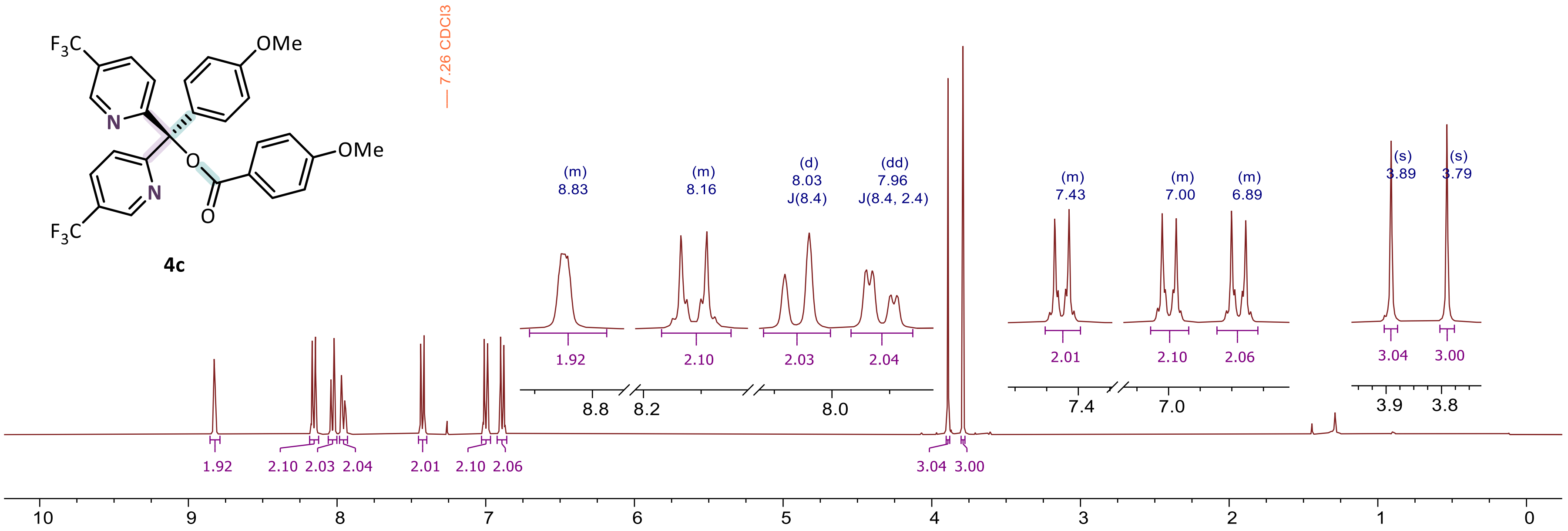

^{13}C NMR (100 MHz, $CDCl_3$):

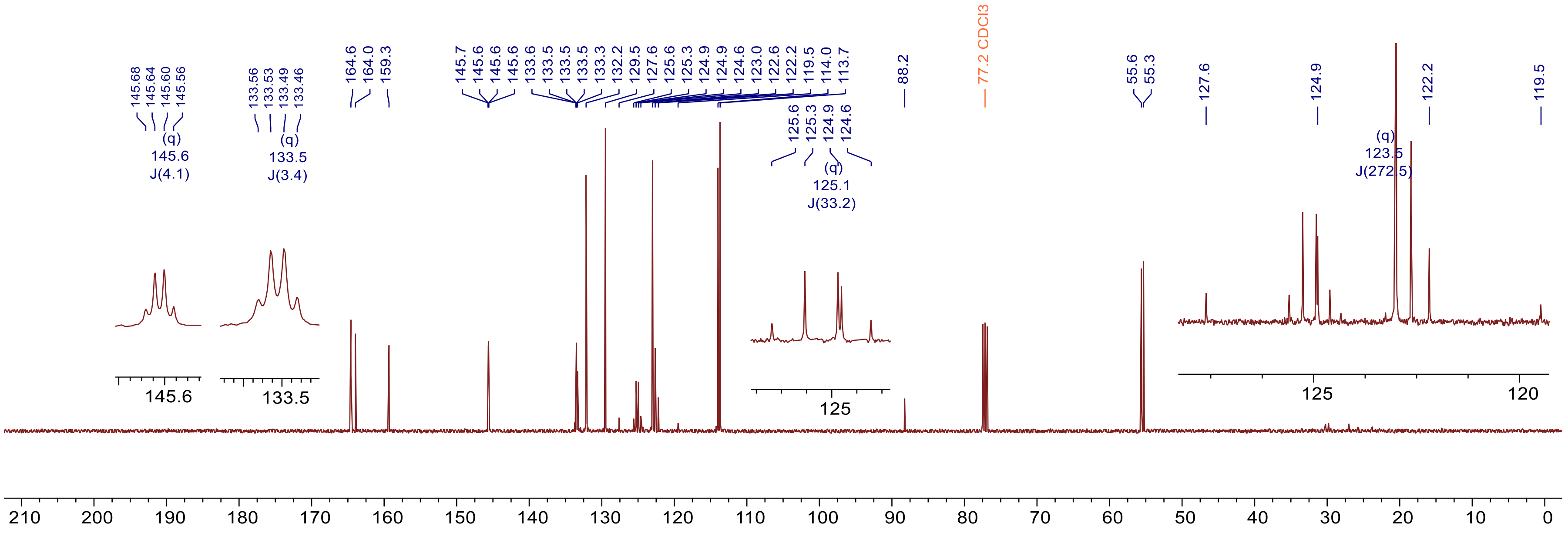

^{19}F NMR (376 MHz, $CDCl_3$):

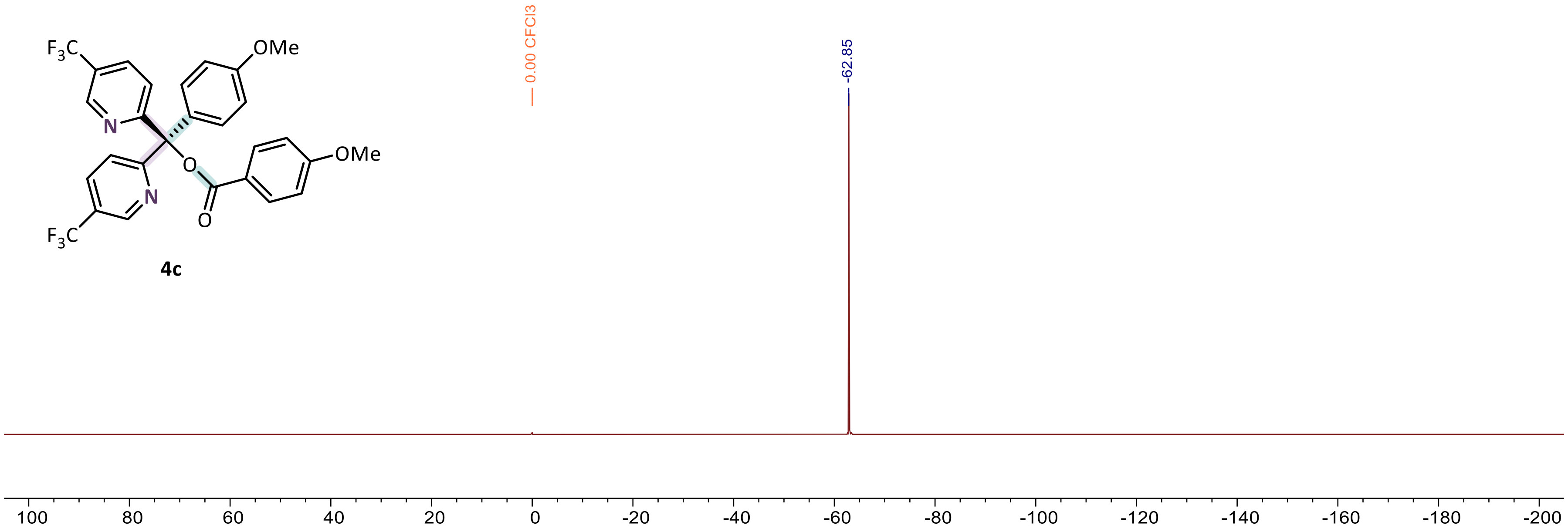

^1H NMR (400 MHz, $CDCl_3$):

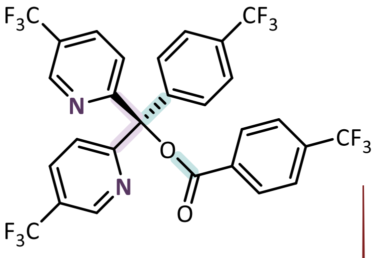

4d

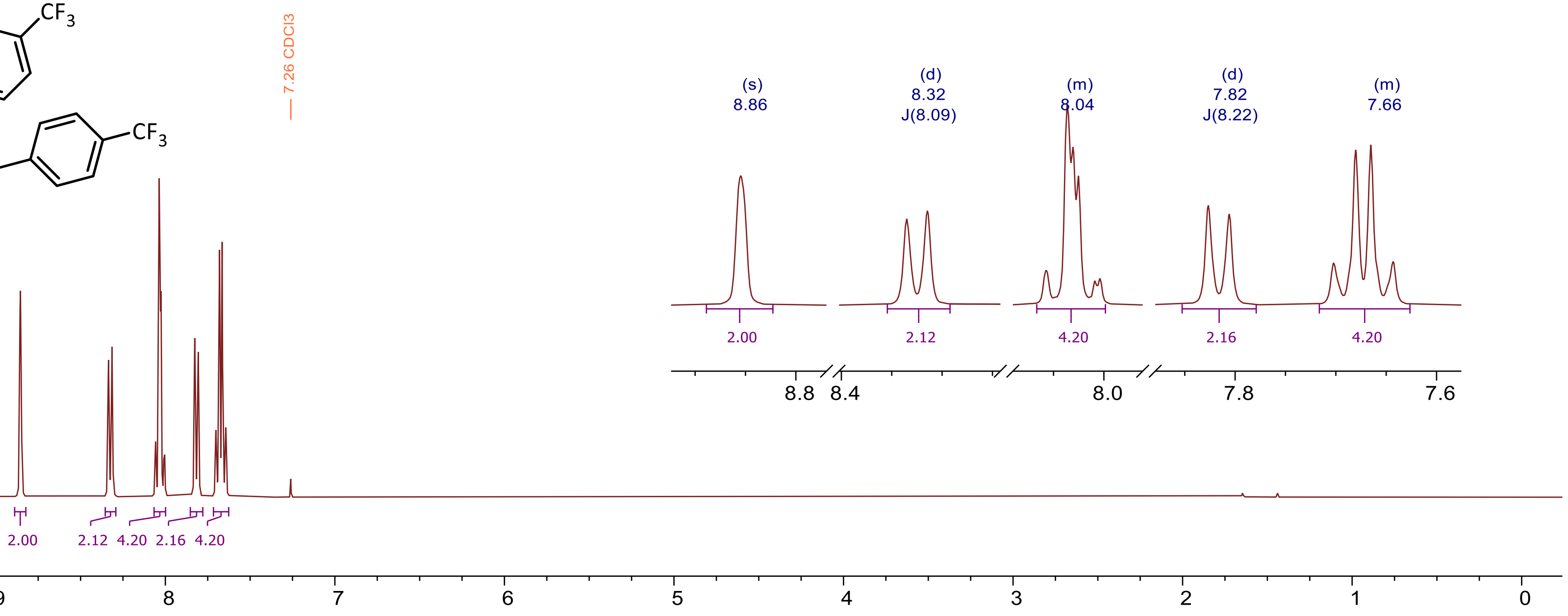

^{19}F NMR (376 MHz, $CDCl_3$):

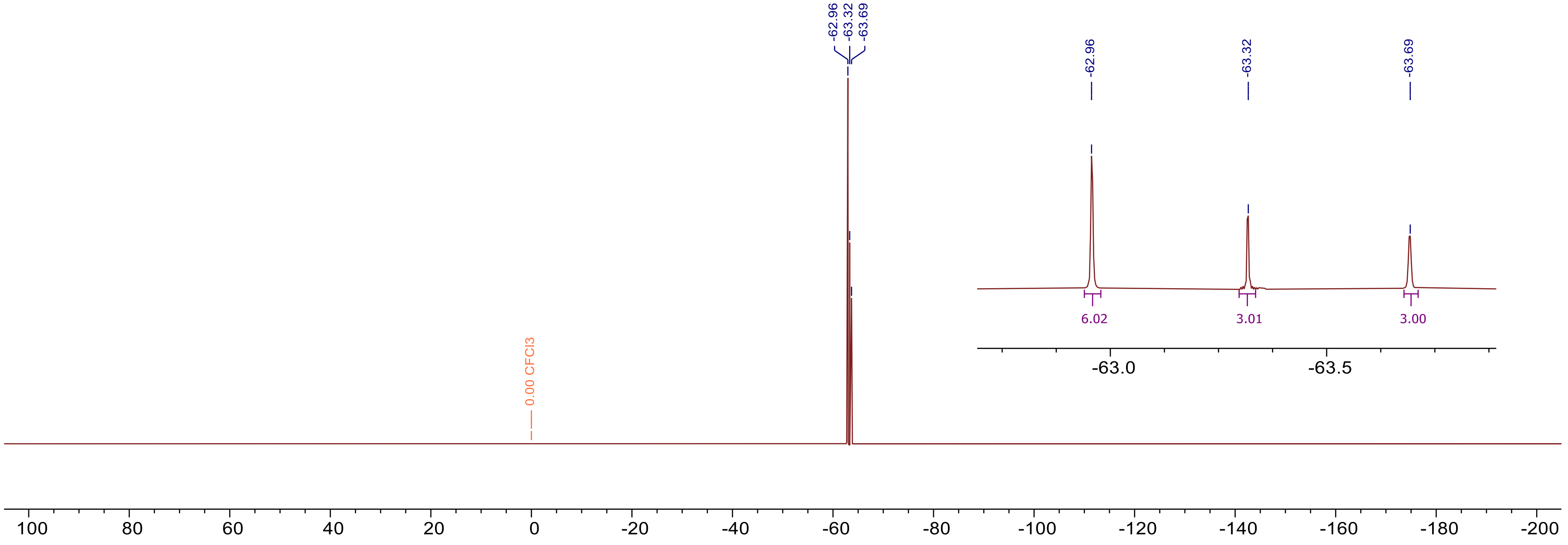

^{13}C NMR (100 MHz, $CDCl_3$):

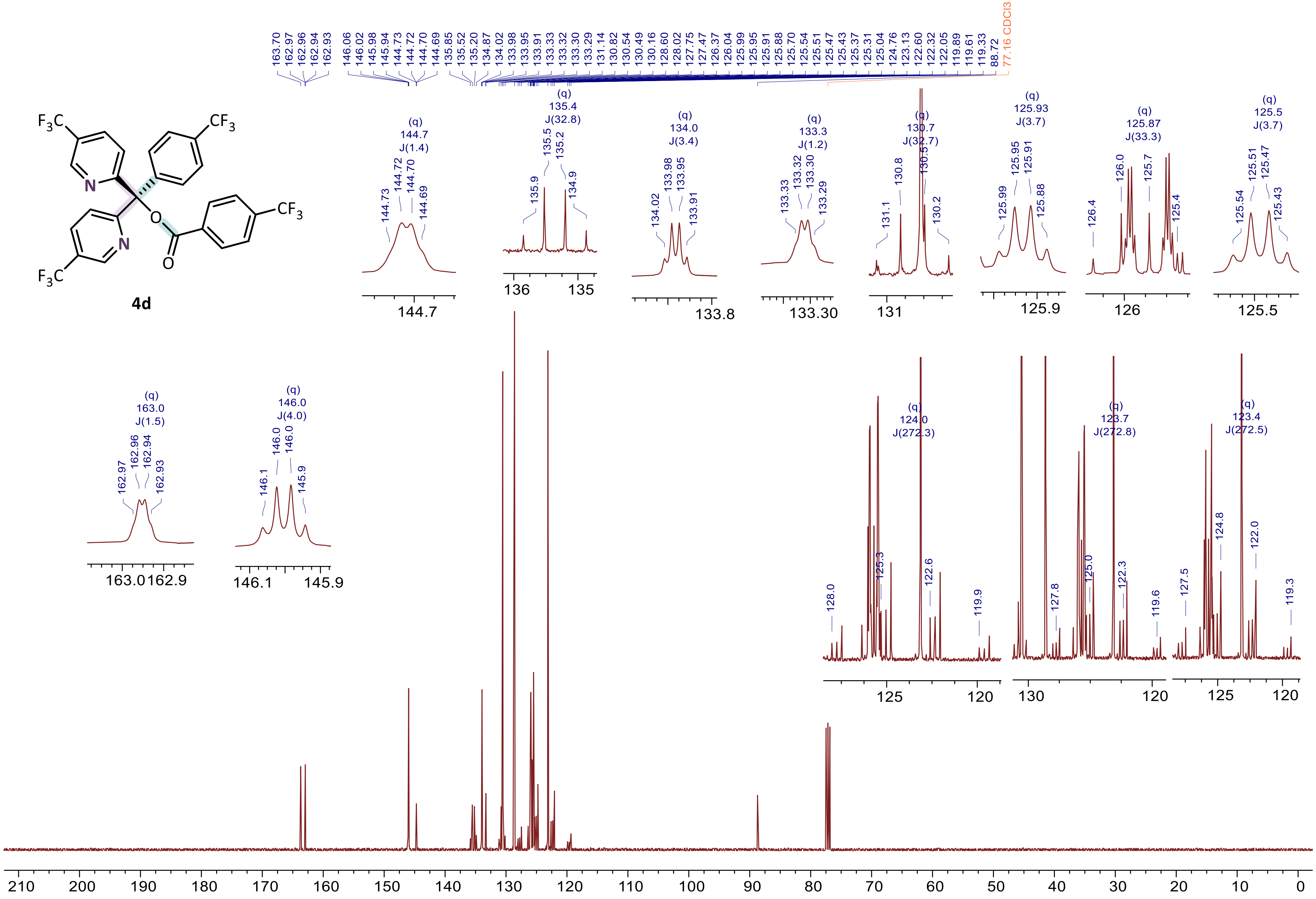

Supplement: Supplementary file 1 [file ol5c03140_si_001.pdf]
